# Systematic analysis of spontaneous tandem genome amplification events in *Yersinia pestis*

**DOI:** 10.1371/journal.pone.0338460

**Published:** 2025-12-31

**Authors:** Dmitry N. Konanov, Olga N. Liubimova, Alexander V. Kovrizhnikov, Ignat V. Sonets, Alina N. Balykova, Alexandra V. Lukina-Gronskaya, Anna S. Speranskaya, Danil V. Krivonos, Galina A. Eroshenko, Elena N. Ilina, Vadim M. Govorun, Vladimir V. Kutyrev

**Affiliations:** 1 Research Institute for System Biology and Medicine of Rospotrebnadzor, Moscow, Russia; 2 Moscow Center for Advanced Studies, Moscow, Russia; 3 Russian Research Anti-Plague Institute “Microbe”, Saratov, Russia; Universidad Nacional de Colombia Campus Palmira, COLOMBIA

## Abstract

Tandem amplification of genomic fragments is quite common in bacteria growing under stress conditions, while spontaneous genome amplification events are rare, unstable and generally poorly described. Plague pathogen *Yersinia pestis* is a unique microorganism that contains an enormous number of short repeat sequences in its genome and as a result is very prone to spontaneous genome rearrangements including large tandem genome amplification events. Eleven *Y. pestis* strains sequenced during this study and more than thousand read archives from SRA were analyzed in this study. It was shown that genomes of more than half of *Y. pestis* laboratory isolates contain tandem repeats. They are mainly caused by the presence of multicopy IS-elements but a few of them are associated with multicopy rRNA clusters, so the rearrangement mechanism is most likely RecA-dependent recombination. Four regions with unstable copy number reproduced between different bioprojects were found. One of them was identified as an integrative mobilizable element carrying a probably incomplete Type 4 secretion system. More interesting, two other reproducible regions were not identified as mobile elements but had the length and GC-content almost identical to the length and GC-content of pMT1 and pCD1 plasmids.

## 1. Introduction

Tandem genome amplifications are recombination events quite common in bacteria, usually observed in bacterial populations cultivated under stress conditions, such as antibiotic action, oxidative stress, action of heavy metals, and so on [1]. In nature, spontaneous large tandem amplification events are not so rare but usually unstable, probably due to their negative effect on fitness cost [1]. Thus, their rate in *Salmonella typhimurium* in specific *his* locus was estimated to be around 10^−4^ per cell [2] but the amplified regions were lost as easily as they gain, which could provide transient adaptivity to changing environmental conditions [3].

In *Salmonella*, the mechanism was most likely to be the RecA-dependent site-specific recombination since *recA*^-^ mutants had the duplication frequency 6000-folder reduced compared to the wild type [2]. However, the amplification events mediated by “copy-out-paste-in” transposition have been also described [4], although the RecA-dependent recombination remains to be the main amplification driver. RecA-dependent recombination requires the presence of two short repeated sequences flanking the amplified region, which are usually two identical copies of IS-elements (insertion sequence elements) or, slightly rarer, rRNA clusters [1].

The plague pathogen *Yersinia pestis*, which evolved from *Yersinia pseudotuberculosis* [5], had caused a few plague pandemics beginning from the Bronze Age [6–8]. Based on a comprehensive comparative genomic analysis of the members of the order ‘Enterobacteriales’, the bacterium *Y. pestis* was recently assigned to the newly identified family Yersiniaceae with the type genus *Yersinia* [9]. Two closely related pathogenic *Yersinia* – *Y. pestis* and *Y. pseudotuberculosis* are an excellent model for studying the molecular mechanisms of evolution of a highly virulent systemic pathogen – the causative agent of a deadly infection from an enteropathogenic saprophytic predecessor widely found in the environment [10]. The process of rapid evolution of *Y. pestis* included the acquisition of two plasmids of pathogenicity pMT1 and pPla, followed by the loss of functionality of many genes, which is generally characteristic of the evolution of bacteria that have entered the path of parasitic existence.

In addition, *Y. pestis* differs from other *Yersinia* species by an enormous number of IS-elements in its genome [11], which provoke a lot of large genome rearrangements in the chromosome [12]. However, events of tandem genome amplification have been clearly described only for *Yersinia enterocolitica* under stress conditions [13], and were barely mentioned for a couple *Yersinia pestis* strains [14,15], although it must be very prone to spontaneous duplications because of a lot of repeats in the genome.

On the other hand, the adaptive capabilities of *Y. pestis* are known to be associated with its genome plasticity [16], so the tandem genome amplification also might be an adaptive mechanism and should not be neglected. In this study, we aimed to consider large amplification events in the plague pathogen, estimate their frequency and potential drivers, and to consider the genes preferably located in the amplified regions.

## 2. Results

### 2.1. *Y. pestis* genomes could contain long tandem duplications

In this study, eleven *Y. pestis* strains were sequenced on two platforms and assembled. The list of considered *Y. pestis* strains included phylogenetic lines of highly pathogenic (0.ANT5, 2.MED1), and ancient (0.PE4h, 0.PE4t, and 0.PE4a) subspecies, where each except 2.MED1 subspecies was represented by two strains, and 2.MED1 was represented by three strains. The studied strains were collected from various loci in Russia, the Kyrgyz Republic, and the Republic of Tajikistan between 1971 and 2020 (S1 File), and the study was conducted in 2023–2024. To demonstrate phylogenetic diversity of the studied strains, 166 complete genomes of *Y. pestis*, for which phylogenetic groups were assigned [17] were additionally collected. The phylogenetic tree constructed based on co-aligned core genomes is available in S1 File.

**Fig 1:**
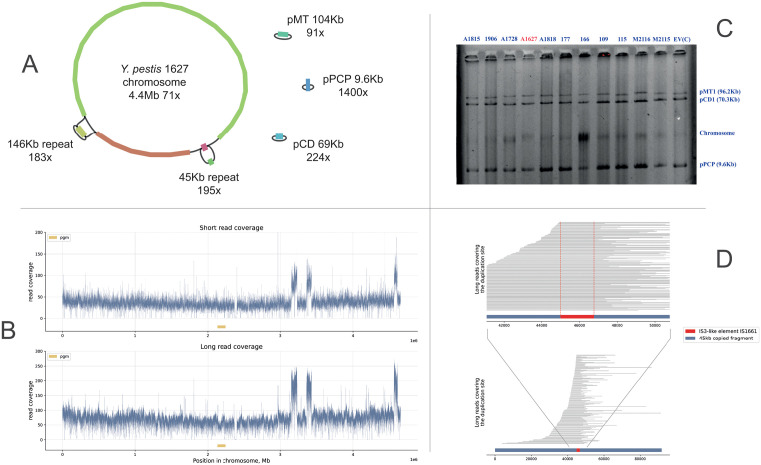
(A) The assembly graph obtained with Flye (v2.8.1) for *Y. pestis* 1627 strain. The chromosome includes two fragments (146Kb and 45Kb) with significantly increased coverage which is most likely explained by genome amplification events. (B) The read coverage of the *Y. pestis* 1627 chromosome (here, the upper plot was generated using short Illumina reads, the lower using long ONT reads). In both cases, reads were mapped on the reference *Y. pestis* CO92 genome. Here, the 146kb fragment appeared as two separated high-coverage peaks due to genomic rearrangements. (C) Electrophoresis results demonstrated absence of additional replicons. The strain of interest 1627 is colored by red. (D) The read coverage of the IS-element and two neighboring copies of the 45kb fragment from *Y. pestis* 1627 which proves tandem amplification. Here, the Copy1-ISe-Copy2 fasta sequence was artificially created and used as a reference. Only the long reads that covered both copies and the IS-element were selected for this illustration. Totally, 137 long reads approved the tandem topology of the 45kb amplified region.

Interestingly, in four sequenced strains, the chromosome in the assembly graph was not clearly circular as expected, and contained one or two fragments that were not resolved. These fragments had coverage depth significantly higher than the median chromosome coverage, so these chromosomal regions were represented in the genome in more than single copies. Thus, the *Y. pestis* strain 1627 had two such fragments with lengths 146Kb and 45Kb and more than 2-fold increased copy number (Fig 1A). The mapping of the raw reads on the reference *Y. pestis* CO92 genome clearly proved the same effect of increased copy number of a few chromosomal regions (Fig 1B). Based on the long Oxford Nanopore reads (Fig 1D), it was concluded that the copy number increase in the 45Kb region was caused by a tandem repeat of the long chromosomal regions flanked by two copies of the IS3-family element. To obtain the Fig 1D, an artificial sequence containing two copies of the 45Kb region connected via IS3 sequence was constructed and used as the mapping reference. Finally, for each studied *Y. pestis* strain, the electrophoresis investigations did not reveal the presence of any additional replicons (Fig 1C), which further confirmed the chromosomal localization and tandem repeats topology of the identified over-copied regions.

In total, five different regions with significantly increased or decreased coverage depth were found in 11 strains. The identified amplification drivers were IS21-like element IS100, IS200/IS605-like element IS1541B, IS3-like element IS1661, and IS256, which are the most represented IS-elements in the *Y. pestis* genome. In all cases, where the copy number was increased, the tandem repeat topology was confirmed by long reads, i.e., there were populations of reads supporting the connection of the repeat with the rest of the chromosome, and populations of reads supporting the connection of the repeat with itself in the “head-to-tail” manner (S1 File).

Interestingly, in *Y. pestis* strain 177 and 1815, the detected assembly graph anomalies had the length and the GC-content very close to the length and the GC-content of pMT1 and pCD1 plasmids respectively (S1 File). This phenomenon will be discussed further in more detail. It should also be noted, that in further sections, the 45K regions will be considered together with flanking IS3-elements, that is why it will be named 46Kb instead of 45Kb.

### 2.2. IS-elements are the main genome amplification drivers in *Y. pestis* but rRNA clusters can also be drivers

To extend the analysis, three of the most massive bioprojects containing *Y. pestis* WGS data were downloaded from the SRA database (accession numbers PRJNA421720, PRJNA891617, and PRJNA910854). We developed an algorithm that detects the regions with changed copy numbers (both decreased and increased) based on coverage depth profile. In total, 1125 read archives were processed. To avoid false-positive inference, all the results were manually checked and curated if necessary. 535 *Y. pestis* isolates had chromosomal regions with increased coverage, and 386 had regions with decreased copy number. All the coverage depth profiles and the identified regions are available in S1 Data-S3 Data, a table with all identified regions and their coordinates is available as S1 File. The identified amplified regions had the mean length of 100Kb. The longest identified regions had the length up to 1Mb. The mean copy number was about 2.5X, but in few biosamples the copy number was higher than 15X. The examples of a couple of extreme cases are available in S1 File.

Potential drivers of the amplification were inferred by considering the sequences flanking the detected regions. As expected, the most represented drivers were IS-elements of different families, but a number of amplificated regions were flanked by rRNA clusters (Table 1), which means that the amplification mechanism is most likely RecA-dependant site-specific recombination. Interestingly, MITE sequences (Miniature Inverted-repeat Transposable Elements) that are actually the most represented short multi-copy sequences in *Y. pestis* were not identified as amplification drivers, and only two identified cases might have been false positive inference. In total, the observed drivers were in accordance with main drivers of other genome rearrangement events such as inversion and transposition described in [18].

**Table 1:** The main drivers of tandem genome amplification in *Y. pestis.*

flanking sequence	regions with increased copy number	regions with decreased copy number	number in the genome
MITE sequence	2	0	56
IS21-like element IS100	431	383	44
IS200/IS605-like element IS1541B family transposase	82	3	36
IS200/IS605-like element IS1541A family transposase	159	23	28
IS256 family transposase	110	22	21
IS3-like element IS1661 family transposase	127	31	15
rRNA cluster	55	0	6
driver not identified	4	0	1

### 2.3. The reproducible amplified regions might mimicry the plasmids’ genome signatures

**Fig 2:**
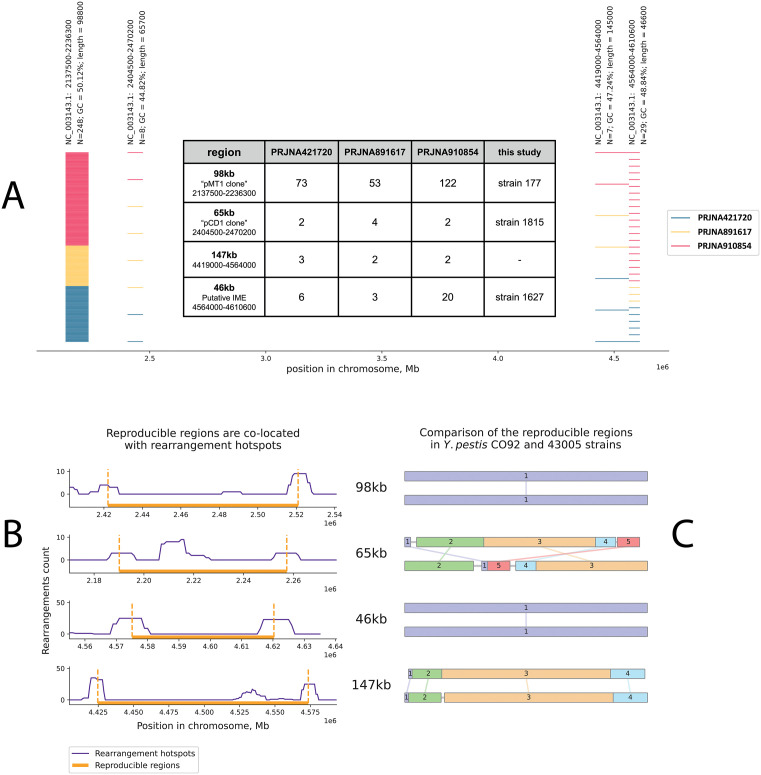
(A) The localization of the regions with unstable copy number reproduced in three analyzed bioprojects. The number of horizontal lines represent the number of biosamples where the reproducible regions were found, the color represents the bioprojects they belonged to. The most represented region (2137500-2236300) is the *pgm* region, which has the GC-content and the length very close to the pMT1 plasmid. The second region (2404500-2470200) has genome signatures close to the pCD1 plasmid. The region located in positions 4564000-4610600 has been identified as an integrative mobilizable element (IME). The longest reproducible region (4419000-4564000) shares the flanking IS3-element with the IME (that is why its length was identified as 145Kb instead of actual 147Kb). Additionally, the prevalence of the reproducible regions in strains from external bioprojects and this study is shown in the table (also available in S1 File). (B) Association of the reproducible regions with rearrangement hotspots described in [18]. In this plot, genome positions correspond to the *Y. pestis* 43005 strain since it was used as the reference in [18]. Only the 65kb region was not flanked by pronounced rearrangement hotspots because of structural changes in this region in the *Y. pestis* 43005 genome compared to CO92 (C).

The localization of the amplified regions seemed generally random but some amplification hot spots could be observed (S1 File). Reproducible regions were defined as the regions with changed copy number which were found in all three bioprojects in at least two different samples in each bioproject. Only four regions with chosen properties were identified. Their localization in the reference *Y. pestis* CO2 chromosome and their frequency is shown on Fig 2A.

The first reproducible region was the same 46Kb (45Kb without flanking IS3-element) chromosomal fragment that was found in the *Y. pestis* 1627 strain. This fragment had an increased copy number in 29 samples from all 3 bioprojects. In addition, based on raw reads we found that the same fragment is amplified in the *Y. pestis* PBM19 [19] (accession numbers SRR2175564 and SRR2175566), but in the corresponding complete genome ASM83423v1 available in RefSeq this tandem repeat is not resolved and presented only in one copy. This reproducible 46Kb fragment contains two *att-*sites, and genes encoding DNA relaxase, T4SS component VirB6, and T4 coupling protein (S1 File), and has been annotated by ICEfinder [20] as a putative integrative mobilizable element (IME), so it might exist and replicate in extra-chromosomal form.

The second and third reproducible regions should be considered together. Their lengths were estimated around 98Kb and 65Kb, respectively, and their GC-contents were 50.1% and 44.8%. The 98Kb fragment is actually the *pgm* region [21] that had a decreased copy number or total deletion in 248 samples. However, in PRJNA910854, at least 3 samples were detected, where *pgm* or its fragments had increased copy number (SRA accession numbers SRR23592771, SRR23592829, SRR23592764). Although amplification of *pgm* is significantly rarer than its elimination from the genome, we suppose that here both increase and decrease in copy number are provided by the same molecular mechanism, that is why it was decided not to distinguish *pgm* in our analysis from other true high-copy tandem duplications. The 65Kb region has not been described earlier as a mobilizable element, but had significantly increased copy number in 8 biosamples from all 3 bioprojects. Most interestingly, these 98Kb and 65Kb reproducible regions were the same as were found in the strains 177 and 1815 mentioned above, so both length and GC-content of these regions were almost identical to these characteristics of pMT1 and pCD1 plasmids respectively. It does not seem just a coincidence, because the fragments with such length and GC-content are strictly unique in the *Y. pestis* genome.

To estimate statistical significance of the observed GC-content/length similarity, we calculated the chances to obtain the same or better similarity by random selection of chromosomal regions. The calculated chance to randomly select two chromosomal regions so similar to pCD1 and pMT1 plasmids in four independent trials is about 9.3 × 10^−6^ (based on multinomial distribution formula). This indicates that reproduced instability of copy number in these two regions is highly likely associated with the observed similarity to the *Y. pestis* plasmids. Moreover, comparison of the 3-mer spectra of these fragments and corresponding plasmids revealed that the codon spectra of the 65Kb amplified region is closer to the pCD1 plasmid than any other random chromosome segment of the same length (p-value = 0.001 based on 1000 random selections) (S1 File). In the case of *pgm* and pMT1, the codon spectra of them were not so similar, but they both differ from other randomly selected chromosomal regions (S1 File). It should be noted that terms “mimicry” or “plasmid clones“ which will be used above are only contextual, and do not mean that these chromosomal fragments have significant nucleotide similarity or common genes with corresponding plasmids. Actually, the only genes shared between 98Kb/65Kb regions and pMT1/pCD1 plasmids are genes of IS-element transposases, while the rest gene content is totally different.

The fourth reproducible 147Kb region was the longest but was found only in 7 external biosamples and none of strains from this study (note that it is not related to 146Kb described in section 2.1). It did not have any ICE or IME factors, and its length or GC-content were not similar to any known *Y. pestis* plasmid. This fragment shares the flanking amplification driver (IS3-like element) with the 46Kb mobilizable element, and probably might use its mobilization factors.

To compare localization of the four reproducible regions with rearrangement hotspots described by Wu et al. [18], genome positions of these regions were redefined using *Y. pestis* 43005 genome as a reference. Localization of regions 98Kb, 46kb, and 147kb was associated with rearrangement hotspots (Fig 2B). Opposite to them, the 65kb region was not clearly flanked by rearrangement hotspots, most likely due to structural changes in this region in the 43005 strain compared to CO92 (Fig 2C).

### 2.4. Functional enrichment of the regions with unstable and stable copy number

Functional enrichment of all identified regions with unstable copy numbers (including non-reproducible regions) was performed based on KEGG Orthology groups [22]. The most over-represented pathways, found in these regions, were biosynthesis of polyketide and siderophore nonribosomal peptide. Actually, both these metabolic pathways represent biosynthesis of yersiniabactin, which is encoded by a gene cluster located in the *pgm* region [23], known for its high plasticity [24]. Besides yersiniabactin biosynthesis, significant over-representation in the considered regions (Chi-test FDR corrected p-value < 0.001) was shown for few other pathways, including biofilm formation, exopolysaccharide biosynthesis, and metabolism of some amino acids (Table 2). In the same manner, the genes which tended not to be located in the regions with unstable copy number were inferred (Table 3).

**Table 2:** KEGG pathways over-represented in the regions with unstable copy number.

KEGG Pathway	p-value
02026 Biofilm formation – Escherichia coli [PATH:ko02026]	0.00053997
02000 Transporters [BR:ko02000]	0.0003061
02020 Two-component system [PATH:ko02020]	0.00047286
99995 Signaling proteins	0.00010885
00543 Exopolysaccharide biosynthesis [PATH:ko00543]	0.00038138
00330 Arginine and proline metabolism [PATH:ko00330]	1e-08
01053 Biosynthesis of siderophore group nonribosomal peptides [PATH:ko01053]	0.0
01008 Polyketide biosynthesis proteins [BR:ko01008]	0.0

**Table 3:** KEGG pathways under-represented in the regions with unstable copy number.

KEGG Pathway	p-value
00230 Purine metabolism [PATH:ko00230]	8.986e-05
03036 Chromosome and associated proteins [BR:ko03036]	4.587e-05
02040 Flagellar assembly [PATH:ko02040]	0.00021512
00540 Lipopolysaccharide biosynthesis [PATH:ko00540]	0.00029531
03010 Ribosome [PATH:ko03010]	0.00077556
00190 Oxidative phosphorylation [PATH:ko00190]	6.525e-05

### 2.5. The association between presence of over-copied regions and available metadata

**Fig 3:**
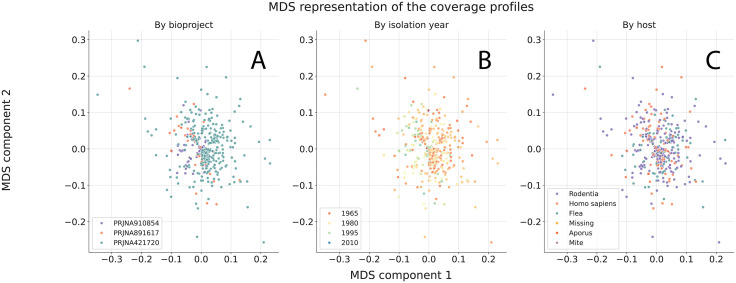
The MDS representation of the coverage depth profiles obtained for external data. Points are colored (**A)** according to the bioproject to which studied biosamples belonged to (**B)** according to the isolation year specified for the studied biosamples (**C)** according to the host from which the biosamples were isolated.

Based on the metadata available for the biosamples analyzed, the association between metadata and genome coverage profiles was checked. The only feature that demonstrated visible association with the presence and localization of over-copied regions was the BioProject accession number (Fig 3A), while other metadata that included host and isolation year did not show any visible clusterization (Fig 3B–C). MDS plots built for each bioproject independently are available in S1 File. Neither the host specified, nor the year of isolation, nor the __cpLocation of the isolates showed an association with the coverage profiles.

## 3. Discussion

The genome plasticity of the plague pathogen *Yersinia pestis* is a known phenomena. The main drivers of large recombination events in Y. pestis are multi-copied IS-elements of different families, which are presented in the *Y. pestis* genome in drastically more copies than in closely related bacteria including *Y. enterocolitica*, *Y. pseudotuberculosis* or even in any representatives of *Enterobacterales* [25].

In this study, it was shown that in addition to large genome rearrangements, some regions of the *Y. pestis* chromosome may vary by their copy number. In such regions, in most cases, the copy number is significantly increased compared to the chromosome. However, the most variable region – the *pgm* region – demonstrated stable decrease in copy number, which indirectly indicates the population heterogeneity. According to literature, the loss of *pgm* is a quite frequent event in *Y. pestis* laboratory isolates [24], so this result was expected.

Mapping of long ONT reads revealed that the increased copy number is explained by tandem repetition of long chromosomal regions which is more known as tandem genome amplification. Unfortunately, for the external samples, the tandem topology could not be checked based only on short reads but we suppose that they have the same nature. The amplified regions could have the length up to 1Mb and the copy number higher than 15X in some strains. It was shown that most regions with a changed copy number were flanked by two identical copies of IS-elements highly presented in the *Y. pestis* genome such as IS100, IS1541A and IS1541B, IS256, and IS1661. However, 55 processed isolates harbor the amplified regions flanked by rRNA*-*clusters, which means that the presence of IS-element transposases is not obligate (but may be preferred) for the tandem amplification in *Y. pestis*, and the recombination mechanism is most likely RecA-dependent site-specific recombination. Indeed, it has been shown that the frequency of genome duplications caused by recombination of long repeated sequences is in strong dependence on RecA activity [26]. Additional evidence of RecA-dependance is that RecA plays a predominant role in instability of the *pgm* locus [27]. Few biosamples were found where the number of copies of *pgm* or its fragments was increased compared to the rest of the chromosome, and we suppose that both increase and decrease in *pgm* copy number are provided by the same molecular mechanism.

However, there are other RecA-independent amplification mechanisms supposed, such as the single-strand annealing pathway. Thus, in *Salmonella enterica*, frequency of duplication of *argH* gene located between two *rrn* loci was identical in RecA- mutants and the wild type [28]. Another option is the “copy-out-paste-in” transposition pathway described for a number of IS-element families, mainly for IS3 [29]. Actually, this process does not result in tandem duplication, but can increase a copy number of the chromosomal genes involved in the transposition. Interestingly, MITE sequences that are actually the most represented short multi-copy sequences in *Y. pestis* [30] were not identified as amplification drivers. Probably, the length of MITEs which is about 70 bp is too short to provide successful non-equal exchange between sister chromatids.

Although mainly the regions with changed copy number demonstrated random localization, a few hotspots reproduced between bioprojects were found. As expected, one of them was the *pgm* region mentioned above. Another clear hotspot was identified as an integrative mobilizable element and was found in a lot of external biosamples including one RefSeq *Y. pestis* complete genome PBM19 and one our strain. In external data, we have not found evidence that this region could exist in extrachromosomal form since short reads do not allow to clearly distinguish the tandem repeats from the coiled form, but this region includes few known mobilization factors.

In total, four reproducible regions with unstable copy number were identified, and the most intriguing finding was a strong similarity of two identified regions with *Y. pestis* plasmids. Thus, the *pgm* region has the length of approximately 98Kb, and the GC-content of 50.1% that is almost identical to the corresponding genomic signatures of the pMT1 plasmid. At the same time, the reproducible region located in positions 2402800–2471400 demonstrated similarity of the same characteristics with the pCD1 plasmid. Taking into account that, first, in both cases the regions with such length and GC-content were unique in the reference *Y. pestis* genome, and, second, the GC-content of all *Y. pestis* replicons is very stable between different strains, the observed similarity of genome signatures can hardly be just a coincidence. Despite sharing GC-content and length, these chromosomal regions do not share any gene content features with plasmids except IS-element transposase genes. In the case of the 65Kb region, significant similarity in codon spectra with the pCD1 plasmid was also observed. Both pMT1 and pCD1 “clones” with a changed copy number were found in strains 177 and 1815 sequenced during this study. Unfortunately, the mechanism of such molecular mimicry remains a matter of speculation.

The adaptive role of such high variance in copy number in *Y. pestis* compared to other bacteria also remains unclear. A few metabolic pathways significantly over-represented and under-represented in the identified regions were found, but we could not link them to the phenotypic properties of strains since there were very few metadata for the selected biosamples in SRA. A number of housekeeping pathways, such as chromosome maintenance, ribosome biosynthesis, and oxidative phosphorylation, were found among the pathways significantly under-represented in the regions with unstable copy number. Perhaps, changing the expression level of these genes might have negative consequences for the bacterial cell, so their copy number should remain more stable.

No clear association was found between presence of specific amplified regions and metadata available for the biosamples studied, and the only feature that showed visible association was the bioproject accession number, which is mainly explained by the fact that the samples from PRJNA421720 had higher variability of the identified regions, while in biosamples from PRJNA891617 and PRJNA910854 a very limited number of different amplified chromosome fragments was observed, so it can be supposed that different conditions of cultivation and storage of the *Y. pestis* isolates could somehow affect the appearance of described recombinations. In general, the author suppose that under laboratory conditions the *Y. pestis* populations are more prone to spontaneous tandem genome amplification than in a natural environment but that requires further confirmation.

## 4. Methods and materials

### 4.1. Bacteria procedures

The *Y. pestis* strains used in this study were obtained from the Russian Research Anti-Plague Institute “Microbe” (Saratov, Russia) and presented in S1 File. Strains were grown from single colonies on Luria-Bertani agar (LB) (pH 7.2 ± 0.1) for 48 h at 28°C for biomass build-up [31,32]. Traditional methods of laboratory diagnostics were used to assess the phenotypic properties of the strains [33]. All strains had the following phenotypes: F1+ (able to produce fraction 1 antigen or the capsule), Pst+ (able to produce pesticin) and Pgm+ (pigmentation on Congo red media).

### 4.2. DNA extraction

For DNA extraction, 5 ml LB broth (pH 7.2) was seeded with overnight bacterial culture 10^8^ CFU Y. pestis and was cultured at 28 °C in a shaker incubator at 100–110 RPMs to the exponential growth phase (OD565 = 2 McFarland). A McFarland Densitometr DEN-1B (BioSan) was used for OD determination. The DNA of the *Y.pestis* strains was obtained using phenol−chloroform extraction [34].

### 4.3. Screening of the plasmid profile of Y. pestis strains

The plasmid profile of *Y. pestis* strains was determined according to the method of C.I. Kado and Liu S.T [35]. For plasmid screening, Y. pestis strains were cultured on LB agar at 28 ºC for 24 hours. The three-plasmid reference strain Y. pestis EV line NIIEG with known molecular mass of plasmids (pMT1, pCD1, pPCP1) was used as a molecular marker. Electrophoresis was performed in 1хTAE buffer (pH 7.8–7.9) using 0.7% agarose gel at 12 W/cm^2^ for 1–2 h. The presence of plasmids in the genome was confirmed by detecting plasmid DNA bands: ~ 96, 2 bp (pMT1), ~ 70.3 bp (pCD1), and ~9.6 bp (pPCP1). Electrophoretic separation was analyzed using a Bio Rad Gel Doc XR+ transilluminator (USA).

### 4.4. Library Preparation and high-throughput sequencing

The concentration of total DNA was measured using Qubit 4.0 Fluorometer and Qubit dsDNA BR Quantification Assay Kit (Thermo Fisher Scientific, USA). Library preparation was performed using the NEBNext ULtra™ II End Repair/dA-Tailing Module (NEB, USA) according to the manufacturer’s protocol. Barcode ligation was performed using Blunt/TA Ligase Master Mix (NEB, USA) and SQK-NBD104.24 (ONT, UK). High-throughput sequencing was performed using PromethION with FLO-PRO002 Flow Cell (R9.4.1) and Flow Cell Priming Kit EXP-FLP002 (Oxford Nanopore Technologies, UK).

Short read sequencing was performed on Illumina MiSeq sequencing platform (PE300) using the core facilities of the Lopukhin FRCC PCM “Genomics, proteomics, metabolomics” (http://rcpcm.org/?p=2806).

### 4.5. External data acquisition

The bioprojects in the SRA database were selected so that for each biosample the host, isolation year and __cpLocation were specified, and there were at least 100 different biosamples per bioproject. To provide satisfactory mapping specificity, only read archives with read length not less than 100 were considered. In total, 1125 read archives containing *Y. pestis* whole-genome sequencing data were downloaded from SRA (bioprojects accession numbers PRJNA421720, PRJNA891617, PRJNA910854).

NCBI metadata file containing information for the studied biosamples about isolation year, __cpLocation, and host was used to analyze the association between coverage depth profiles and these selected features.

### 4.6. Detection of regions with abnormal copy number

To detect regions with a changed copy number, a specialized algorithm was developed. First, raw reads are mapped to the reference genome (in this study *Y. pestis* CO92 was used). The resulting BAM-file is processed using the “samtools depth -a” command, which returns a coverage depth profile. This profile is used to detect target regions.

The algorithm works in the sliding window manner, iteratively selecting chromosomal segments of a fixed size (5000 bp), and compares the median coverage depth in the segment (*segment_median*) with the median coverage depth of the whole chromosome (*global_median*). If median coverage of the segment is greater than global_median*1.25 or less than global_median*0.75, the algorithm considers the segment positions as positions with a changed copy number. When all chromosomal segments are processed, the algorithm has noisy information about localization of regions with a changed copy number. The next step is correction of the resulting regions by filling short gaps between target regions and removing too short target regions. The algorithm returns a BED-file with assigned regions and fold-change values. A more detailed description of the algorithm is available in S1 File. The algorithm has few hyperparameters that were optimized based on a limited number of coverage profiles for which the localization of the target regions was assigned manually. The scheme of the parameters optimization is described in S1 File.

Although the developed approach demonstrated satisfactory accuracy, we manually curated all the results to avoid false-positive inference.

### 4.7. Amplification drivers identification

Firstly, based on the k-mer approach, we found all the repeated sequences in *Y. pestis* CO92 regions. This list included IS-elements of different families, rRNA clusters, and MITE-sequences. All these sequences were considered as potential amplification drivers. For each detected amplified region, we checked if it was flanked by one or two of these potential drivers. The distance of 2Kb between a region flank and the driver was chosen as a threshold. Different rRNA cluster genes (5S, 16S, 23S) were considered as one driver. Two ORFs from IS21-like element IS100 (*IstA* and *IstB*) also were considered as one driver.

### 4.8. Software and statistical methods used

The raw reads were firstly trimmed using Trimmomatic and Porechop for short and long reads respectively. Unicycler (v0.4.8) and Flye (v2.8.1-b1676) were used for genome assembly. BWA and minimap2 were used for read mapping. Samtools was used for mapping results manipulating. Roary 3.13.0 was used to generate core genome alignment. Fasttree 2.1.11 was used to build a phylogenetic tree.

KofamKoala was used for functional enrichment. For each gene, assigned KEGG records were transformed to high-level KO pathways from the third level of the KEGG hierarchy. For target and control genome regions independently, the vectors of KO frequencies were constructed. Next, each vector feature, representing a frequency of a single third-level KEGG pathway, was compared between target and control regions using Chi-contingency test (Scipy.stats.chi_contingency) with FDR correction (statsmodels.stats.multites.multipletests). KO pathways with p-value < 0.001 were considered as significant.

For MDS analysis of coverage depth profiles, the positions of the regions with changed copy number were represented as binary vectors of length equal to the reference genome length, where the ones indicated positions with changed copy number. Resultant binary vectors, after manual curation, were used to calculate the pairwise Jaccard distance matrix. This matrix was utilized as a pre-computed dissimilarity matrix in sklearn.manifold.MDS to reduce dimensions to 2 components.

## 5. Conclusion

The *Y. pestis* chromosome is very prone to the appearance of long tandem repeats. The main amplification drivers are IS-elements of different families but rRNA clusters have been identified as recombination drivers as well so the amplification mechanism is most likely site-specific RecA-dependent recombination. There were four regions with a changed copy number which were reproducible between all studied bioprojects. One of these regions was identified as a putative integrative mobilizable element but we have not managed to detect it in the extrachromosomal form based on our data or external read archives. Two regions reproduced between bioprojects demonstrated strong similarity of their length and GC-content with pMT1 and pCD1 plasmids.

## Supporting information

S1 File Includes 12 subsections with the main supplementary figures and tables. (DOCX)

S1 Data.
Is a multipage pdf-fileÂ with coverage depth profiles and identified regions for bioprojectÂ PRJNA421720. (PDF)

S2 Data.
Is aÂ multipage pdf-file with coverage depth profiles and identified regions for bioprojectÂ PRJNA891627. (PDF)

S3 Data Is a multipage pdf-file with coverage depth profiles and identified regions for bioprojectÂ PRJNA910854. (PDF)

S4 Data Contains coordinates of all identified regions and fold-change values in a table format. (XLSX)

S1 raw images Raw images is the original photo of the electrophoretic gel used in Fig 1. (PDF)
