# Supplementary material for: Systematic analysis of spontaneous tandem genome amplification events in Yersinia pestis
Source: PLoS One. 2025 Dec 31;20(12):e0338460. doi: 10.1371/journal.pone.0338460 (PMC12755819; doi:10.1371/journal.pone.0338460)
Supplement: S1 File — (DOCX) [file pone.0338460.s001.docx]

**Supplementary Materials for “Systematic analysis of tandem genome amplification events in *Yersinia pestis*”**

1. **The collection of the strains investigated in the study.**

**Supplementary Table 1. The list of *Y. pestis* strains used in the study.**

| **№** | **Strain** | **Isolation source, date** | **Isolation place^^[[1]](#footnote-1)^^** | **Subspecies, biovar, phylogenetic line branch^^[[2]](#footnote-2)^^** |
| --- | --- | --- | --- | --- |
| 1 | 177 | *Marmota baibacina*, 08.09.2019 | Upper-Naryn high-mountain plague focus, Kyrgyz Republic | *Y. pestis* subsp. *pestis* (bv. antiqua*)*  0.ANT5 |
| 2 | 166 | *Marmota baibacina*, 25.11.2020 | Sarydzhas high mountain plague focus, Kyrgyz Republic | *Y. pestis* subsp. *pestis* (bv. antiqua*)*  0.ANT5 |
| 3 | 1906 | *Meriones meridianus*  12.05.2014 | Caspian sandy plague focus, Republic of Kalmykia, Russia | *Y. pestis* subsp. *pestis* (bv. medievalis*)* 2.MED1 |
| 4 | 2115 | *Meriones meridianus*  31.10.2014 | Caspian sandy plague focus, Republic of Kalmykia, Russia | *Y. pestis* subsp. *pestis* (bv. medievalis)  2.MED1 |
| 5 | 2116 | *Xenopsylla conformis*  03.11.2014 | Caspian sandy plague focus, Republic of Kalmykia, Russia | *Y. pestis* subsp. *pestis* (bv. medievalis)  2.MED1 |
| 6 | 1815 | *Ceratophyllus caspius*  06.09.1980 | Talas high-mountain plague focus, Kyrgyz Republic | *Y. pestis* subsp. *central asiatica* (bv. talassica)  0.PE4t |
| 7 | 1818 | *Alticola argentatus*  28.08.1980 | Talas high-mountain plague focus, Kyrgyz Republic | *Y. pestis* subsp. *central asiatica* (bv. talassica)  0.PE4t |
| 8 | 1728 | *Alticola argentatus*  09.07.1972 | Hissar high-mountain plague focus, Republic of Tajikistan | *Y. pestis* subsp. *central asiatica* (bv. hissarica)  0.PE4h |
| 9 | 1627 | *Neodon juldaschi*  08.06.1971 | Hissar high-mountain plague focus, Republic of Tajikistan | *Y. pestis* subsp. *central asiatica* (bv. hissarica)  0.PE4h |
| 10 | 109 | *Сtenophyllus hirticrus*  29.04.2019 | Gorno-Altai high mountain plague focus, Altai Republic, Russia | *Y. pestis* subsp. *central asiatica* (bv. altaica)  0.PE4a |
| 11 | 115 | *Paramonopsyllus scalonae*  29.04.2019 | Gorno-Altai high mountain plague focus, Altai Republic, Russia | *Y. pestis* subsp. *central asiatica* (bv. altaica)  0.PE4a |

1. **Phylogenetic diversity of the studied strains.**

**

**

**Supplementary Figure 1.** Phylogenetic tree of 166 complete genomes from GenBank and 11 strains used in this study (orange). Each node label consists of the assigned phylogenetic line and the strain name.

1. **Confirming tandem repeat topology of the regions based on long reads.**

To confirm the tandem repeat topology of the identified regions with increased copy number, mapping of long ONT reads to these regions was considered. **Supplementary Figure X** shows mapping of long reads to the 146Kb amplified region in *Y. pestis* 1627 as an example of the analysis. The same procedure was conducted on five amplified regions detected in strains sequenced during this study, and the number of reads supporting head-to-tail connection, and connection with the rest chromosome was calculated. The results are presented in **Supplementary Table X**.

**
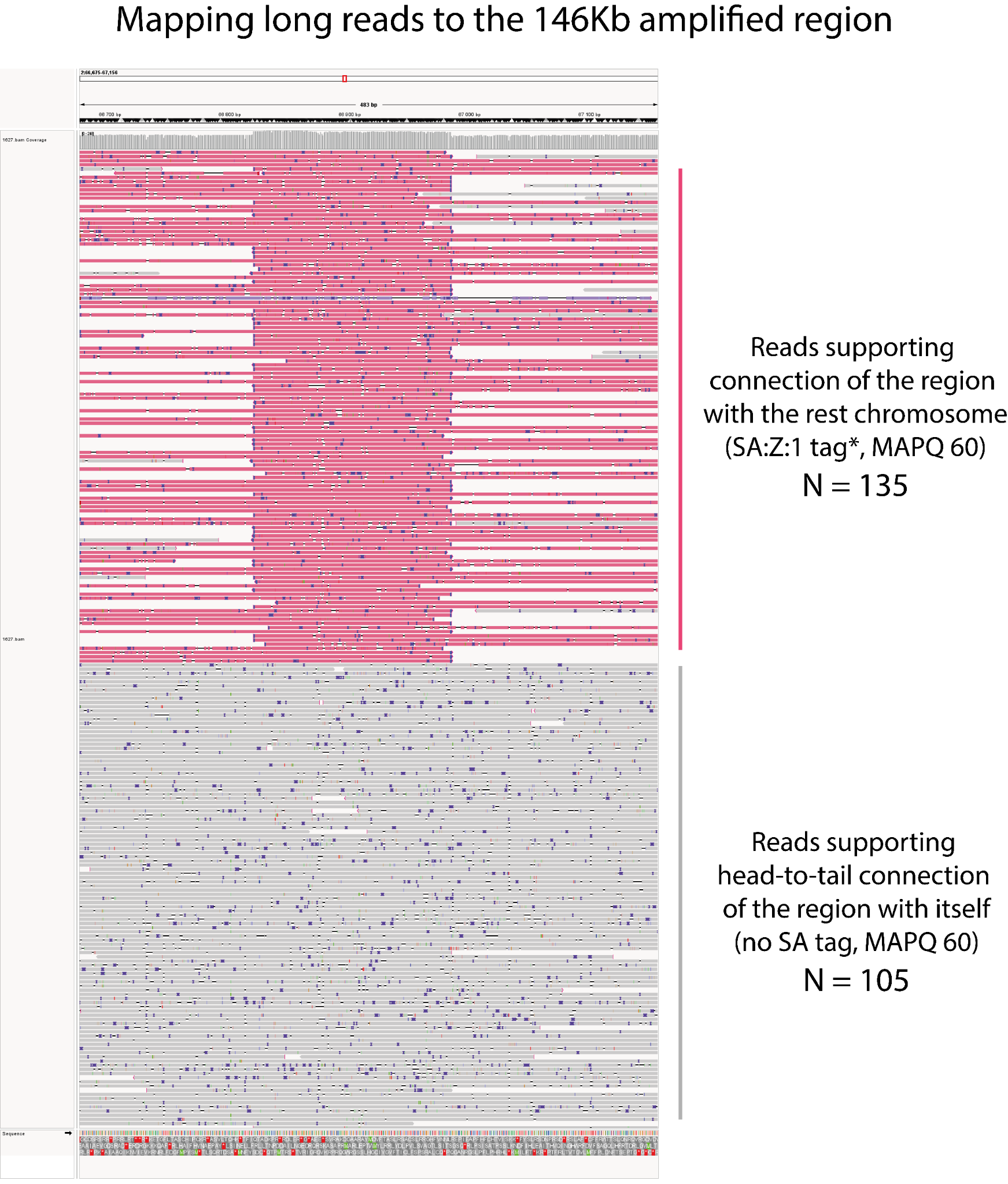
**

**Supplementary Figure 2.** Long reads mapped to the contig “2” representing 146Kb amplified region in *Y. pestis* 1627 strain. The reads are sorted and colored based on SA:Z:1 tag, that means the presence of supplementary alignment of the read to another contig “1” representing the rest chromosome. Only reads with MAPQ 60 were considered.

**Supplementary Table 2. The number of long reads supporting tandem repeat topology of the amplified regions.**

| **Region** | **N reads supporting head-to-tail connection** | **N reads supporting connection with the rest chromosome** |
| --- | --- | --- |
| 46Kb, *Y. pestis* 1627 | 137 | 103 |
| 146Kb, *Y. pestis* 1627 | 105 | 135 |
| 151Kb, *Y. pestis* 1728 | 53 | 104 |
| 65Kb, *Y. pestis* 1815 | 228 | 78 |
| 25Kb, *Y. pestis* 1818 | 294 | 336 |

1. **Chromosomal regions with changed copy number in 177 and 1815 strains.**

**
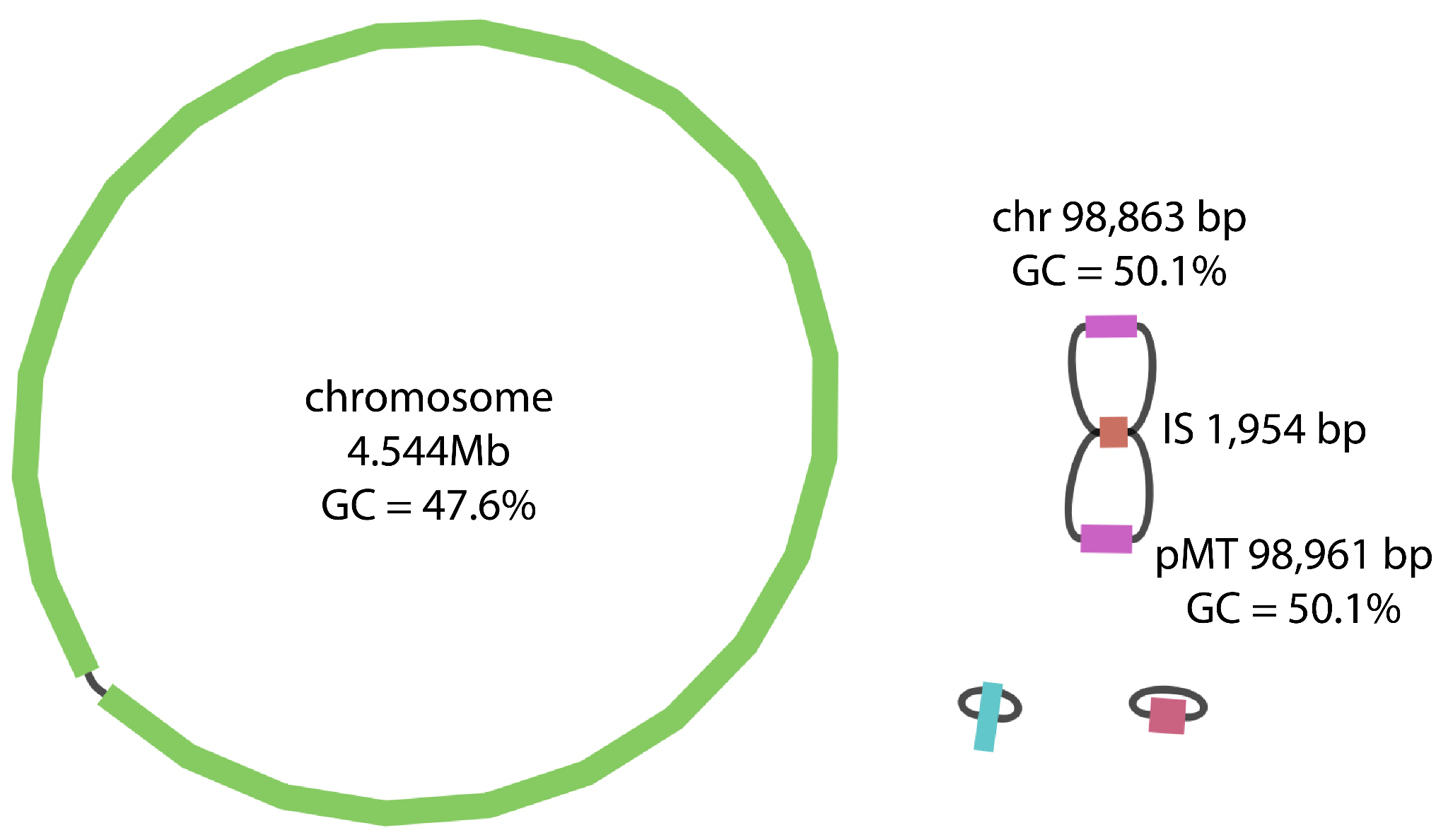
**

**Supplementary Figure 3.** The assembly graph for *Y. pestis* 177 strain. The chromosomal region with decreased copy number (“chr 98,863bp”, coverage depth ~0.5X compared to the main chromosome) had the length and GC-content very close to the pMT1-plasmid.

**
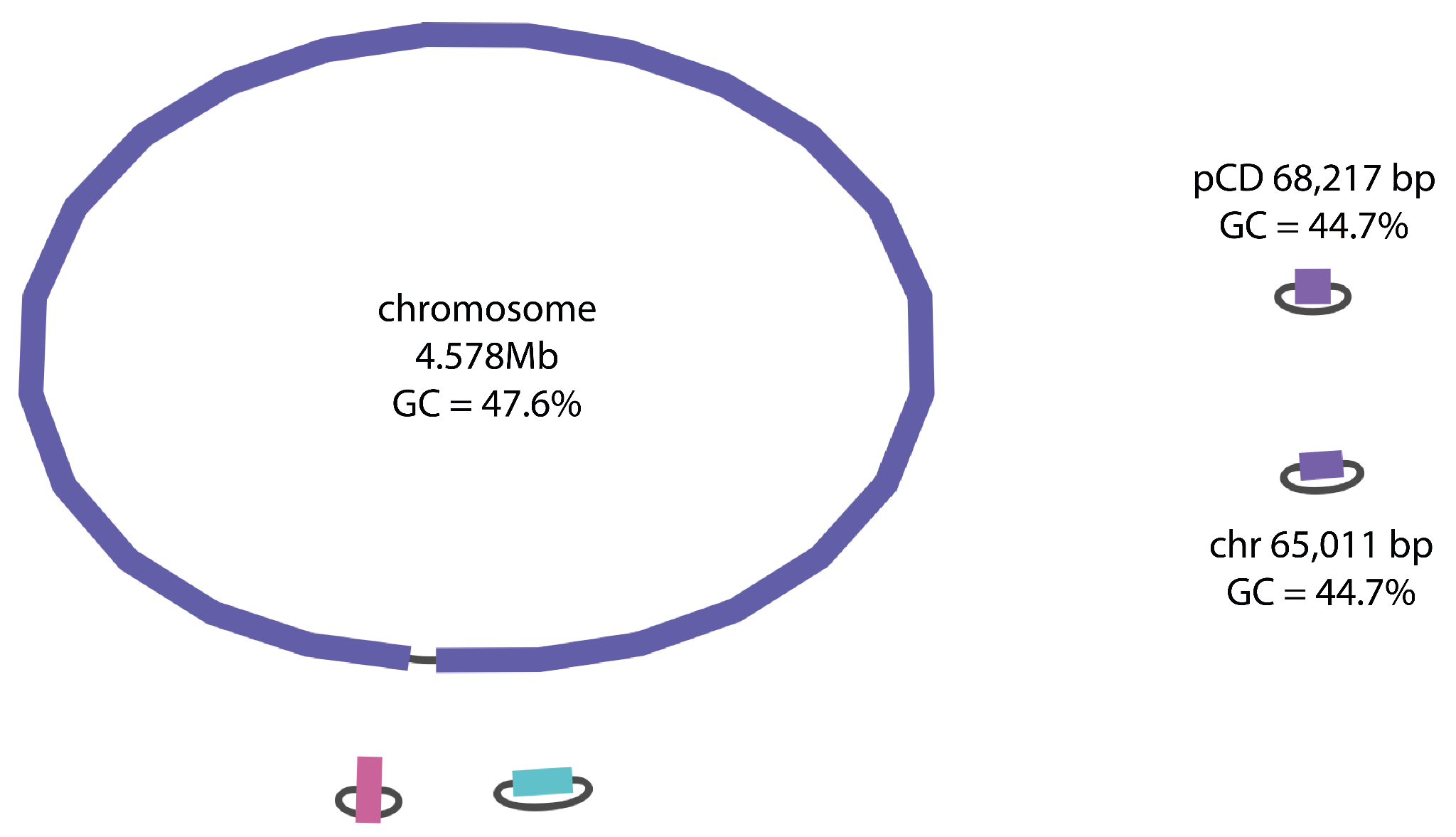
**

**Supplementary Figure 4.** The assembly graph for *Y. pestis* 1815 strain. The chromosomal region with increased copy number (“chr 65,011bp”, coverage depth ~1.5X compared to the main chromosome) had the length and GC-content very close to the pCD1-plasmid.

It should be noted that both chromosomal regions from Supplementary Figures 1 and 2, although assembled as coiled structures independent from the main chromosome, actually were located in the chromosome.

1. **The examples of amplified regions with extremal length or coverage depth values.**

**
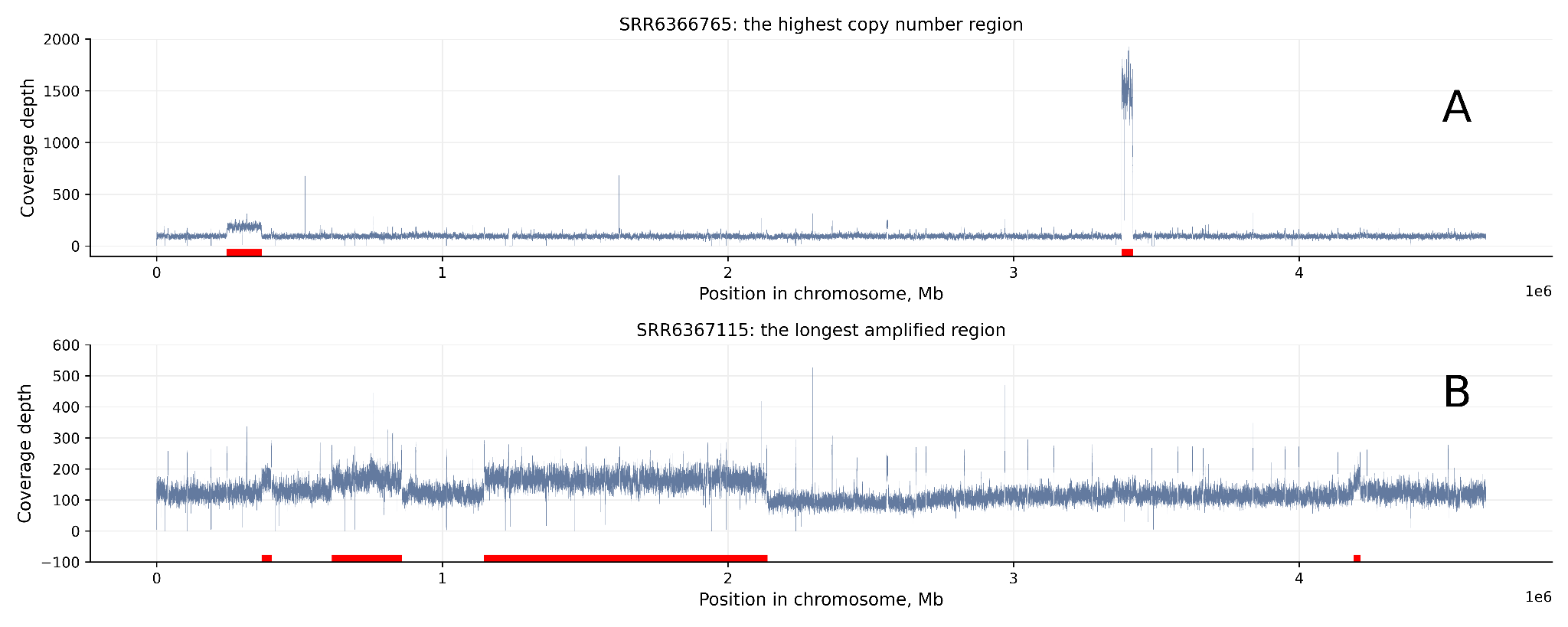
**

**Supplementary Figure 5.** Two extreme cases of amplified regions. **A)** The chromosomal region with a copy number higher than 15X. **B)** The longest chromosomal region with significantly increased copy number.

1. **The localization of the recombination hotspots.**

**
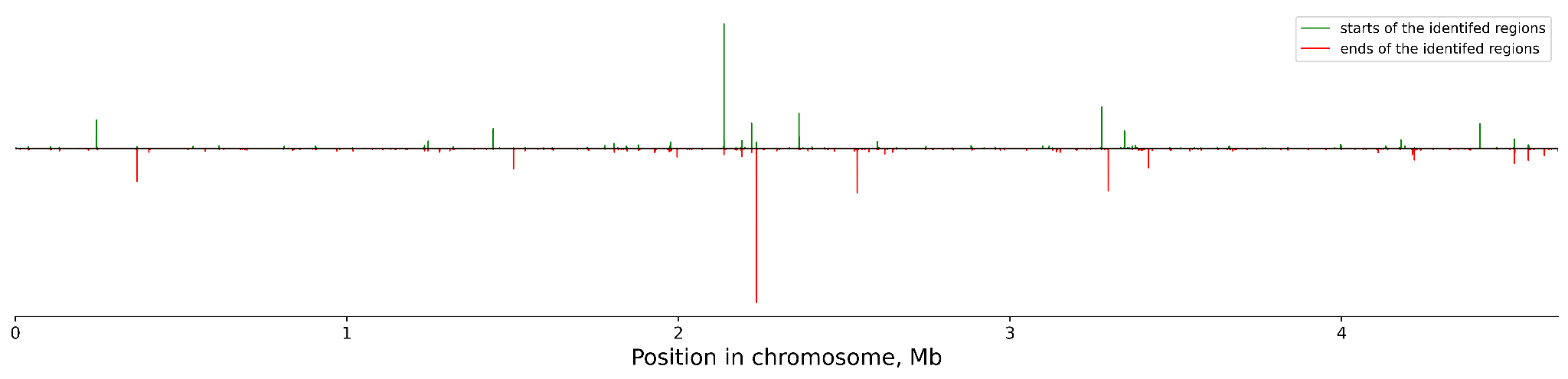
**

**Supplementary Figure 6.** The localization of the regions with changed copy number in the *Y. pestis* chromosome (*Y. pestis* CO92 genome was used as the reference). The green branches represent start positions of the identified regions, the red branches represent their end positions. All identified regions were used to plot these profiles. Indeed, the amplification events are not equally distributed along the chromosome and concentrate in certain amplification hotspots.

1. **The structure of the integrative mobilizable element identified by ICEfinder.**


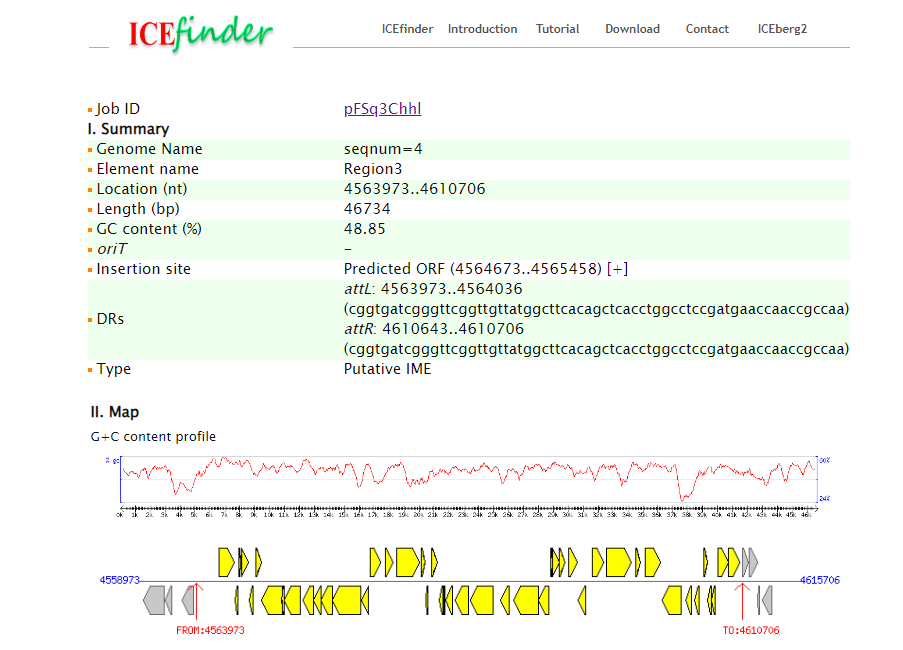


**Supplementary Figure 7.** The identified putative IME found in the *Y. pestis* CO92 genome. The IME coordinates are almost identical to the coordinates of the chromosomal region which had significantly increased copy number in 29 external biosamples from three bioprojects, and in our *Y. pestis* strain 1627.

1. **The comparison of codon spectra of the identified regions similar to the *Y. pestis* plasmids and corresponding plasmids pMT1 and pCD1.**

Two of four identified regions with reproducibly changed copy number demonstrated a strong similarity in their length and GC-content with pMT1 and pCD1 plasmids, so we decided to additionally check if there was similarity in their codon content.

We extracted the target regions, the corresponding plasmid sequences, and added to them a number of randomly selected not overlapping chromosome regions of the same length (~100Kb for pMT1, ~70Kb for pCD1) as a control. The codon content was calculated as the relative frequencies of nucleotide 3-mers. For MDS, CLR-transformed vectors with Euclidean distance were used.

**8.1. pCD1 plasmid**

The pCD1 plasmid and its chromosomal “mate” demonstrated a strong similarity of their codon spectra (p-value = 0.001 based on 1000 random selections), which can be observed on both MDS and heatmap plots (**Supplementary Figure 6**). The random region 10, located close to the pCD1 on the heatmap, had occasional overlap with the pCD1 “clone” region due to random selection of the control regions.

**
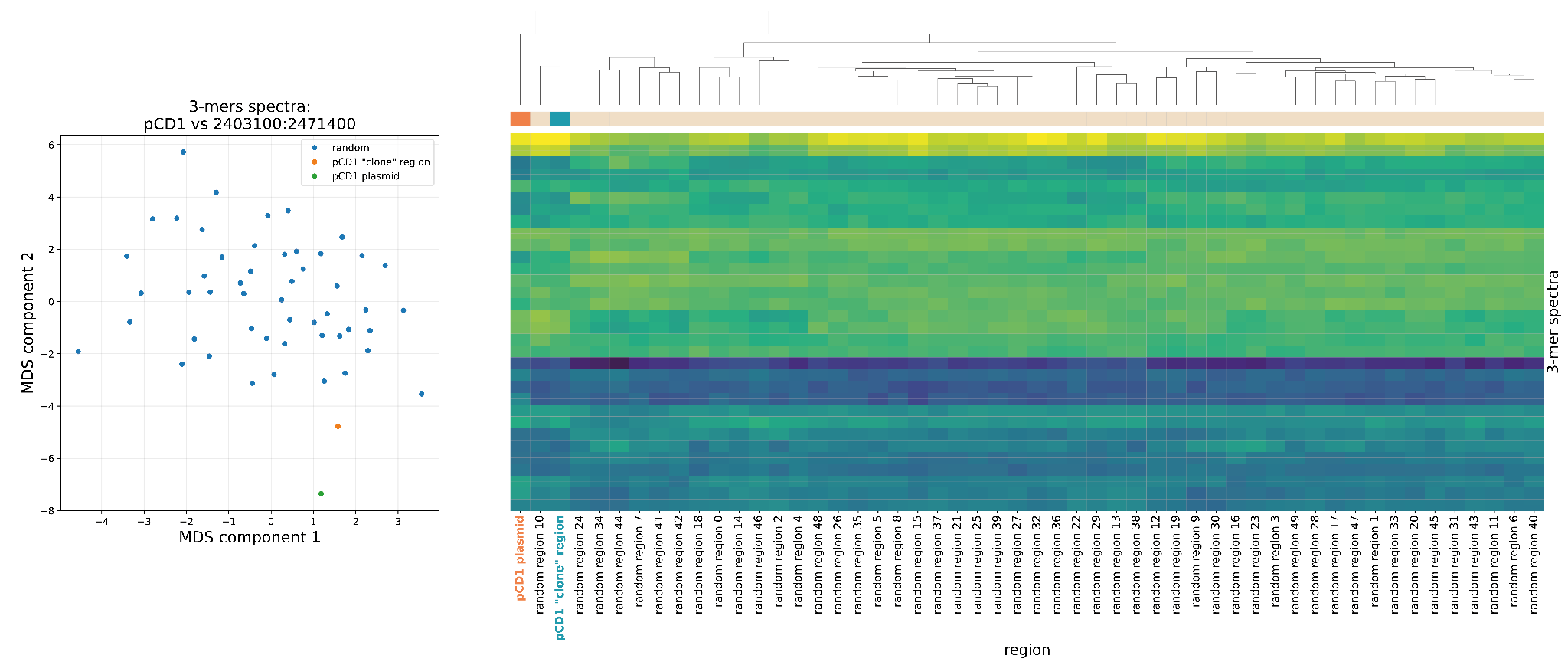
**

**Supplementary Figure 8.** The k-mer spectra of the pCD1 plasmid, its chromosomal “clone” (NC_003143.1 : 2403100-2471400), and a number of random *Y. pestis* chromosomal fragments of the same length.

**8.2. pMT1 plasmid**

Another situation was observed for pMT1 and its chromosomal mate. Their codon did not show visible similarity, but both these fragments had codon spectra different from the rest of the chromosome (**Supplementary Figure 7**).

**
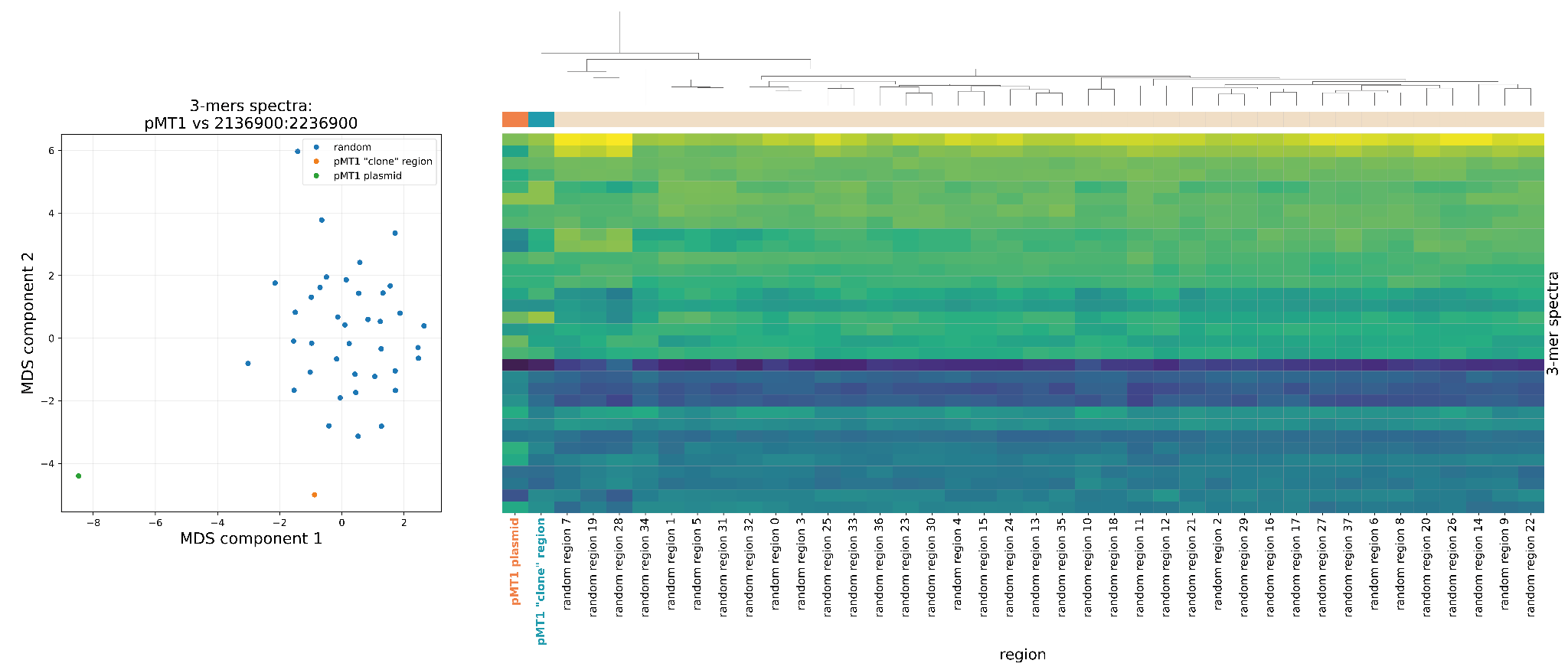
**

**Supplementary Figure 9.** The k-mer spectra of the pCD1 plasmid, its chromosomal “clone” (NC_003143.1 : 2136900-2236900), and a number of random *Y. pestis* chromosomal fragments of the same length.

1. **Regions with unstable copy number reproduced in different bioprojects.**

**Supplementary Table 3.** Regions with unstable copy number reproduced in different bioprojects.

| **region** | **PRJNA421720** | **PRJNA891617** | **PRJNA910854** | **this study** |
| --- | --- | --- | --- | --- |
| **98kb**  “pMT1 clone”  2137500-2236300 | 73 | 53 | 122 | strain 177 |
| **65kb**  “pCD1 clone”  2404500-2470200 | 2 | 4 | 2 | strain 1815 |
| **147kb**  4419000-4564000 | 3 | 2 | 2 | - |
| **46kb**  Putative IME  4564000-4610600 | 6 | 3 | 20 | strain 1627 |

1. **The association between available metadata and coverage profiles.**

To avoid batch effects caused by probably different conditions of cultivation and cultures storage, we performed MDS analysis for each bioproject independently. We did not find any significant associations with isolation year, location and host. For bioprojects PRJNA891617 and PRJNA910854, the observed number of points is less than the number of samples since a lot of profiles in these bioprojects did not contain any coverage anomalies.

**
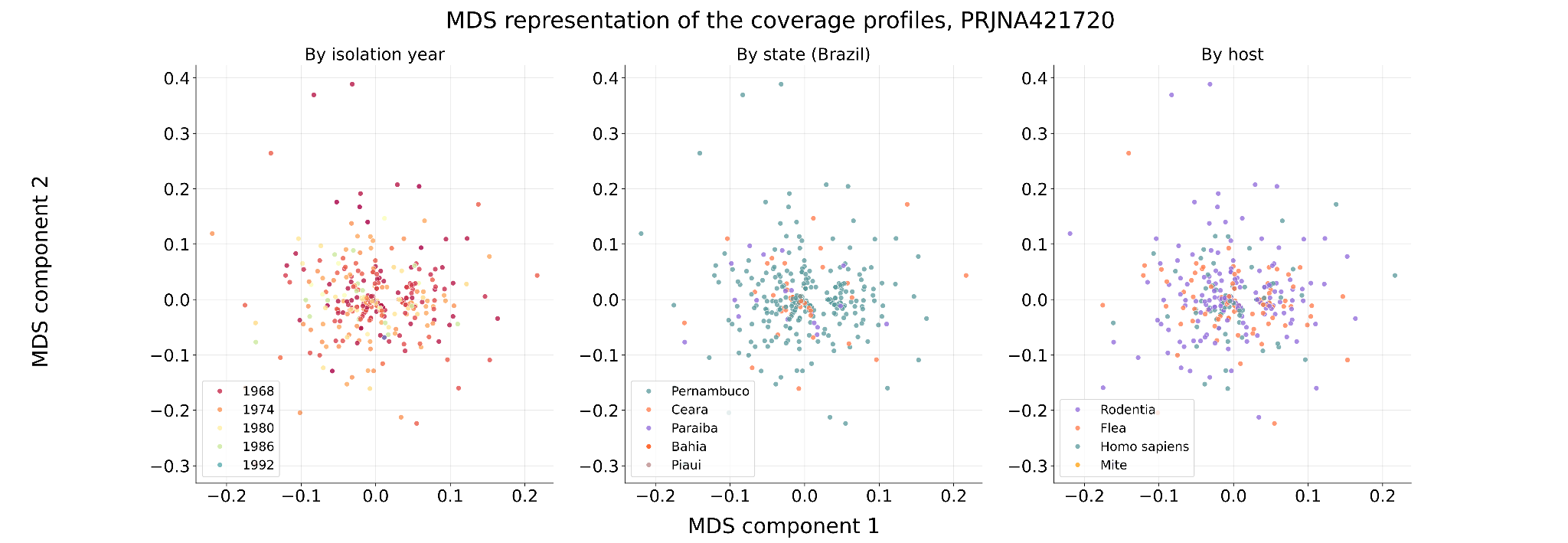
**

**Supplementary Figure 10.** MDS analysis of the samples from bioproject PRJNA421720.

**
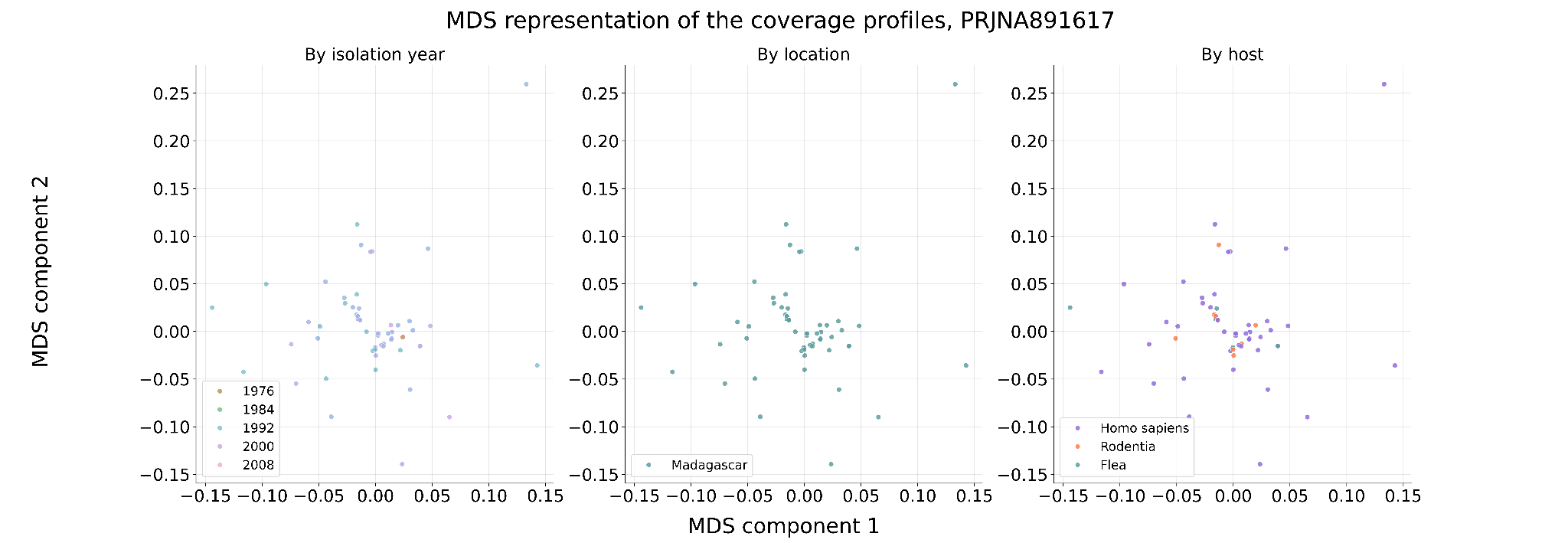
**

**Supplementary Figure 11.** MDS analysis of the samples from bioproject PRJNA891617.

**
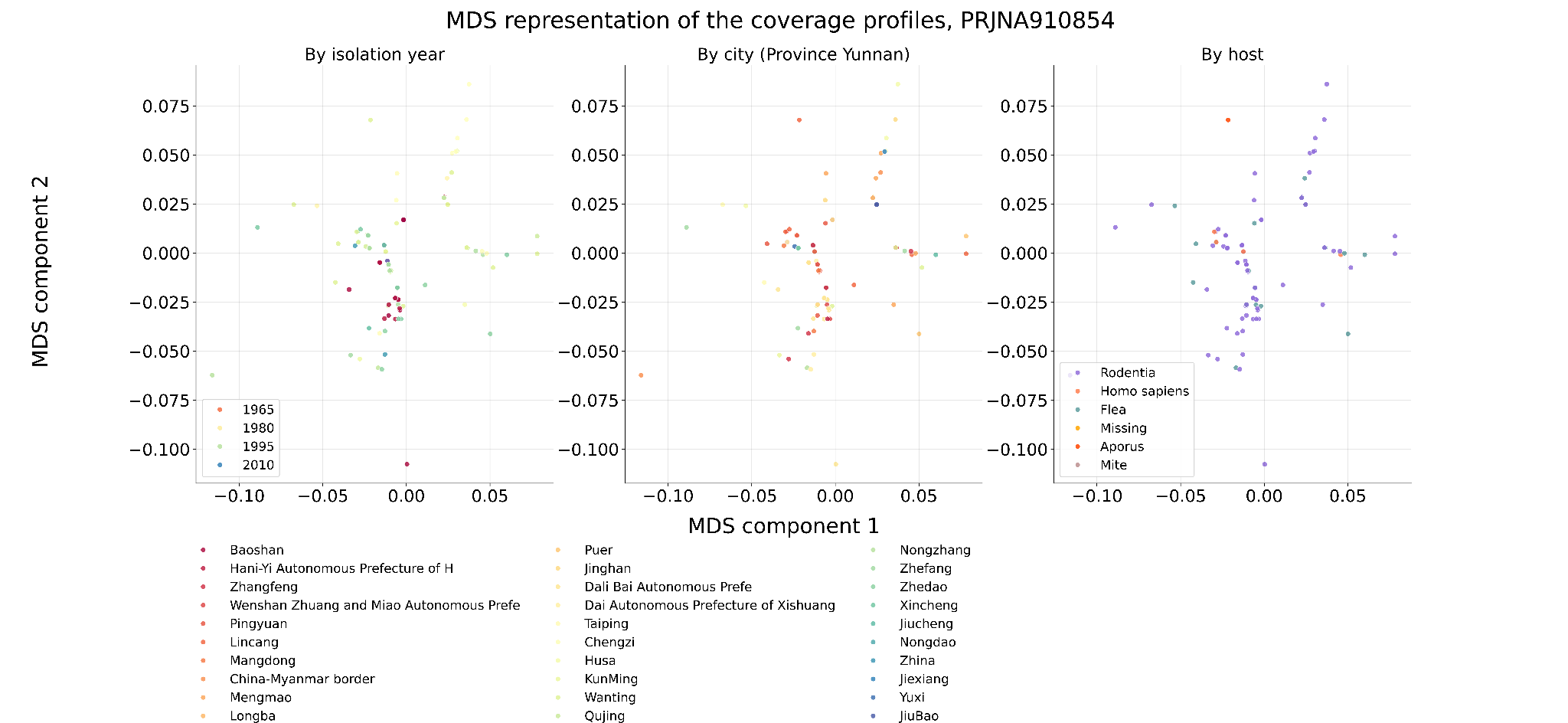
Supplementary Figure 12.** MDS analysis of the samples from bioproject PRJNA910854.

1. **Detection of chromosomal regions with a changed copy number.**

The algorithm has a number of hyperparameters:

***window_size*** = 5000

***step*** = 100

***upper_threshold*** = 1.25

***lower_threshold*** = 0.75

***min_length*** = 10000

The algorithm requires the results of “samtools depth -a” command as **an input**. Only the longest contig representing the chromosome is considered.

First, the algorithm initializes a vector *coverage_vector* and fills it with zeros. The size of the vector equals the length of the chromosome, so each vector feature represents a single chromosome position.

Next, the algorithm selects the first segment of the chromosome with length of ***window_size***, and calculates the median coverage depth in this segment (***segment_median***). This value is compared with the median coverage depth of the whole chromosome (***global_median***). If ***segment_median*** > ***global_median***upper_threshold***, or ***segment_median*** < ***global_median***lower_threshold****,* the algorithm changes corresponding values in ***coverege_vector*** to ones. Next, the algorithm moves to ***step*** positions in the chromosome, and considers a new segment.

After all segments have been considered, the binary vector ***coverege_vector*** consists of zeros and ones, where ones mean the positions with changed copy number. However, at this stage localization of zeros and ones in *coverege_vector* is rather noisy due to stochastic variance in the coverage depth. The next step is correction of ***coverage_vector*** by removing too short regions, and filling too short gaps between longer regions (both defined by the parameter ***min_length***). After the correction, ***coverege_vector*** contains long regions filled with ones and zeros. The positions of regions filled with ones are saved in BED-fomat, that is the algorithm **output**.

**Supplementary Figure 13** illustrates the main algorithm ideas.

\ **
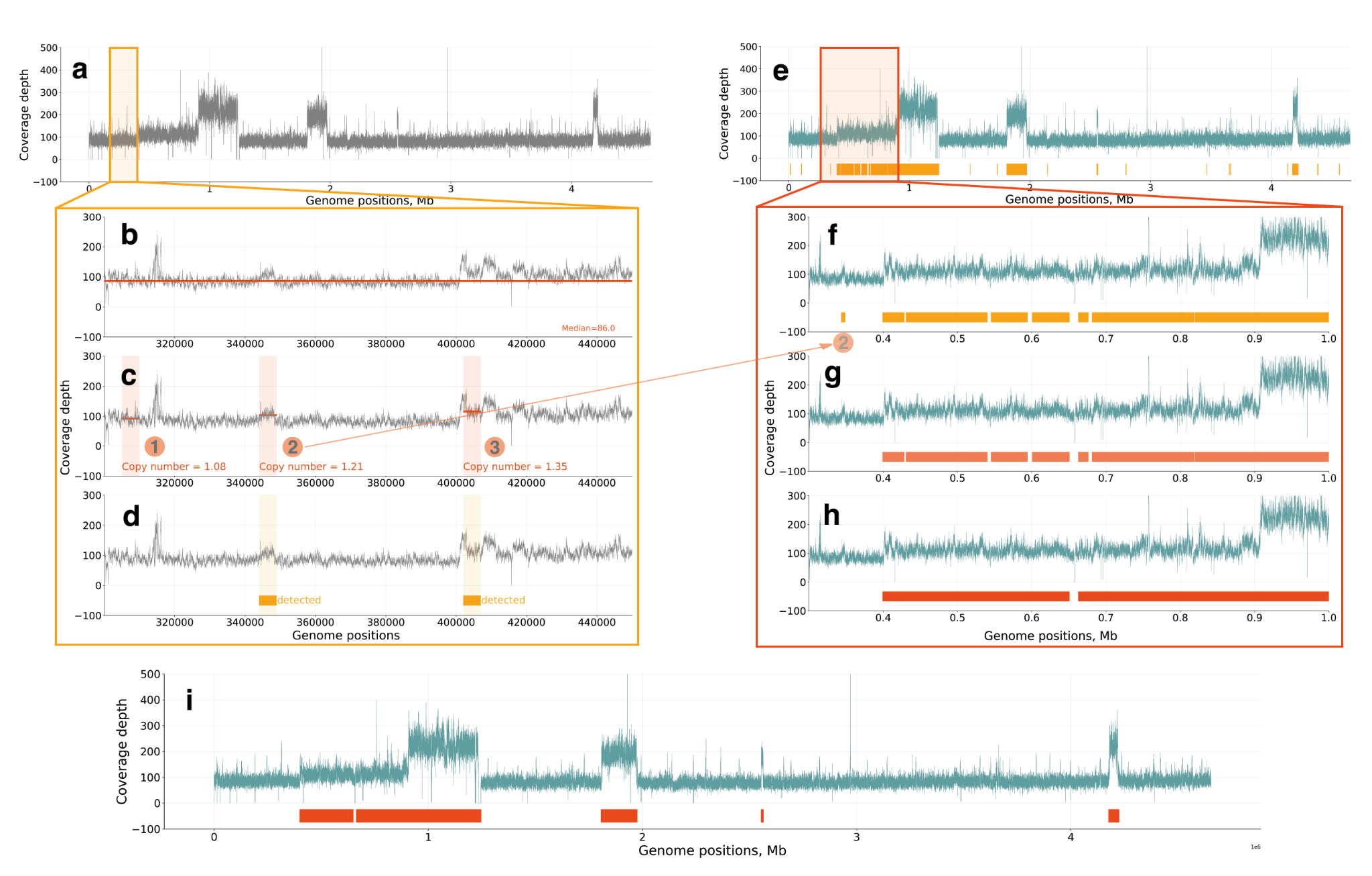
**

**Supplementary Figure 13.** The principal scheme of the detection of regions with a changed copy number. **a)** The input coverage depth profile provided by the “samtools depth -a” command. **b)** Calculation of the global median coverage. **c)** Calculation of the local coverage depth in all chromosomal segments. Only three segments are colored for illustration. Segments 2 and 3 satisfy the *upper_threshold* **=** 1.2, while region 1 does not. **d)** Only segments satisfying *upper_threshold*, are selected for next analysis. **e)** The whole chromosome with detected regions, and **f)** its fragment chosen for the further illustration. **g)** removing region 2 and other regions shorter than *min_length* **h)** Closing short gaps between long regions filed with ones. **i)** The final result of the algorithm (whole chromosome). Positions of the red regions are saved in BED-format.

1. **Optimization of the algorithm parameters.**

The parameters used in the algorithm described in Supplementary Materials 11, were preliminary optimized on a limited number of coverage profiles where the regions with changed copy number were assigned manually. For computational efficiency, the Grid Search approach was used to find the best combination of the parameters.

Initially, we manually selected 23 profiles from the PRJNA421720 and PRJNA891617 bioprojects based on two criteria: the length of regions with altered copy numbers and the highest difference between manual annotations and algorithmic results using default parameters. We used the Jaccard distance as an optimized metric. Jaccard distance (scipy.spatial.distance.jaccard) was calculated between manually assigned and predicted binary vectors with length of the genome size, where ones represented positions with changed copy number.

For each profile, all combinations of parameters were tested and evaluated. We created a score optimization table in which the value for each parameter set represented the cumulative scores of the profiles, inversely related to the Jaccard distance. The combination of parameters with the highest score in this optimization table was selected as the optimal set. The results indicated the best parameters were: ***context_length*** = 5000, ***step*** = 100, ***upper_threshold*** = 1.25, and ***lower_threshold*** = 0.75.

1. Cadastre of epidemic and epizootic manifestations of plague in the territory of the Russian Federation and neighboring countries (1876–2015). Edited by Kutyrev VV, Popova AYu. Saratov: “Amirit”. 2016 [↑](#footnote-ref-1)
2. Kutyrev VV, Eroshenko GA, Motin VL, Nosov NY, Krasnov JM, Kukleva LM, Nikiforov KA, Al'khova ZV, Oglodin EG, Guseva NP. Phylogeny and Classification of Yersinia pestis Through the Lens of Strains From the Plague Foci of Commonwealth of Independent States. Front Microbiol. 2018 May 25;9:1106. doi: 10.3389/fmicb.2018.01106. PMID: 29887859; PMCID: PMC5980970. [↑](#footnote-ref-2)
