# Supplementary material for: Systematic analysis of spontaneous tandem genome amplification events in Yersinia pestis
Source: PLoS One. 2025 Dec 31;20(12):e0338460. doi: 10.1371/journal.pone.0338460 (PMC12755819; doi:10.1371/journal.pone.0338460)
Supplement: S2 Data. — (PDF) [file pone.0338460.s003.pdf]

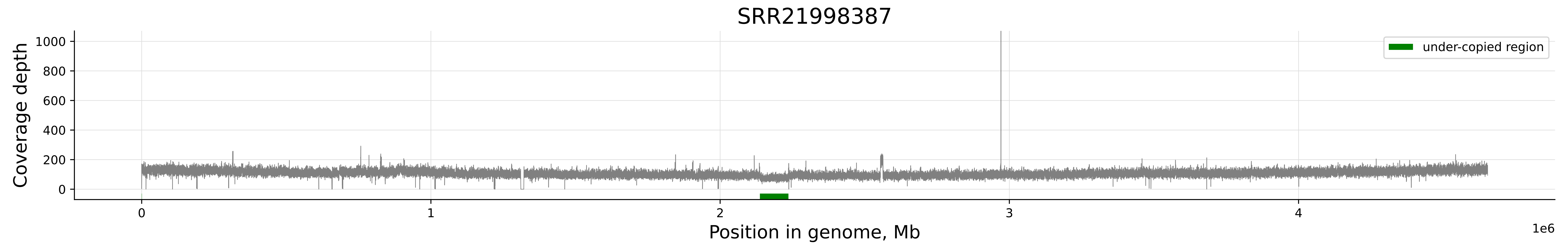
SRR21998387
Coverage depth
Position in genome, Mb
1e6
under-copied region
0
200
400
600
800
1000
0
1
2
3
4

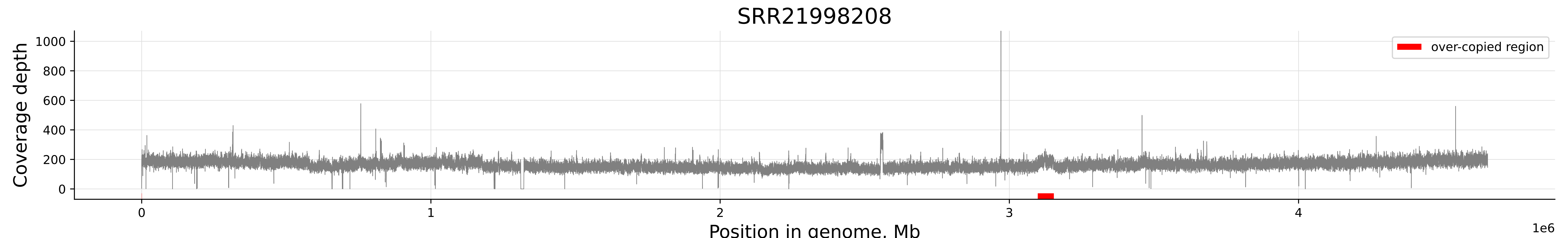
SRR21998208
Coverage depth
1000
800
600
400
200
0
over-copied region
0
1
2
3
4
1e6
Position in genome, Mb

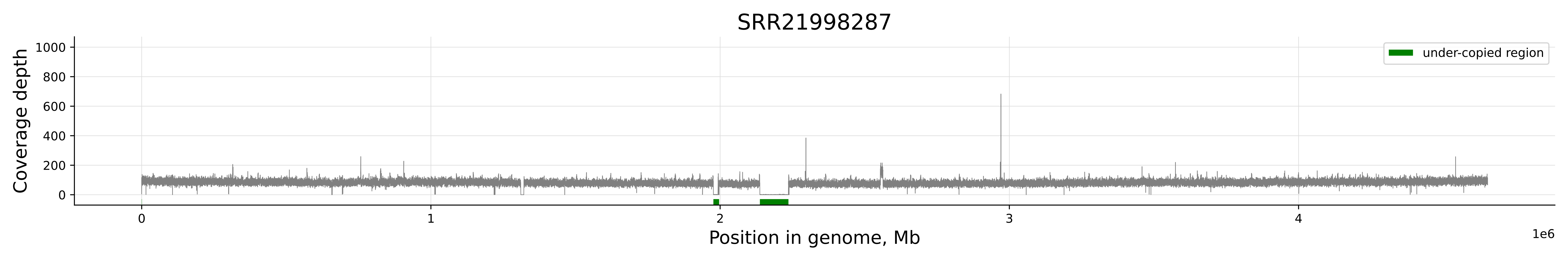

SRR21998287
Coverage depth
0
200
400
600
800
1000
under-copied region
0
1
2
3
4
1e6
Position in genome, Mb

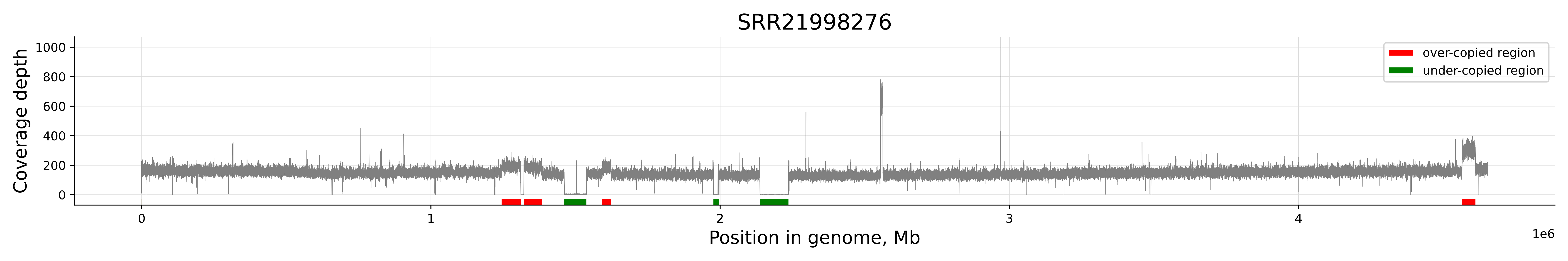

SRR21998276
Coverage depth
0
200
400
600
800
1000
Position in genome, Mb
0
1
2
3
4
1e6
over-copied region
under-copied region

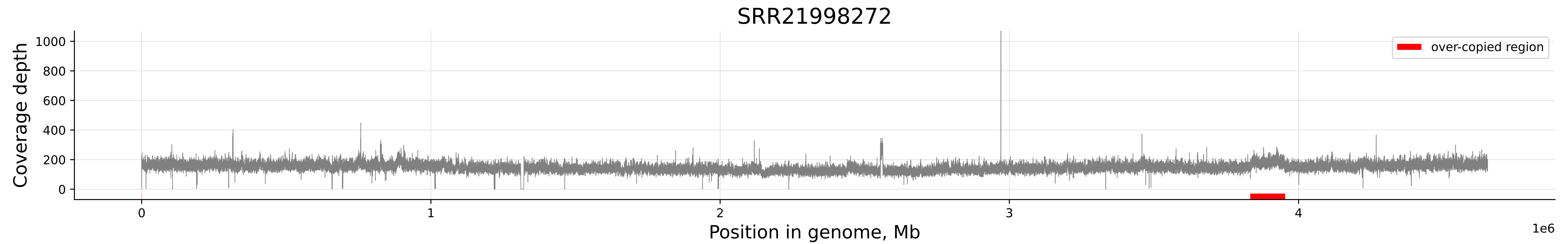
SRR21998272
Coverage depth
Position in genome, Mb
over-copied region
1e6

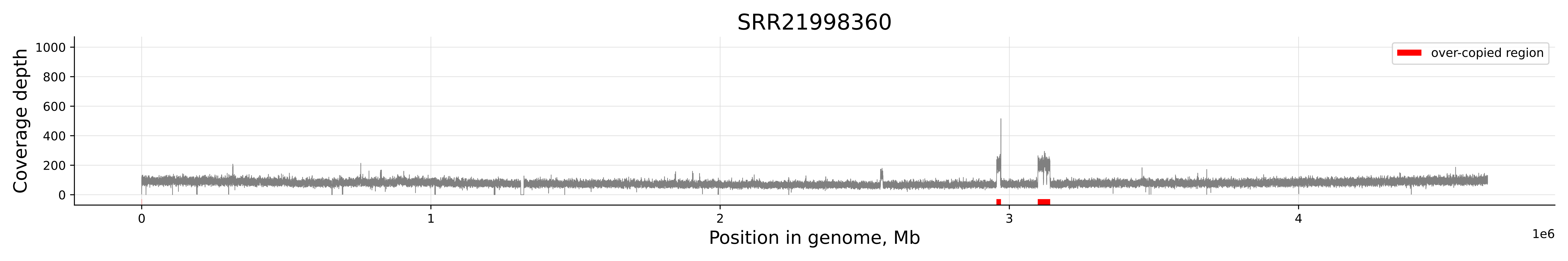

SRR21998360
Coverage depth
1000
800
600
400
200
0
over-copied region
0
1
2
3
4
1e6
Position in genome, Mb

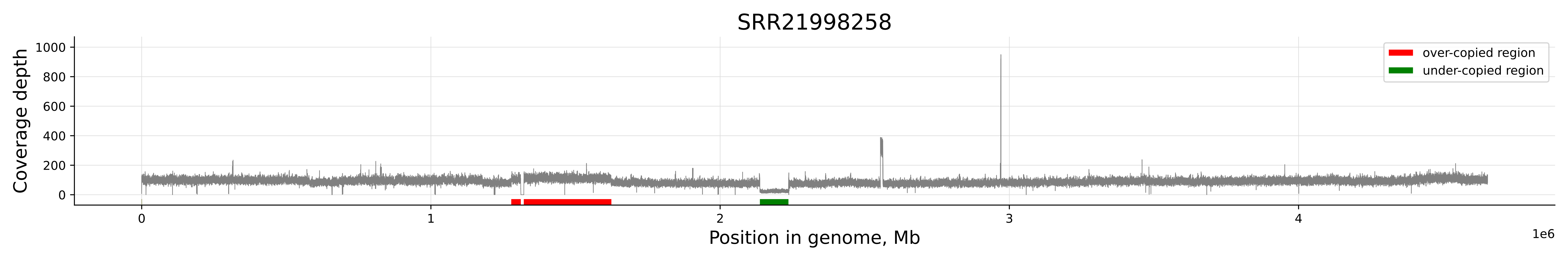

SRR21998258
Coverage depth
0
200
400
600
800
1000
0
1
2
3
4
1e6
Position in genome, Mb
over-copied region
under-copied region

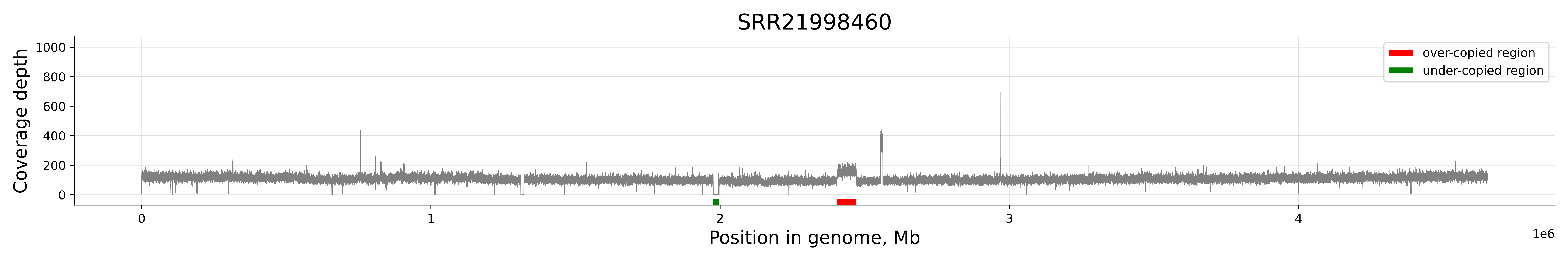
SRR21998460
Coverage depth
0
200
400
600
800
1000
0
1
2
3
4
1e6
Position in genome, Mb
over-copied region
under-copied region

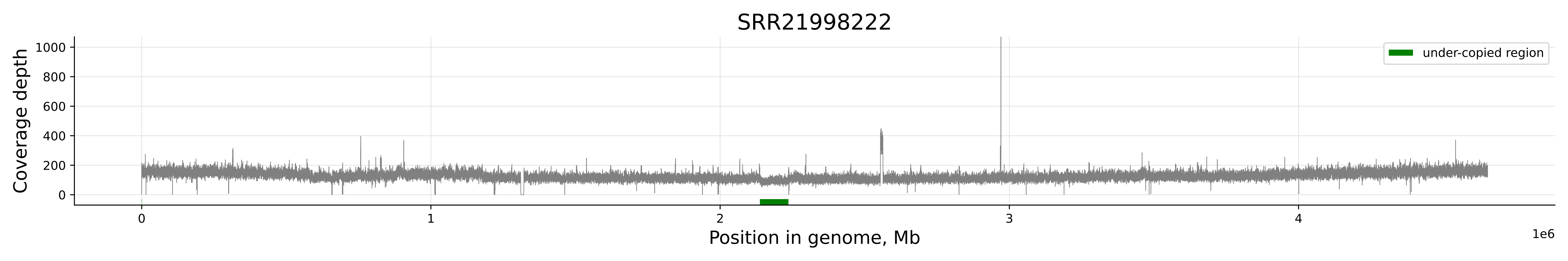
SRR21998222
Coverage depth
1000
800
600
400
200
0
under-copied region
0
1
2
3
4
1e6
Position in genome, Mb

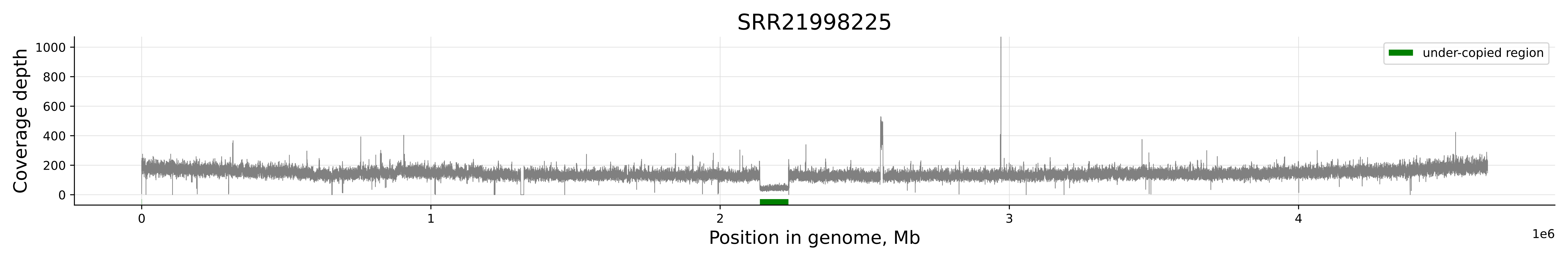
SRR21998225
Coverage depth
1000
800
600
400
200
0
under-copied region
0
1
2
3
4
1e6
Position in genome, Mb

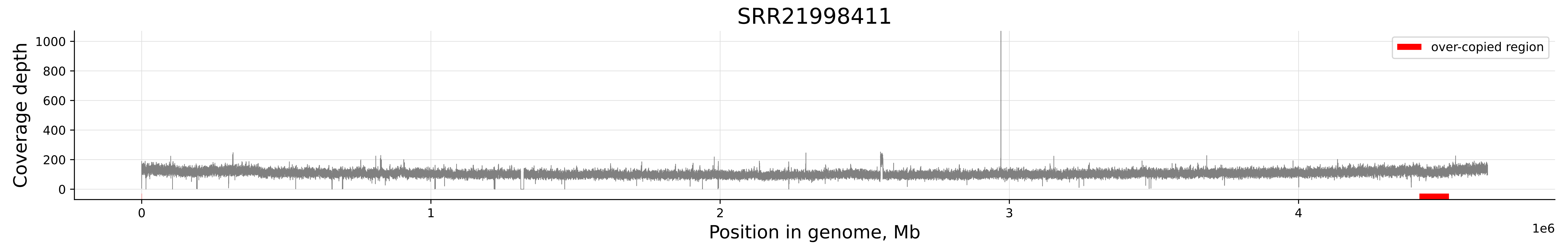

SRR21998411
over-copied region
Coverage depth
1000
800
600
400
200
0
0
1
2
3
4
1e6
Position in genome, Mb

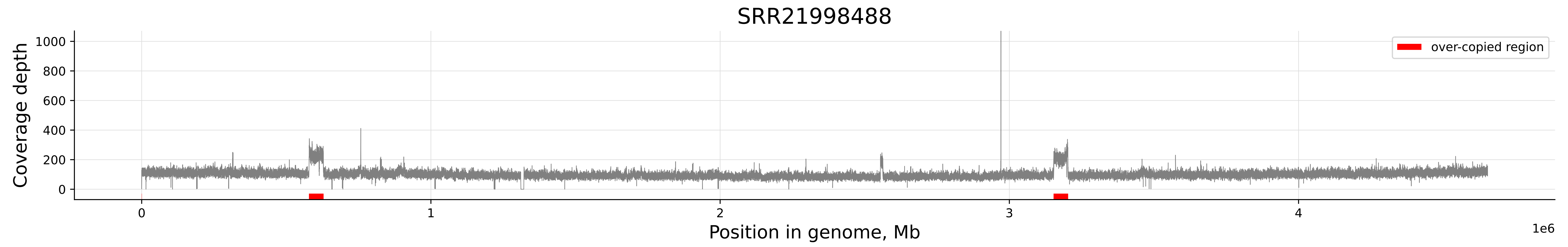

SRR21998488
Coverage depth
1000
800
600
400
200
0
over-copied region
0
1
2
3
4
1e6
Position in genome, Mb

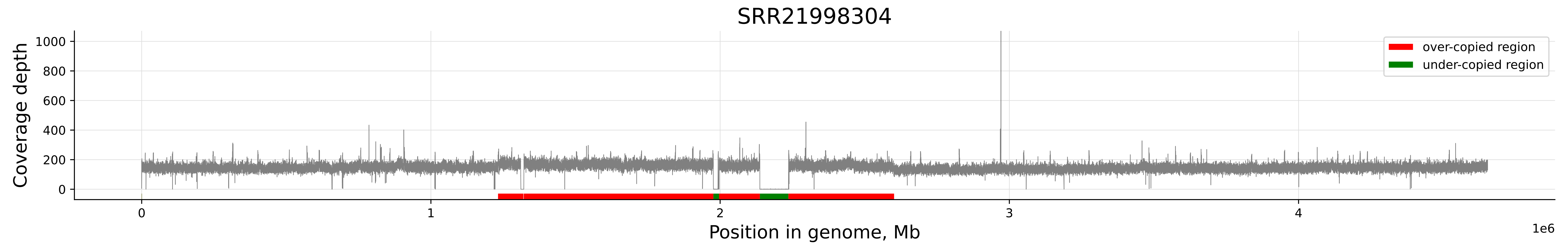

SRR21998304
Coverage depth
1000
800
600
400
200
0
over-copied region
under-copied region
0
1
2
3
4
1e6
Position in genome, Mb

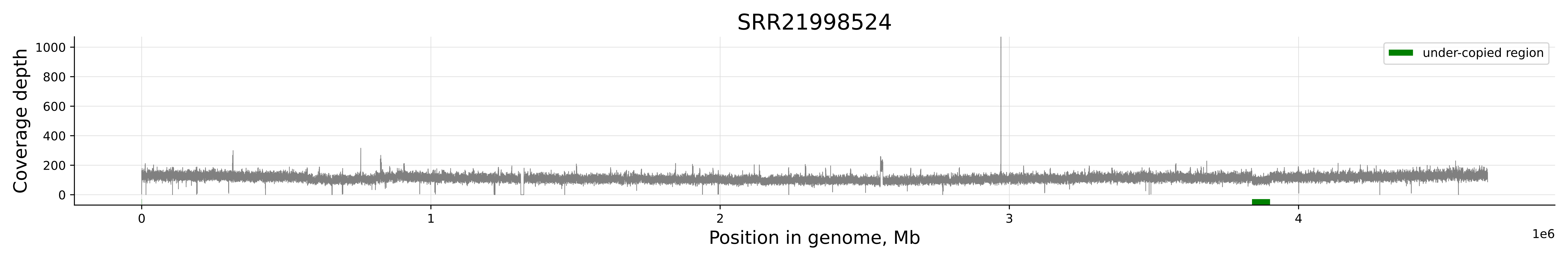
SRR21998524
Coverage depth
1000
800
600
400
200
0
under-copied region
0
1
2
3
4
Position in genome, Mb
1e6

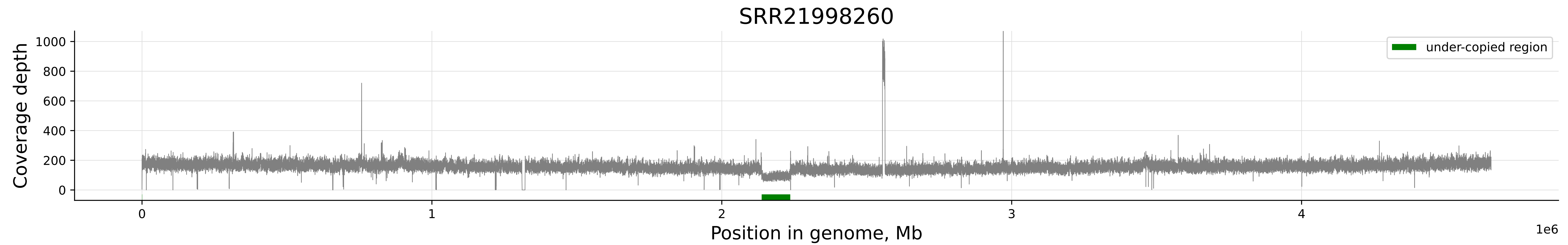
SRR21998260
under-copied region
Coverage depth
0
200
400
600
800
1000
0
1
2
3
4
1e6
Position in genome, Mb

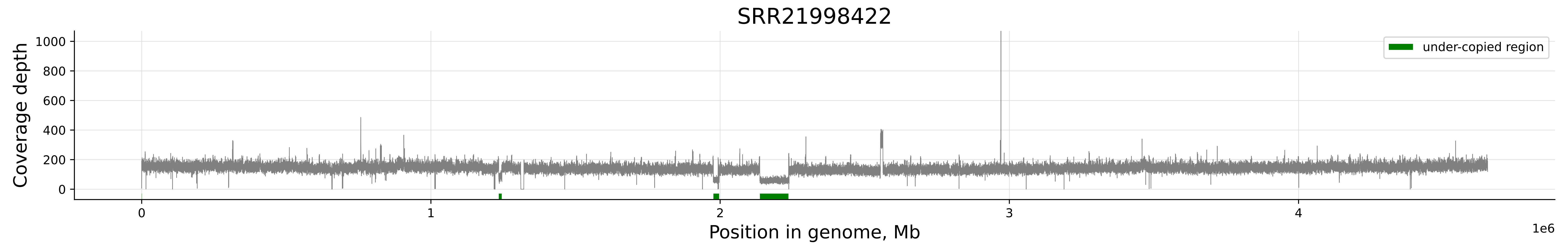
SRR21998422
Coverage depth
Position in genome, Mb
under-copied region
1e6
0
1
2
3
4
0
200
400
600
800
1000

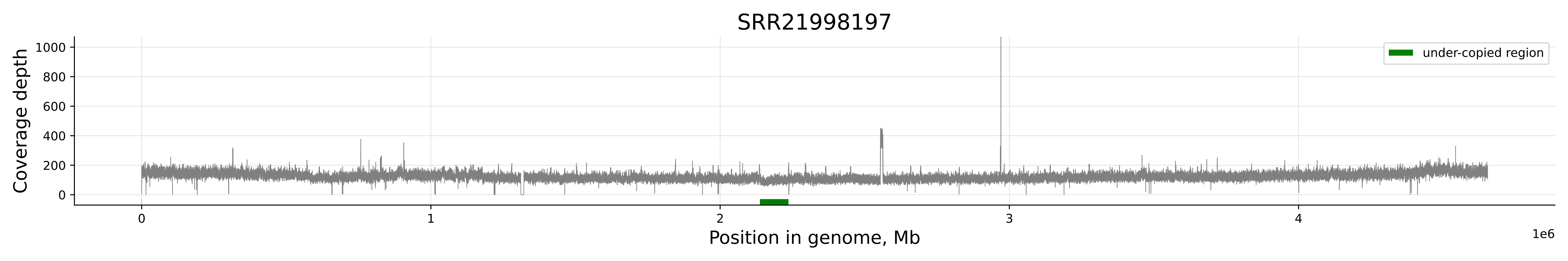

SRR21998197
under-copied region
Coverage depth
0
200
400
600
800
1000
0
1
2
3
4
1e6
Position in genome, Mb

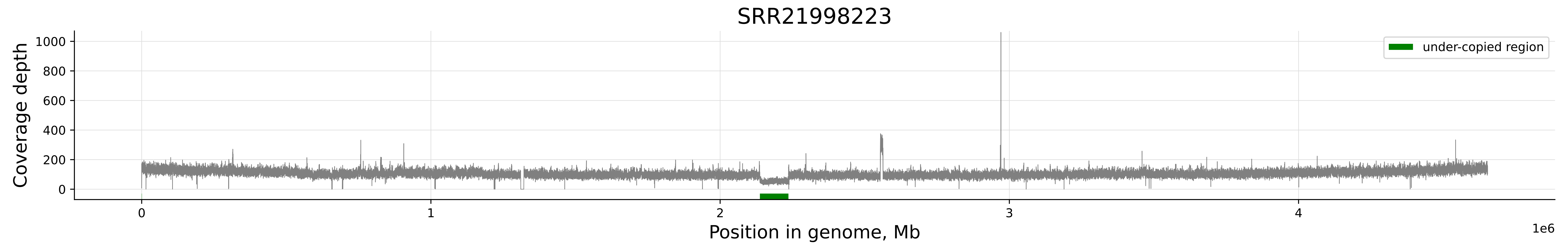

SRR21998223
Coverage depth
Position in genome, Mb
1e6
under-copied region
0
200
400
600
800
1000
0
1
2
3
4

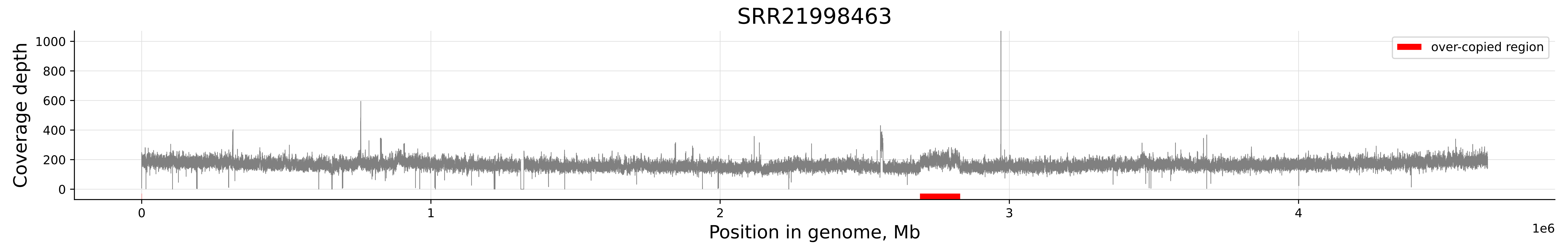
SRR21998463
Coverage depth
Position in genome, Mb
over-copied region
1e6

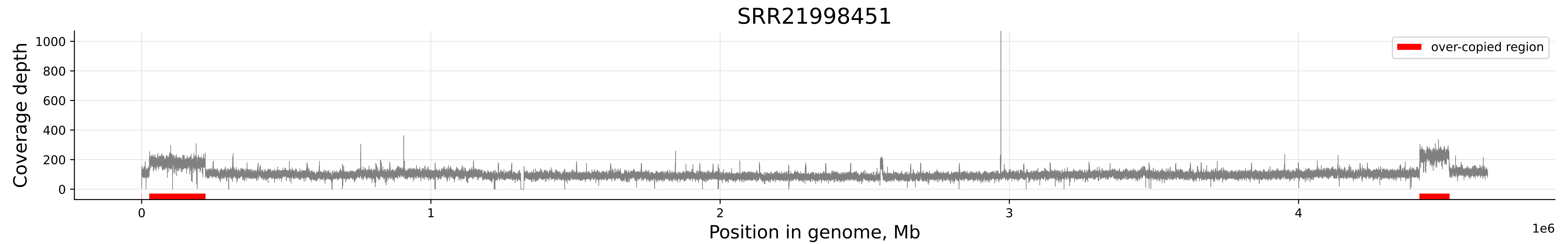

SRR21998451
Coverage depth
1000
800
600
400
200
0
over-copied region
0
1
2
3
4
1e6
Position in genome, Mb

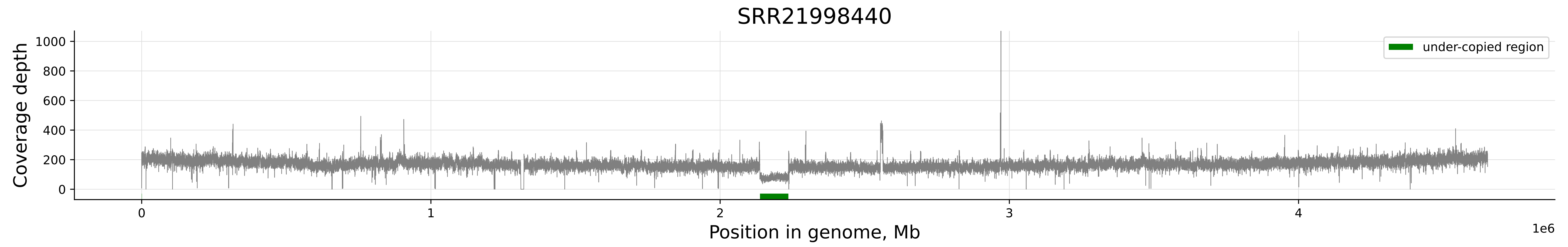
SRR21998440
under-copied region
Coverage depth
0
200
400
600
800
1000
0
1
2
3
4
1e6
Position in genome, Mb

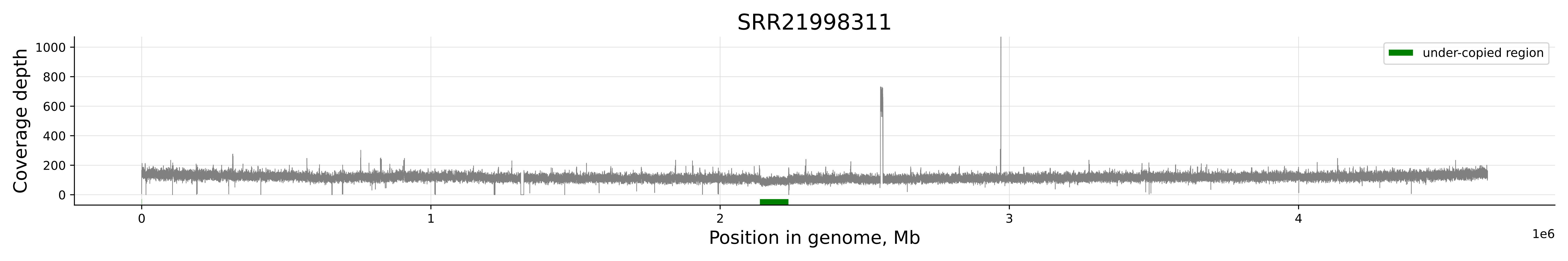
SRR21998311
Coverage depth
1000
800
600
400
200
0
0
1
2
3
4
1e6
Position in genome, Mb
under-copied region

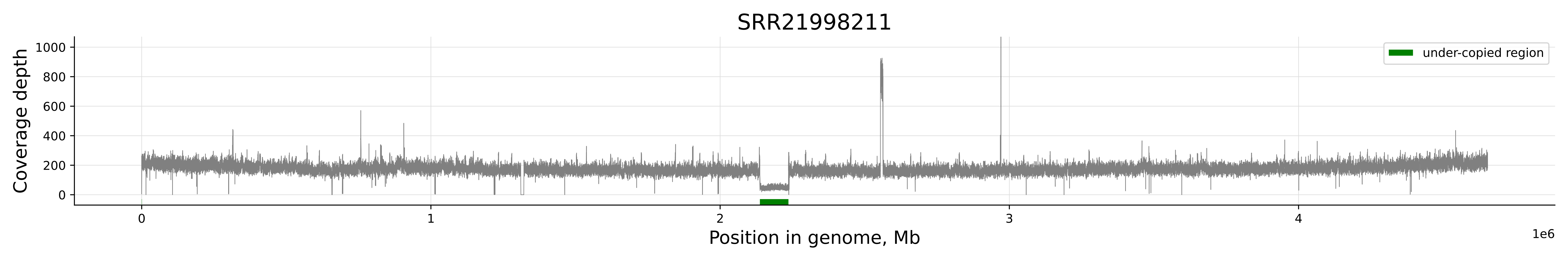
SRR21998211
Coverage depth
1000
800
600
400
200
0
0
1
2
3
4
1e6
Position in genome, Mb
under-copied region

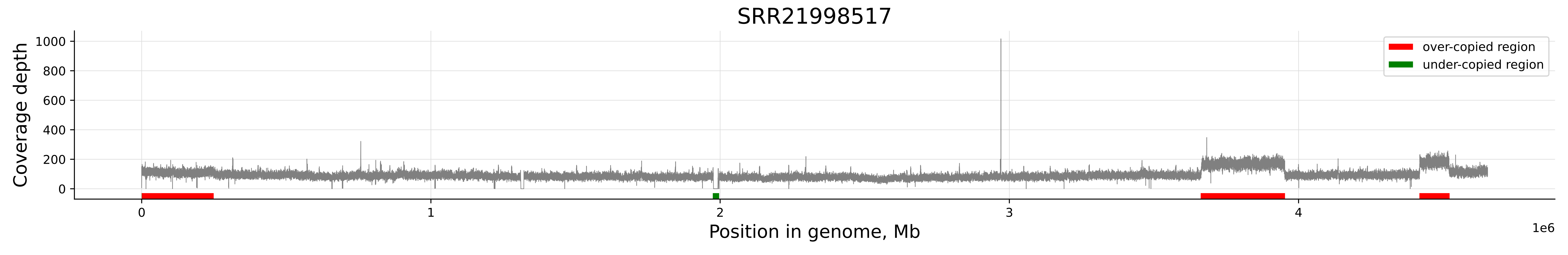
SRR21998517
Coverage depth
Position in genome, Mb
over-copied region
under-copied region
1e6

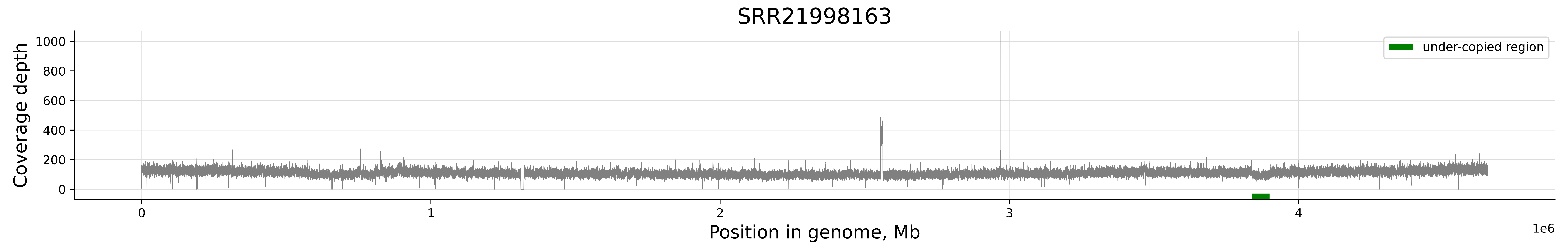
SRR21998163
Coverage depth
Position in genome, Mb
under-copied region
1000
800
600
400
200
0
1
2
3
4
1e6

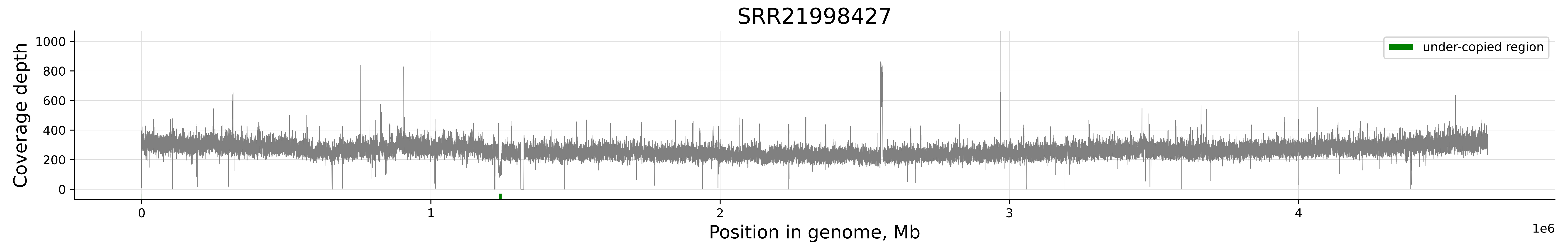

SRR21998427
Coverage depth
Position in genome, Mb
under-copied region
1000
800
600
400
200
0
0
1
2
3
4
1e6

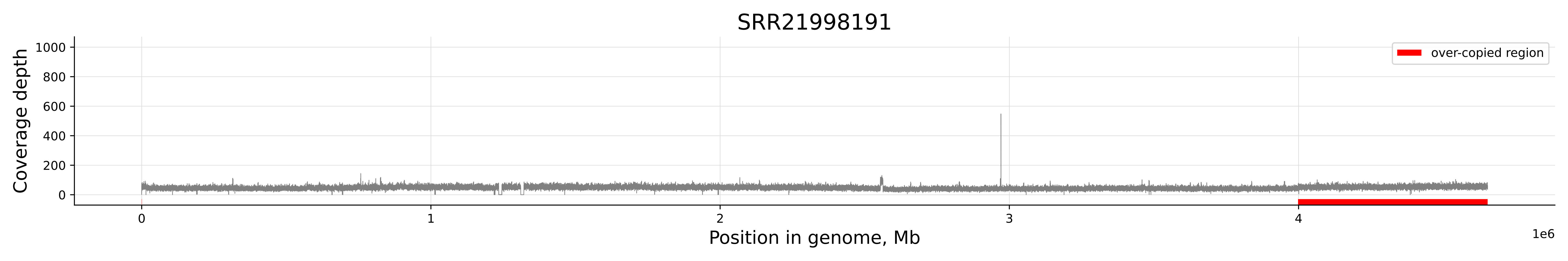

SRR21998191
Coverage depth
1000
800
600
400
200
0
0
1
2
3
4
1e6
Position in genome, Mb
over-copied region

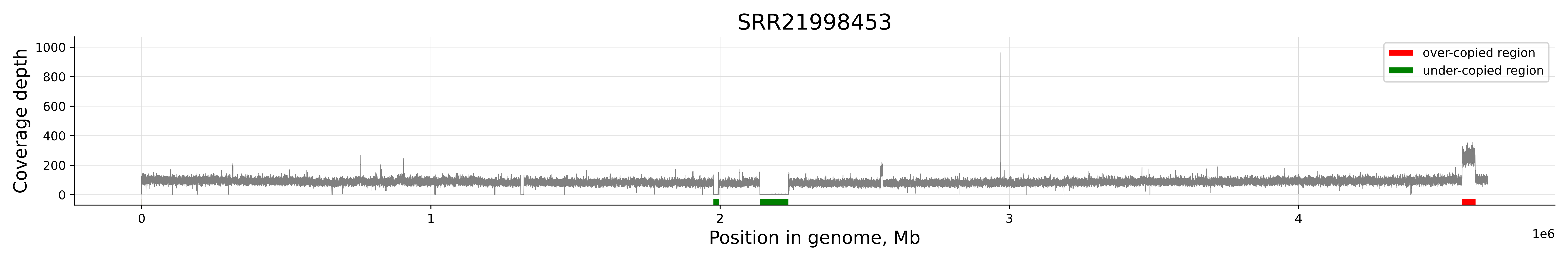
SRR21998453
Coverage depth
Position in genome, Mb
1e6
over-copied region
under-copied region
0
200
400
600
800
1000
0
1
2
3
4

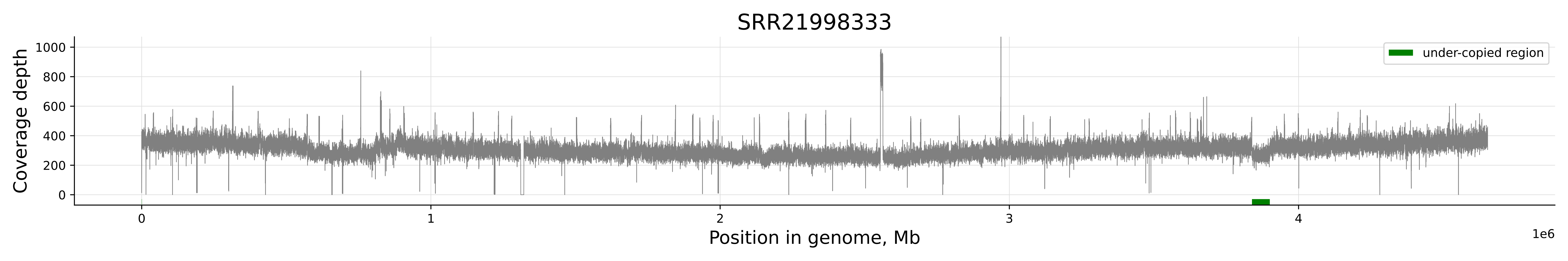
SRR21998333
under-copied region
Coverage depth
0
200
400
600
800
1000
0
1
2
3
4
1e6
Position in genome, Mb

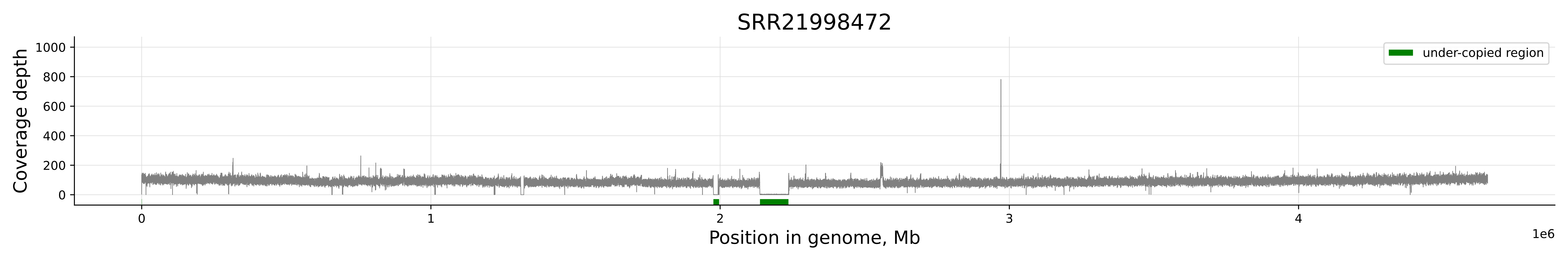

SRR21998472
under-copied region
Coverage depth
0
200
400
600
800
1000
0
1
2
3
4
1e6
Position in genome, Mb

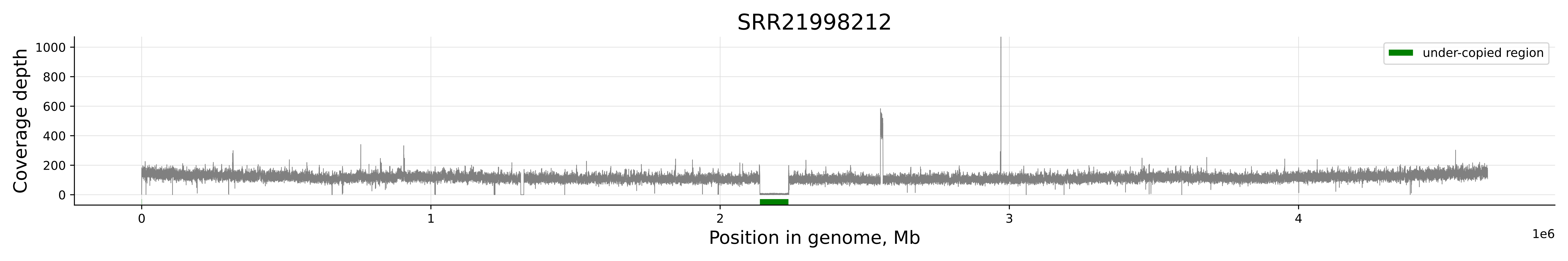
SRR21998212
Coverage depth
Position in genome, Mb
1e6
under-copied region
0
200
400
600
800
1000
0
1
2
3
4

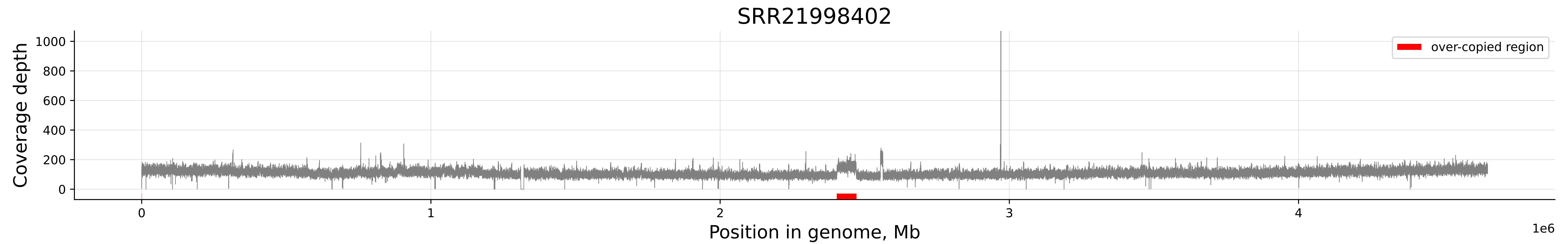

SRR21998402
Coverage depth
Position in genome, Mb
over-copied region
1e6
0
200
400
600
800
1000
0
1
2
3
4

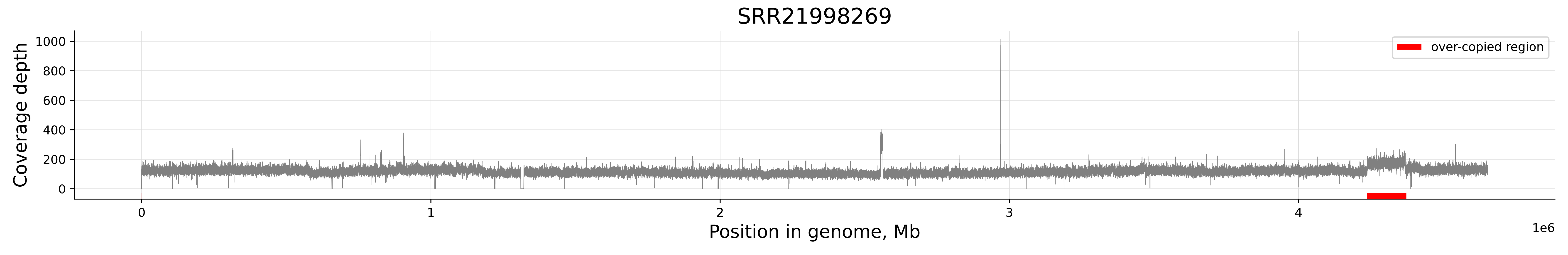
SRR21998269
Coverage depth
1000
800
600
400
200
0
over-copied region
0
1
2
3
4
Position in genome, Mb
1e6

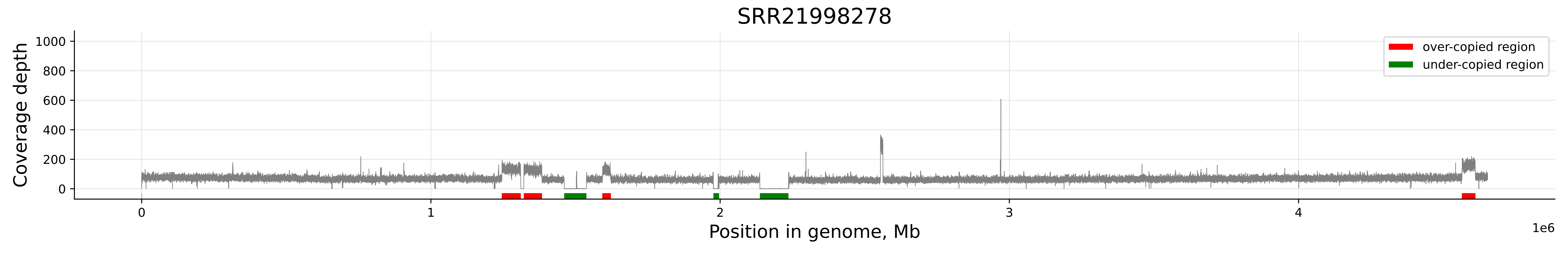
SRR21998278
Coverage depth
Position in genome, Mb
1e6
over-copied region
under-copied region
0
200
400
600
800
1000
0
1
2
3
4

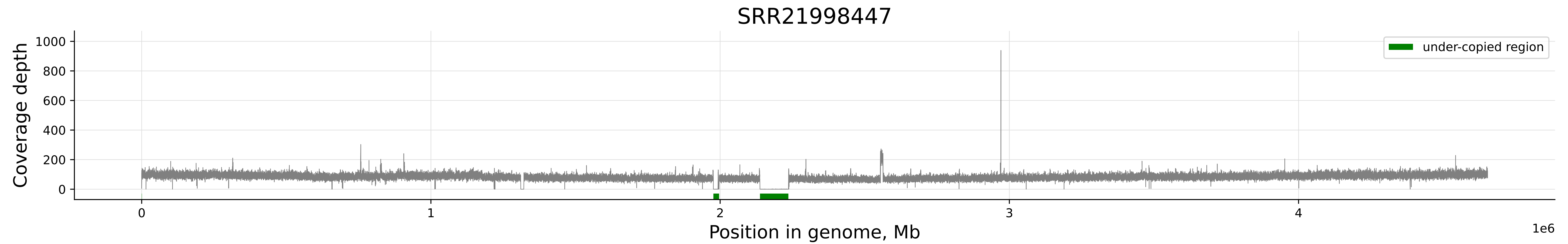

SRR21998447
Coverage depth
Position in genome, Mb
under-copied region
1e6

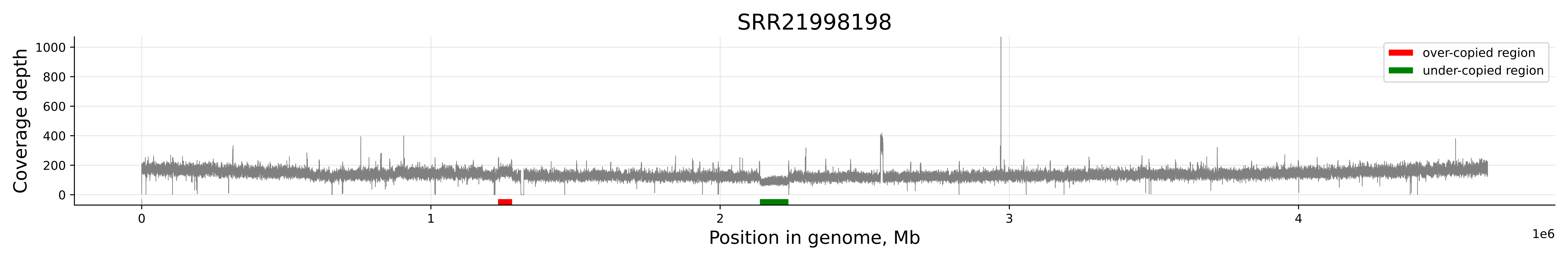
SRR21998198
Coverage depth
Position in genome, Mb
over-copied region
under-copied region
1e6

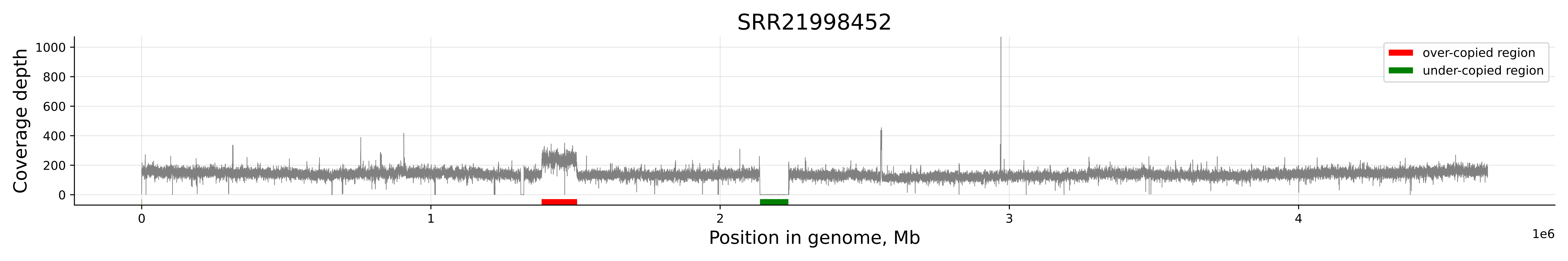

SRR21998452
Coverage depth
0
200
400
600
800
1000
0
1
2
3
4
1e6
Position in genome, Mb
over-copied region
under-copied region

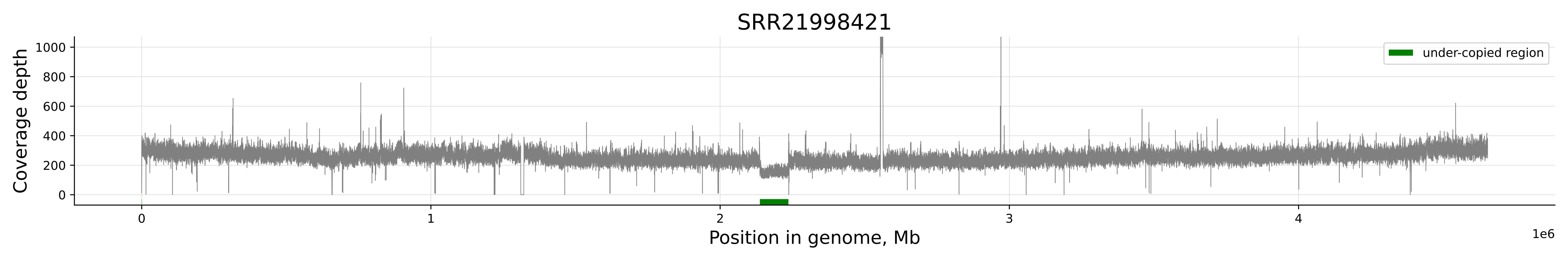

SRR21998421
under-copied region
Coverage depth
0
200
400
600
800
1000
0
1
2
3
4
1e6
Position in genome, Mb

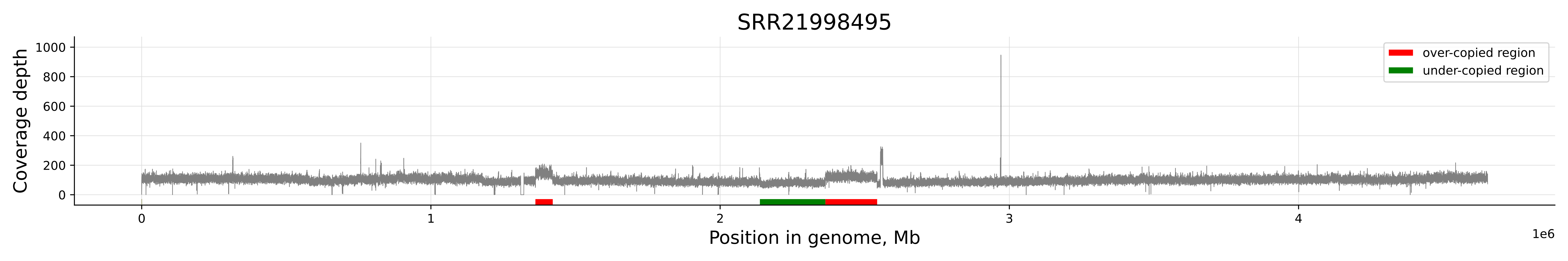
SRR21998495
Coverage depth
1000
800
600
400
200
0
0
1
2
3
4
1e6
Position in genome, Mb
over-copied region
under-copied region

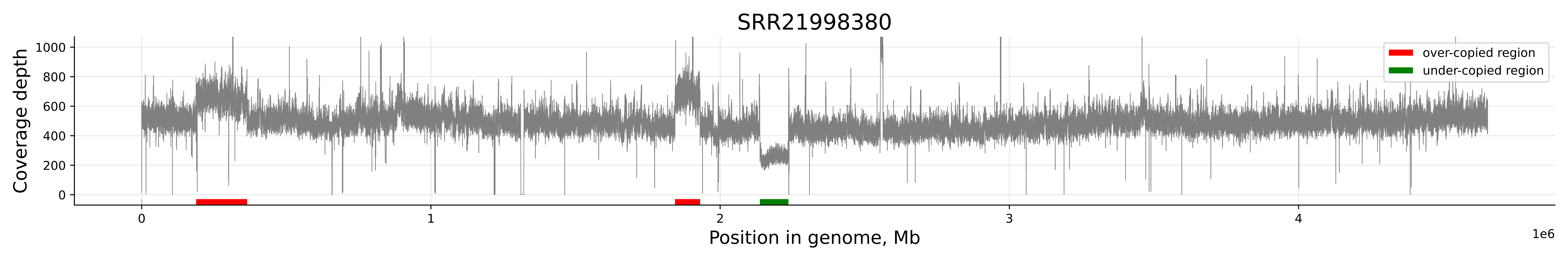

SRR21998380
Coverage depth
1000
800
600
400
200
0
0
1
2
3
4
1e6
Position in genome, Mb
over-copied region
under-copied region

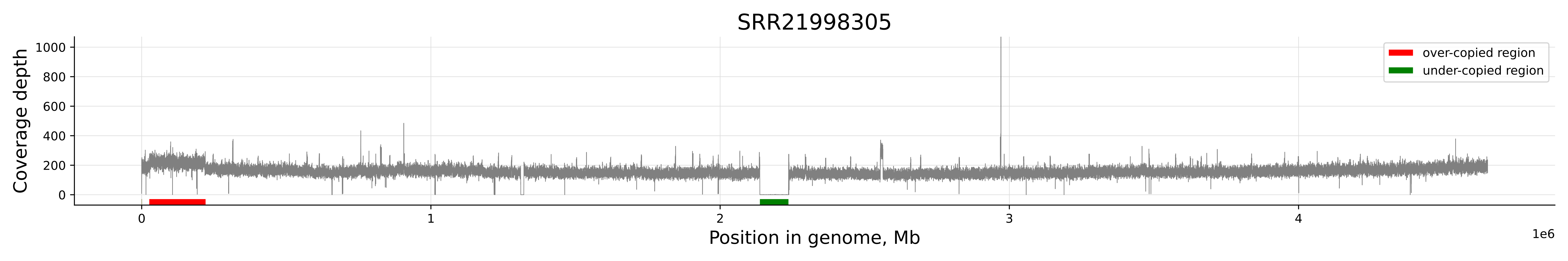
SRR21998305
Coverage depth
1000
800
600
400
200
0
0
1
2
3
4
1e6
Position in genome, Mb
over-copied region
under-copied region

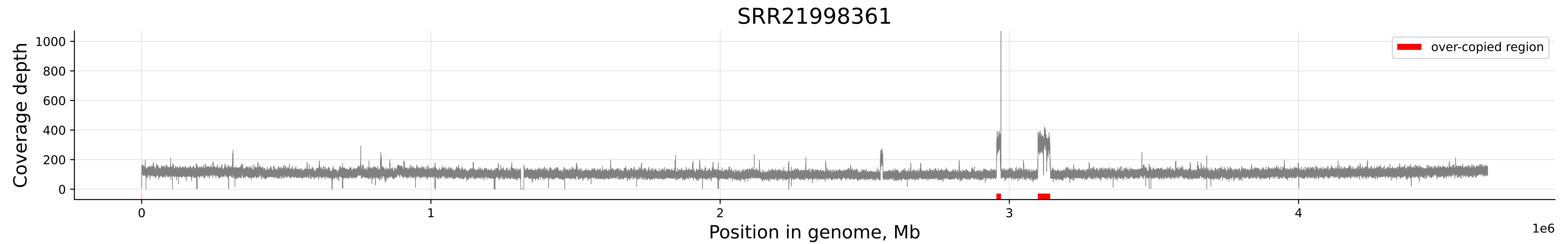

SRR21998361
Coverage depth
1000
800
600
400
200
0
over-copied region
0
1
2
3
4
1e6
Position in genome, Mb

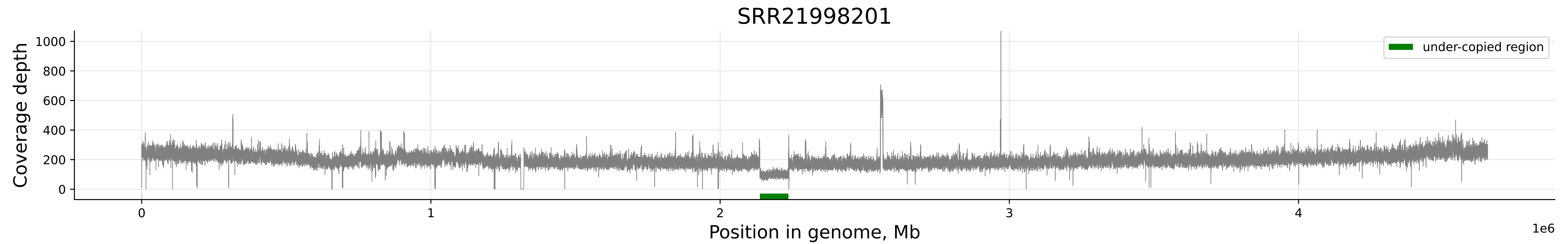

SRR21998201
under-copied region
Coverage depth
0
200
400
600
800
1000
0
1
2
3
4
1e6
Position in genome, Mb

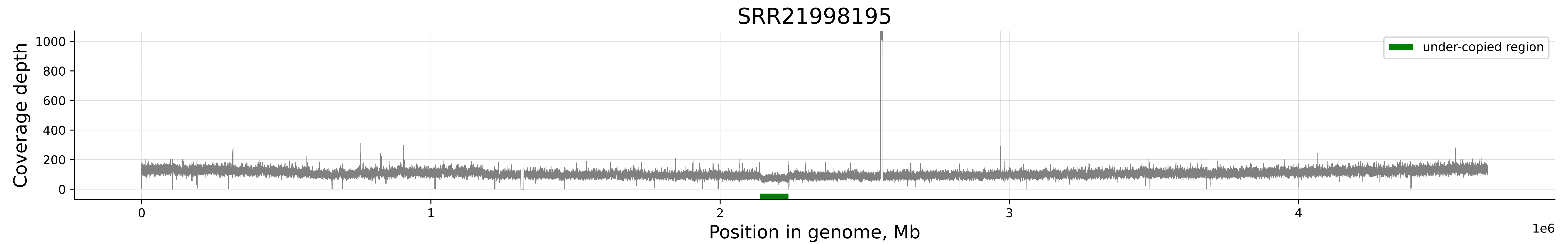

SRR21998195
Coverage depth
1000
800
600
400
200
0
under-copied region
0
1
2
3
4
Position in genome, Mb
1e6

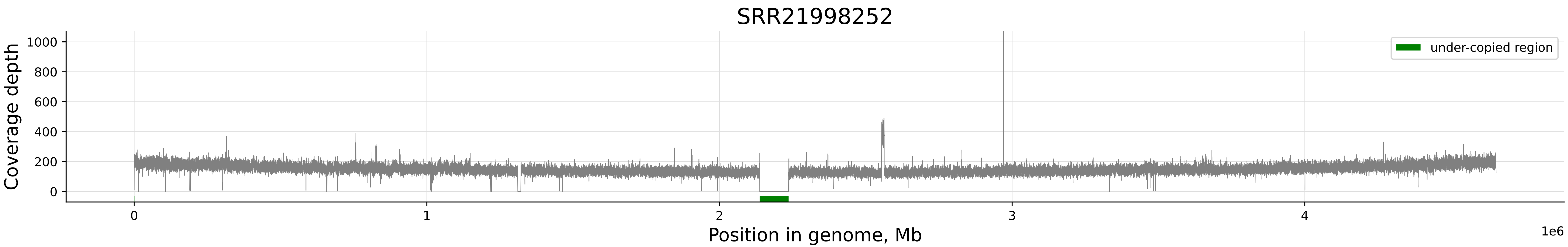

SRR21998252
Coverage depth
0
200
400
600
800
1000
under-copied region
0
1
2
3
4
1e6
Position in genome, Mb

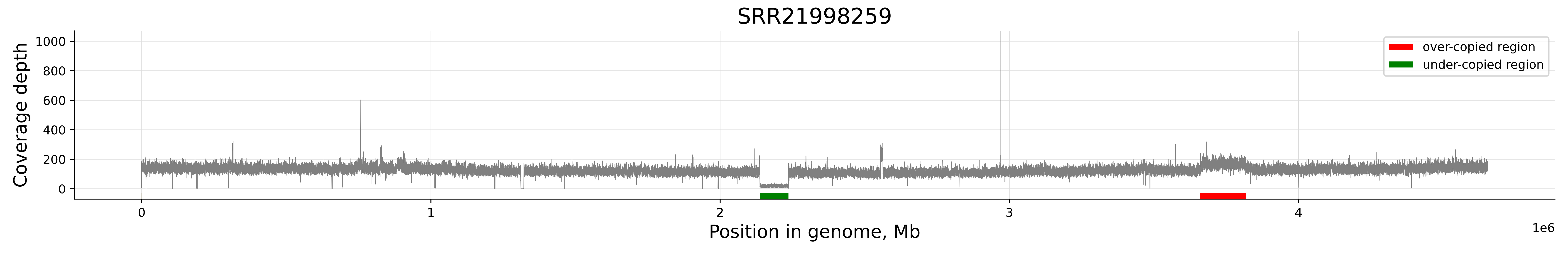
SRR21998259
Coverage depth
Position in genome, Mb
1e6
over-copied region
under-copied region
0
200
400
600
800
1000
0
1
2
3
4

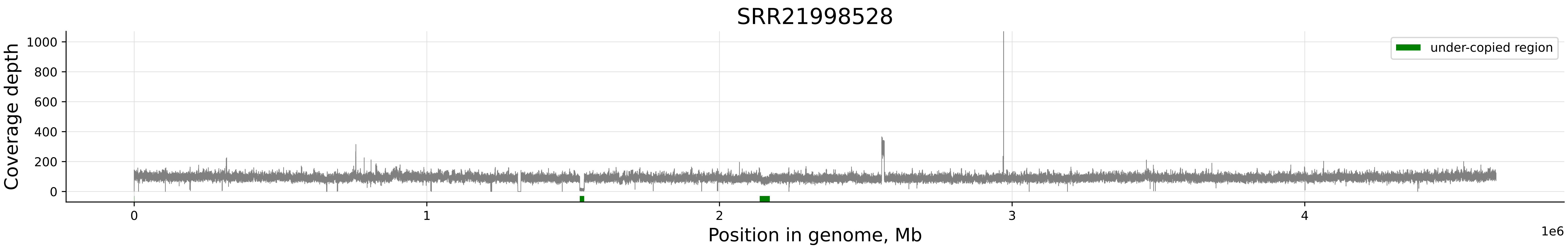
SRR21998528
under-copied region
Coverage depth
1000
800
600
400
200
0
0
1
2
3
4
1e6
Position in genome, Mb

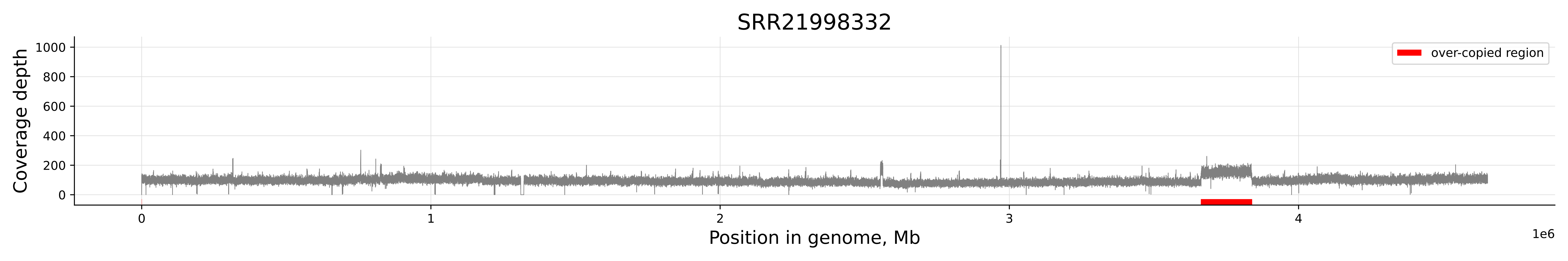
SRR21998332
Coverage depth
Position in genome, Mb
over-copied region
1000
800
600
400
200
0
0
1
2
3
4
1e6

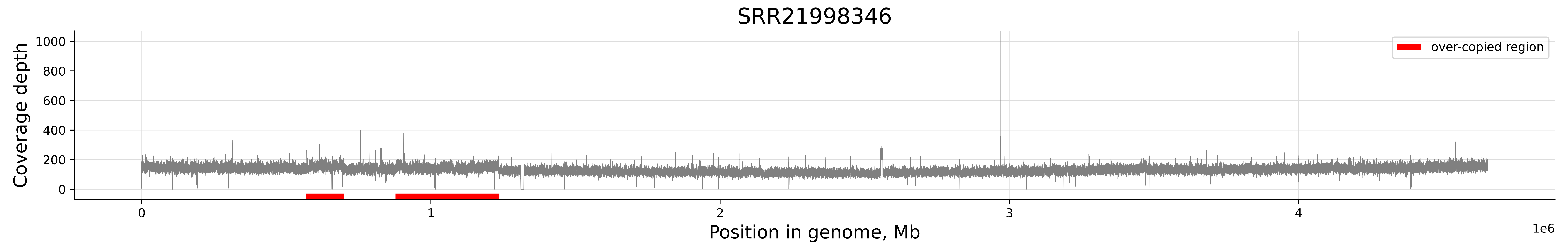
SRR21998346
Coverage depth
1000
800
600
400
200
0
0
1
2
3
4
1e6
Position in genome, Mb
over-copied region

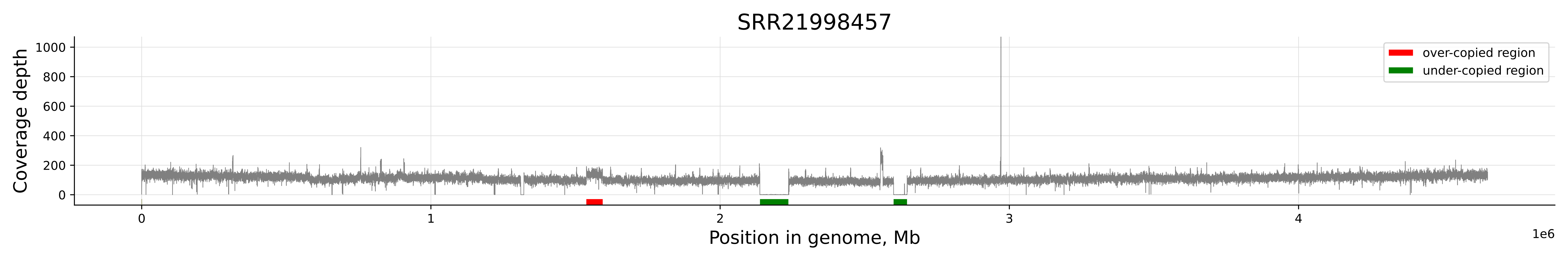
SRR21998457
Coverage depth
Position in genome, Mb
1e6
over-copied region
under-copied region
0
200
400
600
800
1000
1
2
3
4

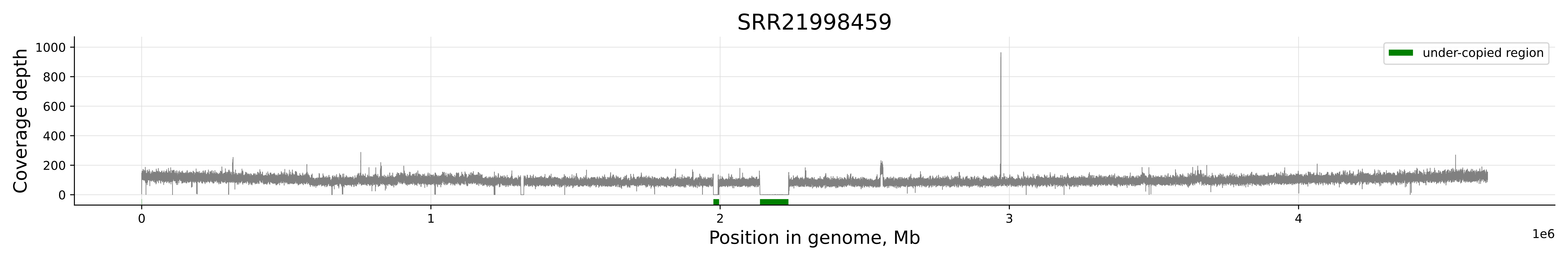
SRR21998459
Coverage depth
Position in genome, Mb
under-copied region
1e6
0
200
400
600
800
1000
1
2
3
4

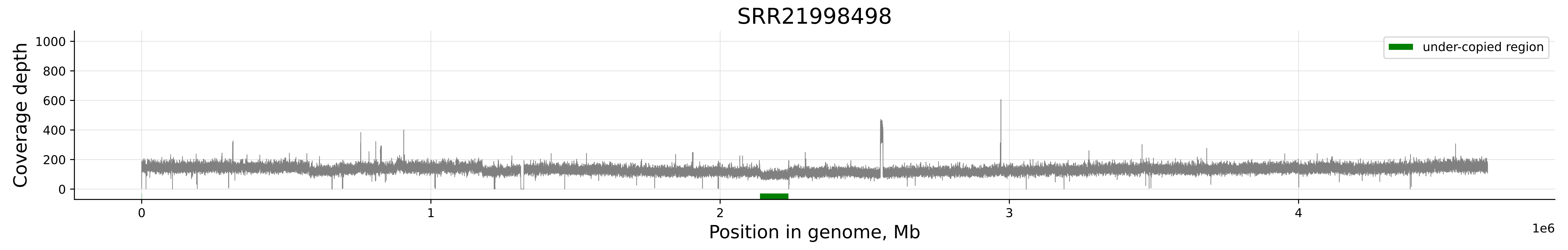
SRR21998498
Coverage depth
1000
800
600
400
200
0
under-copied region
0
1
2
3
4
1e6
Position in genome, Mb

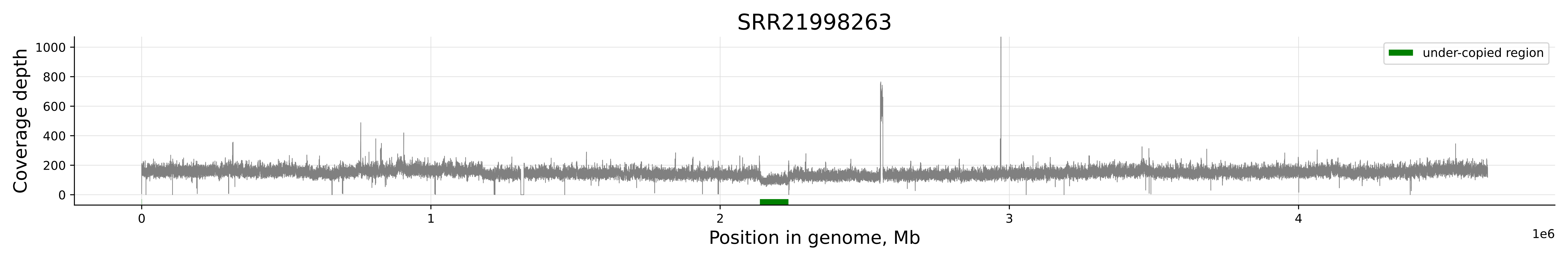

SRR21998263
Coverage depth
1000
800
600
400
200
0
under-copied region
0
1
2
3
4
Position in genome, Mb
1e6

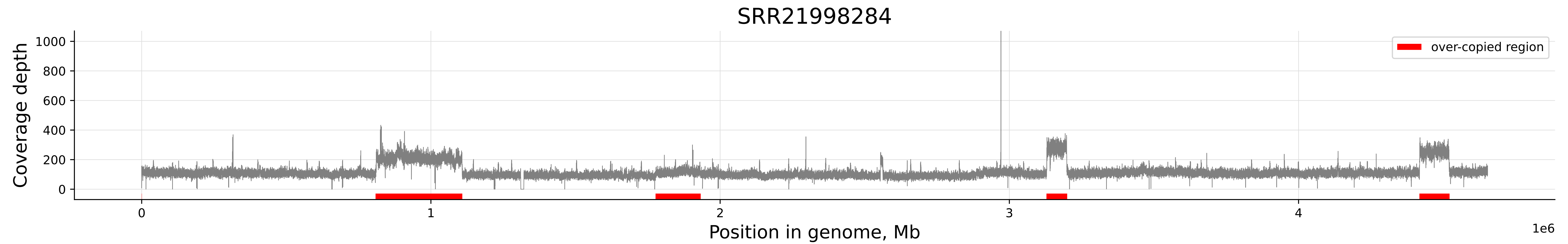

SRR21998284
Coverage depth
1000
800
600
400
200
0
0
1
2
3
4
1e6
Position in genome, Mb
over-copied region

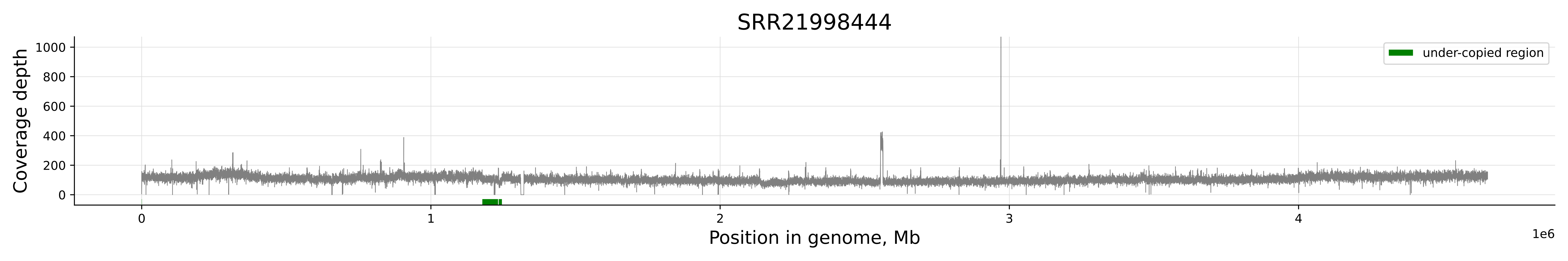
SRR21998444
Coverage depth
Position in genome, Mb
under-copied region
1e6

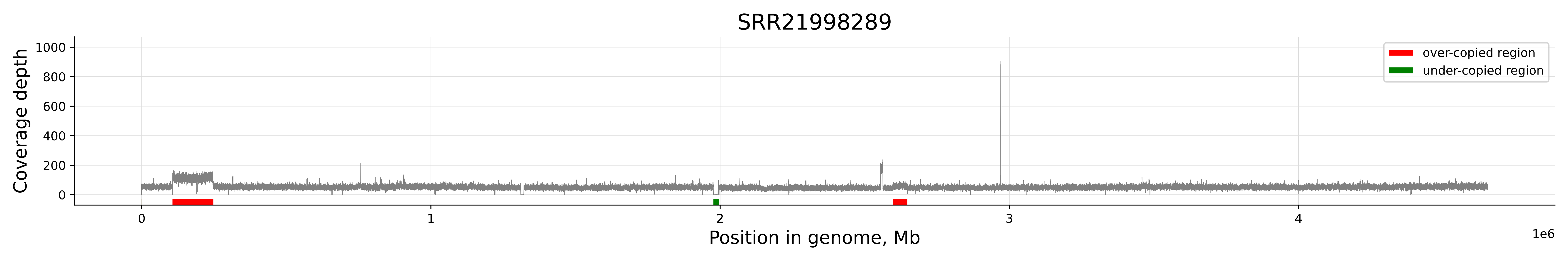
SRR21998289
Coverage depth
Position in genome, Mb
1e6
over-copied region
under-copied region
0
200
400
600
800
1000
0
1
2
3
4

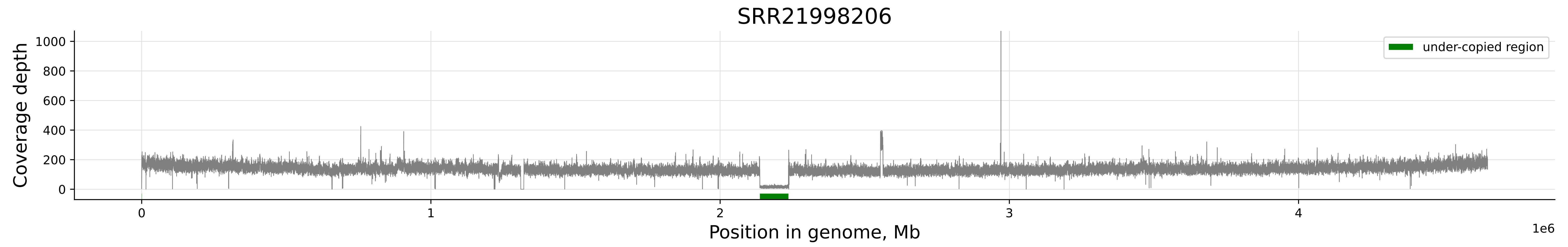
SRR21998206
under-copied region
Coverage depth
1000
800
600
400
200
0
0
1
2
3
4
1e6
Position in genome, Mb

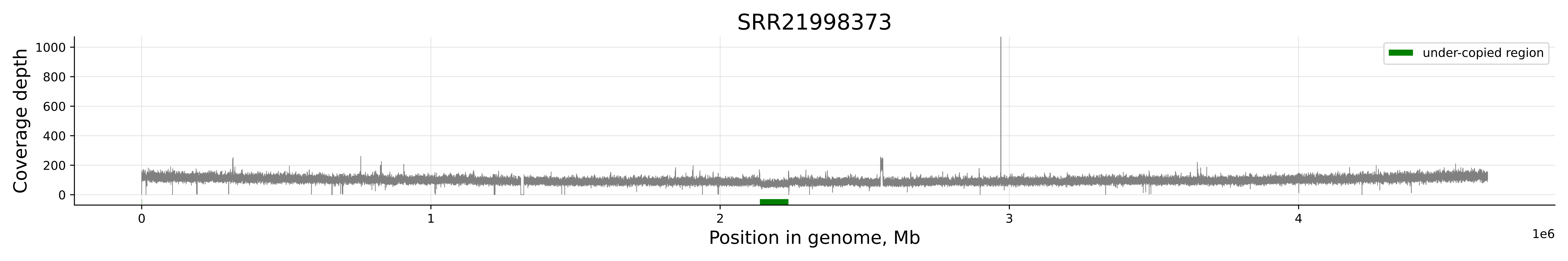
SRR21998373
Coverage depth
Position in genome, Mb
1e6
under-copied region
0
200
400
600
800
1000
0
1
2
3
4

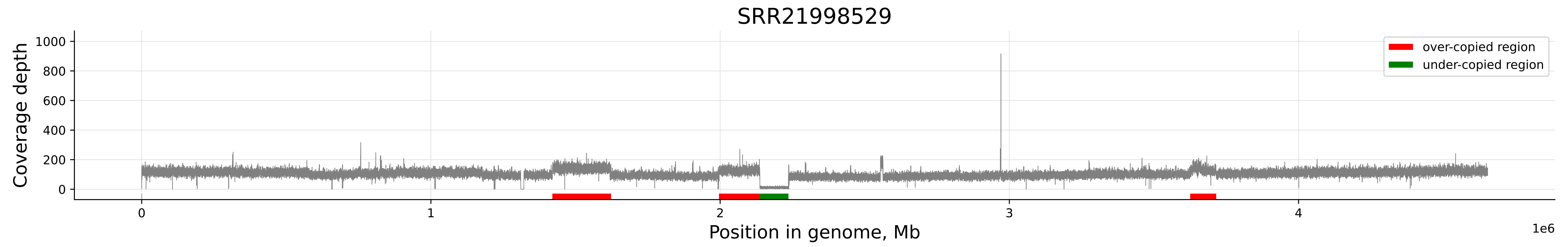
SRR21998529
Coverage depth
Position in genome, Mb
1e6
over-copied region
under-copied region
0
200
400
600
800
1000
0
1
2
3
4

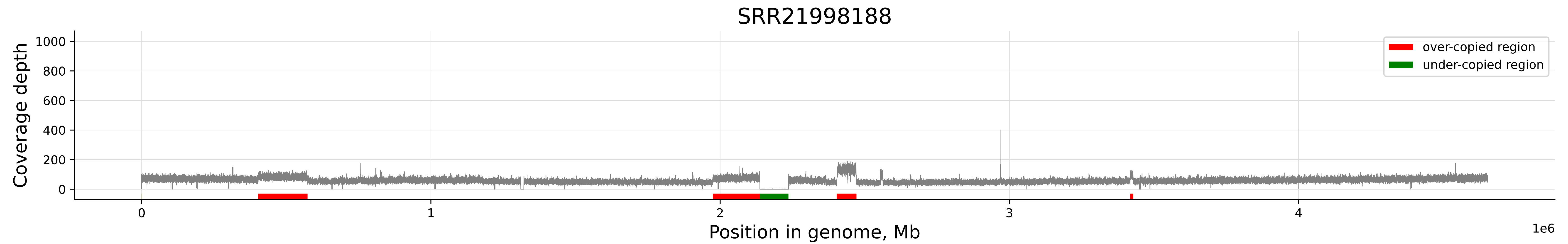
SRR21998188
Coverage depth
1000
800
600
400
200
0
0
1
2
3
4
1e6
Position in genome, Mb
over-copied region
under-copied region

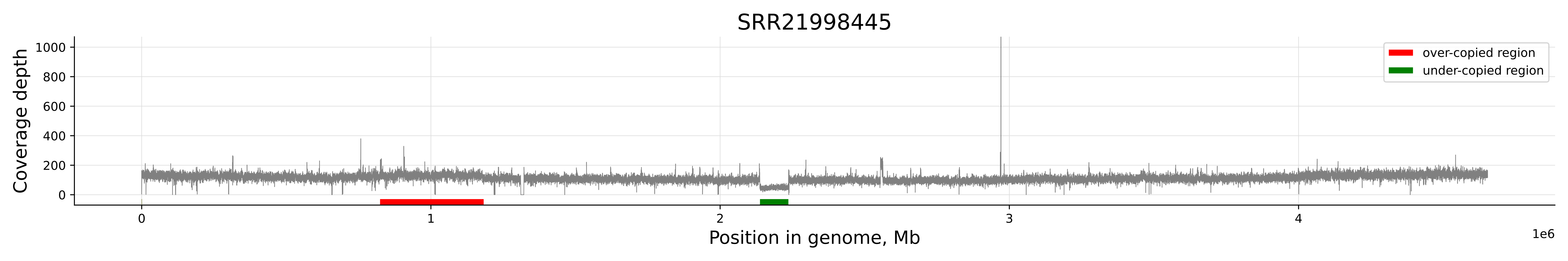
SRR21998445
Coverage depth
Position in genome, Mb
1e6
over-copied region
under-copied region
0
200
400
600
800
1000
0
1
2
3
4

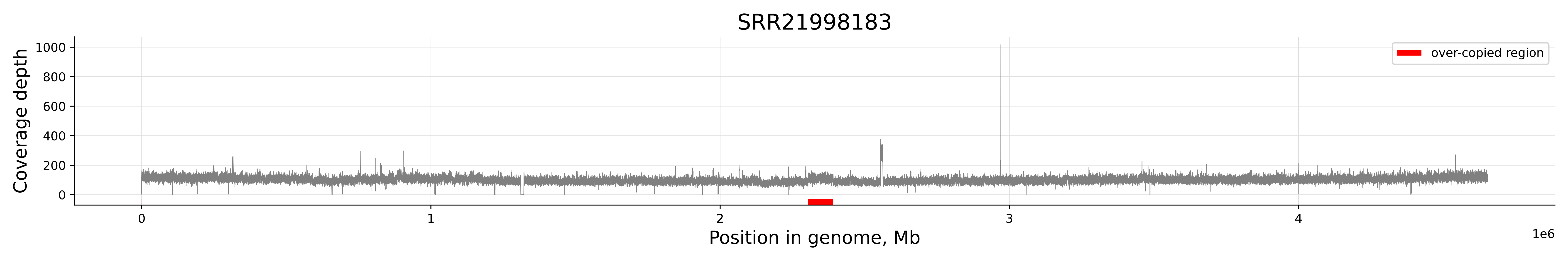

SRR21998183
Coverage depth
1000
800
600
400
200
0
over-copied region
0
1
2
3
4
1e6
Position in genome, Mb

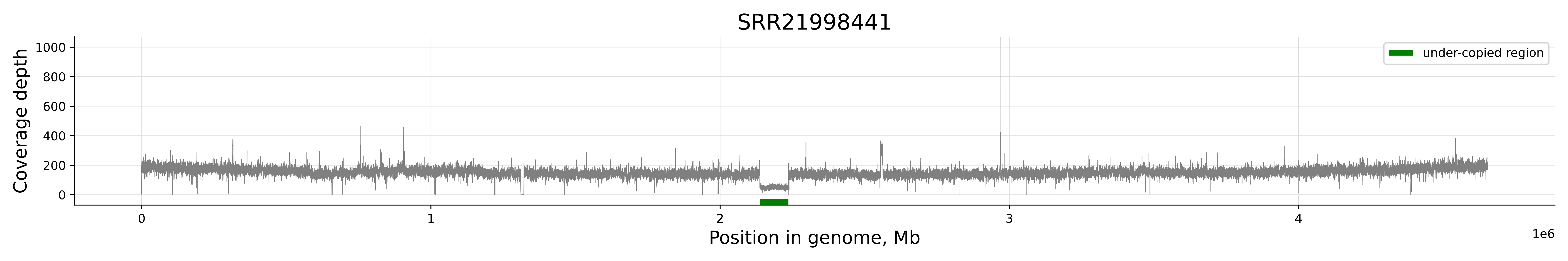
SRR21998441
Coverage depth
1000
800
600
400
200
0
0
1
2
3
4
1e6
Position in genome, Mb
under-copied region

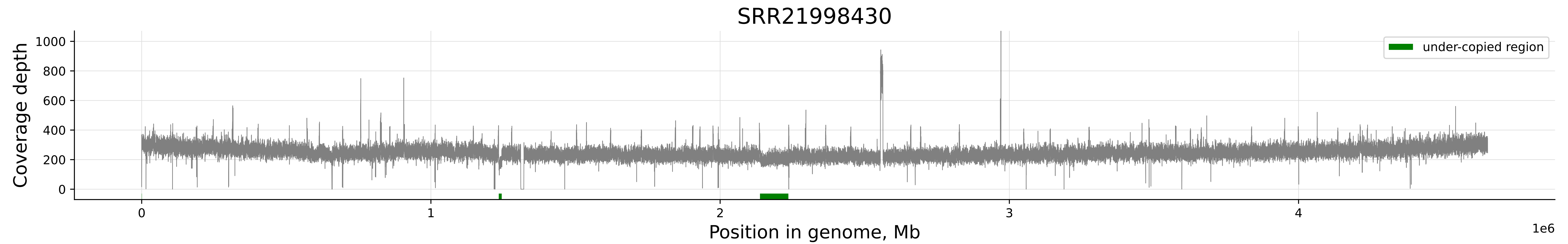
SRR21998430
Coverage depth
1000
800
600
400
200
0
under-copied region
0
1
2
3
4
1e6
Position in genome, Mb

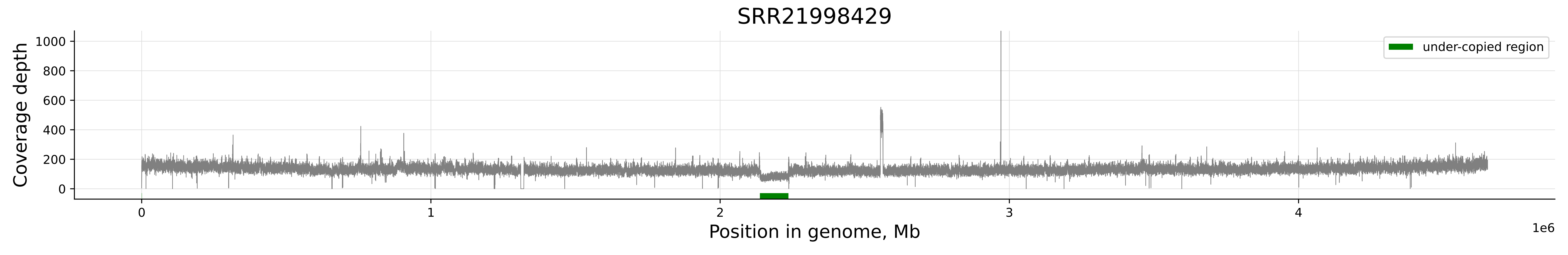
SRR21998429
Coverage depth
Position in genome, Mb
under-copied region
1e6
0
200
400
600
800
1000
0
1
2
3
4

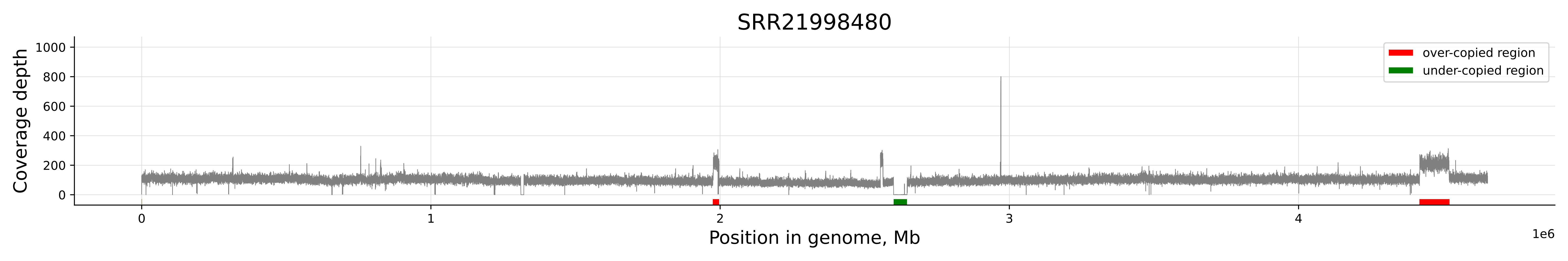

SRR21998480
Coverage depth
1000
800
600
400
200
0
0
1
2
3
4
Position in genome, Mb
1e6
over-copied region
under-copied region

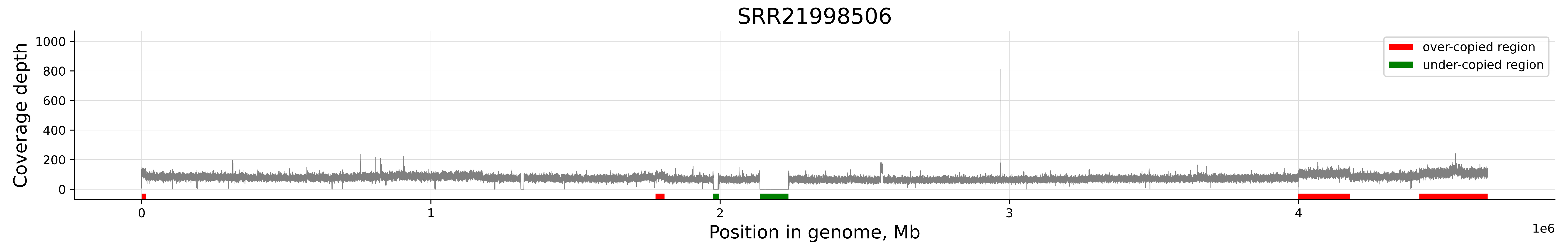

SRR21998506
Coverage depth
Position in genome, Mb
over-copied region
under-copied region
1e6

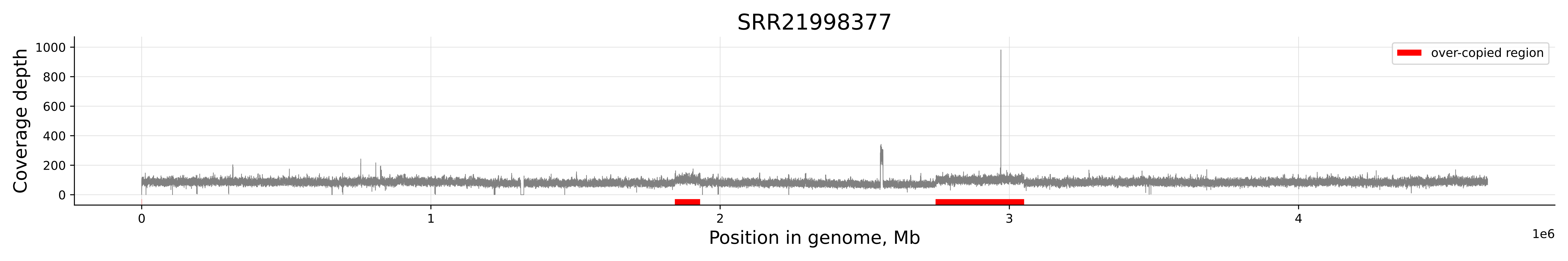
SRR21998377
Coverage depth
Position in genome, Mb
over-copied region
1e6

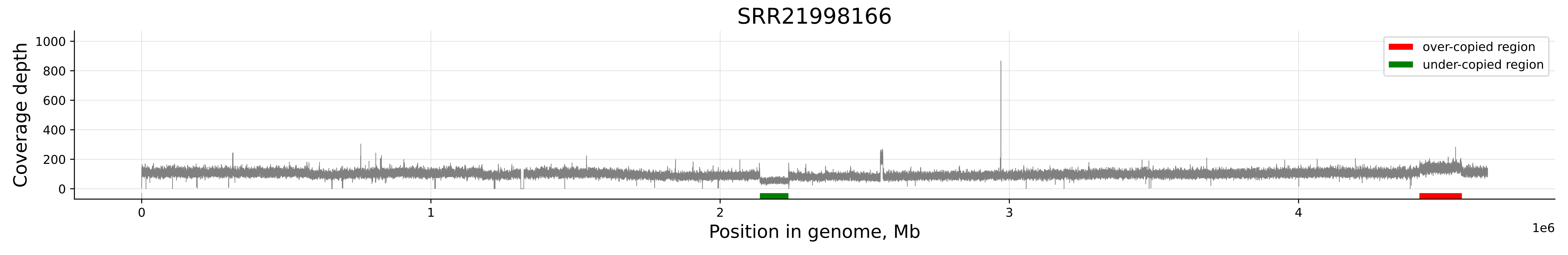
SRR21998166
Coverage depth
1000
800
600
400
200
0
0
1
2
3
4
1e6
Position in genome, Mb
over-copied region
under-copied region

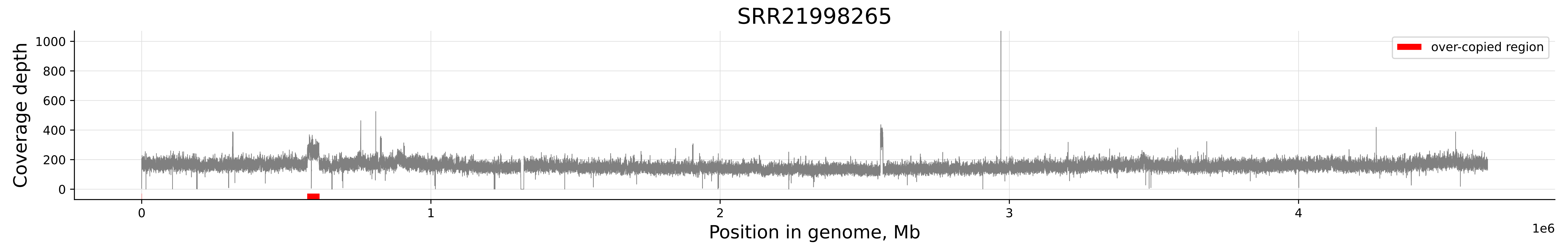
SRR21998265
Coverage depth
Position in genome, Mb
over-copied region
1e6

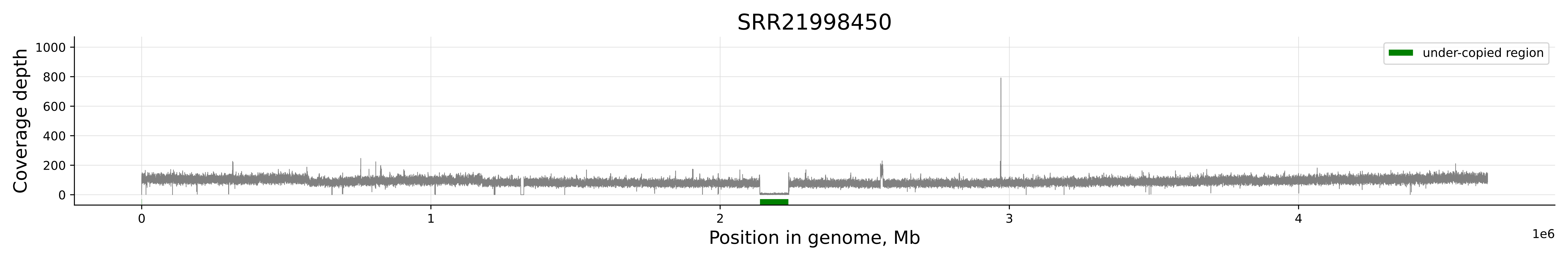

SRR21998450
Coverage depth
1000
800
600
400
200
0
under-copied region
0
1
2
3
4
1e6
Position in genome, Mb

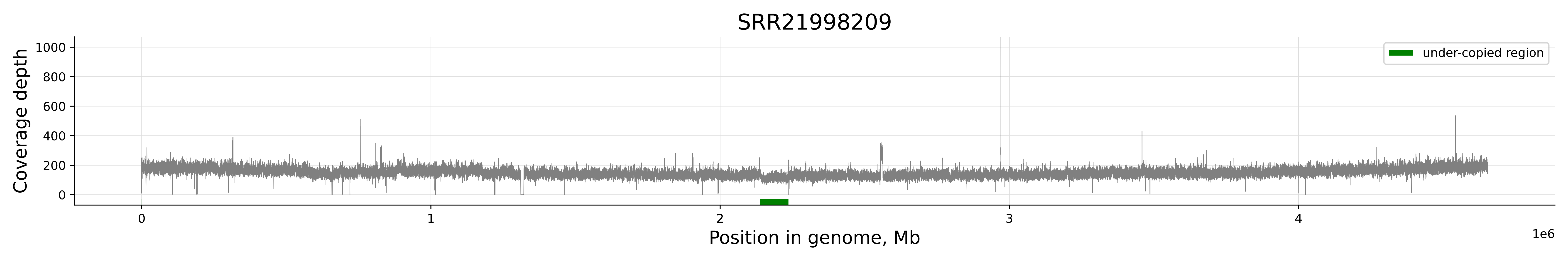
SRR21998209
under-copied region
Coverage depth
0
200
400
600
800
1000
0
1
2
3
4
1e6
Position in genome, Mb

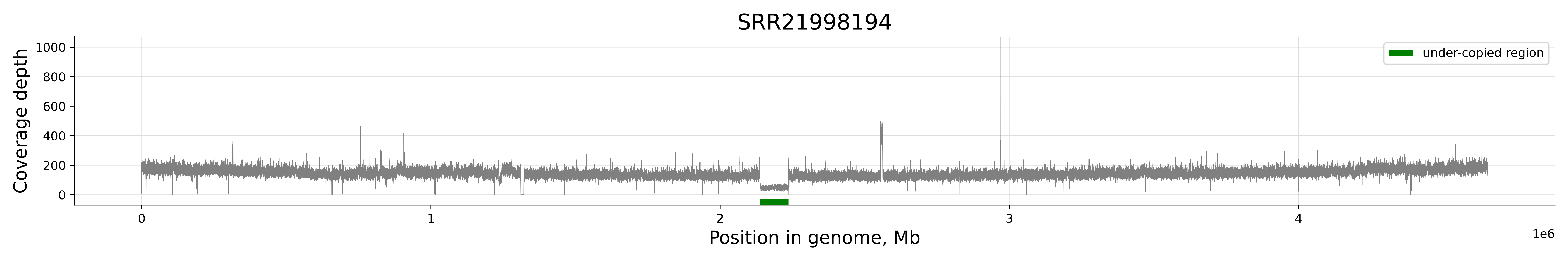

SRR21998194
under-copied region
Coverage depth
1000
800
600
400
200
0
0
1
2
3
4
1e6
Position in genome, Mb

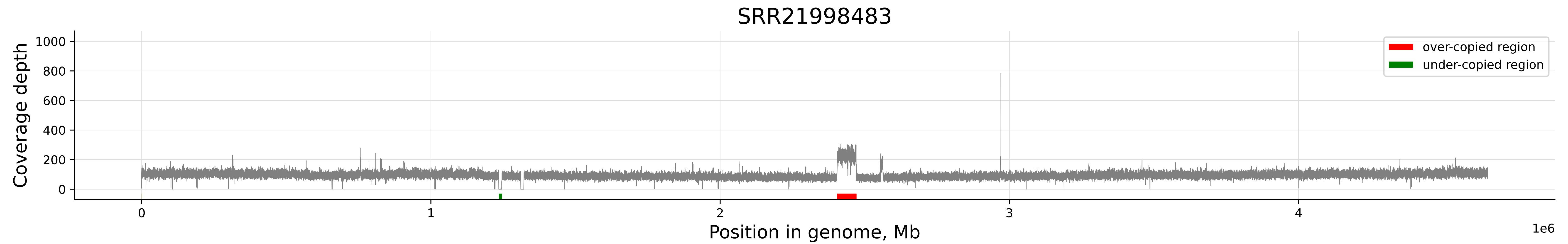
SRR21998483
Coverage depth
1000
800
600
400
200
0
over-copied region
under-copied region
0
1
2
3
4
1e6
Position in genome, Mb

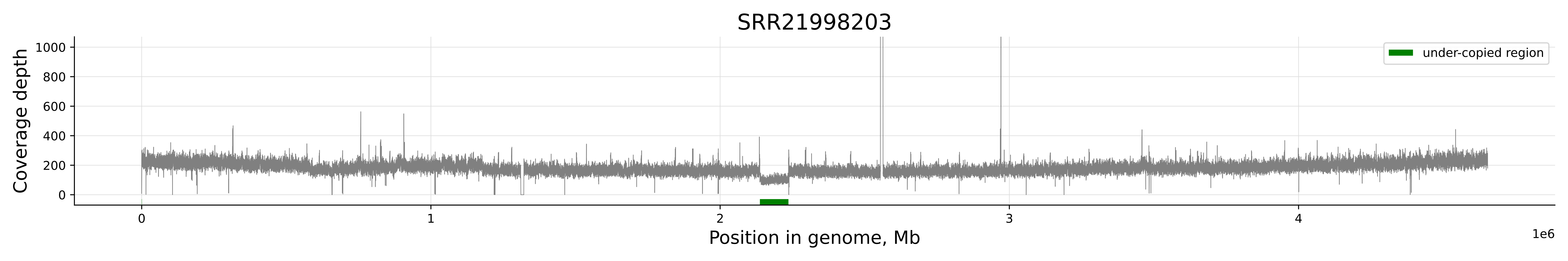

SRR21998203
under-copied region
Coverage depth
0
200
400
600
800
1000
Position in genome, Mb
0
1
2
3
4
1e6

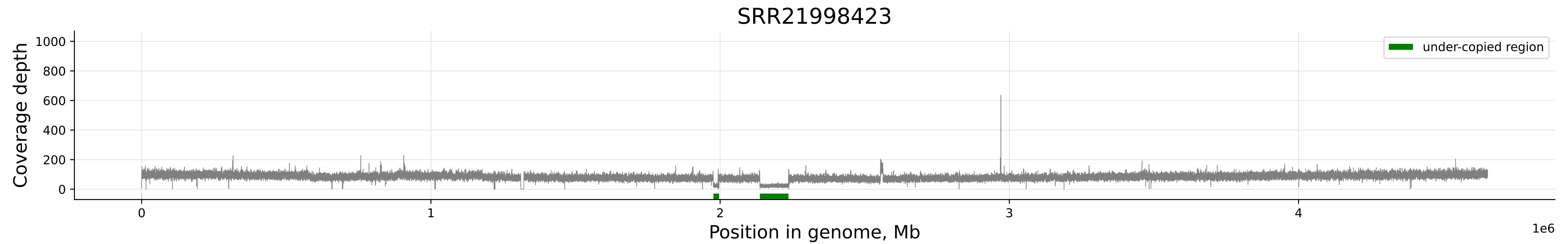
SRR21998423
Coverage depth
Position in genome, Mb
under-copied region
1000
800
600
400
200
0
0
1
2
3
4
1e6

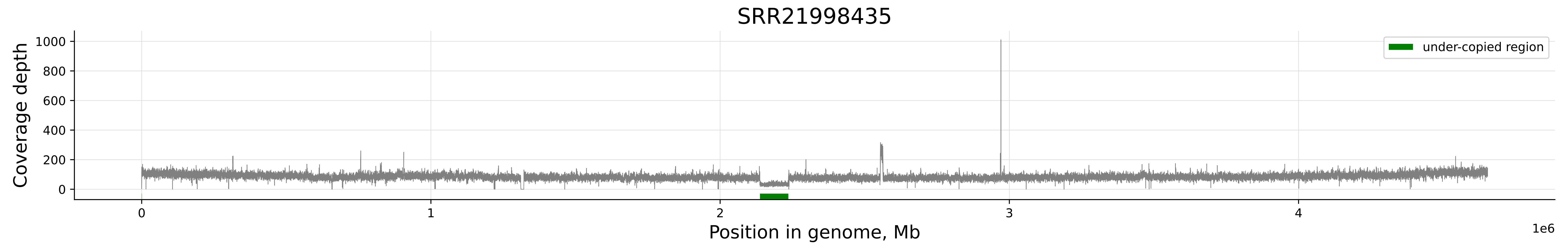

SRR21998435
Coverage depth
1000
800
600
400
200
0
under-copied region
0
1
2
3
4
1e6
Position in genome, Mb

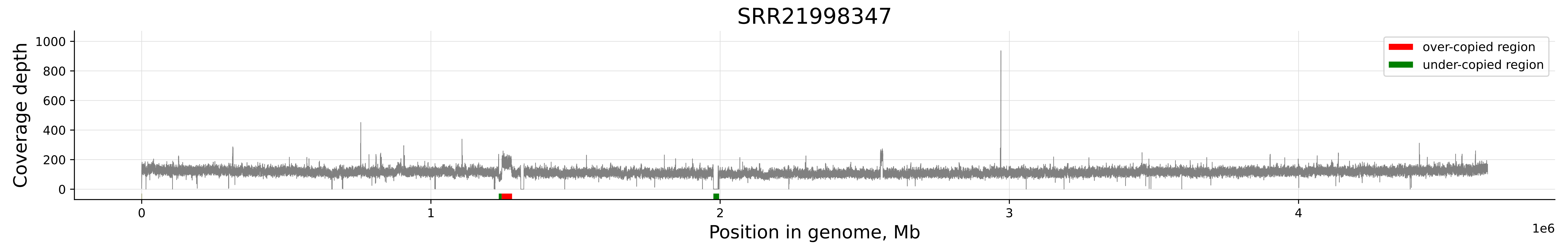
SRR21998347
Coverage depth
1000
800
600
400
200
0
over-copied region
under-copied region
0
1
2
3
4
1e6
Position in genome, Mb

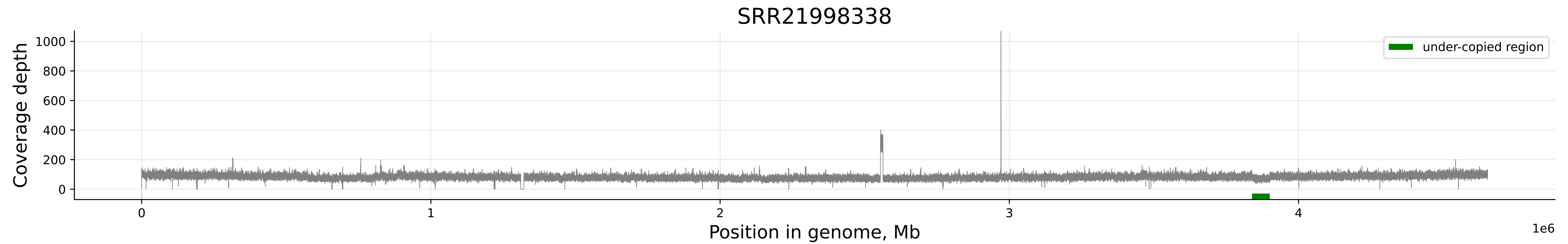
SRR21998338
Coverage depth
Position in genome, Mb
under-copied region
0
200
400
600
800
1000
1
2
3
4
1e6

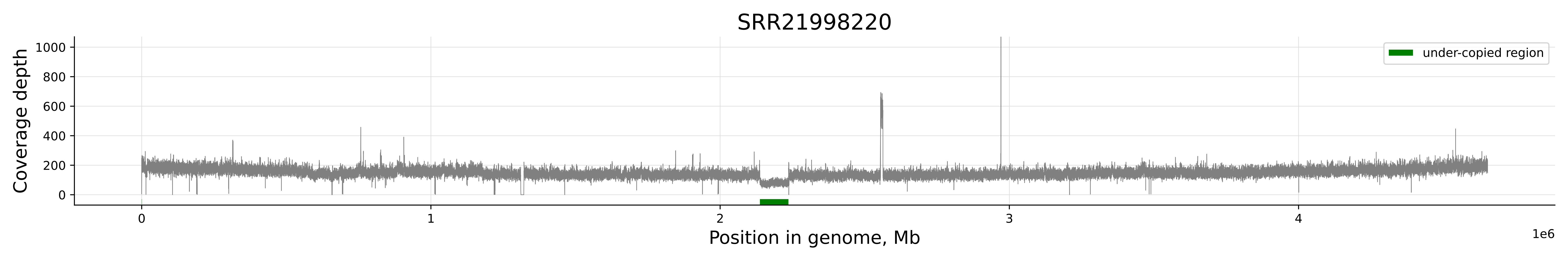

SRR21998220
Coverage depth
0
200
400
600
800
1000
under-copied region
0
1
2
3
4
1e6
Position in genome, Mb

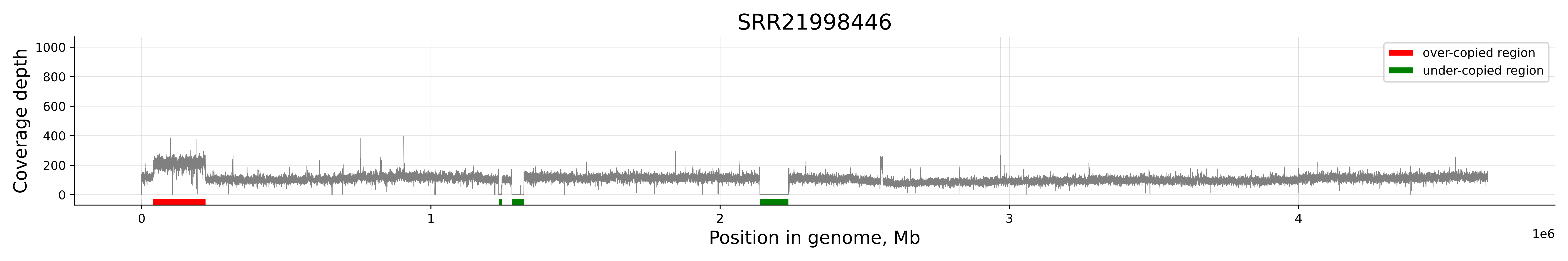
SRR21998446
Coverage depth
0
200
400
600
800
1000
over-copied region
under-copied region
0
1
2
3
4
1e6
Position in genome, Mb

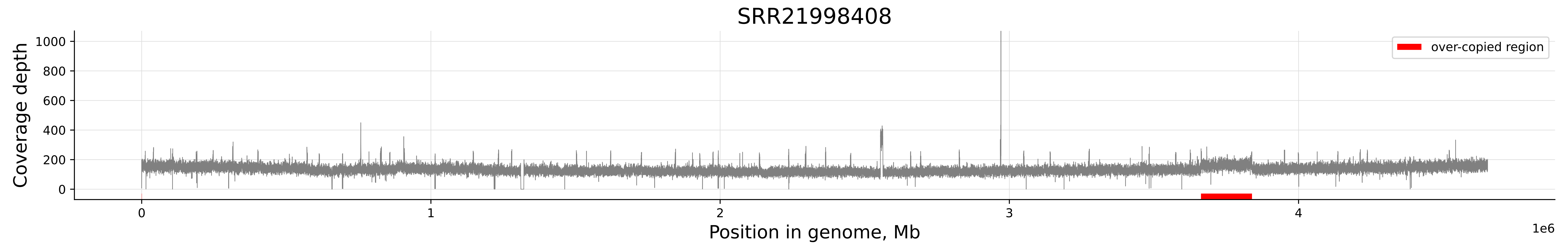
SRR21998408
Coverage depth
Position in genome, Mb
1e6
over-copied region
0
200
400
600
800
1000
0
1
2
3
4

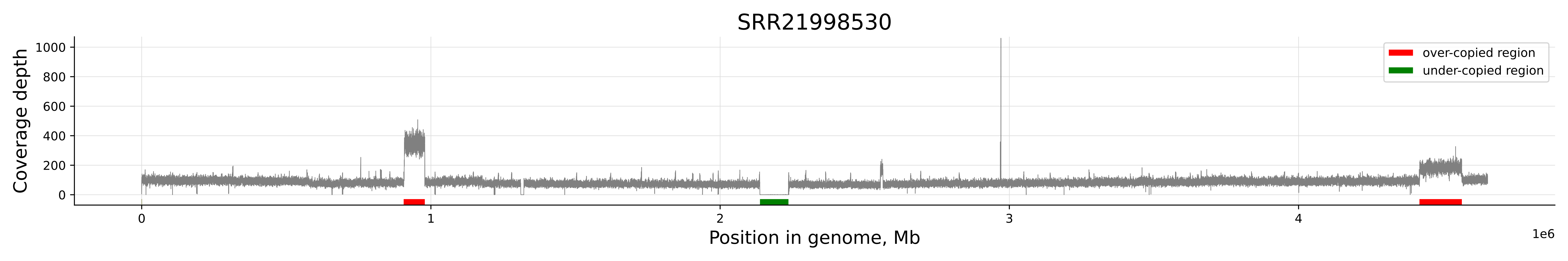
SRR21998530
Coverage depth
1000
800
600
400
200
0
0
1
2
3
4
1e6
Position in genome, Mb
over-copied region
under-copied region

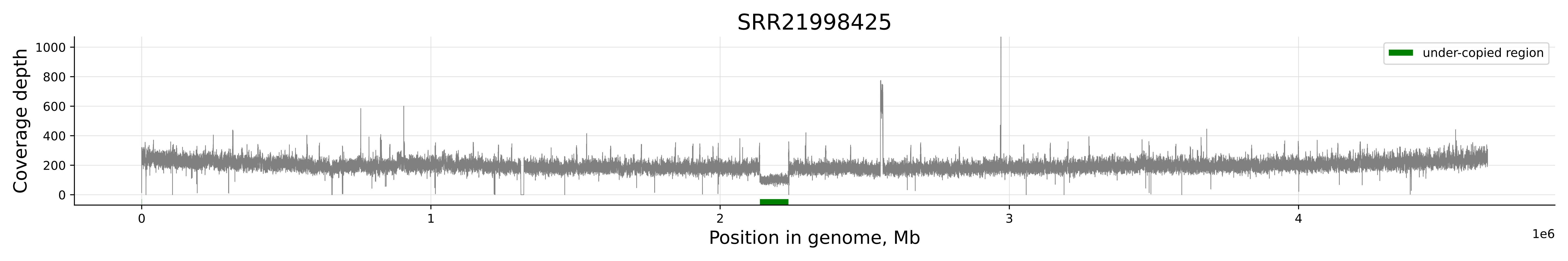
SRR21998425
Coverage depth
Position in genome, Mb
1e6
under-copied region
0
200
400
600
800
1000
0
1
2
3
4

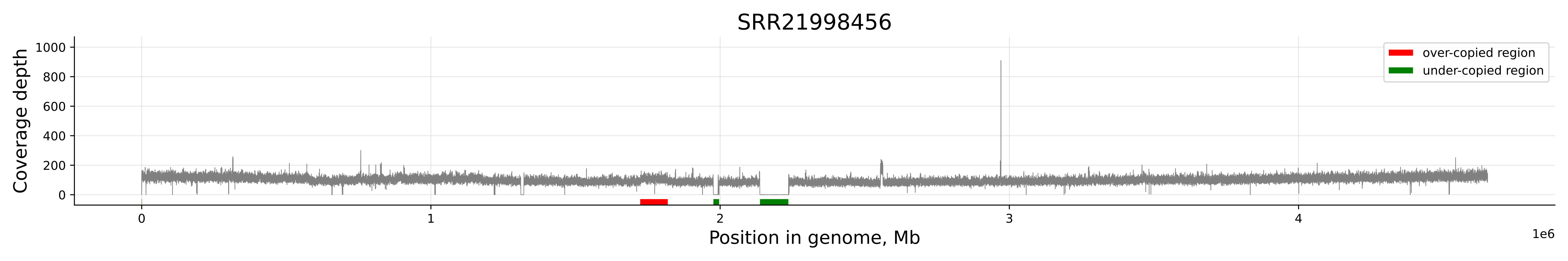
SRR21998456
Coverage depth
Position in genome, Mb
over-copied region
under-copied region
1000
800
600
400
200
0
0
1
2
3
4
1e6
