# Supplementary figures and images for: Systematic analysis of spontaneous tandem genome amplification events in Yersinia pestis
Source: PLoS One. 2025 Dec 31;20(12):e0338460. doi: 10.1371/journal.pone.0338460 (PMC12755819; doi:10.1371/journal.pone.0338460)

The image segment  
used in Figure 1C

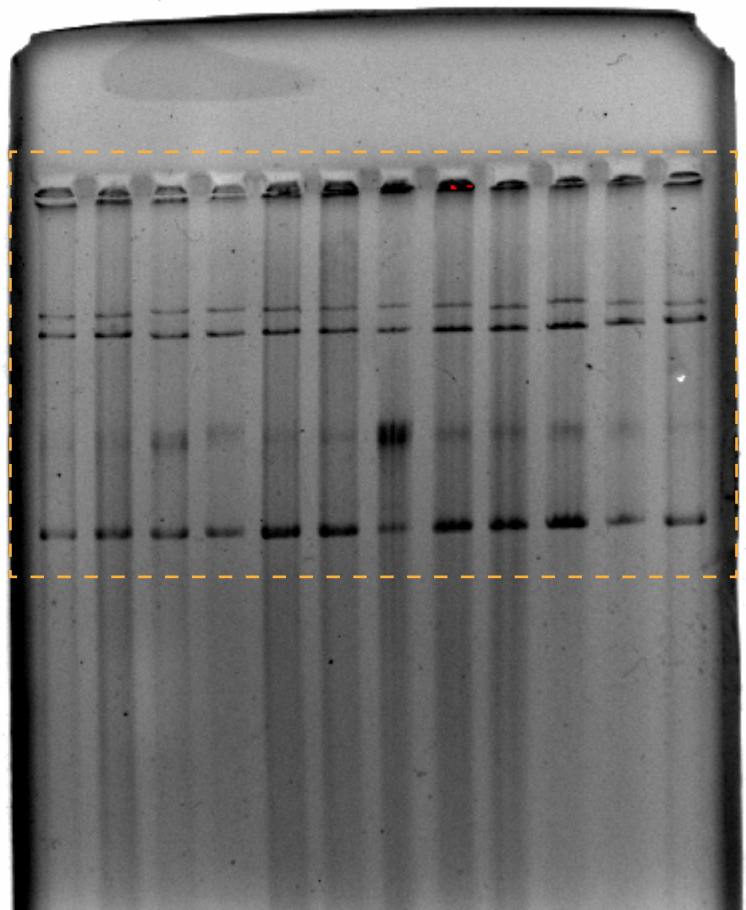

S1\_raw\_images.pdf

Supplement: S1 raw images — (PDF) [file pone.0338460.s006.pdf]
