# Supplementary material for: Systematic analysis of spontaneous tandem genome amplification events in Yersinia pestis
Source: PLoS One. 2025 Dec 31;20(12):e0338460. doi: 10.1371/journal.pone.0338460 (PMC12755819; doi:10.1371/journal.pone.0338460)
Supplement: S1 Data. — (PDF) [file pone.0338460.s002.pdf]

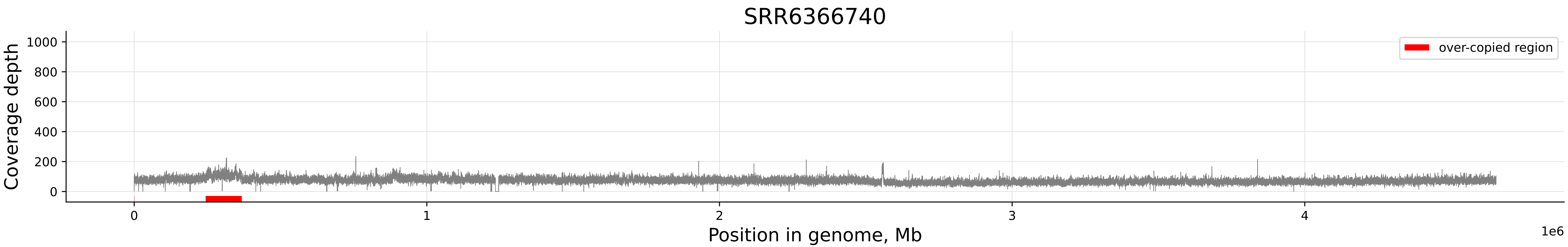

SRR6366740
over-copied region
Coverage depth
1000
800
600
400
200
0
0
1
2
3
4
1e6
Position in genome, Mb

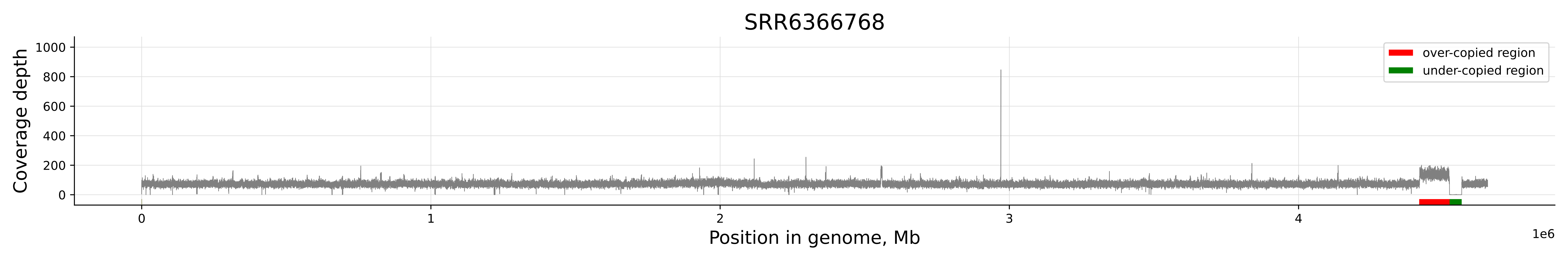

SRR6366768
Coverage depth
1000
800
600
400
200
0
0
1
2
3
4
1e6
Position in genome, Mb
over-copied region
under-copied region

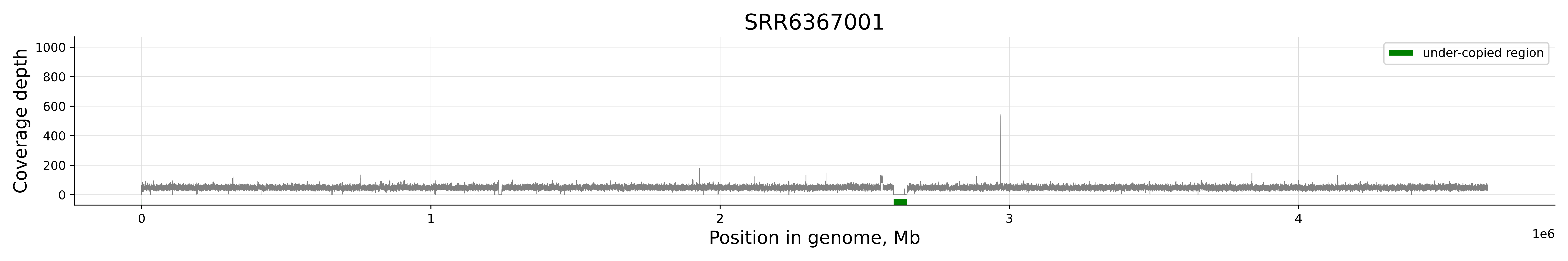

SRR6367001
Coverage depth
1000
800
600
400
200
0
under-copied region
0
1
2
3
4
1e6
Position in genome, Mb

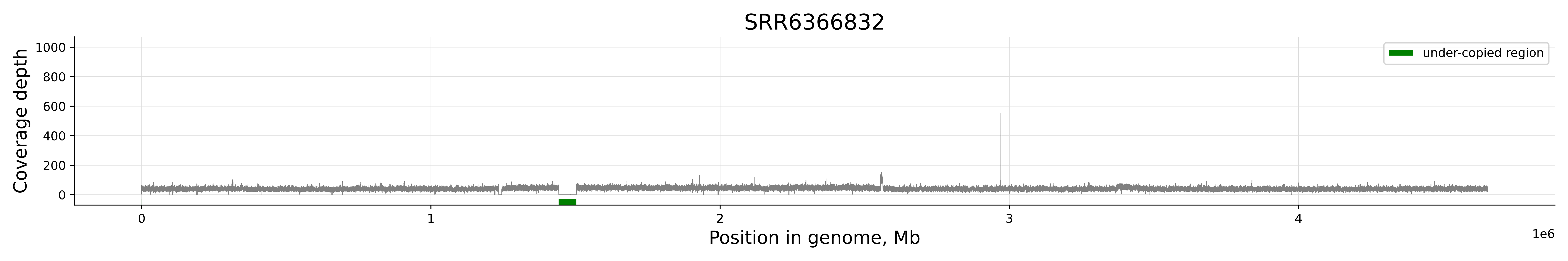
SRR6366832
Coverage depth
Position in genome, Mb
1e6
under-copied region
0
200
400
600
800
1000
0
1
2
3
4

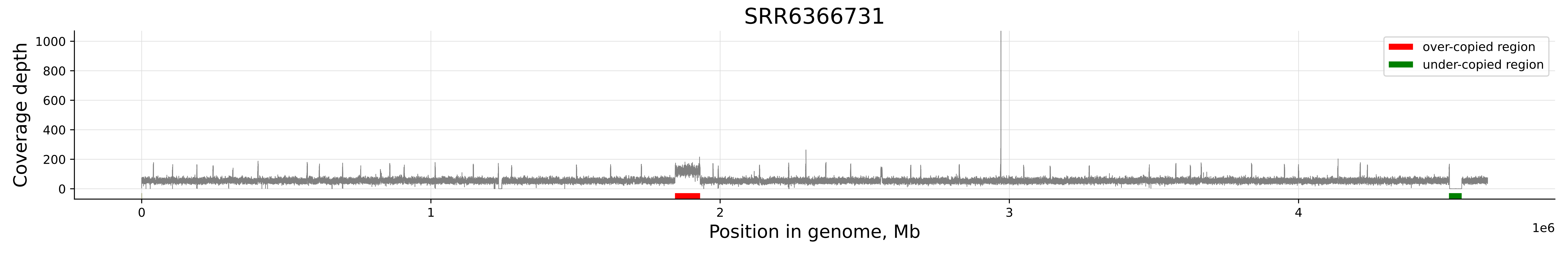

SRR6366731
Coverage depth
Position in genome, Mb
1e6
over-copied region
under-copied region
0
200
400
600
800
1000
0
1
2
3
4

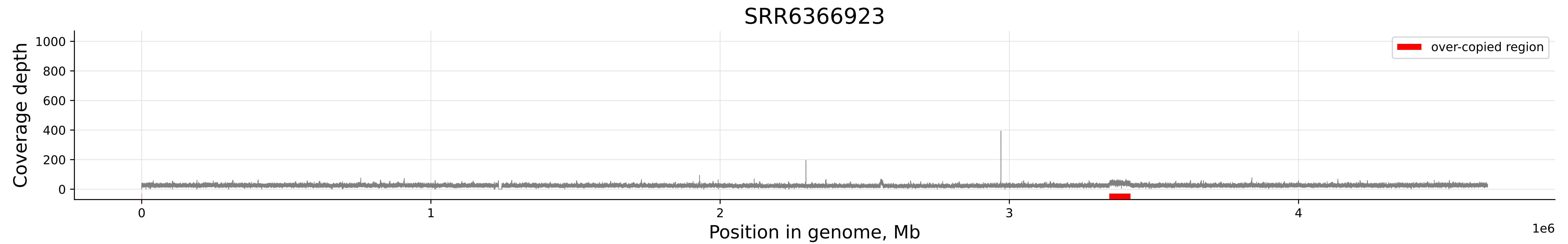
SRR6366923
Coverage depth
Position in genome, Mb
over-copied region
1000
800
600
400
200
0
1
2
3
4
1e6

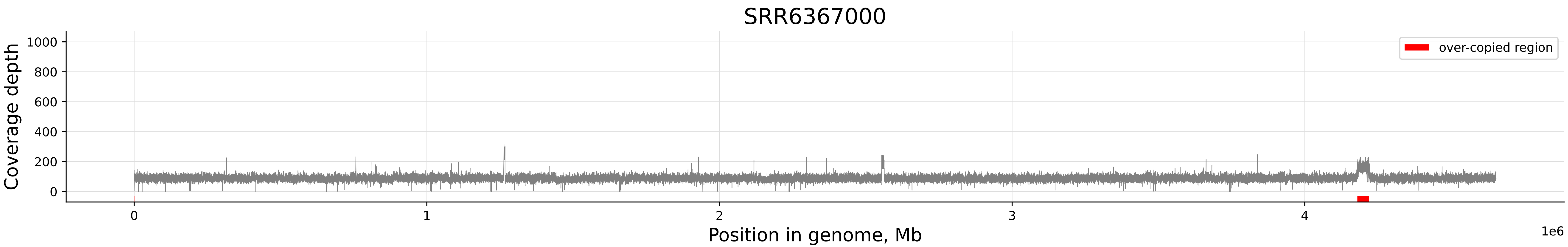

SRR6367000
Coverage depth
1000
800
600
400
200
0
over-copied region
0
1
2
3
4
1e6
Position in genome, Mb

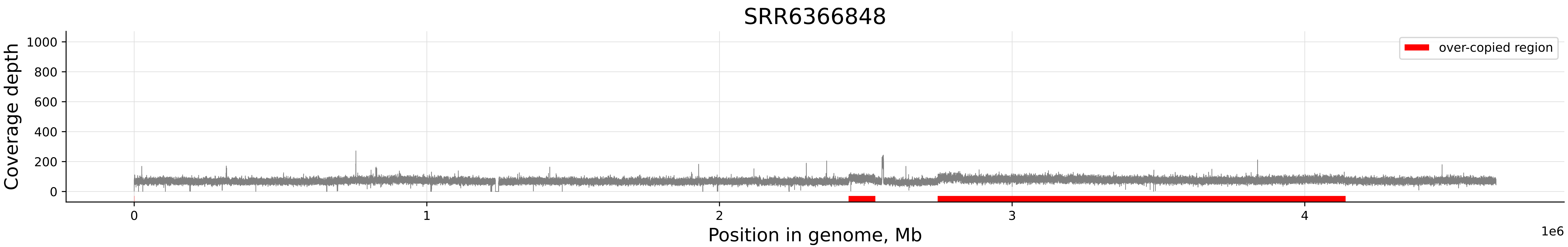

SRR6366848
Coverage depth
1000
800
600
400
200
0
over-copied region
0
1
2
3
4
1e6
Position in genome, Mb

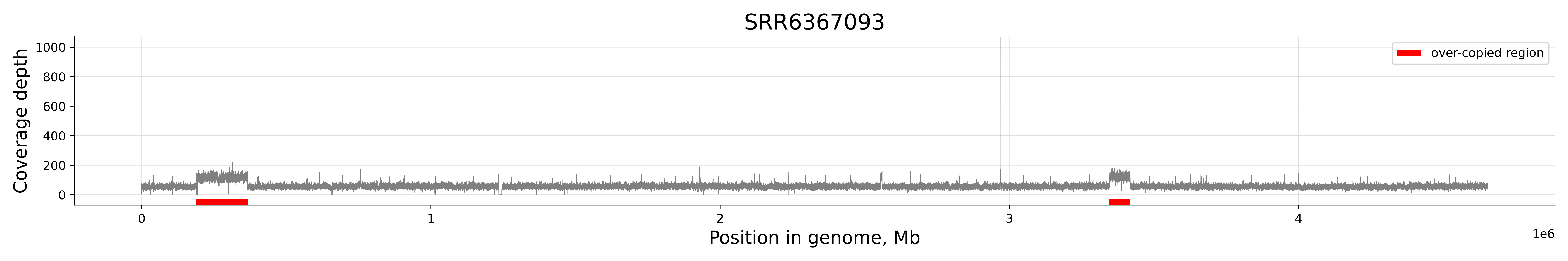

SRR6367093
Coverage depth
1000
800
600
400
200
0
over-copied region
0
1
2
3
4
1e6
Position in genome, Mb

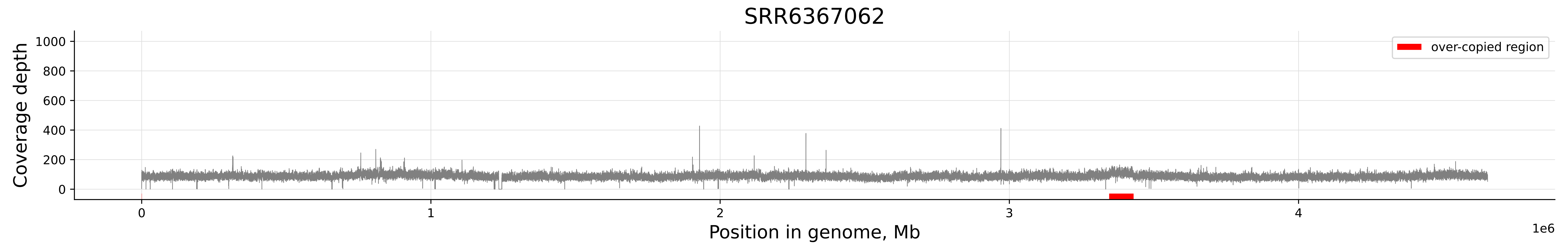
SRR6367062
Coverage depth
1000
800
600
400
200
0
over-copied region
0
1
2
3
4
1e6
Position in genome, Mb

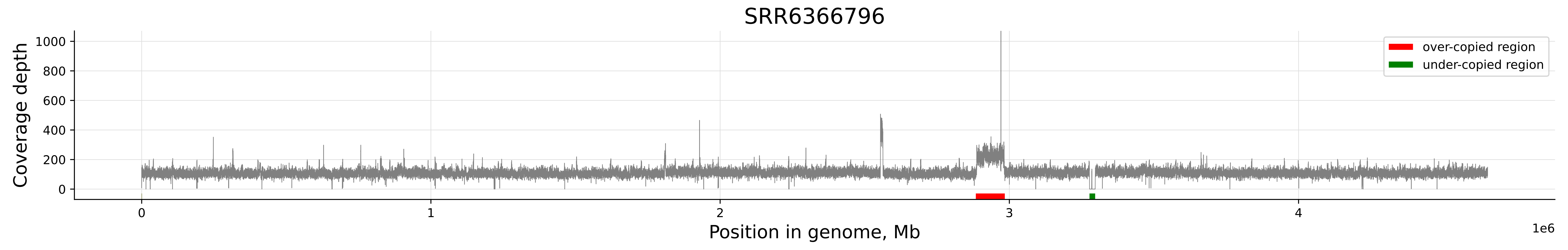
SRR6366796
Coverage depth
0
200
400
600
800
1000
over-copied region
under-copied region
0
1
2
3
4
1e6
Position in genome, Mb

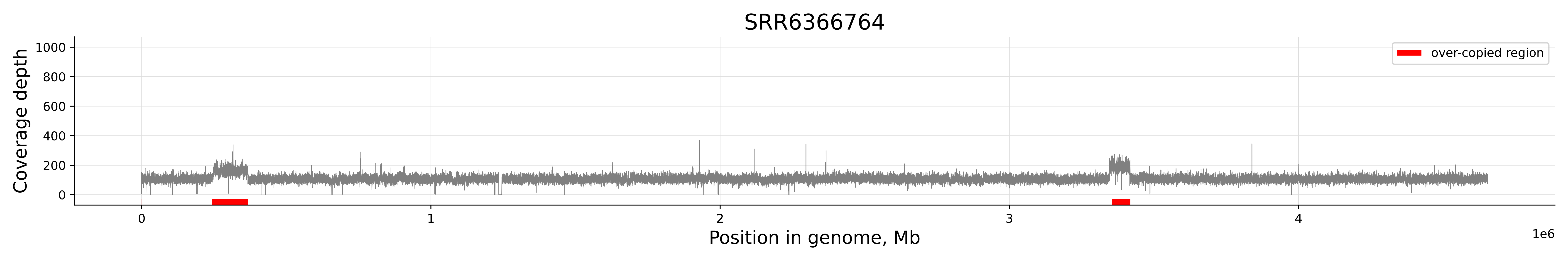

SRR6366764
Coverage depth
Position in genome, Mb
over-copied region
1000
800
600
400
200
0
0
1
2
3
4
1e6

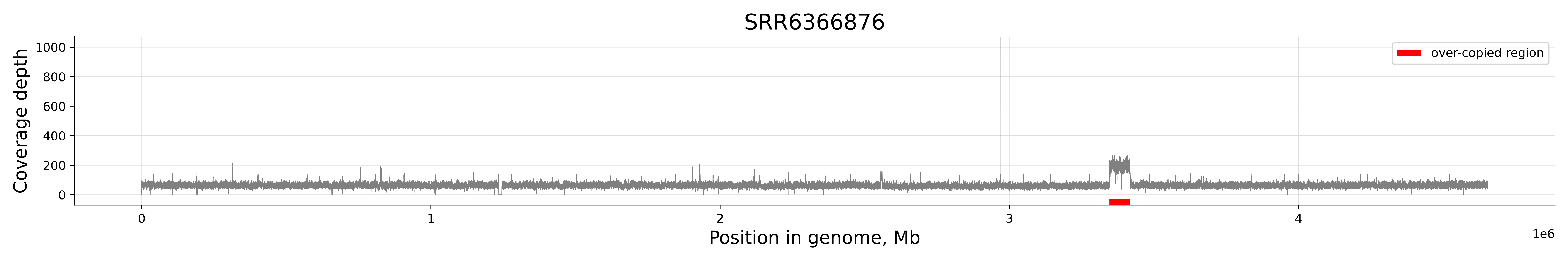

SRR6366876
over-copied region
Coverage depth
0
200
400
600
800
1000
0
1
2
3
4
1e6
Position in genome, Mb

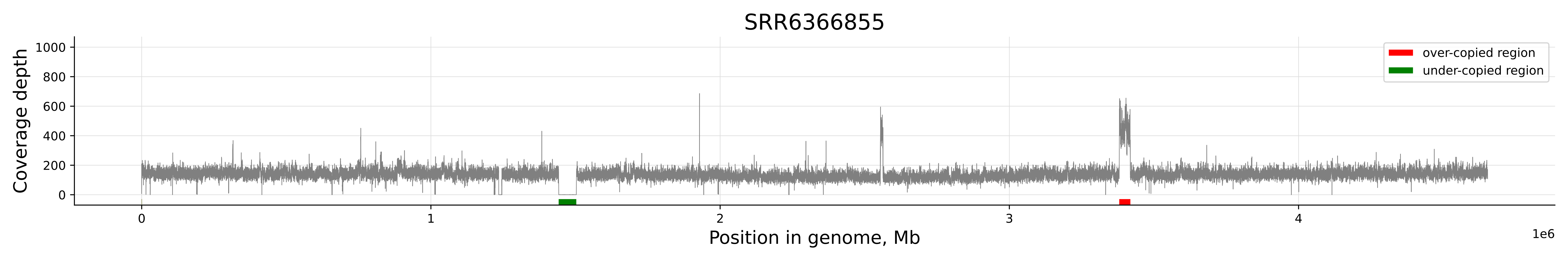
SRR6366855
Coverage depth
1000
800
600
400
200
0
over-copied region
under-copied region
0
1
2
3
4
1e6
Position in genome, Mb

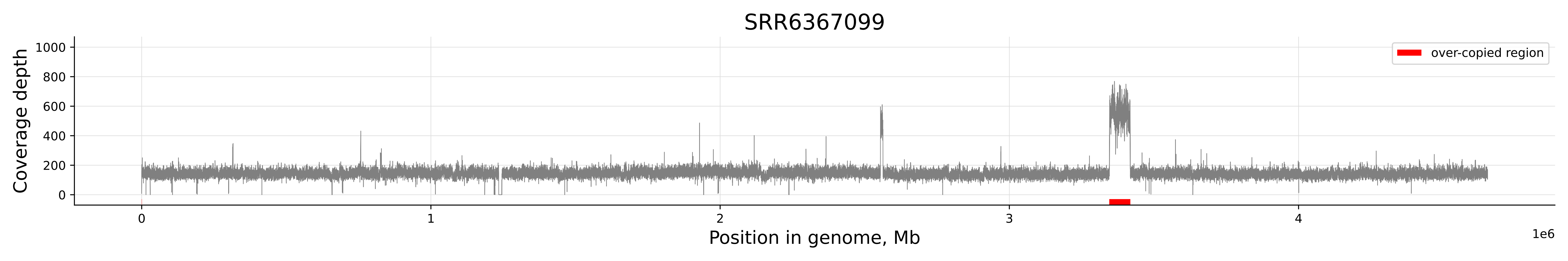
SRR6367099
Coverage depth
Position in genome, Mb
over-copied region
1000
800
600
400
200
0
0
1
2
3
4
1e6

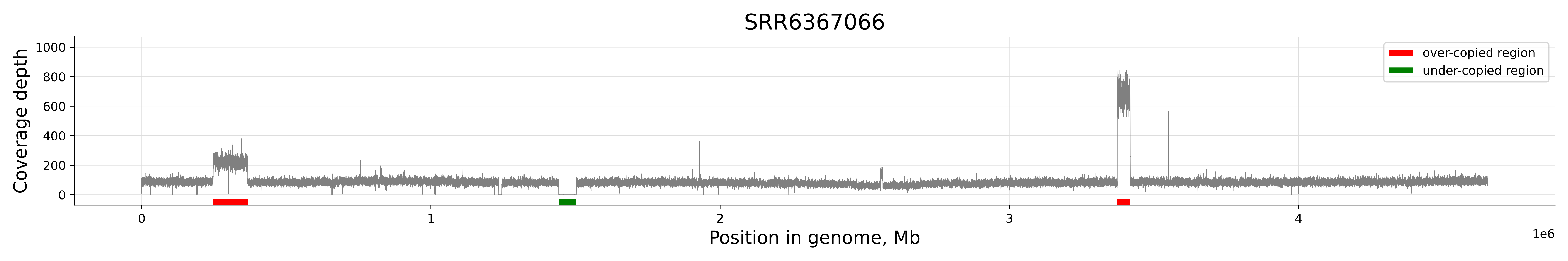

SRR6367066
Coverage depth
Position in genome, Mb
1e6
over-copied region
under-copied region
0
200
400
600
800
1000
0
1
2
3
4

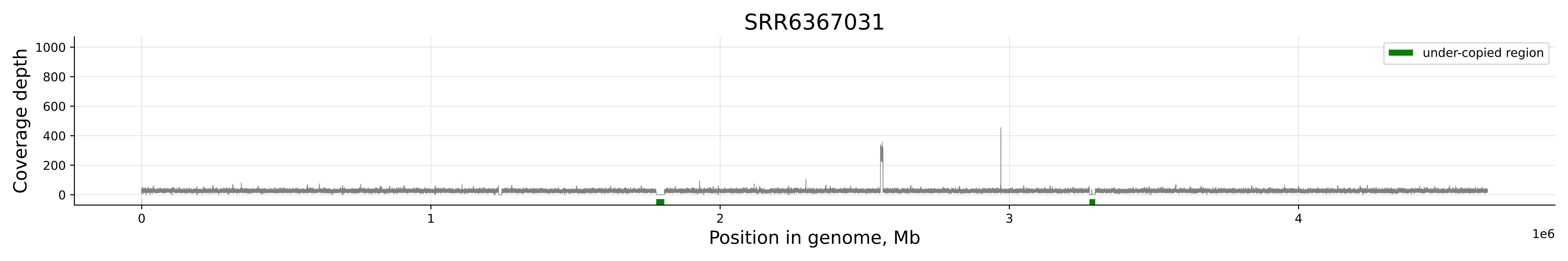
SRR6367031
Coverage depth
1000
800
600
400
200
0
0
1
2
3
4
1e6
Position in genome, Mb
under-copied region

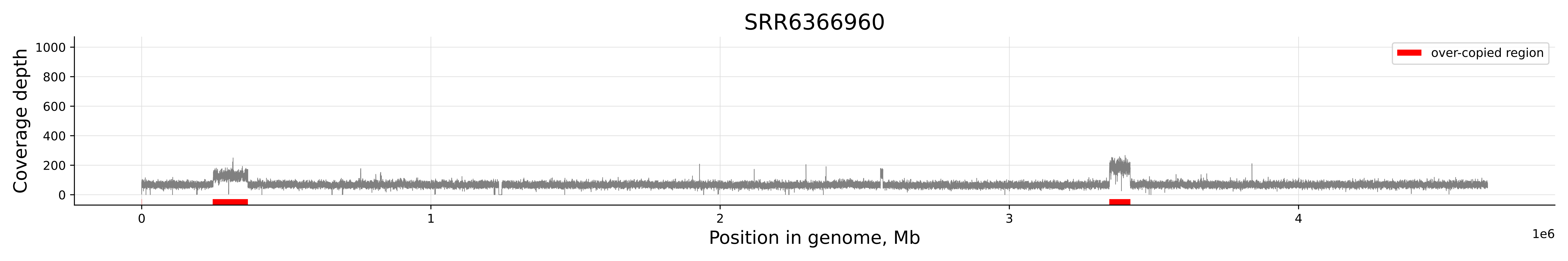

SRR6366960
Coverage depth
1000
800
600
400
200
0
over-copied region
0
1
2
3
4
1e6
Position in genome, Mb

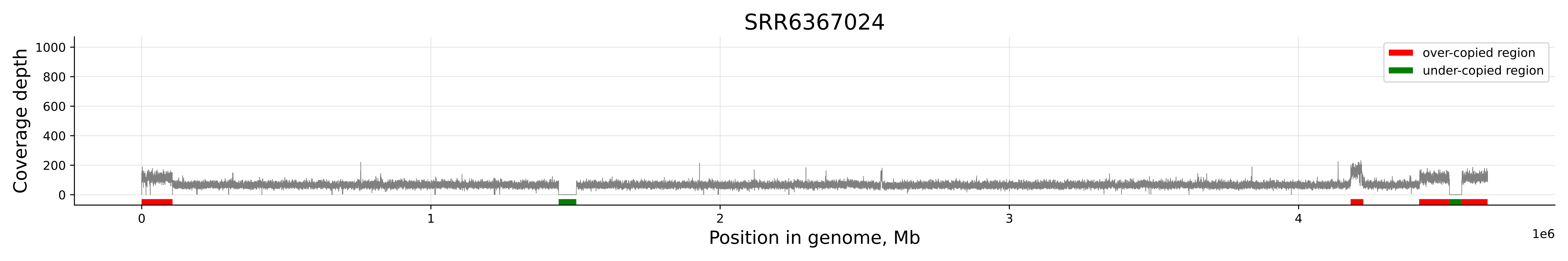

SRR6367024
Coverage depth
1000
800
600
400
200
0
0
1
2
3
4
1e6
Position in genome, Mb
over-copied region
under-copied region

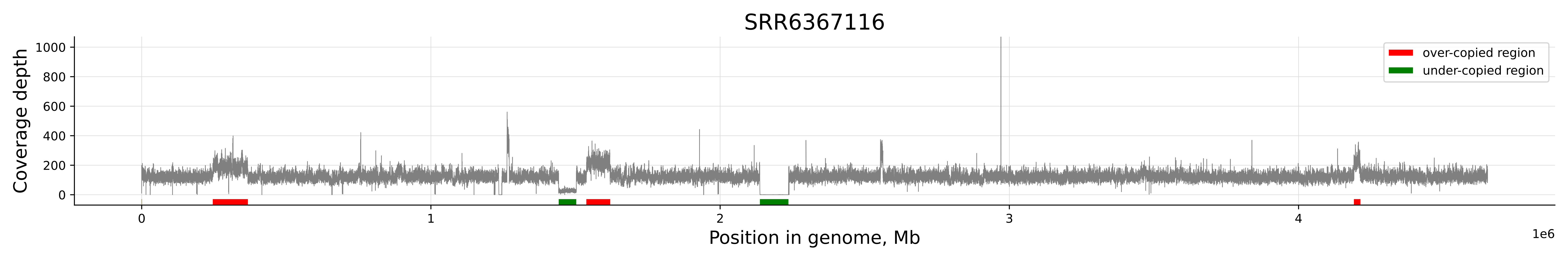

SRR6367116
over-copied region
under-copied region
Coverage depth
0
200
400
600
800
1000
0
1
2
3
4
1e6
Position in genome, Mb

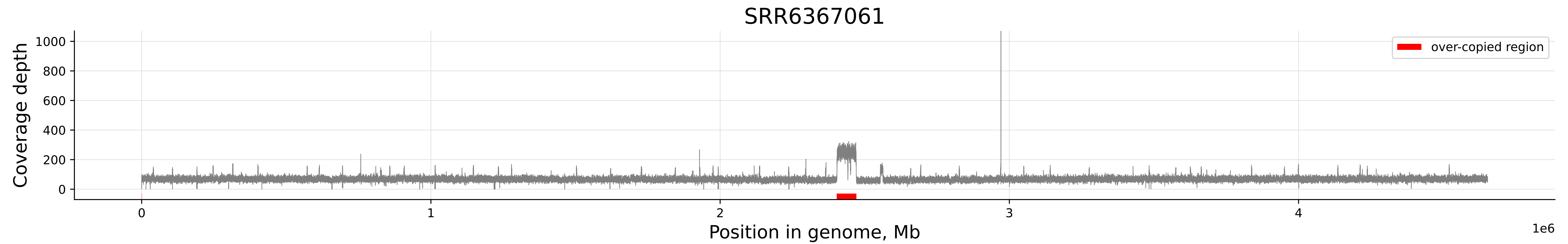
SRR6367061
Coverage depth
1000
800
600
400
200
0
over-copied region
0
1
2
3
4
1e6
Position in genome, Mb

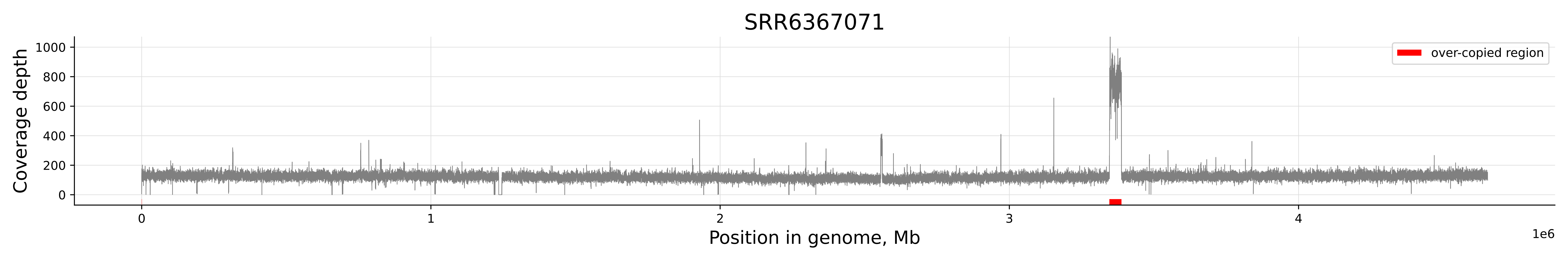
SRR6367071
Coverage depth
Position in genome, Mb
over-copied region
1e6
0
1
2
3
4
0
200
400
600
800
1000

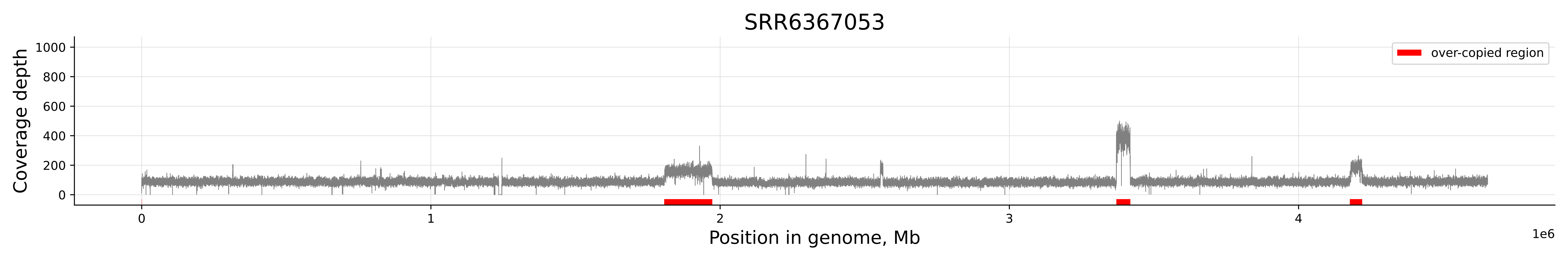
SRR6367053
Coverage depth
1000
800
600
400
200
0
0
1
2
3
4
1e6
Position in genome, Mb
over-copied region

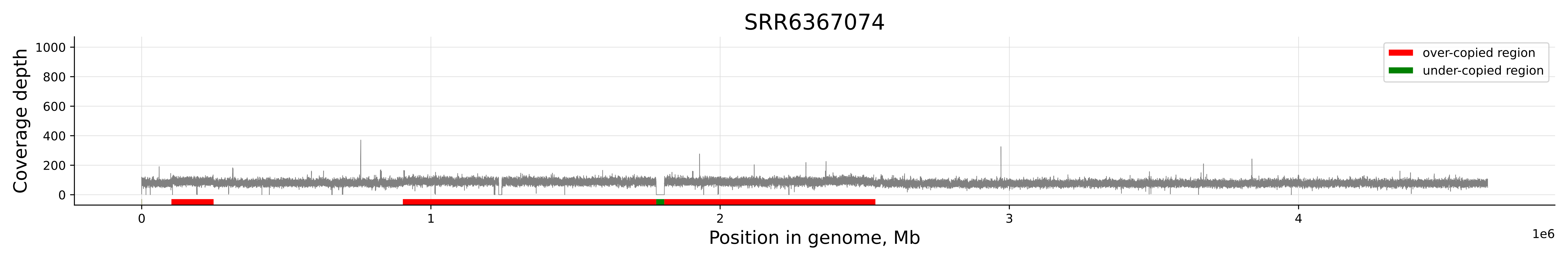

SRR6367074
Coverage depth
1000
800
600
400
200
0
over-copied region
under-copied region
0
1
2
3
4
1e6
Position in genome, Mb

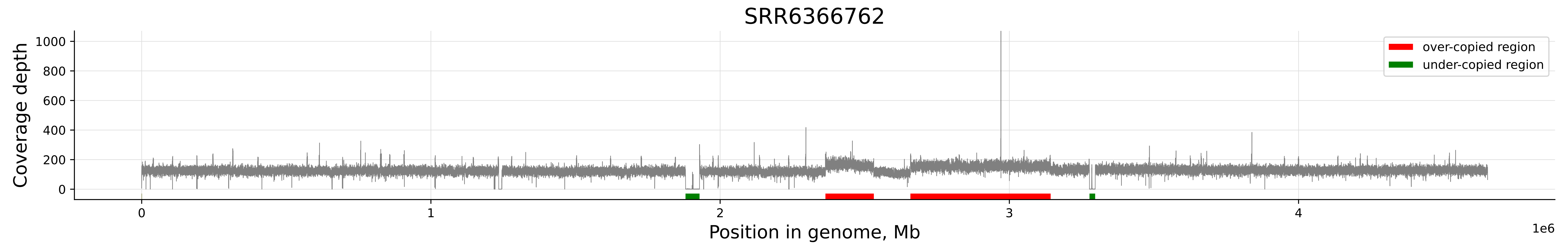

SRR6366762
Coverage depth
0
200
400
600
800
1000
over-copied region
under-copied region
0
1
2
3
4
Position in genome, Mb
1e6

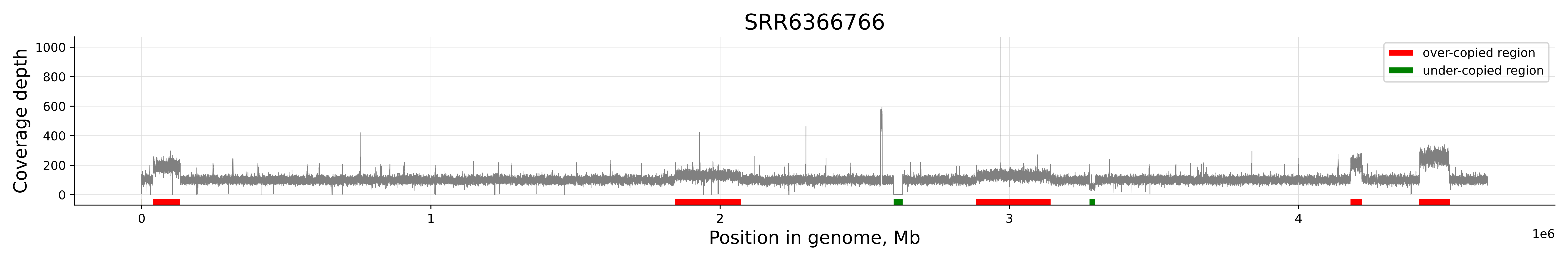
SRR6366766
Coverage depth
0
200
400
600
800
1000
over-copied region
under-copied region
0
1
2
3
4
1e6
Position in genome, Mb

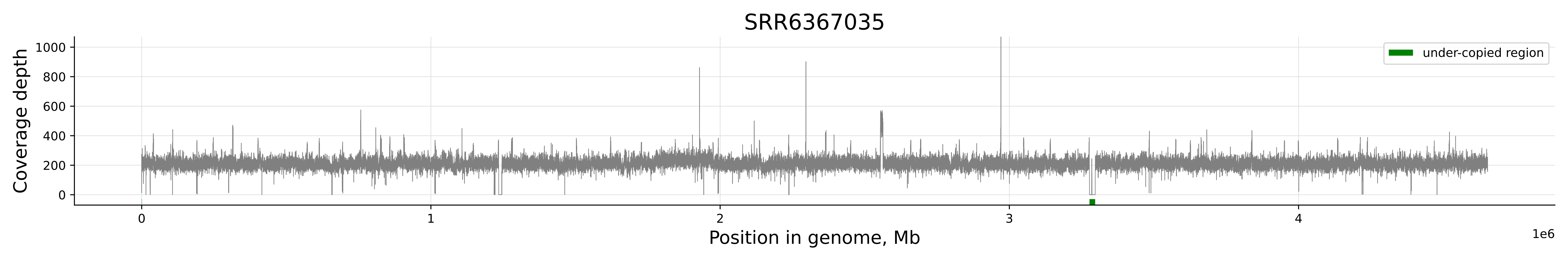
SRR6367035
Coverage depth
Position in genome, Mb
under-copied region
1e6

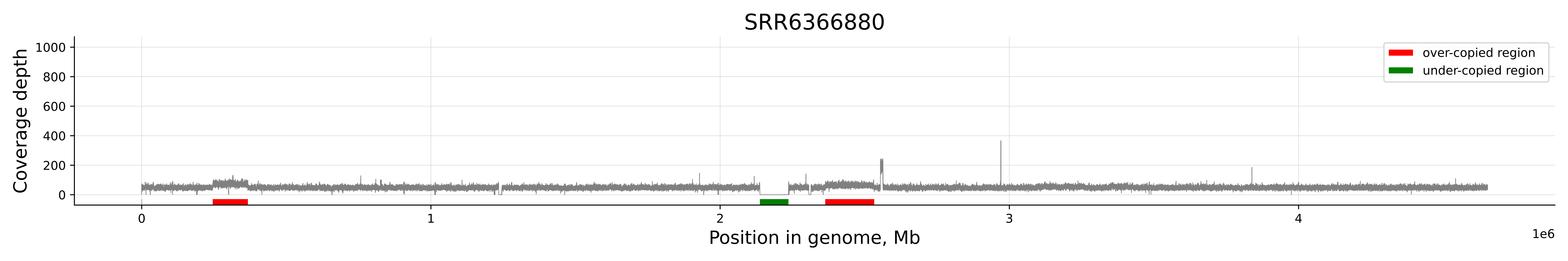
SRR6366880
Coverage depth
Position in genome, Mb
over-copied region
under-copied region
1000
800
600
400
200
0
0
1
2
3
4
1e6

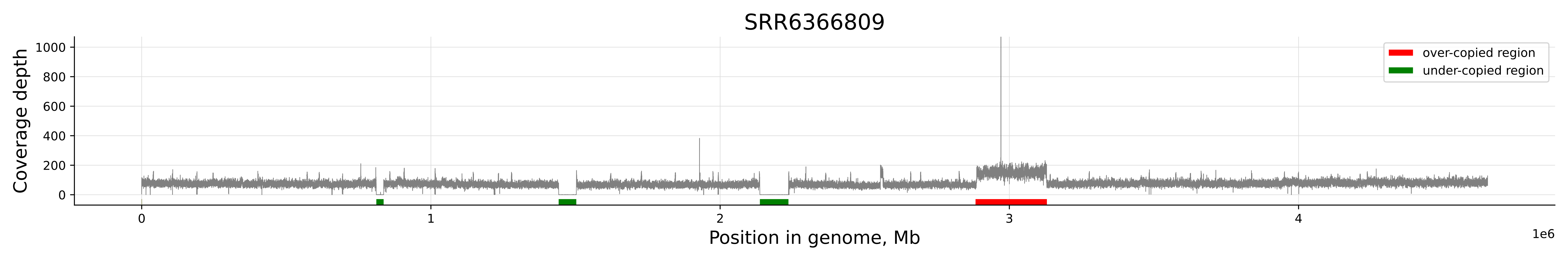
SRR6366809
Coverage depth
0
200
400
600
800
1000
0
1
2
3
4
1e6
Position in genome, Mb
over-copied region
under-copied region

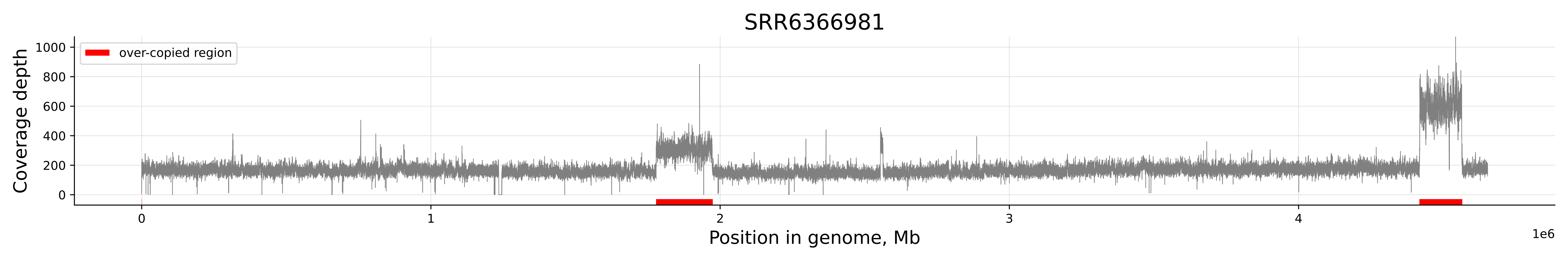

SRR6366981
over-copied region
Coverage depth
0
200
400
600
800
1000
0
1
2
3
4
1e6
Position in genome, Mb

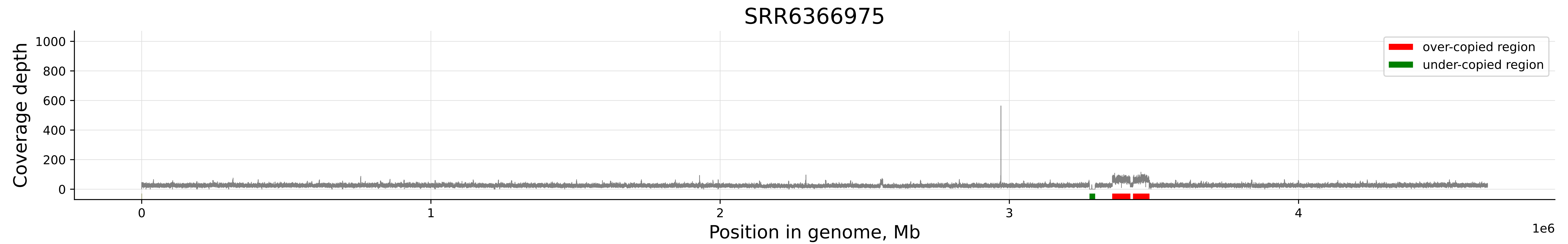
SRR6366975
over-copied region
under-copied region
Coverage depth
1000
800
600
400
200
0
0
1
2
3
4
1e6
Position in genome, Mb

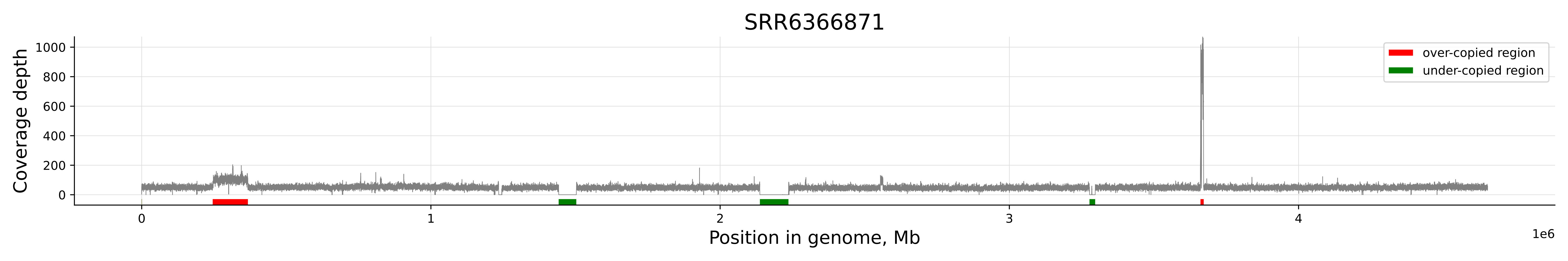
SRR6366871
Coverage depth
1000
800
600
400
200
0
over-copied region
under-copied region
0
1
2
3
4
1e6
Position in genome, Mb

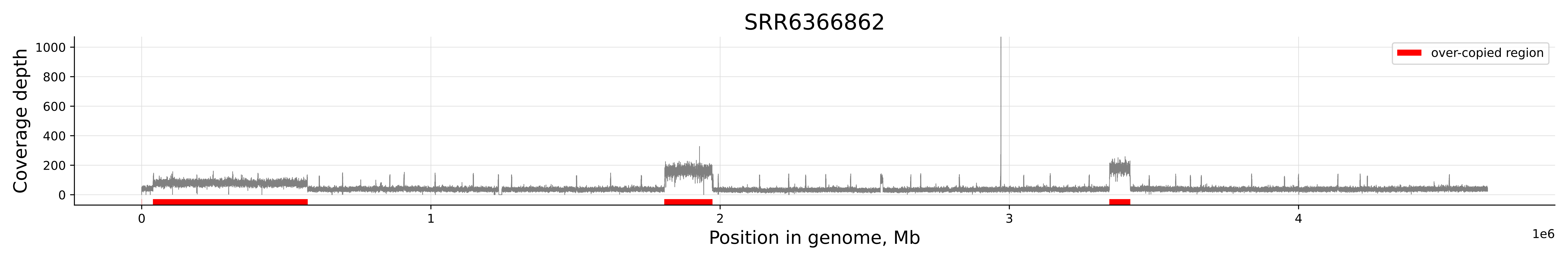

SRR6366862
Coverage depth
Position in genome, Mb
over-copied region
0
200
400
600
800
1000
1
2
3
4
1e6

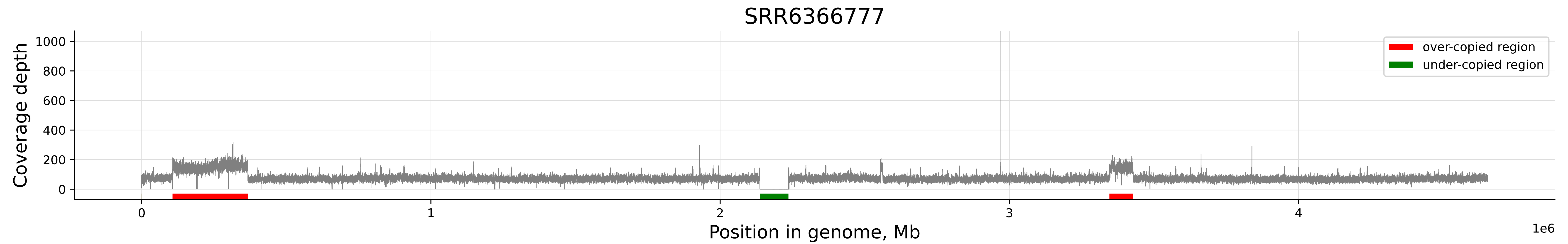
SRR6366777
Coverage depth
1000
800
600
400
200
0
0
1
2
3
4
1e6
Position in genome, Mb
over-copied region
under-copied region

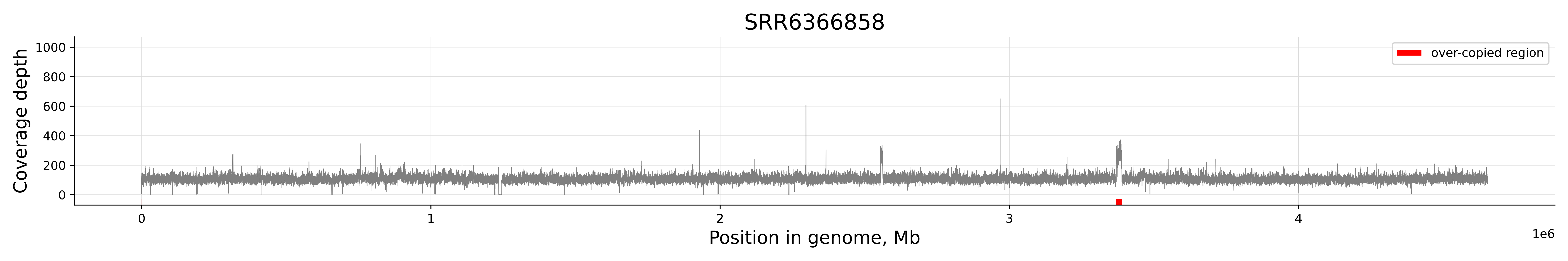

SRR6366858
Coverage depth
1000
800
600
400
200
0
over-copied region
0
1
2
3
4
1e6
Position in genome, Mb

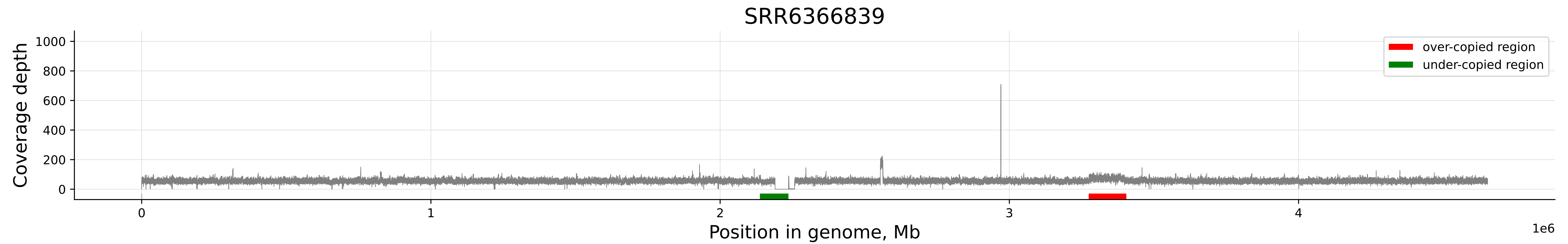
SRR6366839
Coverage depth
1000
800
600
400
200
0
over-copied region
under-copied region
0
1
2
3
4
1e6
Position in genome, Mb

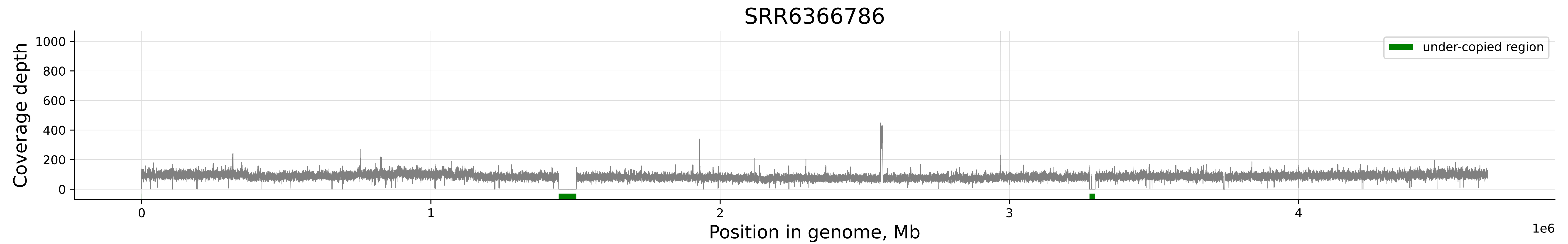

SRR6366786
Coverage depth
Position in genome, Mb
under-copied region
0
200
400
600
800
1000
0
1
2
3
4
1e6

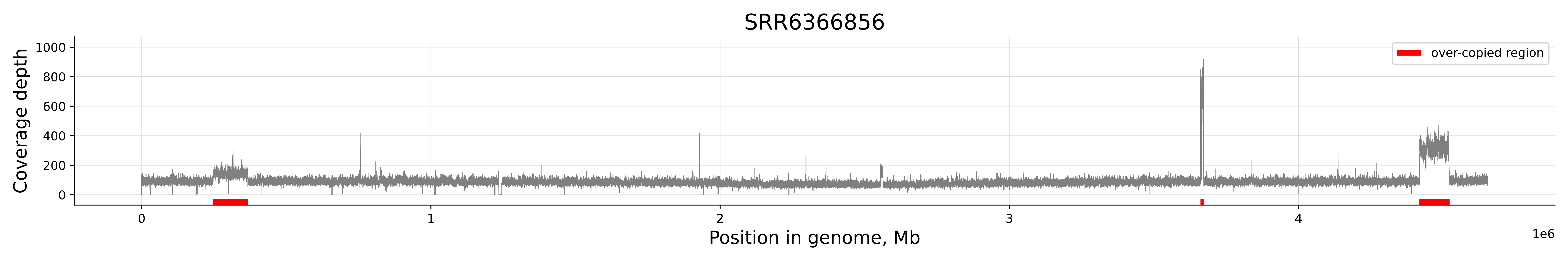
SRR6366856
over-copied region
Coverage depth
1000
800
600
400
200
0
0
1
2
3
4
1e6
Position in genome, Mb

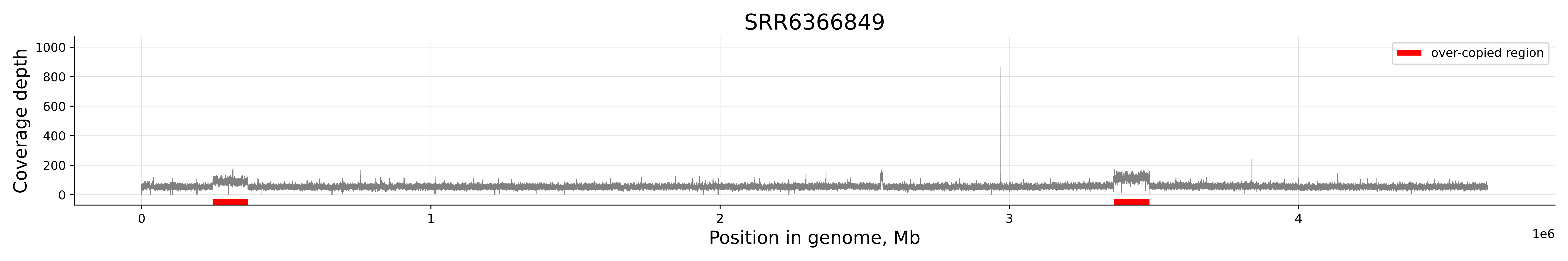

SRR6366849
Coverage depth
Position in genome, Mb
over-copied region
1e6

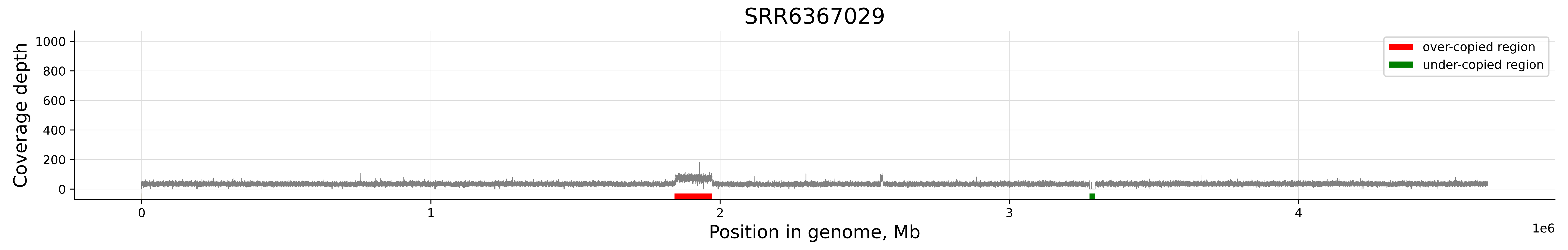
SRR6367029
Coverage depth
Position in genome, Mb
over-copied region
under-copied region
1000
800
600
400
200
0
0
1
2
3
4
1e6

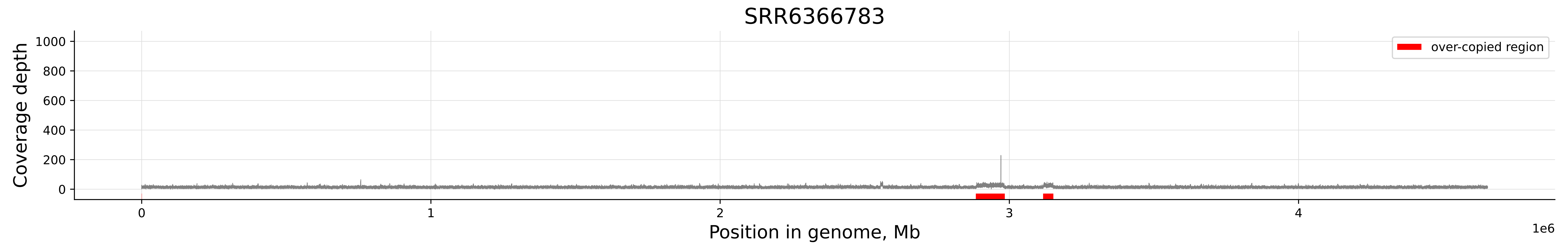

SRR6366783
Coverage depth
1000
800
600
400
200
0
over-copied region
0
1
2
3
4
1e6
Position in genome, Mb

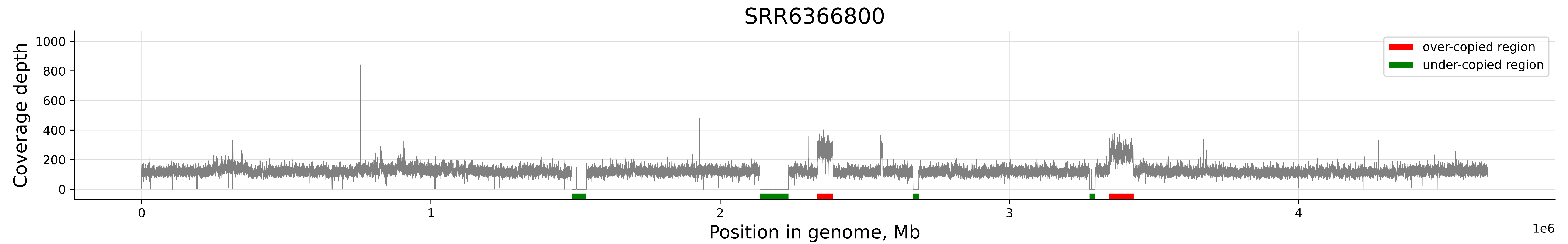

SRR6366800
over-copied region
under-copied region
Coverage depth
0
200
400
600
800
1000
0
1
2
3
4
1e6
Position in genome, Mb

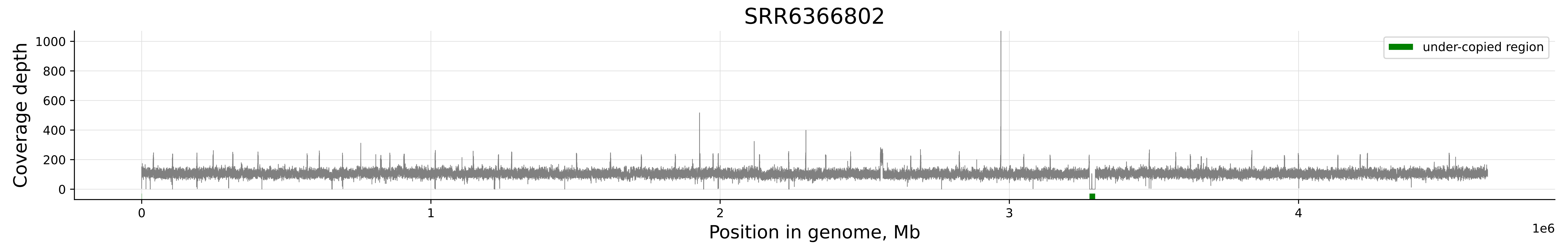
SRR6366802
Coverage depth
Position in genome, Mb
1e6
under-copied region
0
200
400
600
800
1000
0
1
2
3
4

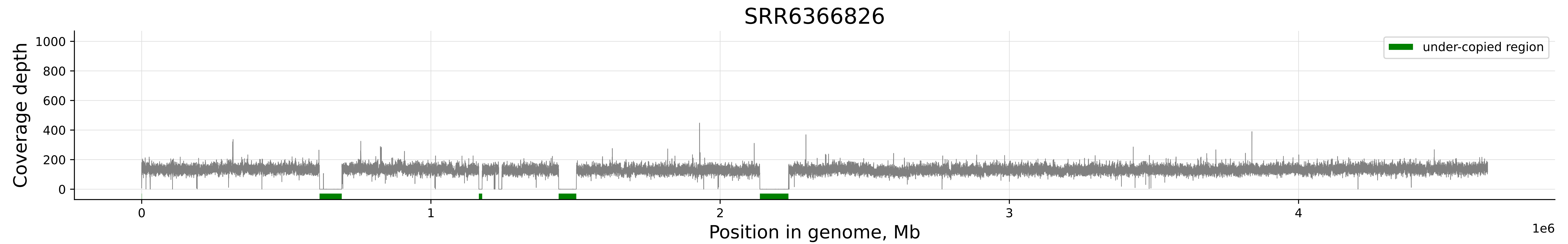
SRR6366826
Coverage depth
Position in genome, Mb
1e6
under-copied region
0
200
400
600
800
1000
0
1
2
3
4

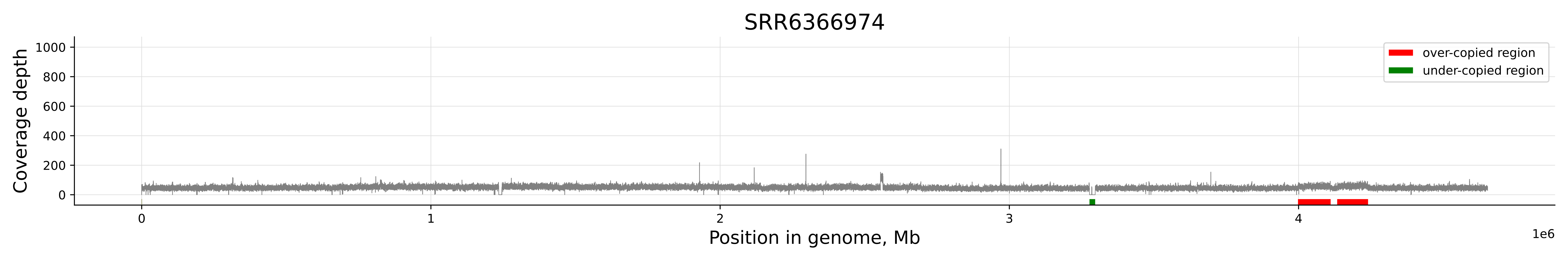
SRR6366974
over-copied region
under-copied region
Coverage depth
1000
800
600
400
200
0
0
1
2
3
4
1e6
Position in genome, Mb

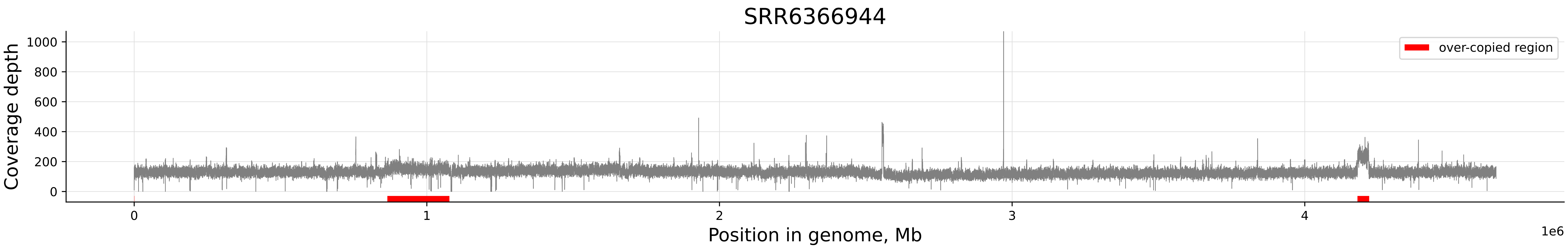
SRR6366944
over-copied region
Coverage depth
0
200
400
600
800
1000
0
1
2
3
4
1e6
Position in genome, Mb

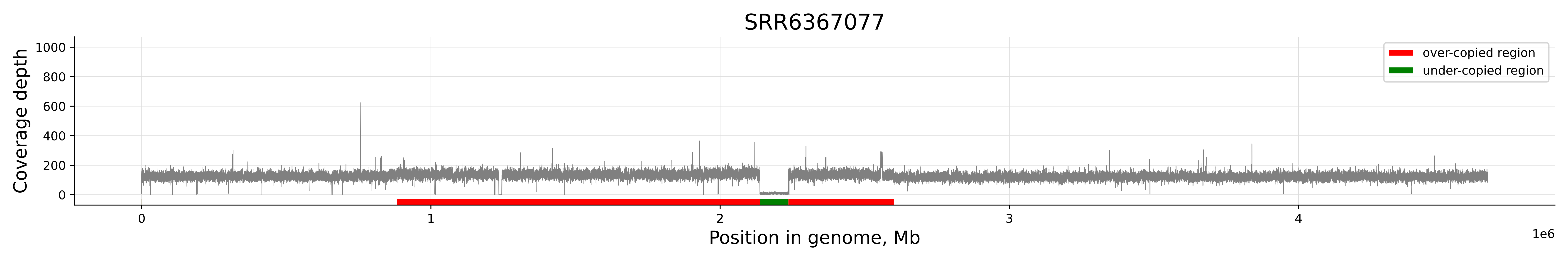
SRR6367077
Coverage depth
Position in genome, Mb
over-copied region
under-copied region
0
200
400
600
800
1000
0
1
2
3
4
1e6

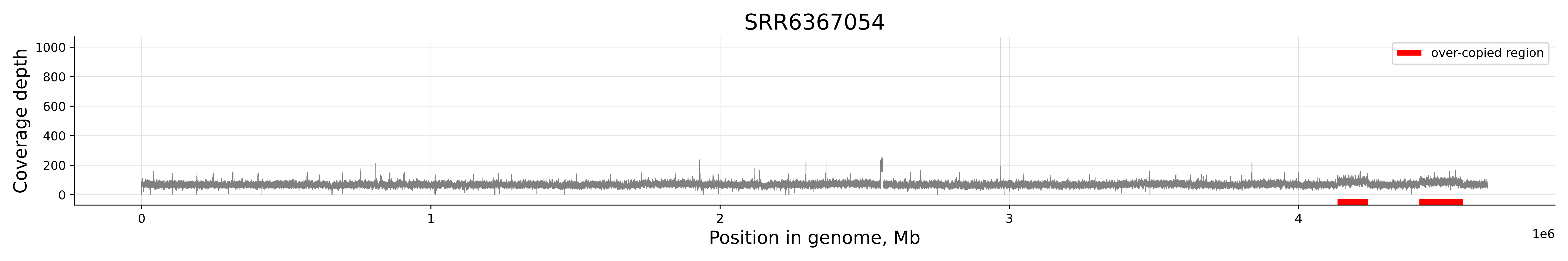

SRR6367054
Coverage depth
Position in genome, Mb
over-copied region
1000
800
600
400
200
0
0
1
2
3
4
1e6

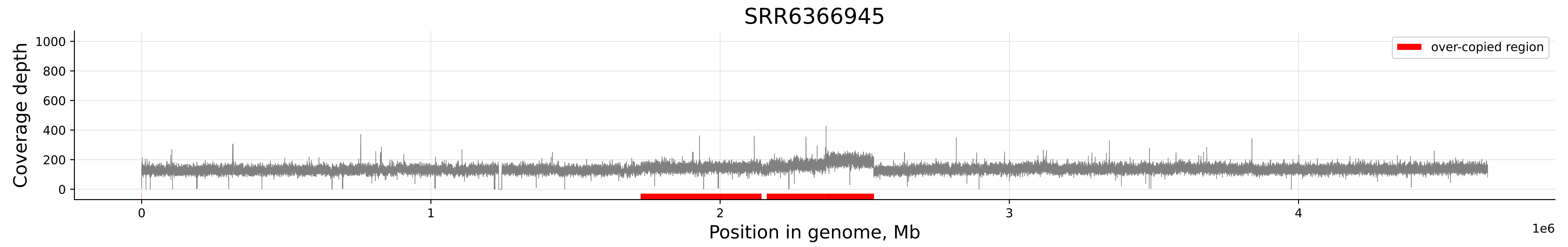
SRR6366945
Coverage depth
Position in genome, Mb
over-copied region
1000
800
600
400
200
0
0
1
2
3
4
1e6

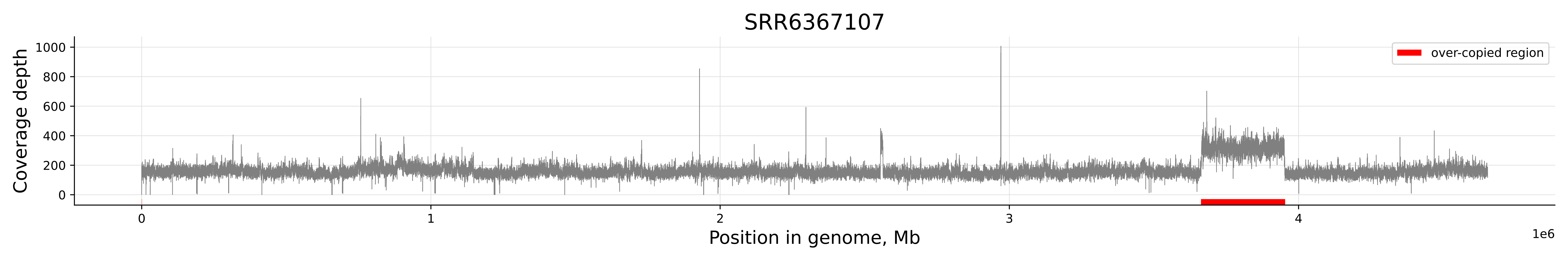

SRR6367107
Coverage depth
Position in genome, Mb
over-copied region
1000
800
600
400
200
0
0
1
2
3
4
1e6

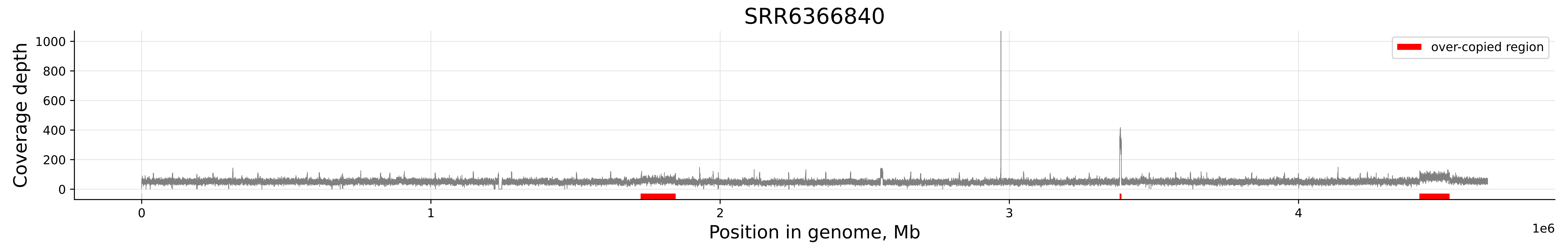

SRR6366840
Coverage depth
1000
800
600
400
200
0
over-copied region
0
1
2
3
4
1e6
Position in genome, Mb

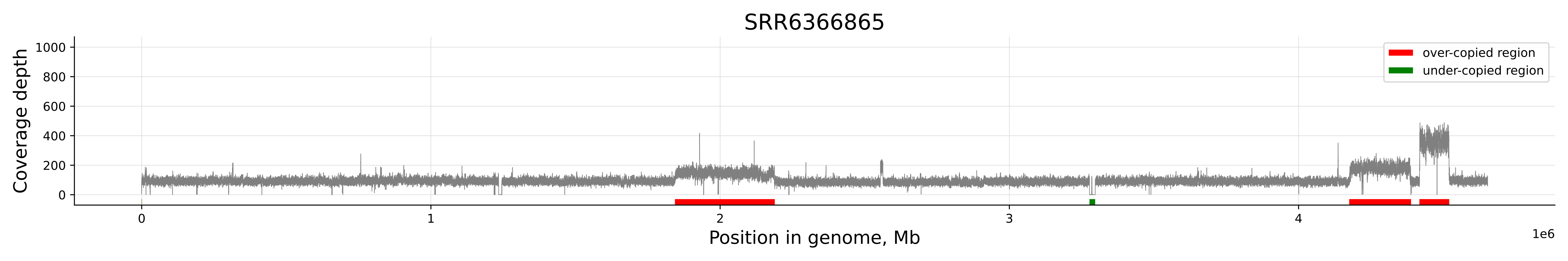
SRR6366865
Coverage depth
Position in genome, Mb
1e6
over-copied region
under-copied region
0
200
400
600
800
1000
0
1
2
3
4

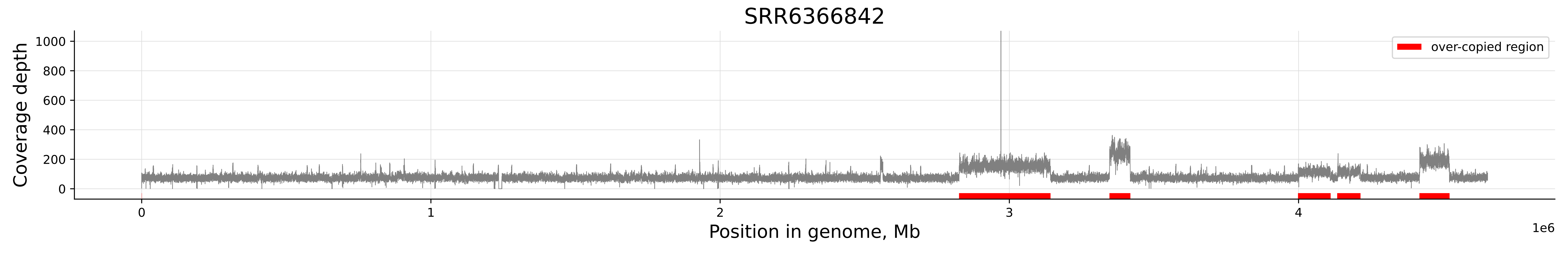

SRR6366842
Coverage depth
0
200
400
600
800
1000
over-copied region
0
1
2
3
4
Position in genome, Mb
1e6

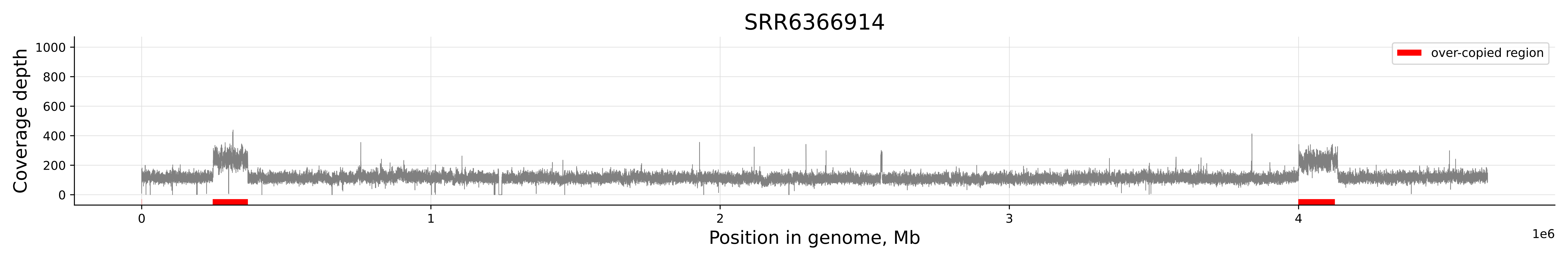

SRR6366914
Coverage depth
1000
800
600
400
200
0
0
1
2
3
4
Position in genome, Mb
1e6
over-copied region

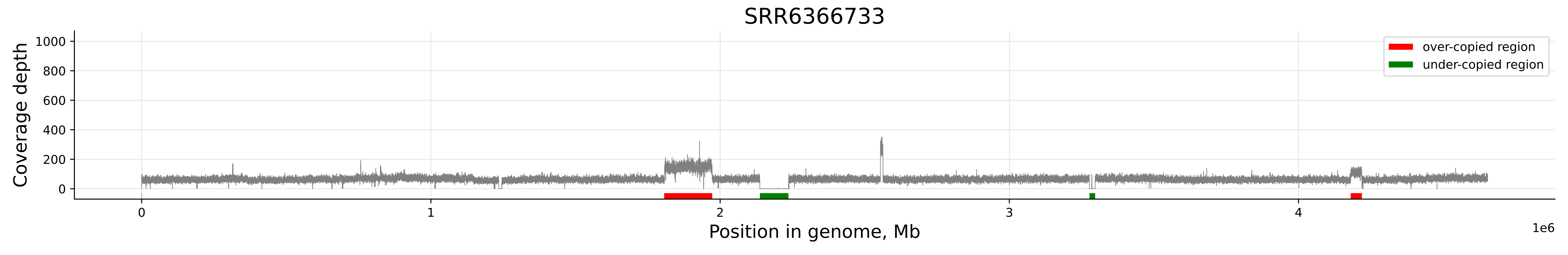

SRR6366733
Coverage depth
Position in genome, Mb
1e6
over-copied region
under-copied region
0
200
400
600
800
1000
1
2
3
4

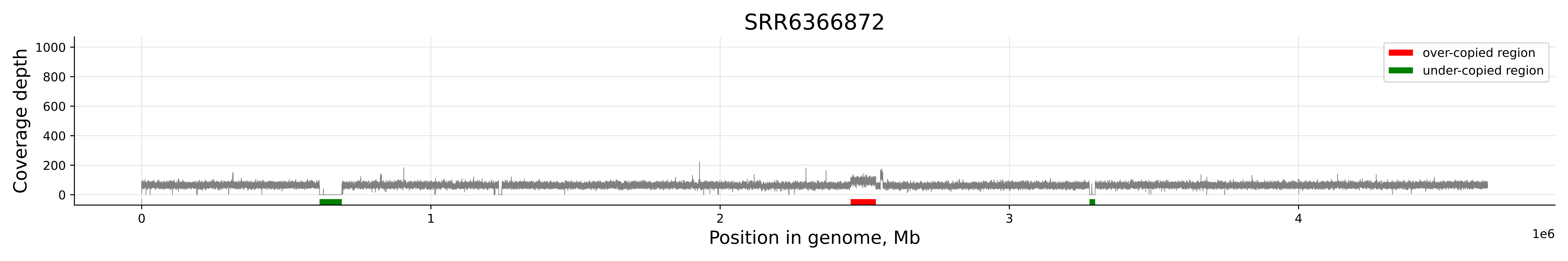
SRR6366872
Coverage depth
1000
800
600
400
200
0
0
1
2
3
4
Position in genome, Mb
1e6
over-copied region
under-copied region

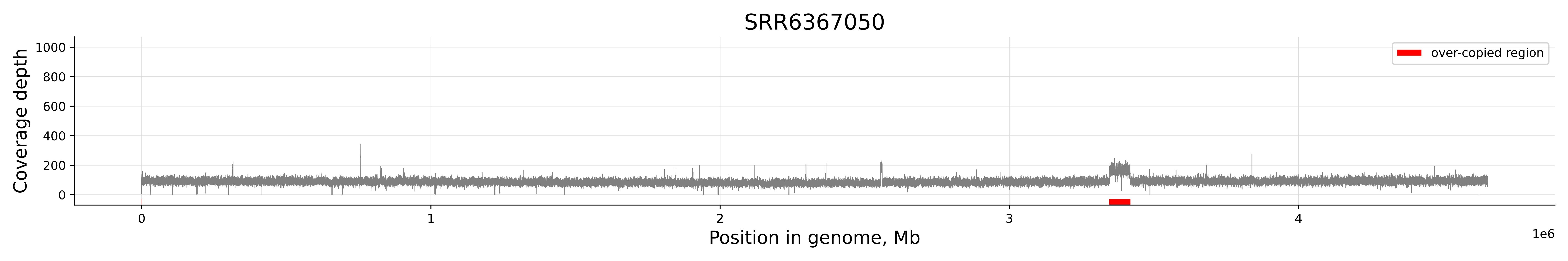

SRR6367050
Coverage depth
1000
800
600
400
200
0
over-copied region
0
1
2
3
4
1e6
Position in genome, Mb

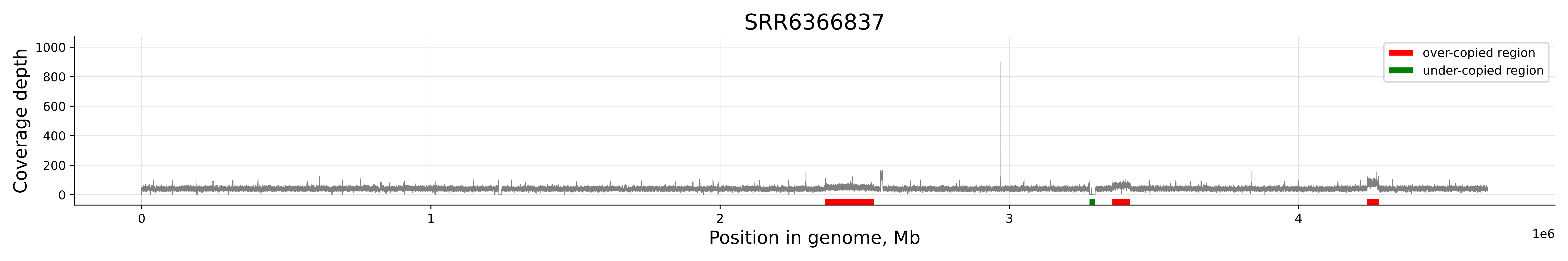
SRR6366837
over-copied region
under-copied region
Coverage depth
1000
800
600
400
200
0
0
1
2
3
4
1e6
Position in genome, Mb

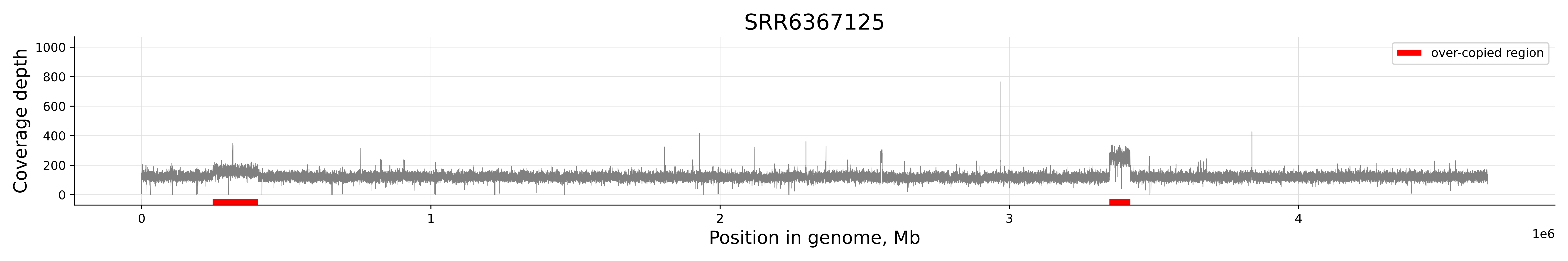
SRR6367125
over-copied region
Coverage depth
1000
800
600
400
200
0
0
1
2
3
4
1e6
Position in genome, Mb

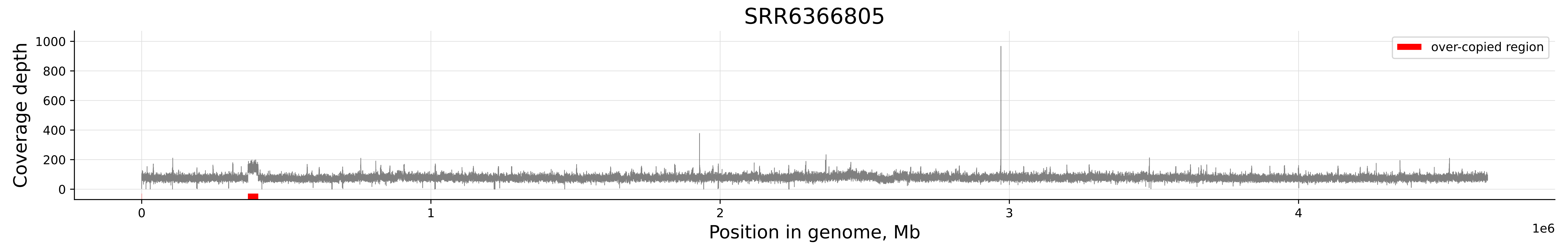

SRR6366805
Coverage depth
1000
800
600
400
200
0
over-copied region
0
1
2
3
4
1e6
Position in genome, Mb

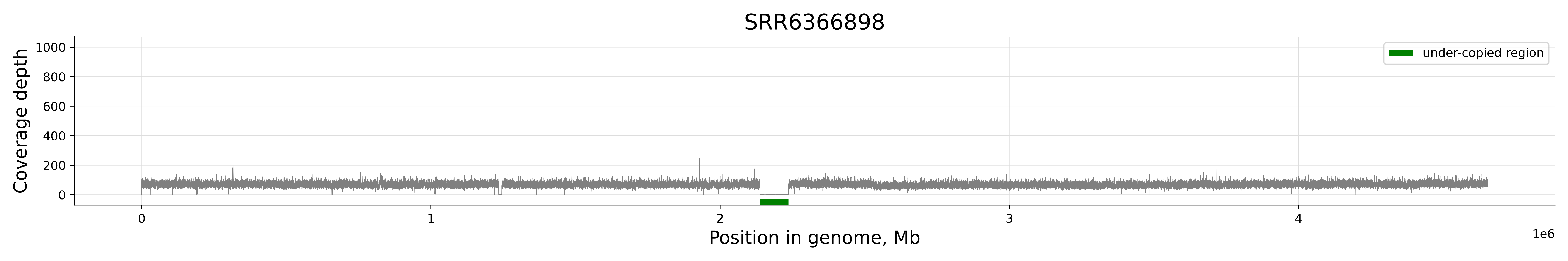
SRR6366898
under-copied region
Coverage depth
1000
800
600
400
200
0
0
1
2
3
4
1e6
Position in genome, Mb

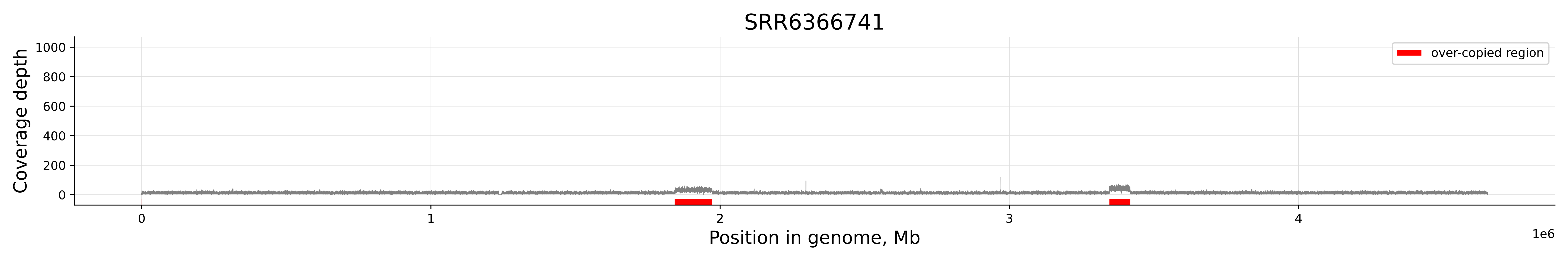
SRR6366741
over-copied region
Coverage depth
0
200
400
600
800
1000
0
1
2
3
4
1e6
Position in genome, Mb

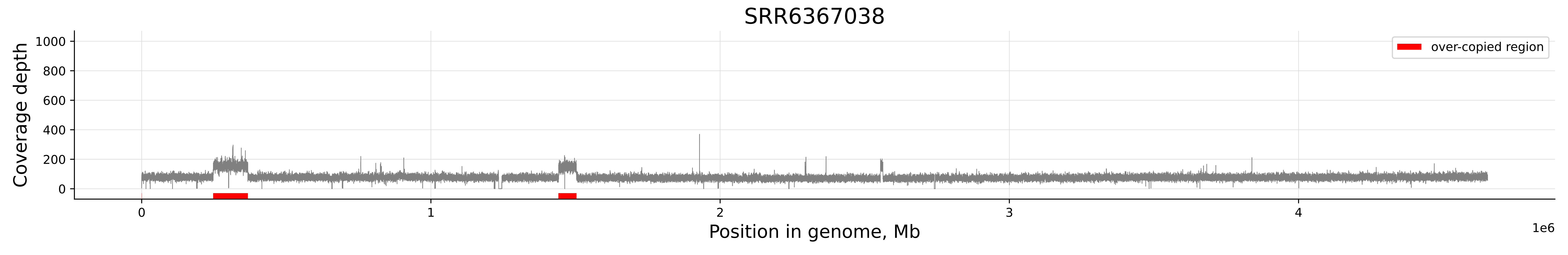
SRR6367038
Coverage depth
Position in genome, Mb
over-copied region
1e6
0
200
400
600
800
1000
0
1
2
3
4

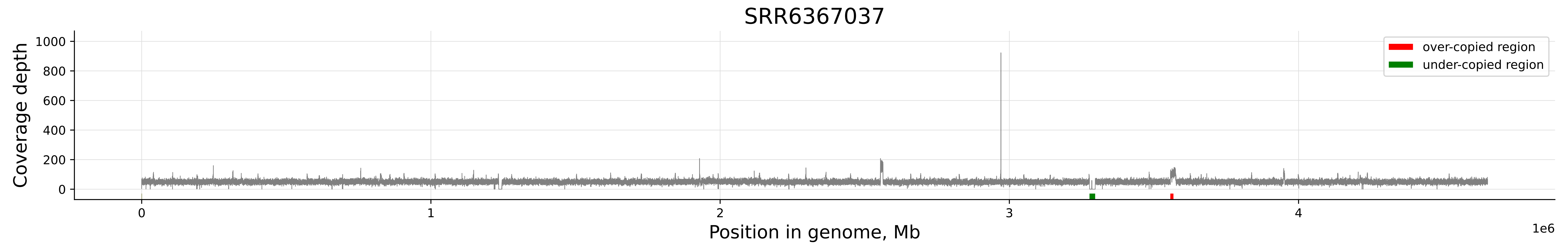

SRR6367037
Coverage depth
Position in genome, Mb
1e6
over-copied region
under-copied region
0
200
400
600
800
1000
0
1
2
3
4

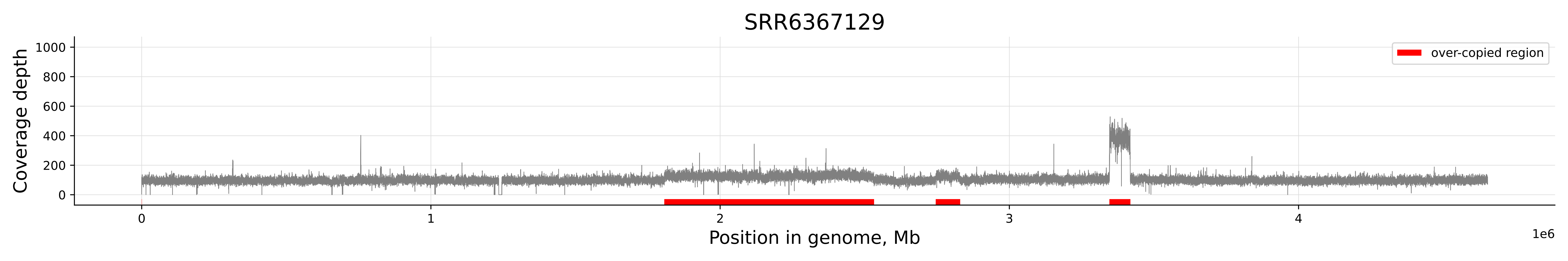
SRR6367129
Coverage depth
1000
800
600
400
200
0
over-copied region
0
1
2
3
4
1e6
Position in genome, Mb

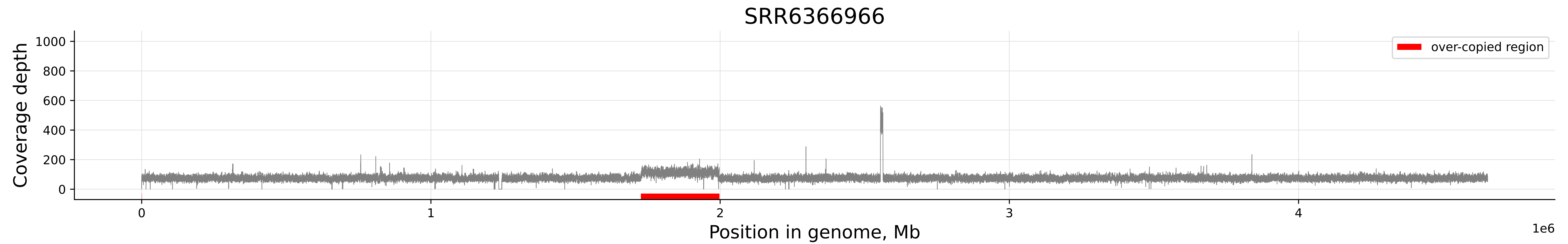
SRR6366966
Coverage depth
1000
800
600
400
200
0
0
1
2
3
4
1e6
Position in genome, Mb
over-copied region

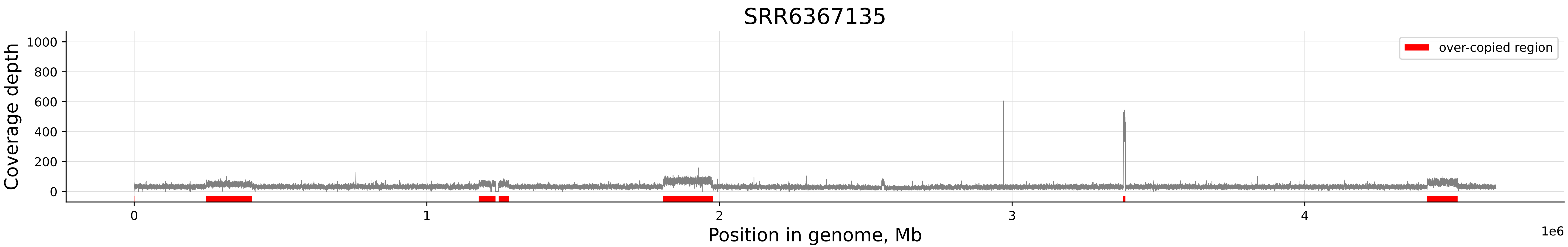

SRR6367135
Coverage depth
Position in genome, Mb
over-copied region
1e6

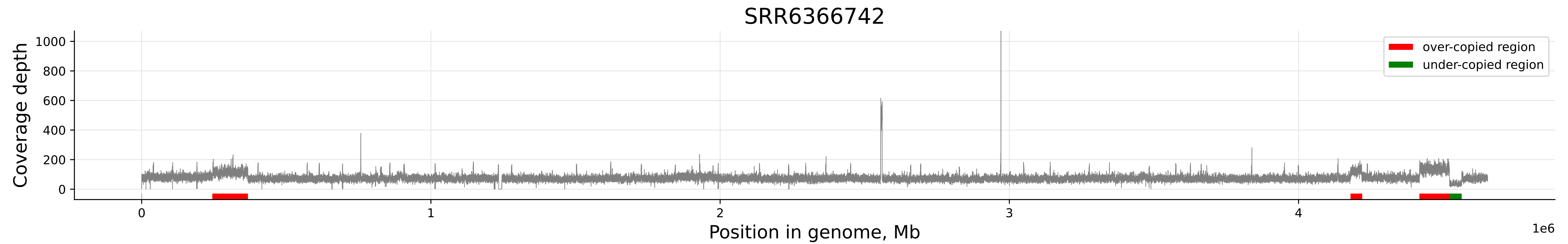

SRR6366742
over-copied region
under-copied region
Coverage depth
1000
800
600
400
200
0
0
1
2
3
4
1e6
Position in genome, Mb

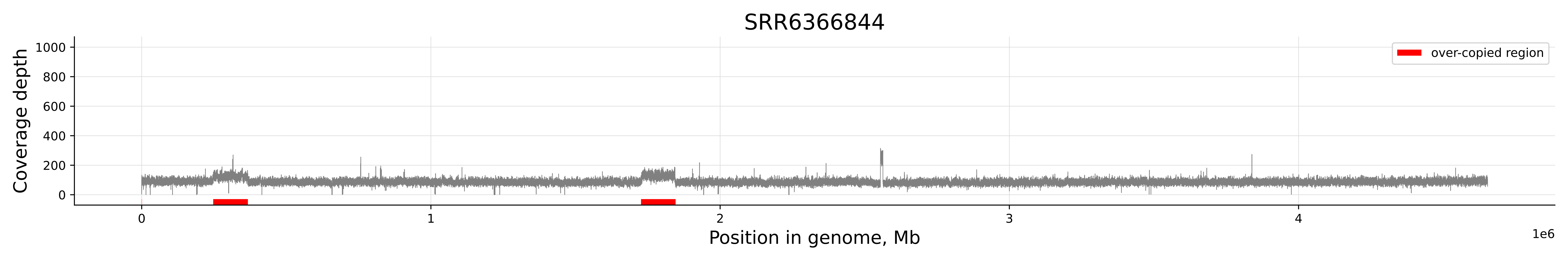
SRR6366844
over-copied region
Coverage depth
1000
800
600
400
200
0
0
1
2
3
4
1e6
Position in genome, Mb

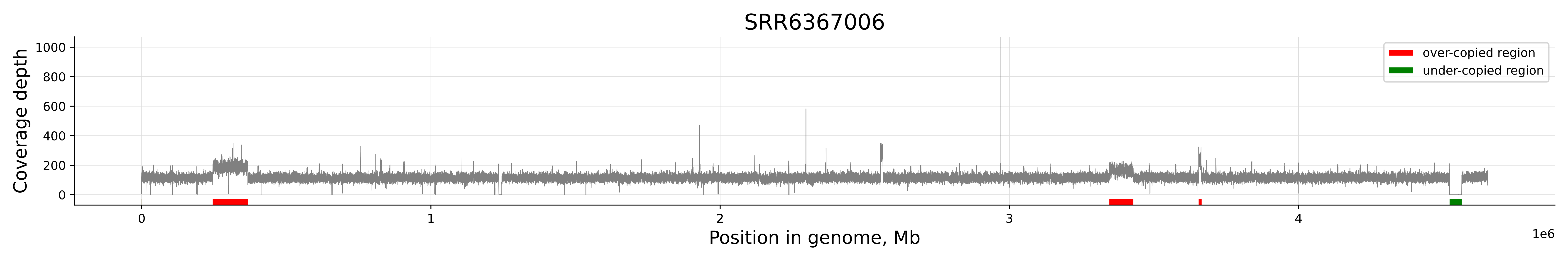

SRR6367006
Coverage depth
1000
800
600
400
200
0
0
1
2
3
4
1e6
Position in genome, Mb
over-copied region
under-copied region

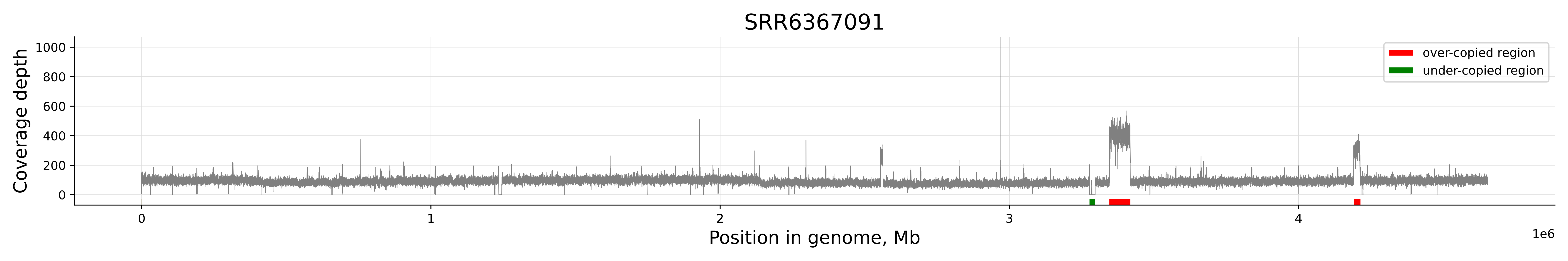
SRR6367091
Coverage depth
Position in genome, Mb
1e6
over-copied region
under-copied region
0
200
400
600
800
1000
0
1
2
3
4

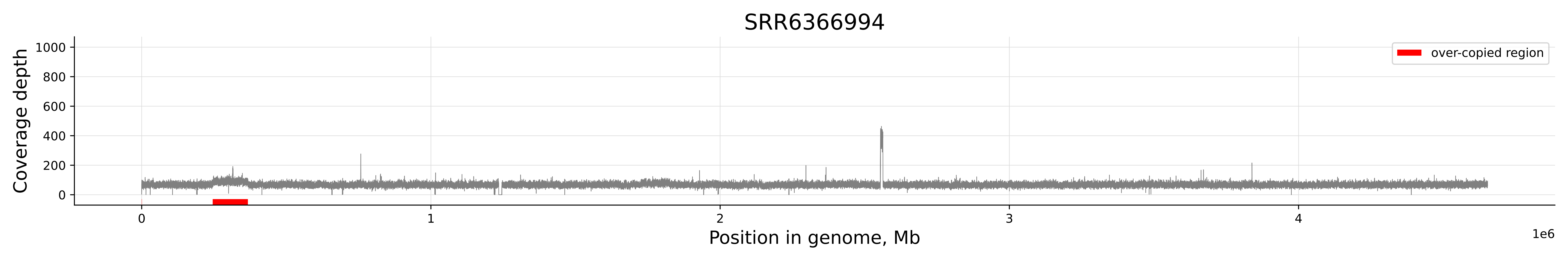

SRR6366994
Coverage depth
1000
800
600
400
200
0
0
1
2
3
4
1e6
Position in genome, Mb
over-copied region

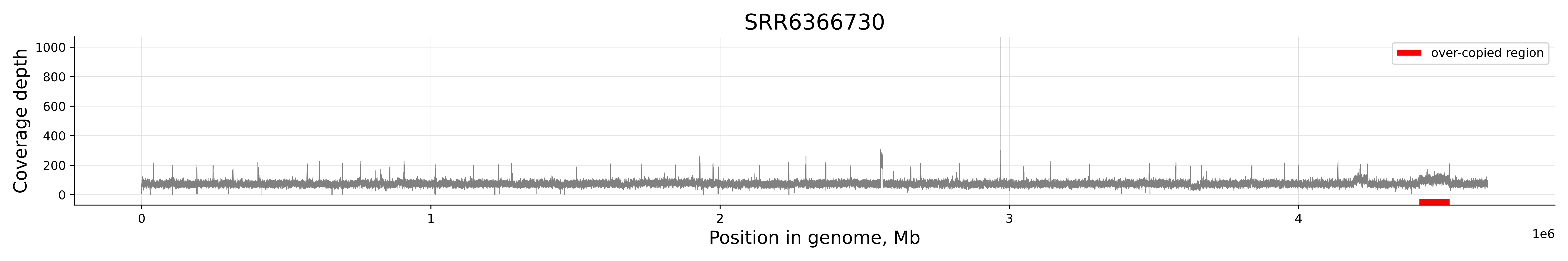

SRR6366730
Coverage depth
1000
800
600
400
200
0
0
1
2
3
4
1e6
Position in genome, Mb
over-copied region

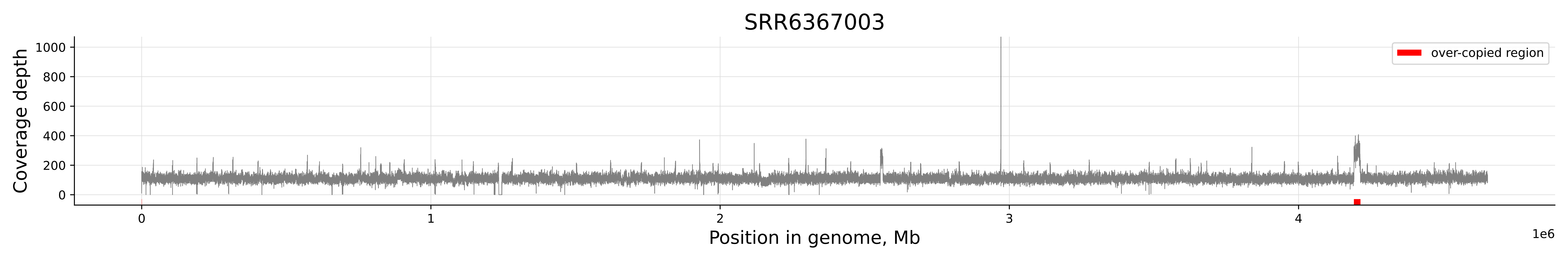

SRR6367003
Coverage depth
1000
800
600
400
200
0
over-copied region
0
1
2
3
4
1e6
Position in genome, Mb

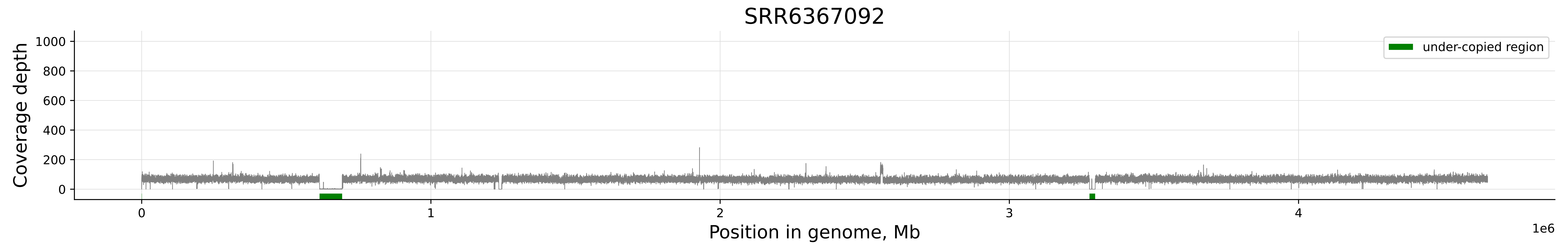
SRR6367092
under-copied region
Coverage depth
1000
800
600
400
200
0
0
1
2
3
4
1e6
Position in genome, Mb

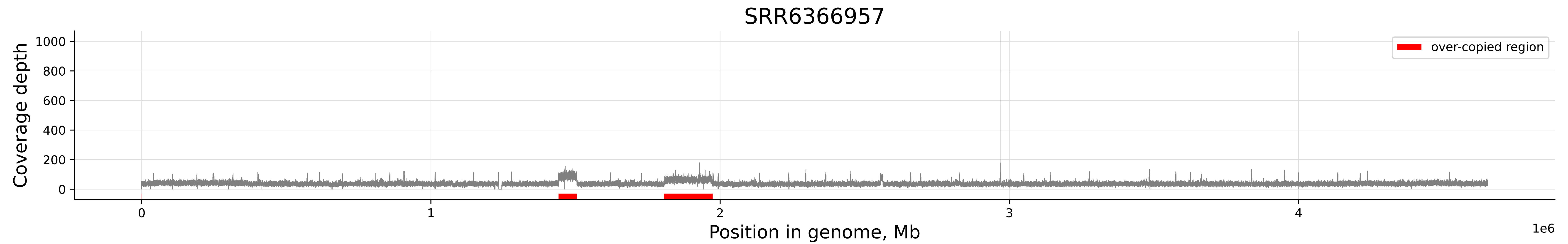

SRR6366957
Coverage depth
1000
800
600
400
200
0
over-copied region
0
1
2
3
4
1e6
Position in genome, Mb

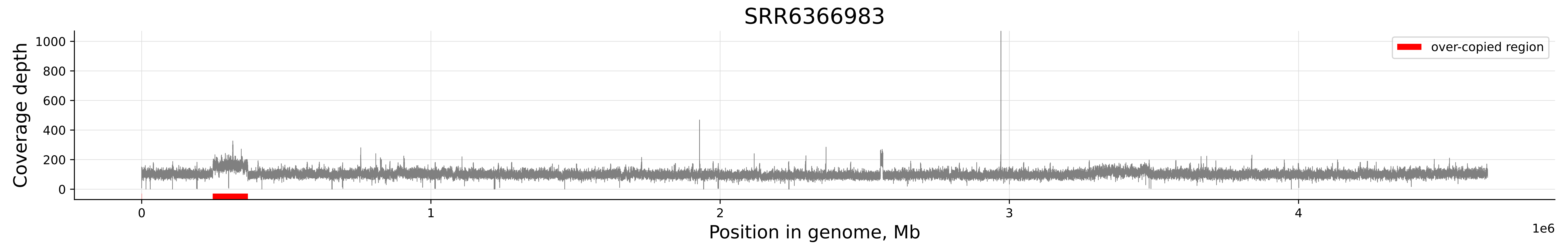
SRR6366983
over-copied region
Coverage depth
1000
800
600
400
200
0
0
1
2
3
4
1e6
Position in genome, Mb

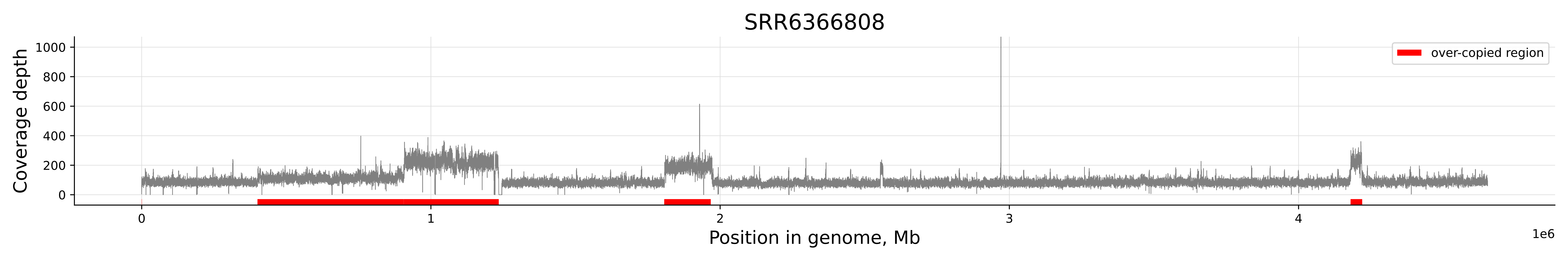

SRR6366808
Coverage depth
1000
800
600
400
200
0
0
1
2
3
4
1e6
Position in genome, Mb
over-copied region

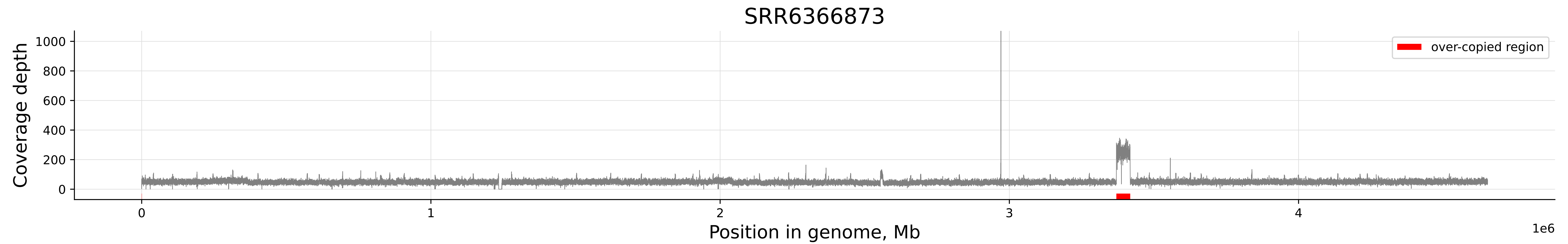

SRR6366873
Coverage depth
1000
800
600
400
200
0
over-copied region
0
1
2
3
4
1e6
Position in genome, Mb

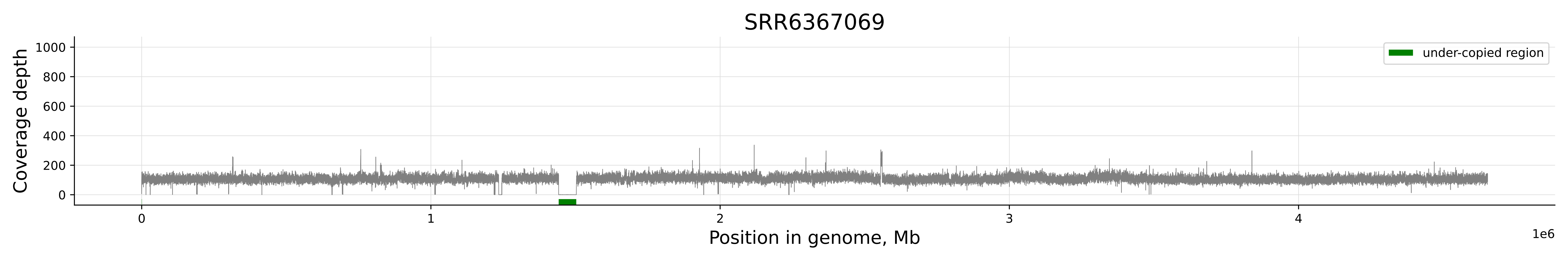
SRR6367069
under-copied region
Coverage depth
0
200
400
600
800
1000
0
1
2
3
4
1e6
Position in genome, Mb

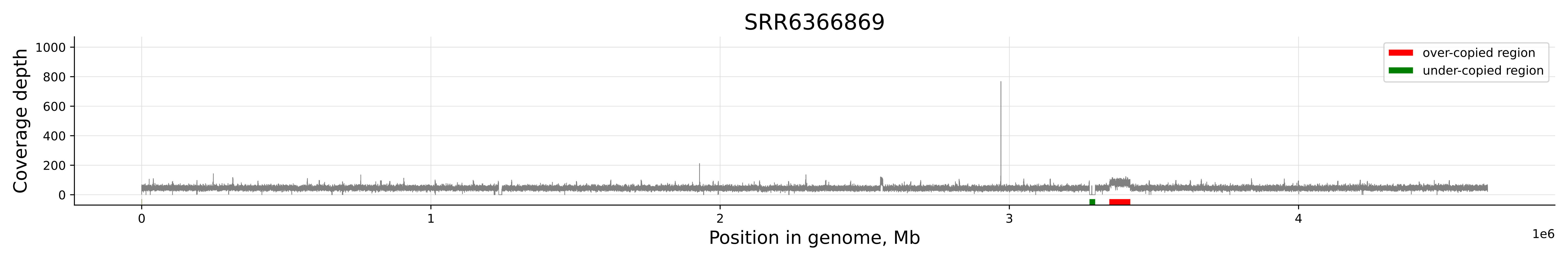
SRR6366869
Coverage depth
Position in genome, Mb
1e6
over-copied region
under-copied region
0
200
400
600
800
1000
0
1
2
3
4

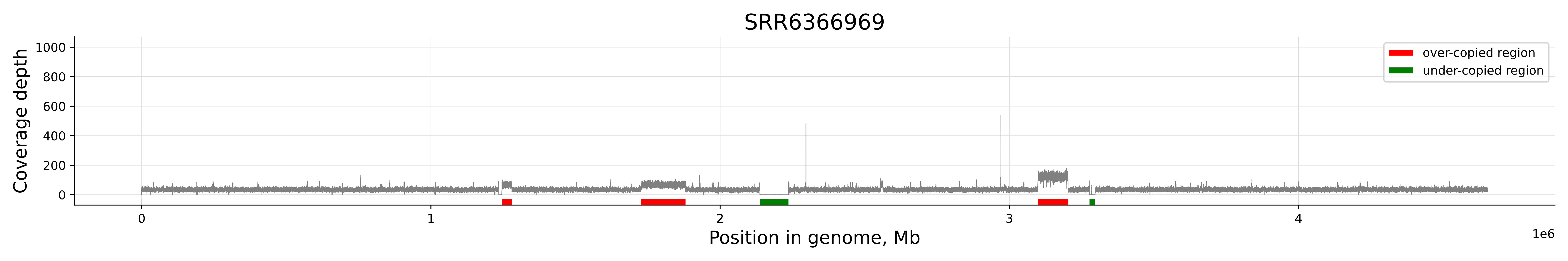
SRR6366969
Coverage depth
Position in genome, Mb
1e6
over-copied region
under-copied region
0
200
400
600
800
1000
0
1
2
3
4

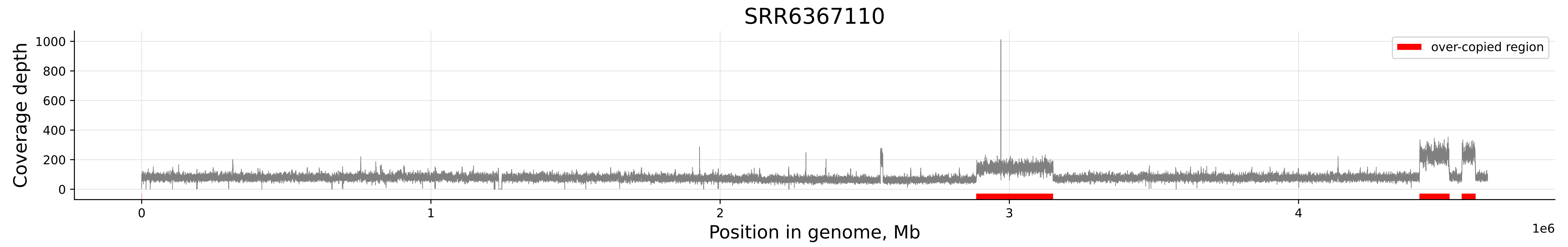

SRR6367110
Coverage depth
1000
800
600
400
200
0
over-copied region
0
1
2
3
4
1e6
Position in genome, Mb

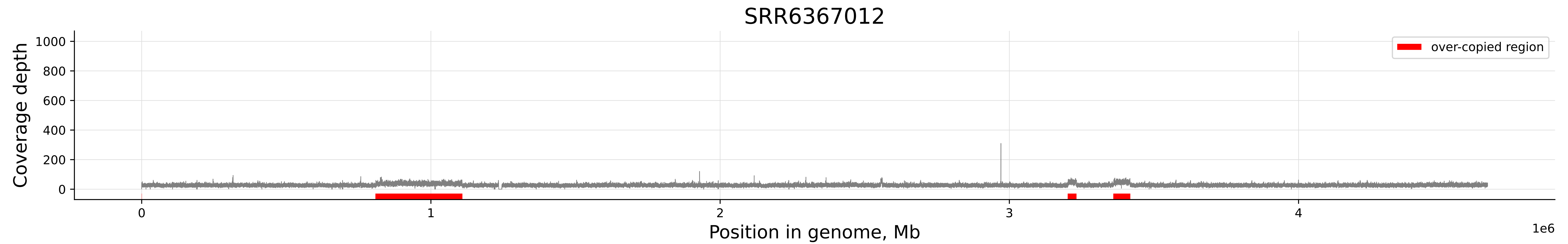
SRR6367012
Coverage depth
1000
800
600
400
200
0
0
1
2
3
4
1e6
Position in genome, Mb
over-copied region

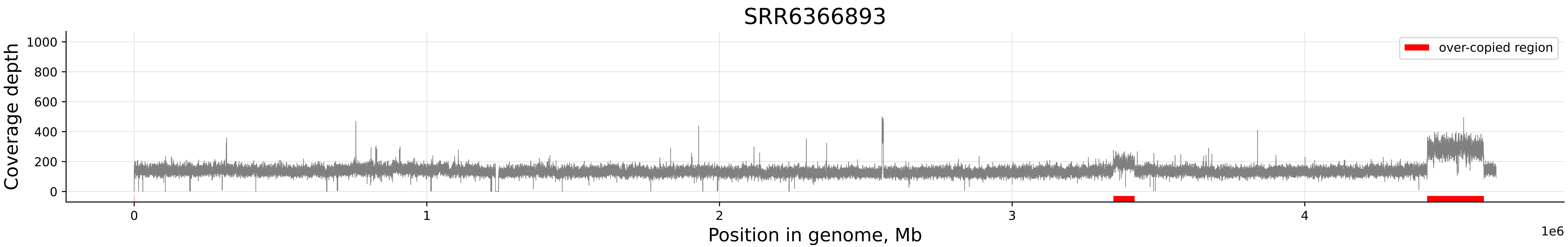
SRR6366893
over-copied region
Coverage depth
0
200
400
600
800
1000
0
1
2
3
4
1e6
Position in genome, Mb

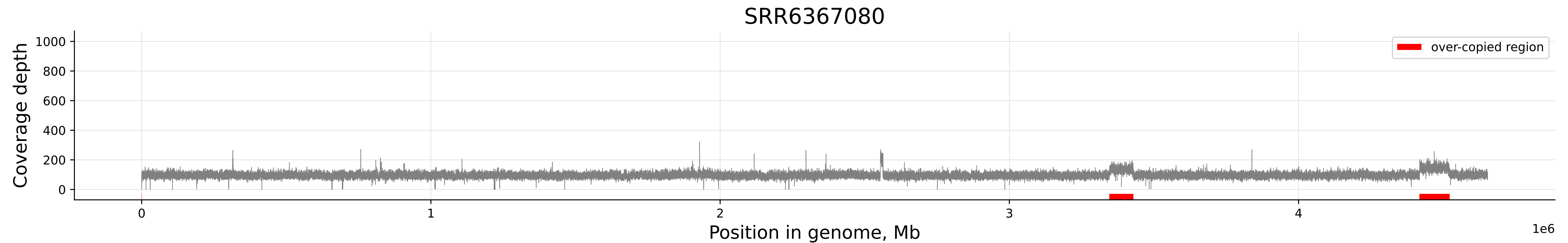

SRR6367080
Coverage depth
1000
800
600
400
200
0
over-copied region
0
1
2
3
4
Position in genome, Mb
1e6

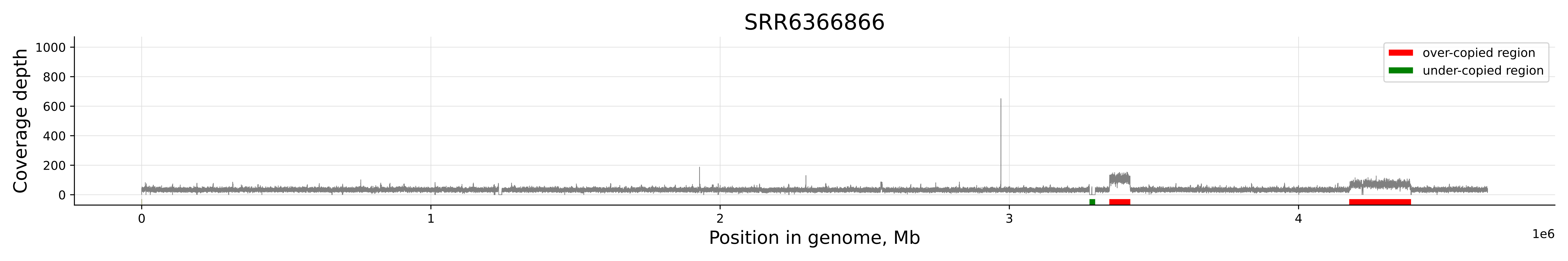

SRR6366866
Coverage depth
0
200
400
600
800
1000
0
1
2
3
4
1e6
Position in genome, Mb
over-copied region
under-copied region

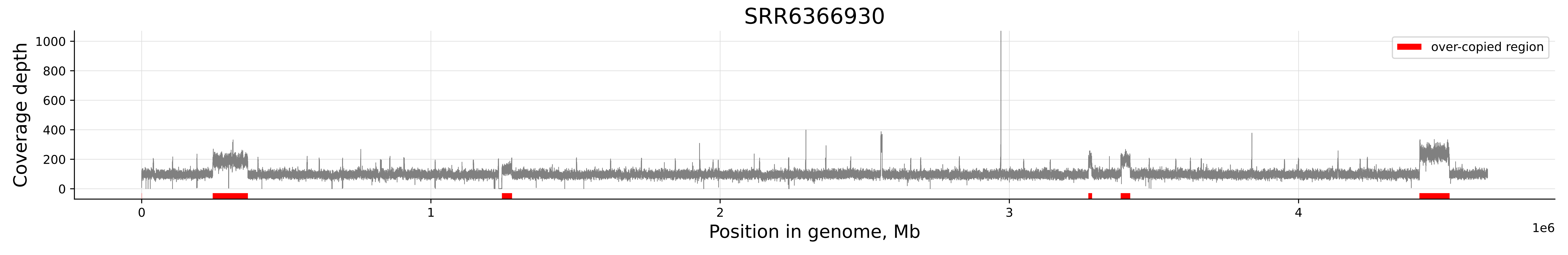

SRR6366930
over-copied region
Coverage depth
0
200
400
600
800
1000
Position in genome, Mb
0
1
2
3
4
1e6

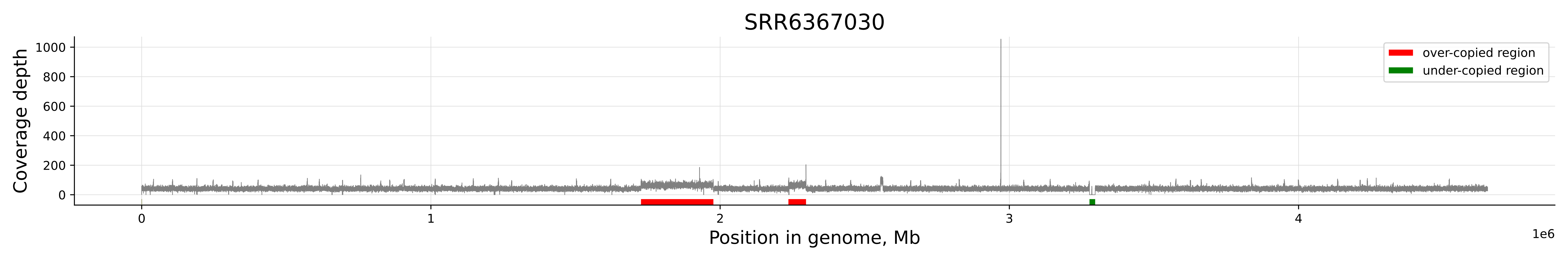
SRR6367030
Coverage depth
Position in genome, Mb
over-copied region
under-copied region
1e6
0
1
2
3
4
0
200
400
600
800
1000

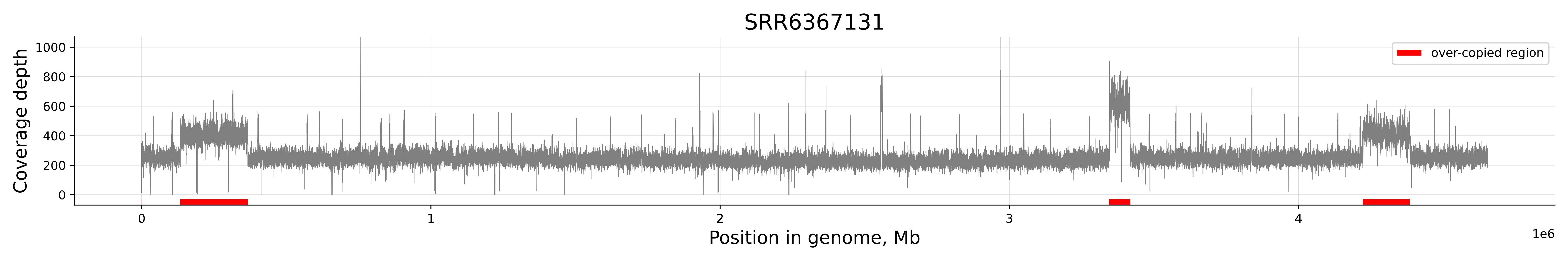
SRR6367131
Coverage depth
1000
800
600
400
200
0
0
1
2
3
4
1e6
Position in genome, Mb
over-copied region

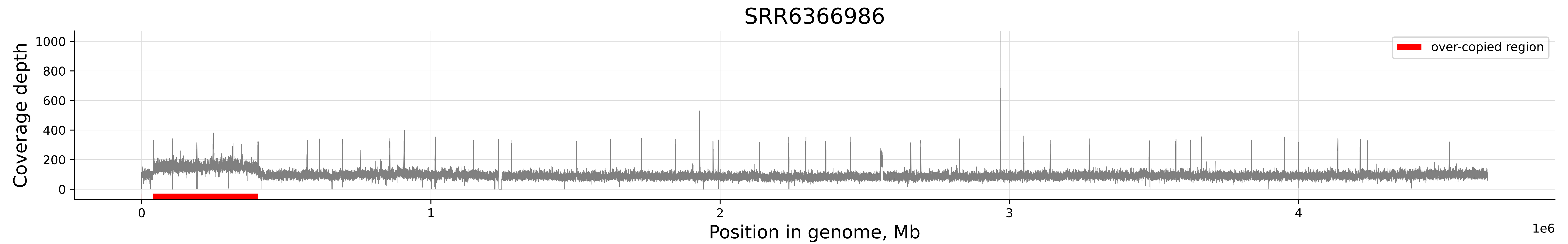
SRR6366986
Coverage depth
Position in genome, Mb
over-copied region
1e6
0
200
400
600
800
1000
0
1
2
3
4

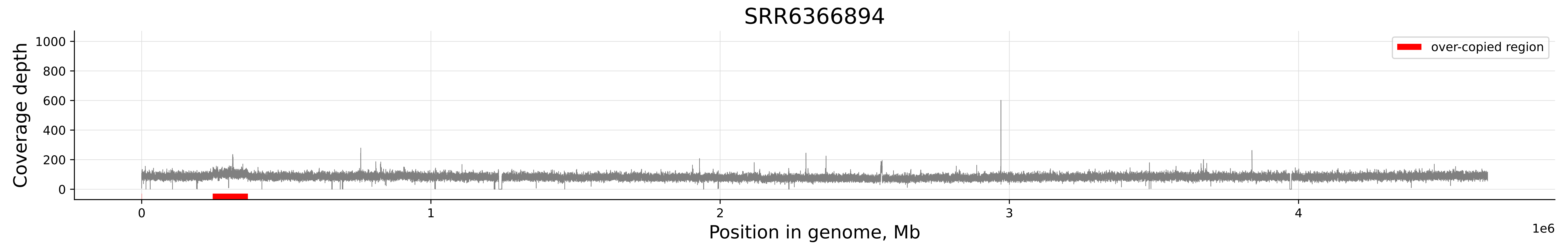
SRR6366894
Coverage depth
1000
800
600
400
200
0
0
1
2
3
4
1e6
Position in genome, Mb
over-copied region

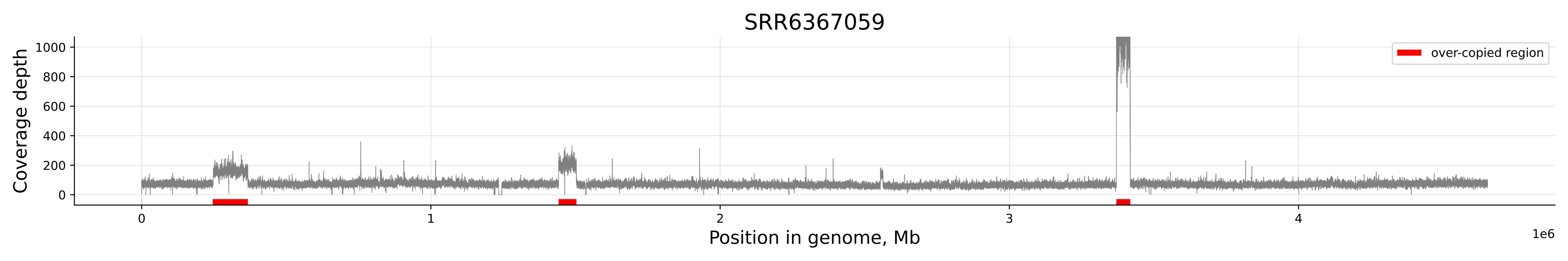
SRR6367059
Coverage depth
1000
800
600
400
200
0
0
1
2
3
4
1e6
Position in genome, Mb
over-copied region

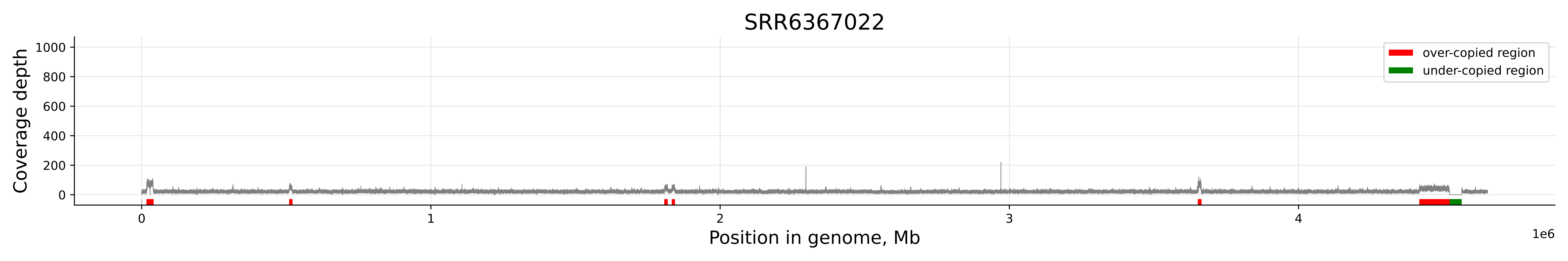
SRR6367022
Coverage depth
1000
800
600
400
200
0
0
1
2
3
4
1e6
Position in genome, Mb
over-copied region
under-copied region

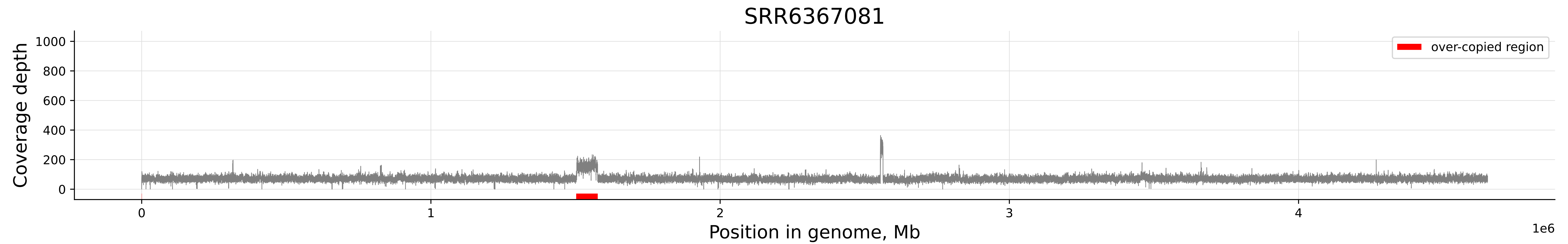

SRR6367081
Coverage depth
1000
800
600
400
200
0
over-copied region
0
1
2
3
4
1e6
Position in genome, Mb

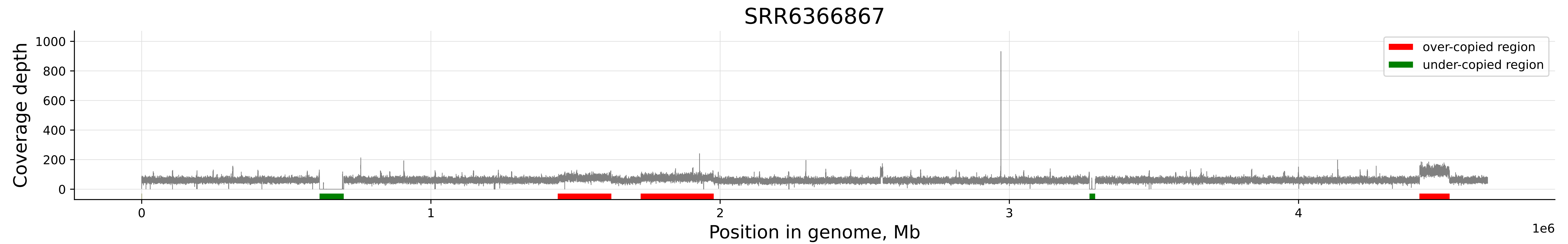

SRR6366867
Coverage depth
1000
800
600
400
200
0
over-copied region
under-copied region
0
1
2
3
4
Position in genome, Mb
1e6

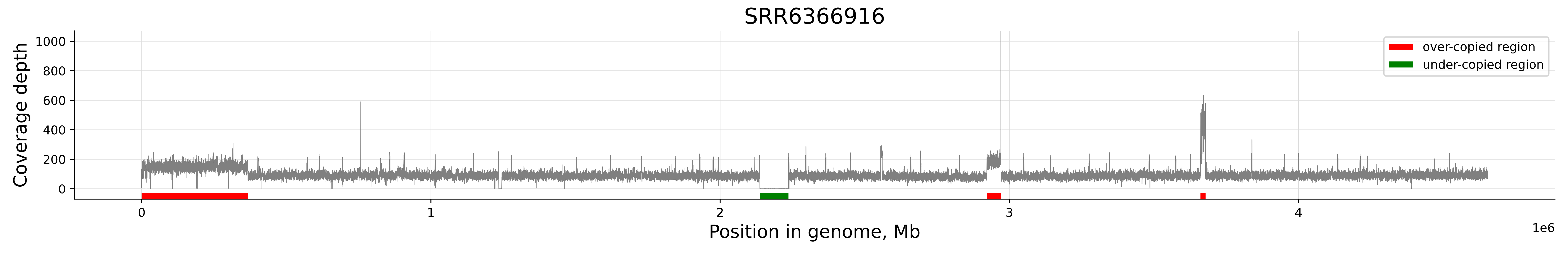
SRR6366916
over-copied region
under-copied region
Coverage depth
0
200
400
600
800
1000
0
1
2
3
4
1e6
Position in genome, Mb

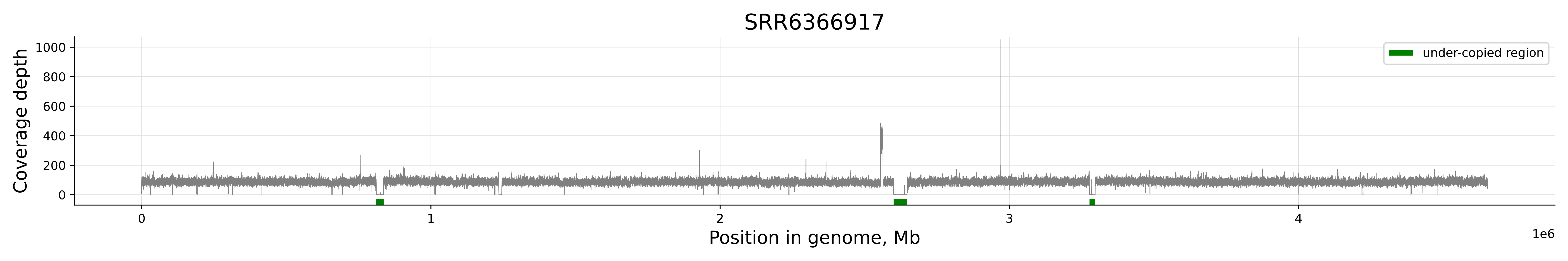

SRR6366917
Coverage depth
1000
800
600
400
200
0
under-copied region
0
1
2
3
4
1e6
Position in genome, Mb

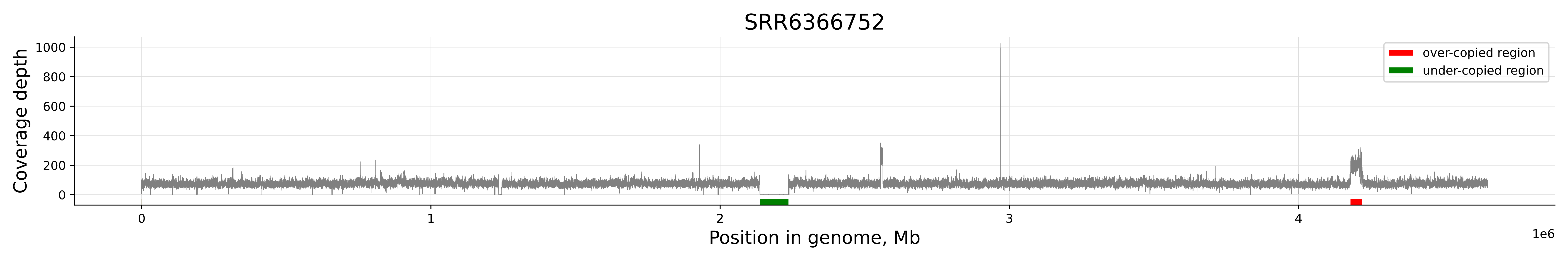

SRR6366752
Coverage depth
1000
800
600
400
200
0
0
1
2
3
4
1e6
Position in genome, Mb
over-copied region
under-copied region

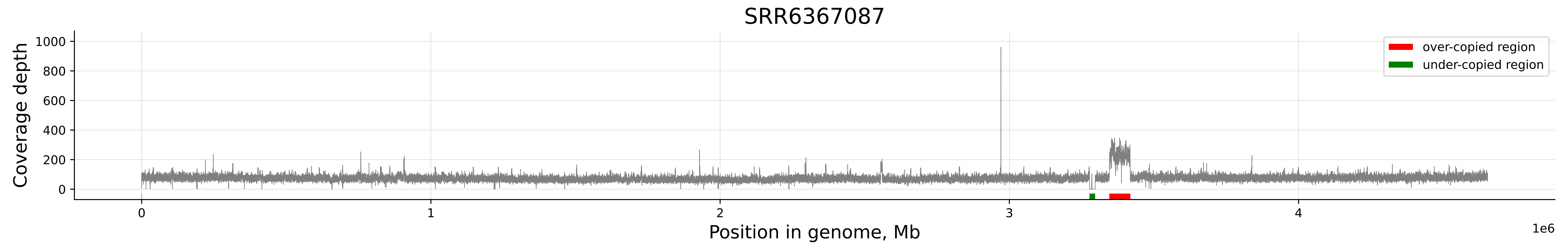

SRR6367087
Coverage depth
1000
800
600
400
200
0
over-copied region
under-copied region
0
1
2
3
4
1e6
Position in genome, Mb

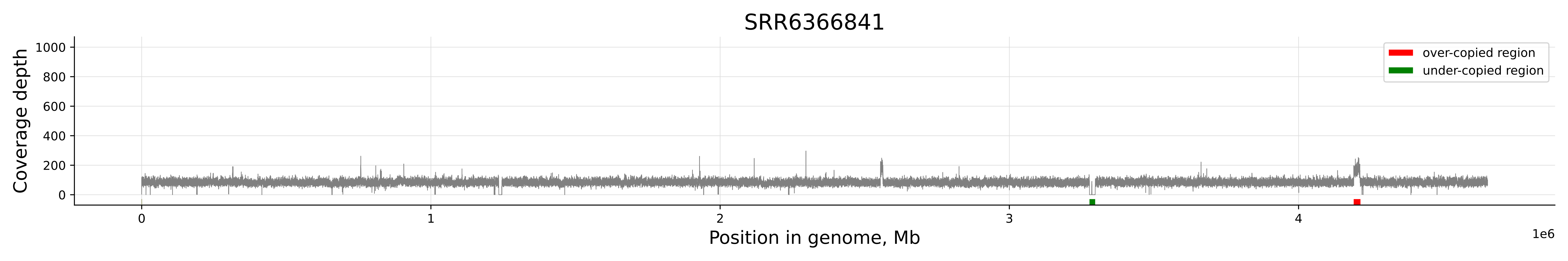

SRR6366841
Coverage depth
Position in genome, Mb
over-copied region
under-copied region
1e6

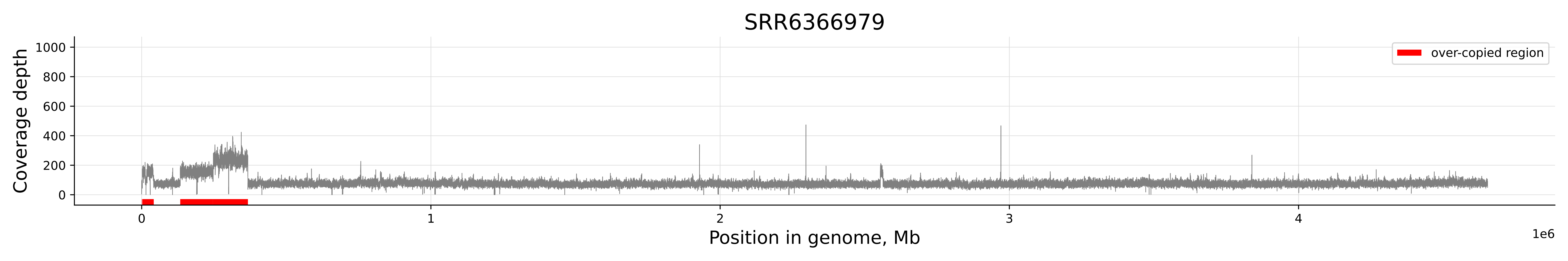

SRR6366979
over-copied region
Coverage depth
0
200
400
600
800
1000
0
1
2
3
4
1e6
Position in genome, Mb

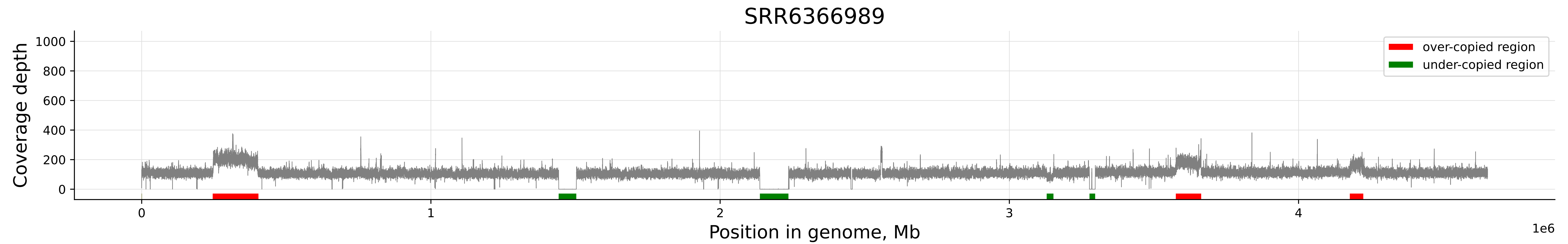

SRR6366989
Coverage depth
0
200
400
600
800
1000
0
1
2
3
4
1e6
Position in genome, Mb
over-copied region
under-copied region

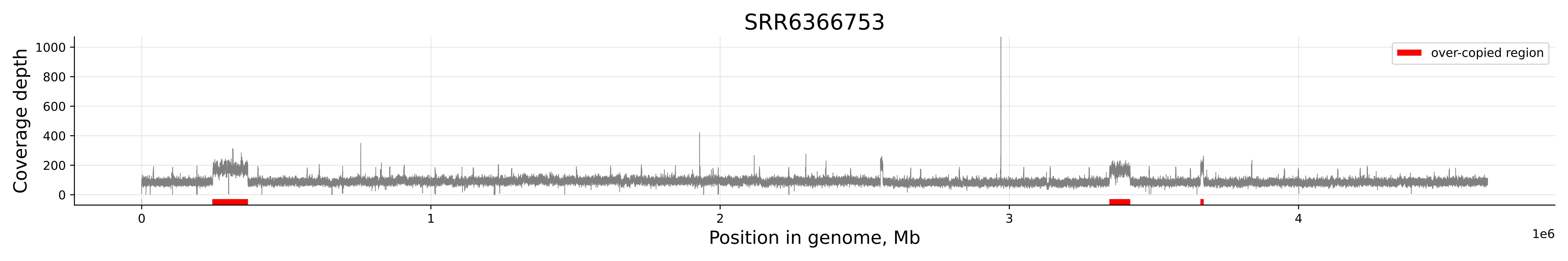

SRR6366753
Coverage depth
1000
800
600
400
200
0
over-copied region
0
1
2
3
4
1e6
Position in genome, Mb

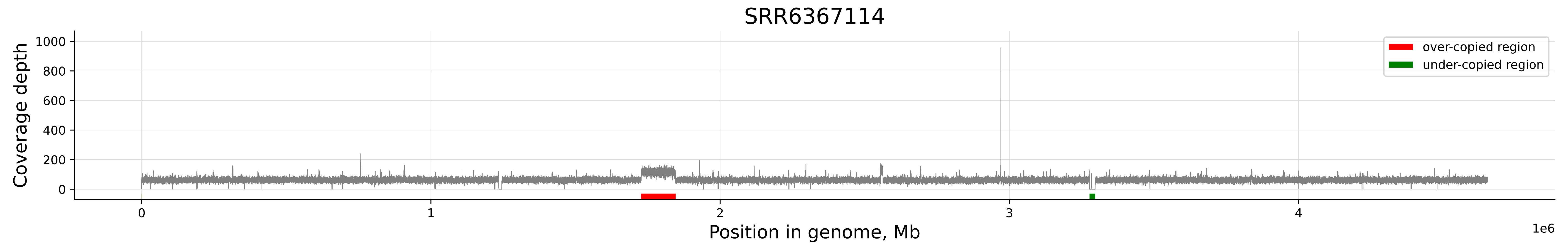

SRR6367114
Coverage depth
0
200
400
600
800
1000
over-copied region
under-copied region
0
1
2
3
4
1e6
Position in genome, Mb

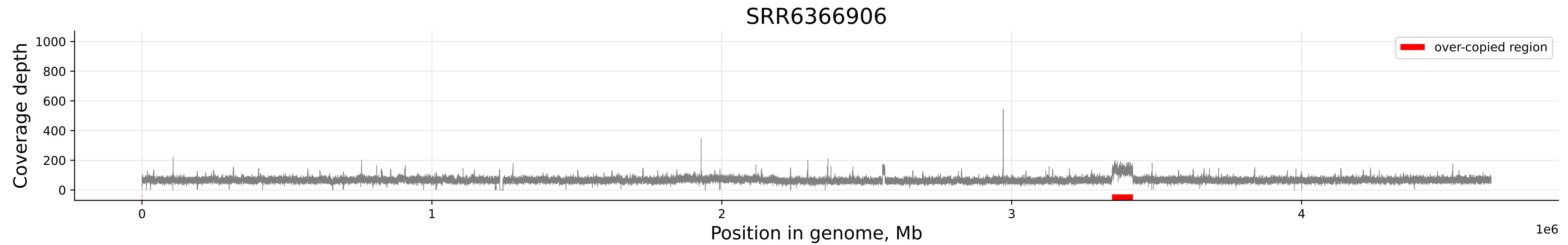

SRR6366906
Coverage depth
Position in genome, Mb
over-copied region
1e6

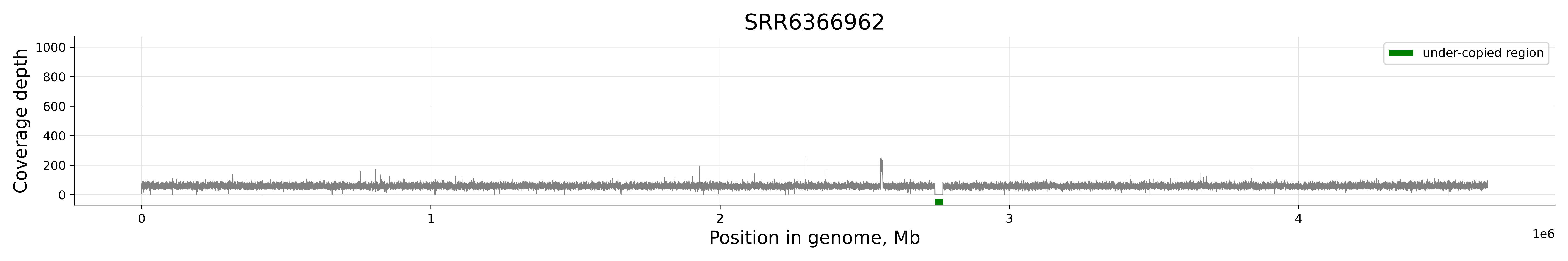
SRR6366962
under-copied region
Coverage depth
1000
800
600
400
200
0
0
1
2
3
4
Position in genome, Mb
1e6

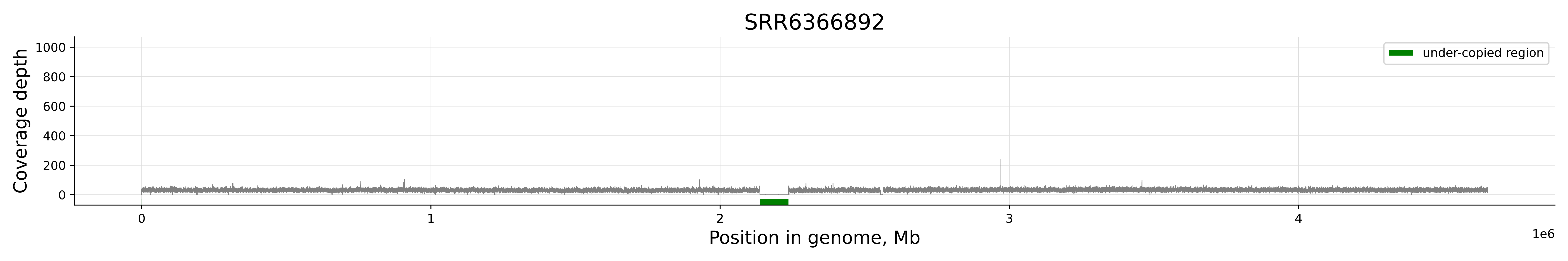
SRR6366892
Coverage depth
Position in genome, Mb
under-copied region
1e6

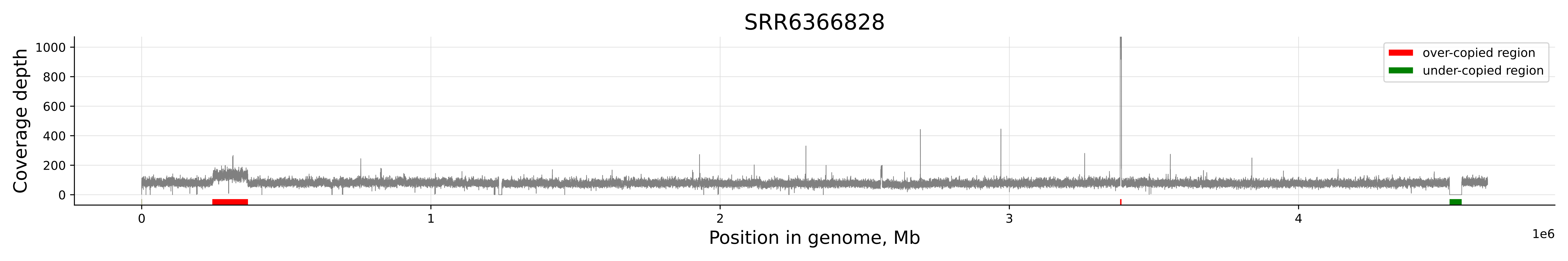
SRR6366828
Coverage depth
Position in genome, Mb
1e6
over-copied region
under-copied region
0
200
400
600
800
1000
0
1
2
3
4

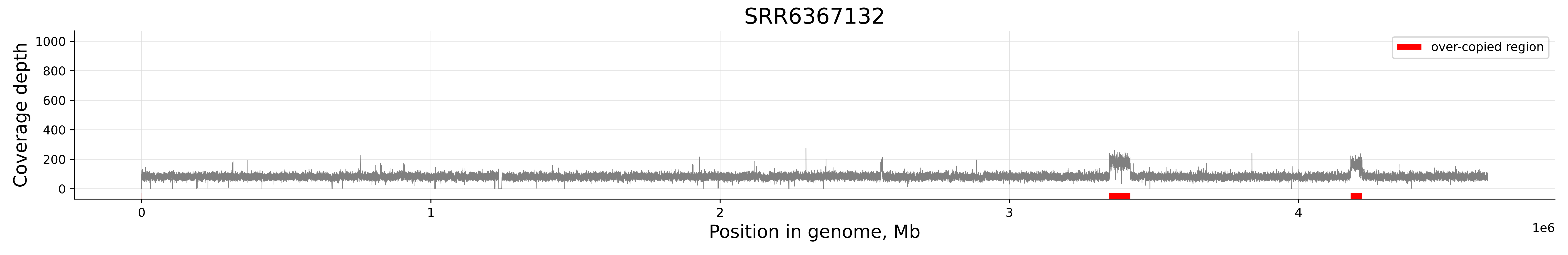
SRR6367132
Coverage depth
1000
800
600
400
200
0
over-copied region
0
1
2
3
4
1e6
Position in genome, Mb

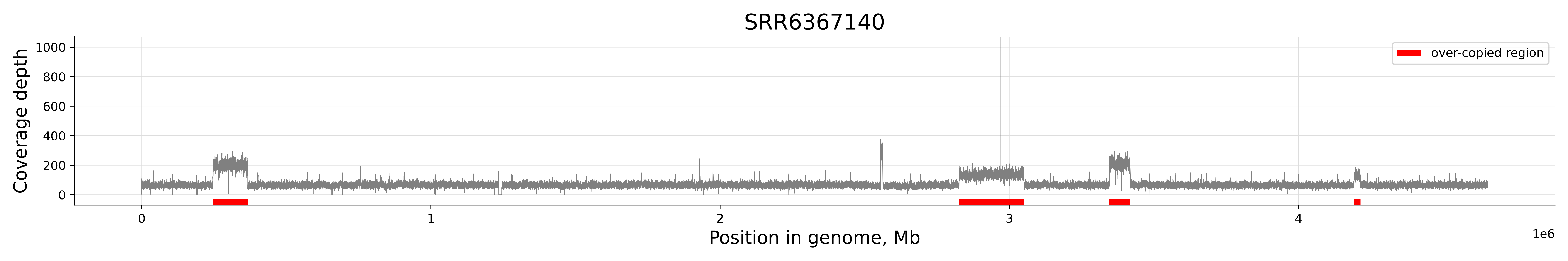
SRR6367140
Coverage depth
1000
800
600
400
200
0
over-copied region
0
1
2
3
4
1e6
Position in genome, Mb

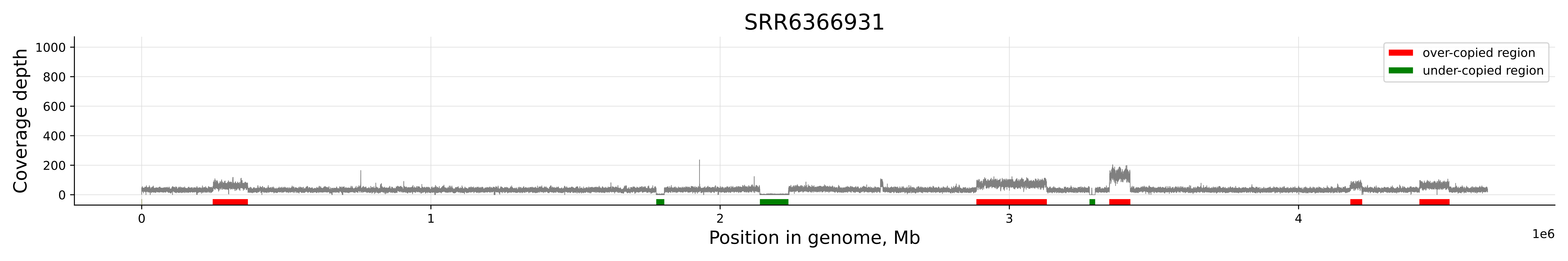
SRR6366931
Coverage depth
1000
800
600
400
200
0
0
1
2
3
4
1e6
Position in genome, Mb
over-copied region
under-copied region

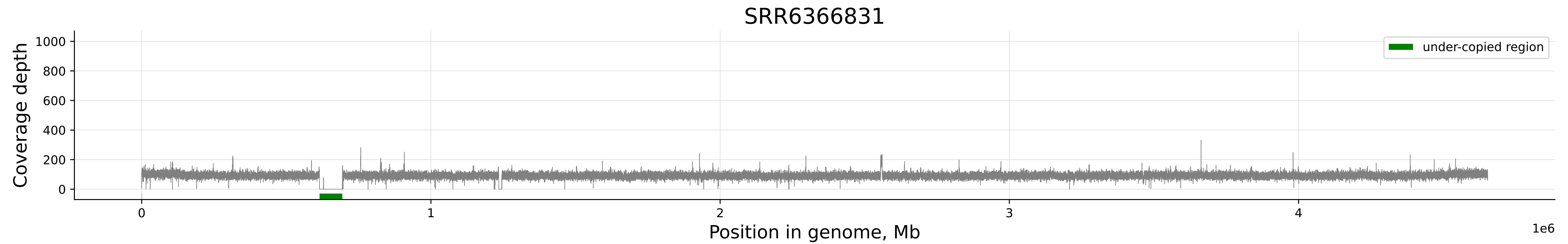
SRR6366831
Coverage depth
Position in genome, Mb
under-copied region
1000
800
600
400
200
0
0
1
2
3
4
1e6

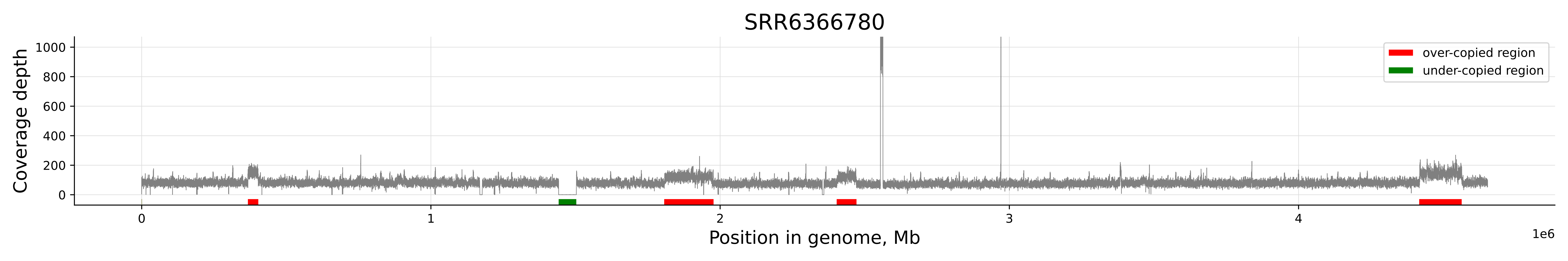
SRR6366780
Coverage depth
Position in genome, Mb
1e6
over-copied region
under-copied region
0
200
400
600
800
1000
0
1
2
3
4

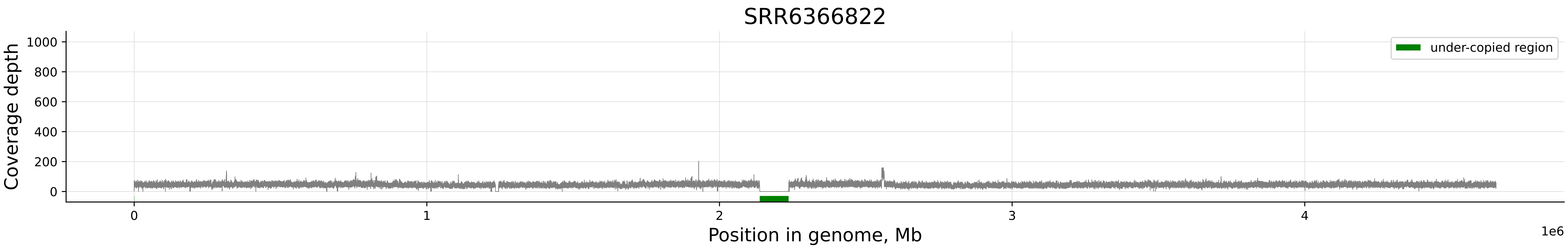

SRR6366822
Coverage depth
1000
800
600
400
200
0
under-copied region
0
1
2
3
4
1e6
Position in genome, Mb

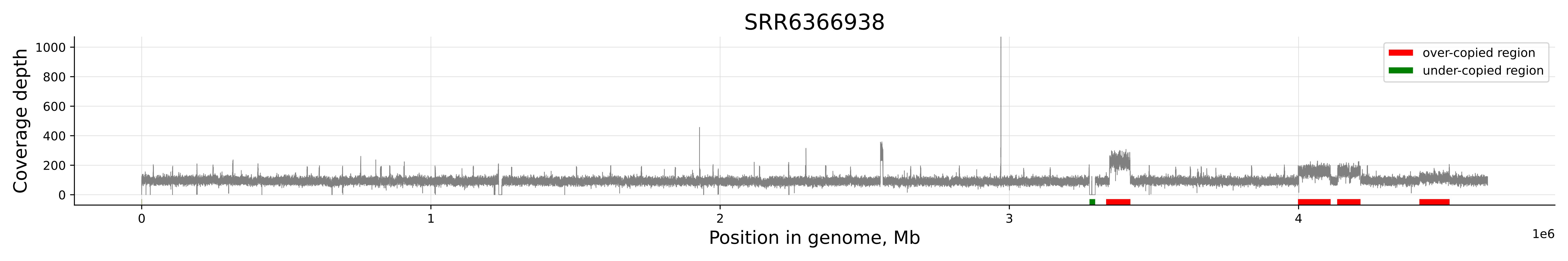
SRR6366938
Coverage depth
Position in genome, Mb
1e6
over-copied region
under-copied region
0
200
400
600
800
1000
0
1
2
3
4

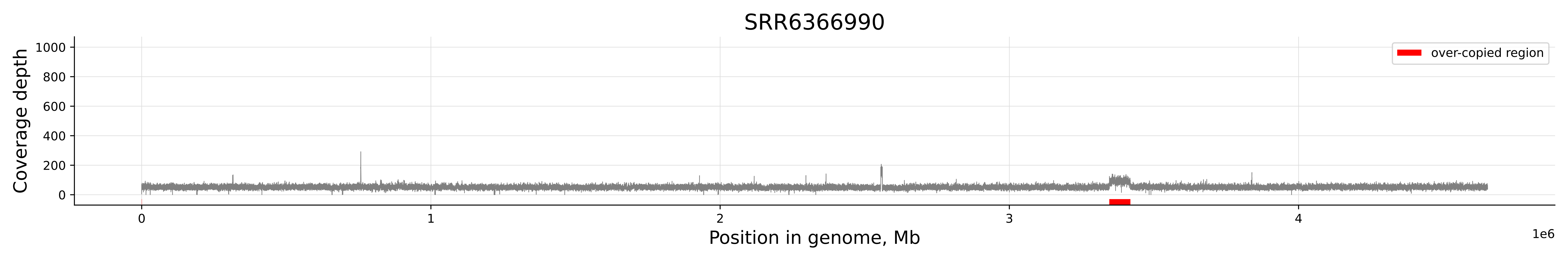

SRR6366990
Coverage depth
Position in genome, Mb
over-copied region
1000
800
600
400
200
0
0
1
2
3
4
1e6

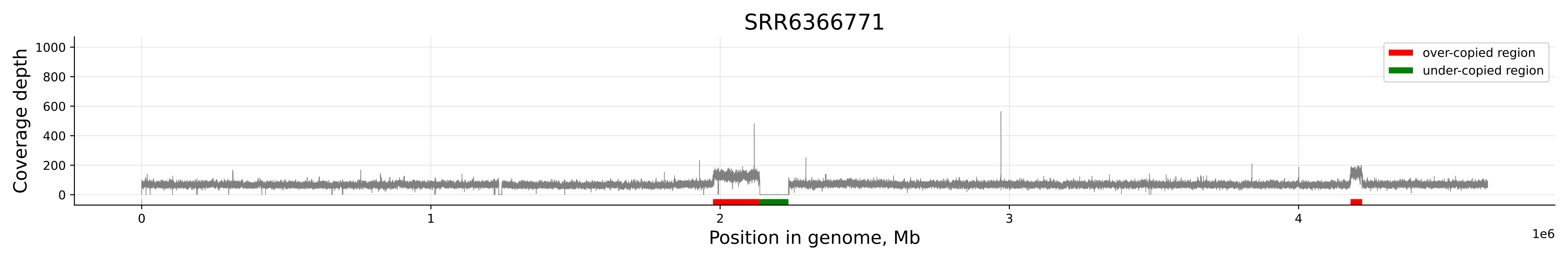
SRR6366771
over-copied region
under-copied region
Coverage depth
1000
800
600
400
200
0
0
1
2
3
4
1e6
Position in genome, Mb

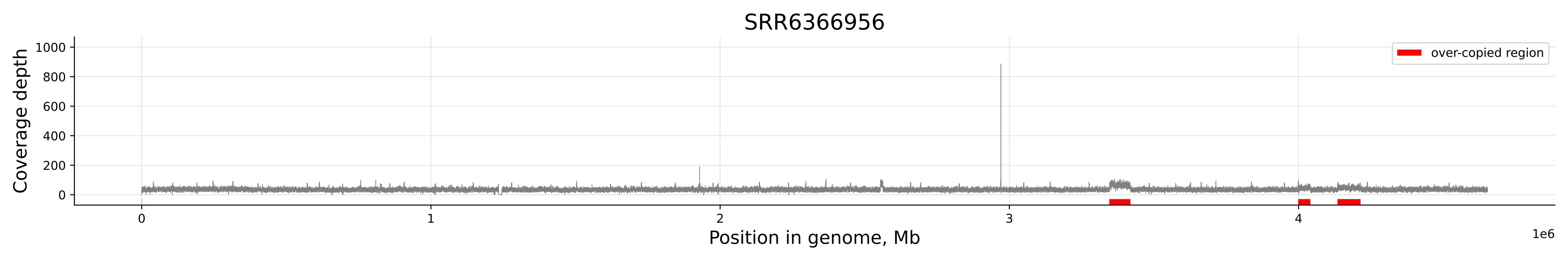

SRR6366956
Coverage depth
1000
800
600
400
200
0
0
1
2
3
4
1e6
Position in genome, Mb
over-copied region

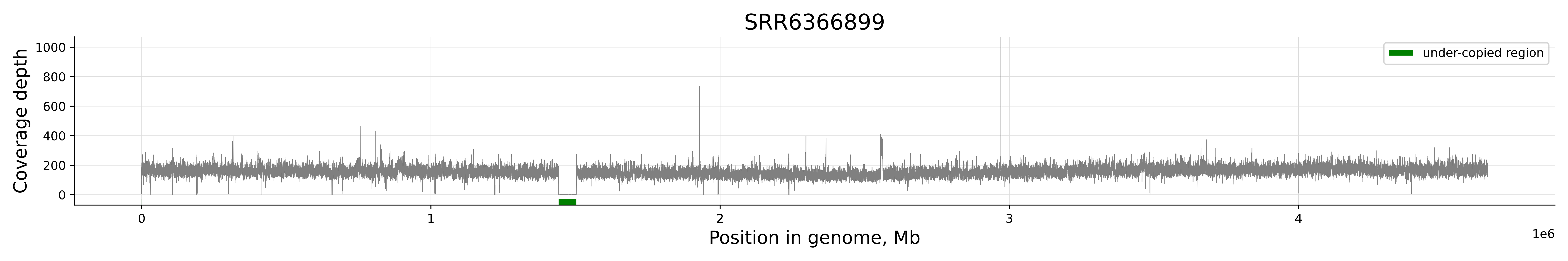

SRR6366899
Coverage depth
1000
800
600
400
200
0
under-copied region
0
1
2
3
4
Position in genome, Mb
1e6

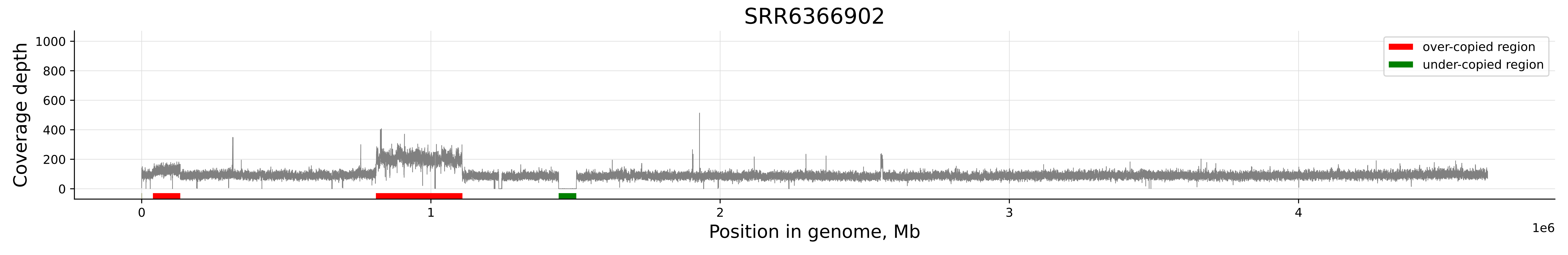
SRR6366902
Coverage depth
1000
800
600
400
200
0
0
1
2
3
4
1e6
Position in genome, Mb
over-copied region
under-copied region

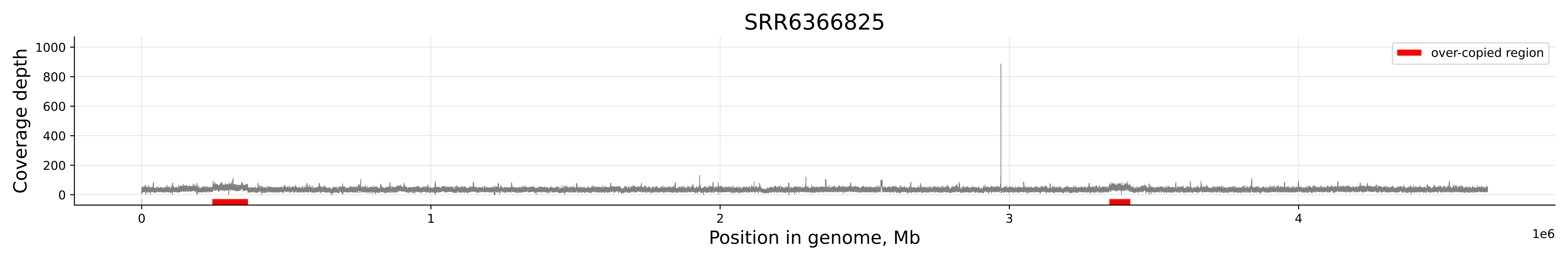
SRR6366825
Coverage depth
1000
800
600
400
200
0
0
1
2
3
4
1e6
Position in genome, Mb
over-copied region

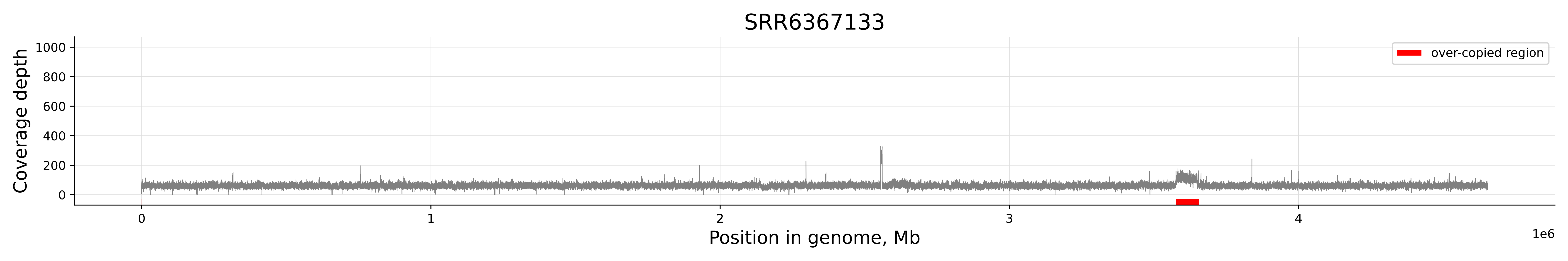
SRR6367133
Coverage depth
Position in genome, Mb
over-copied region
0
200
400
600
800
1000
0
1
2
3
4
1e6

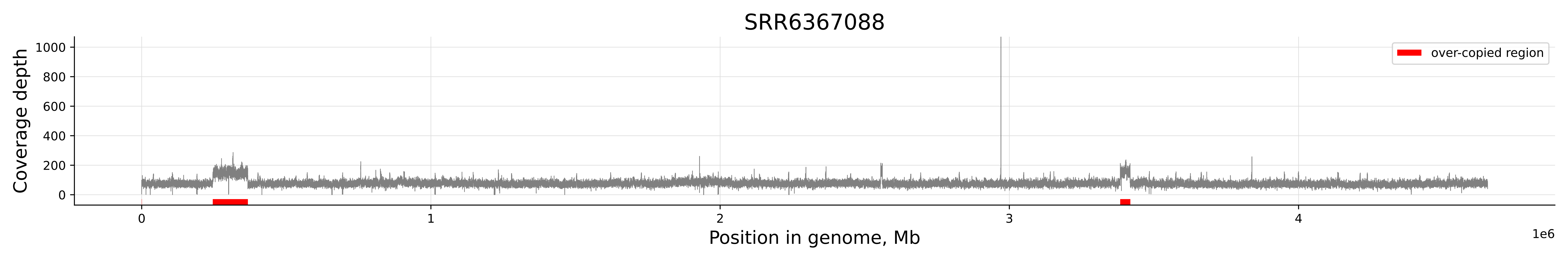

SRR6367088
over-copied region
Coverage depth
0
200
400
600
800
1000
0
1
2
3
4
1e6
Position in genome, Mb

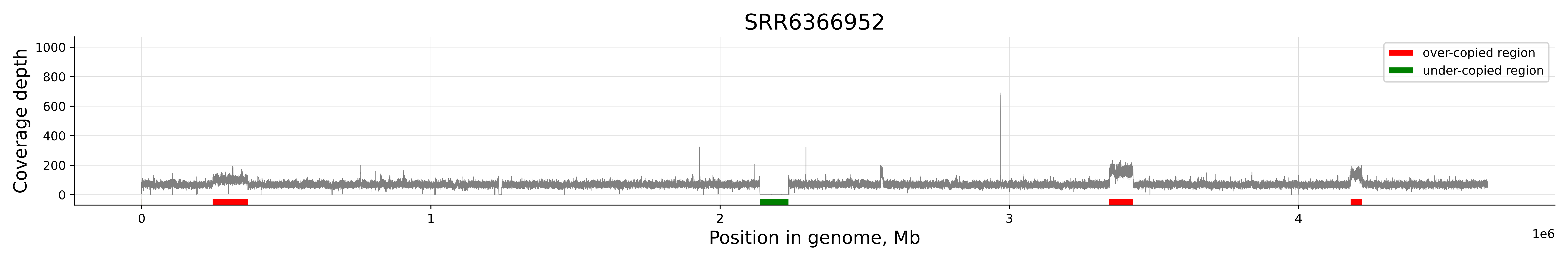

SRR6366952
Coverage depth
Position in genome, Mb
over-copied region
under-copied region
1000
800
600
400
200
0
0
1
2
3
4
1e6

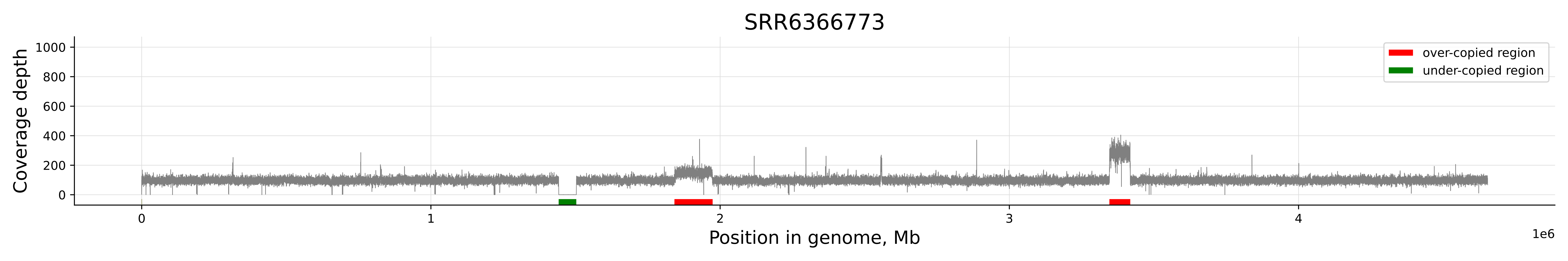
SRR6366773
Coverage depth
1000
800
600
400
200
0
0
1
2
3
4
1e6
Position in genome, Mb
over-copied region
under-copied region

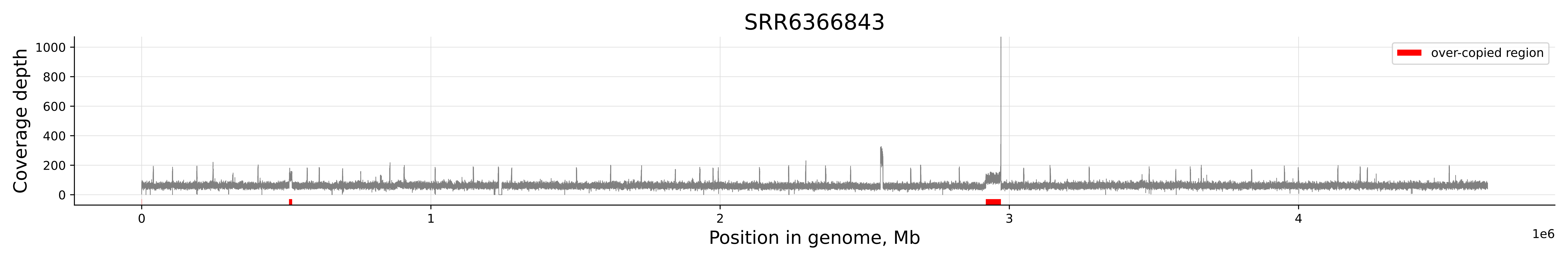
SRR6366843
Coverage depth
Position in genome, Mb
over-copied region
1e6
0
200
400
600
800
1000
0
1
2
3
4

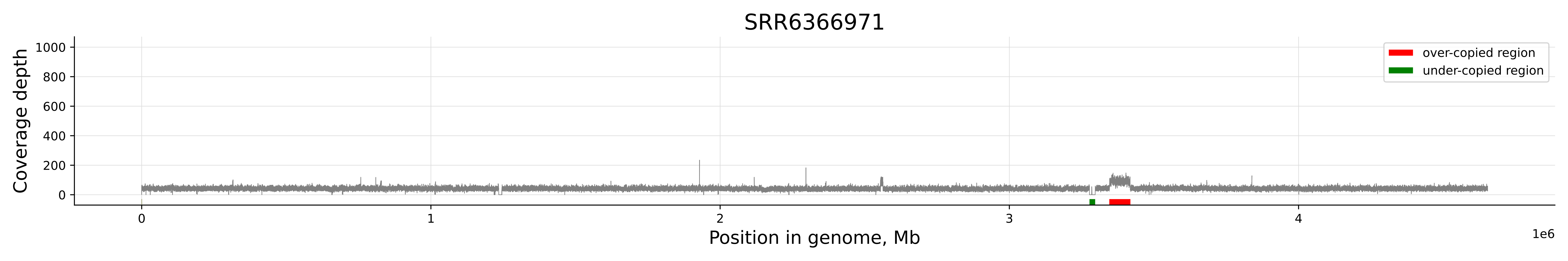
SRR6366971
Coverage depth
1000
800
600
400
200
0
0
1
2
3
4
1e6
Position in genome, Mb
over-copied region
under-copied region

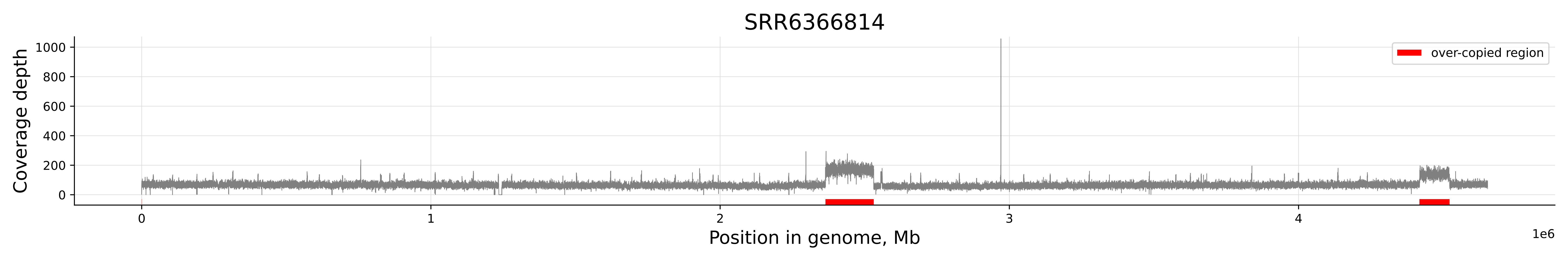
SRR6366814
Coverage depth
Position in genome, Mb
over-copied region
1e6

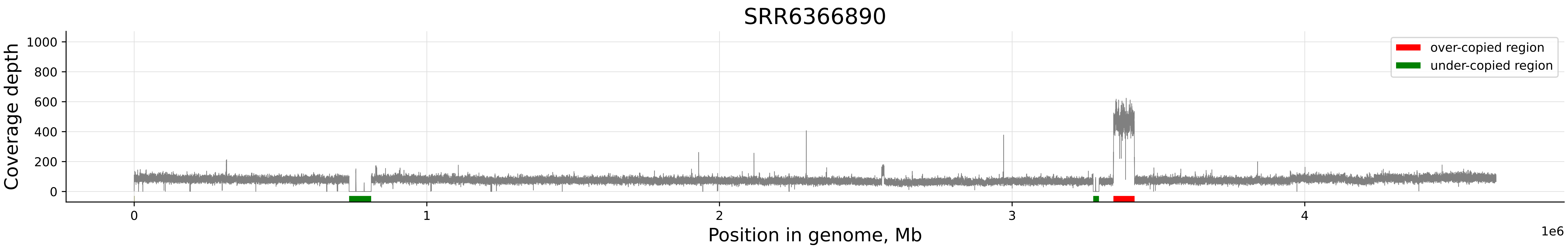

SRR6366890
Coverage depth
Position in genome, Mb
1e6
over-copied region
under-copied region
0
200
400
600
800
1000
0
1
2
3
4

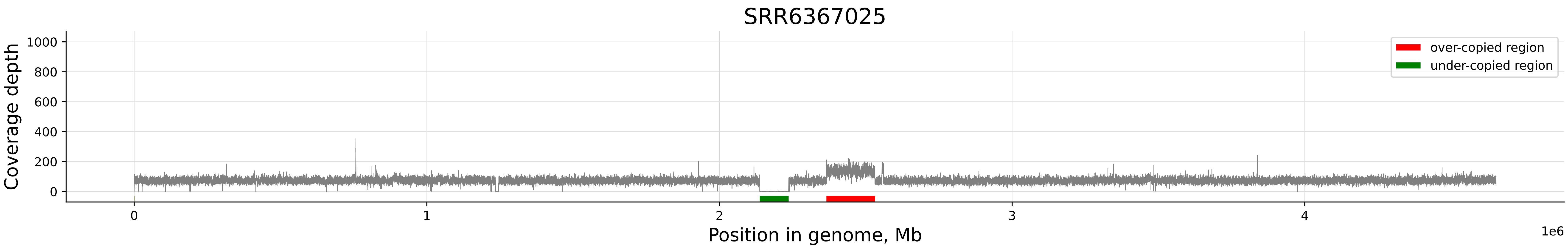
SRR6367025
Coverage depth
1000
800
600
400
200
0
over-copied region
under-copied region
0
1
2
3
4
1e6
Position in genome, Mb

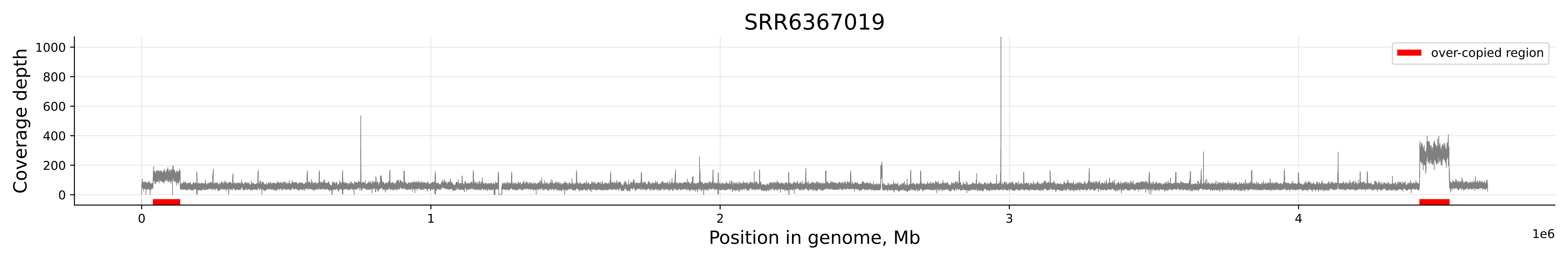
SRR6367019
Coverage depth
1000
800
600
400
200
0
over-copied region
0
1
2
3
4
1e6
Position in genome, Mb

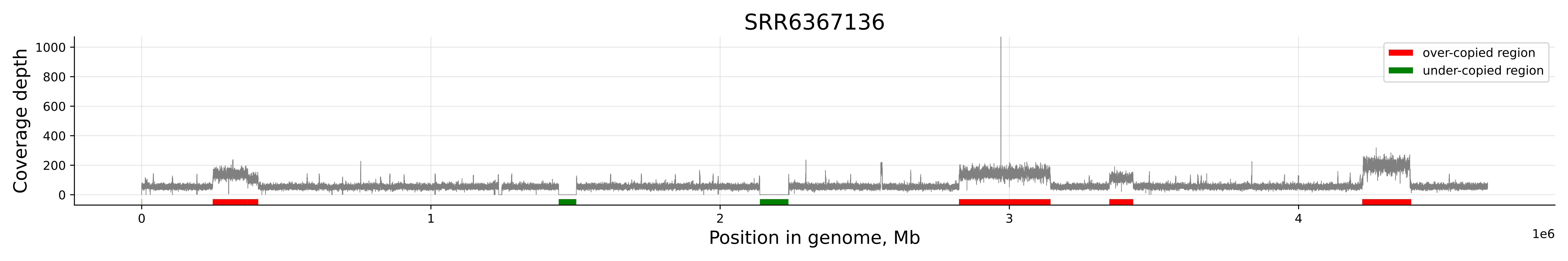
SRR6367136
Coverage depth
1000
800
600
400
200
0
over-copied region
under-copied region
0
1
2
3
4
1e6
Position in genome, Mb

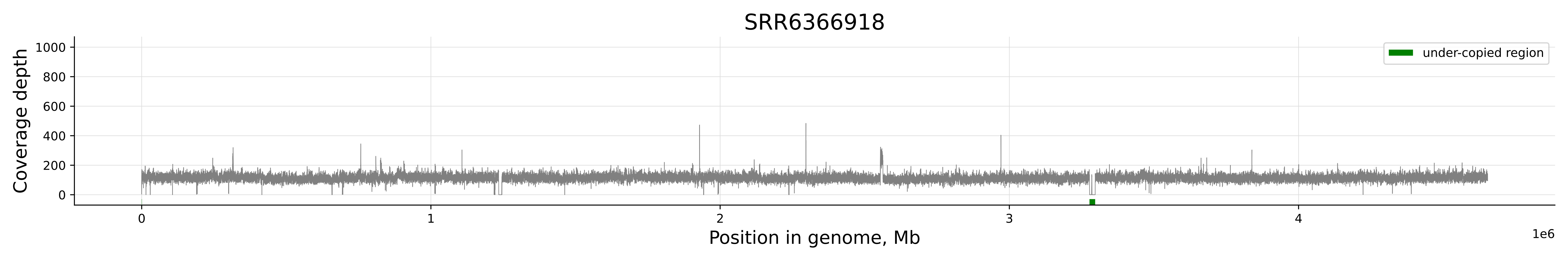

SRR6366918
Coverage depth
1000
800
600
400
200
0
under-copied region
0
1
2
3
4
1e6
Position in genome, Mb

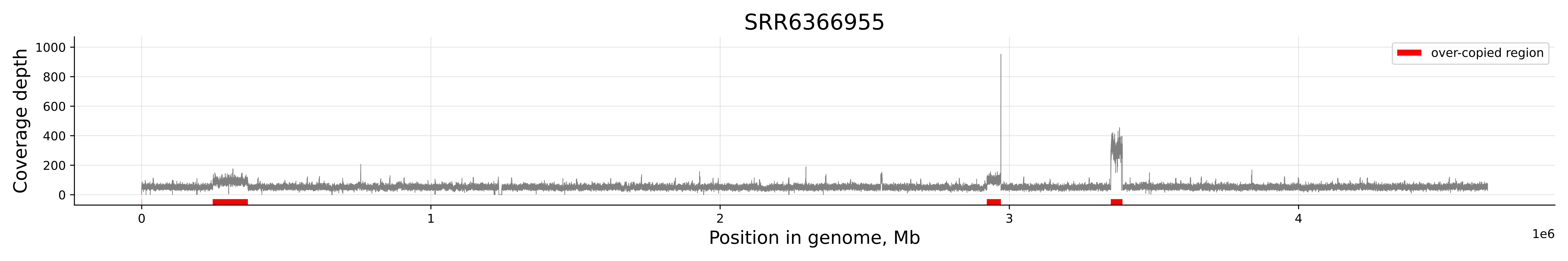
SRR6366955
Coverage depth
1000
800
600
400
200
0
over-copied region
0
1
2
3
4
1e6
Position in genome, Mb

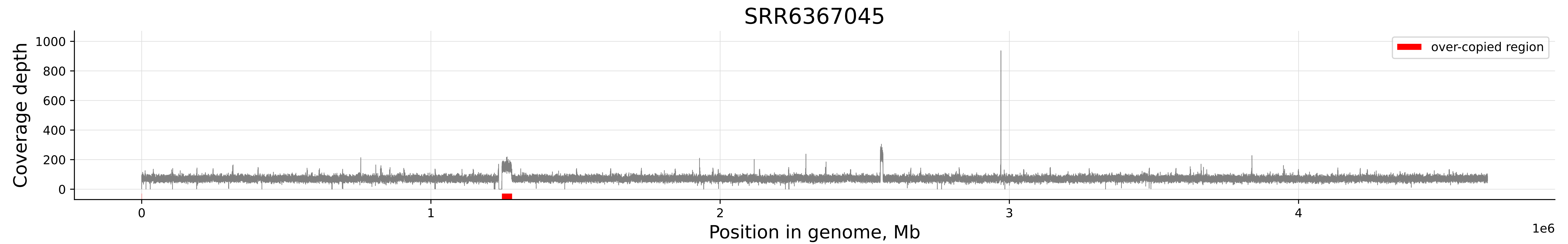

SRR6367045
Coverage depth
1000
800
600
400
200
0
over-copied region
0
1
2
3
4
1e6
Position in genome, Mb

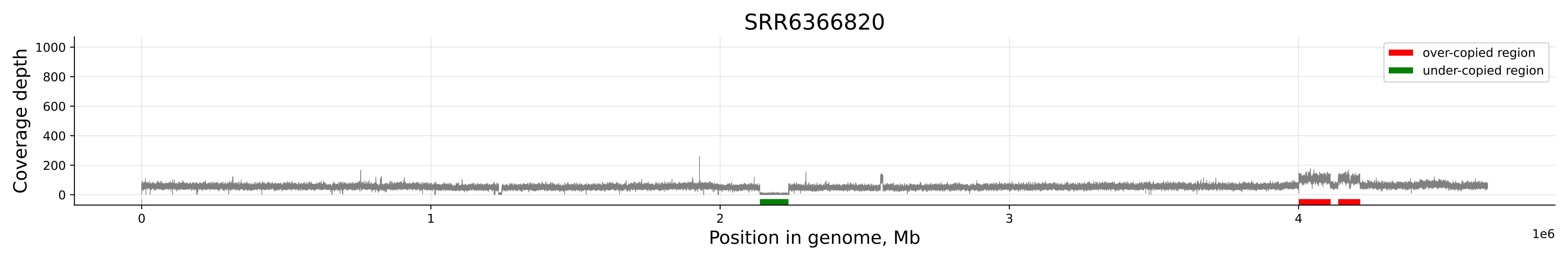
SRR6366820
Coverage depth
1000
800
600
400
200
0
0
1
2
3
4
1e6
Position in genome, Mb
over-copied region
under-copied region

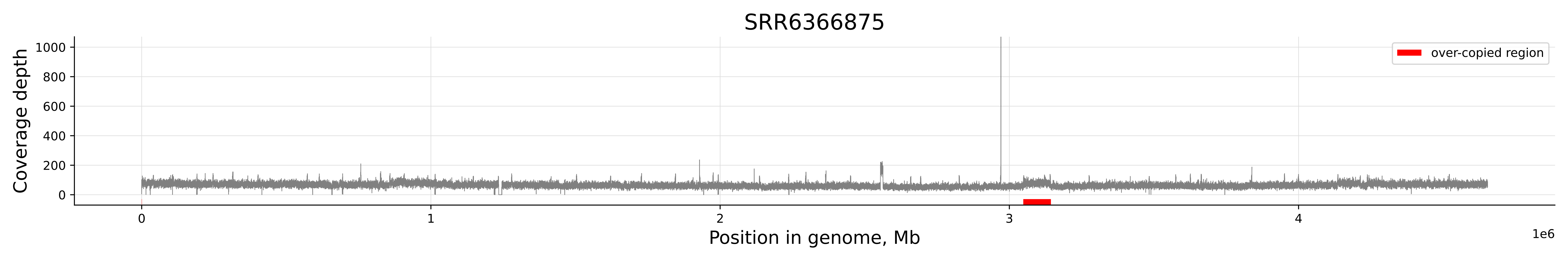
SRR6366875
Coverage depth
0
200
400
600
800
1000
over-copied region
0
1
2
3
4
1e6
Position in genome, Mb

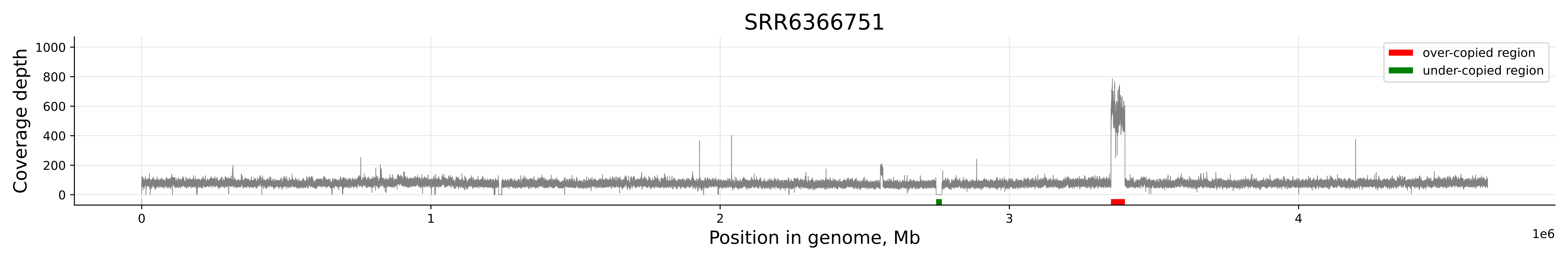

SRR6366751
Coverage depth
1000
800
600
400
200
0
0
1
2
3
4
1e6
Position in genome, Mb
over-copied region
under-copied region

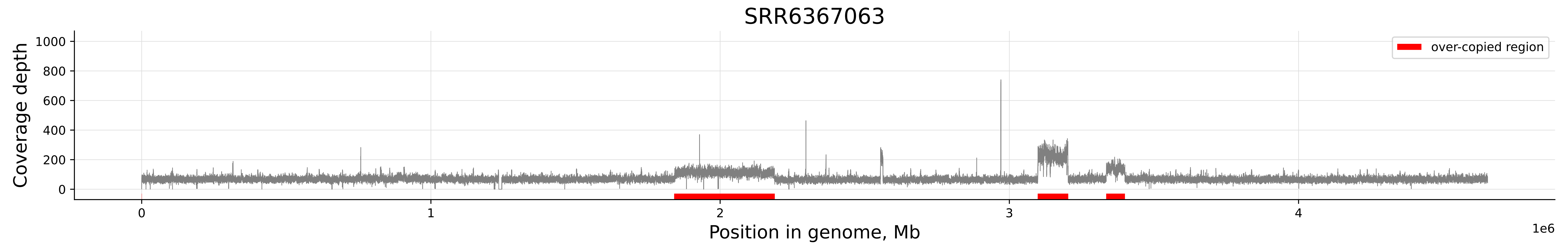
SRR6367063
over-copied region
Coverage depth
1000
800
600
400
200
0
0
1
2
3
4
1e6
Position in genome, Mb

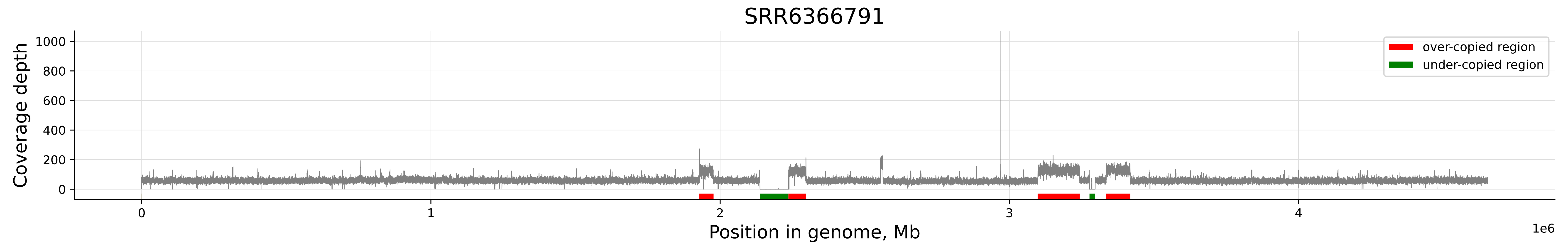
SRR6366791
Coverage depth
Position in genome, Mb
1e6
over-copied region
under-copied region
0
200
400
600
800
1000
0
1
2
3
4

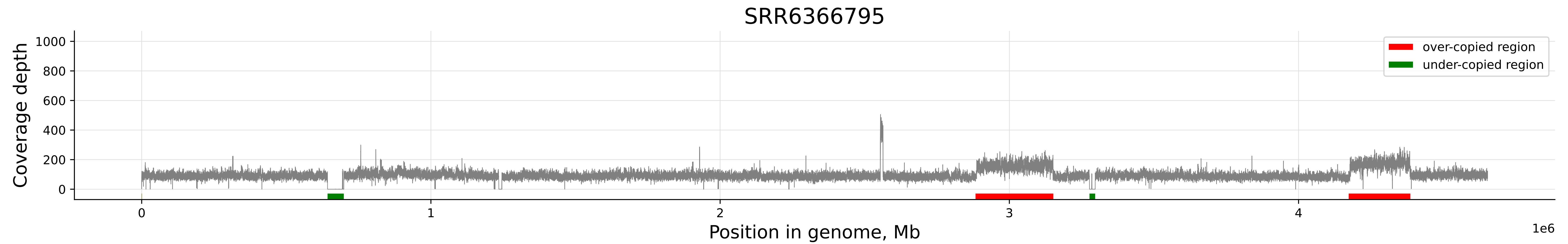
SRR6366795
Coverage depth
1000
800
600
400
200
0
0
1
2
3
4
1e6
Position in genome, Mb
over-copied region
under-copied region

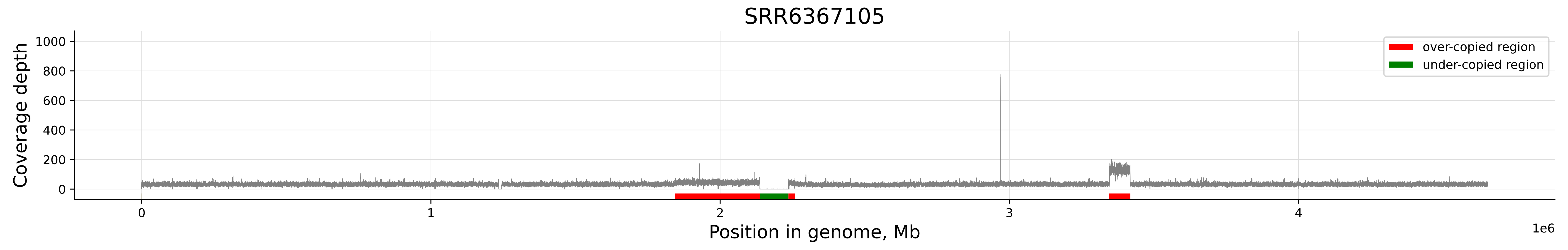

SRR6367105
Coverage depth
Position in genome, Mb
over-copied region
under-copied region
1000
800
600
400
200
0
0
1
2
3
4
1e6

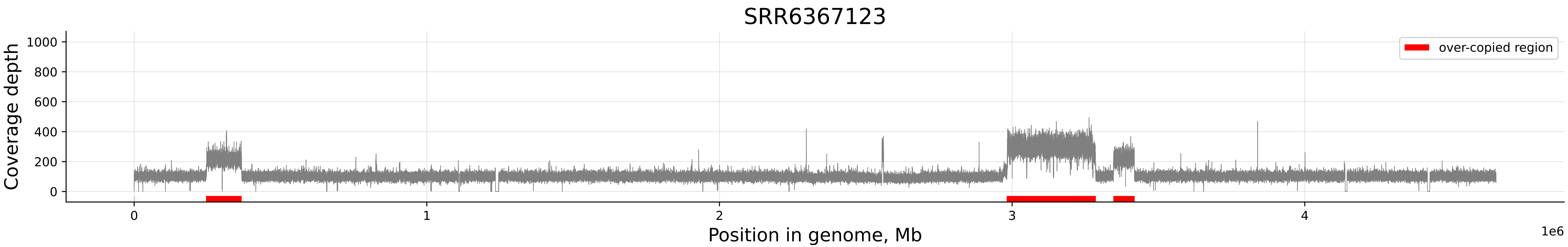

SRR6367123
Coverage depth
1000
800
600
400
200
0
over-copied region
0
1
2
3
4
1e6
Position in genome, Mb

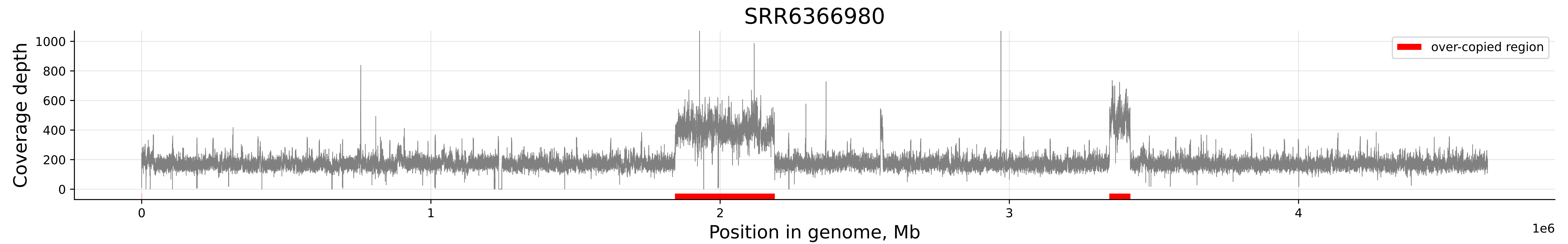
SRR6366980
over-copied region
Coverage depth
0
200
400
600
800
1000
0
1
2
3
4
1e6
Position in genome, Mb

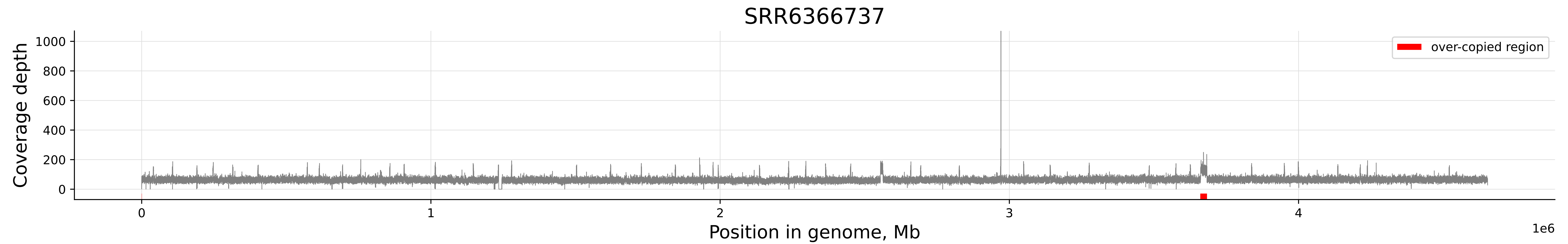
SRR6366737
Coverage depth
Position in genome, Mb
over-copied region
1000
800
600
400
200
0
1
2
3
4
1e6

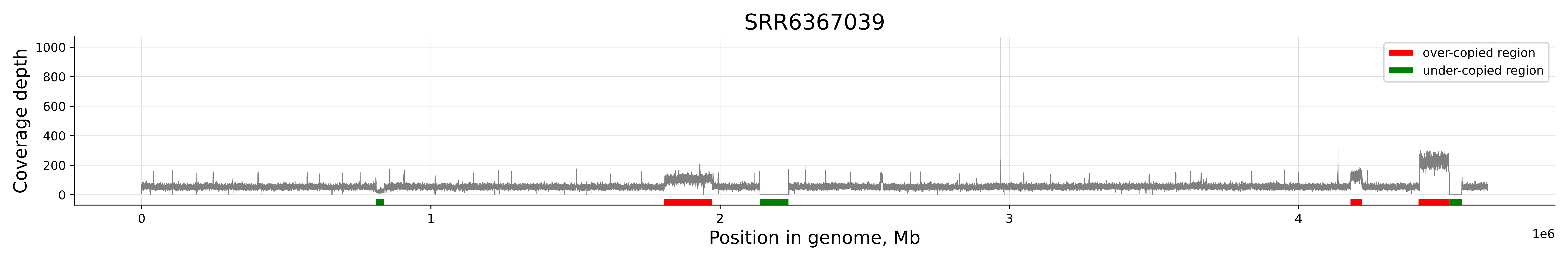

SRR6367039
over-copied region
under-copied region
Coverage depth
0
200
400
600
800
1000
0
1
2
3
4
1e6
Position in genome, Mb

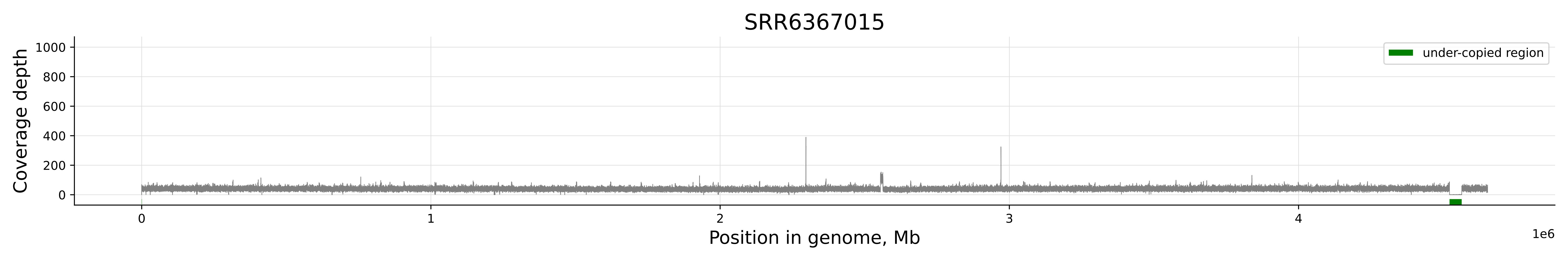

SRR6367015
Coverage depth
1000
800
600
400
200
0
under-copied region
0
1
2
3
4
1e6
Position in genome, Mb

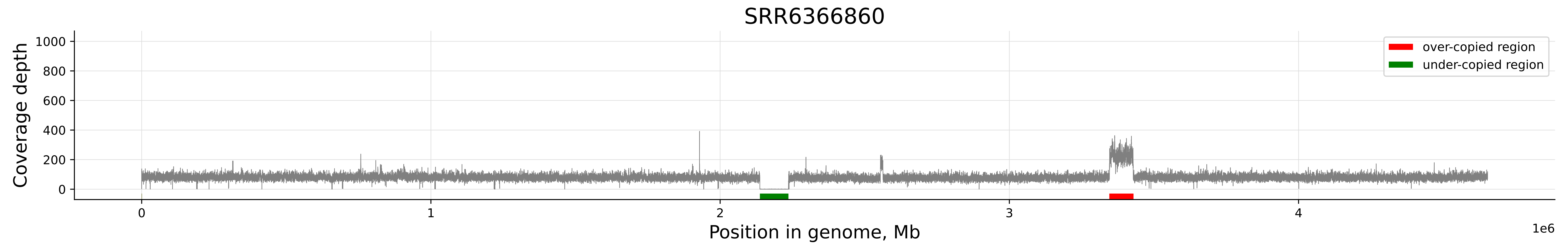
SRR6366860
Coverage depth
1000
800
600
400
200
0
0
1
2
3
4
1e6
Position in genome, Mb
over-copied region
under-copied region

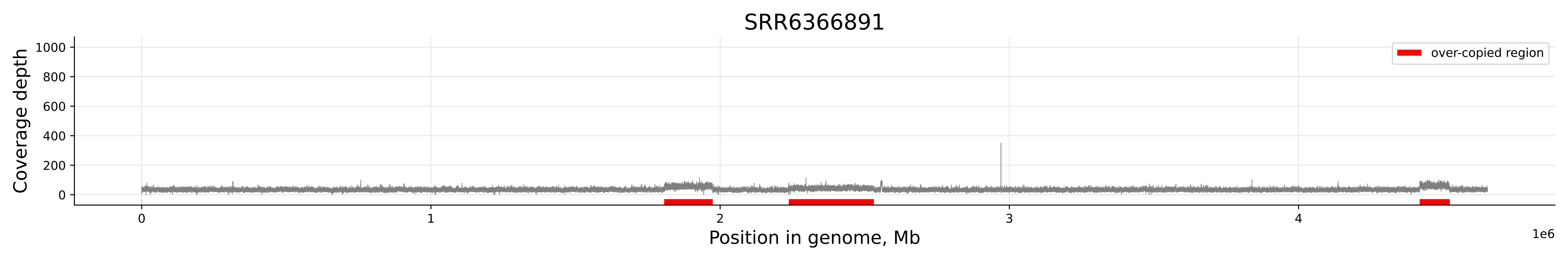

SRR6366891
Coverage depth
1000
800
600
400
200
0
over-copied region
0
1
2
3
4
1e6
Position in genome, Mb

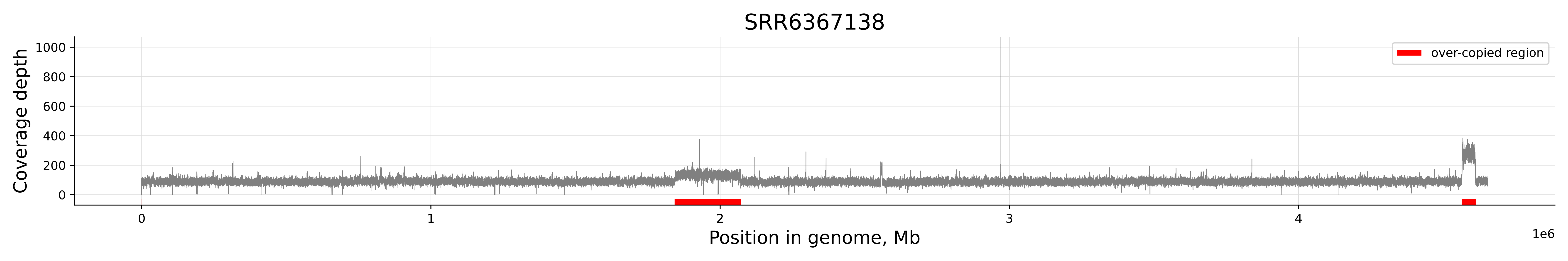

SRR6367138
over-copied region
Coverage depth
1000
800
600
400
200
0
0
1
2
3
4
1e6
Position in genome, Mb

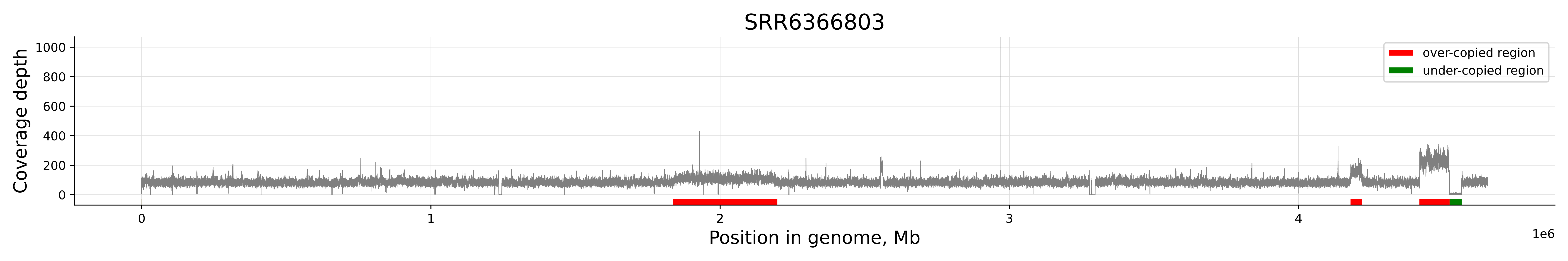

SRR6366803
Coverage depth
1000
800
600
400
200
0
over-copied region
under-copied region
0
1
2
3
4
Position in genome, Mb
1e6

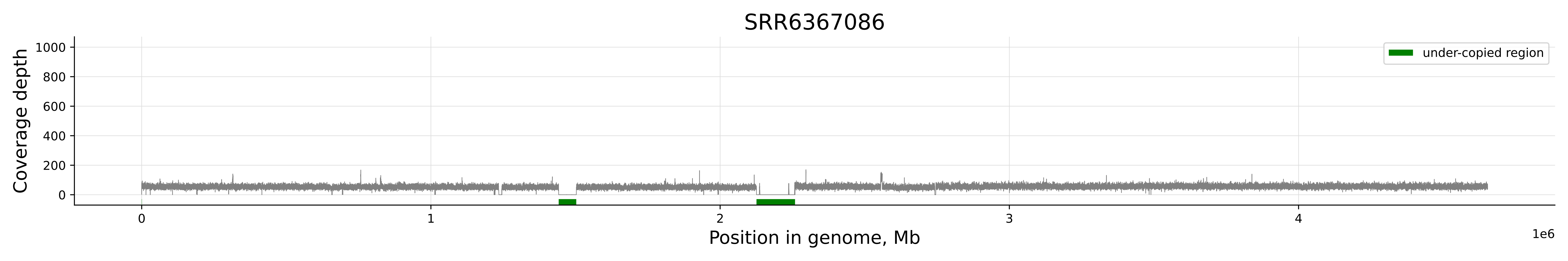

SRR6367086
Coverage depth
Position in genome, Mb
1e6
under-copied region
0
200
400
600
800
1000
0
1
2
3
4

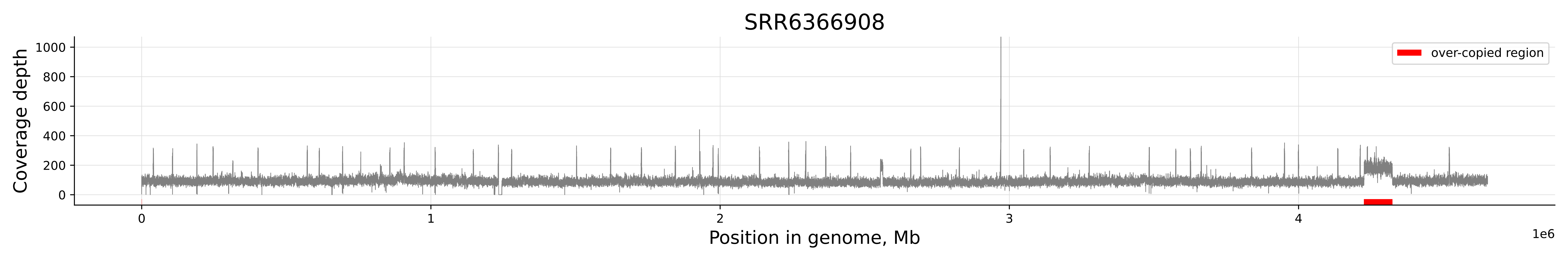
SRR6366908
Coverage depth
1000
800
600
400
200
0
over-copied region
0
1
2
3
4
1e6
Position in genome, Mb

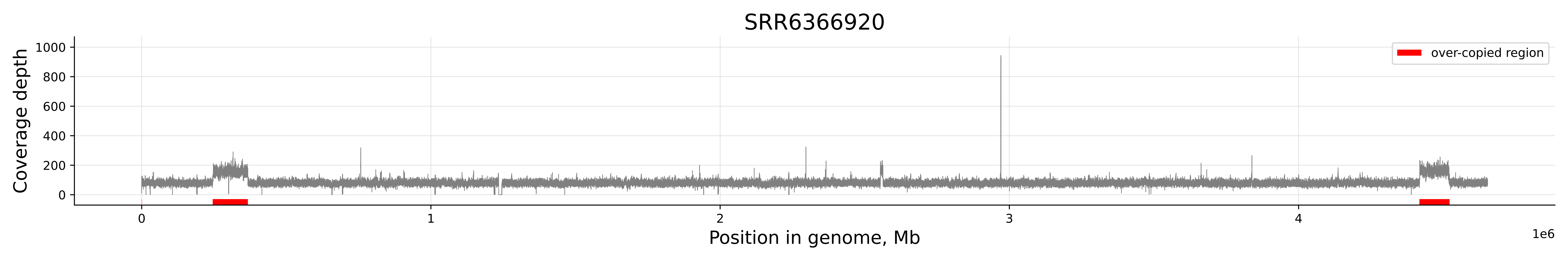
SRR6366920
over-copied region
Coverage depth
1000
800
600
400
200
0
0
1
2
3
4
1e6
Position in genome, Mb

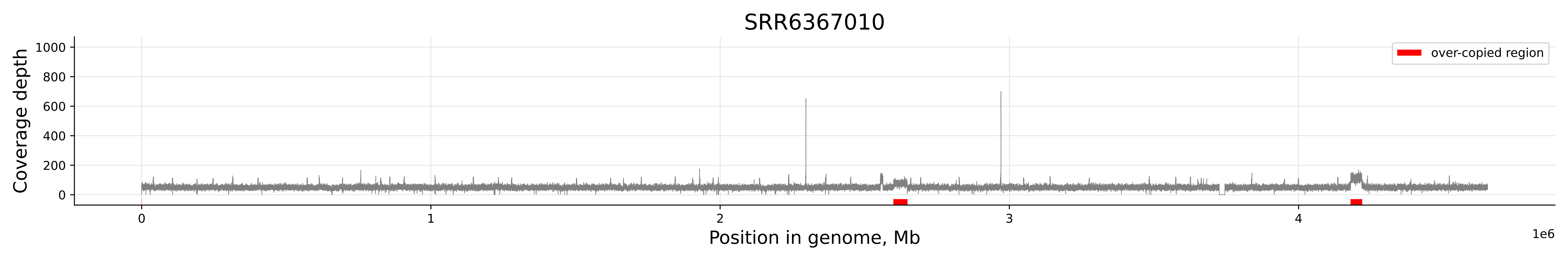

SRR6367010
Coverage depth
1000
800
600
400
200
0
over-copied region
0
1
2
3
4
1e6
Position in genome, Mb

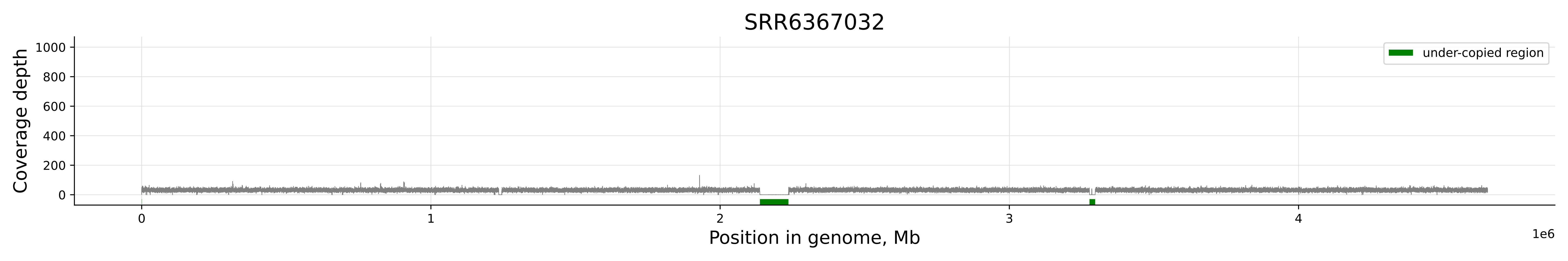

SRR6367032
Coverage depth
1000
800
600
400
200
0
0
1
2
3
4
1e6
Position in genome, Mb
under-copied region

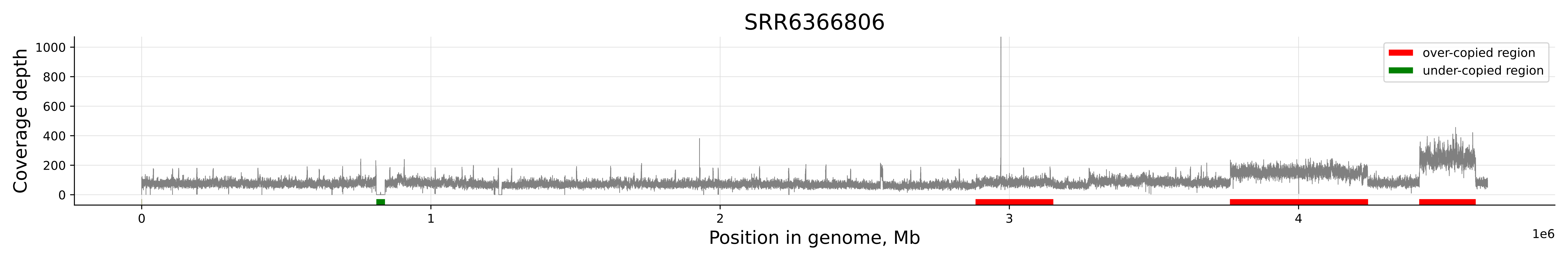

SRR6366806
Coverage depth
1000
800
600
400
200
0
over-copied region
under-copied region
0
1
2
3
4
1e6
Position in genome, Mb

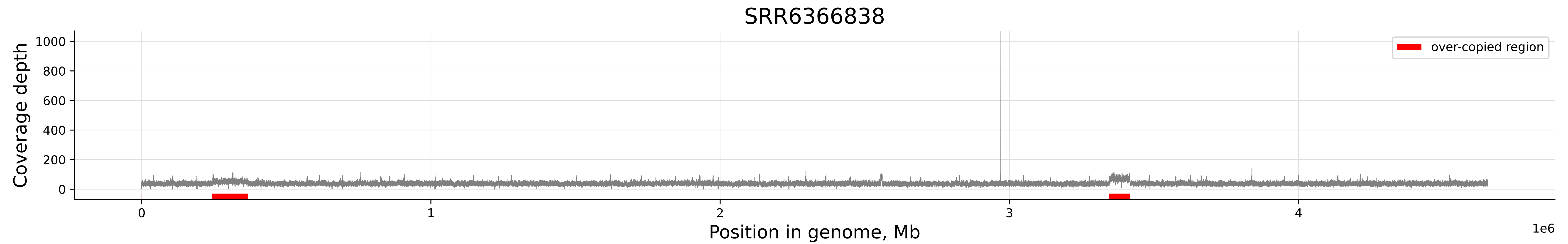
SRR6366838
Coverage depth
Position in genome, Mb
over-copied region
0
200
400
600
800
1000
0
1
2
3
4
1e6

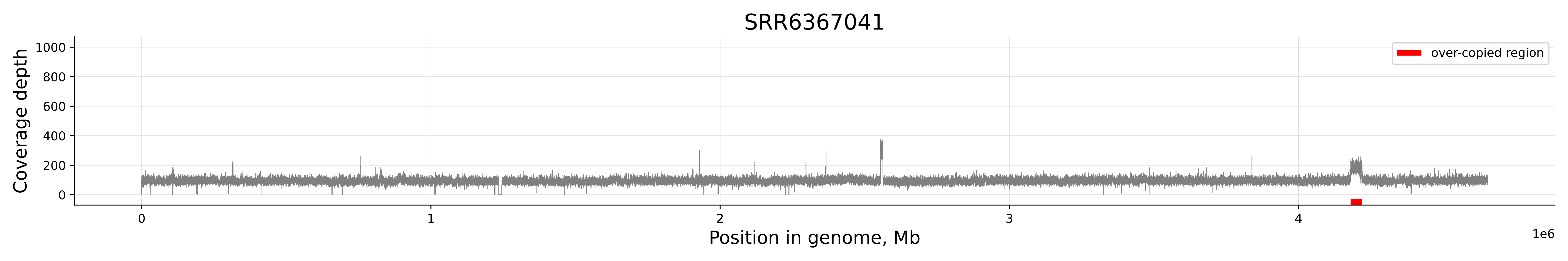
SRR6367041
Coverage depth
0
200
400
600
800
1000
over-copied region
0
1
2
3
4
1e6
Position in genome, Mb

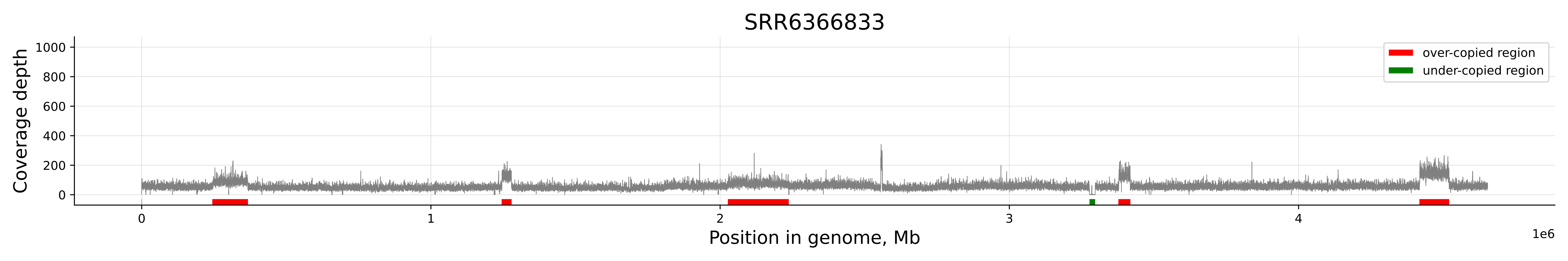

SRR6366833
over-copied region
under-copied region
Coverage depth
1000
800
600
400
200
0
0
1
2
3
4
1e6
Position in genome, Mb

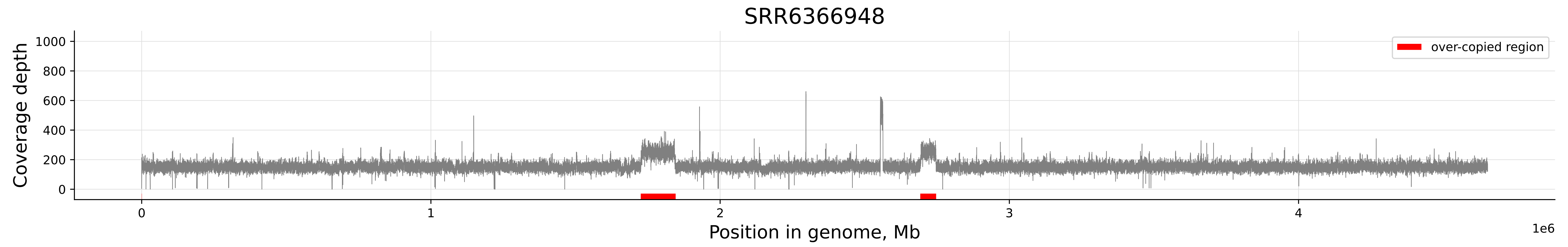
SRR6366948
Coverage depth
Position in genome, Mb
over-copied region
1e6
0
200
400
600
800
1000
0
1
2
3
4

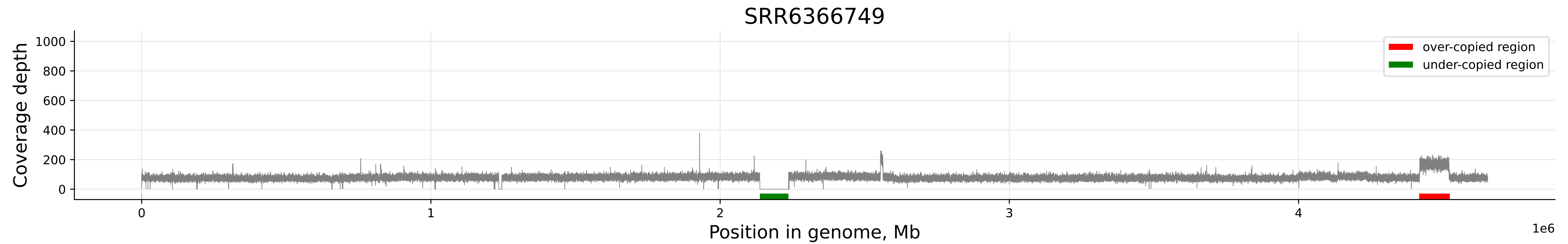
SRR6366749
Coverage depth
1000
800
600
400
200
0
0
1
2
3
4
1e6
Position in genome, Mb
over-copied region
under-copied region

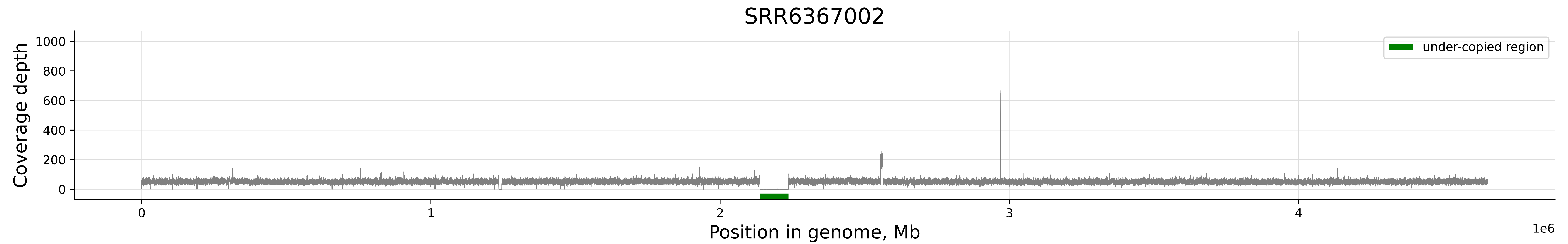

SRR6367002
Coverage depth
Position in genome, Mb
under-copied region
1000
800
600
400
200
0
1
2
3
4
1e6

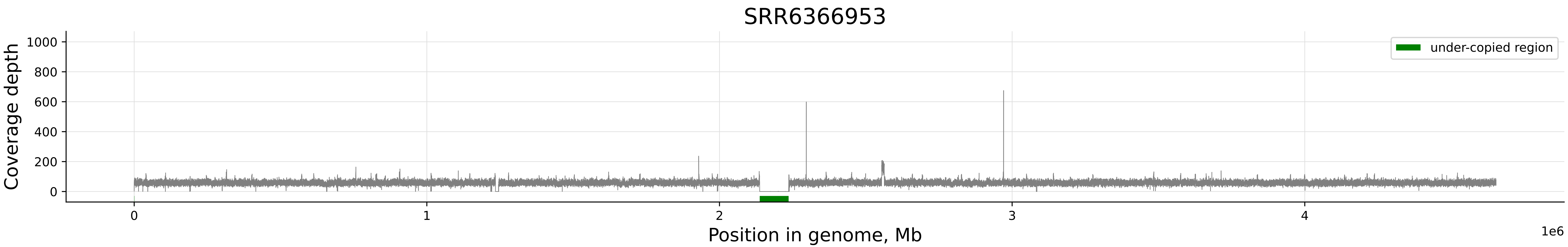
SRR6366953
Coverage depth
1000
800
600
400
200
0
Position in genome, Mb
0
1
2
3
4
1e6
under-copied region

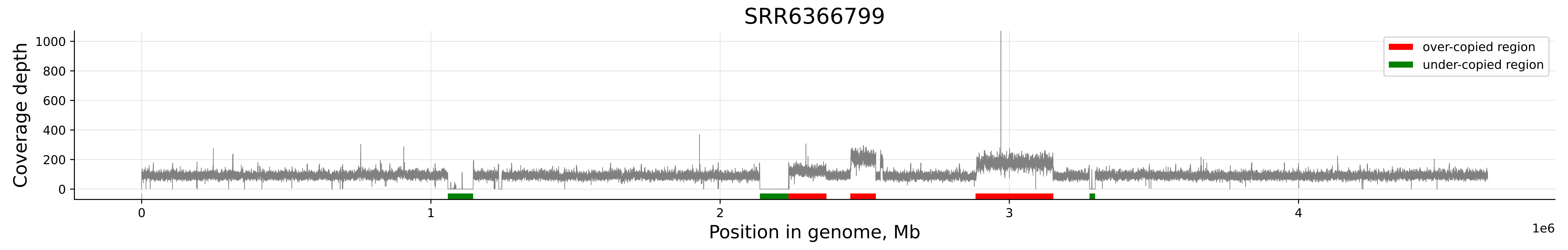

SRR6366799
Coverage depth
1000
800
600
400
200
0
over-copied region
under-copied region
0
1
2
3
4
1e6
Position in genome, Mb

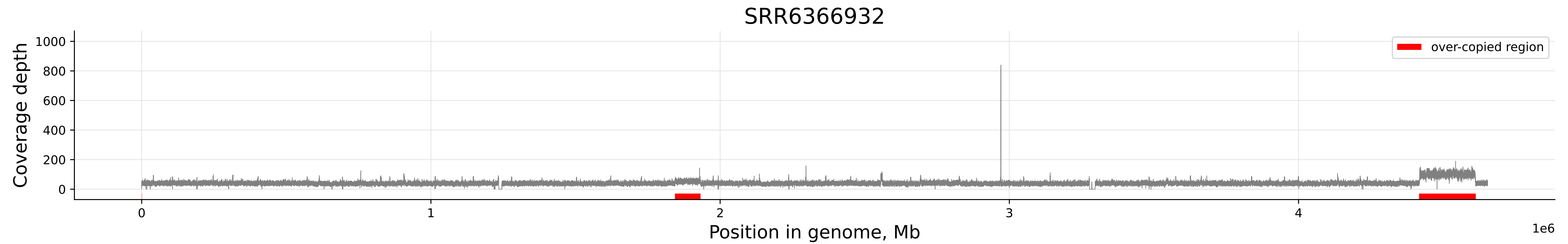
SRR6366932
Coverage depth
Position in genome, Mb
over-copied region
1000
800
600
400
200
0
0
1
2
3
4
1e6

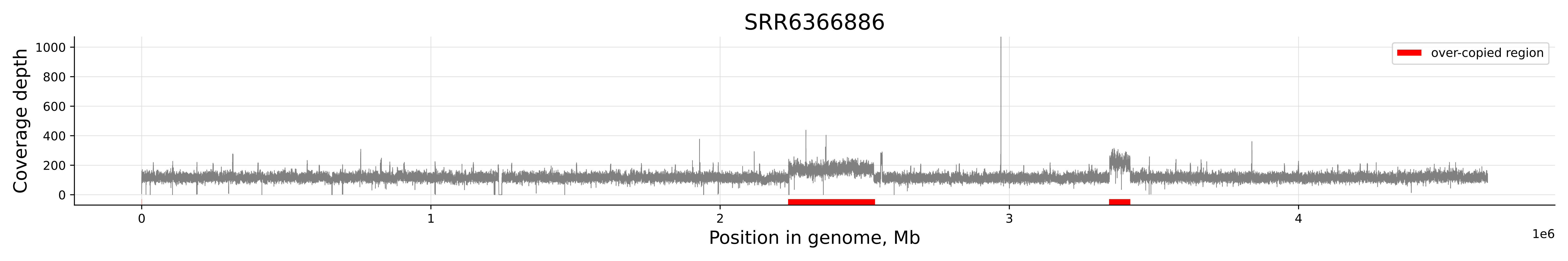

SRR6366886
Coverage depth
Position in genome, Mb
over-copied region
1e6
0
200
400
600
800
1000
0
1
2
3
4

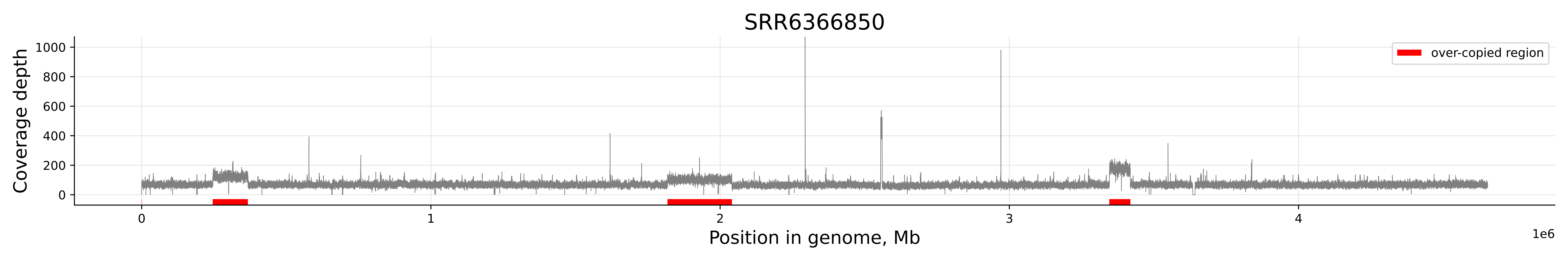
SRR6366850
Coverage depth
Position in genome, Mb
over-copied region
1e6
0
200
400
600
800
1000
1
2
3
4

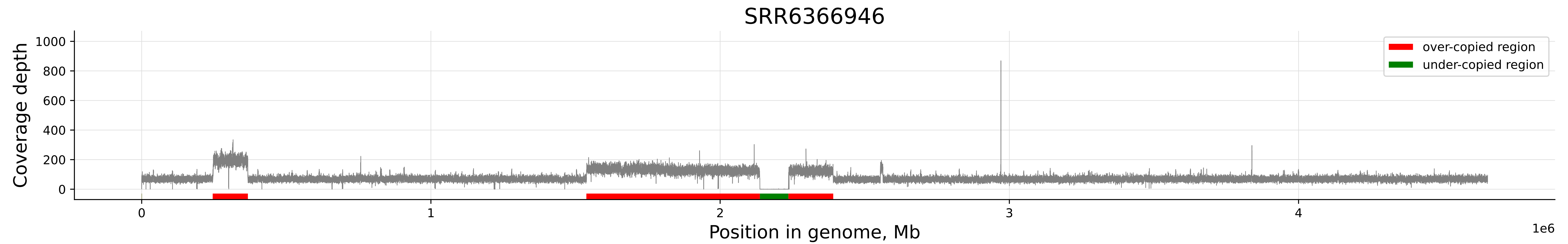

SRR6366946
Coverage depth
Position in genome, Mb
over-copied region
under-copied region
1000
800
600
400
200
0
0
1
2
3
4
1e6

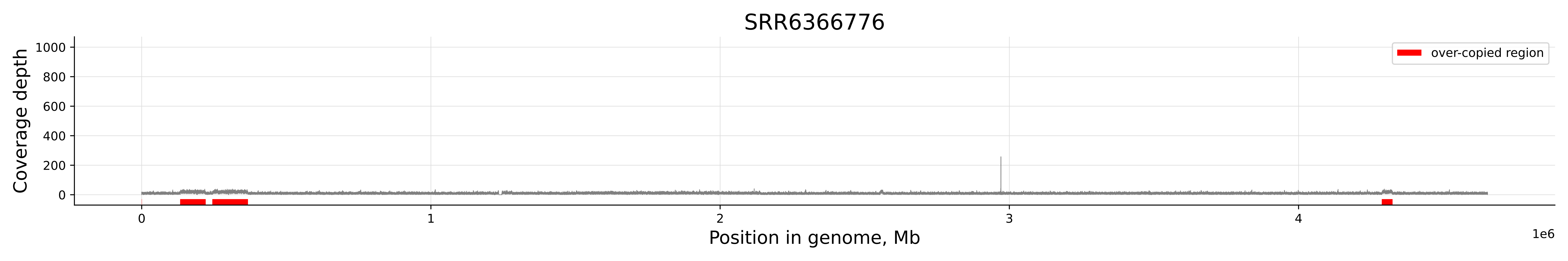
SRR6366776
Coverage depth
1000
800
600
400
200
0
over-copied region
0
1
2
3
4
1e6
Position in genome, Mb

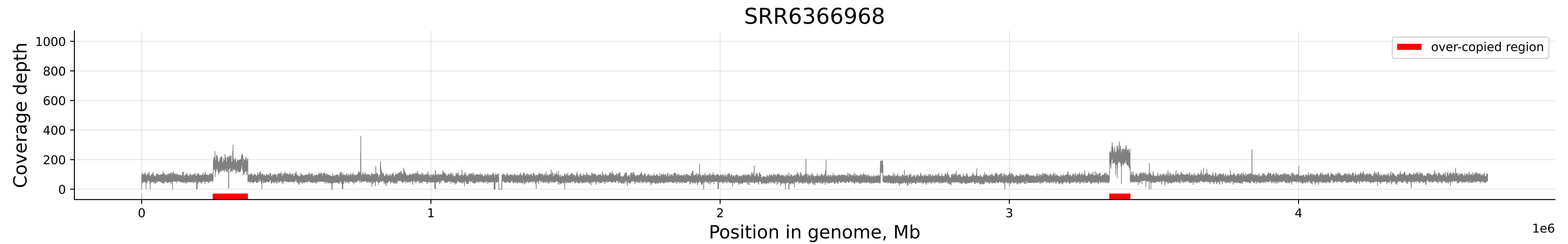
SRR6366968
Coverage depth
1000
800
600
400
200
0
over-copied region
0
1
2
3
4
1e6
Position in genome, Mb

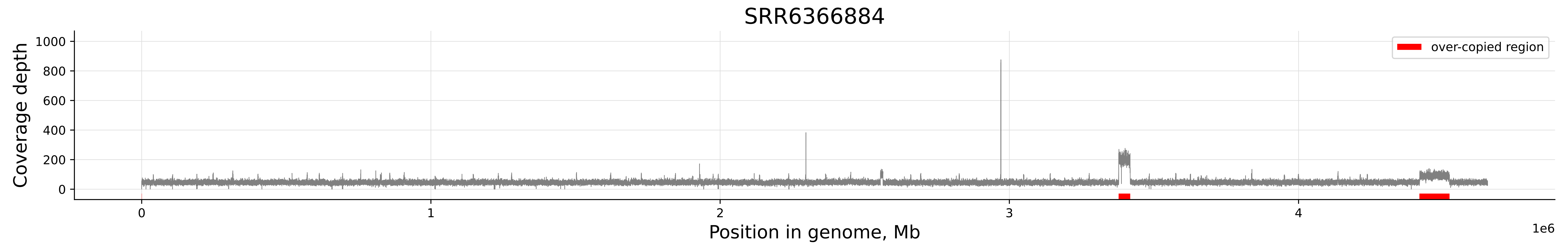

SRR6366884
Coverage depth
Position in genome, Mb
over-copied region
1000
800
600
400
200
0
1
2
3
4
1e6

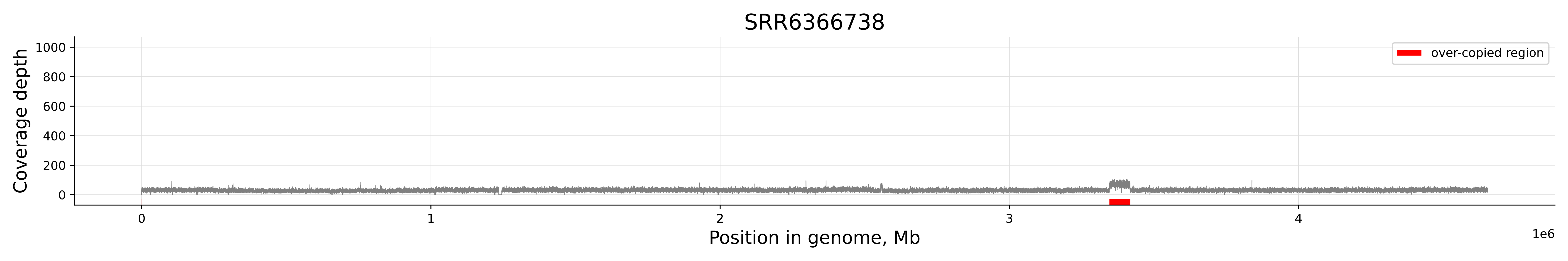

SRR6366738
over-copied region
Coverage depth
1000
800
600
400
200
0
0
1
2
3
4
1e6
Position in genome, Mb

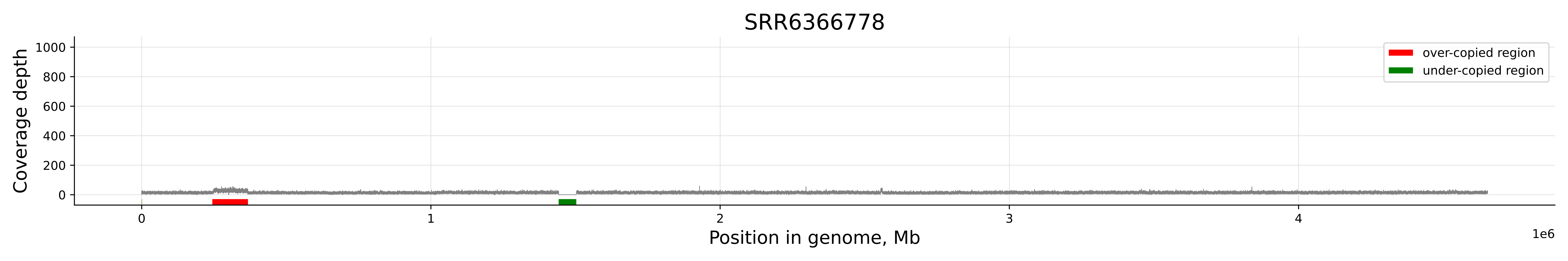
SRR6366778
Coverage depth
1000
800
600
400
200
0
0
1
2
3
4
1e6
Position in genome, Mb
over-copied region
under-copied region

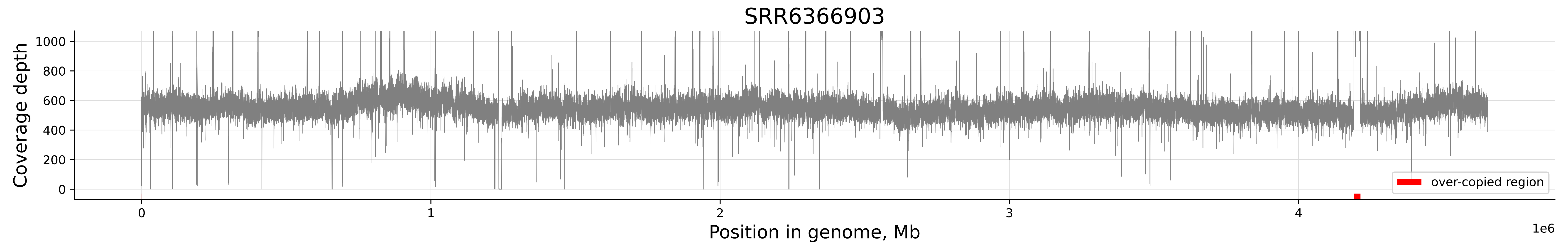
SRR6366903
Coverage depth
1000
800
600
400
200
0
0
1
2
3
4
1e6
Position in genome, Mb
over-copied region

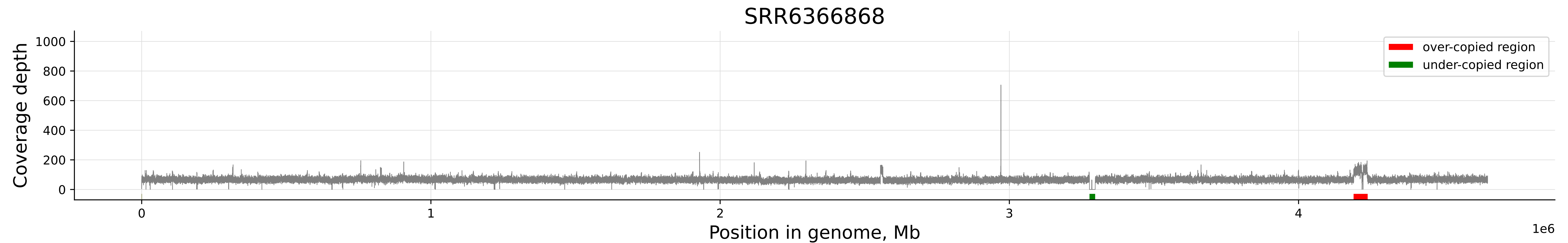
SRR6366868
Coverage depth
1000
800
600
400
200
0
0
1
2
3
4
1e6
Position in genome, Mb
over-copied region
under-copied region

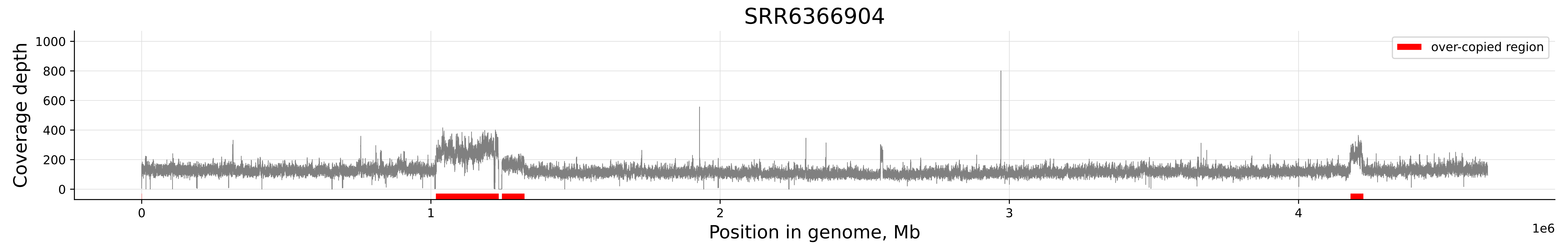
SRR6366904
Coverage depth
Position in genome, Mb
over-copied region
1e6
0
200
400
600
800
1000
0
1
2
3
4

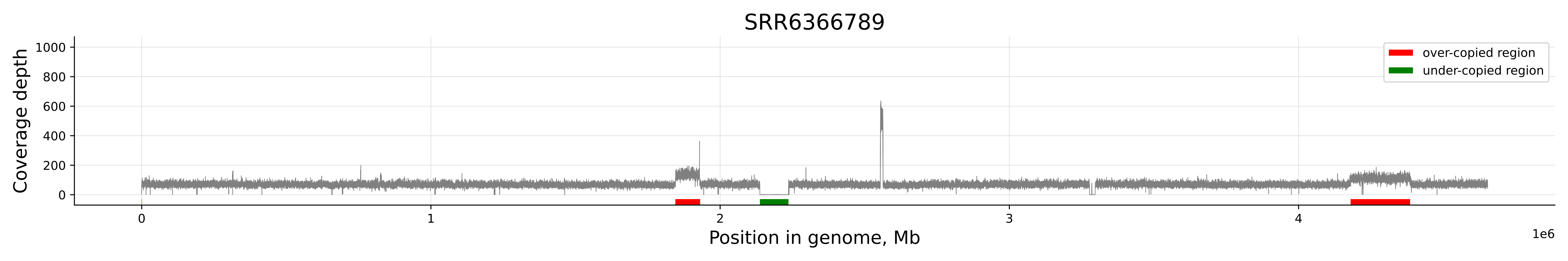
SRR6366789
Coverage depth
0
200
400
600
800
1000
0
1
2
3
4
1e6
Position in genome, Mb
over-copied region
under-copied region

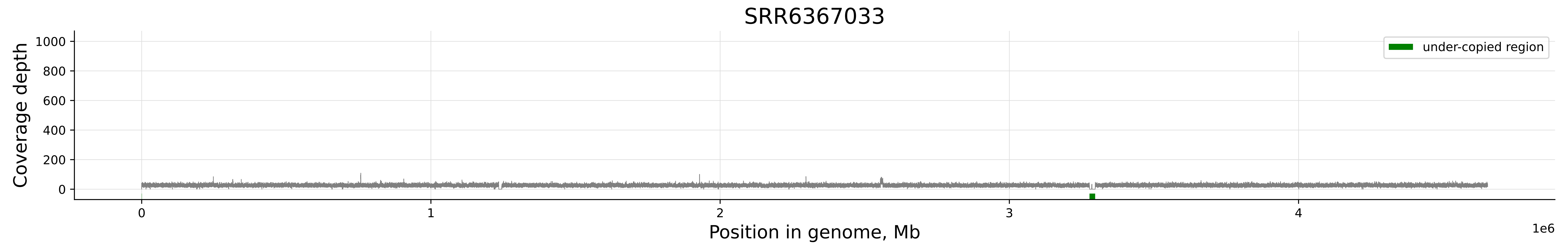
SRR6367033
Coverage depth
1000
800
600
400
200
0
0
1
2
3
4
1e6
Position in genome, Mb
under-copied region

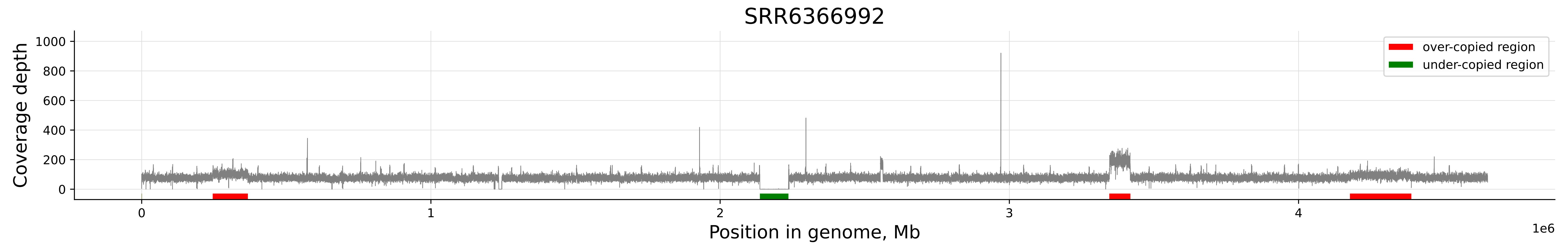

SRR6366992
Coverage depth
1000
800
600
400
200
0
over-copied region
under-copied region
0
1
2
3
4
1e6
Position in genome, Mb

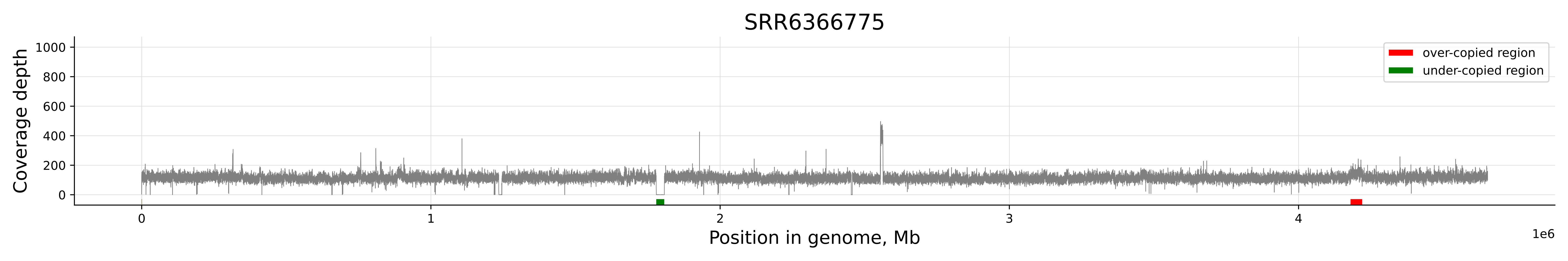

SRR6366775
Coverage depth
1000
800
600
400
200
0
over-copied region
under-copied region
0
1
2
3
4
1e6
Position in genome, Mb

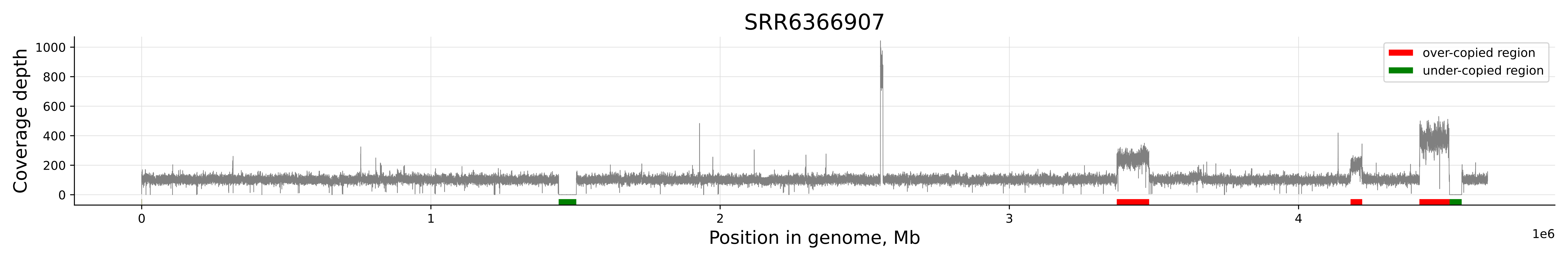
SRR6366907
Coverage depth
Position in genome, Mb
over-copied region
under-copied region
1e6
0
200
400
600
800
1000
0
1
2
3
4

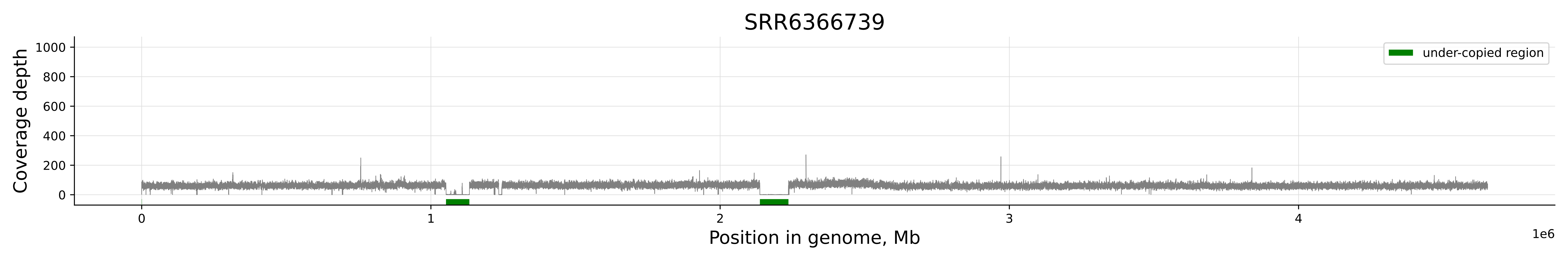

SRR6366739
Coverage depth
1000
800
600
400
200
0
under-copied region
0
1
2
3
4
1e6
Position in genome, Mb

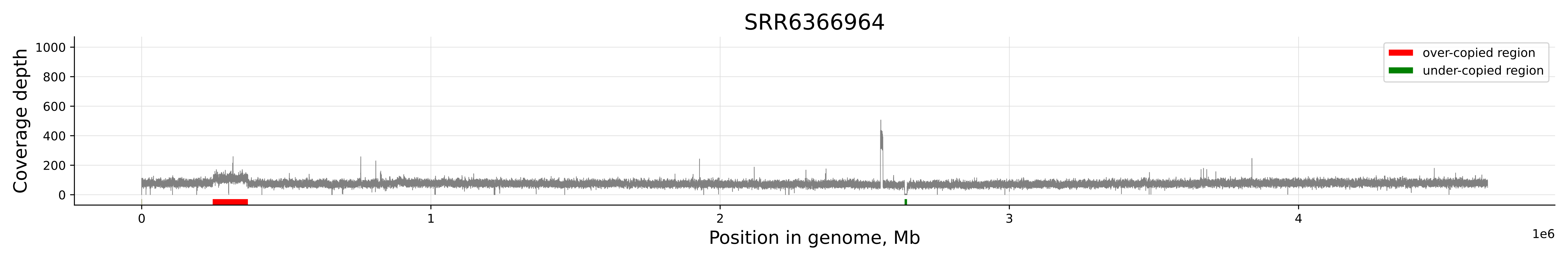

SRR6366964
over-copied region
under-copied region
Coverage depth
0
200
400
600
800
1000
0
1
2
3
4
1e6
Position in genome, Mb

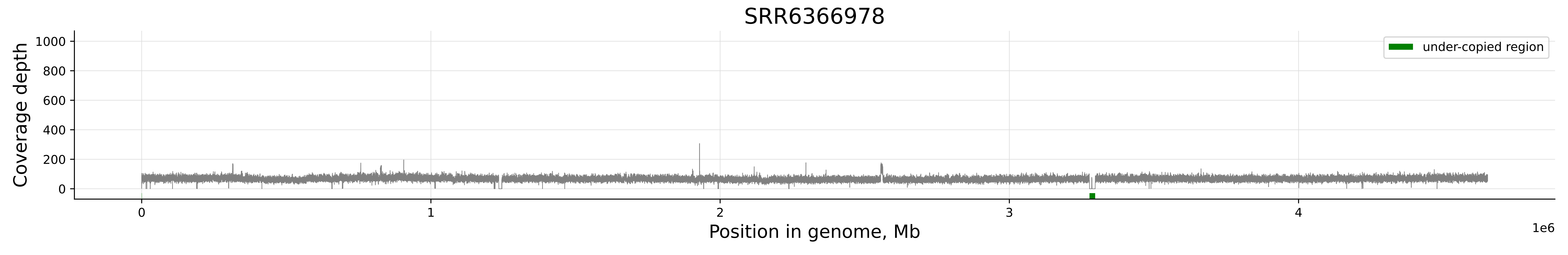
SRR6366978
under-copied region
Coverage depth
1000
800
600
400
200
0
0
1
2
3
4
1e6
Position in genome, Mb

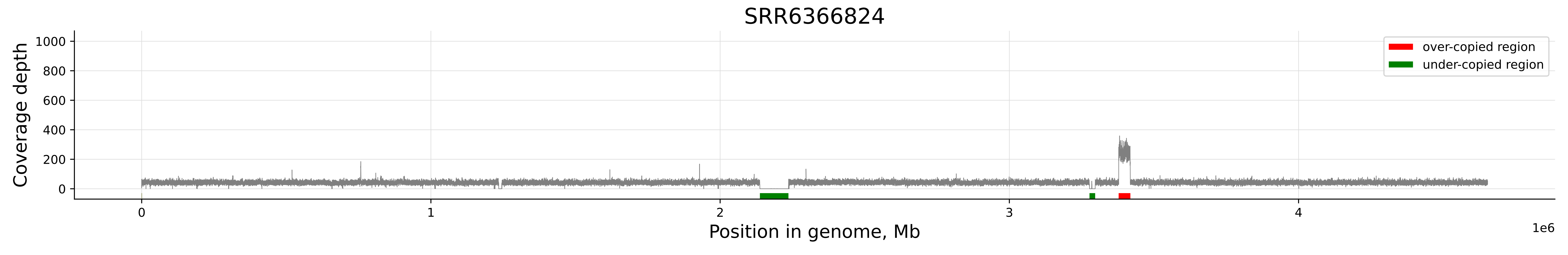
SRR6366824
Coverage depth
Position in genome, Mb
1e6
over-copied region
under-copied region
0
200
400
600
800
1000
0
1
2
3
4

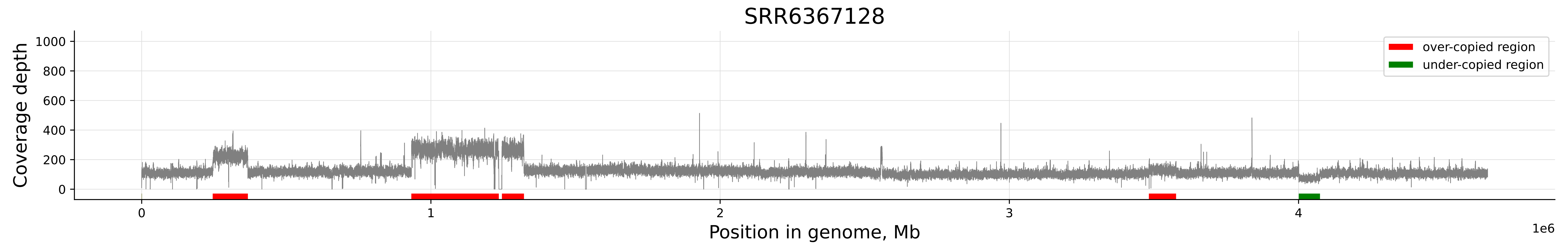
SRR6367128
Coverage depth
1000
800
600
400
200
0
over-copied region
under-copied region
0
1
2
3
4
1e6
Position in genome, Mb

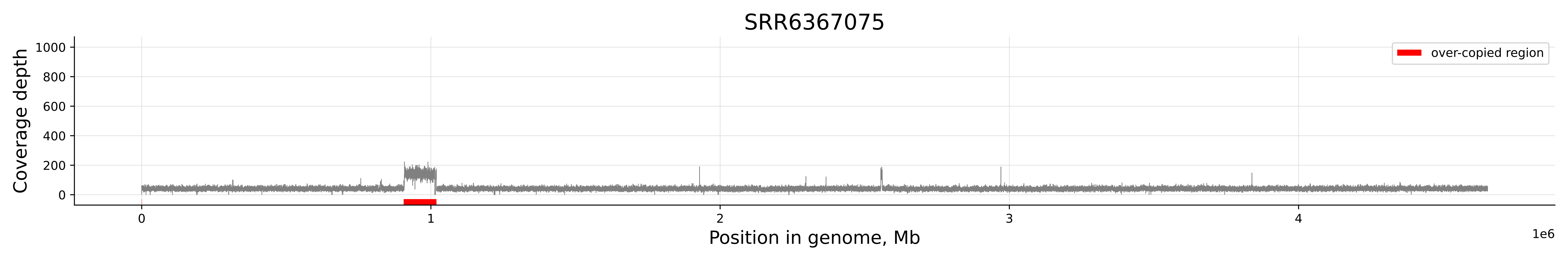
SRR6367075
Coverage depth
1000
800
600
400
200
0
over-copied region
0
1
2
3
4
1e6
Position in genome, Mb

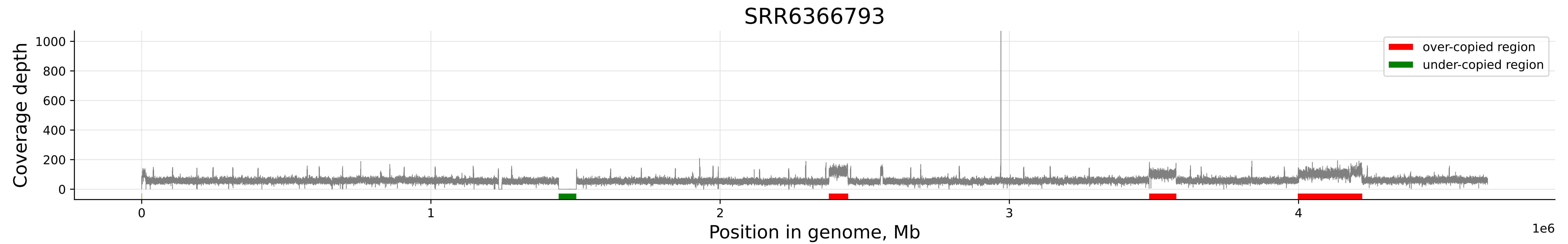

SRR6366793
Coverage depth
Position in genome, Mb
1e6
0
200
400
600
800
1000
0
1
2
3
4
over-copied region
under-copied region

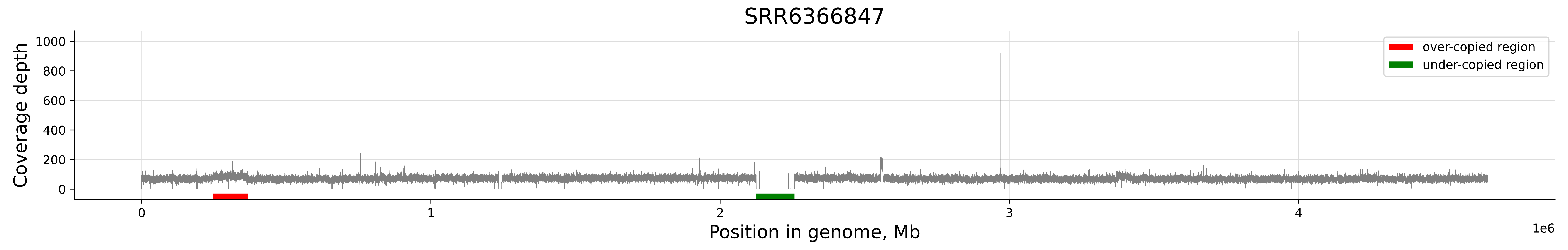

SRR6366847
Coverage depth
1000
800
600
400
200
0
over-copied region
under-copied region
0
1
2
3
4
1e6
Position in genome, Mb

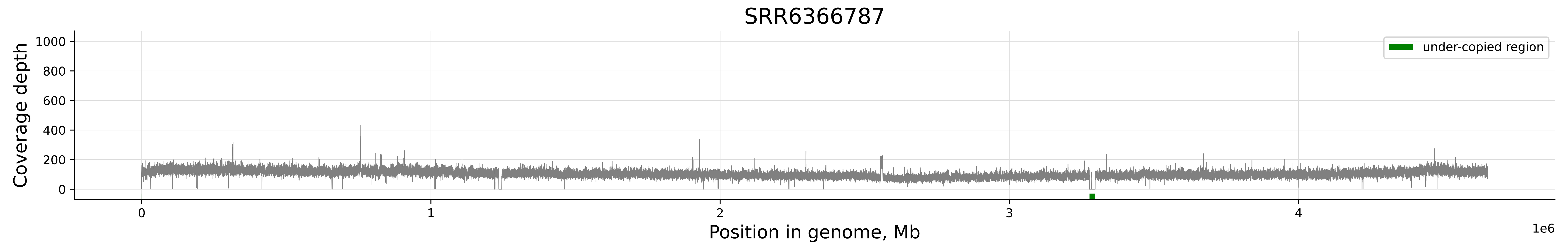

SRR6366787
under-copied region
Coverage depth
0
200
400
600
800
1000
0
1
2
3
4
1e6
Position in genome, Mb

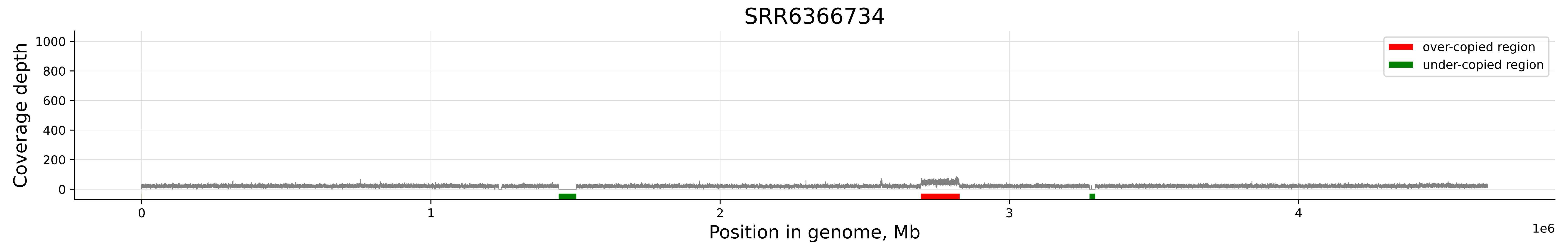

SRR6366734
Coverage depth
Position in genome, Mb
1e6
over-copied region
under-copied region
0
200
400
600
800
1000
0
1
2
3
4

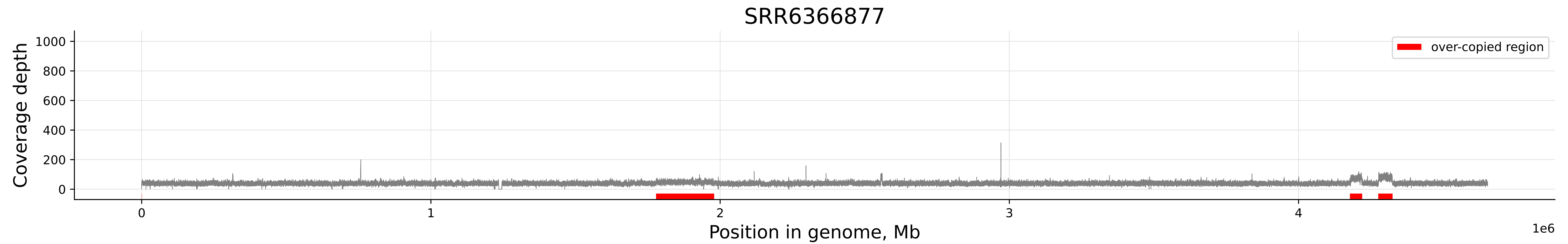

SRR6366877
over-copied region
Coverage depth
1000
800
600
400
200
0
0
1
2
3
4
1e6
Position in genome, Mb

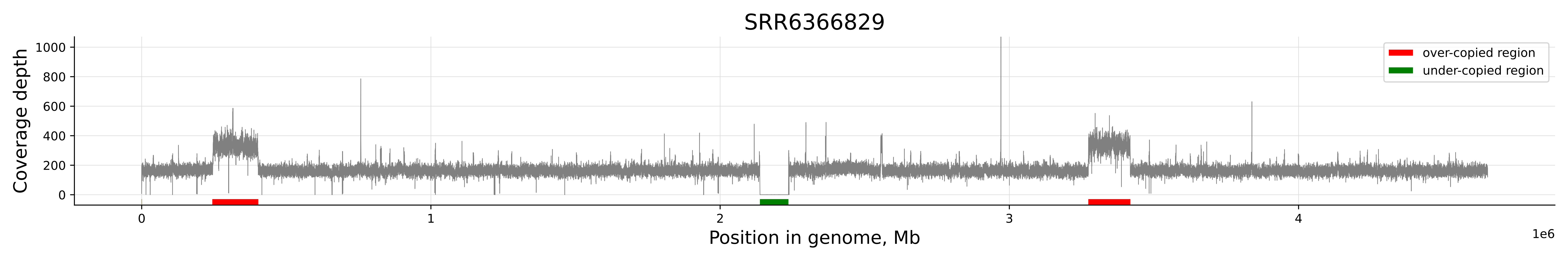

SRR6366829
Coverage depth
1000
800
600
400
200
0
over-copied region
under-copied region
0
1
2
3
4
1e6
Position in genome, Mb

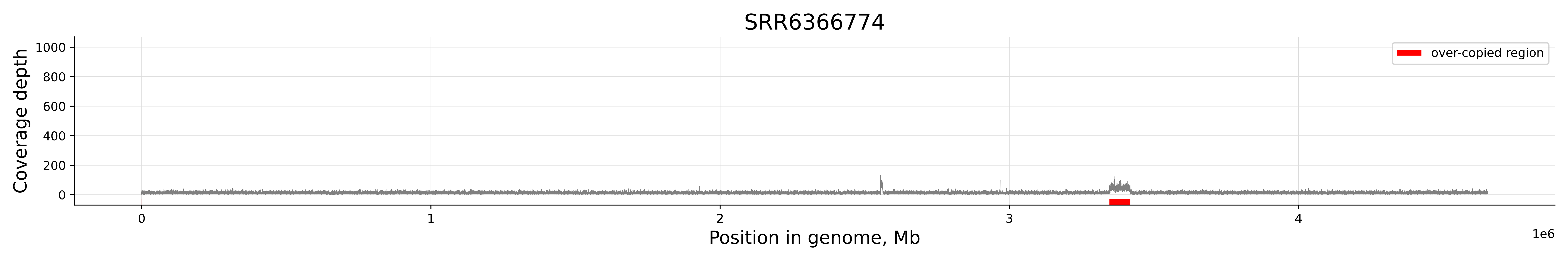
SRR6366774
over-copied region
Coverage depth
Position in genome, Mb
1e6
0
200
400
600
800
1000
0
1
2
3
4

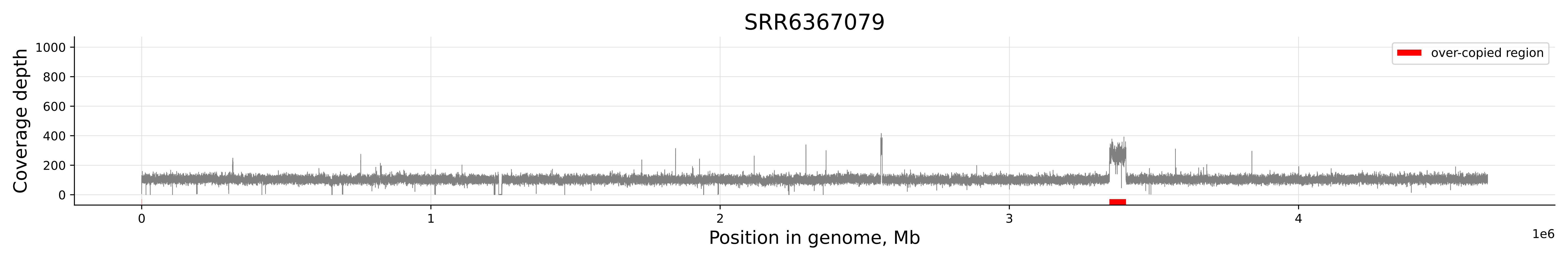
SRR6367079
over-copied region
Coverage depth
0
200
400
600
800
1000
0
1
2
3
4
1e6
Position in genome, Mb

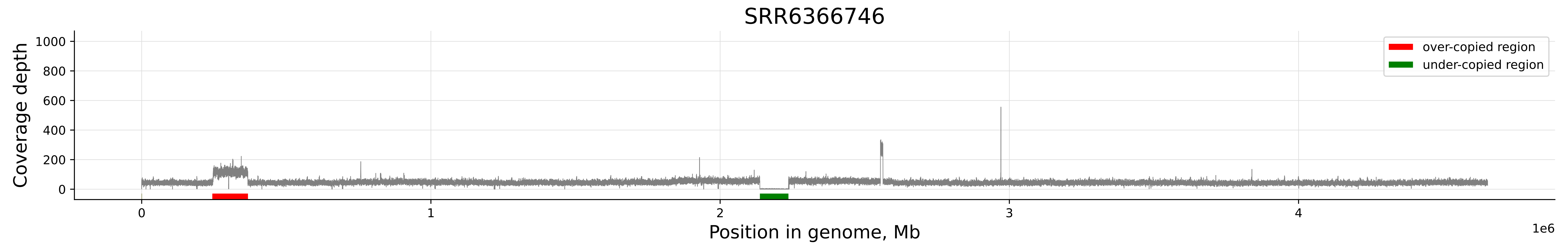
SRR6366746
over-copied region
under-copied region
Coverage depth
1000
800
600
400
200
0
0
1
2
3
4
1e6
Position in genome, Mb

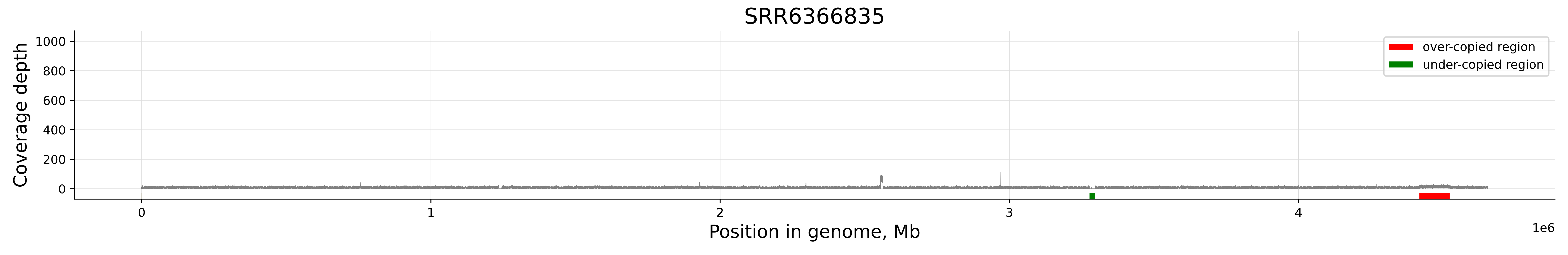
SRR6366835
Coverage depth
Position in genome, Mb
over-copied region
under-copied region
1000
800
600
400
200
0
0
1
2
3
4
1e6

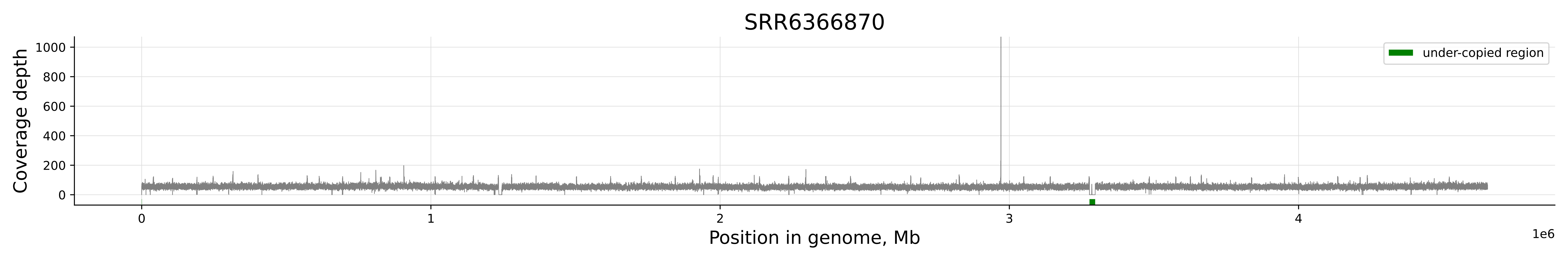
SRR6366870
Coverage depth
1000
800
600
400
200
0
under-copied region
0
1
2
3
4
1e6
Position in genome, Mb

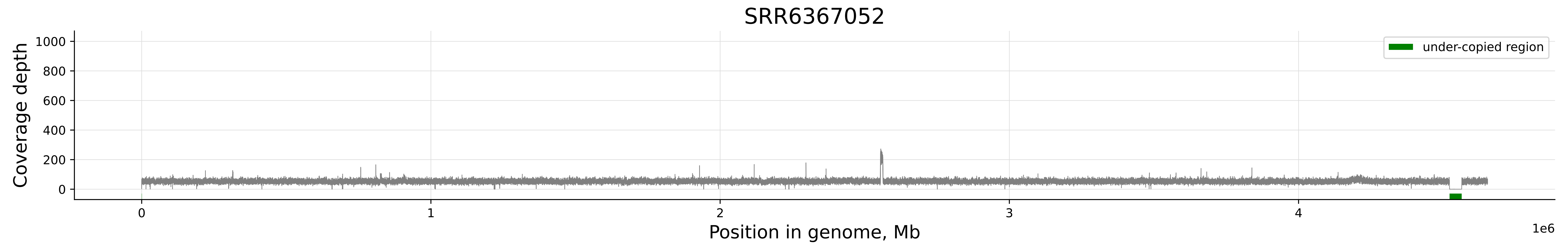

SRR6367052
Coverage depth
1000
800
600
400
200
0
under-copied region
0
1
2
3
4
1e6
Position in genome, Mb

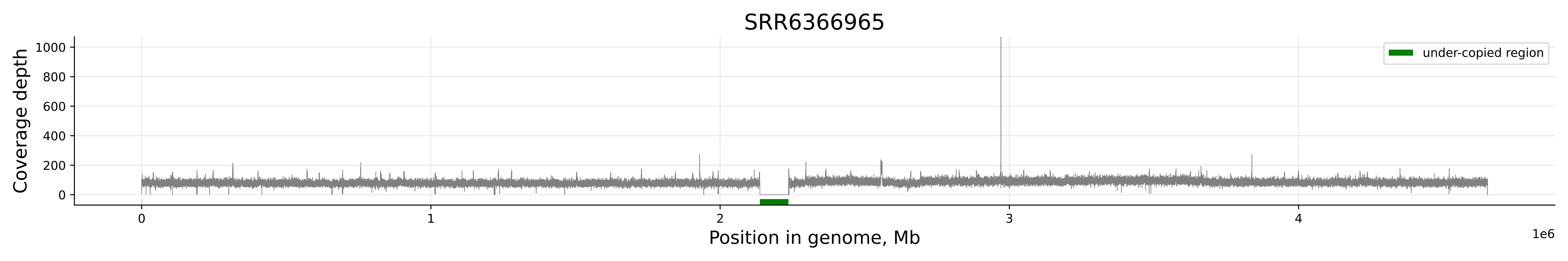

SRR6366965
under-copied region
Coverage depth
1000
800
600
400
200
0
Position in genome, Mb
0
1
2
3
4
1e6

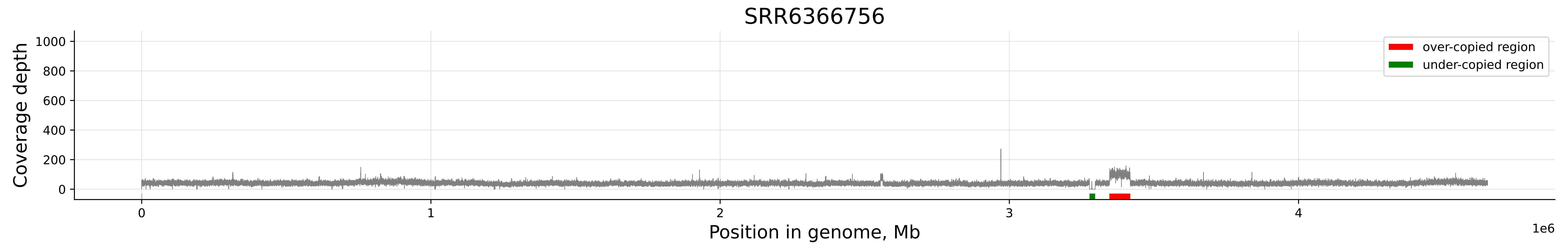

SRR6366756
Coverage depth
1000
800
600
400
200
0
over-copied region
under-copied region
0
1
2
3
4
1e6
Position in genome, Mb

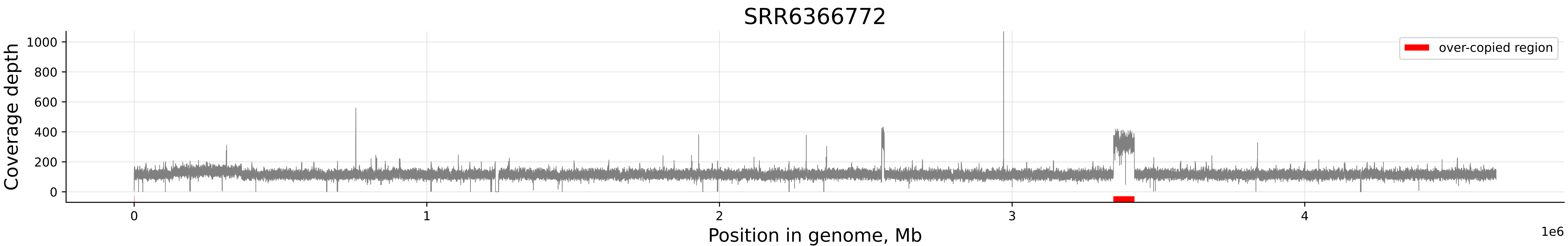
SRR6366772
Coverage depth
1000
800
600
400
200
0
0
1
2
3
4
1e6
Position in genome, Mb
over-copied region

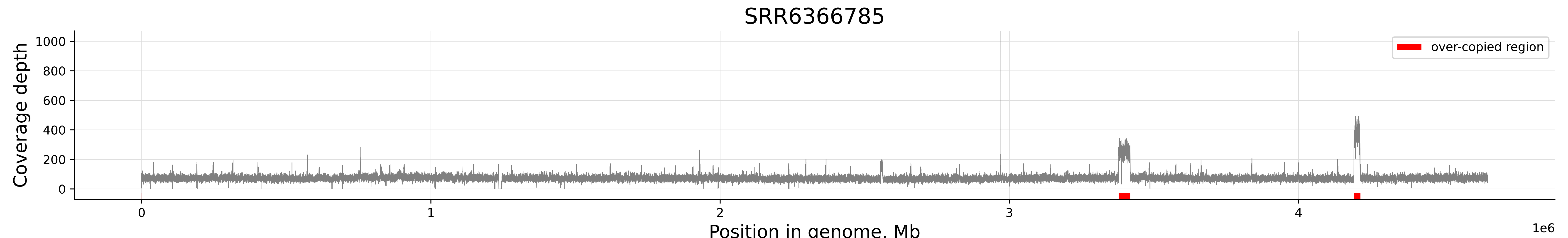
SRR6366785
Coverage depth
Position in genome, Mb
over-copied region
1e6

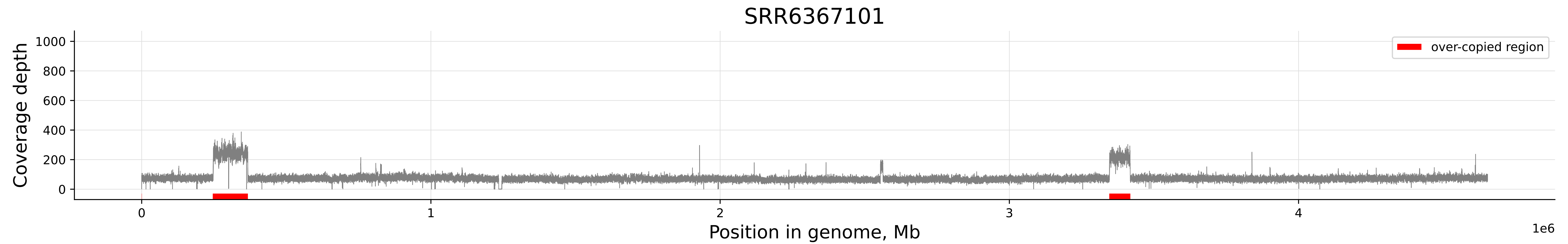

SRR6367101
Coverage depth
1000
800
600
400
200
0
over-copied region
0
1
2
3
4
1e6
Position in genome, Mb

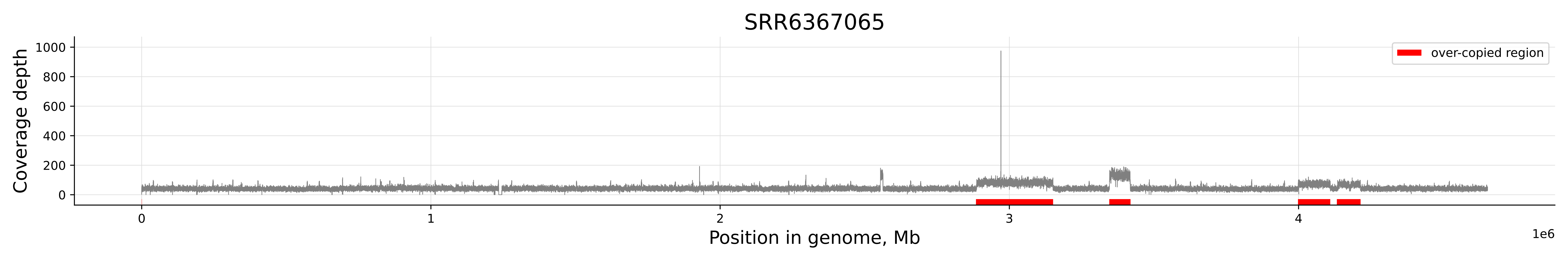

SRR6367065
Coverage depth
Position in genome, Mb
over-copied region
1e6
0
200
400
600
800
1000
0
1
2
3
4

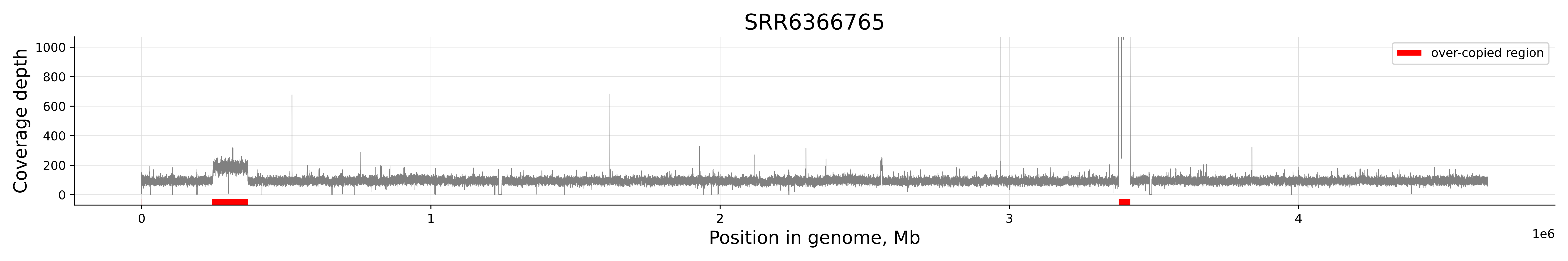
SRR6366765
Coverage depth
Position in genome, Mb
over-copied region
1e6
0
200
400
600
800
1000
0
1
2
3
4

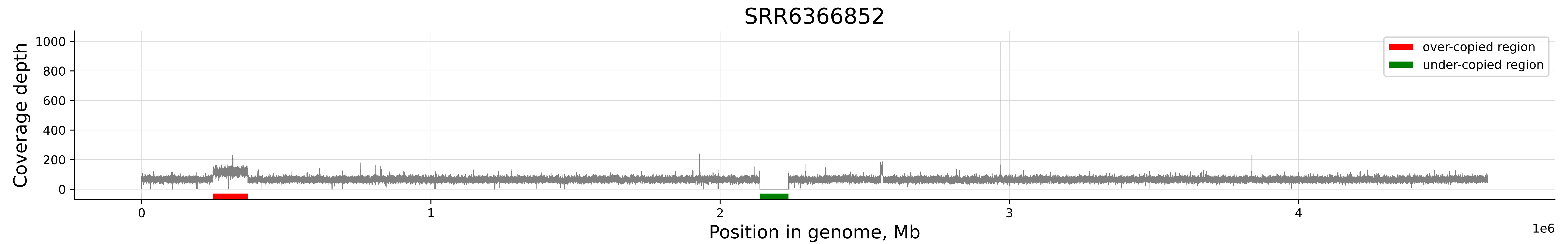
SRR6366852
over-copied region
under-copied region
Coverage depth
1000
800
600
400
200
0
0
1
2
3
4
1e6
Position in genome, Mb

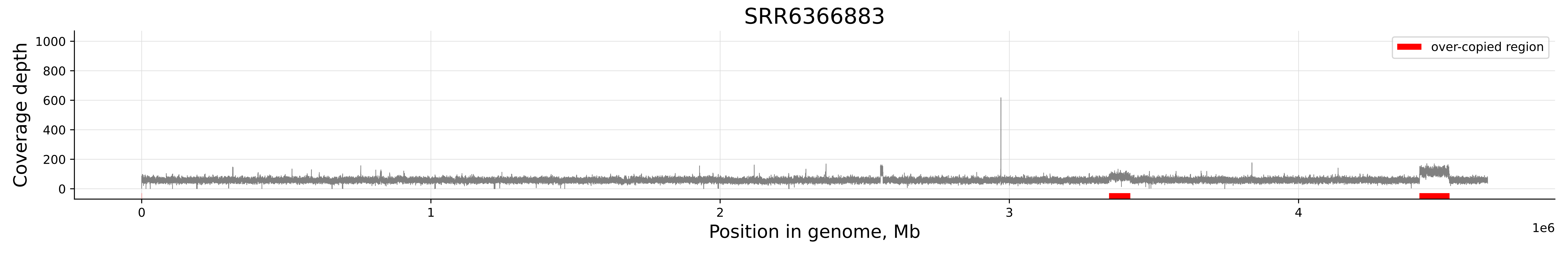
SRR6366883
Coverage depth
Position in genome, Mb
over-copied region
1e6
0
200
400
600
800
1000
0
1
2
3
4

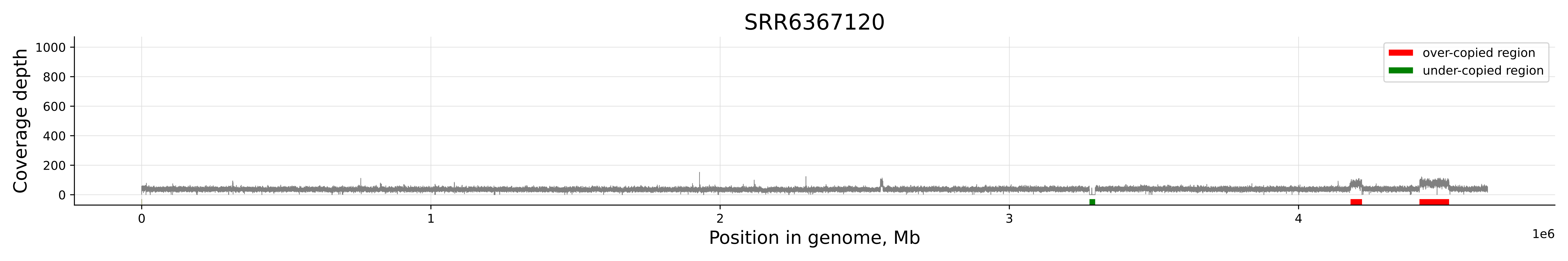
SRR6367120
Coverage depth
1000
800
600
400
200
0
0
1
2
3
4
1e6
Position in genome, Mb
over-copied region
under-copied region

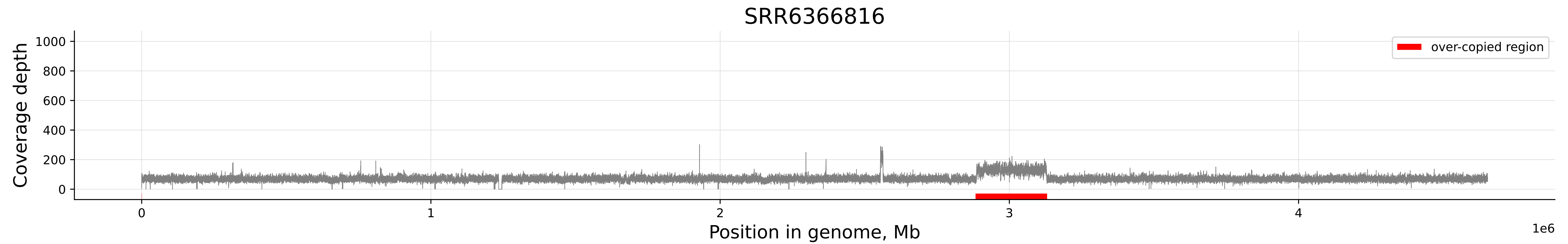

SRR6366816
Coverage depth
1000
800
600
400
200
0
over-copied region
0
1
2
3
4
1e6
Position in genome, Mb

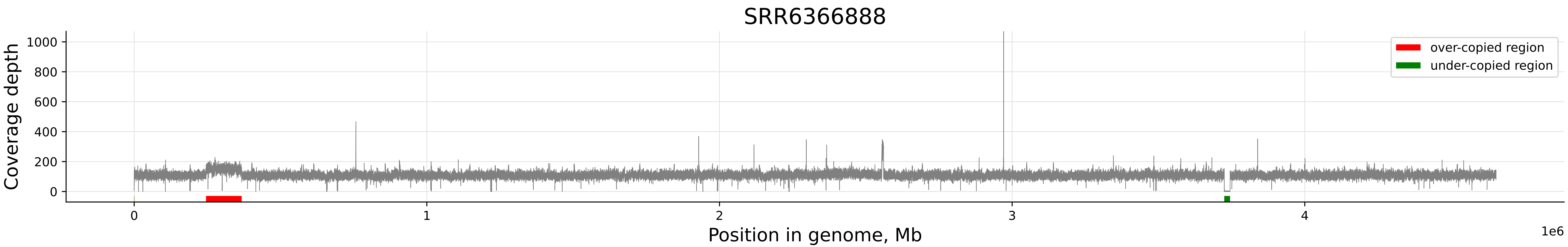
SRR6366888
Coverage depth
1000
800
600
400
200
0
0
1
2
3
4
1e6
Position in genome, Mb
over-copied region
under-copied region

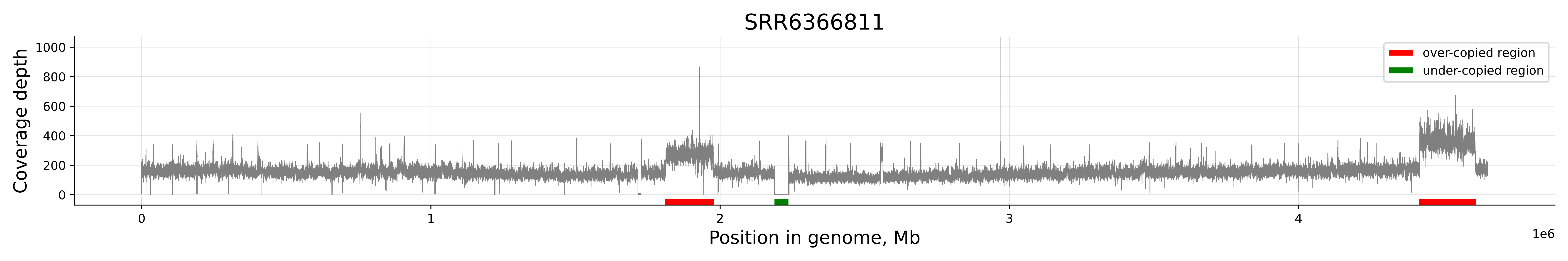
SRR6366811
over-copied region
under-copied region
Coverage depth
1000
800
600
400
200
0
0
1
2
3
4
Position in genome, Mb
1e6

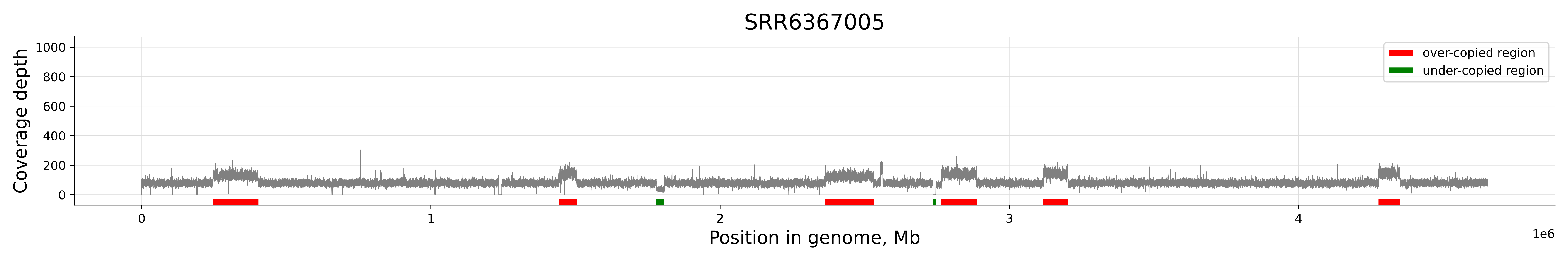

SRR6367005
Coverage depth
1000
800
600
400
200
0
0
1
2
3
4
1e6
Position in genome, Mb
over-copied region
under-copied region

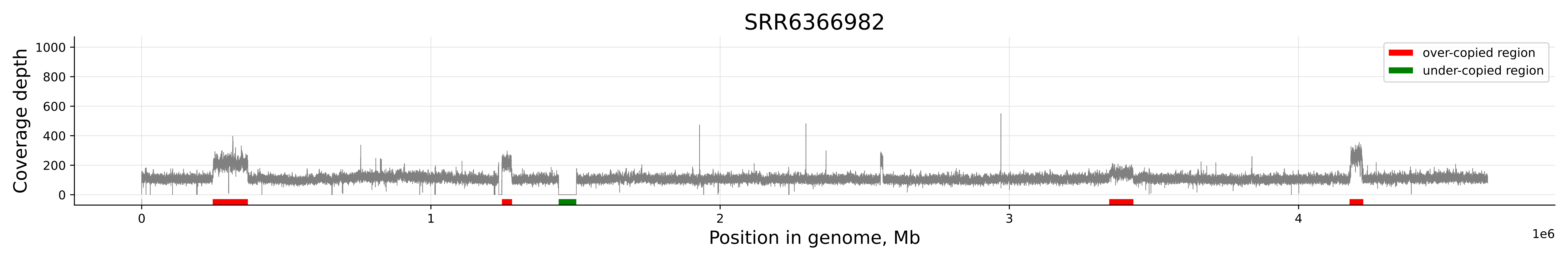
SRR6366982
Coverage depth
0
200
400
600
800
1000
over-copied region
under-copied region
0
1
2
3
4
1e6
Position in genome, Mb

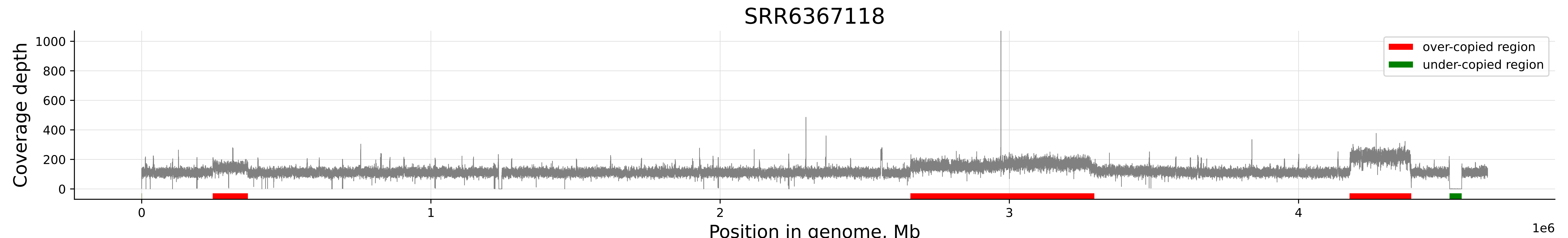
SRR6367118
Coverage depth
Position in genome, Mb
over-copied region
under-copied region
1e6

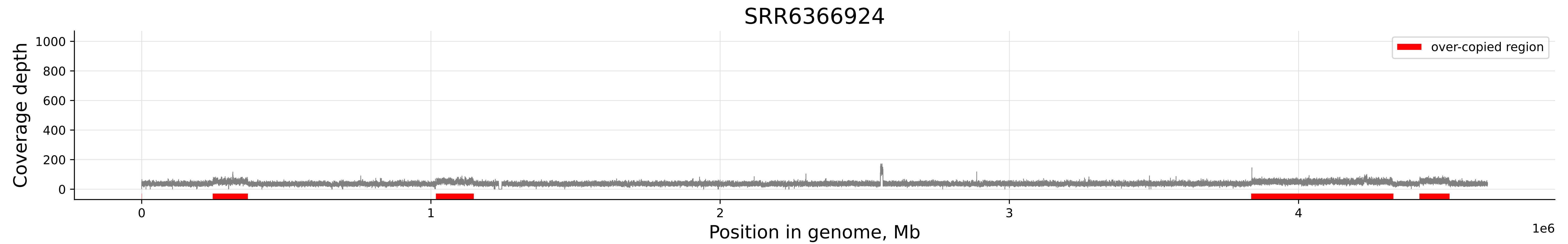
SRR6366924
Coverage depth
1000
800
600
400
200
0
over-copied region
0
1
2
3
4
1e6
Position in genome, Mb

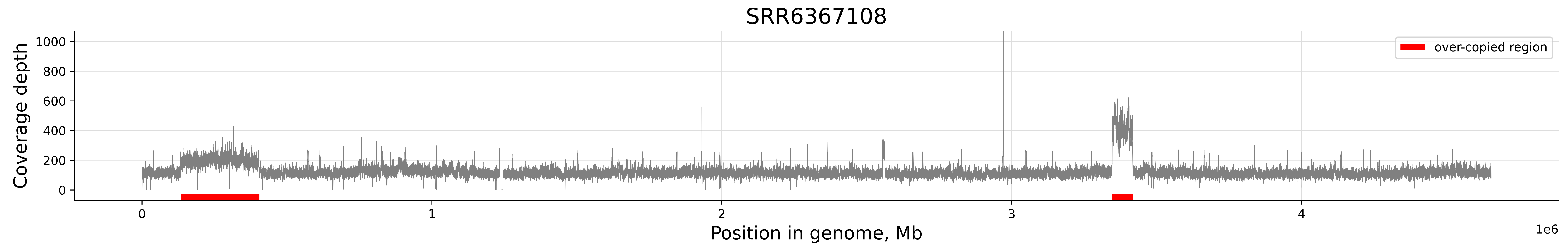

SRR6367108
over-copied region
Coverage depth
1000
800
600
400
200
0
0
1
2
3
4
1e6
Position in genome, Mb

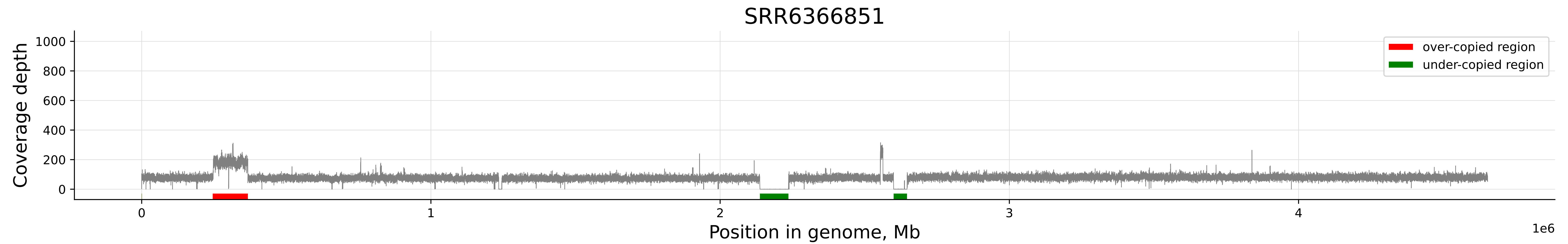
SRR6366851
Coverage depth
1000
800
600
400
200
0
0
1
2
3
4
1e6
Position in genome, Mb
over-copied region
under-copied region

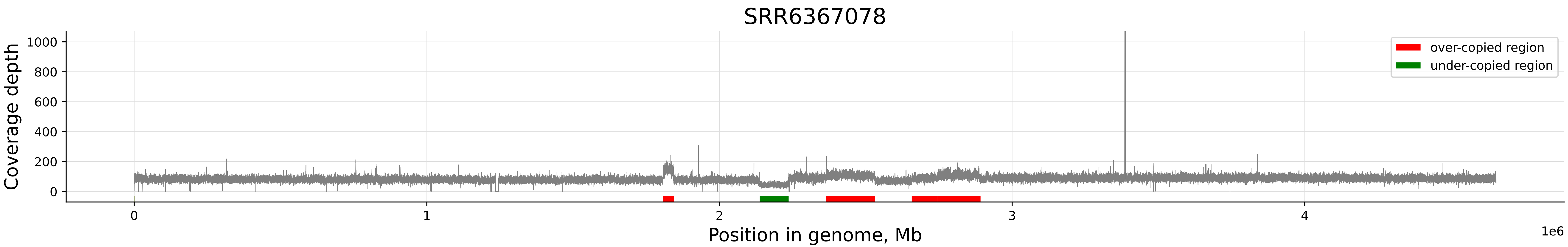
SRR6367078
Coverage depth
1000
800
600
400
200
0
0
1
2
3
4
1e6
Position in genome, Mb
over-copied region
under-copied region

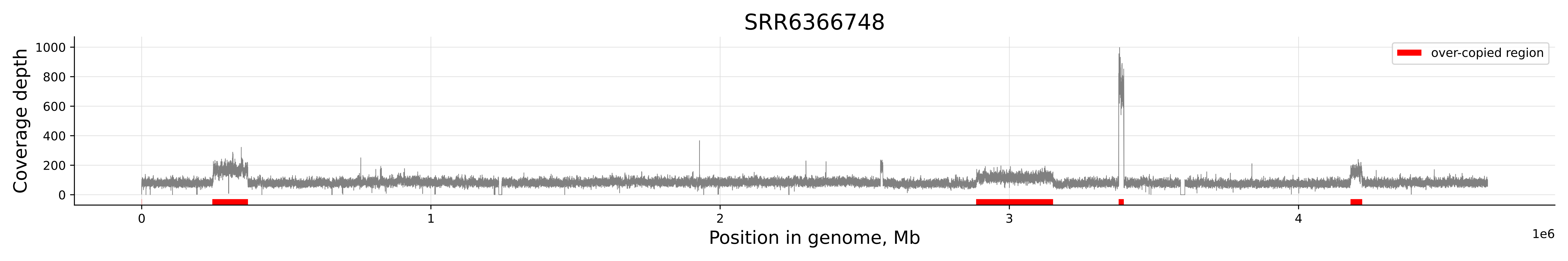

SRR6366748
Coverage depth
Position in genome, Mb
1e6
over-copied region
0
200
400
600
800
1000
0
1
2
3
4

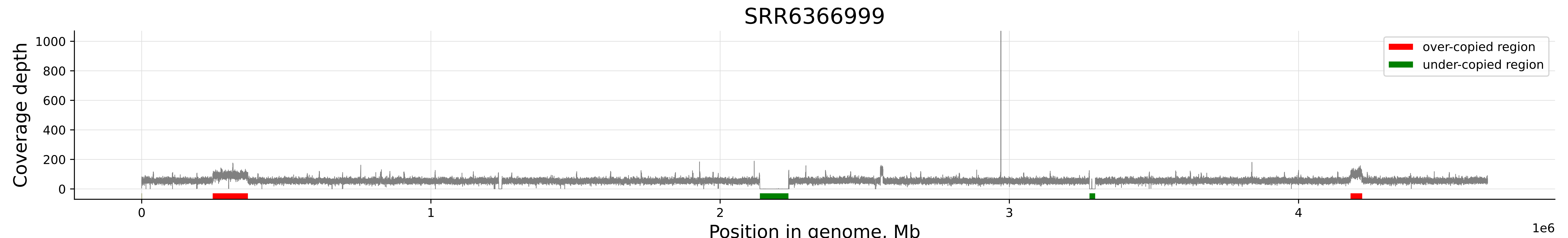

SRR6366999
Coverage depth
Position in genome, Mb
1e6
over-copied region
under-copied region
0
200
400
600
800
1000
0
1
2
3
4

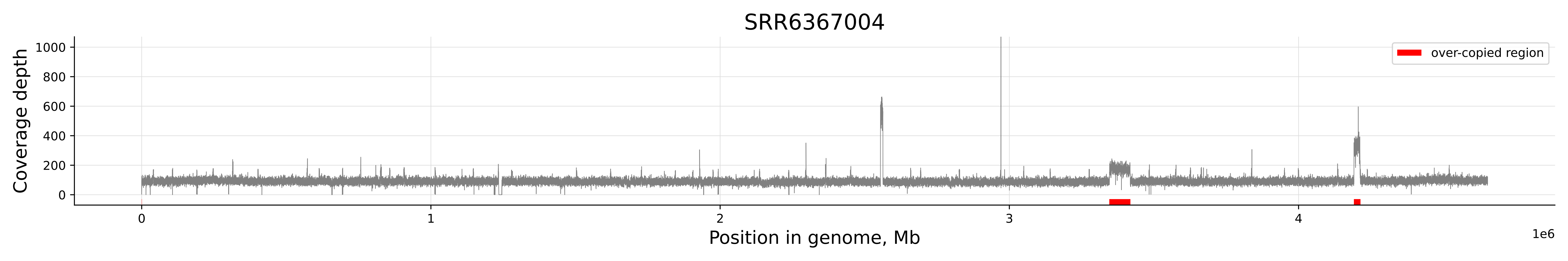
SRR6367004
Coverage depth
1000
800
600
400
200
0
over-copied region
0
1
2
3
4
Position in genome, Mb
1e6

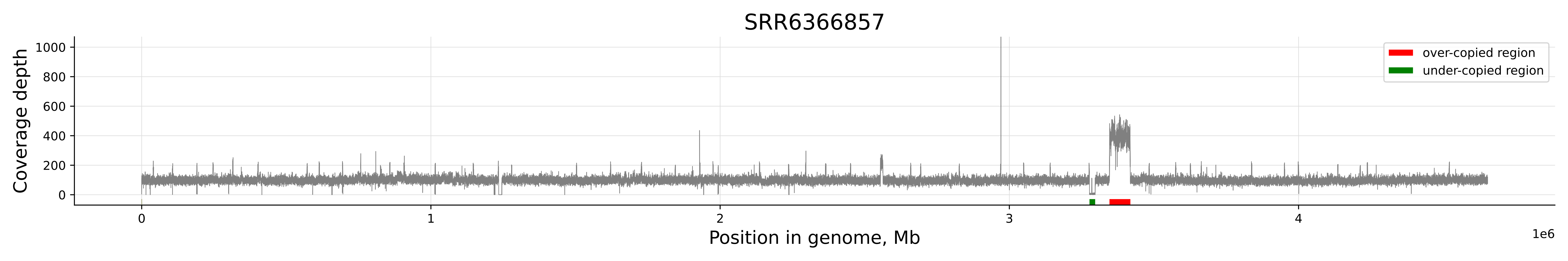

SRR6366857
Coverage depth
Position in genome, Mb
1e6
over-copied region
under-copied region
0
200
400
600
800
1000
0
1
2
3
4

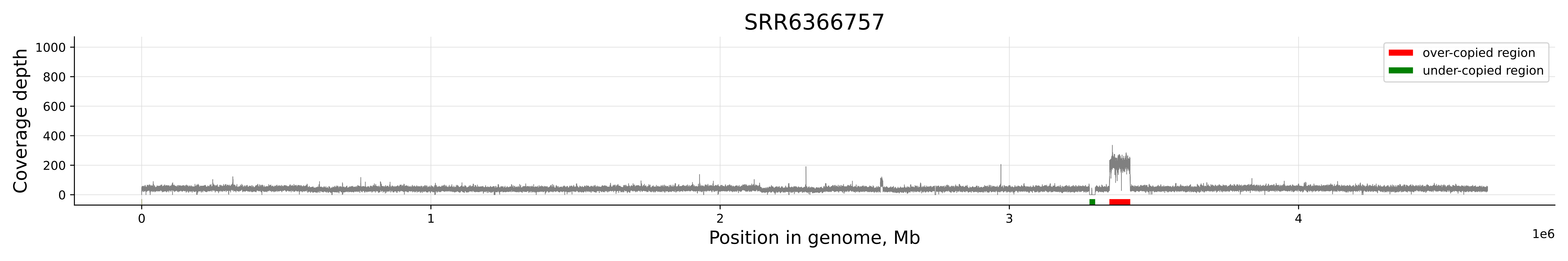
SRR6366757
Coverage depth
Position in genome, Mb
over-copied region
under-copied region
0
200
400
600
800
1000
0
1
2
3
4
1e6

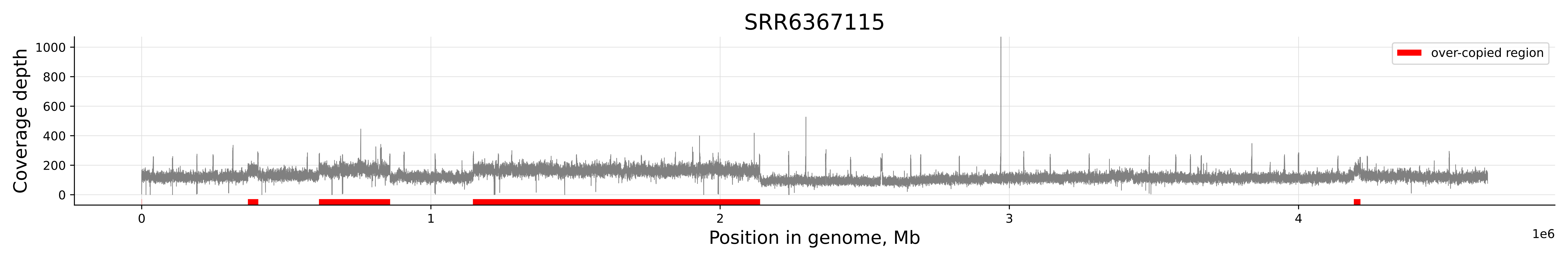

SRR6367115
Coverage depth
1000
800
600
400
200
0
over-copied region
0
1
2
3
4
1e6
Position in genome, Mb

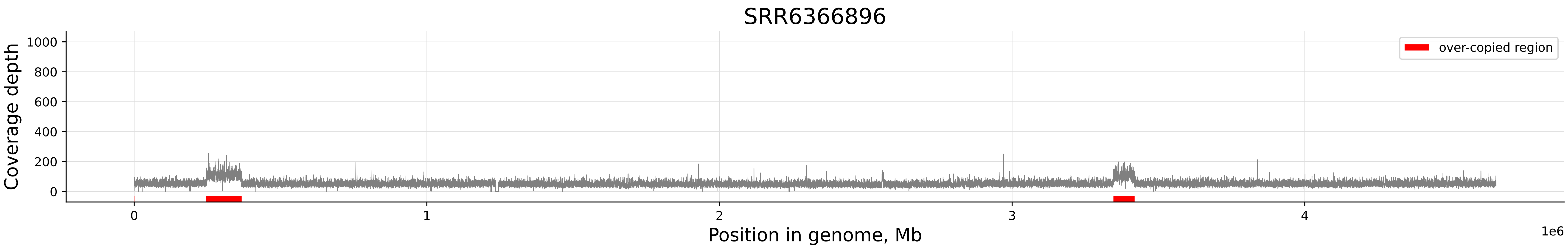
SRR6366896
over-copied region
Coverage depth
1000
800
600
400
200
0
0
1
2
3
4
1e6
Position in genome, Mb

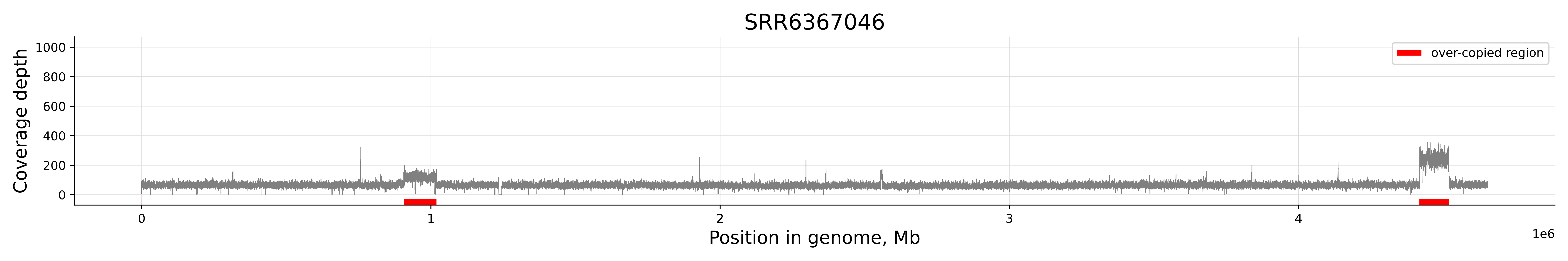
SRR6367046
over-copied region
Coverage depth
1000
800
600
400
200
0
0
1
2
3
4
1e6
Position in genome, Mb

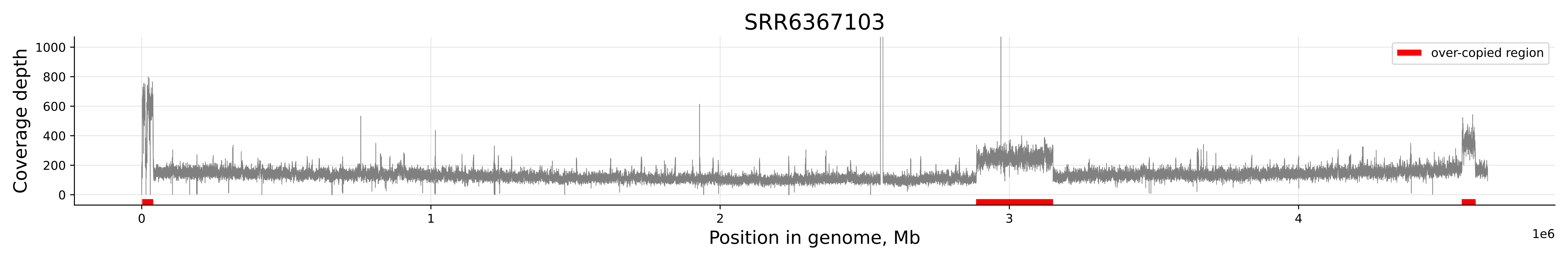

SRR6367103
Coverage depth
Position in genome, Mb
over-copied region
1000
800
600
400
200
0
0
1
2
3
4
1e6

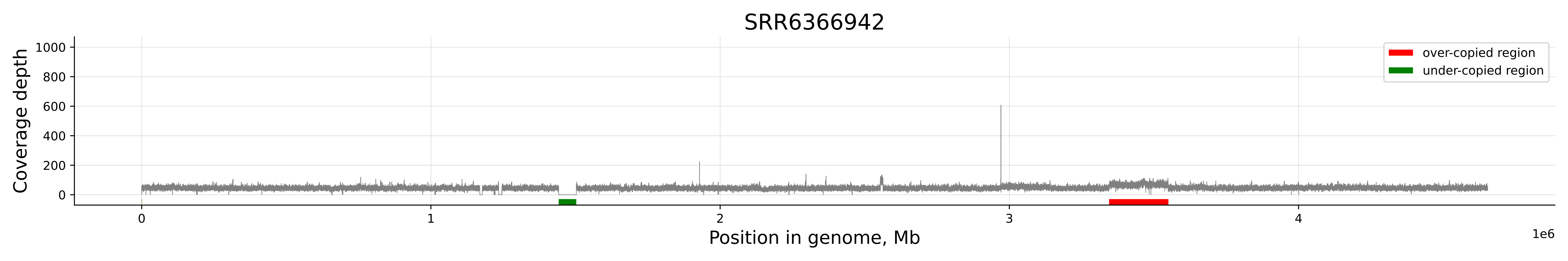

SRR6366942
Coverage depth
1000
800
600
400
200
0
over-copied region
under-copied region
0
1
2
3
4
1e6
Position in genome, Mb

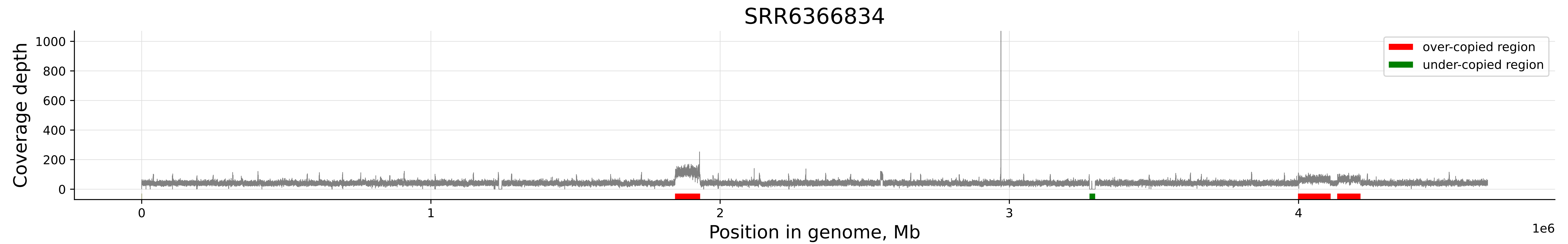

SRR6366834
Coverage depth
Position in genome, Mb
over-copied region
under-copied region
1000
800
600
400
200
0
0
1
2
3
4
1e6

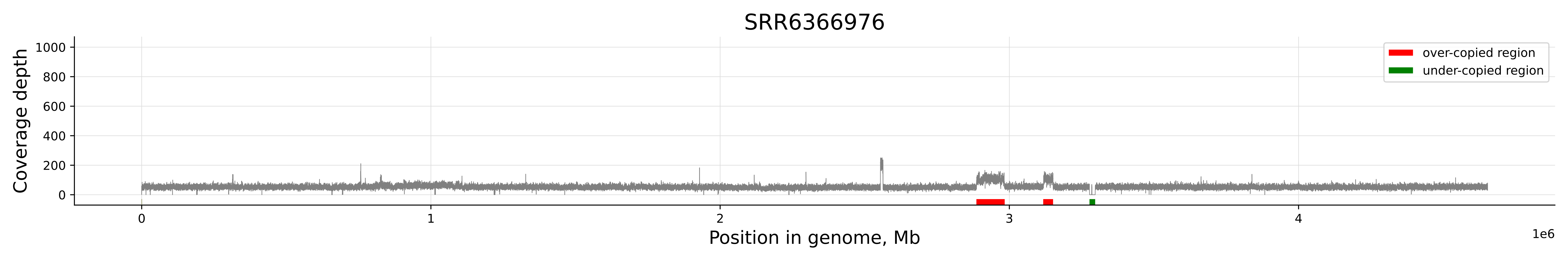
SRR6366976
Coverage depth
1000
800
600
400
200
0
over-copied region
under-copied region
0
1
2
3
4
1e6
Position in genome, Mb

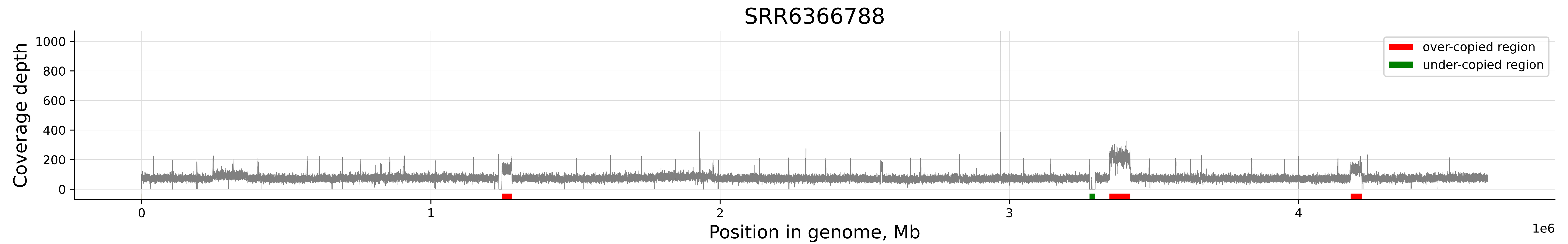

SRR6366788
Coverage depth
Position in genome, Mb
1e6
over-copied region
under-copied region
0
200
400
600
800
1000
0
1
2
3
4

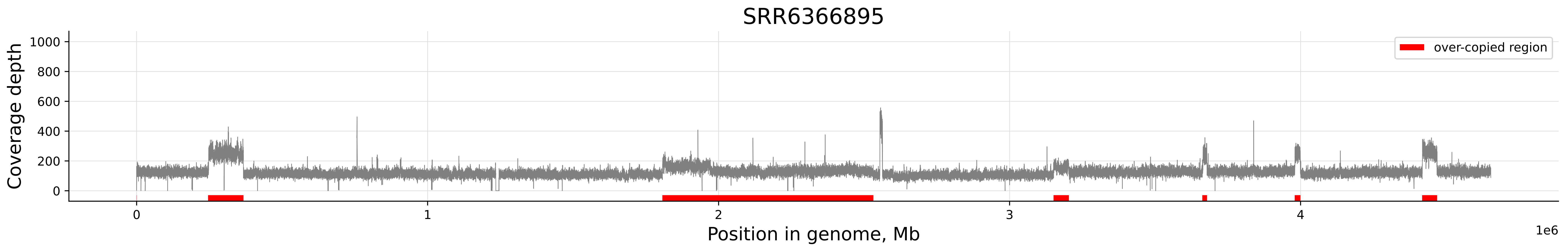
SRR6366895
Coverage depth
0
200
400
600
800
1000
Position in genome, Mb
0
1
2
3
4
1e6
over-copied region

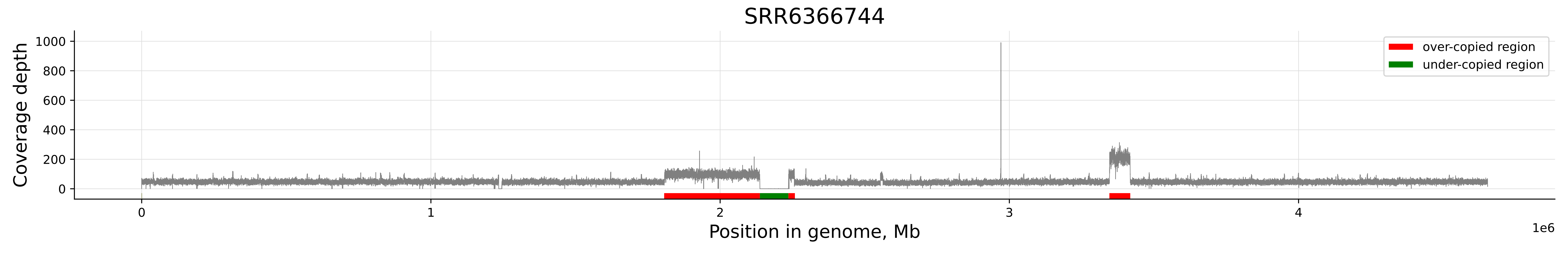
SRR6366744
Coverage depth
1000
800
600
400
200
0
0
1
2
3
4
1e6
Position in genome, Mb
over-copied region
under-copied region

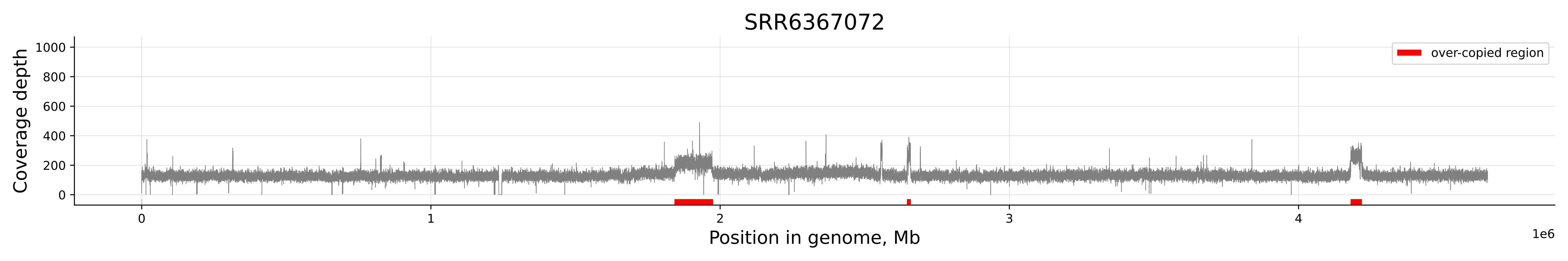

SRR6367072
Coverage depth
Position in genome, Mb
1e6
over-copied region
0
200
400
600
800
1000
0
1
2
3
4

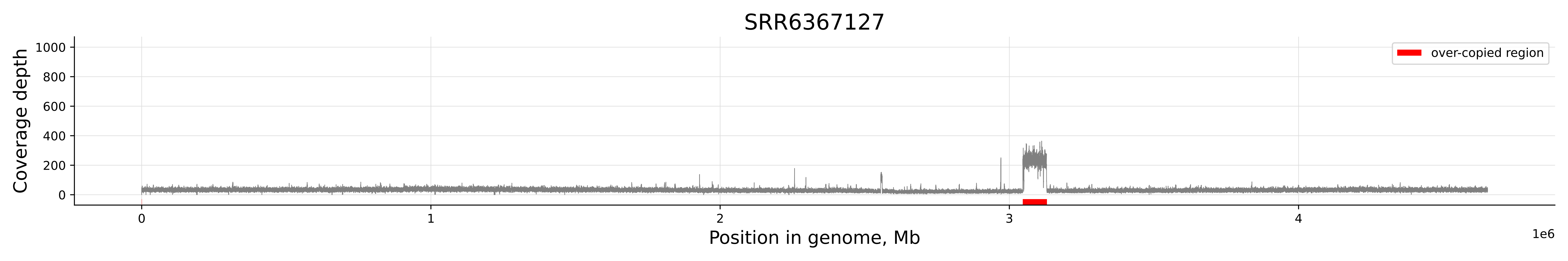
SRR6367127
over-copied region
Coverage depth
1000
800
600
400
200
0
0
1
2
3
4
1e6
Position in genome, Mb

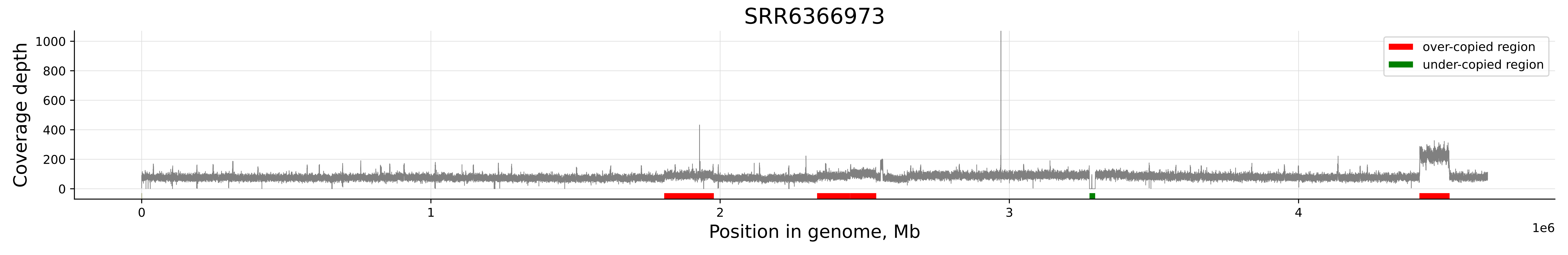

SRR6366973
Coverage depth
1000
800
600
400
200
0
over-copied region
under-copied region
0
1
2
3
4
1e6
Position in genome, Mb

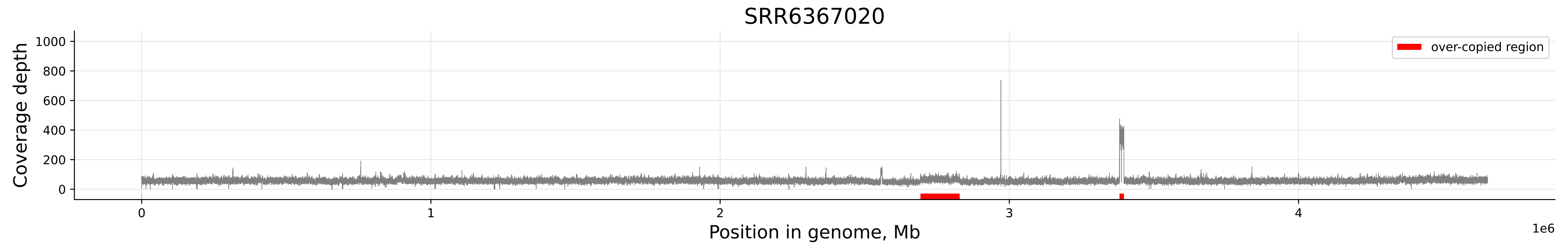
SRR6367020
Coverage depth
1000
800
600
400
200
0
over-copied region
0
1
2
3
4
1e6
Position in genome, Mb

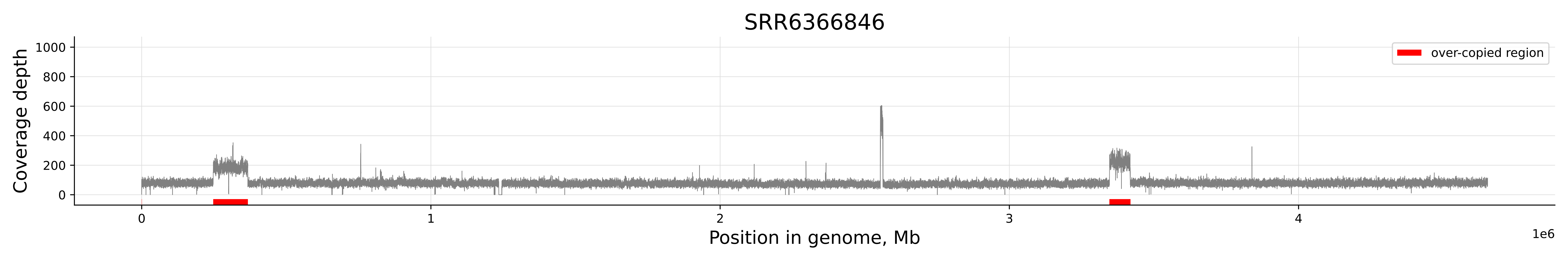

SRR6366846
Coverage depth
1000
800
600
400
200
0
over-copied region
0
1
2
3
4
Position in genome, Mb
1e6

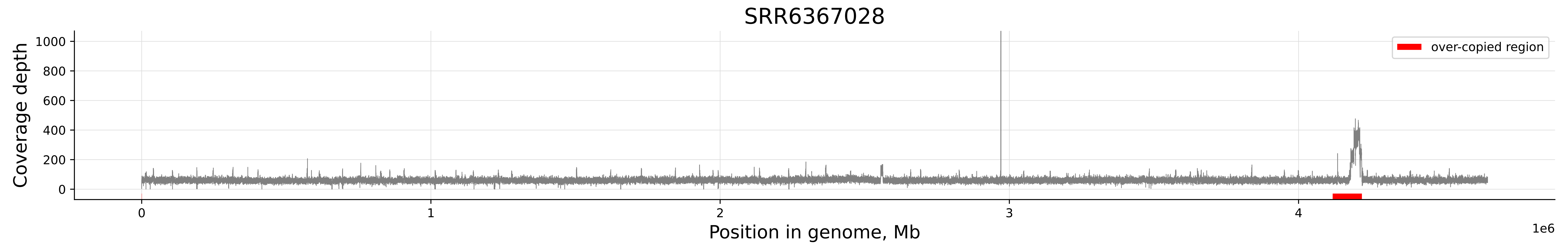
SRR6367028
Coverage depth
1000
800
600
400
200
0
0
1
2
3
4
1e6
Position in genome, Mb
over-copied region

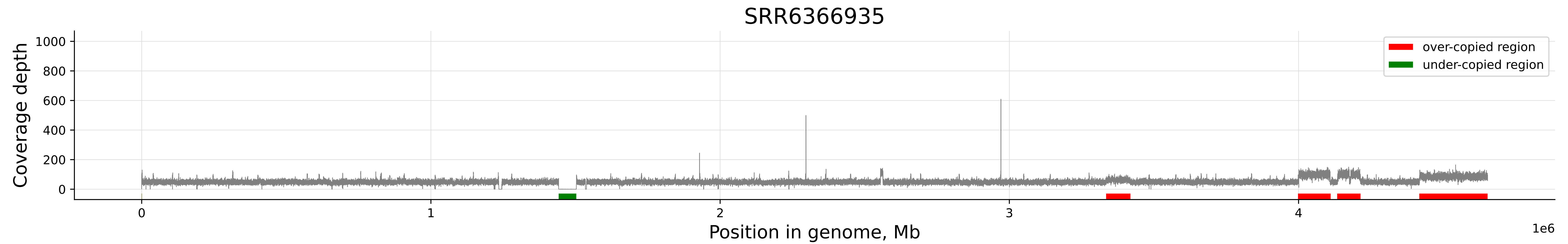
SRR6366935
Coverage depth
1000
800
600
400
200
0
over-copied region
under-copied region
0
1
2
3
4
1e6
Position in genome, Mb

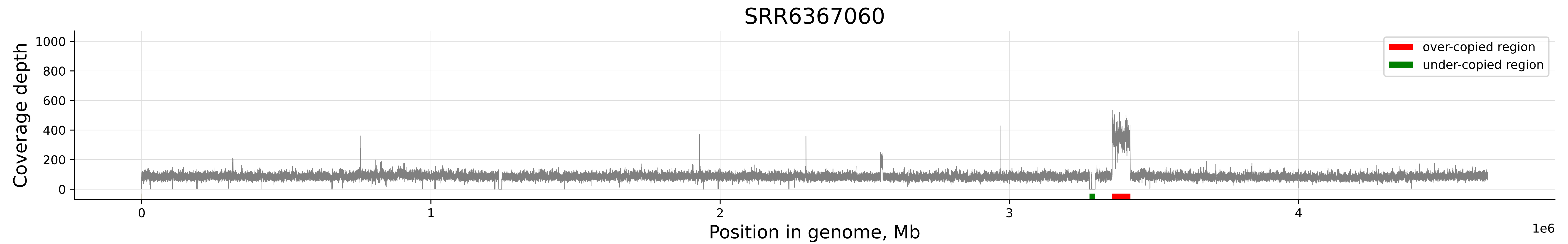
SRR6367060
Coverage depth
1000
800
600
400
200
0
Position in genome, Mb
1e6
over-copied region
under-copied region

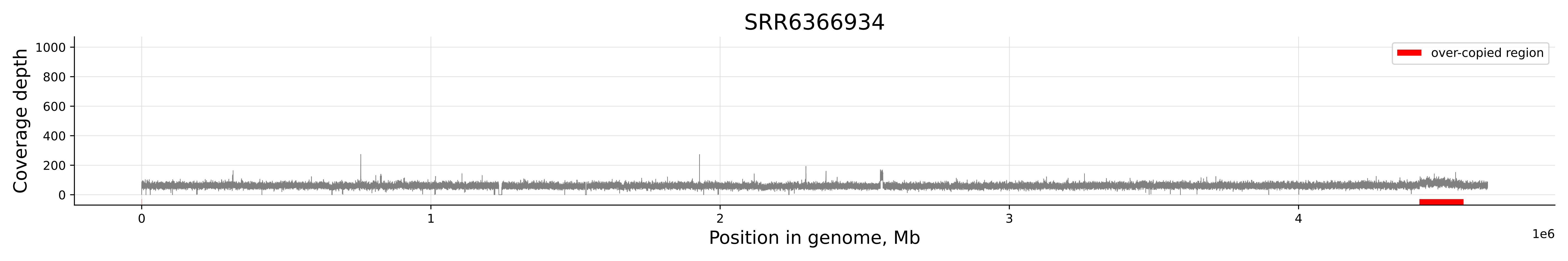

SRR6366934
Coverage depth
1000
800
600
400
200
0
over-copied region
0
1
2
3
4
1e6
Position in genome, Mb

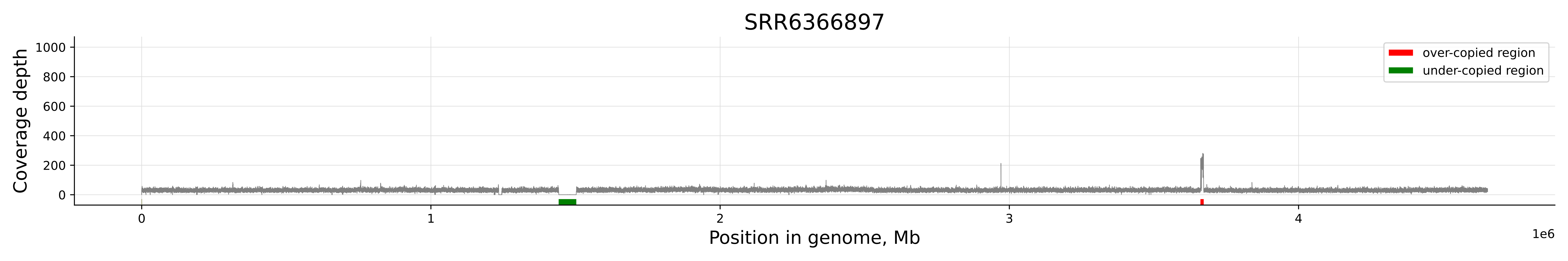
SRR6366897
Coverage depth
1000
800
600
400
200
0
0
1
2
3
4
1e6
Position in genome, Mb
over-copied region
under-copied region

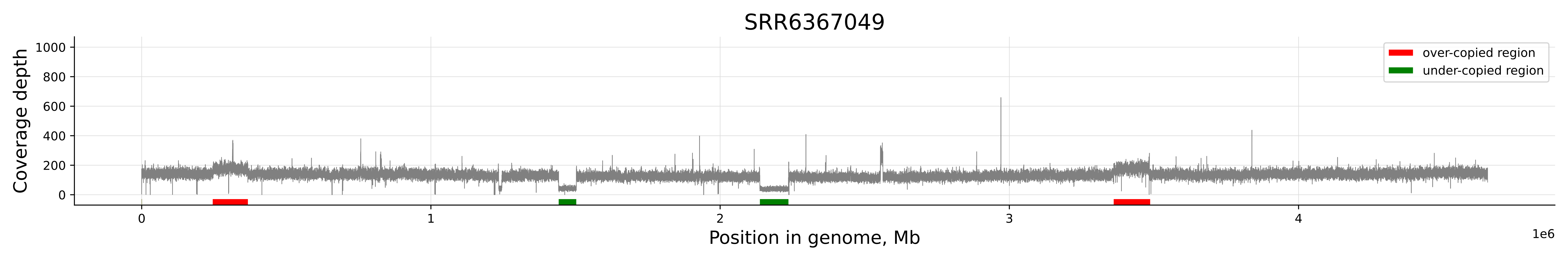
SRR6367049
Coverage depth
Position in genome, Mb
over-copied region
under-copied region
1000
800
600
400
200
0
0
1
2
3
4
1e6

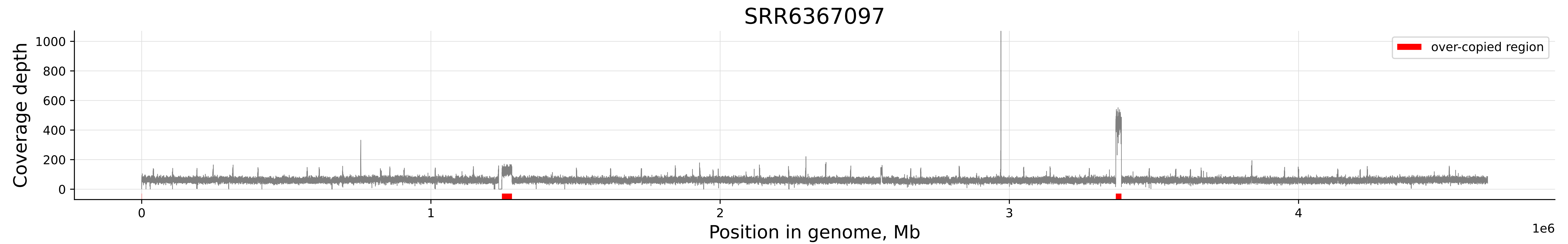

SRR6367097
Coverage depth
1000
800
600
400
200
0
over-copied region
0
1
2
3
4
1e6
Position in genome, Mb

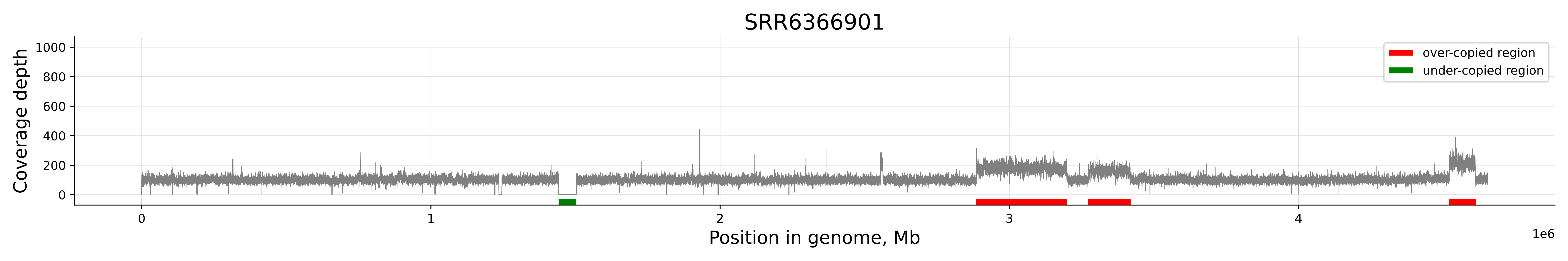
SRR6366901
Coverage depth
1000
800
600
400
200
0
0
1
2
3
4
1e6
Position in genome, Mb
over-copied region
under-copied region

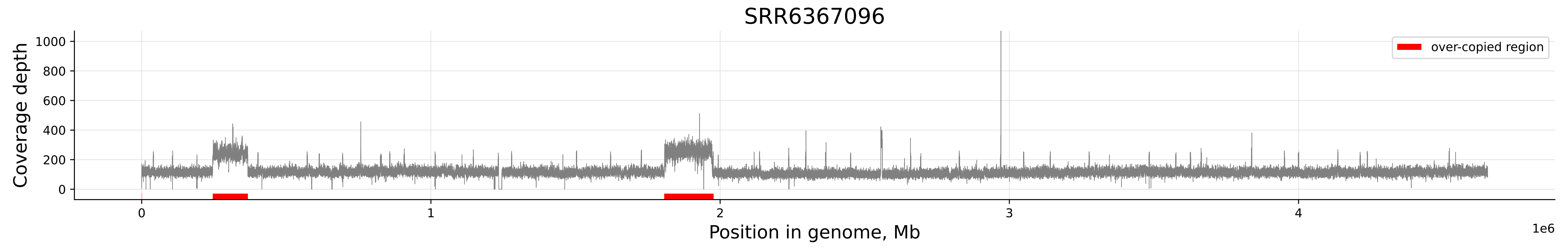

SRR6367096
over-copied region
Coverage depth
1000
800
600
400
200
0
0
1
2
3
4
1e6
Position in genome, Mb

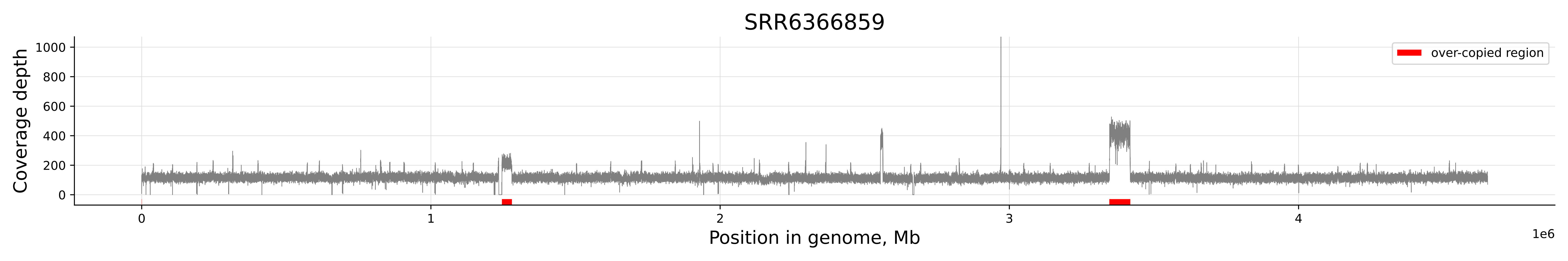
SRR6366859
Coverage depth
1000
800
600
400
200
0
over-copied region
0
1
2
3
4
1e6
Position in genome, Mb

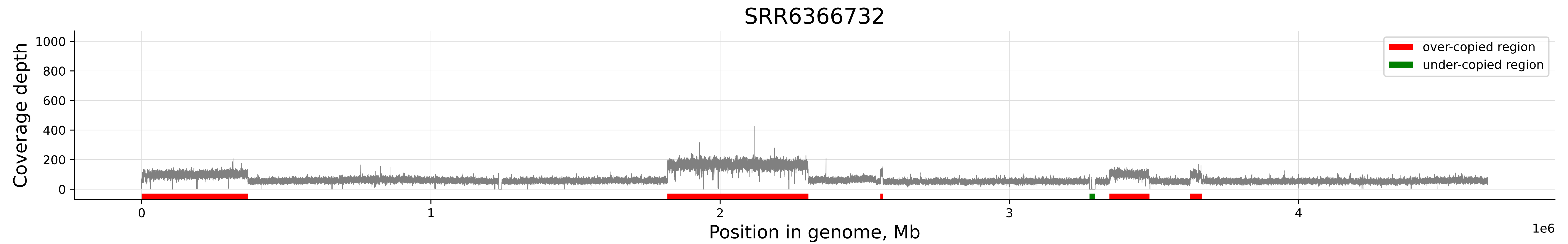
SRR6366732
Coverage depth
1000
800
600
400
200
0
over-copied region
under-copied region
0
1
2
3
4
1e6
Position in genome, Mb

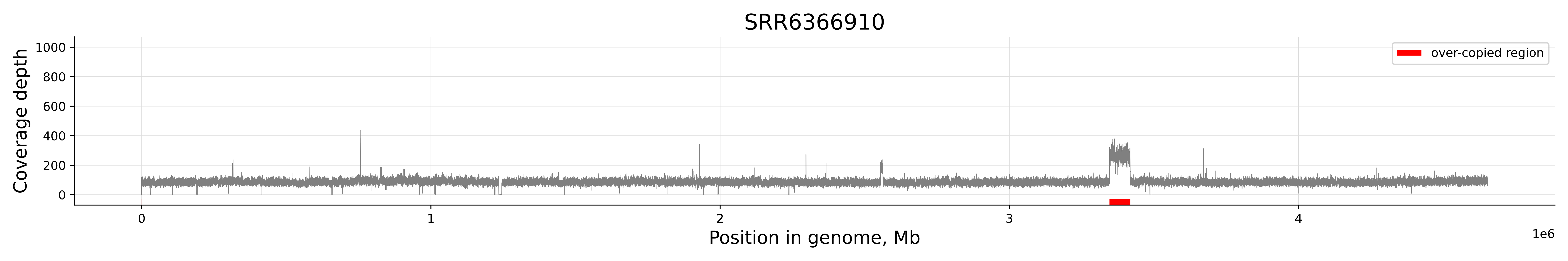
SRR6366910
Coverage depth
Position in genome, Mb
1e6
over-copied region
0
200
400
600
800
1000
1
2
3
4

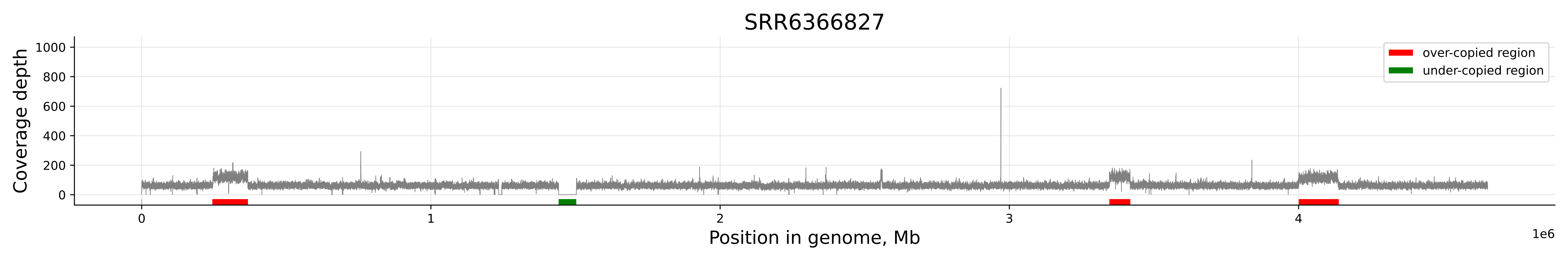
SRR6366827
Coverage depth
0
200
400
600
800
1000
over-copied region
under-copied region
0
1
2
3
4
Position in genome, Mb
1e6

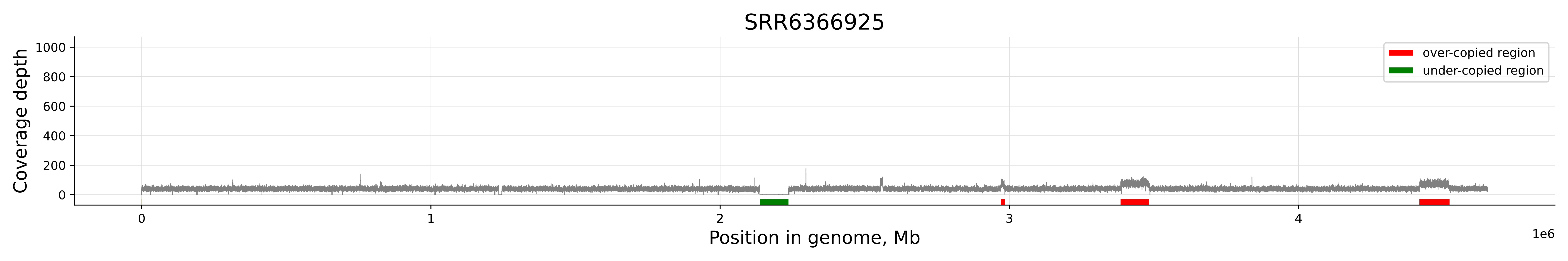
SRR6366925
over-copied region
under-copied region
Coverage depth
0
200
400
600
800
1000
0
1
2
3
4
1e6
Position in genome, Mb

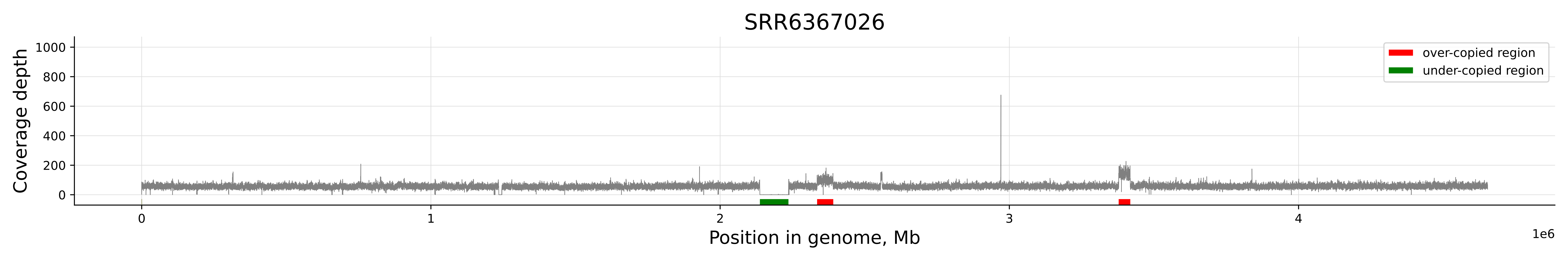

SRR6367026
Coverage depth
Position in genome, Mb
1e6
over-copied region
under-copied region
0
200
400
600
800
1000
0
1
2
3
4

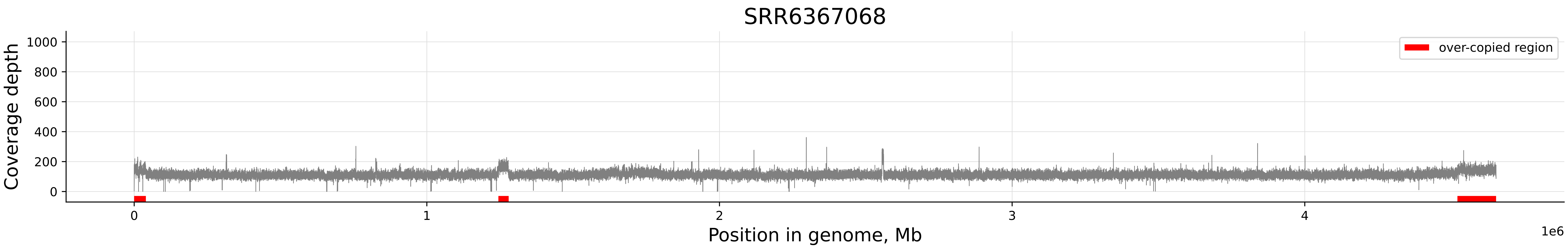
SRR6367068
Coverage depth
Position in genome, Mb
over-copied region
1000
800
600
400
200
0
0
1
2
3
4
1e6

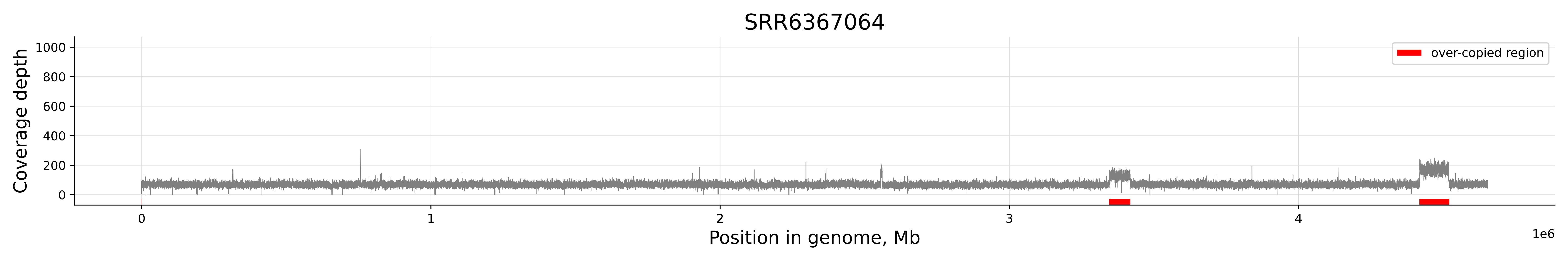

SRR6367064
Coverage depth
1000
800
600
400
200
0
0
1
2
3
4
Position in genome, Mb
1e6
over-copied region

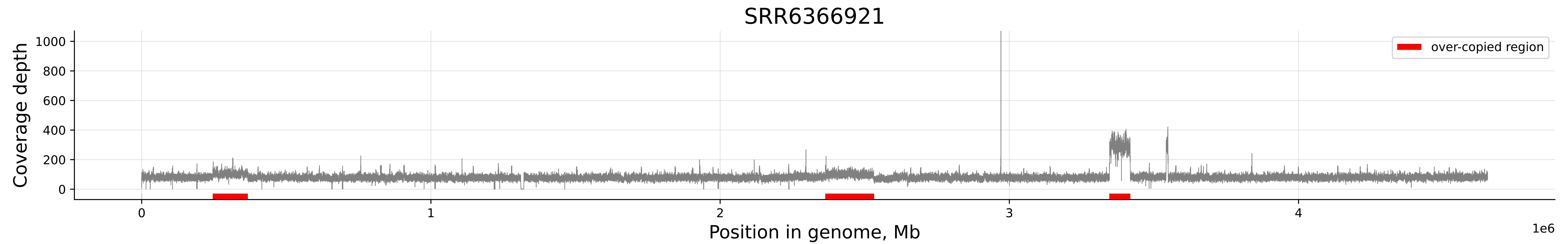
SRR6366921
Coverage depth
Position in genome, Mb
over-copied region
1e6
0
200
400
600
800
1000
0
1
2
3
4

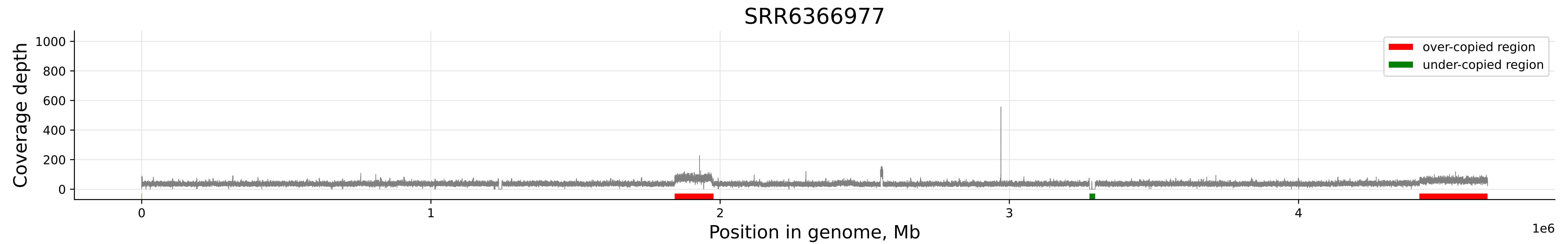
SRR6366977
Coverage depth
Position in genome, Mb
1e6
over-copied region
under-copied region
0
200
400
600
800
1000
0
1
2
3
4

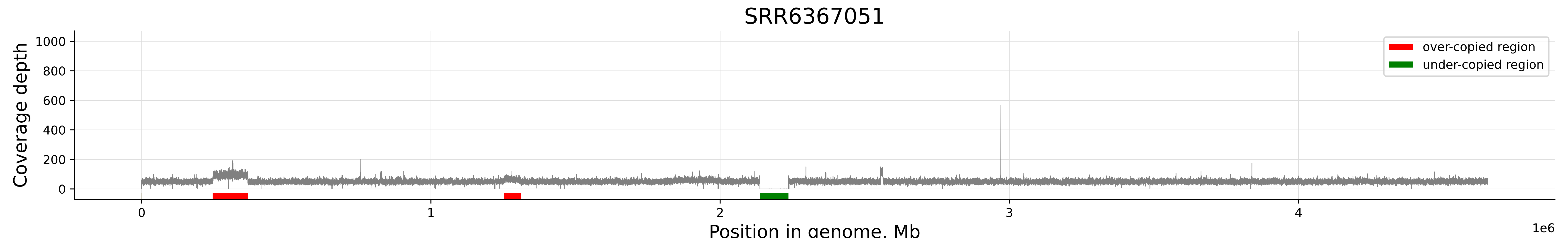

SRR6367051
Coverage depth
Position in genome, Mb
1e6
over-copied region
under-copied region
0
200
400
600
800
1000
0
1
2
3
4

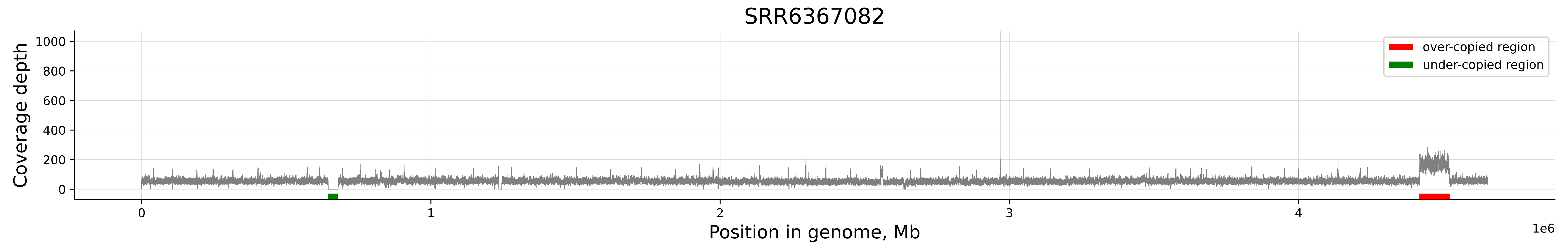
SRR6367082
Coverage depth
1000
800
600
400
200
0
0
1
2
3
4
1e6
Position in genome, Mb
over-copied region
under-copied region

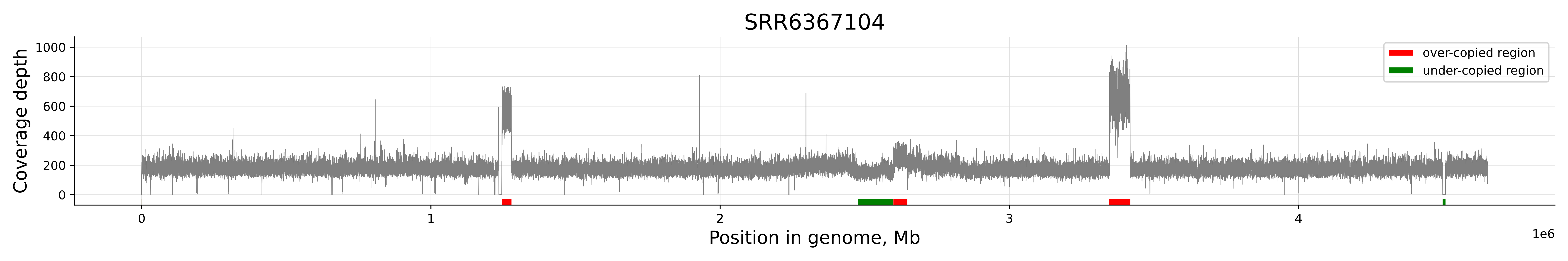

SRR6367104
over-copied region
under-copied region
Coverage depth
0
200
400
600
800
1000
0
1
2
3
4
1e6
Position in genome, Mb

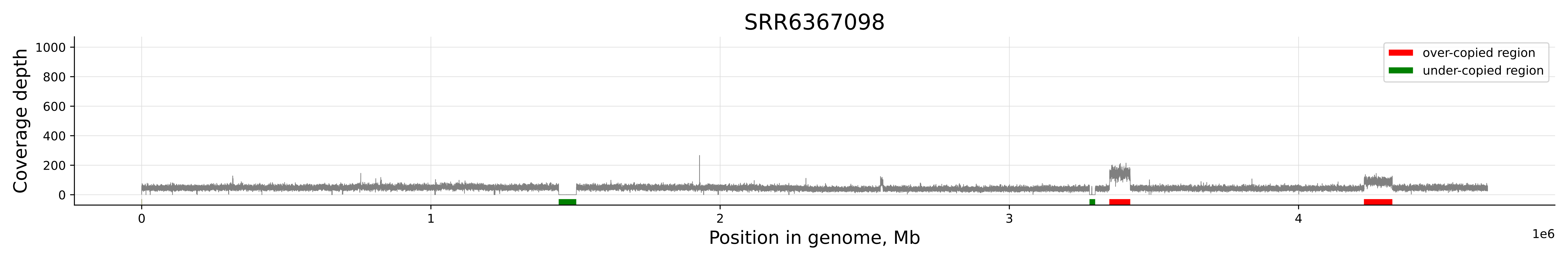

SRR6367098
Coverage depth
Position in genome, Mb
1e6
over-copied region
under-copied region
0
200
400
600
800
1000
0
1
2
3
4

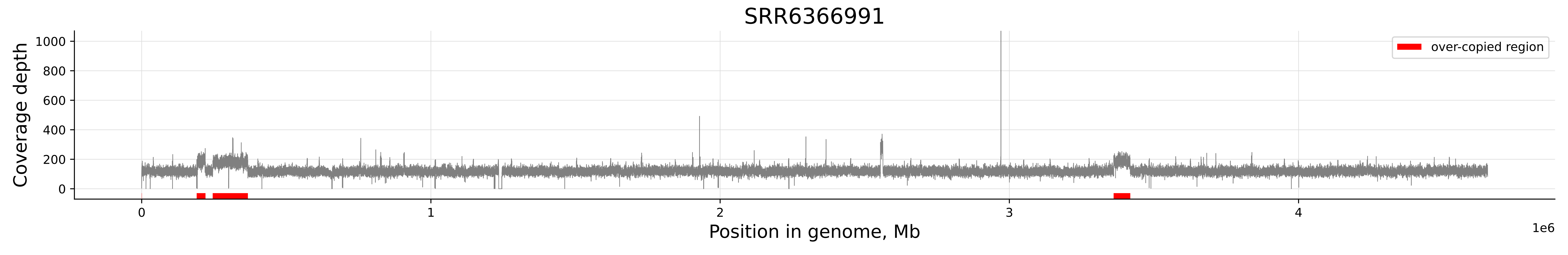

SRR6366991
over-copied region
Coverage depth
0
200
400
600
800
1000
0
1
2
3
4
1e6
Position in genome, Mb

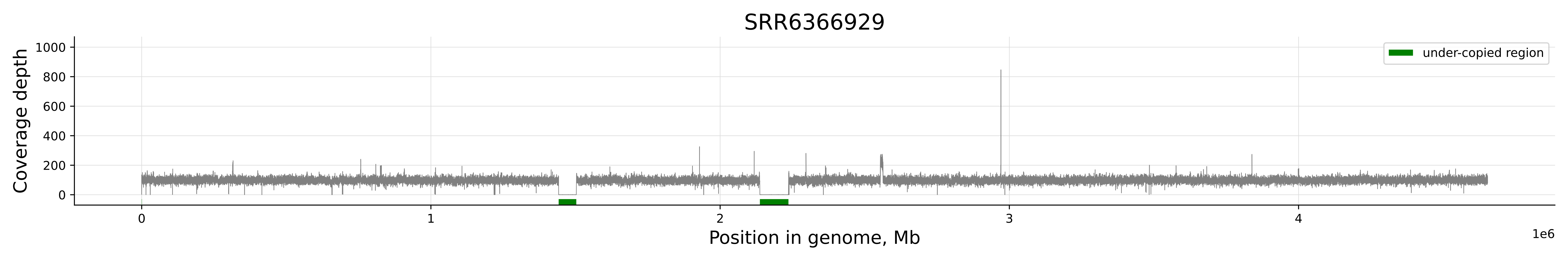

SRR6366929
under-copied region
Coverage depth
1000
800
600
400
200
0
0
1
2
3
4
1e6
Position in genome, Mb

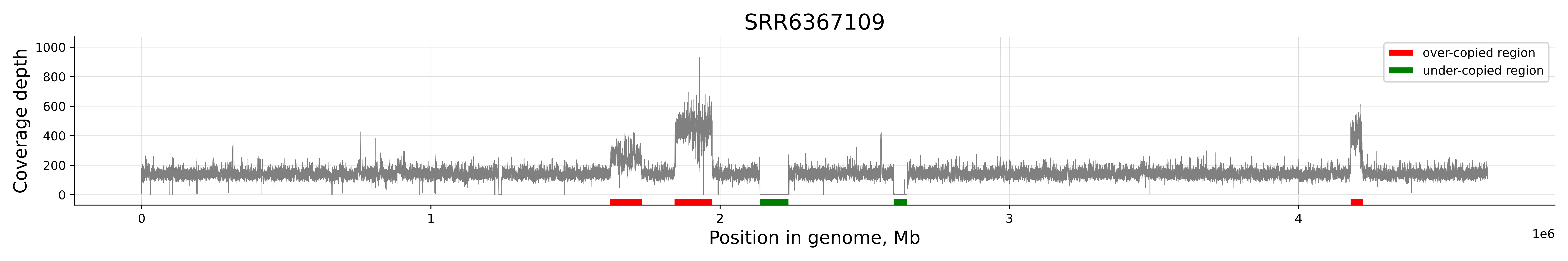

SRR6367109
Coverage depth
Position in genome, Mb
over-copied region
under-copied region
1e6
0
200
400
600
800
1000
0
1
2
3
4

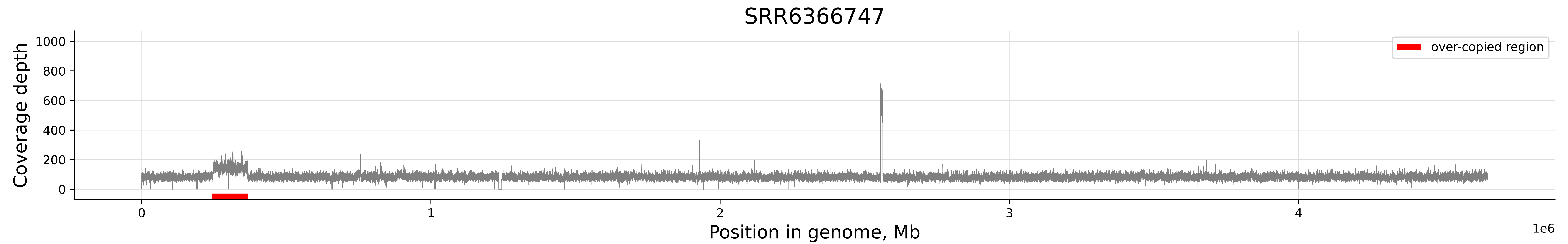
SRR6366747
Coverage depth
Position in genome, Mb
over-copied region
0
200
400
600
800
1000
0
1
2
3
4
1e6

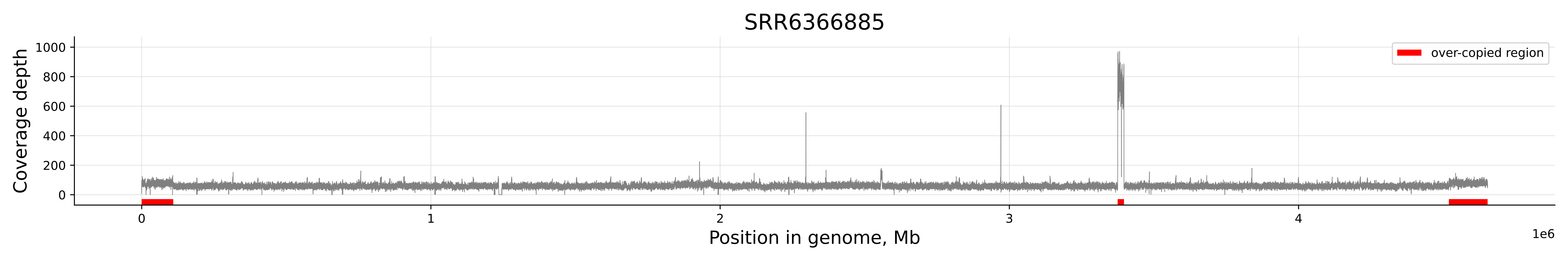
SRR6366885
Coverage depth
Position in genome, Mb
1e6
over-copied region
0
200
400
600
800
1000
0
1
2
3
4

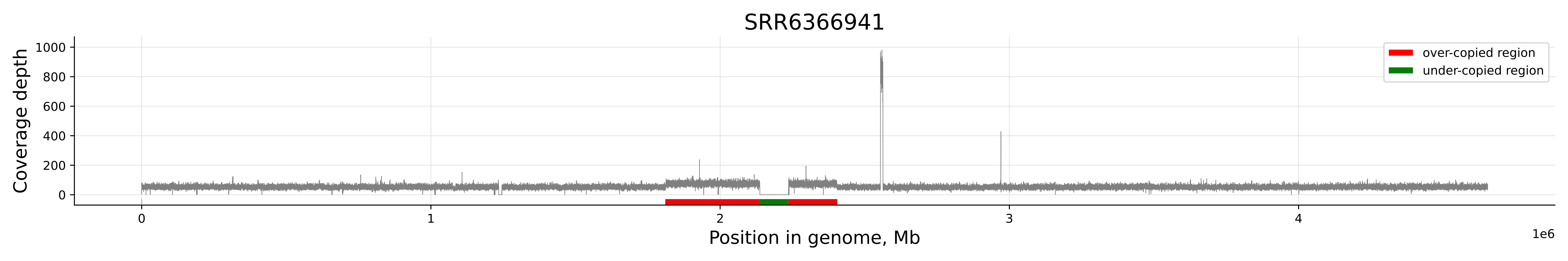

SRR6366941
Coverage depth
Position in genome, Mb
over-copied region
under-copied region
1000
800
600
400
200
0
0
1
2
3
4
1e6

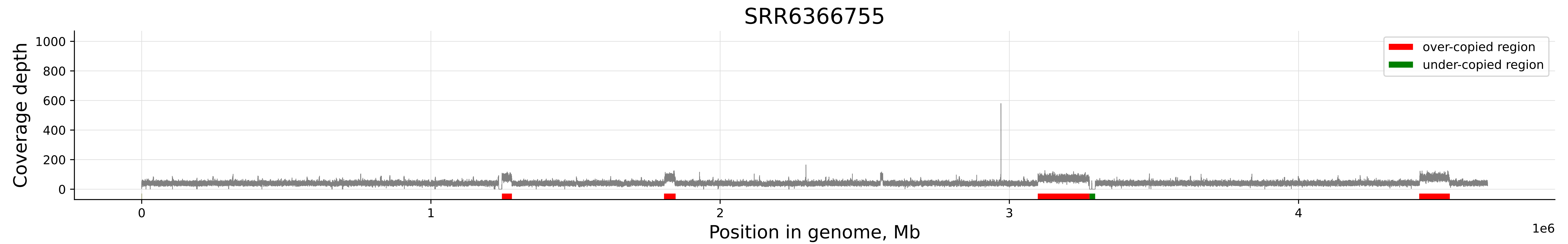

SRR6366755
Coverage depth
1000
800
600
400
200
0
over-copied region
under-copied region
0
1
2
3
4
1e6
Position in genome, Mb

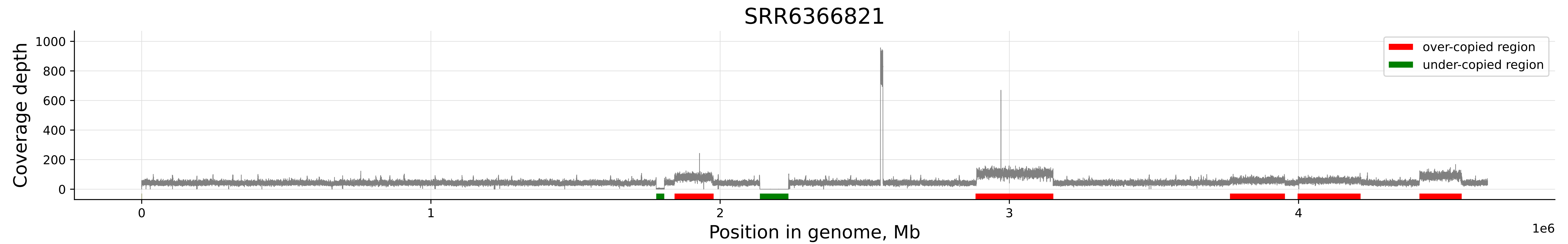
SRR6366821
Coverage depth
1000
800
600
400
200
0
0
1
2
3
4
1e6
Position in genome, Mb
over-copied region
under-copied region

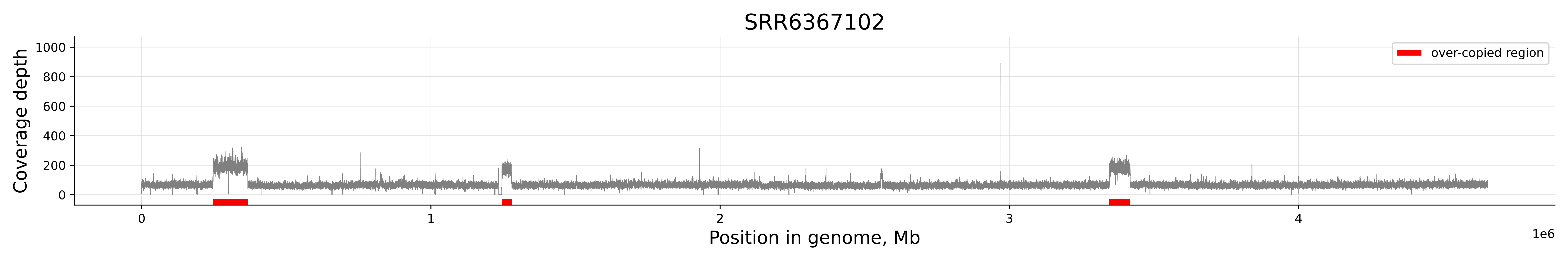
SRR6367102
Coverage depth
1000
800
600
400
200
0
over-copied region
0
1
2
3
4
1e6
Position in genome, Mb

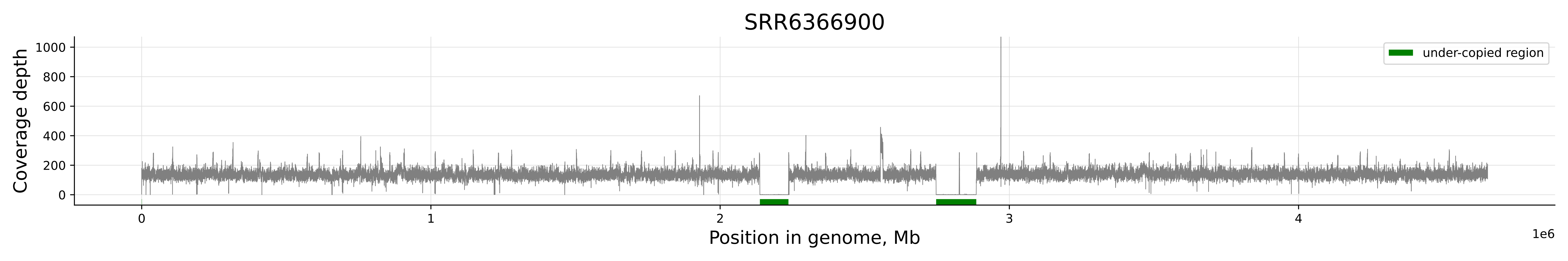
SRR6366900
Coverage depth
Position in genome, Mb
1e6
under-copied region
0
200
400
600
800
1000
0
1
2
3
4

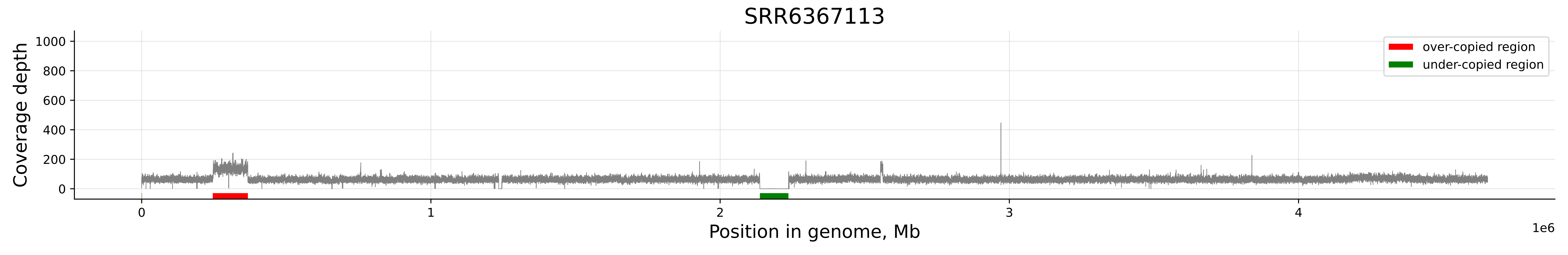
SRR6367113
Coverage depth
1000
800
600
400
200
0
0
1
2
3
4
1e6
Position in genome, Mb
over-copied region
under-copied region

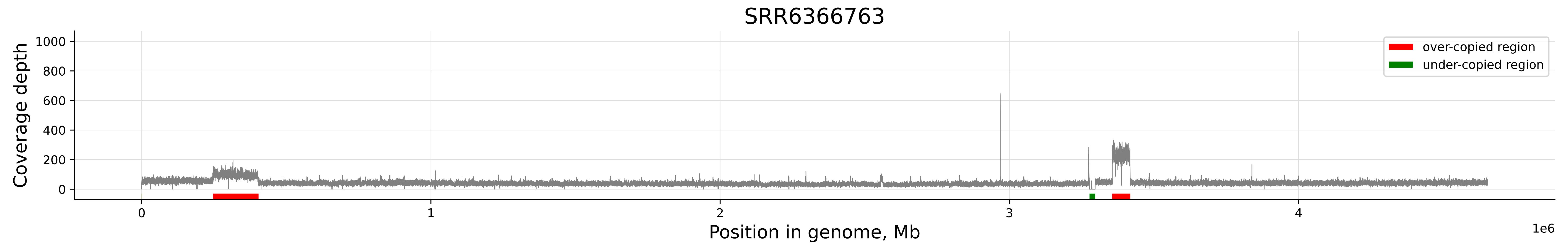

SRR6366763
Coverage depth
1000
800
600
400
200
0
over-copied region
under-copied region
0
1
2
3
4
1e6
Position in genome, Mb

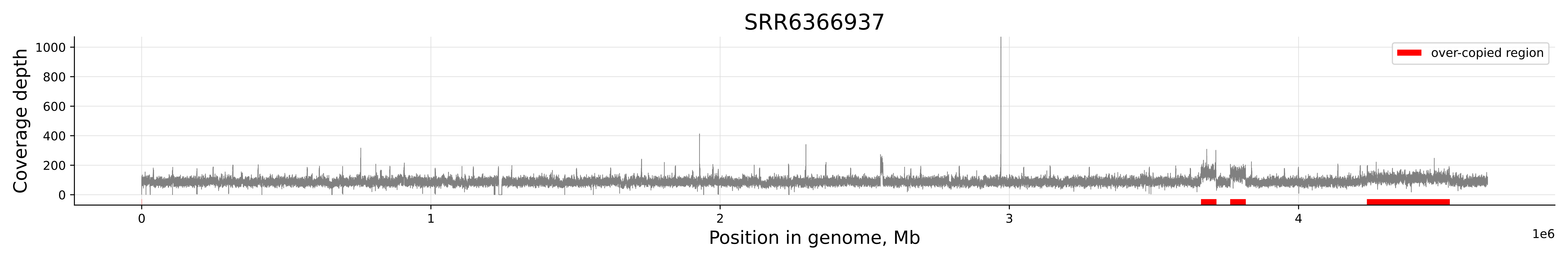

SRR6366937
over-copied region
Coverage depth
0
200
400
600
800
1000
0
1
2
3
4
1e6
Position in genome, Mb

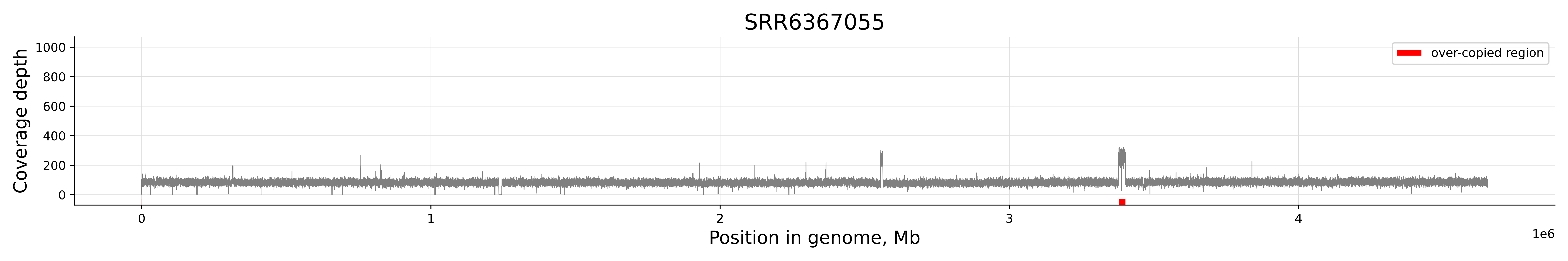
SRR6367055
Coverage depth
1000
800
600
400
200
0
over-copied region
0
1
2
3
4
1e6
Position in genome, Mb

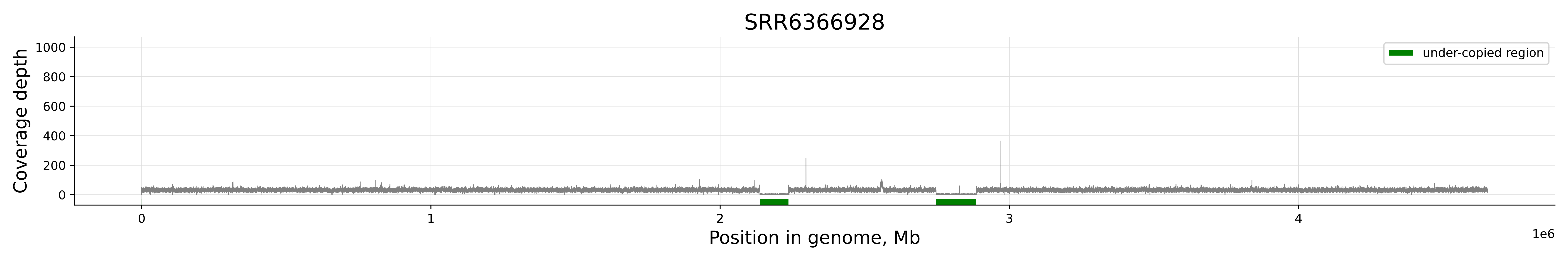
SRR6366928
under-copied region
Coverage depth
1000
800
600
400
200
0
0
1
2
3
4
1e6
Position in genome, Mb

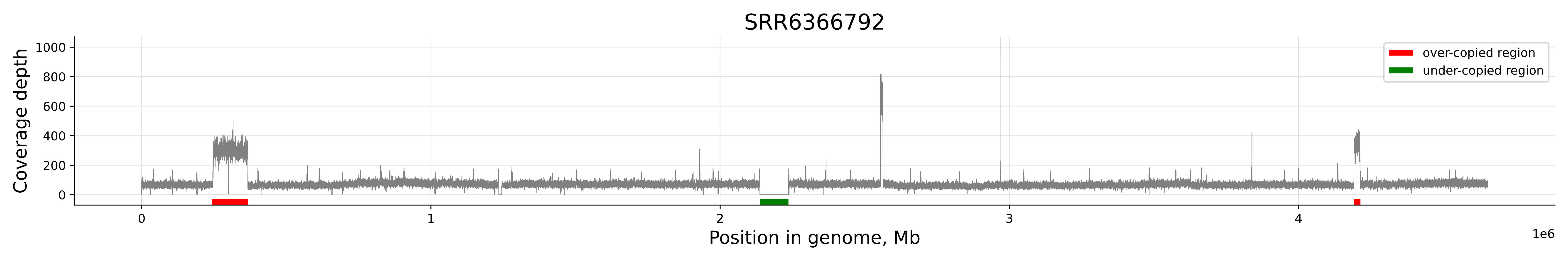

SRR6366792
Coverage depth
1000
800
600
400
200
0
0
1
2
3
4
1e6
Position in genome, Mb
over-copied region
under-copied region

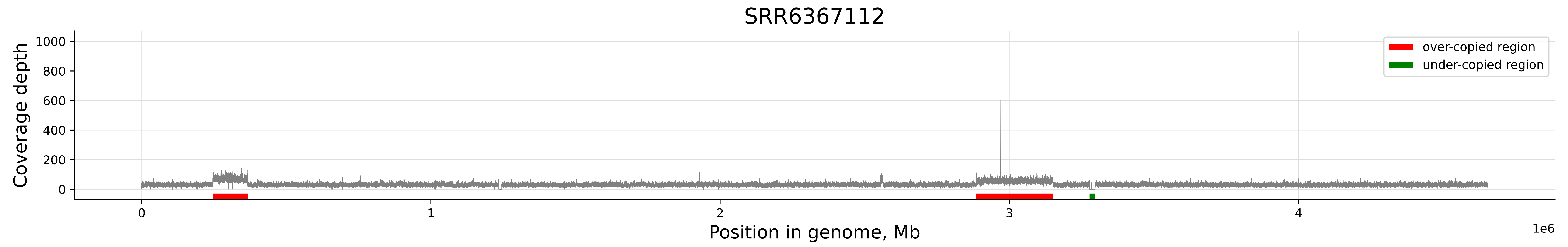

SRR6367112
Coverage depth
1000
800
600
400
200
0
0
1
2
3
4
1e6
Position in genome, Mb
over-copied region
under-copied region

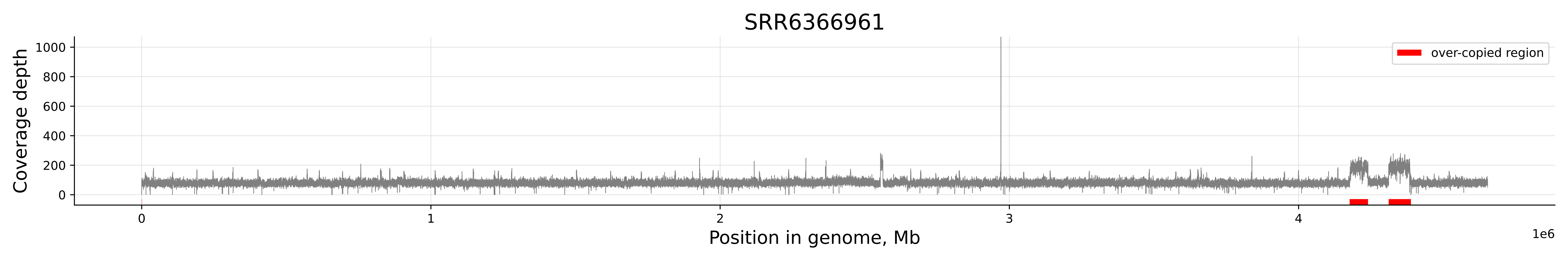
SRR6366961
Coverage depth
1000
800
600
400
200
0
over-copied region
0
1
2
3
4
1e6
Position in genome, Mb

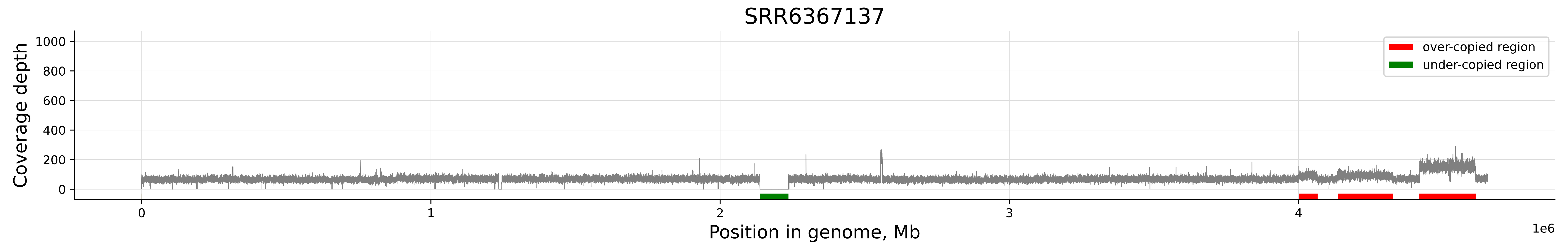

SRR6367137
over-copied region
under-copied region
Coverage depth
Position in genome, Mb
1e6
0
200
400
600
800
1000
0
1
2
3
4

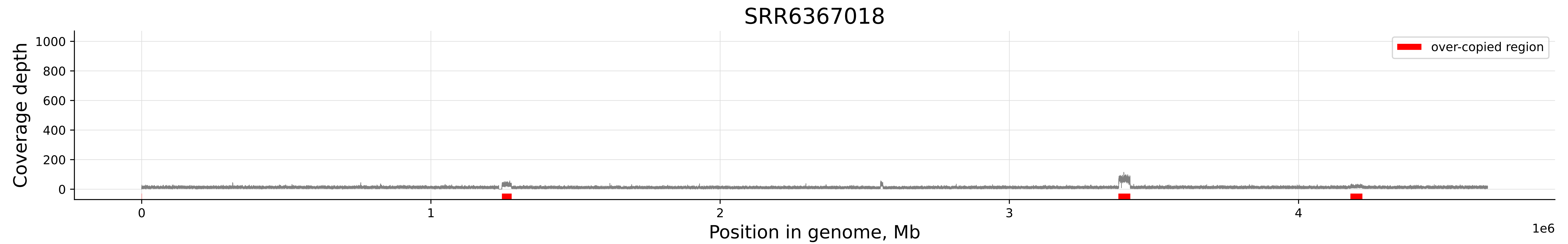

SRR6367018
Coverage depth
1000
800
600
400
200
0
0
1
2
3
4
1e6
Position in genome, Mb
over-copied region

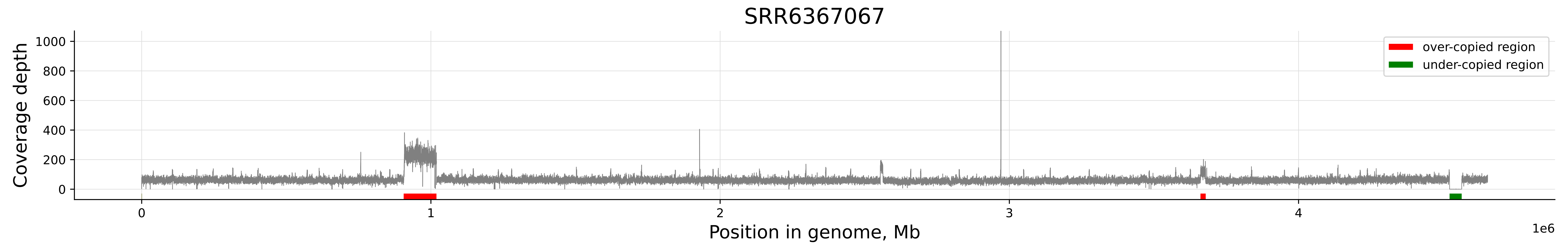
SRR6367067
Coverage depth
1000
800
600
400
200
0
over-copied region
under-copied region
0
1
2
3
4
1e6
Position in genome, Mb

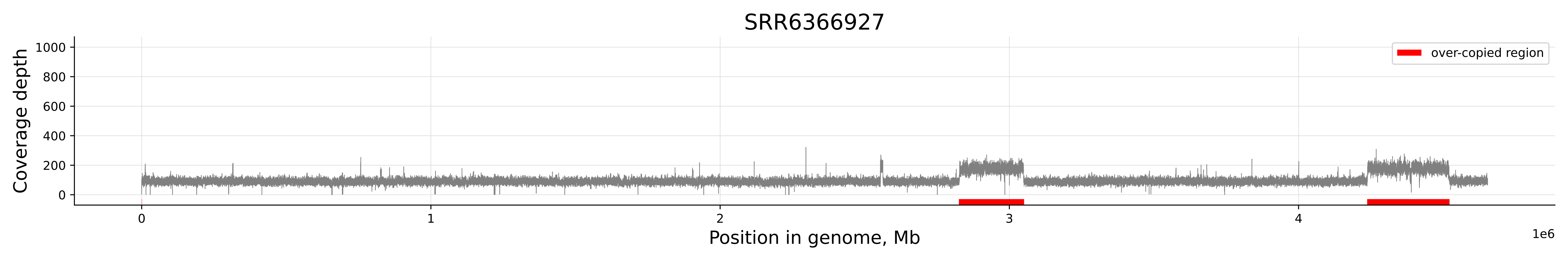
SRR6366927
Coverage depth
1000
800
600
400
200
0
0
1
2
3
4
1e6
Position in genome, Mb
over-copied region

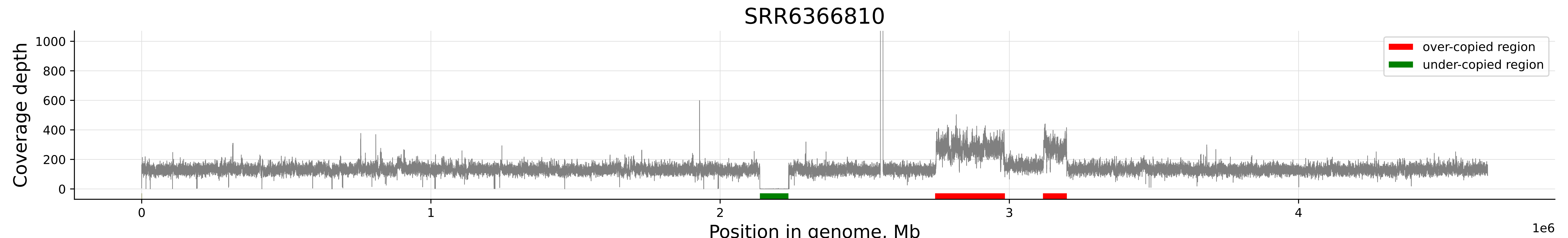
SRR6366810
Coverage depth
Position in genome, Mb
over-copied region
under-copied region
1000
800
600
400
200
0
0
1
2
3
4
1e6

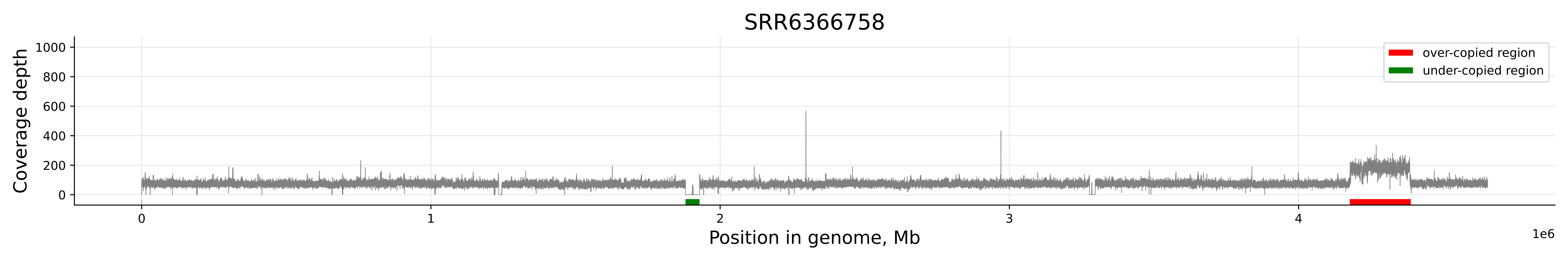

SRR6366758
Coverage depth
Position in genome, Mb
over-copied region
under-copied region
1e6

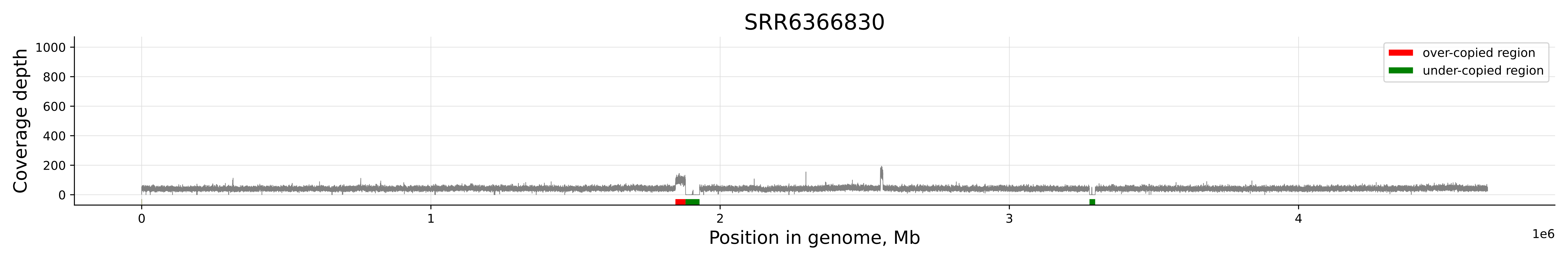

SRR6366830
Coverage depth
Position in genome, Mb
1e6
over-copied region
under-copied region
0
200
400
600
800
1000
0
1
2
3
4

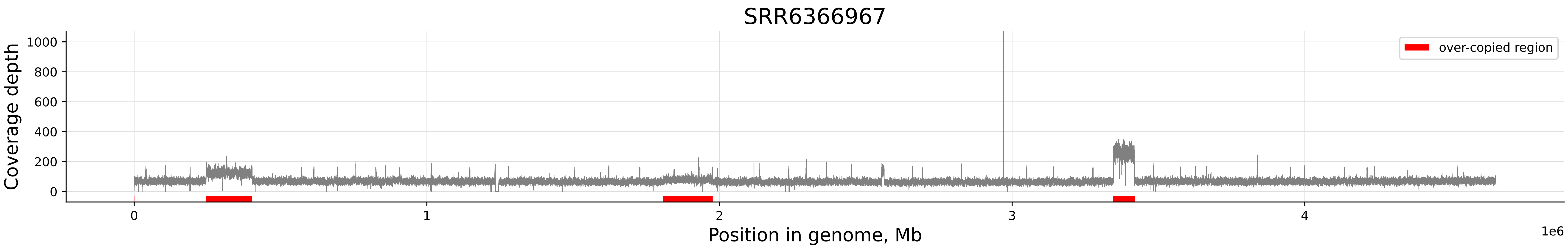

SRR6366967
Coverage depth
1000
800
600
400
200
0
over-copied region
0
1
2
3
4
1e6
Position in genome, Mb

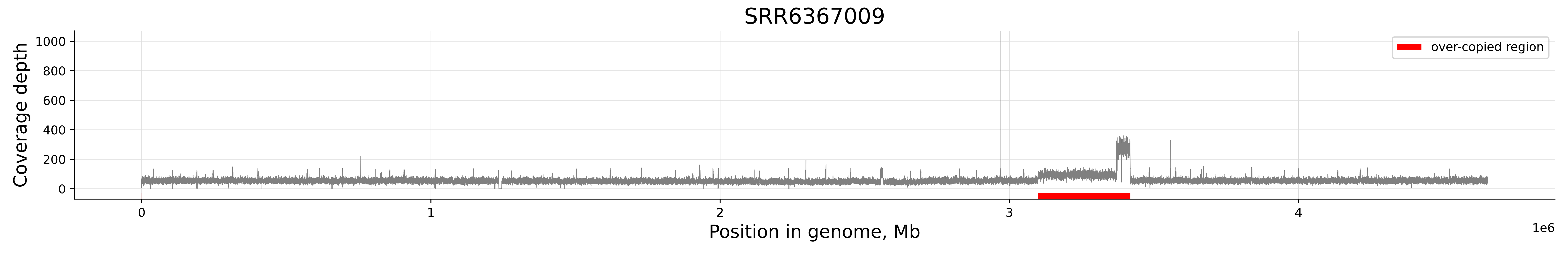

SRR6367009
over-copied region
Coverage depth
1000
800
600
400
200
0
0
1
2
3
4
1e6
Position in genome, Mb

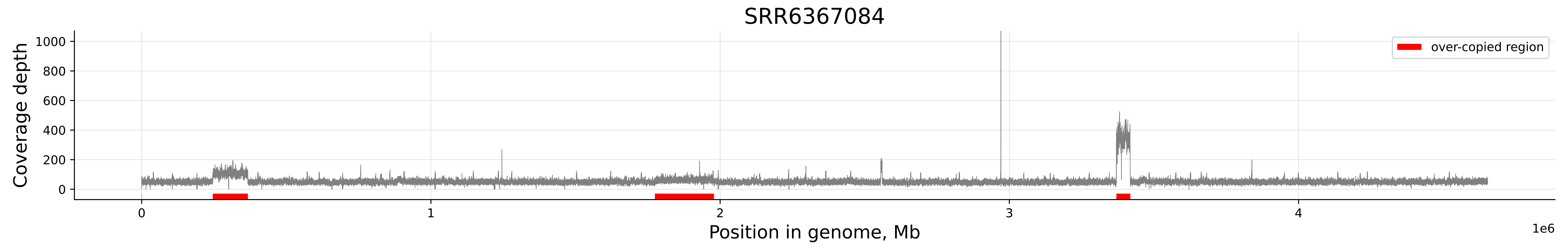
SRR6367084
Coverage depth
1000
800
600
400
200
0
over-copied region
0
1
2
3
4
1e6
Position in genome, Mb

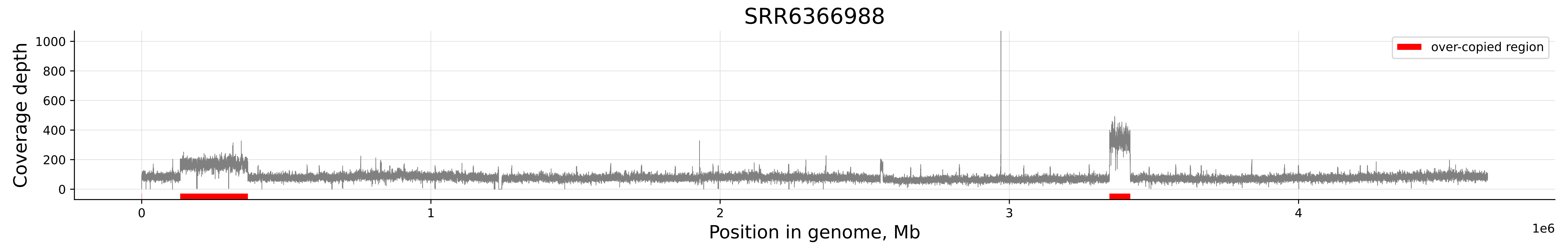

SRR6366988
Coverage depth
1000
800
600
400
200
0
over-copied region
0
1
2
3
4
1e6
Position in genome, Mb

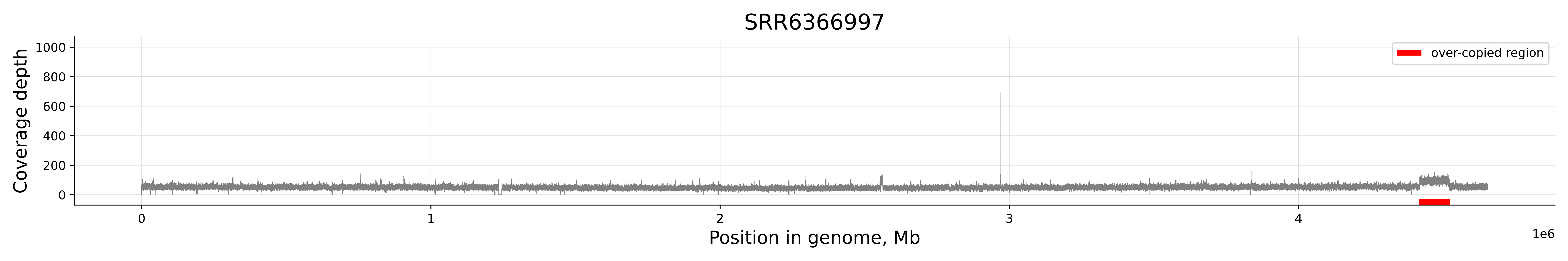

SRR6366997
Coverage depth
Position in genome, Mb
over-copied region
1000
800
600
400
200
0
0
1
2
3
4
1e6

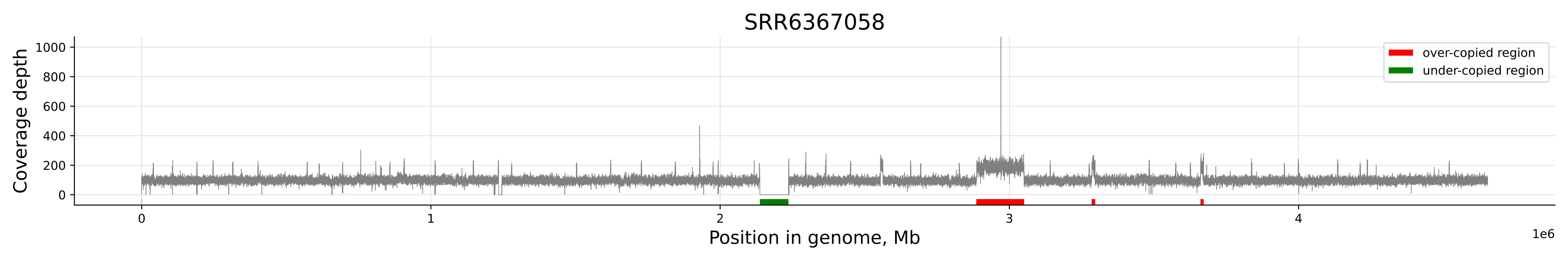

SRR6367058
Coverage depth
1000
800
600
400
200
0
over-copied region
under-copied region
0
1
2
3
4
1e6
Position in genome, Mb

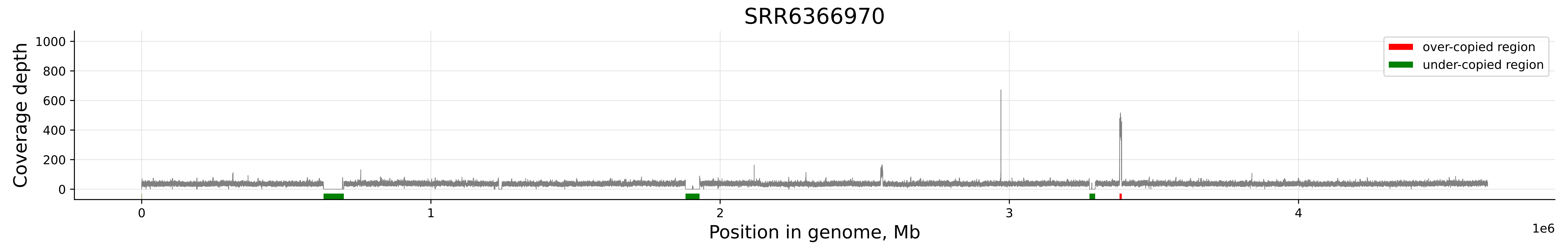
SRR6366970
Coverage depth
Position in genome, Mb
1e6
over-copied region
under-copied region
0
200
400
600
800
1000
0
1
2
3
4

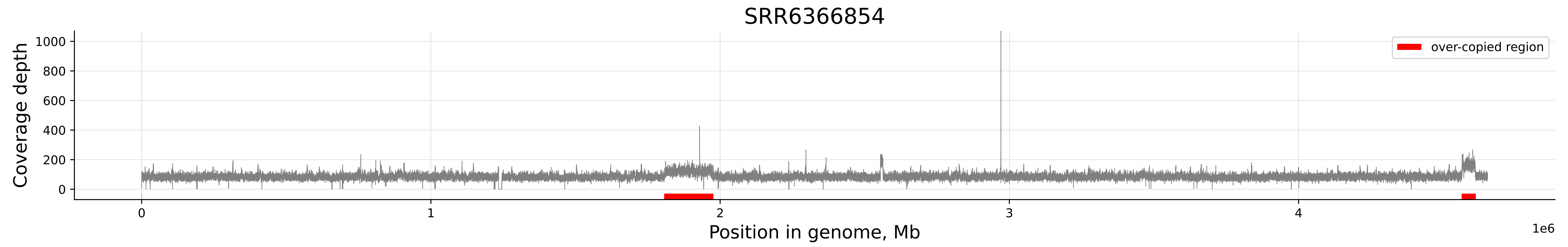

SRR6366854
Coverage depth
1000
800
600
400
200
0
over-copied region
0
1
2
3
4
1e6
Position in genome, Mb

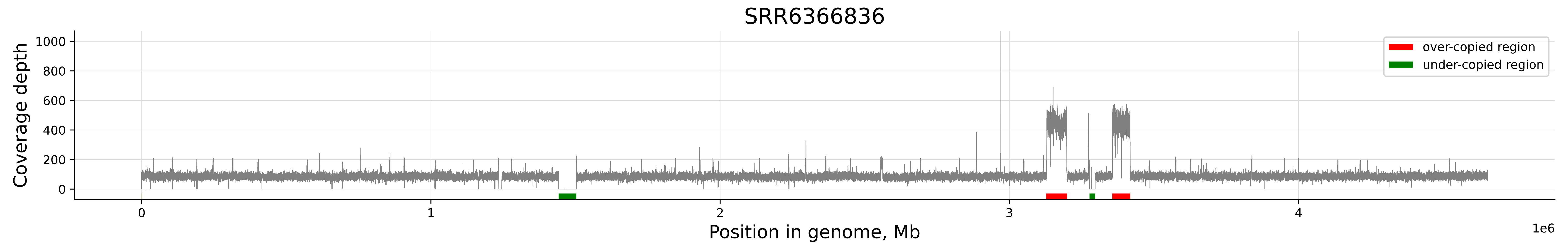

SRR6366836
Coverage depth
1000
800
600
400
200
0
over-copied region
under-copied region
0
1
2
3
4
Position in genome, Mb
1e6

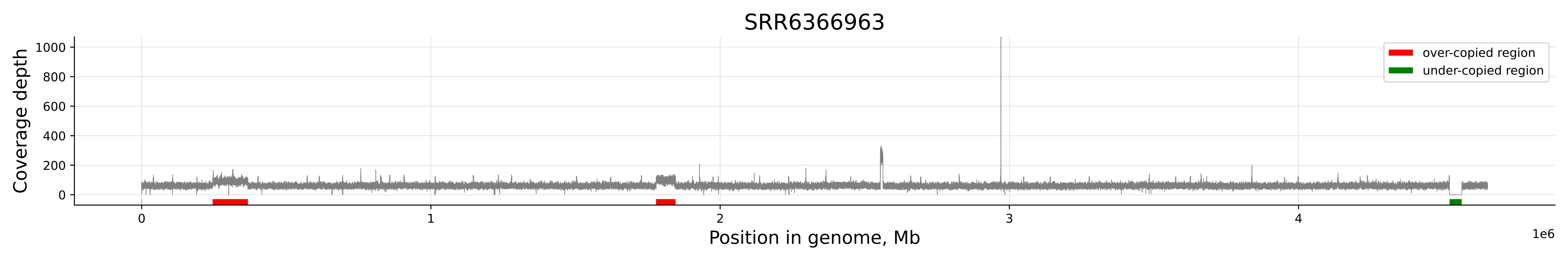
SRR6366963
Coverage depth
1000
800
600
400
200
0
0
1
2
3
4
1e6
Position in genome, Mb
over-copied region
under-copied region

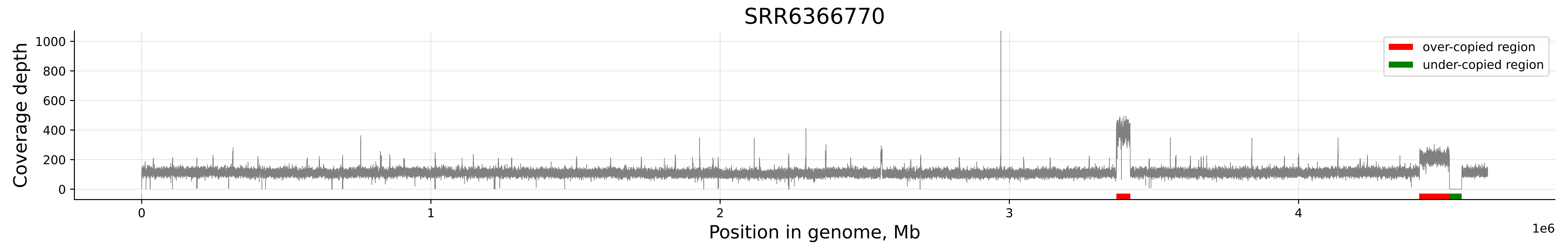

SRR6366770
Coverage depth
Position in genome, Mb
1e6
over-copied region
under-copied region
0
200
400
600
800
1000
0
1
2
3
4

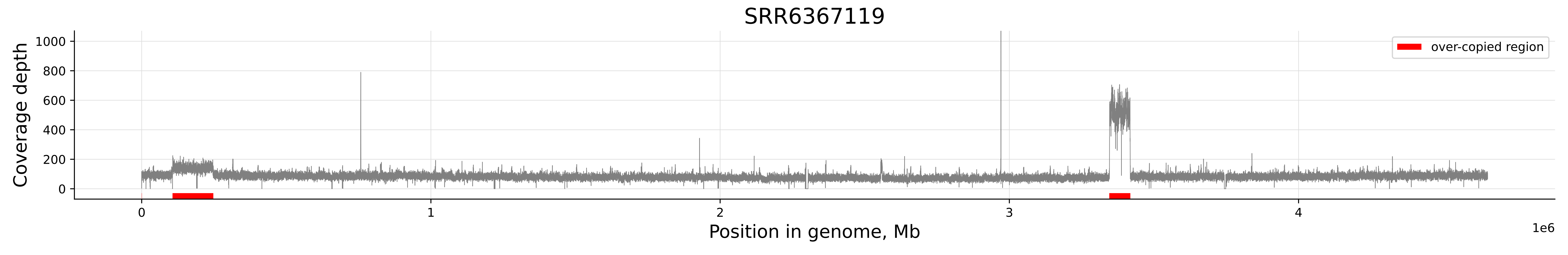

SRR6367119
Coverage depth
Position in genome, Mb
over-copied region
1e6

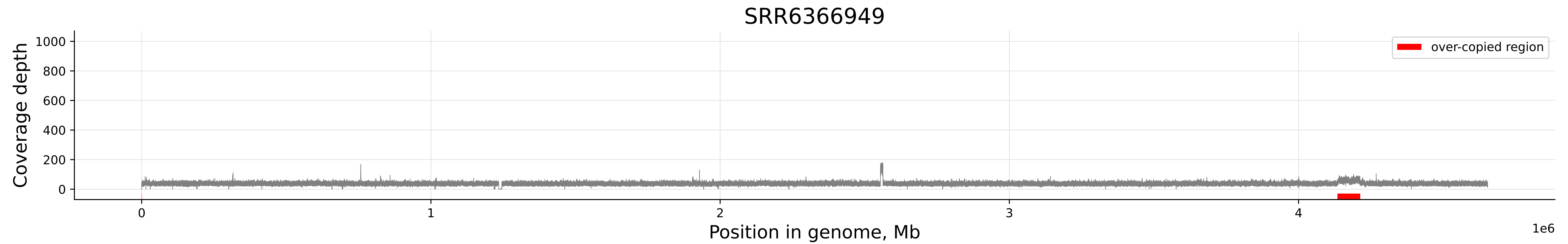

SRR6366949
over-copied region
Coverage depth
1000
800
600
400
200
0
0
1
2
3
4
1e6
Position in genome, Mb

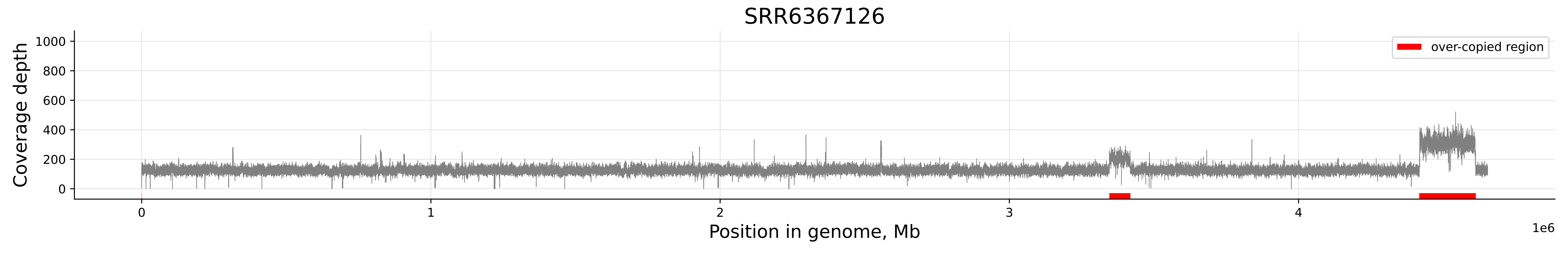

SRR6367126
over-copied region
Coverage depth
1000
800
600
400
200
0
0
1
2
3
4
1e6
Position in genome, Mb

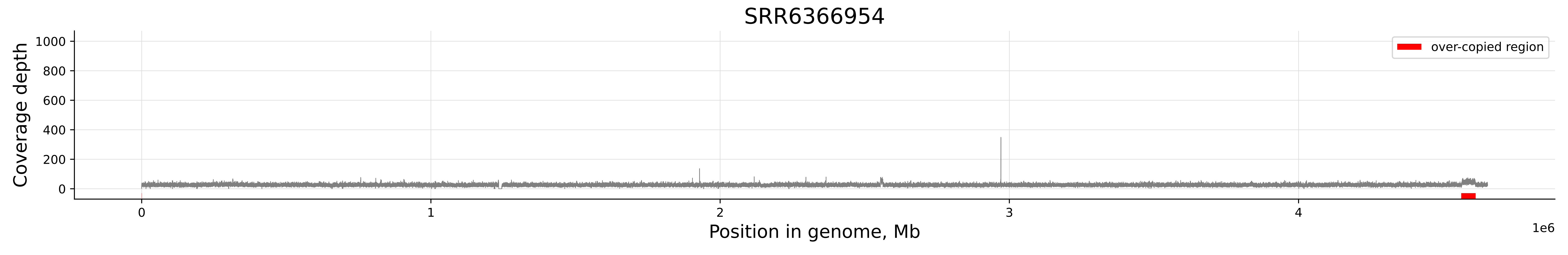

SRR6366954
over-copied region
Coverage depth
0
200
400
600
800
1000
0
1
2
3
4
1e6
Position in genome, Mb

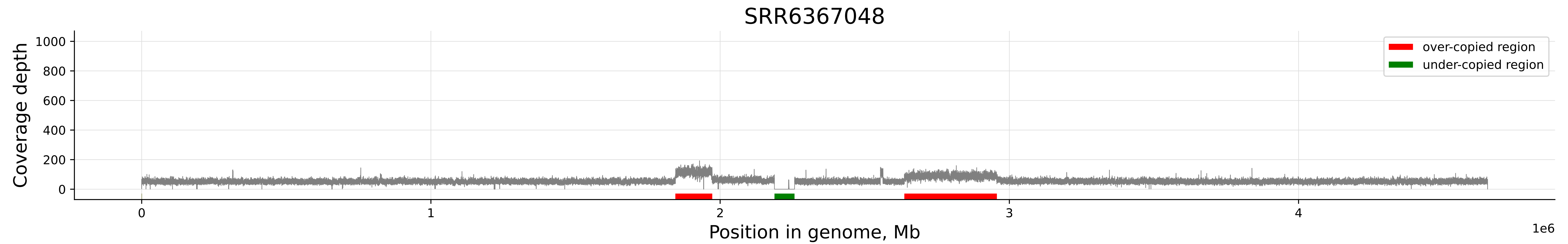

SRR6367048
Coverage depth
1000
800
600
400
200
0
over-copied region
under-copied region
0
1
2
3
4
1e6
Position in genome, Mb

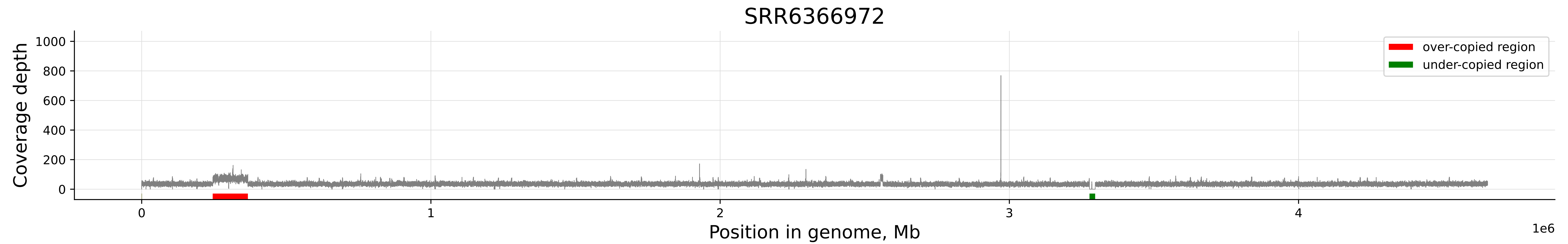

SRR6366972
Coverage depth
1000
800
600
400
200
0
over-copied region
under-copied region
0
1
2
3
4
1e6
Position in genome, Mb

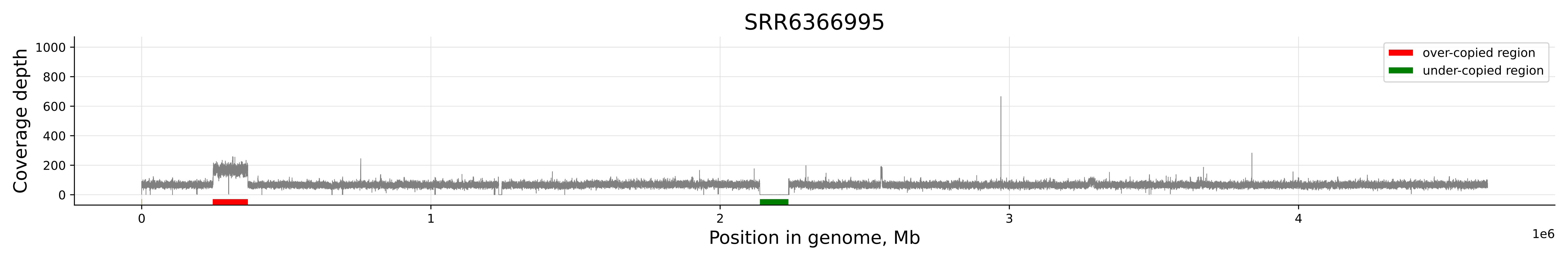
SRR6366995
Coverage depth
1000
800
600
400
200
0
0
1
2
3
4
1e6
Position in genome, Mb
over-copied region
under-copied region

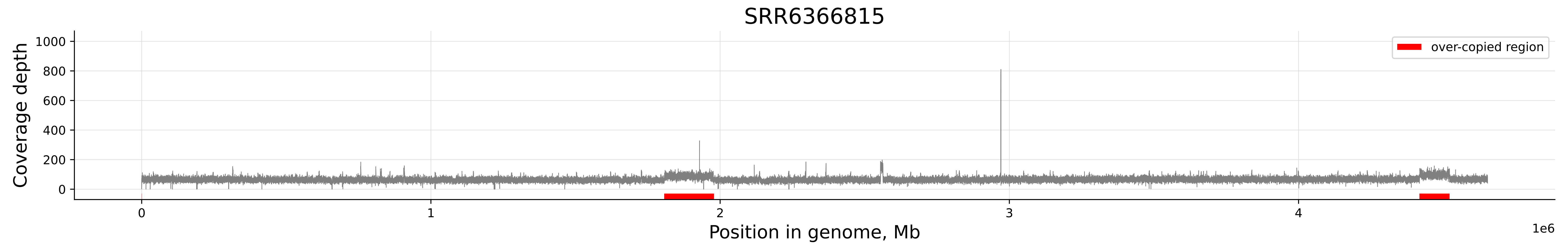
SRR6366815
over-copied region
Coverage depth
0
200
400
600
800
1000
0
1
2
3
4
1e6
Position in genome, Mb

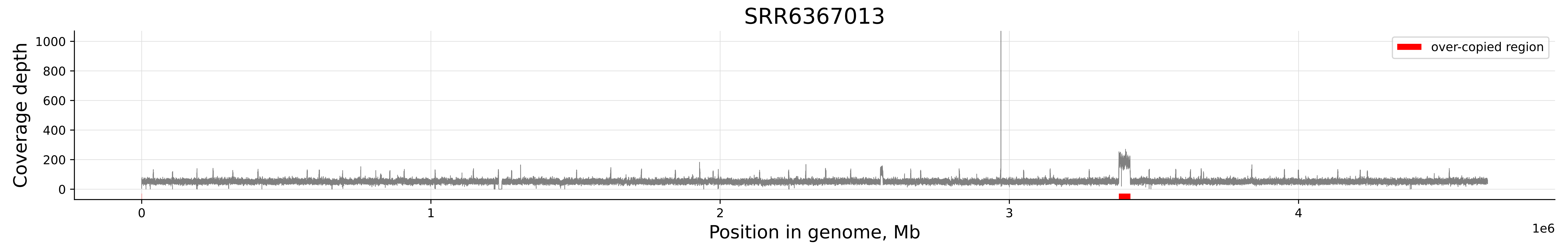

SRR6367013
over-copied region
Coverage depth
1000
800
600
400
200
0
0
1
2
3
4
1e6
Position in genome, Mb

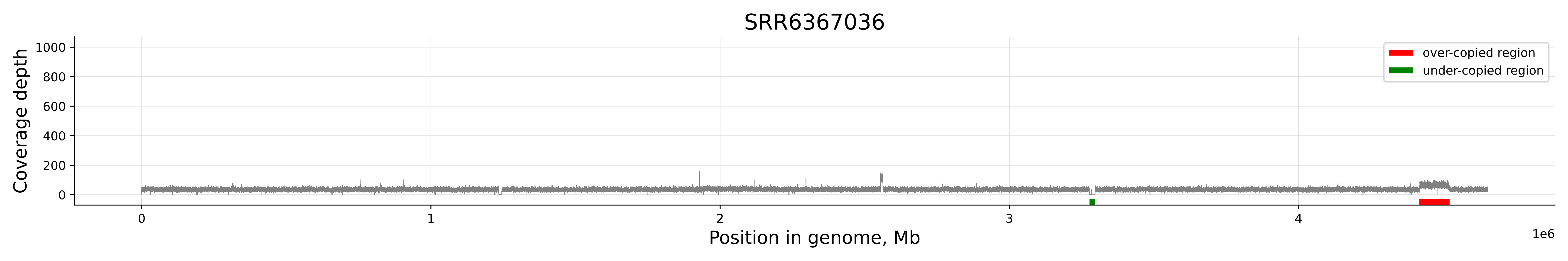

SRR6367036
Coverage depth
1000
800
600
400
200
0
0
1
2
3
4
1e6
Position in genome, Mb
over-copied region
under-copied region

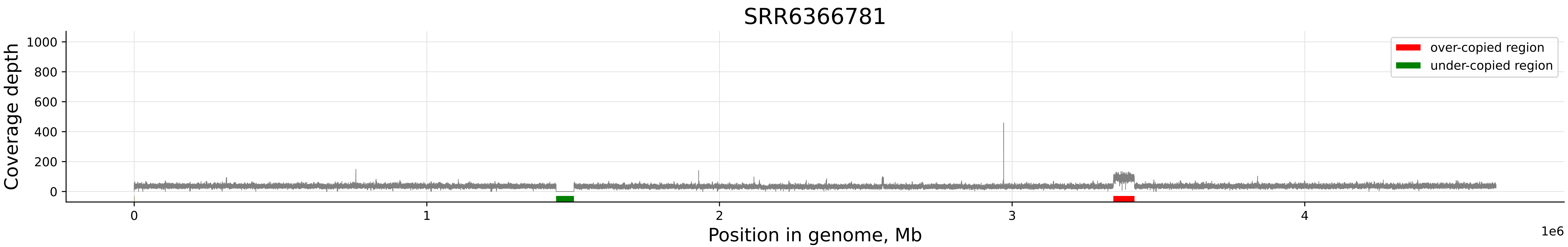

SRR6366781
Coverage depth
Position in genome, Mb
1e6
over-copied region
under-copied region
0
200
400
600
800
1000
0
1
2
3
4

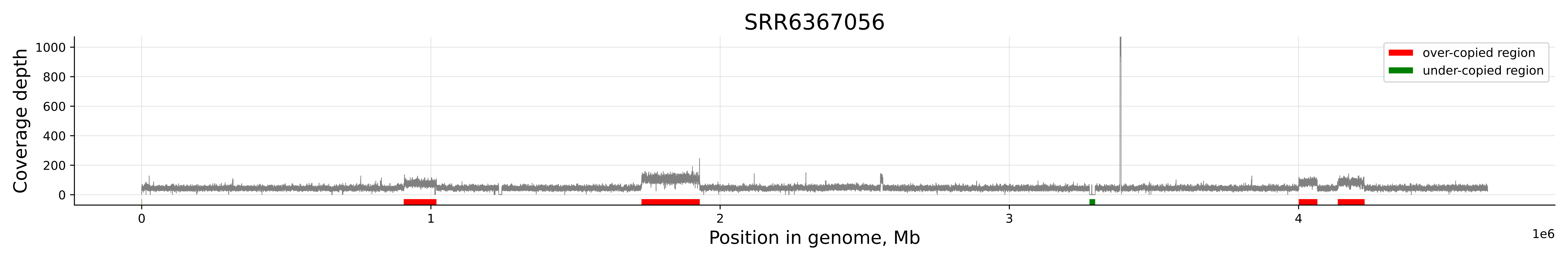

SRR6367056
Coverage depth
0
200
400
600
800
1000
0
1
2
3
4
1e6
Position in genome, Mb
over-copied region
under-copied region

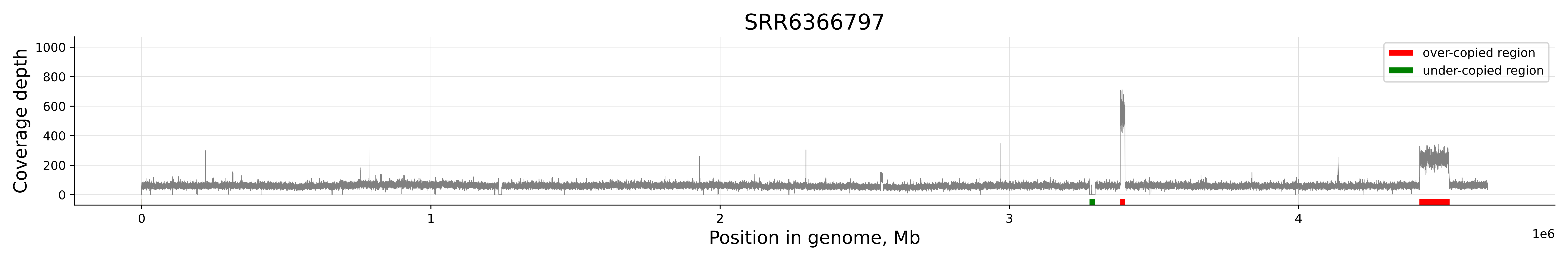

SRR6366797
Coverage depth
1000
800
600
400
200
0
0
1
2
3
4
1e6
Position in genome, Mb
over-copied region
under-copied region

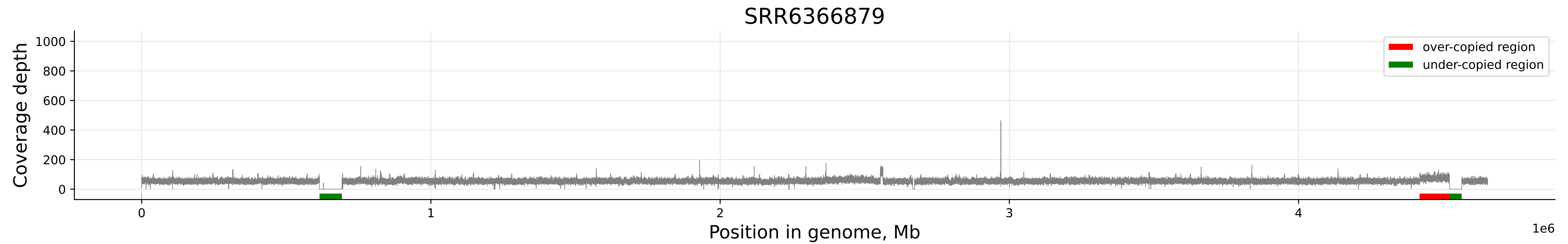
SRR6366879
Coverage depth
1000
800
600
400
200
0
0
1
2
3
4
1e6
Position in genome, Mb
over-copied region
under-copied region

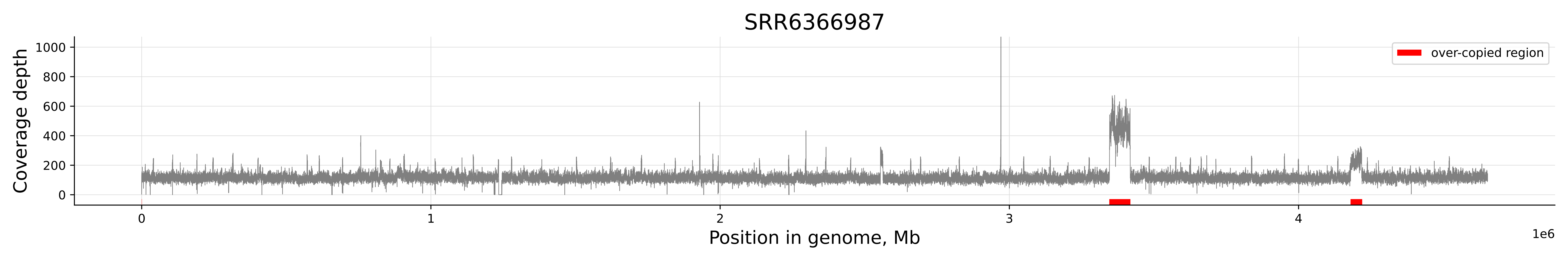

SRR6366987
Coverage depth
1000
800
600
400
200
0
over-copied region
0
1
2
3
4
1e6
Position in genome, Mb

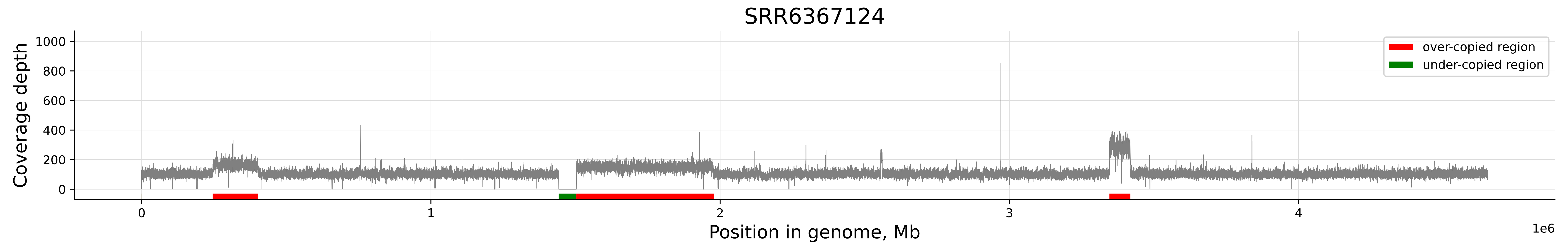
SRR6367124
Coverage depth
1000
800
600
400
200
0
0
1
2
3
4
1e6
Position in genome, Mb
over-copied region
under-copied region

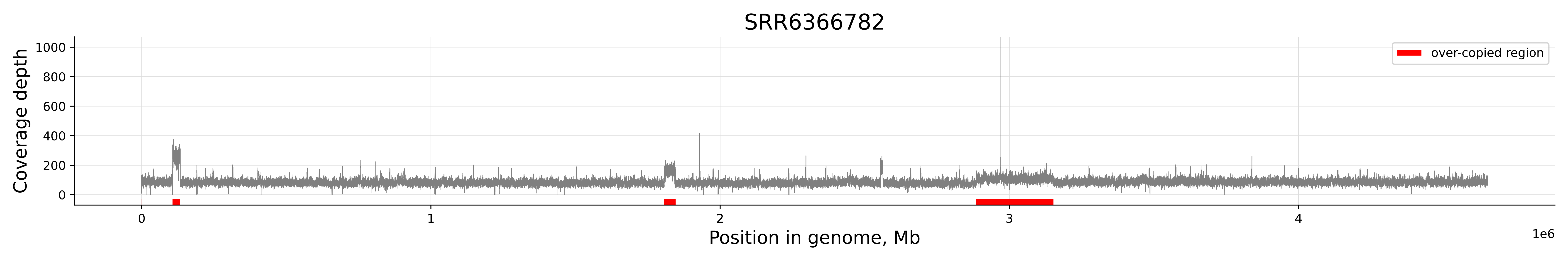

SRR6366782
Coverage depth
1000
800
600
400
200
0
0
1
2
3
4
1e6
Position in genome, Mb
over-copied region

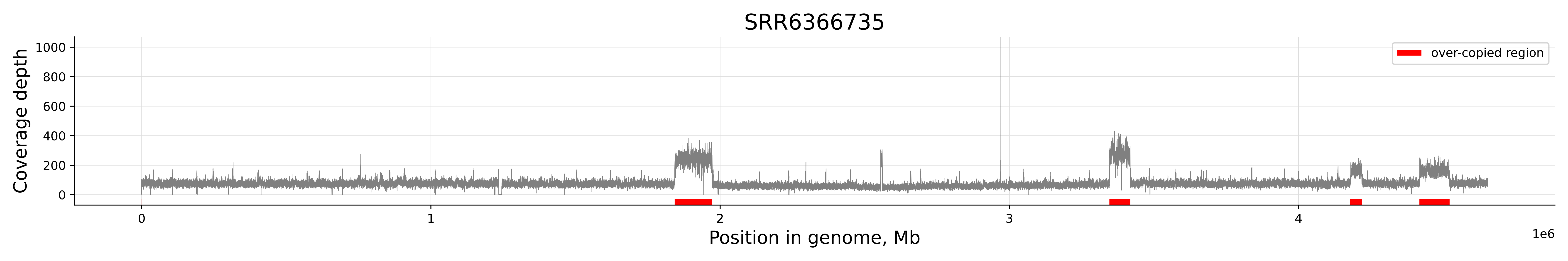

SRR6366735
Coverage depth
1000
800
600
400
200
0
over-copied region
0
1
2
3
4
1e6
Position in genome, Mb

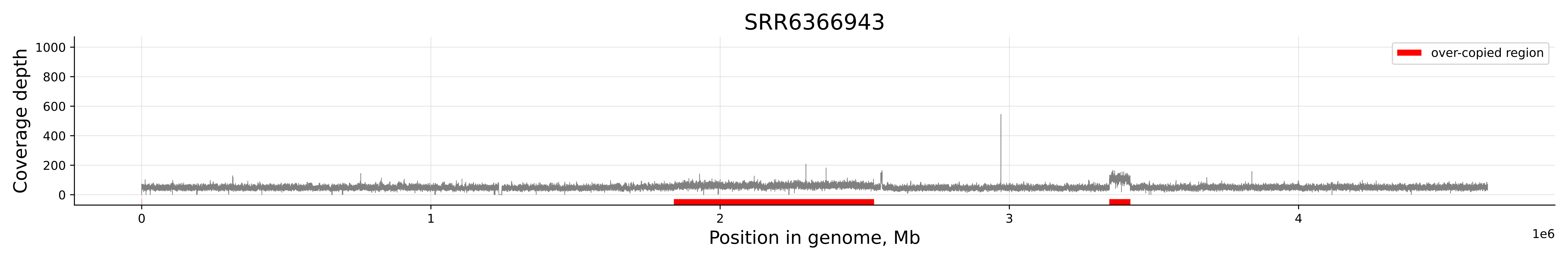
SRR6366943
Coverage depth
1000
800
600
400
200
0
over-copied region
0
1
2
3
4
Position in genome, Mb
1e6

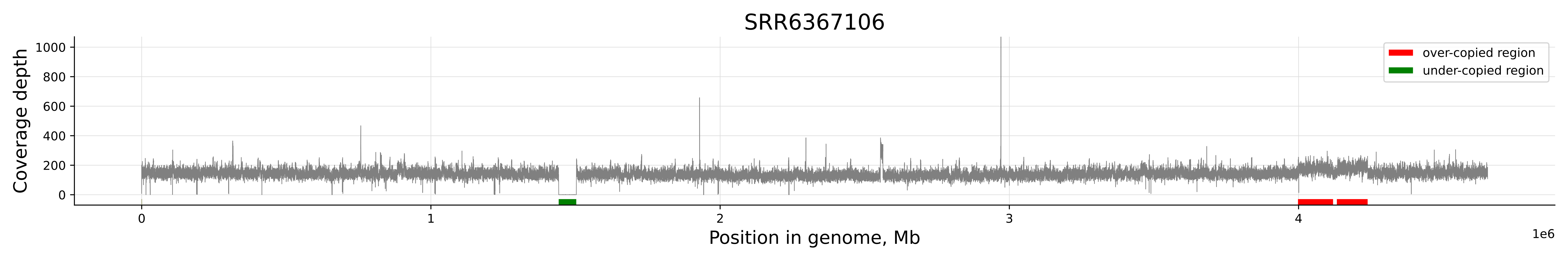

SRR6367106
Coverage depth
1000
800
600
400
200
0
over-copied region
under-copied region
0
1
2
3
4
1e6
Position in genome, Mb

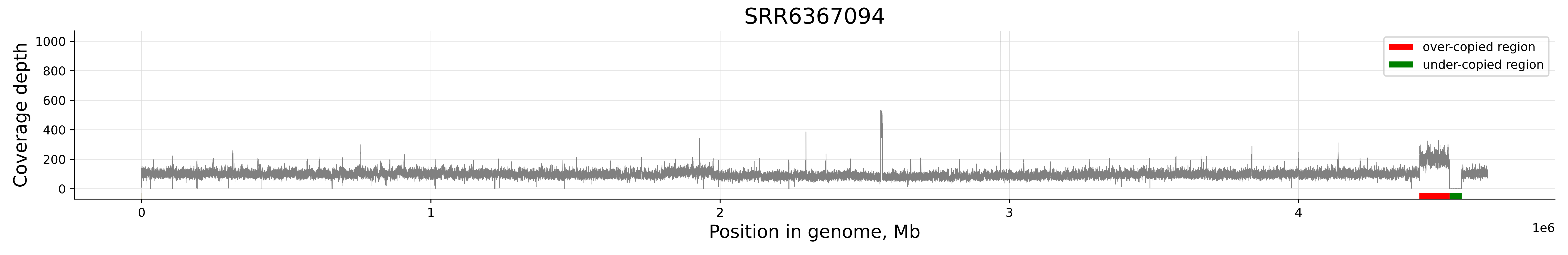
SRR6367094
Coverage depth
Position in genome, Mb
over-copied region
under-copied region
1e6

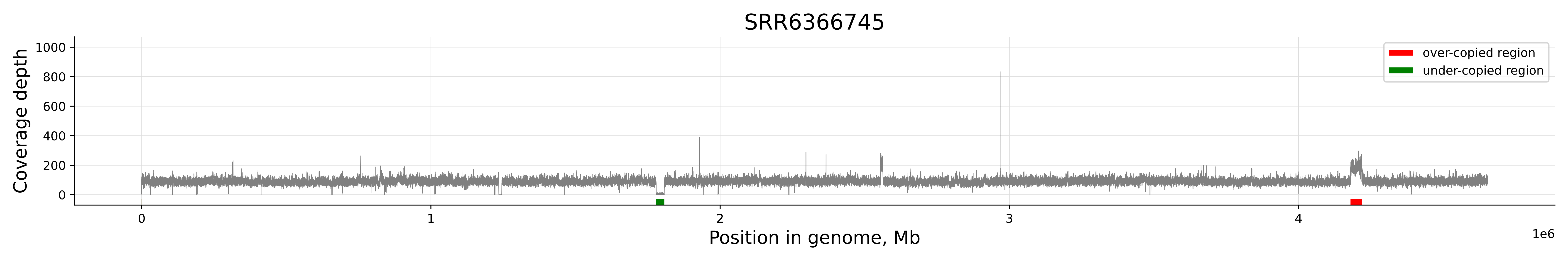

SRR6366745
Coverage depth
1000
800
600
400
200
0
0
1
2
3
4
1e6
Position in genome, Mb
over-copied region
under-copied region

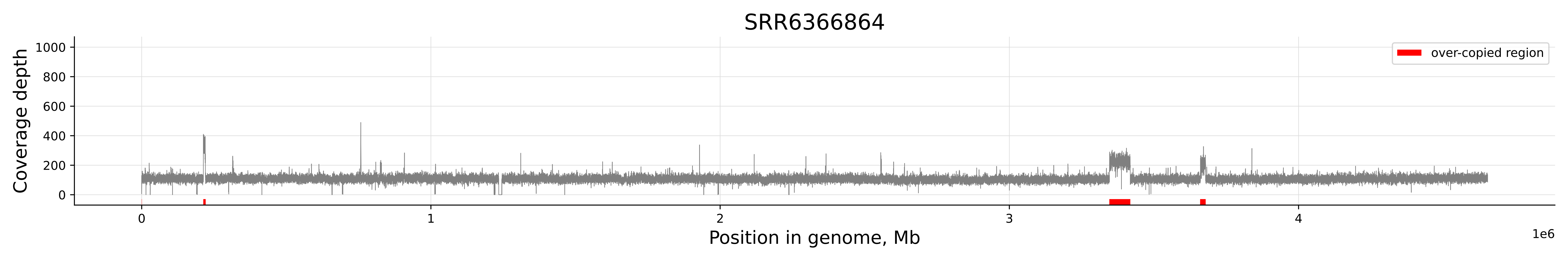
SRR6366864
Coverage depth
1000
800
600
400
200
0
over-copied region
0
1
2
3
4
1e6
Position in genome, Mb

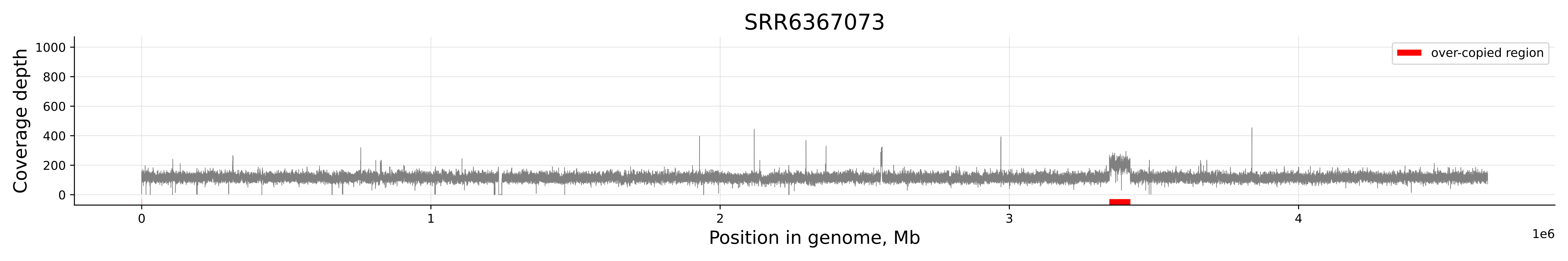
SRR6367073
Coverage depth
1000
800
600
400
200
0
0
1
2
3
4
1e6
Position in genome, Mb
over-copied region

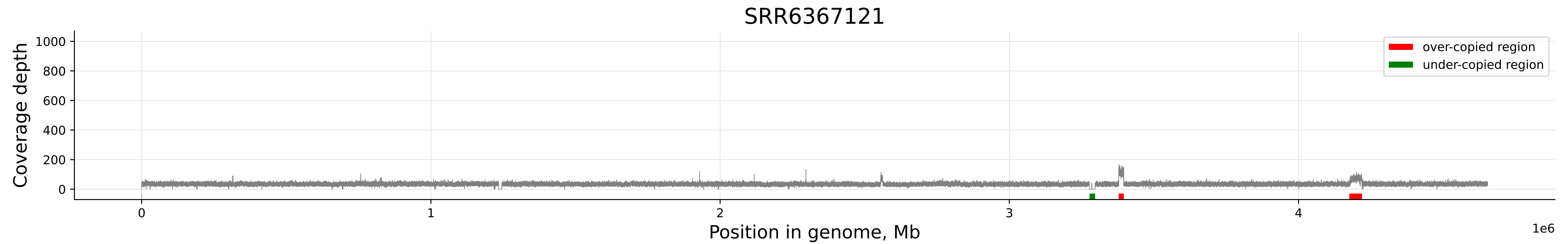
SRR6367121
Coverage depth
1000
800
600
400
200
0
0
1
2
3
4
1e6
Position in genome, Mb
over-copied region
under-copied region

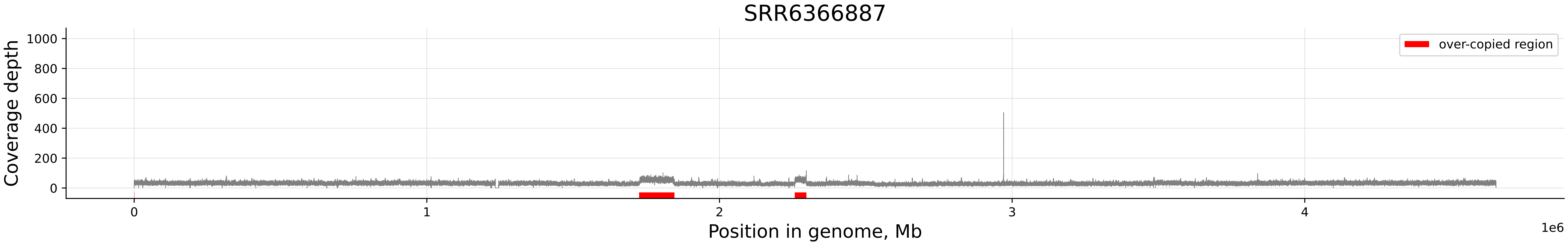
SRR6366887
Coverage depth
1000
800
600
400
200
0
over-copied region
0
1
2
3
4
Position in genome, Mb
1e6

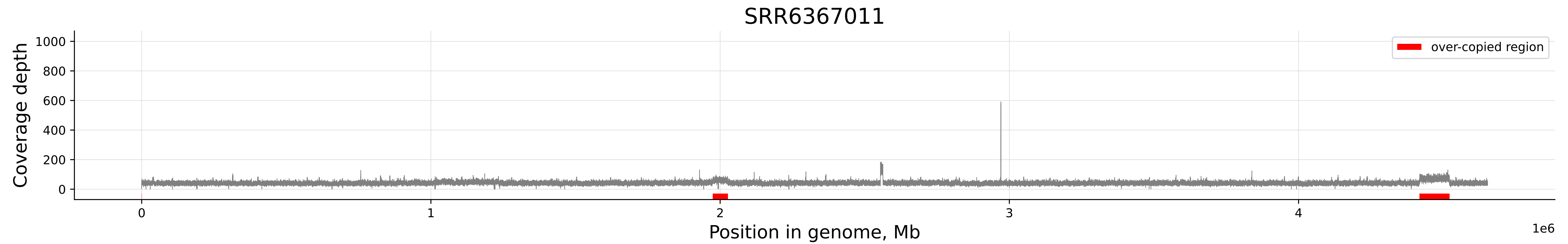

SRR6367011
Coverage depth
1000
800
600
400
200
0
over-copied region
0
1
2
3
4
1e6
Position in genome, Mb

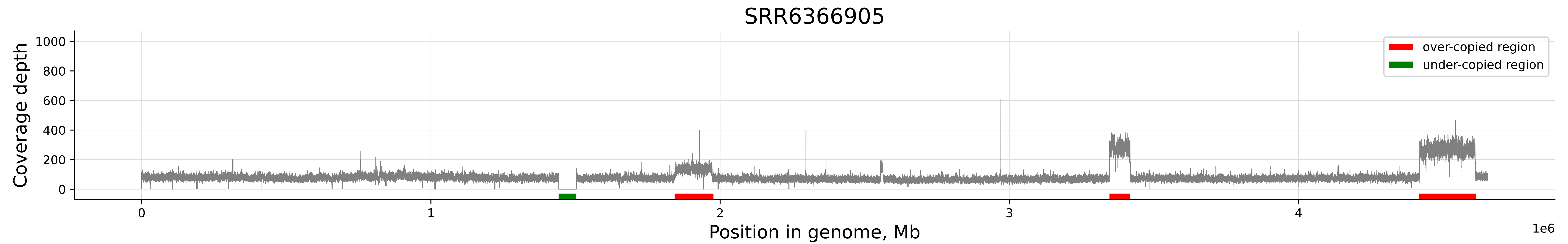
SRR6366905
Coverage depth
1000
800
600
400
200
0
0
1
2
3
4
1e6
Position in genome, Mb
over-copied region
under-copied region

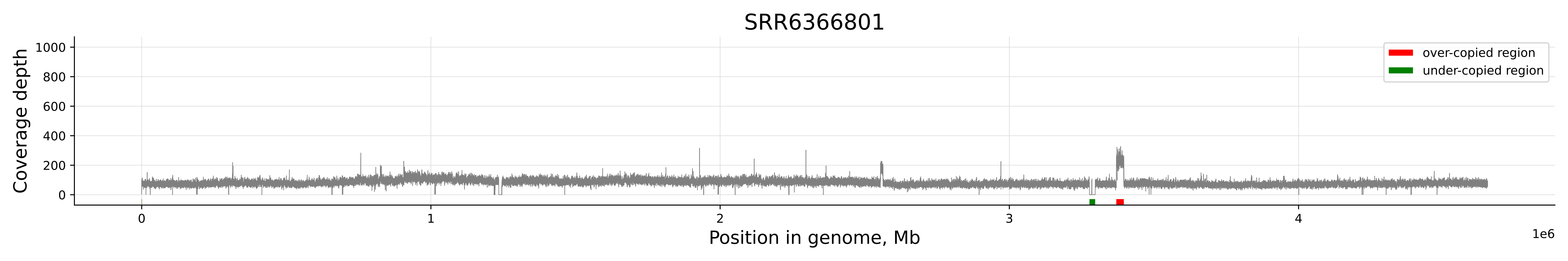
SRR6366801
Coverage depth
Position in genome, Mb
over-copied region
under-copied region
1e6
0
1
2
3
4
0
200
400
600
800
1000

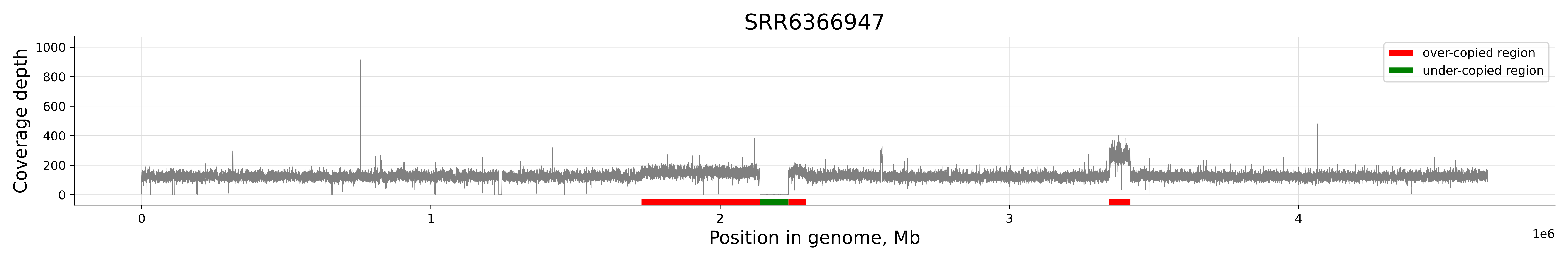
SRR6366947
Coverage depth
1000
800
600
400
200
0
over-copied region
under-copied region
0
1
2
3
4
1e6
Position in genome, Mb

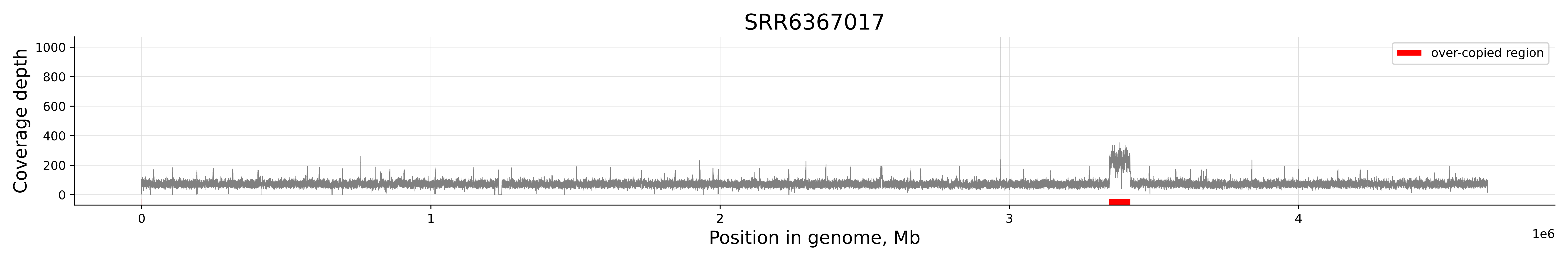
SRR6367017
over-copied region
Coverage depth
1000
800
600
400
200
0
0
1
2
3
4
1e6
Position in genome, Mb

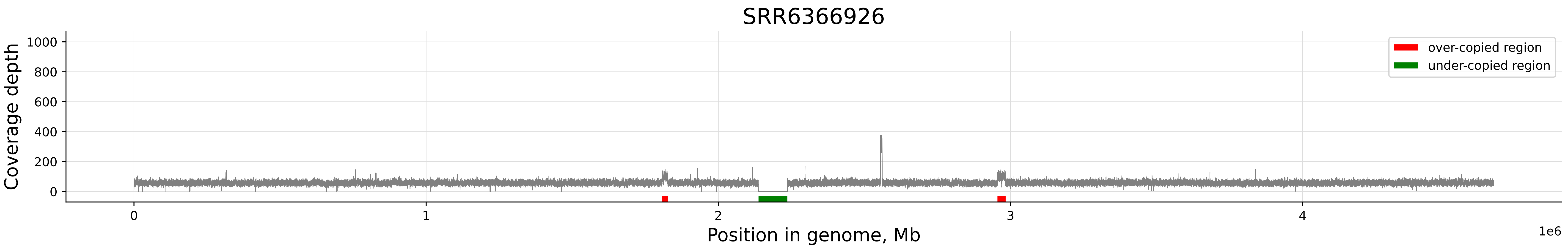

SRR6366926
over-copied region
under-copied region
Coverage depth
0
200
400
600
800
1000
0
1
2
3
4
1e6
Position in genome, Mb

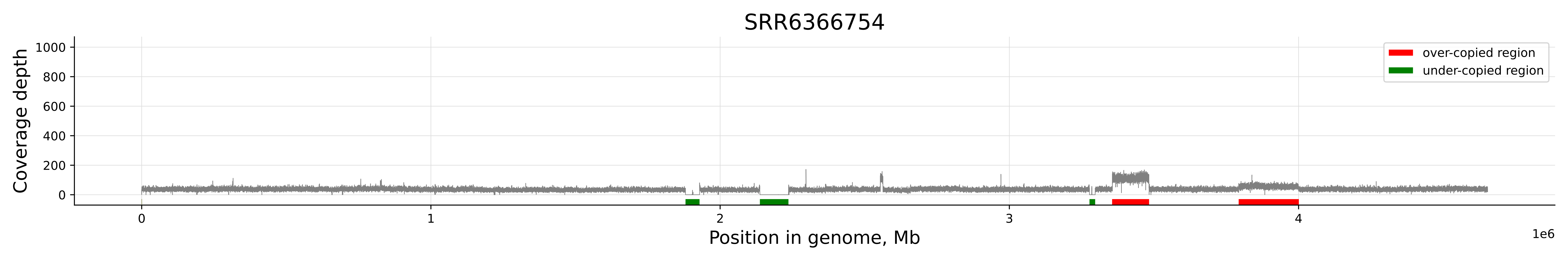

SRR6366754
Coverage depth
Position in genome, Mb
1e6
over-copied region
under-copied region
0
200
400
600
800
1000
0
1
2
3
4

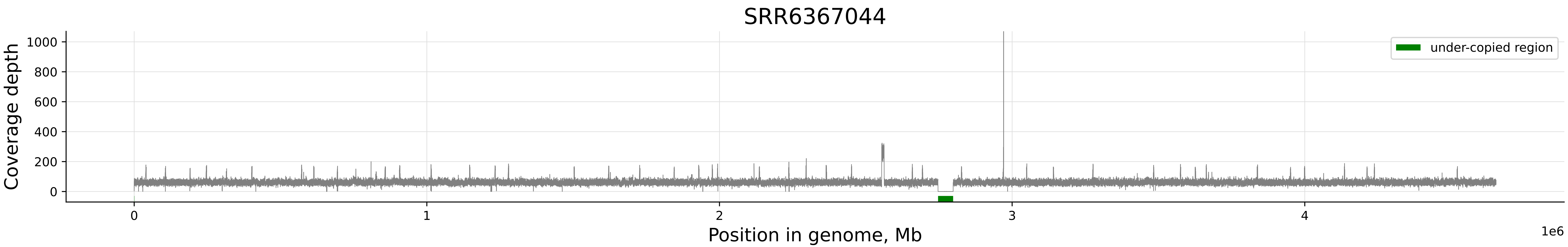
SRR6367044
Coverage depth
1000
800
600
400
200
0
under-copied region
0
1
2
3
4
1e6
Position in genome, Mb

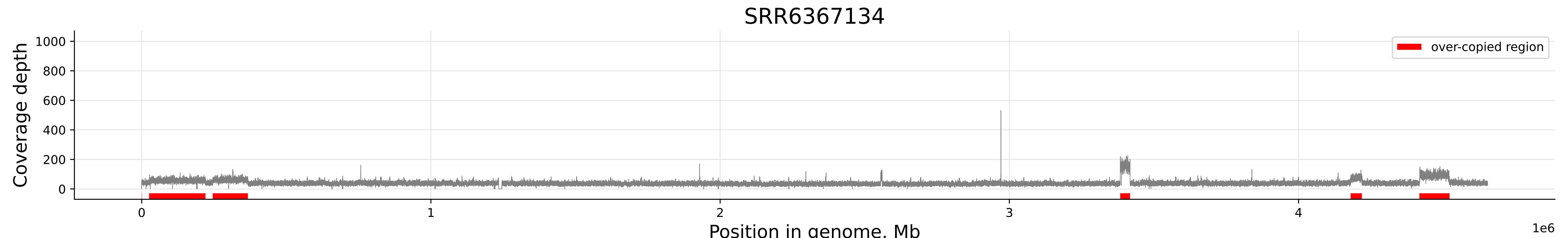
SRR6367134
over-copied region
Coverage depth
1000
800
600
400
200
0
1
2
3
4
Position in genome, Mb
1e6

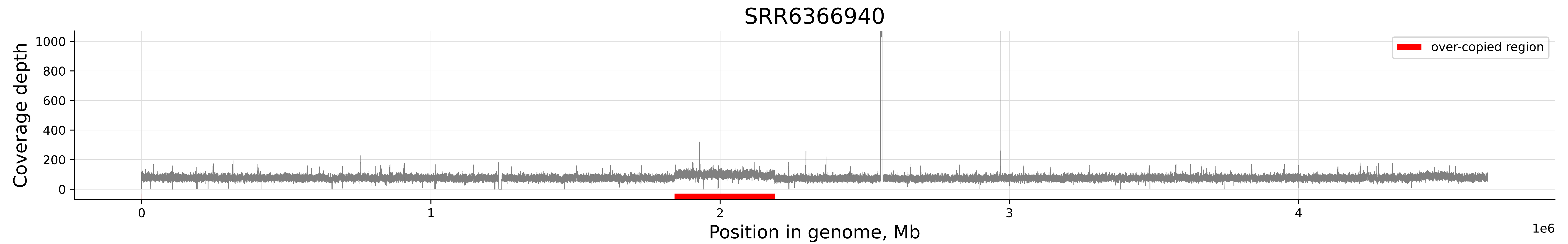

SRR6366940
over-copied region
Coverage depth
0
200
400
600
800
1000
Position in genome, Mb
0
1
2
3
4
1e6

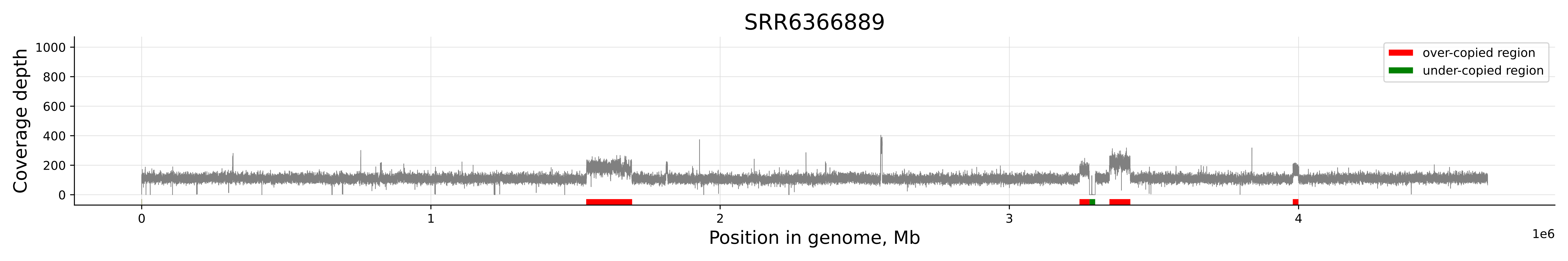
SRR6366889
Coverage depth
1000
800
600
400
200
0
0
1
2
3
4
1e6
Position in genome, Mb
over-copied region
under-copied region

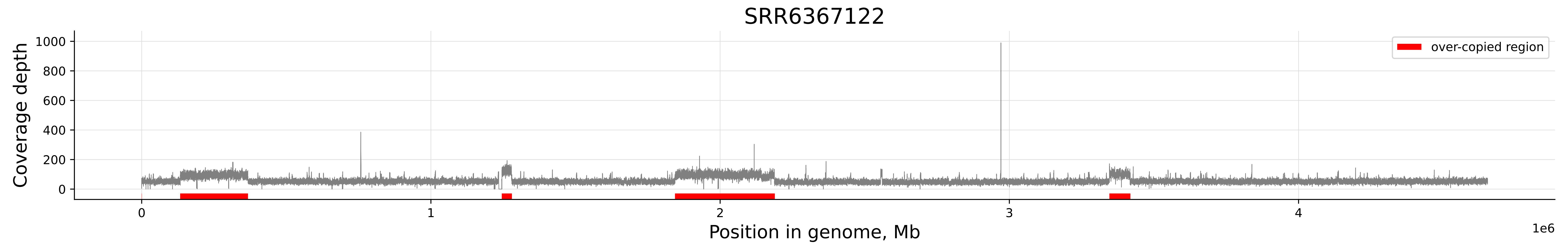

SRR6367122
Coverage depth
1000
800
600
400
200
0
over-copied region
0
1
2
3
4
1e6
Position in genome, Mb

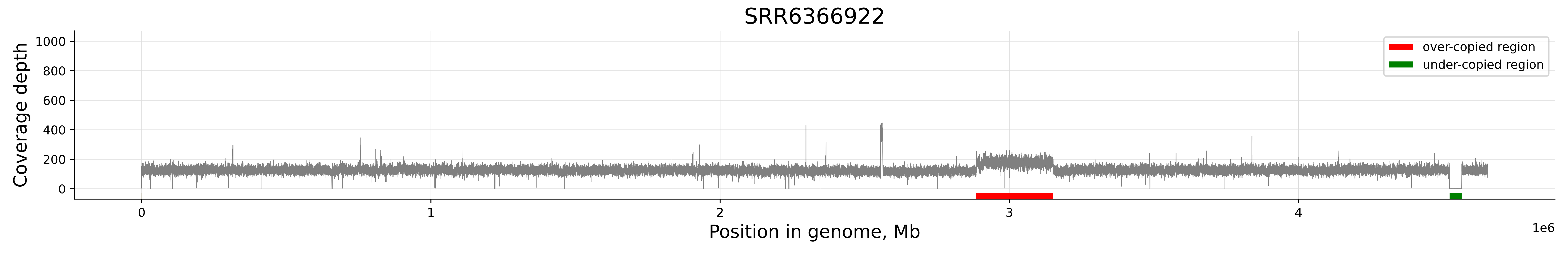
SRR6366922
Coverage depth
Position in genome, Mb
1e6
over-copied region
under-copied region
0
200
400
600
800
1000
0
1
2
3
4

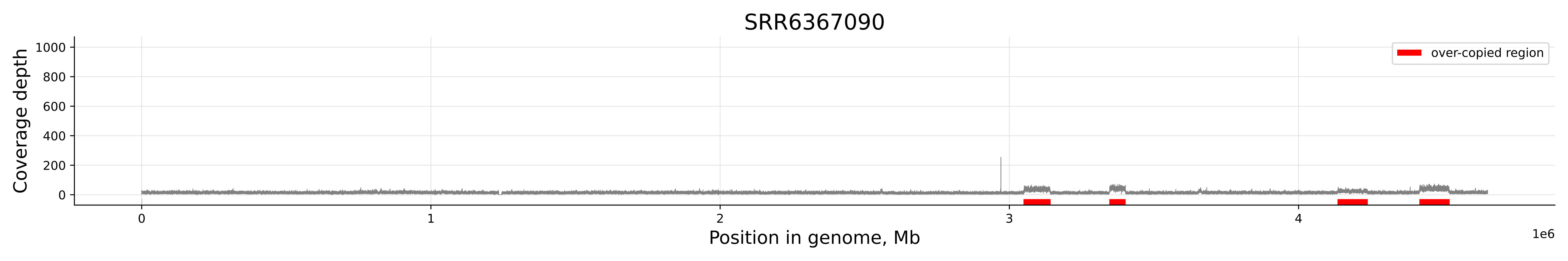
SRR6367090
Coverage depth
1000
800
600
400
200
0
over-copied region
0
1
2
3
4
1e6
Position in genome, Mb

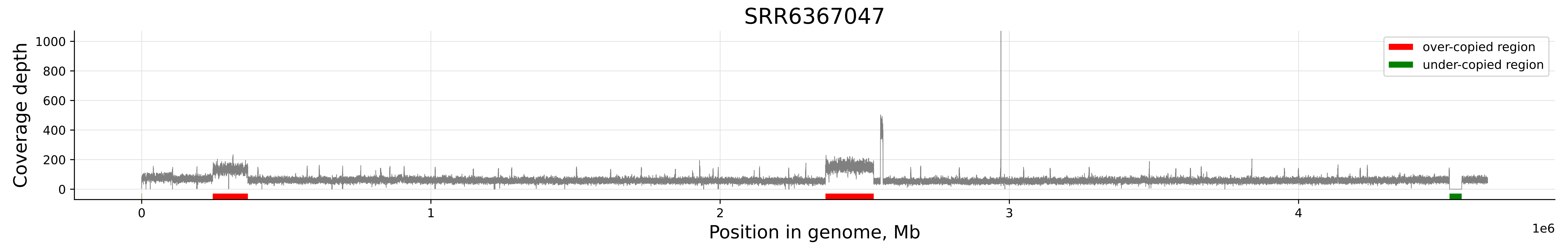

SRR6367047
Coverage depth
Position in genome, Mb
1e6
over-copied region
under-copied region
0
200
400
600
800
1000
1
2
3
4

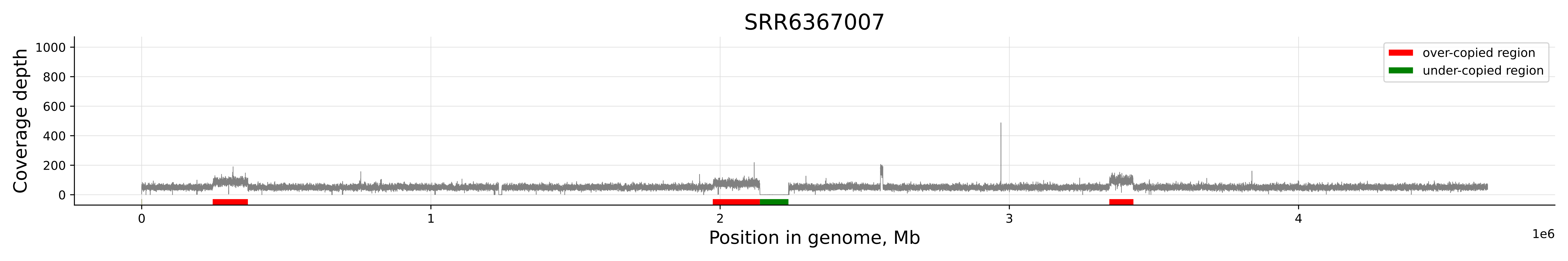

SRR6367007
Coverage depth
Position in genome, Mb
1e6
over-copied region
under-copied region
0
200
400
600
800
1000
1
2
3
4

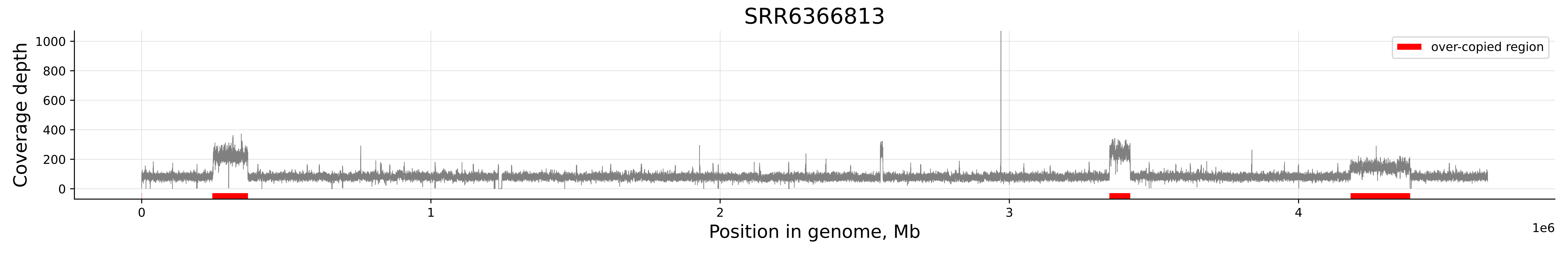

SRR6366813
over-copied region
Coverage depth
0
200
400
600
800
1000
0
1
2
3
4
1e6
Position in genome, Mb

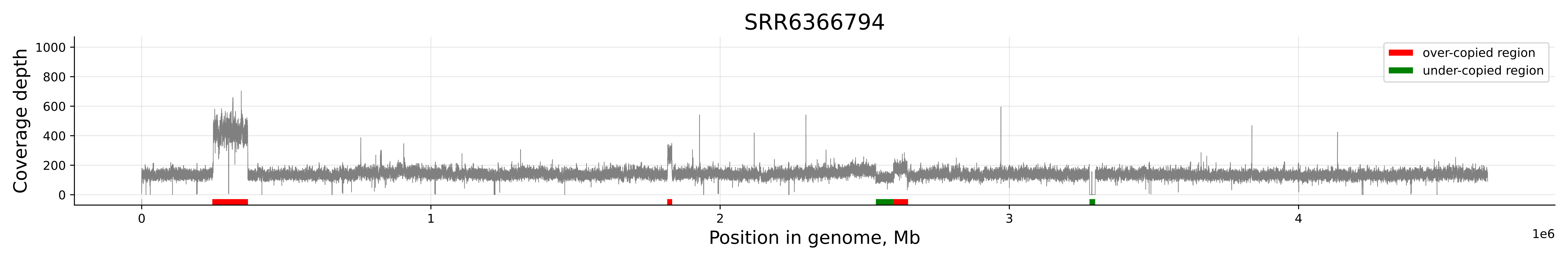
SRR6366794
Coverage depth
1000
800
600
400
200
0
0
1
2
3
4
1e6
Position in genome, Mb
over-copied region
under-copied region

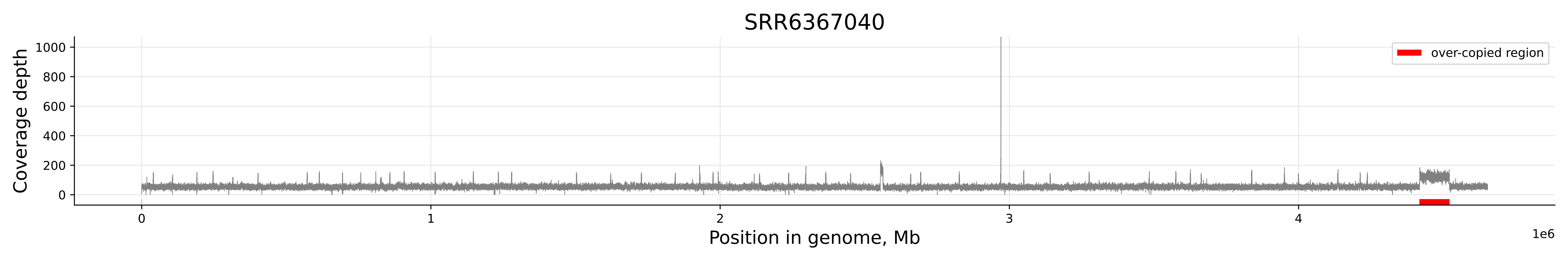
SRR6367040
Coverage depth
1000
800
600
400
200
0
0
1
2
3
4
Position in genome, Mb
1e6
over-copied region

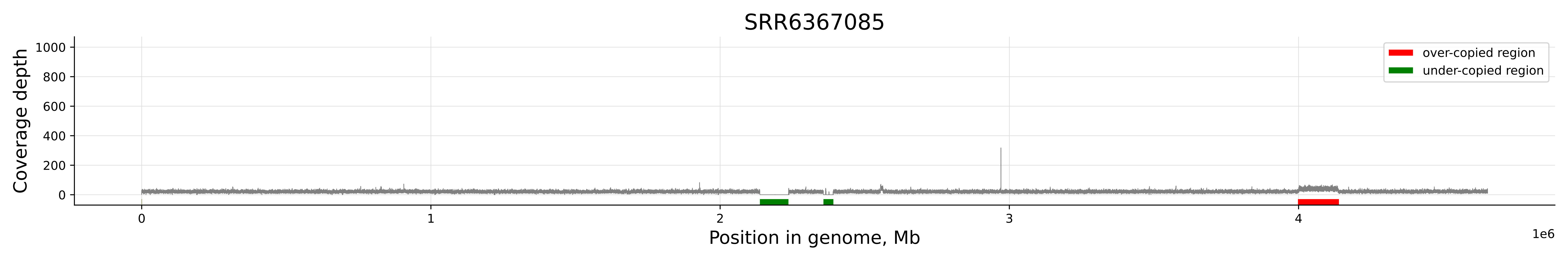
SRR6367085
Coverage depth
1000
800
600
400
200
0
0
1
2
3
4
1e6
Position in genome, Mb
over-copied region
under-copied region

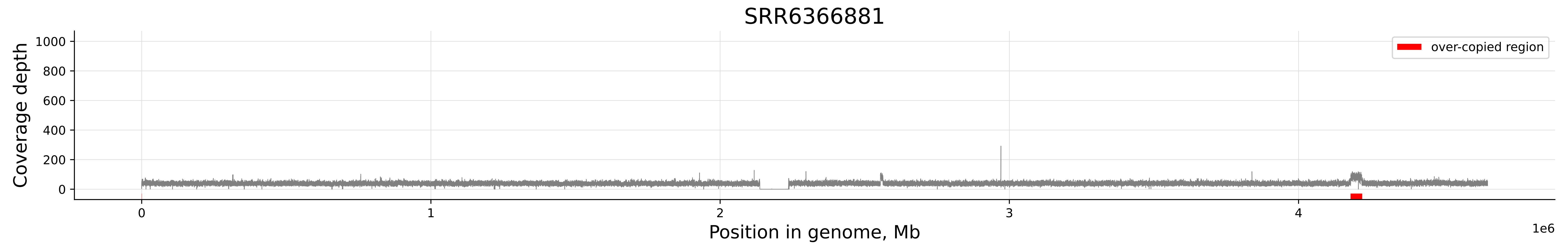

SRR6366881
Coverage depth
1000
800
600
400
200
0
over-copied region
0
1
2
3
4
1e6
Position in genome, Mb

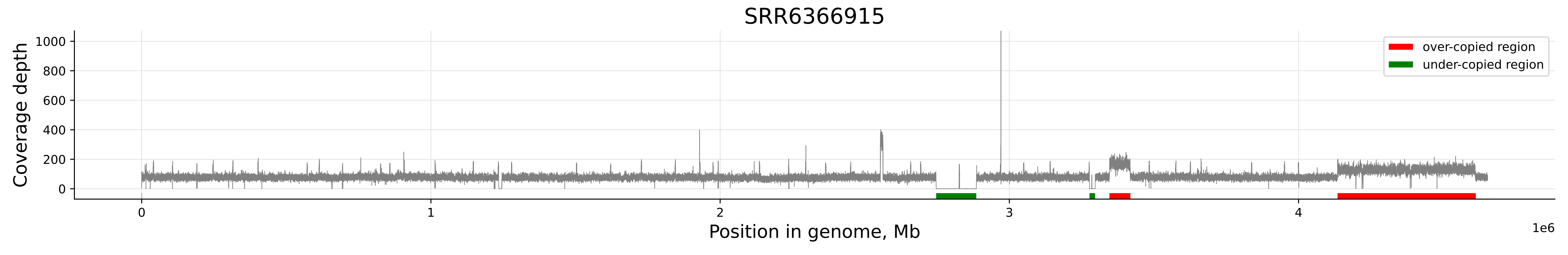
SRR6366915
Coverage depth
1000
800
600
400
200
0
over-copied region
under-copied region
0
1
2
3
4
Position in genome, Mb
1e6

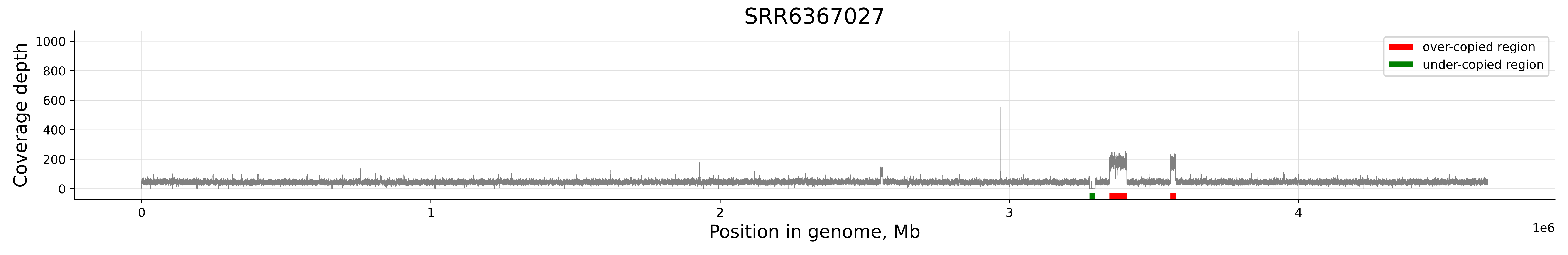
SRR6367027
Coverage depth
Position in genome, Mb
1e6
over-copied region
under-copied region
0
200
400
600
800
1000
1
2
3
4

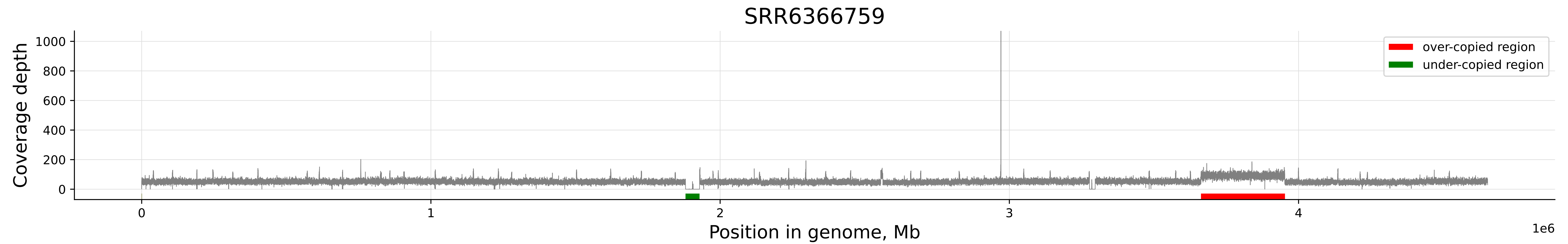
SRR6366759
Coverage depth
1000
800
600
400
200
0
0
1
2
3
4
1e6
Position in genome, Mb
over-copied region
under-copied region

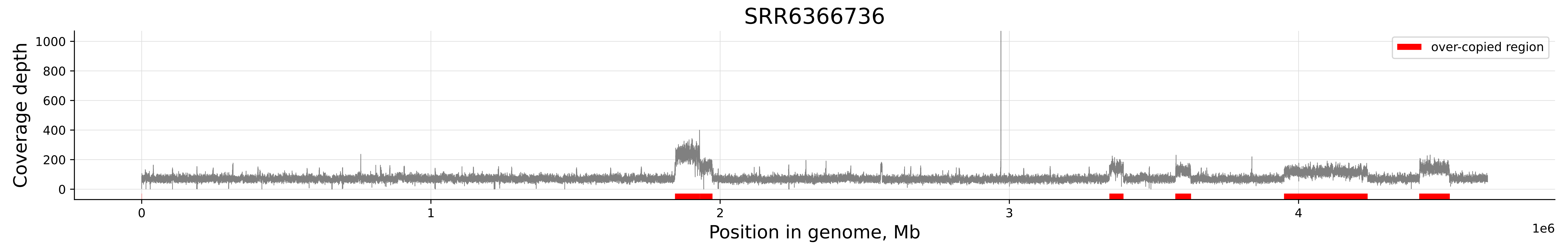

SRR6366736
Coverage depth
1000
800
600
400
200
0
over-copied region
0
1
2
3
4
Position in genome, Mb
1e6

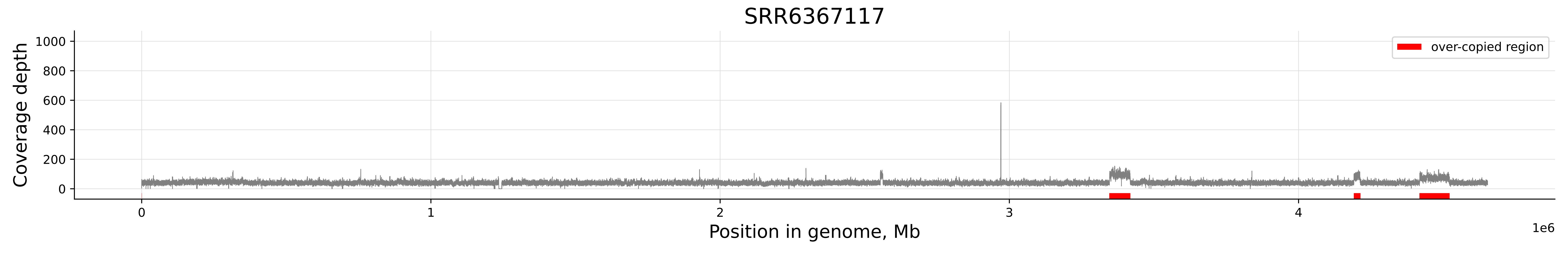
SRR6367117
Coverage depth
1000
800
600
400
200
0
over-copied region
0
1
2
3
4
Position in genome, Mb
1e6

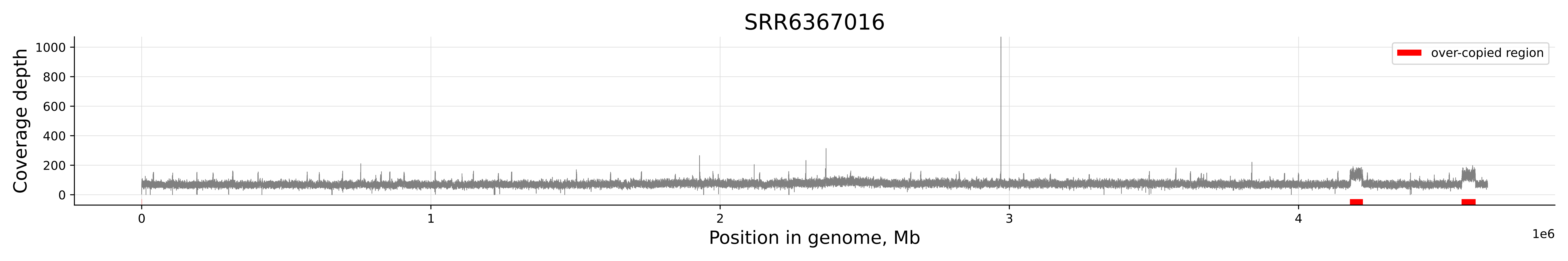

SRR6367016
Coverage depth
1000
800
600
400
200
0
over-copied region
0
1
2
3
4
1e6
Position in genome, Mb

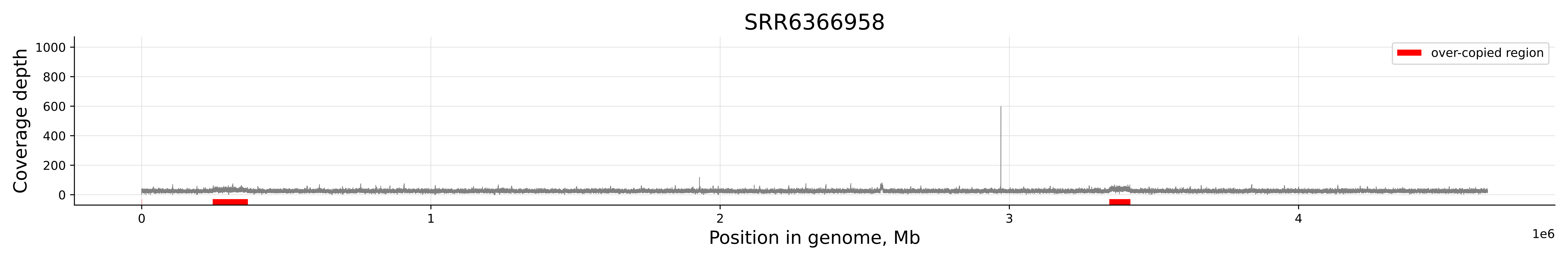

SRR6366958
Coverage depth
1000
800
600
400
200
0
over-copied region
0
1
2
3
4
1e6
Position in genome, Mb

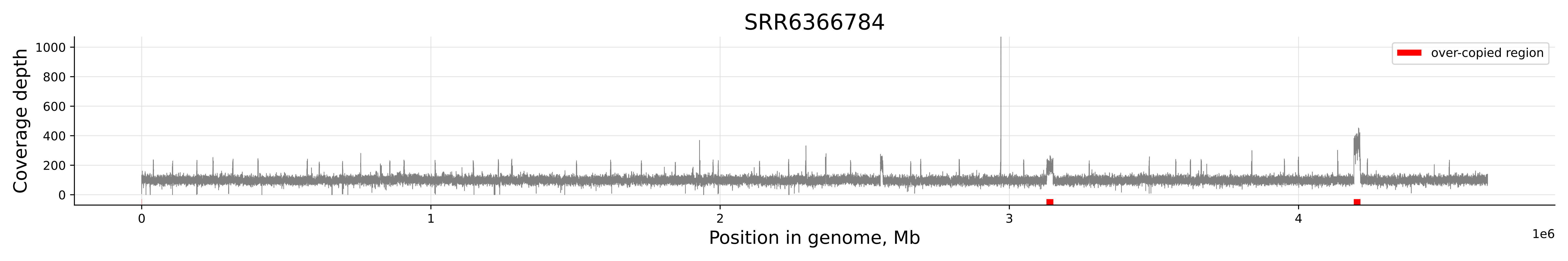
SRR6366784
Coverage depth
1000
800
600
400
200
0
over-copied region
0
1
2
3
4
1e6
Position in genome, Mb

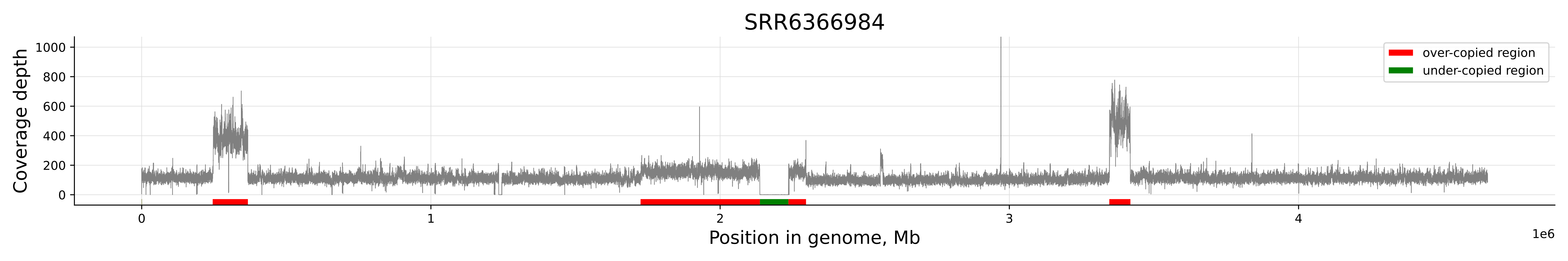

SRR6366984
over-copied region
under-copied region
Coverage depth
0
200
400
600
800
1000
0
1
2
3
4
1e6
Position in genome, Mb

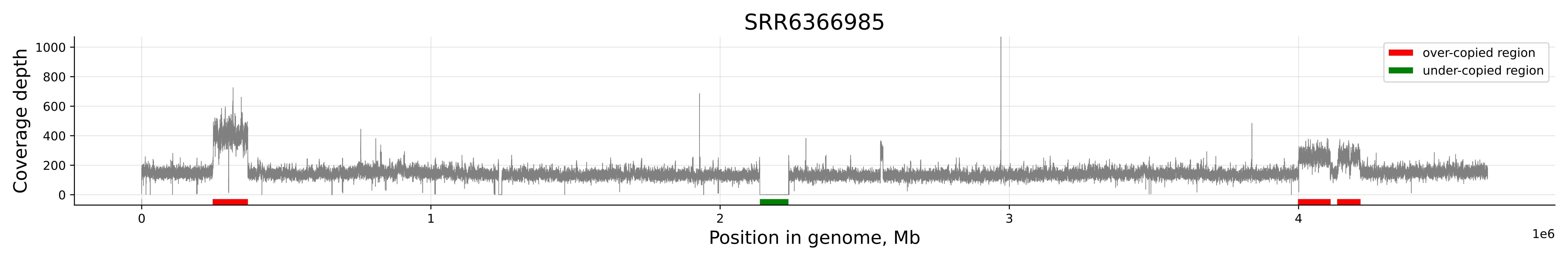

SRR6366985
Coverage depth
Position in genome, Mb
1e6
over-copied region
under-copied region
0
200
400
600
800
1000
0
1
2
3
4

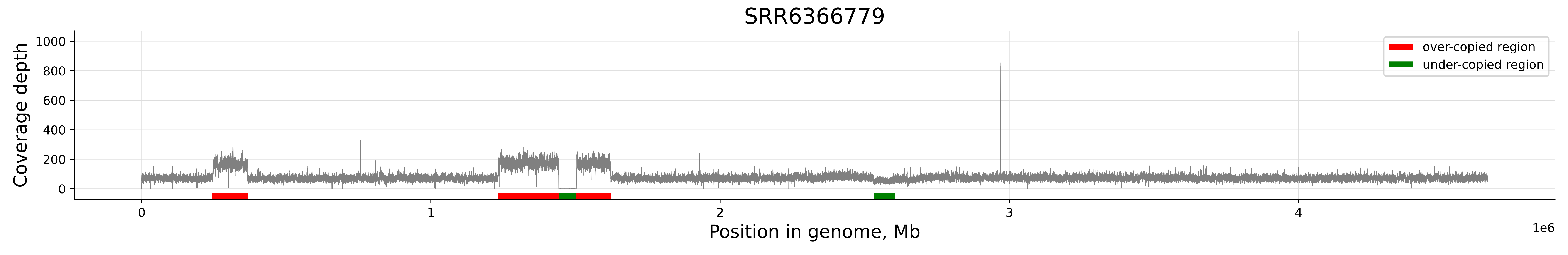
SRR6366779
over-copied region
under-copied region
Coverage depth
0
200
400
600
800
1000
0
1
2
3
4
1e6
Position in genome, Mb

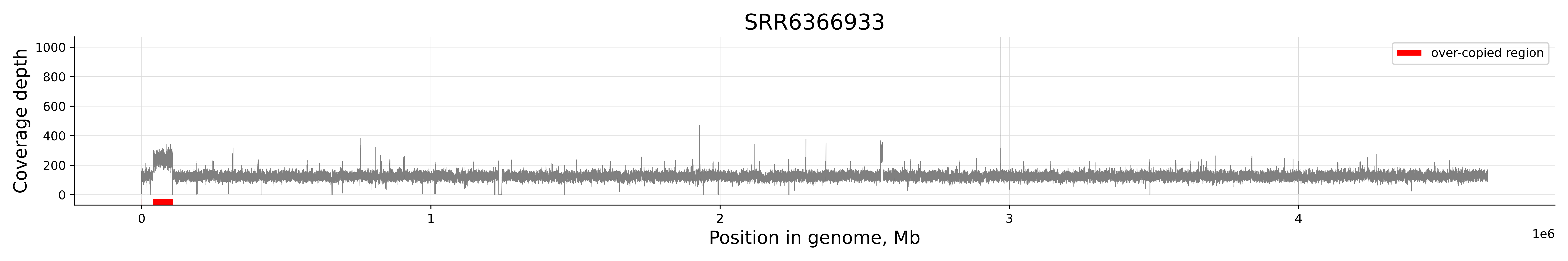

SRR6366933
Coverage depth
1000
800
600
400
200
0
over-copied region
0
1
2
3
4
1e6
Position in genome, Mb

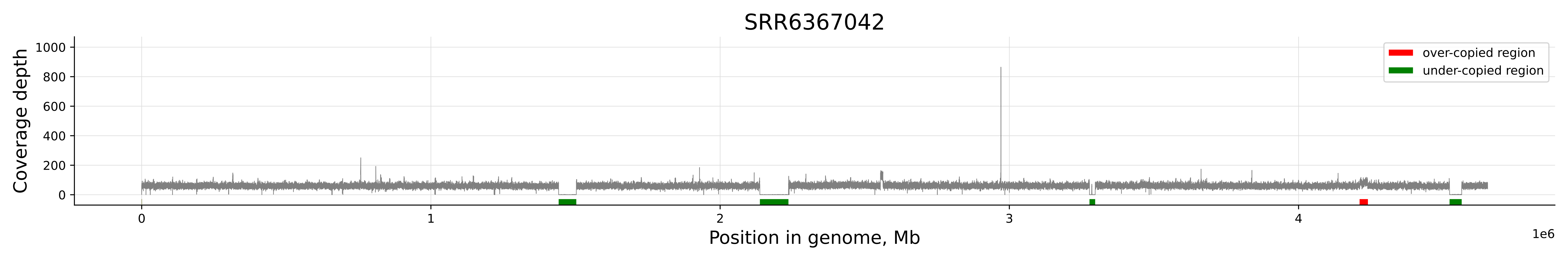

SRR6367042
Coverage depth
0
200
400
600
800
1000
0
1
2
3
4
1e6
Position in genome, Mb
over-copied region
under-copied region

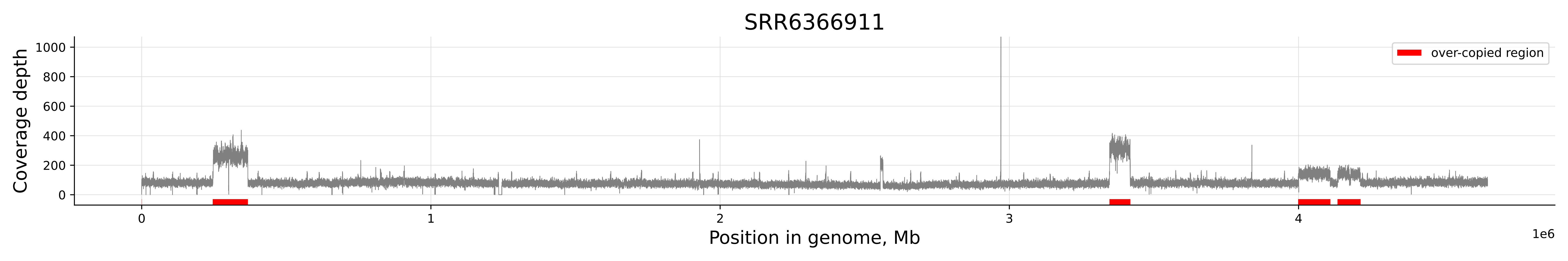
SRR6366911
Coverage depth
Position in genome, Mb
over-copied region
1e6
0
1
2
3
4
0
200
400
600
800
1000

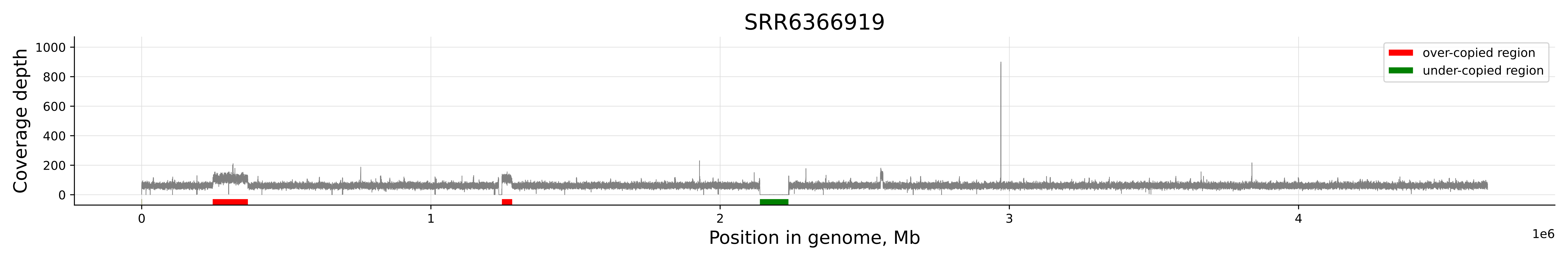

SRR6366919
Coverage depth
1000
800
600
400
200
0
over-copied region
under-copied region
0
1
2
3
4
1e6
Position in genome, Mb

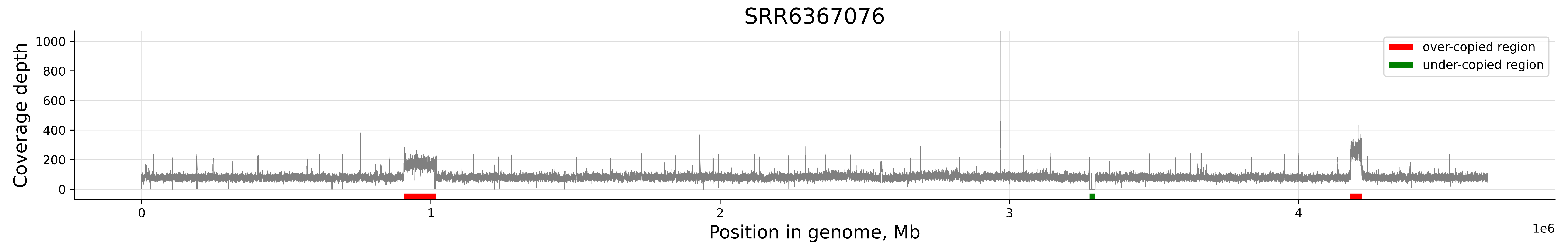

SRR6367076
Coverage depth
1000
800
600
400
200
0
Position in genome, Mb
0
1
2
3
4
1e6
over-copied region
under-copied region

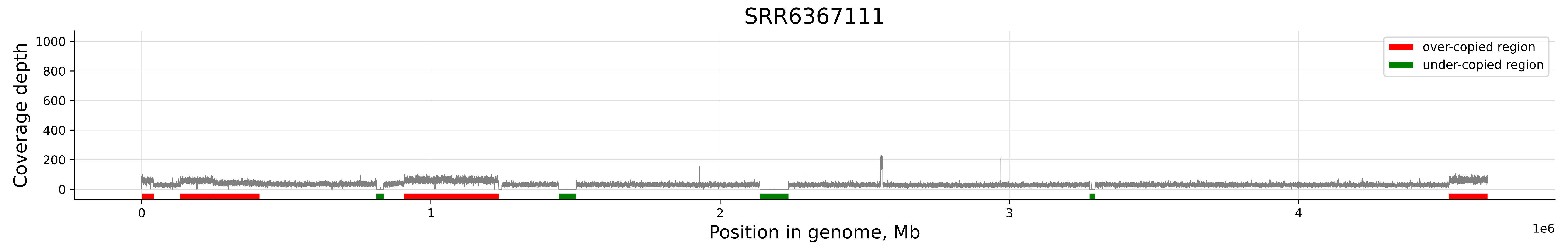

SRR6367111
Coverage depth
1000
800
600
400
200
0
0
1
2
3
4
1e6
Position in genome, Mb
over-copied region
under-copied region

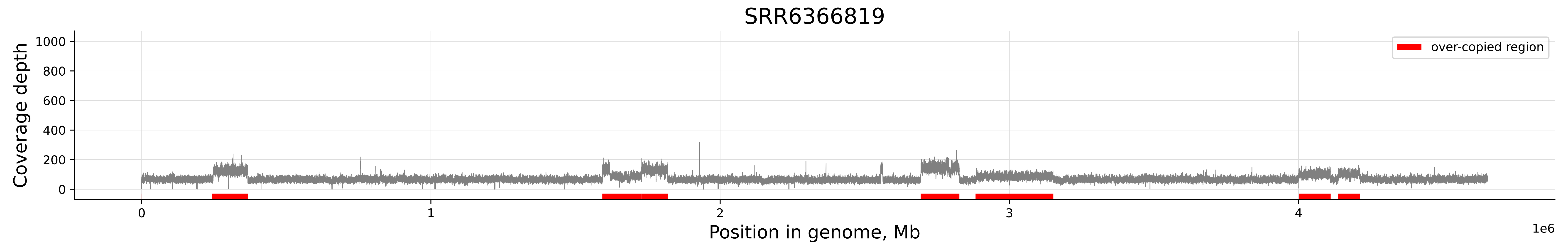

SRR6366819
Coverage depth
1000
800
600
400
200
0
over-copied region
0
1
2
3
4
1e6
Position in genome, Mb

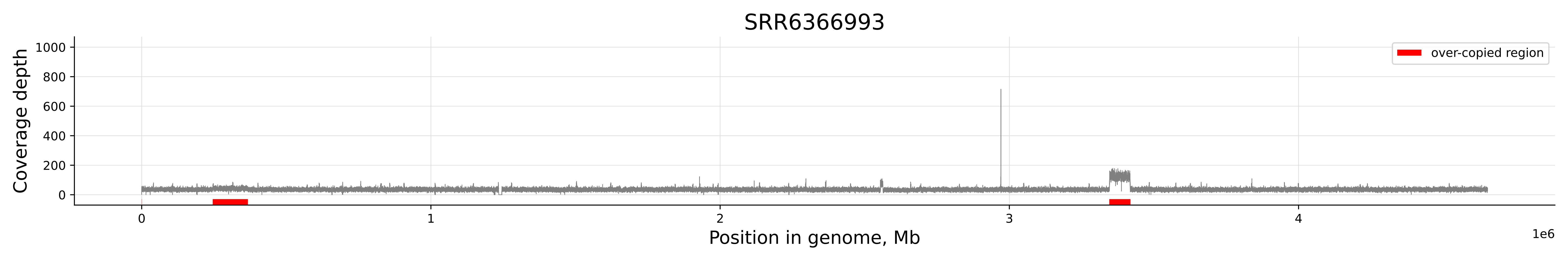

SRR6366993
Coverage depth
Position in genome, Mb
over-copied region
1e6
0
1
2
3
4
0
200
400
600
800
1000

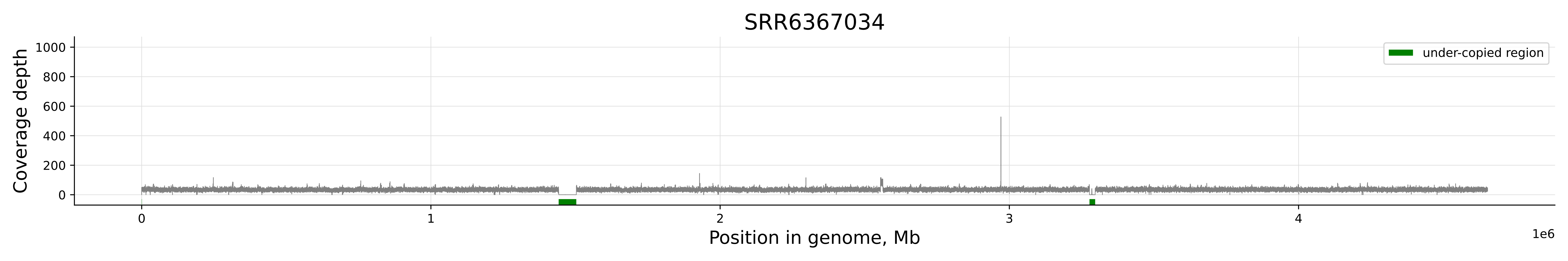
SRR6367034
Coverage depth
1000
800
600
400
200
0
0
1
2
3
4
1e6
Position in genome, Mb
under-copied region

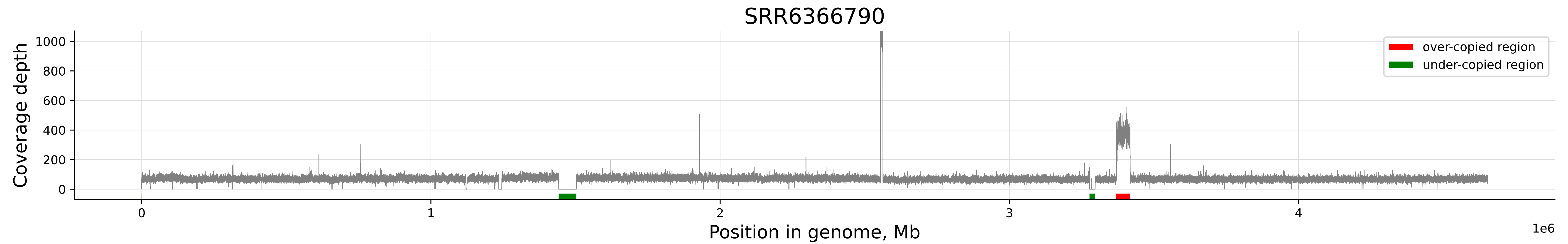
SRR6366790
Coverage depth
Position in genome, Mb
over-copied region
under-copied region
1e6

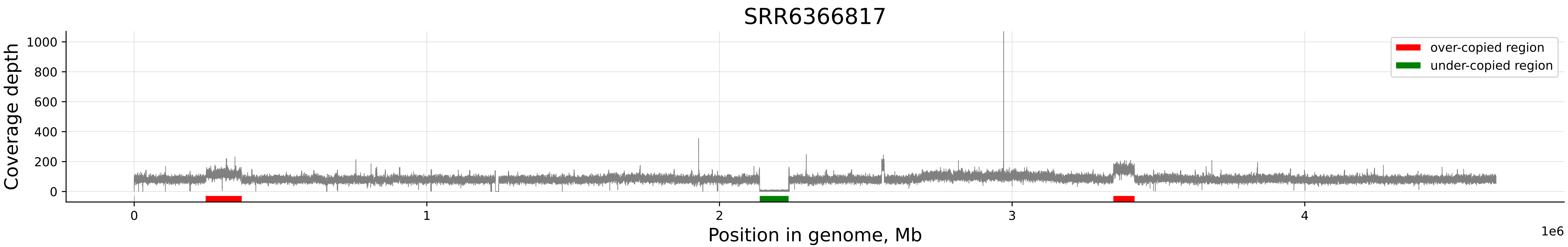
SRR6366817
Coverage depth
1000
800
600
400
200
0
over-copied region
under-copied region
0
1
2
3
4
1e6
Position in genome, Mb

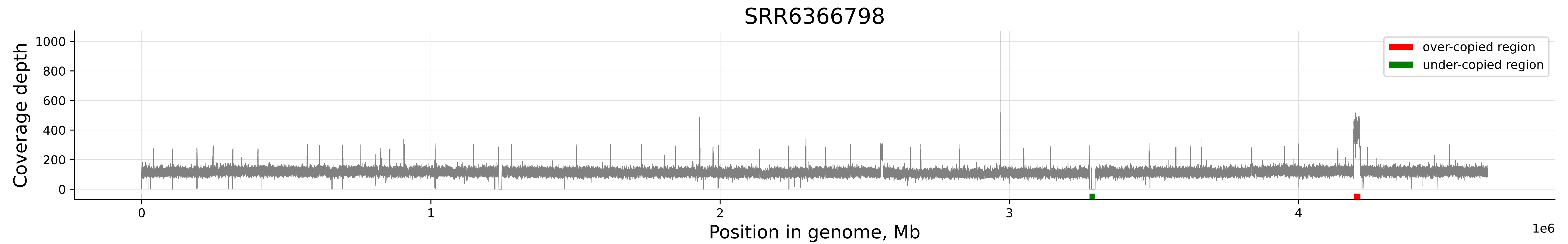
SRR6366798
Coverage depth
Position in genome, Mb
1e6
over-copied region
under-copied region
1000
800
600
400
200
0
0
1
2
3
4

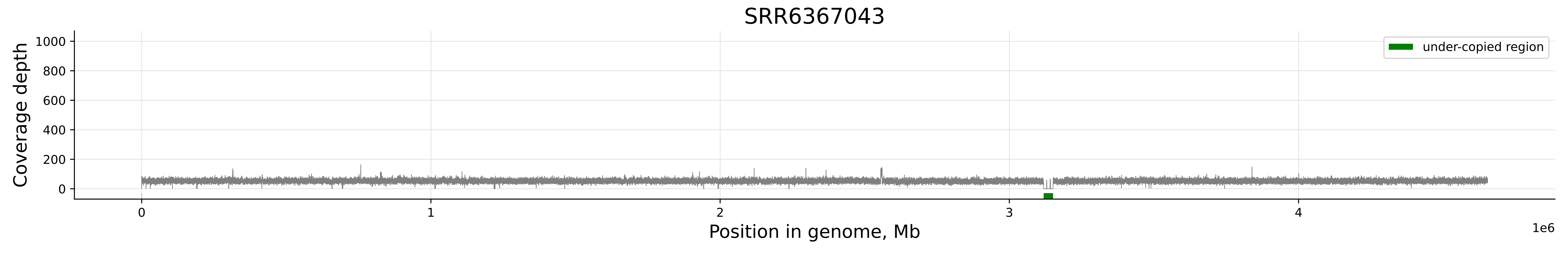

SRR6367043
Coverage depth
1000
800
600
400
200
0
under-copied region
0
1
2
3
4
1e6
Position in genome, Mb

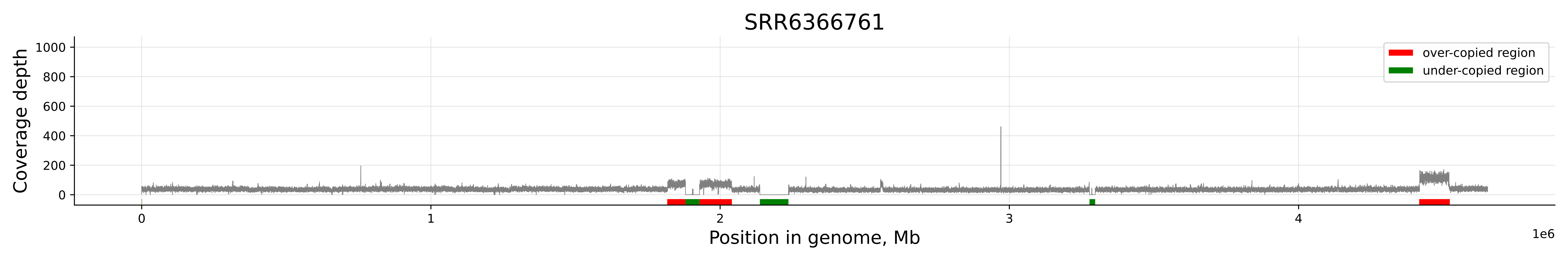

SRR6366761
over-copied region
under-copied region
Coverage depth
1000
800
600
400
200
0
0
1
2
3
4
1e6
Position in genome, Mb

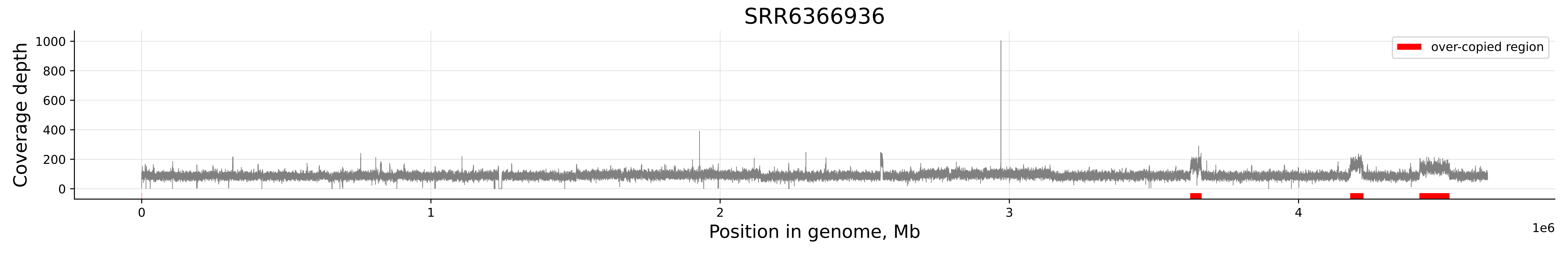
SRR6366936
Coverage depth
1000
800
600
400
200
0
0
1
2
3
4
1e6
Position in genome, Mb
over-copied region

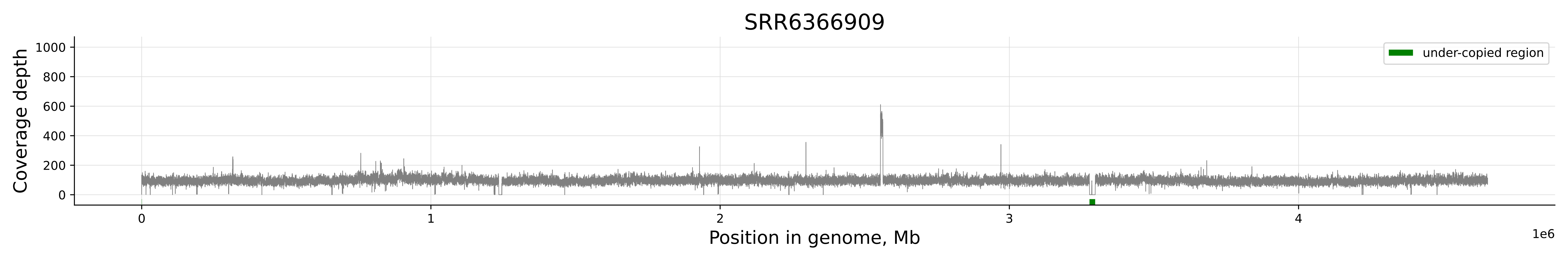
SRR6366909
Coverage depth
Position in genome, Mb
under-copied region
1000
800
600
400
200
0
0
1
2
3
4
1e6

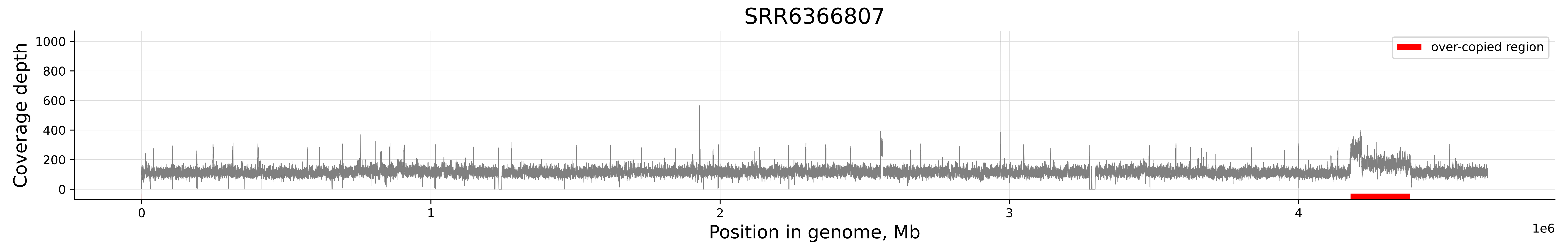
SRR6366807
over-copied region
Coverage depth
1000
800
600
400
200
0
0
1
2
3
4
1e6
Position in genome, Mb

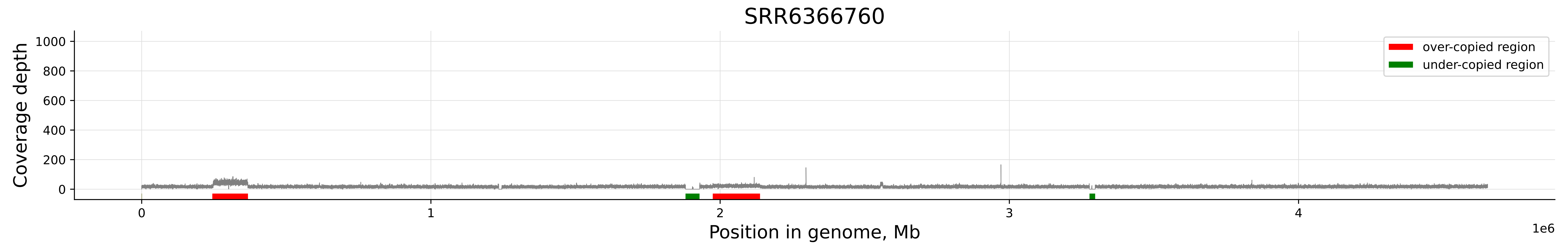
SRR6366760
over-copied region
under-copied region
Coverage depth
Position in genome, Mb
1e6
0
200
400
600
800
1000
0
1
2
3
4

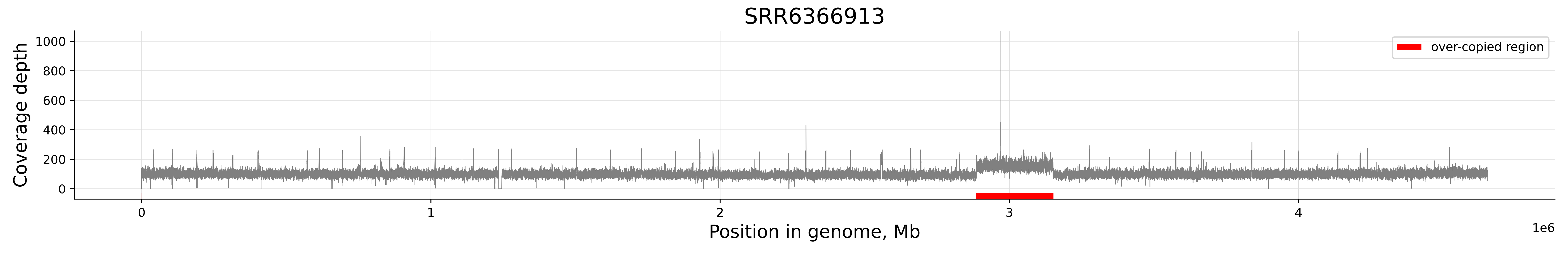
SRR6366913
Coverage depth
Position in genome, Mb
1e6
over-copied region
0
200
400
600
800
1000
1
2
3
4

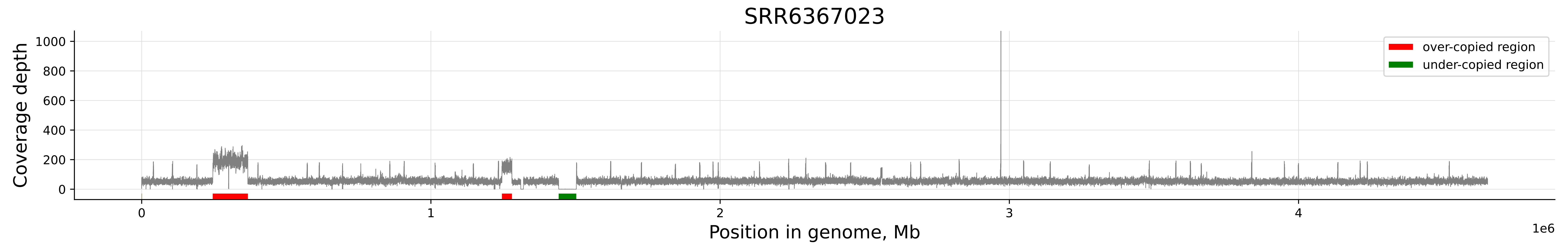

SRR6367023
over-copied region
under-copied region
Coverage depth
1000
800
600
400
200
0
0
1
2
3
4
1e6
Position in genome, Mb

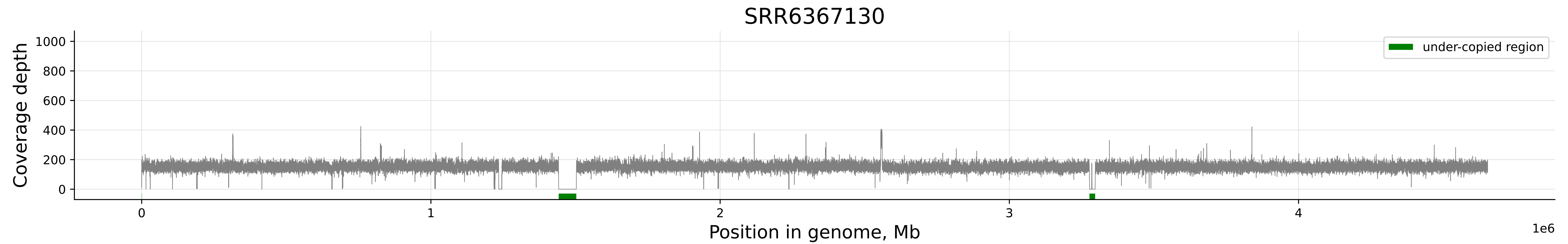
SRR6367130
Coverage depth
Position in genome, Mb
1e6
under-copied region
0
200
400
600
800
1000
0
1
2
3
4
