# Supplementary material for: Systematic analysis of spontaneous tandem genome amplification events in Yersinia pestis
Source: PLoS One. 2025 Dec 31;20(12):e0338460. doi: 10.1371/journal.pone.0338460 (PMC12755819; doi:10.1371/journal.pone.0338460)
Supplement: S3 Data — (PDF) [file pone.0338460.s004.pdf]

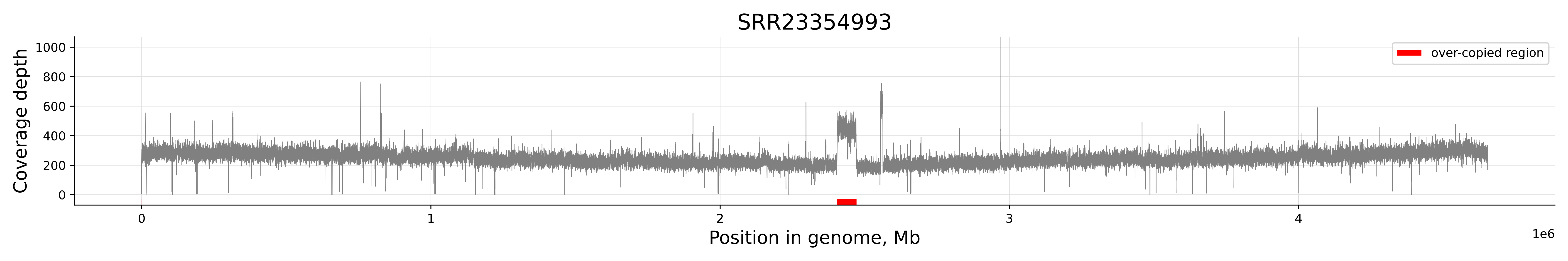
SRR23354993
Coverage depth
1000
800
600
400
200
0
over-copied region
0
1
2
3
4
1e6
Position in genome, Mb

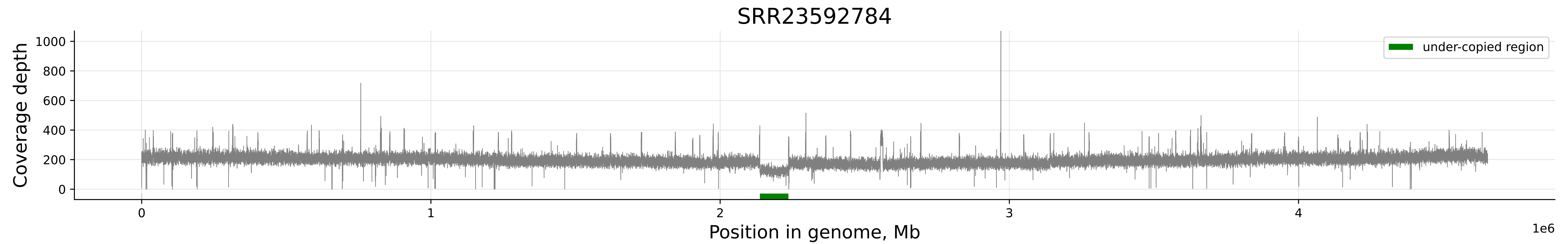
SRR23592784
under-copied region
Coverage depth
0
200
400
600
800
1000
0
1
2
3
4
1e6
Position in genome, Mb

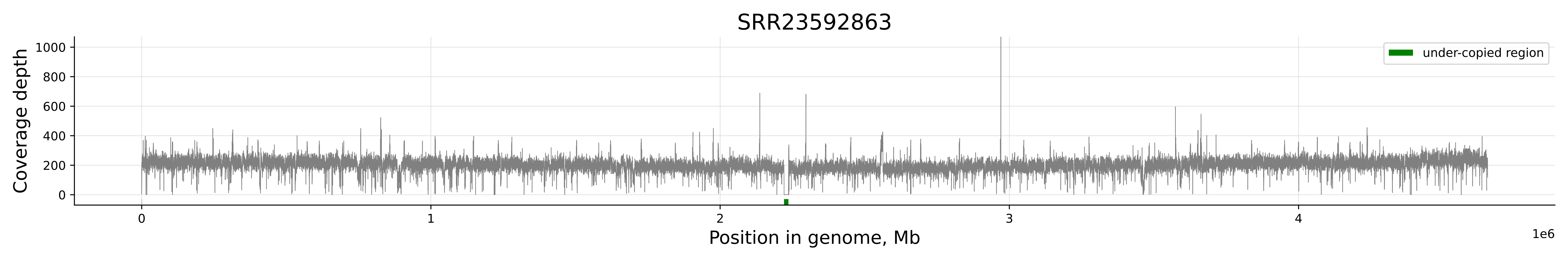

SRR23592863
Coverage depth
1000
800
600
400
200
0
under-copied region
0
1
2
3
4
1e6
Position in genome, Mb

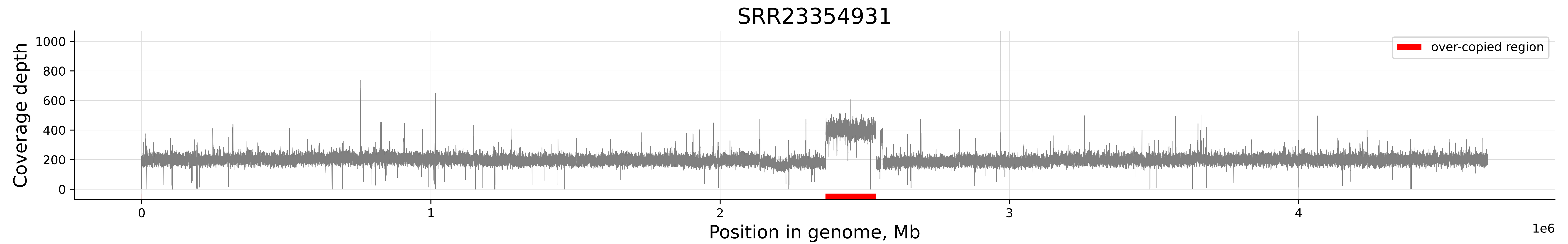
SRR23354931
over-copied region
Coverage depth
0
200
400
600
800
1000
0
1
2
3
4
1e6
Position in genome, Mb

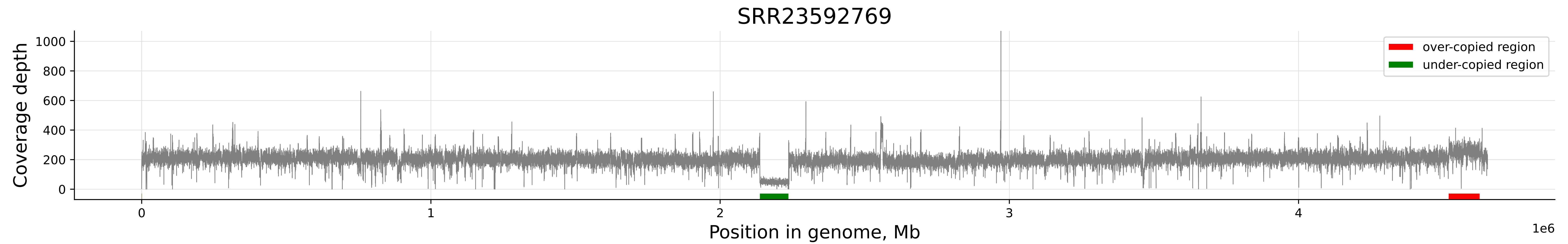
SRR23592769
Coverage depth
Position in genome, Mb
1e6
0
200
400
600
800
1000
0
1
2
3
4
over-copied region
under-copied region

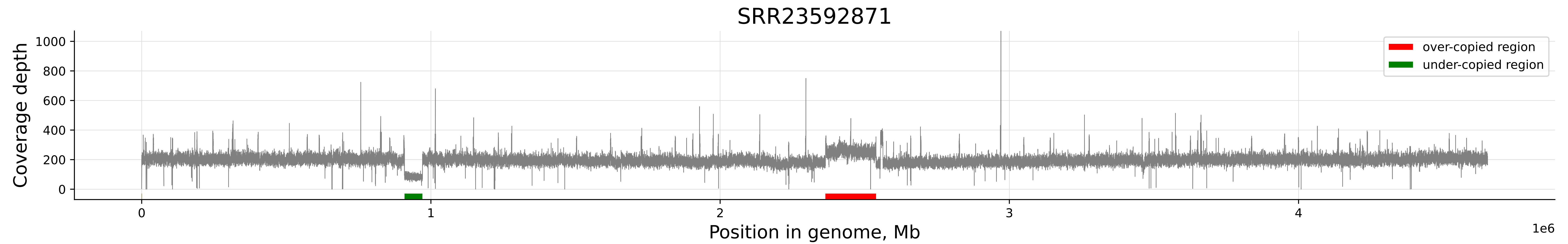
SRR23592871
Coverage depth
Position in genome, Mb
1e6
over-copied region
under-copied region
0
200
400
600
800
1000
0
1
2
3
4

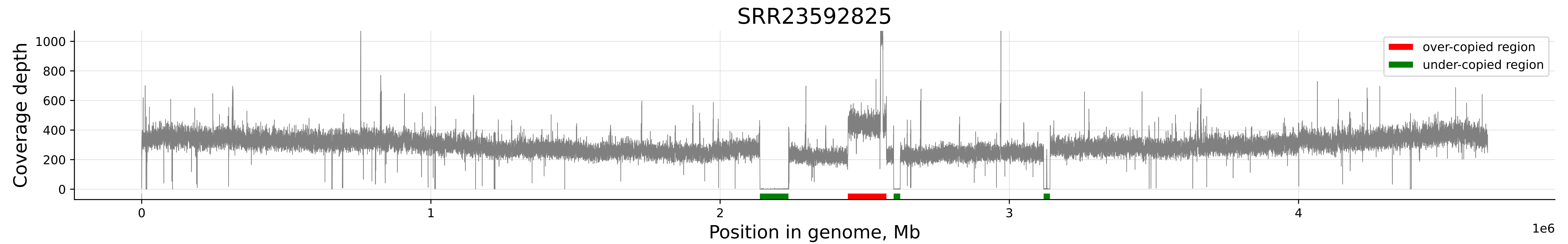
SRR23592825
Coverage depth
Position in genome, Mb
1e6
over-copied region
under-copied region
0
200
400
600
800
1000
0
1
2
3
4

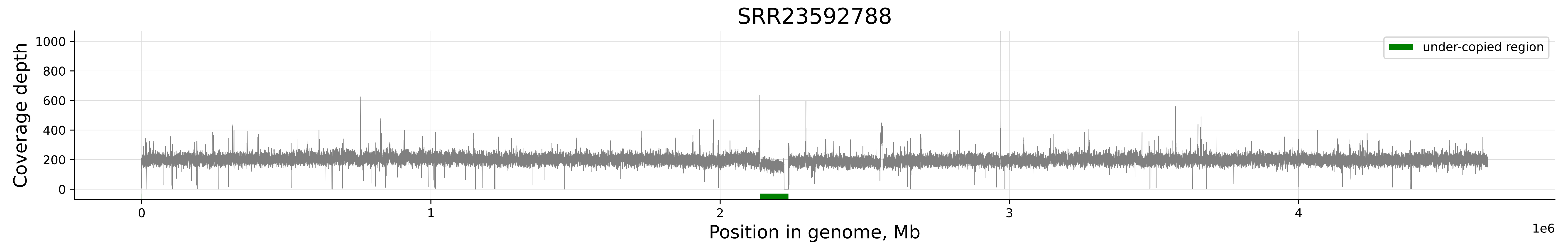
SRR23592788
under-copied region
Coverage depth
1000
800
600
400
200
0
0
1
2
3
4
1e6
Position in genome, Mb

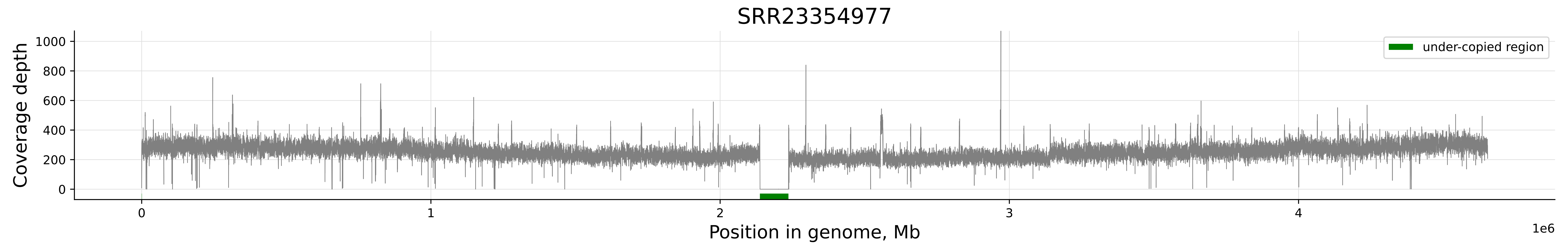
SRR23354977
under-copied region
Coverage depth
1000
800
600
400
200
0
0
1
2
3
4
Position in genome, Mb
1e6

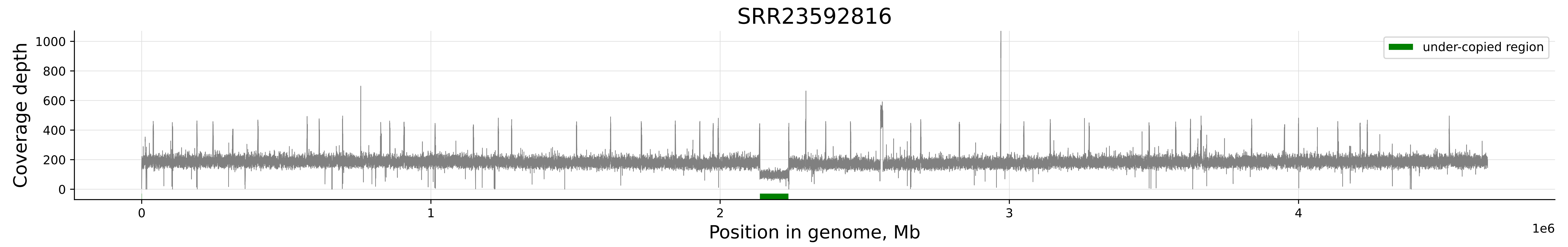
SRR23592816
Coverage depth
1000
800
600
400
200
0
under-copied region
0
1
2
3
4
1e6
Position in genome, Mb

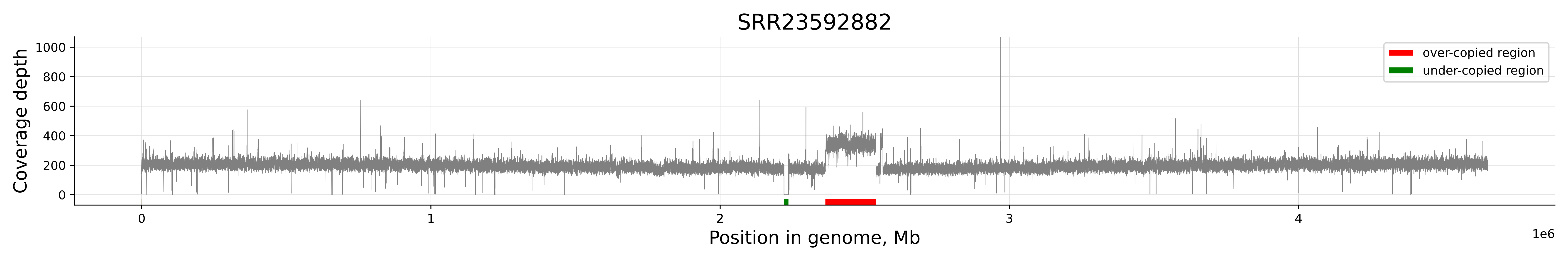
SRR23592882
Coverage depth
Position in genome, Mb
1e6
over-copied region
under-copied region
0
200
400
600
800
1000
0
1
2
3
4

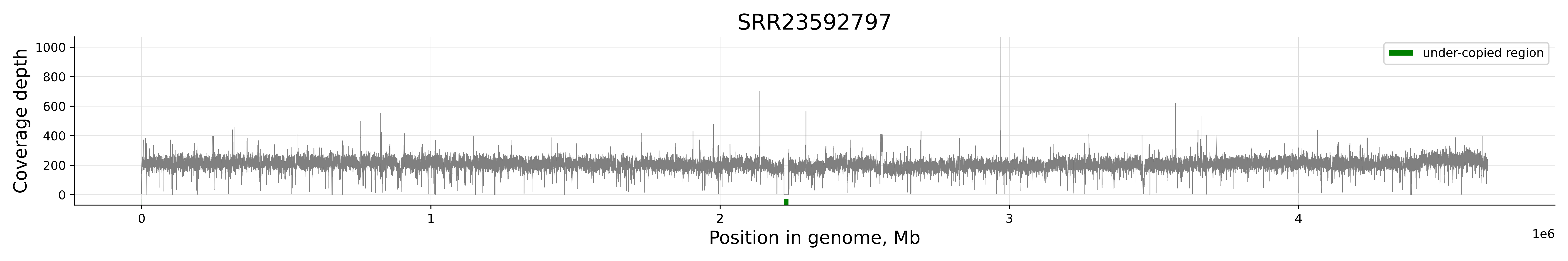
SRR23592797
Coverage depth
0
200
400
600
800
1000
under-copied region
0
1
2
3
4
1e6
Position in genome, Mb

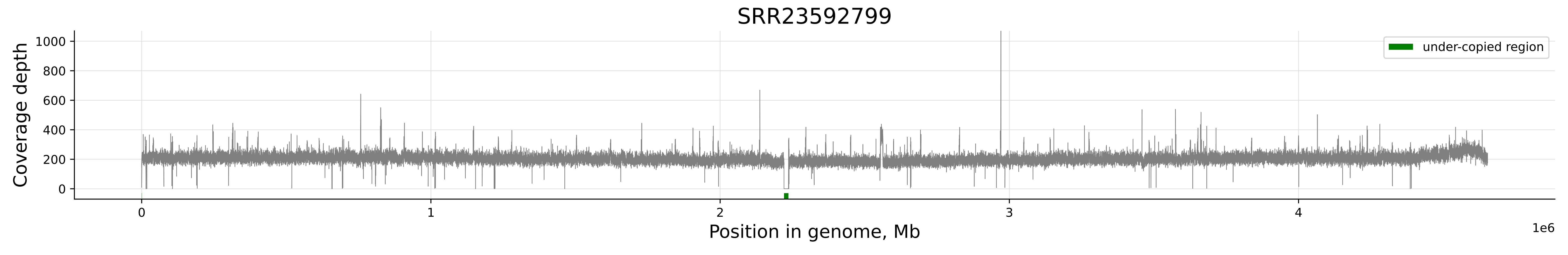

SRR23592799
under-copied region
Coverage depth
1000
800
600
400
200
0
0
1
2
3
4
Position in genome, Mb
1e6

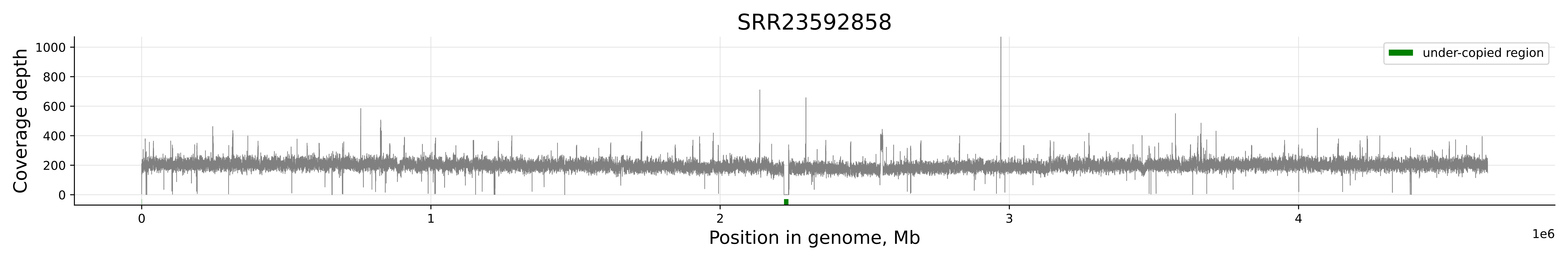
SRR23592858
Coverage depth
Position in genome, Mb
under-copied region
0
200
400
600
800
1000
0
1
2
3
4
1e6

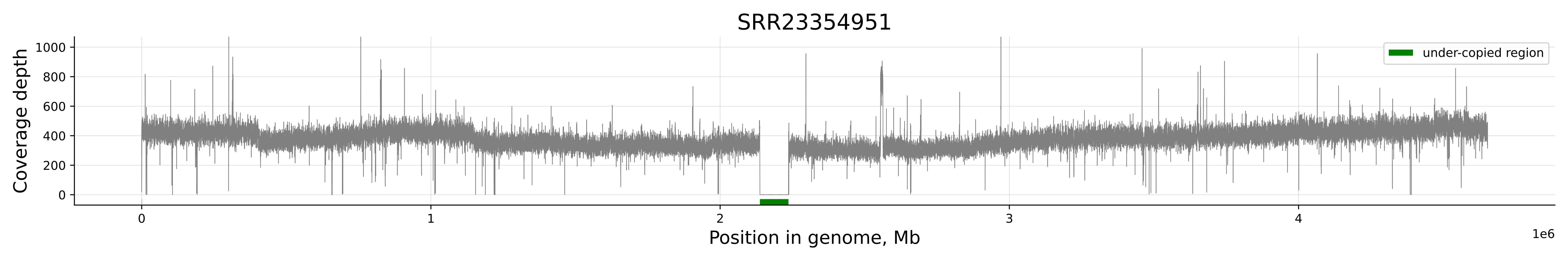

SRR23354951
under-copied region
Coverage depth
0
200
400
600
800
1000
Position in genome, Mb
0
1
2
3
4
1e6

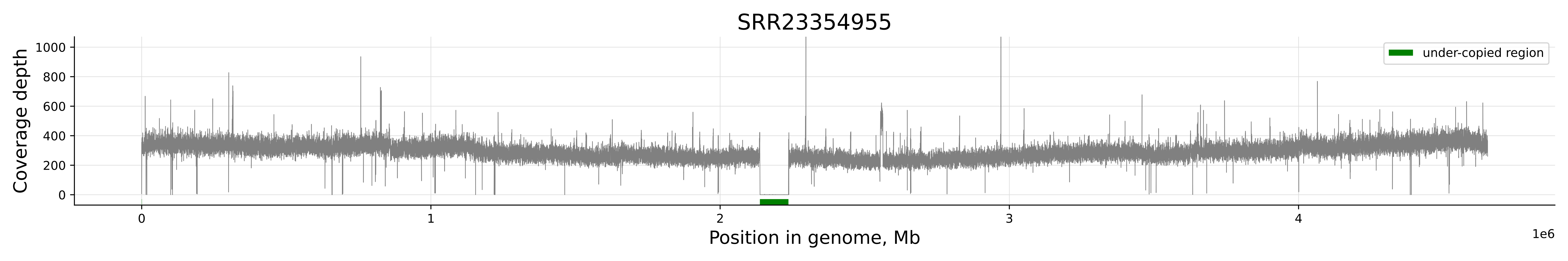
SRR23354955
Coverage depth
Position in genome, Mb
under-copied region
0
200
400
600
800
1000
1
2
3
4
1e6

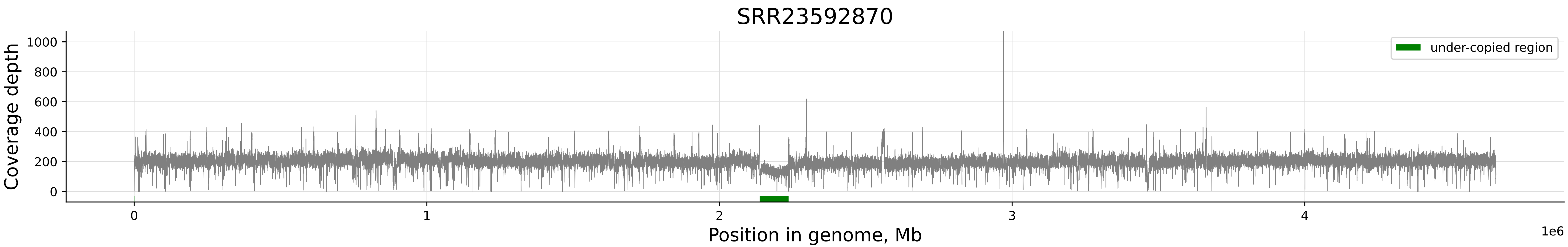

SRR23592870
under-copied region
Coverage depth
0
200
400
600
800
1000
Position in genome, Mb
0
1
2
3
4
1e6

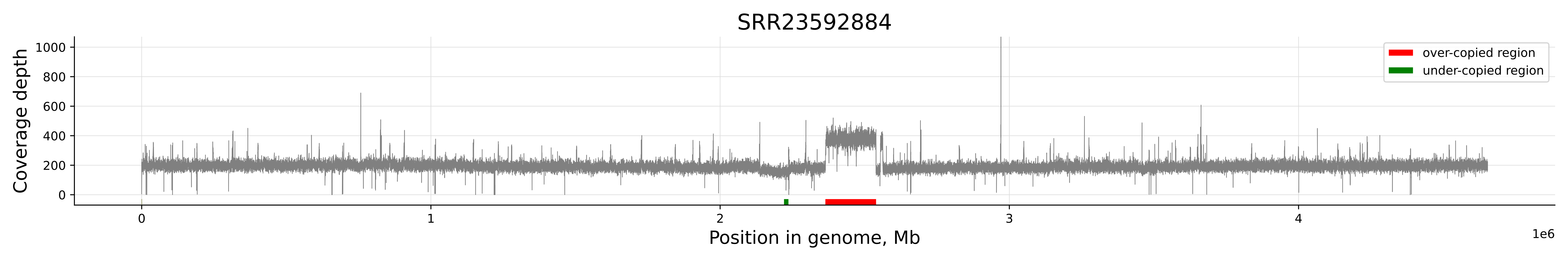

SRR23592884
Coverage depth
1000
800
600
400
200
0
over-copied region
under-copied region
0
1
2
3
4
1e6
Position in genome, Mb

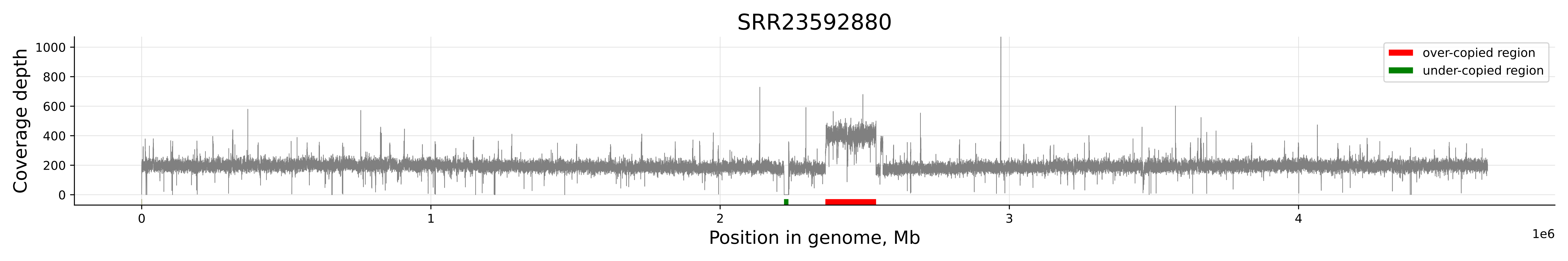
SRR23592880
Coverage depth
0
200
400
600
800
1000
0
1
2
3
4
1e6
Position in genome, Mb
over-copied region
under-copied region

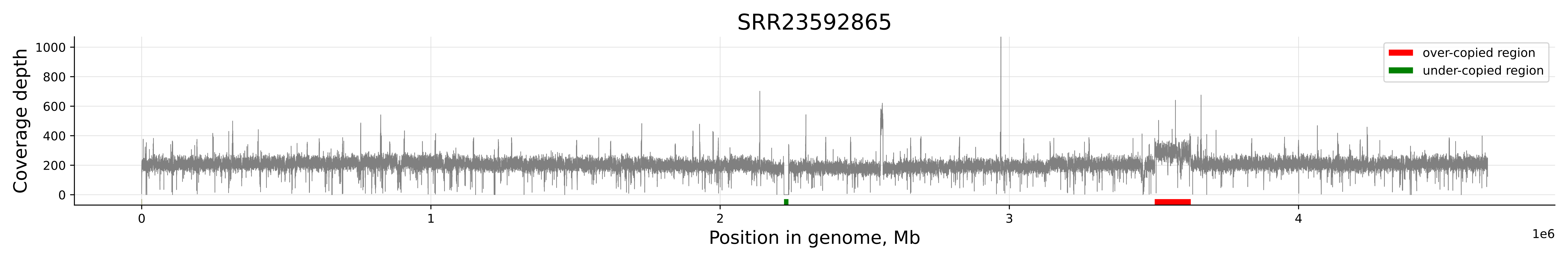
SRR23592865
Coverage depth
Position in genome, Mb
1e6
0
1
2
3
4
0
200
400
600
800
1000
over-copied region
under-copied region

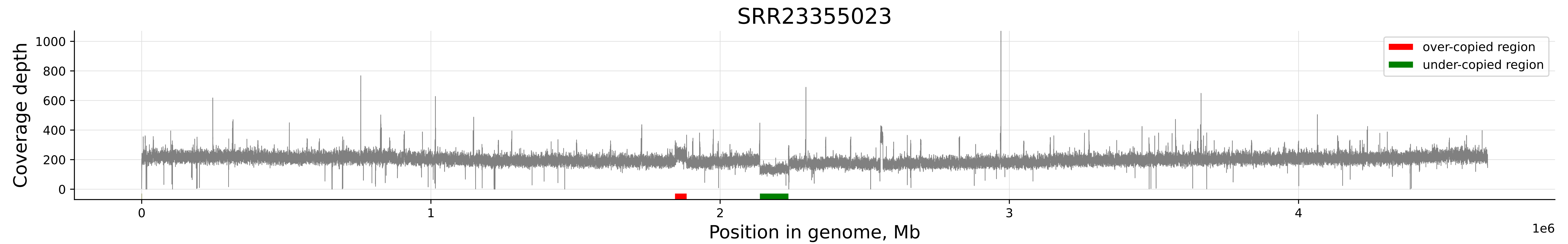

SRR23355023
over-copied region
under-copied region
Coverage depth
0
200
400
600
800
1000
0
1
2
3
4
1e6
Position in genome, Mb

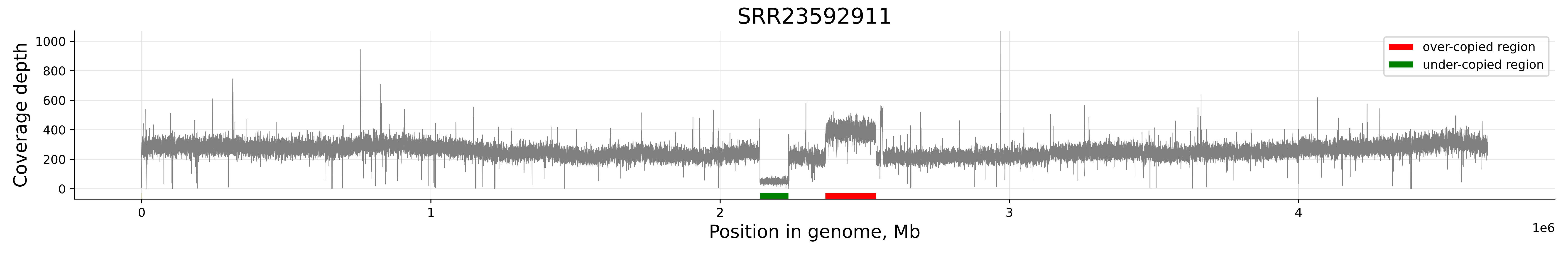
SRR23592911
Coverage depth
1000
800
600
400
200
0
0
1
2
3
4
1e6
Position in genome, Mb
over-copied region
under-copied region

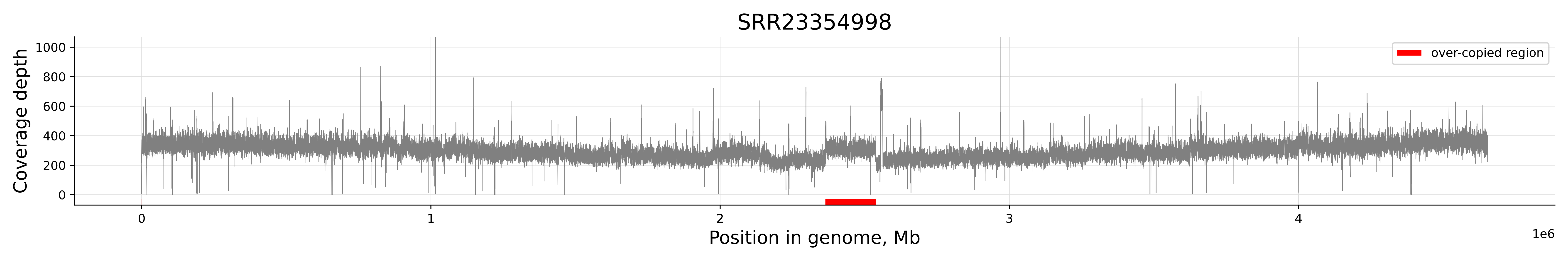
SRR23354998
Coverage depth
Position in genome, Mb
1e6
over-copied region
0
200
400
600
800
1000
0
1
2
3
4

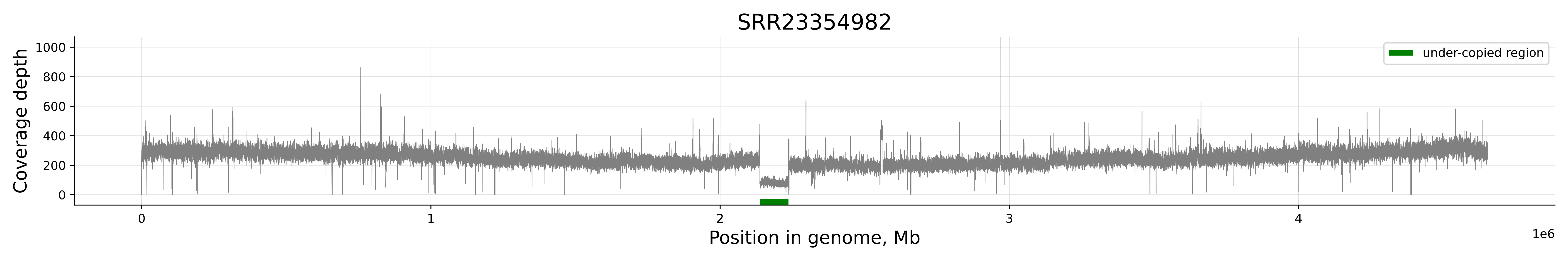

SRR23354982
Coverage depth
0
200
400
600
800
1000
under-copied region
0
1
2
3
4
1e6
Position in genome, Mb

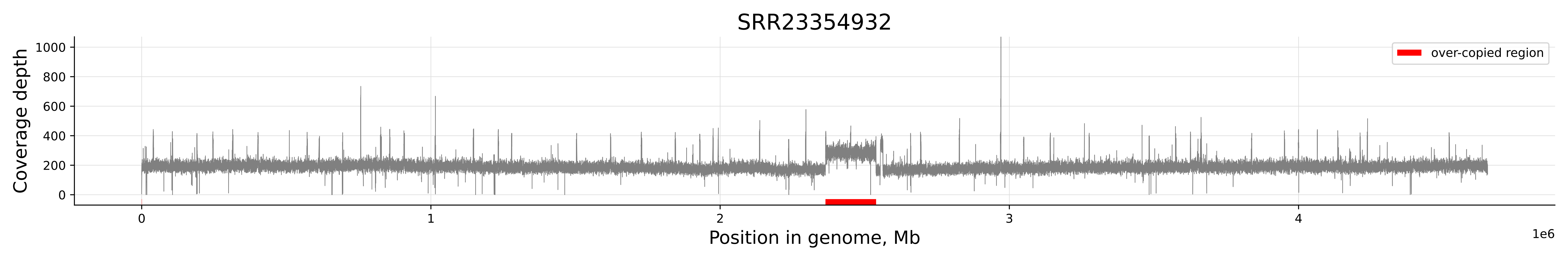

SRR23354932
over-copied region
Coverage depth
0
200
400
600
800
1000
0
1
2
3
4
1e6
Position in genome, Mb

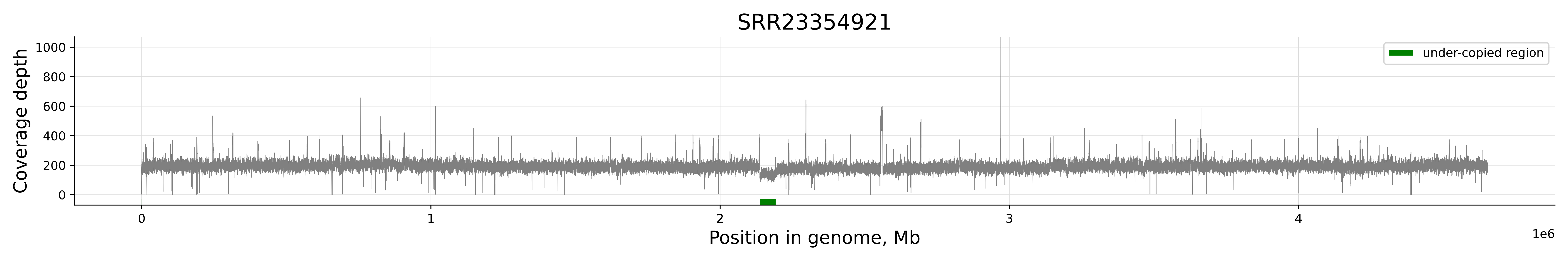
SRR23354921
under-copied region
Coverage depth
1000
800
600
400
200
0
0
1
2
3
4
1e6
Position in genome, Mb

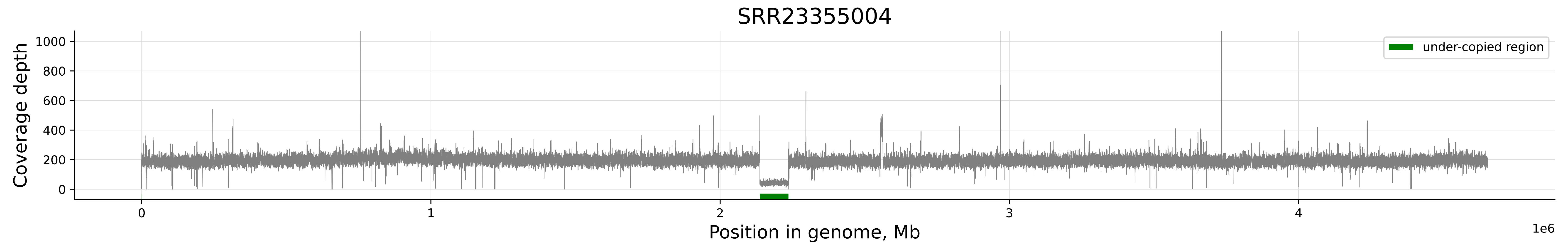

SRR23355004
Coverage depth
1000
800
600
400
200
0
0
1
2
3
4
1e6
Position in genome, Mb
under-copied region

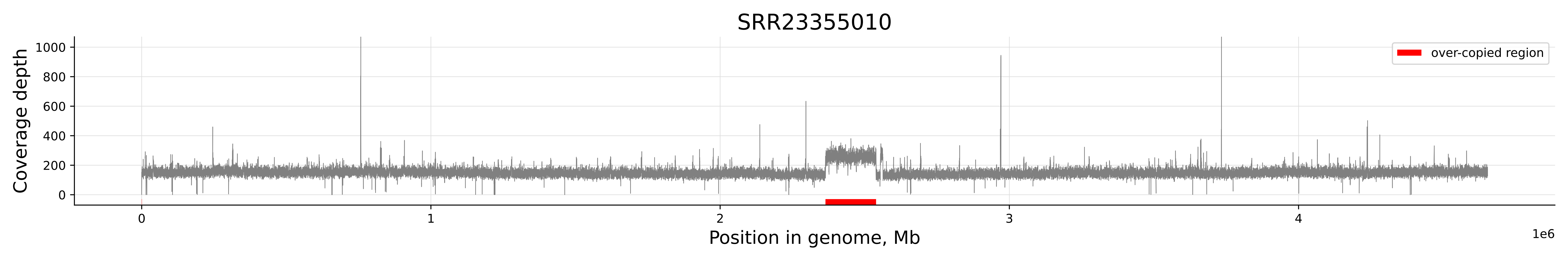

SRR23355010
Coverage depth
1000
800
600
400
200
0
over-copied region
0
1
2
3
4
1e6
Position in genome, Mb

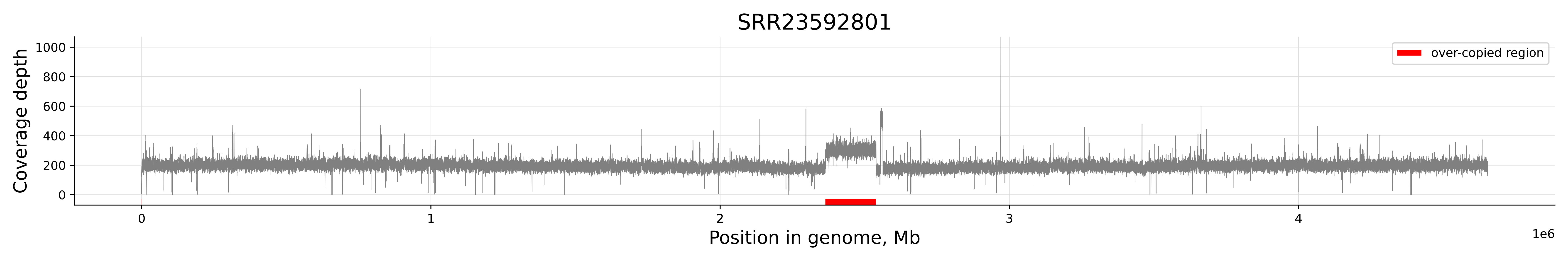

SRR23592801
Coverage depth
1000
800
600
400
200
0
0
1
2
3
4
1e6
Position in genome, Mb
over-copied region

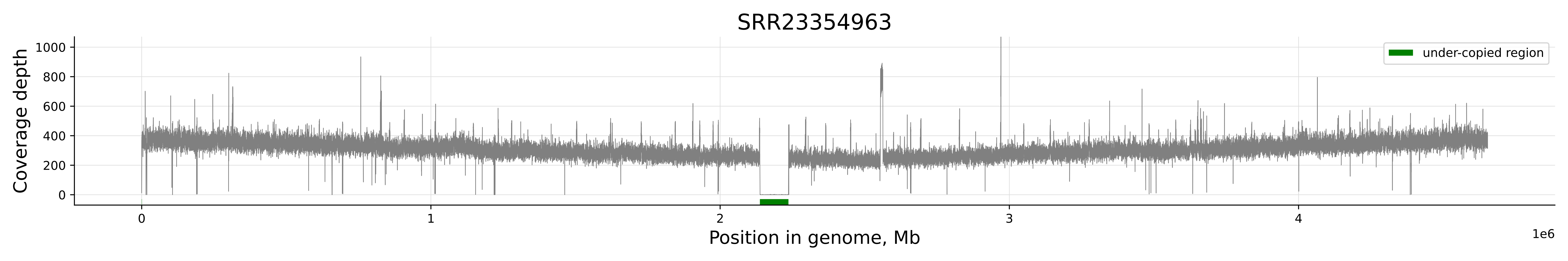

SRR23354963
Coverage depth
Position in genome, Mb
under-copied region
1000
800
600
400
200
0
0
1
2
3
4
1e6

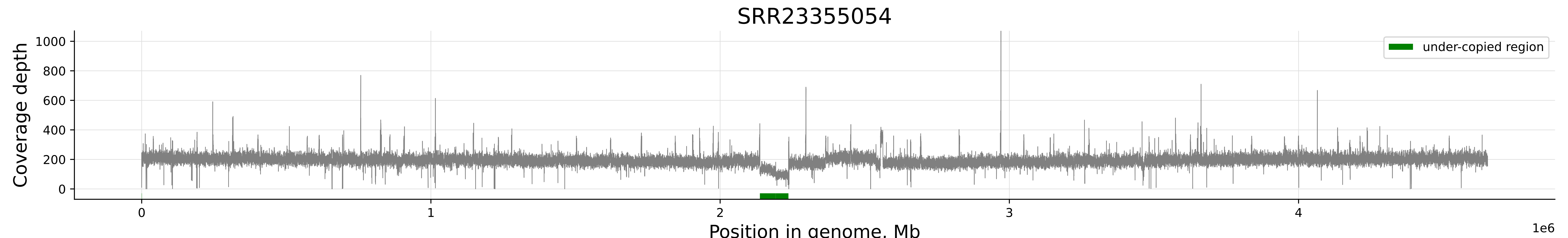
SRR23355054
under-copied region
Coverage depth
1000
800
600
400
200
0
0
1
2
3
4
Position in genome, Mb
1e6

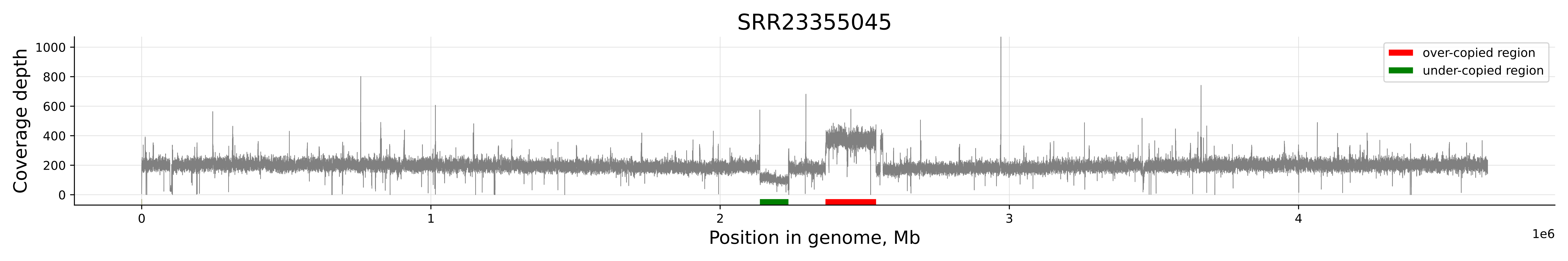

SRR23355045
Coverage depth
1000
800
600
400
200
0
0
1
2
3
4
1e6
Position in genome, Mb
over-copied region
under-copied region

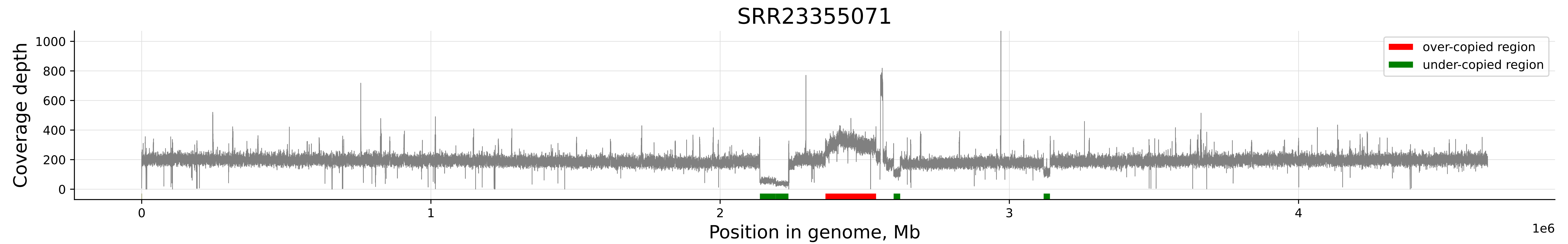
SRR23355071
Coverage depth
0
200
400
600
800
1000
Position in genome, Mb
0
1
2
3
4
1e6
over-copied region
under-copied region

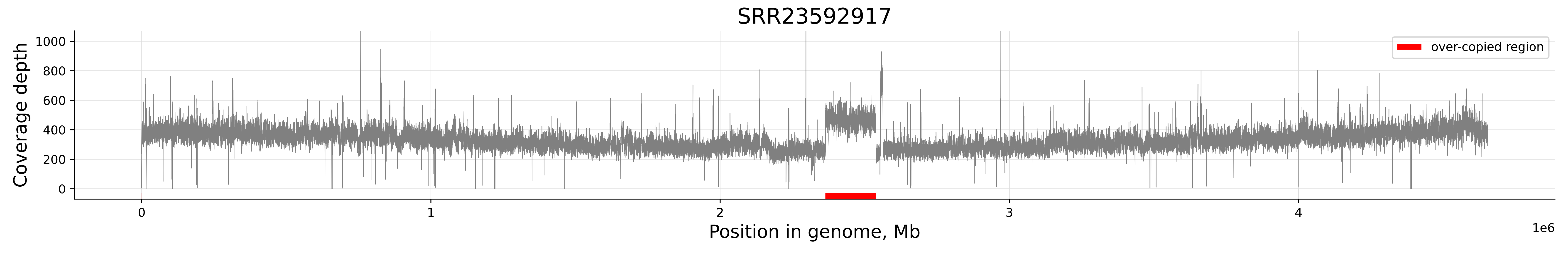

SRR23592917
Coverage depth
1000
800
600
400
200
0
0
1
2
3
4
1e6
Position in genome, Mb
over-copied region

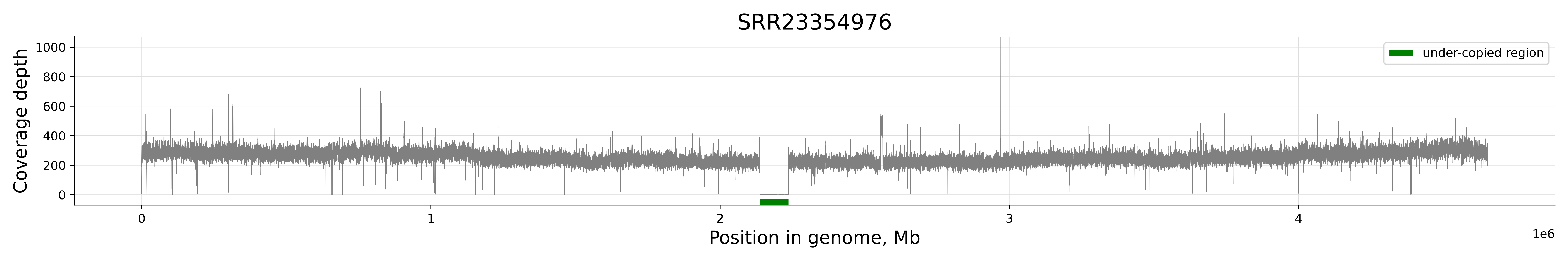
SRR23354976
Coverage depth
Position in genome, Mb
1e6
under-copied region
0
200
400
600
800
1000
0
1
2
3
4

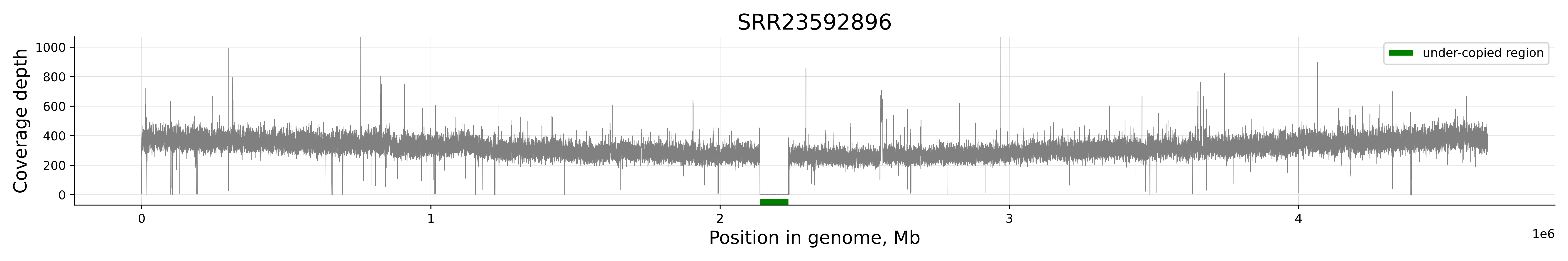

SRR23592896
Coverage depth
1000
800
600
400
200
0
0
1
2
3
4
1e6
Position in genome, Mb
under-copied region

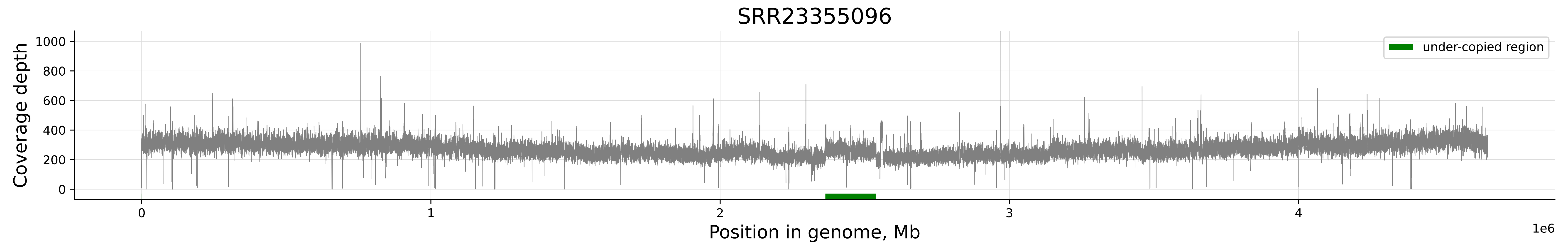

SRR23355096
Coverage depth
Position in genome, Mb
under-copied region
1e6

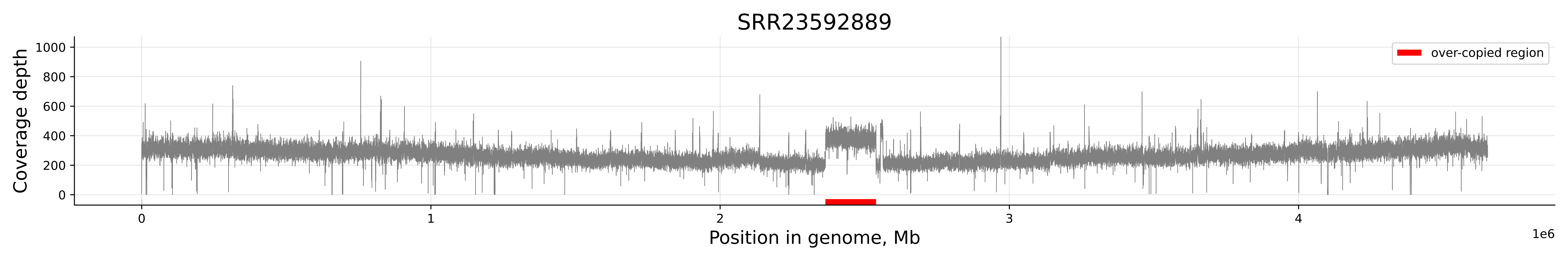

SRR23592889
Coverage depth
Position in genome, Mb
1e6
over-copied region
0
200
400
600
800
1000
0
1
2
3
4

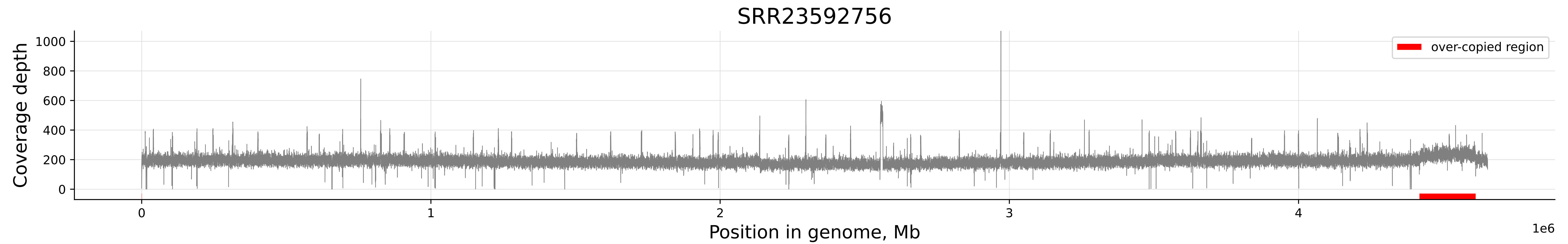

SRR23592756
Coverage depth
1000
800
600
400
200
0
over-copied region
0
1
2
3
4
1e6
Position in genome, Mb

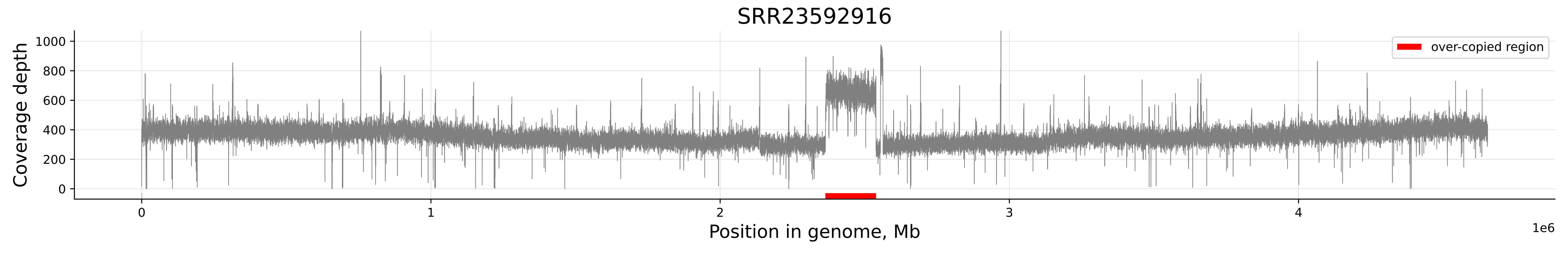

SRR23592916
over-copied region
Coverage depth
0
200
400
600
800
1000
Position in genome, Mb
0
1
2
3
4
1e6

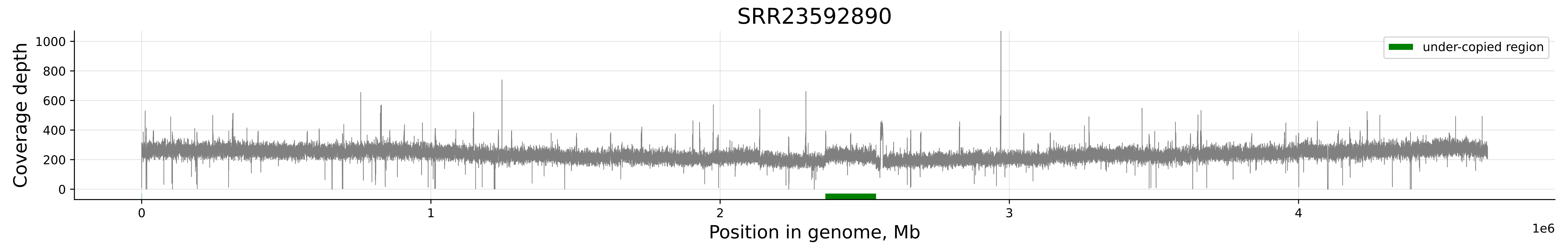
SRR23592890
Coverage depth
Position in genome, Mb
under-copied region
1000
800
600
400
200
0
0
1
2
3
4
1e6

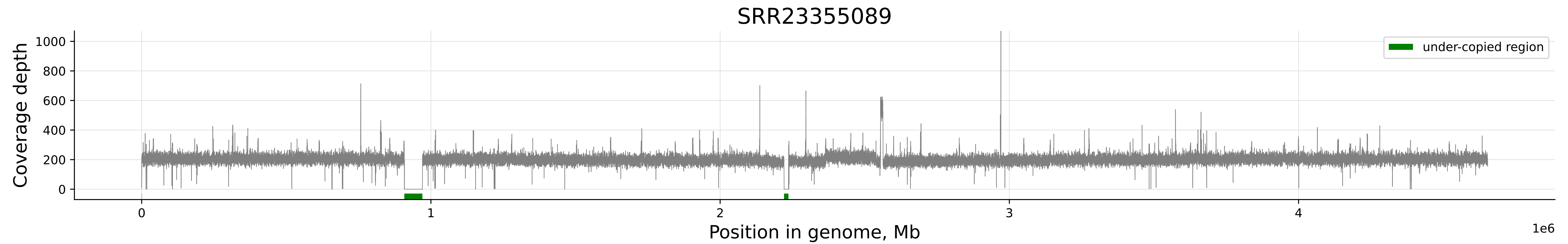
SRR23355089
Coverage depth
Position in genome, Mb
under-copied region
1e6
0
200
400
600
800
1000
0
1
2
3
4

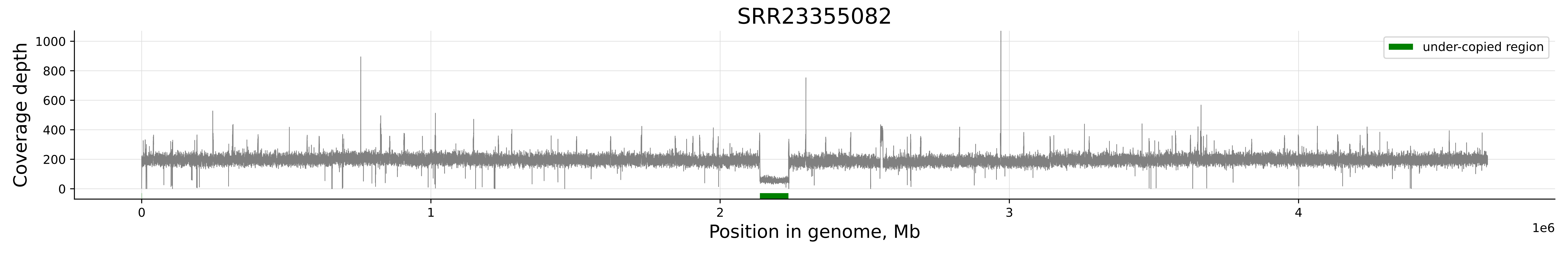
SRR23355082
Coverage depth
1000
800
600
400
200
0
under-copied region
0
1
2
3
4
1e6
Position in genome, Mb

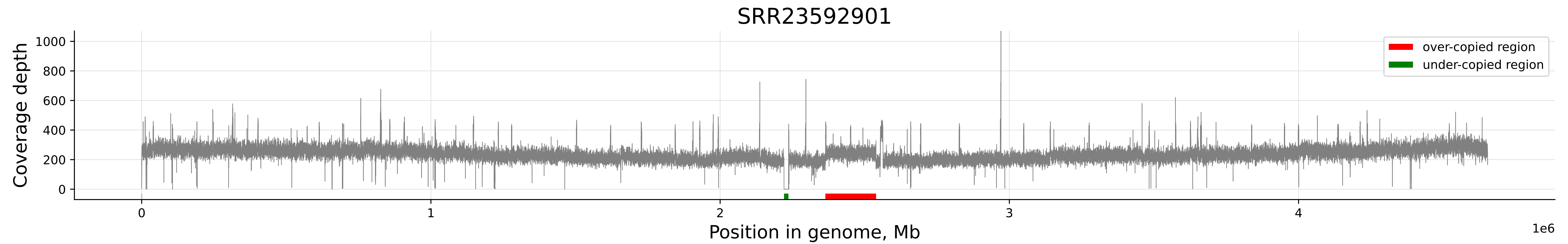

SRR23592901
Coverage depth
Position in genome, Mb
1e6
over-copied region
under-copied region
0
200
400
600
800
1000
0
1
2
3
4

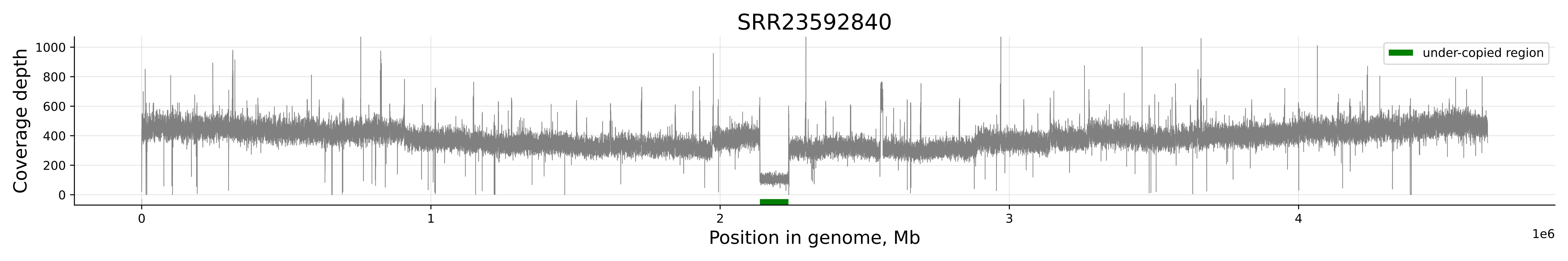

SRR23592840
Coverage depth
Position in genome, Mb
under-copied region
1000
800
600
400
200
0
0
1
2
3
4
1e6

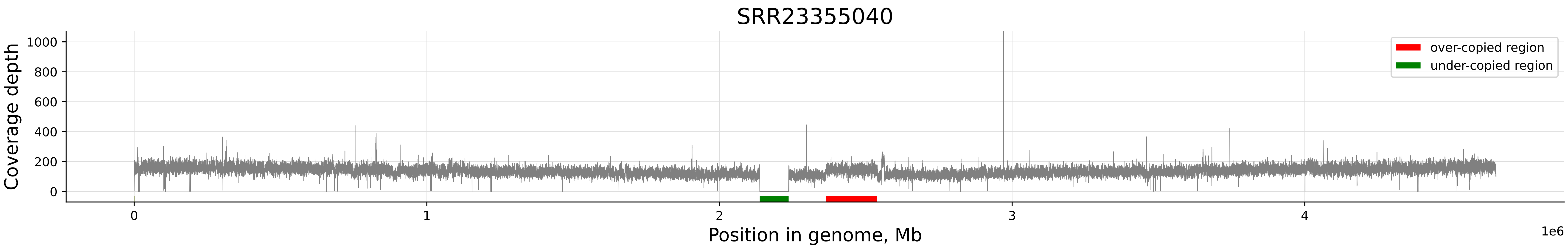

SRR23355040
over-copied region
under-copied region
Coverage depth
1000
800
600
400
200
0
0
1
2
3
4
1e6
Position in genome, Mb

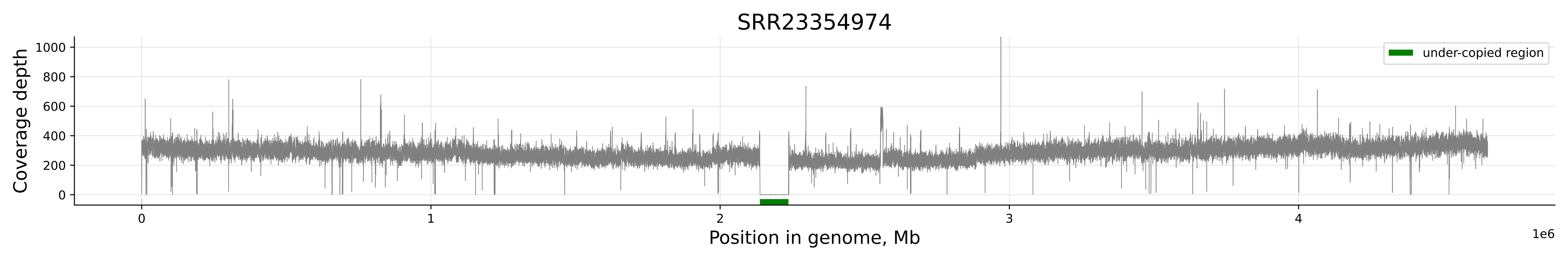

SRR23354974
Coverage depth
Position in genome, Mb
1e6
under-copied region
0
200
400
600
800
1000
0
1
2
3
4

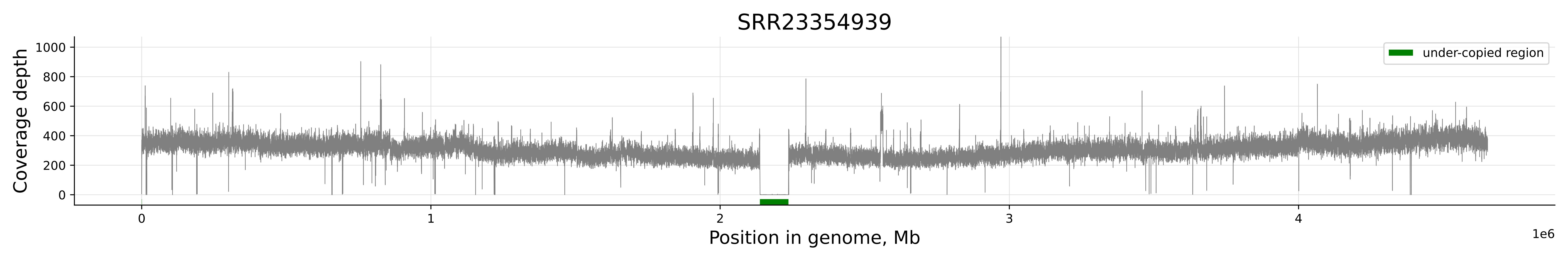
SRR23354939
Coverage depth
Position in genome, Mb
under-copied region
1000
800
600
400
200
0
0
1
2
3
4
1e6

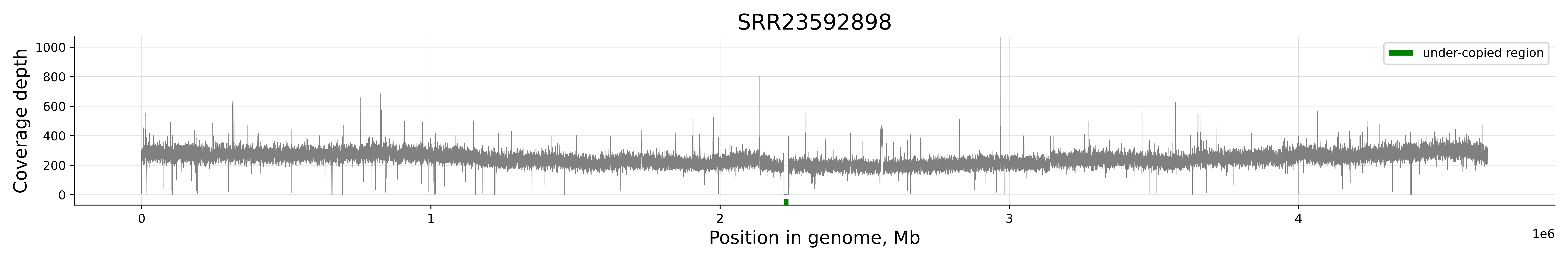

SRR23592898
Coverage depth
1000
800
600
400
200
0
under-copied region
0
1
2
3
4
1e6
Position in genome, Mb

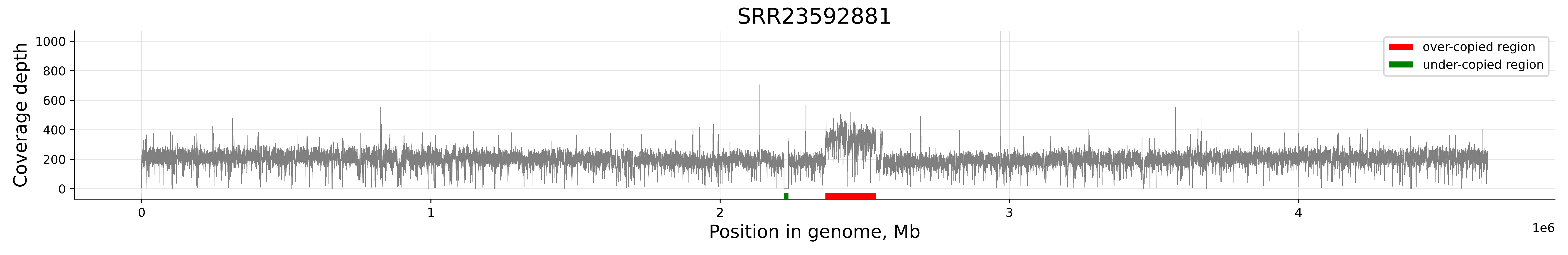
SRR23592881
Coverage depth
1000
800
600
400
200
0
over-copied region
under-copied region
0
1
2
3
4
1e6
Position in genome, Mb

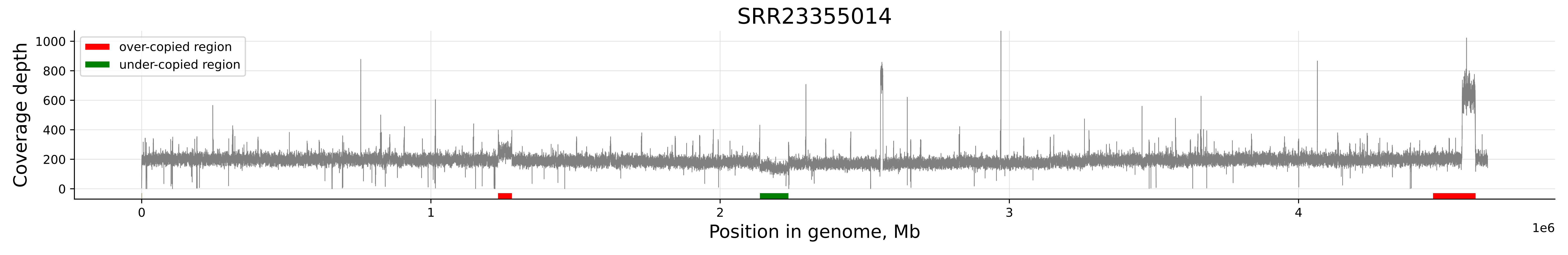

SRR23355014
over-copied region
under-copied region
Coverage depth
1000
800
600
400
200
0
0
1
2
3
4
Position in genome, Mb
1e6

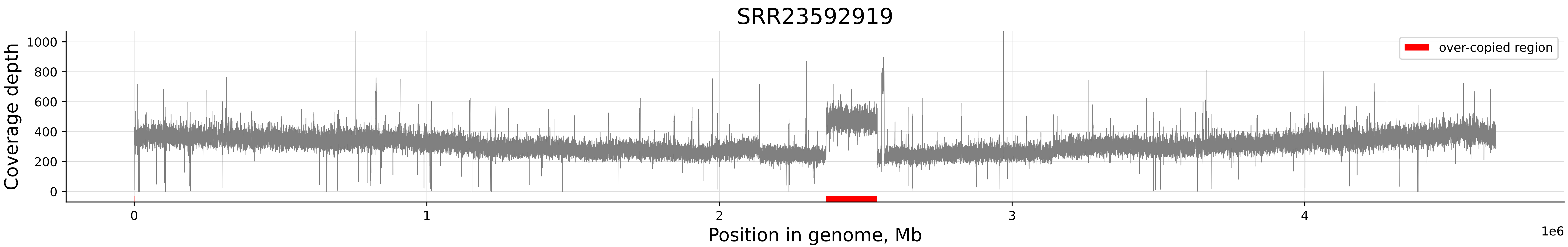
SRR23592919
over-copied region
Coverage depth
0
200
400
600
800
1000
0
1
2
3
4
1e6
Position in genome, Mb

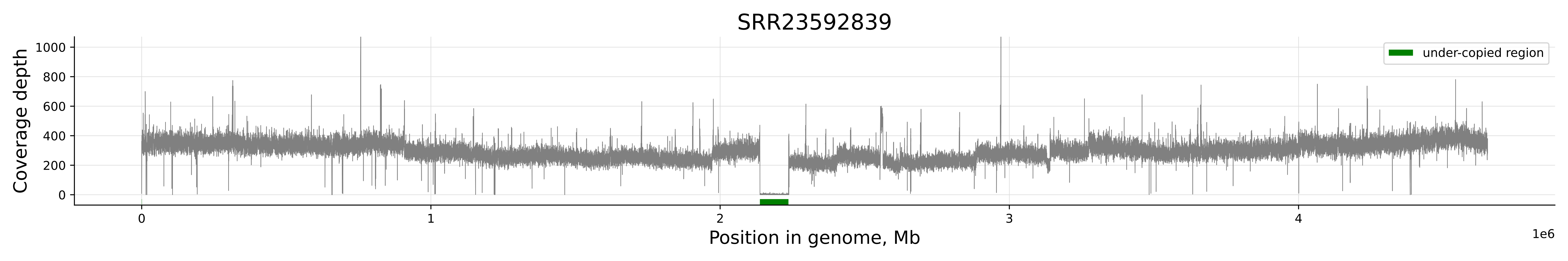

SRR23592839
Coverage depth
1000
800
600
400
200
0
under-copied region
0
1
2
3
4
1e6
Position in genome, Mb

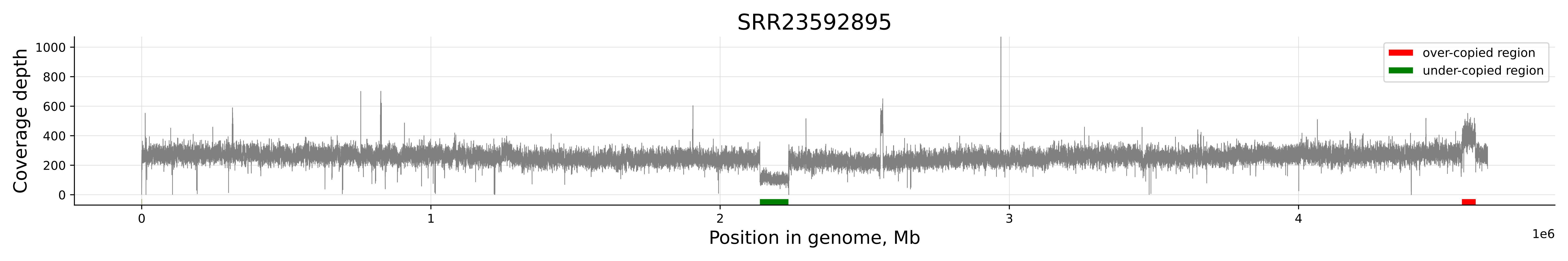

SRR23592895
Coverage depth
1000
800
600
400
200
0
over-copied region
under-copied region
0
1
2
3
4
1e6
Position in genome, Mb

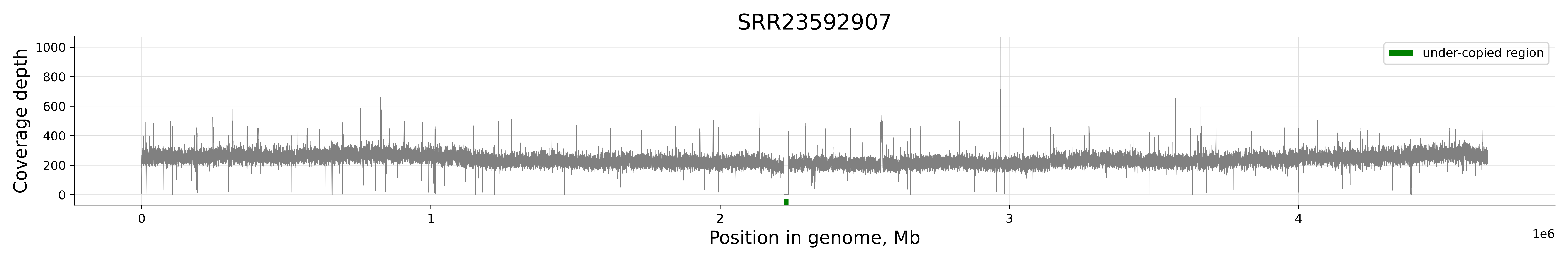

SRR23592907
Coverage depth
Position in genome, Mb
1e6
under-copied region
0
200
400
600
800
1000
0
1
2
3
4

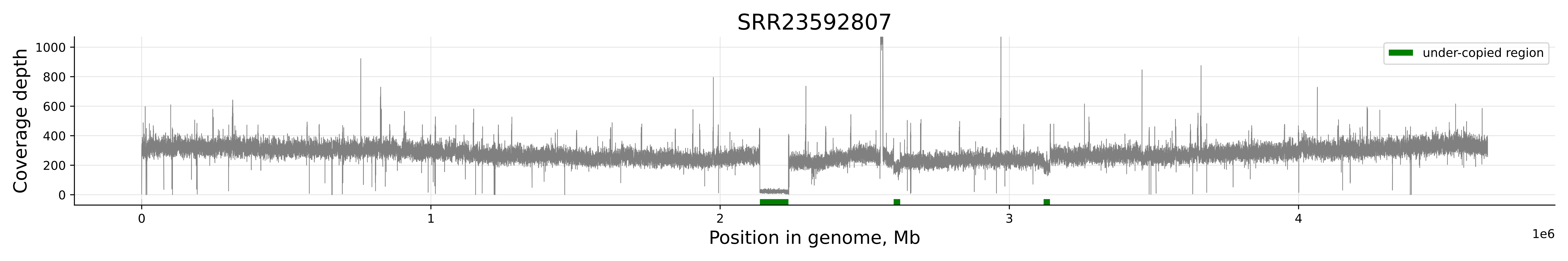

SRR23592807
Coverage depth
Position in genome, Mb
1e6
under-copied region
0
200
400
600
800
1000
0
1
2
3
4

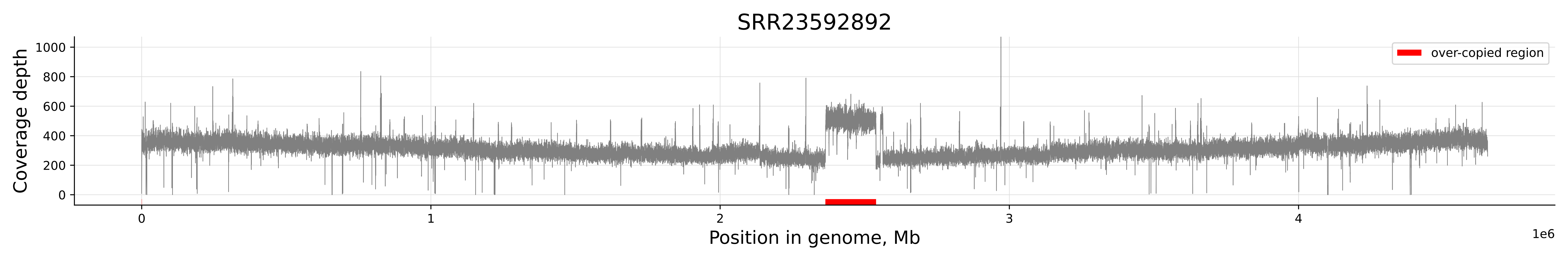

SRR23592892
Coverage depth
Position in genome, Mb
1e6
over-copied region
0
200
400
600
800
1000
0
1
2
3
4

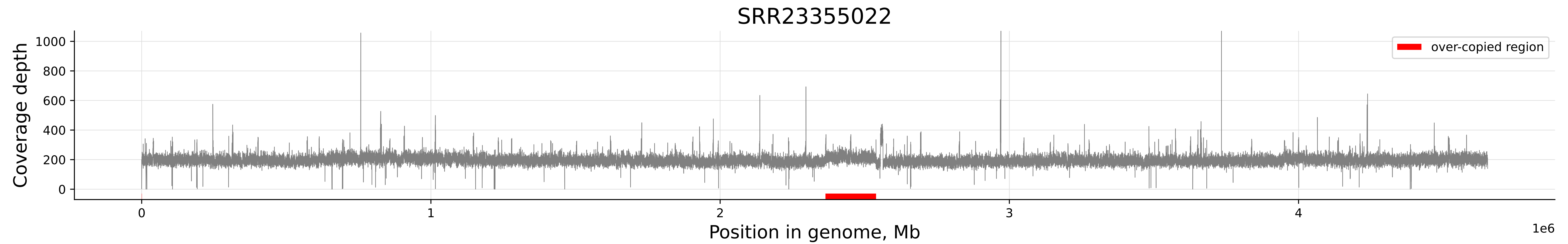

SRR23355022
Coverage depth
0
200
400
600
800
1000
over-copied region
0
1
2
3
4
1e6
Position in genome, Mb

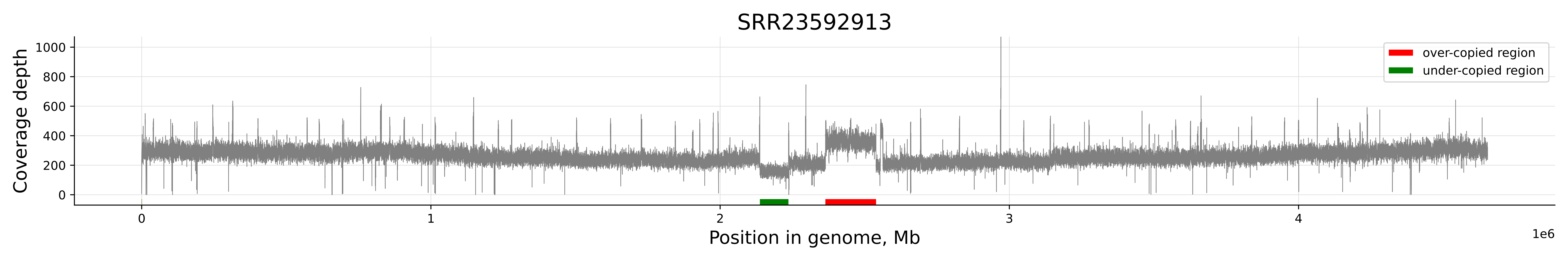

SRR23592913
Coverage depth
Position in genome, Mb
1e6
over-copied region
under-copied region
0
200
400
600
800
1000
0
1
2
3
4

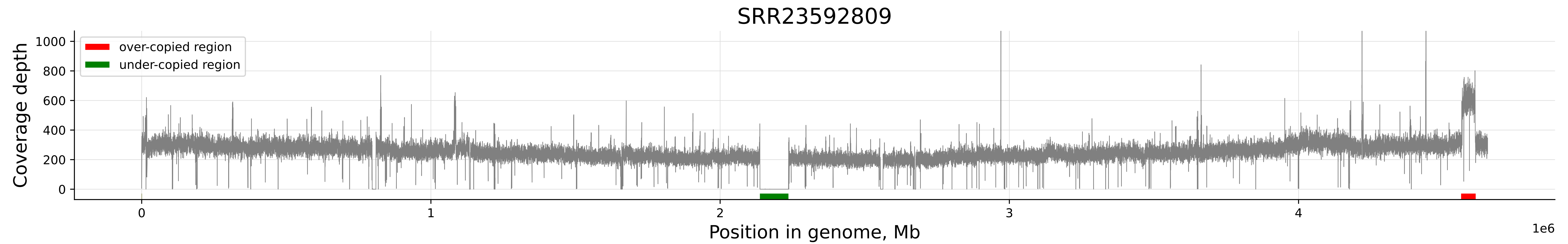

SRR23592809
over-copied region
under-copied region
Coverage depth
0
200
400
600
800
1000
0
1
2
3
4
1e6
Position in genome, Mb

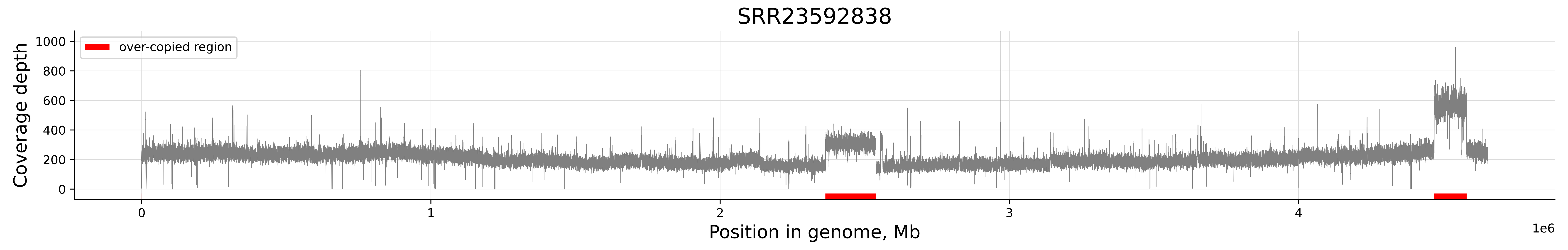
SRR23592838
over-copied region
Coverage depth
0
200
400
600
800
1000
0
1
2
3
4
1e6
Position in genome, Mb

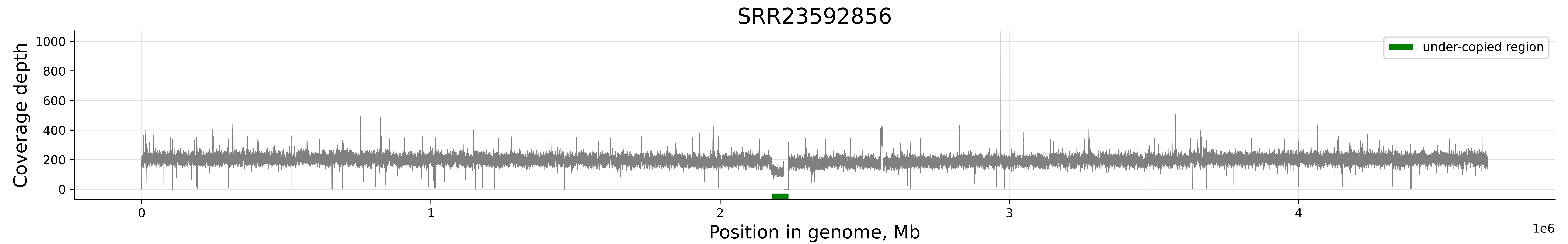

SRR23592856
Coverage depth
1000
800
600
400
200
0
under-copied region
0
1
2
3
4
1e6
Position in genome, Mb

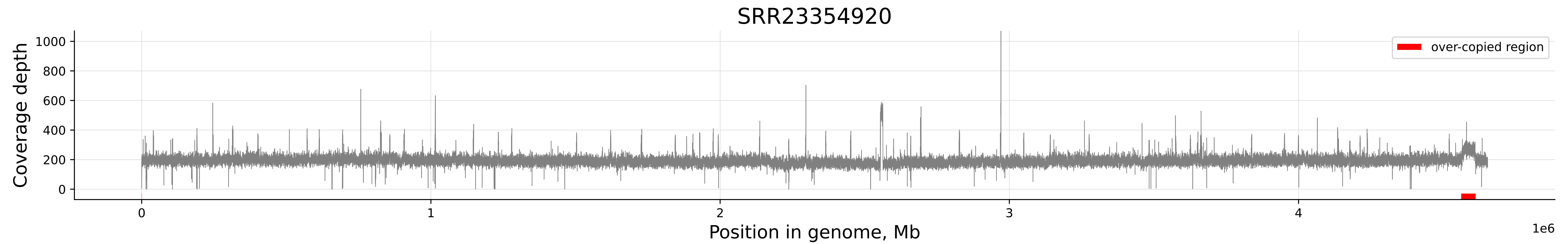
SRR23354920
Coverage depth
Position in genome, Mb
over-copied region
1e6
0
200
400
600
800
1000
0
1
2
3
4

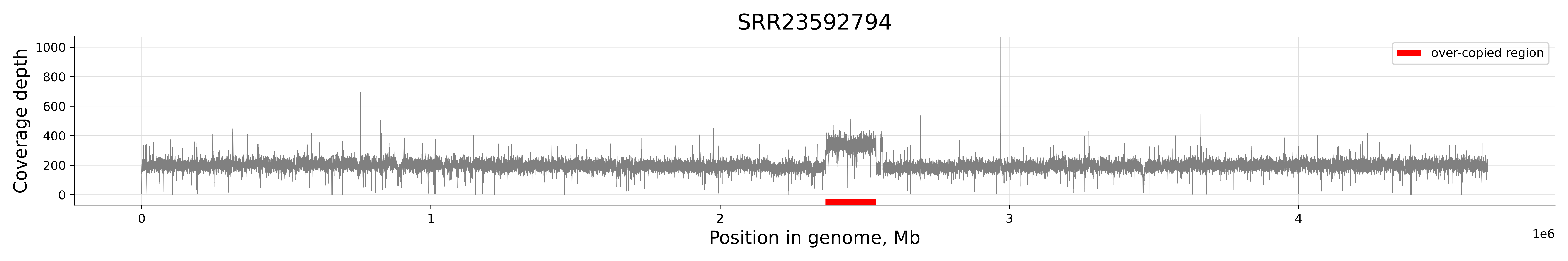

SRR23592794
Coverage depth
1000
800
600
400
200
0
over-copied region
0
1
2
3
4
1e6
Position in genome, Mb

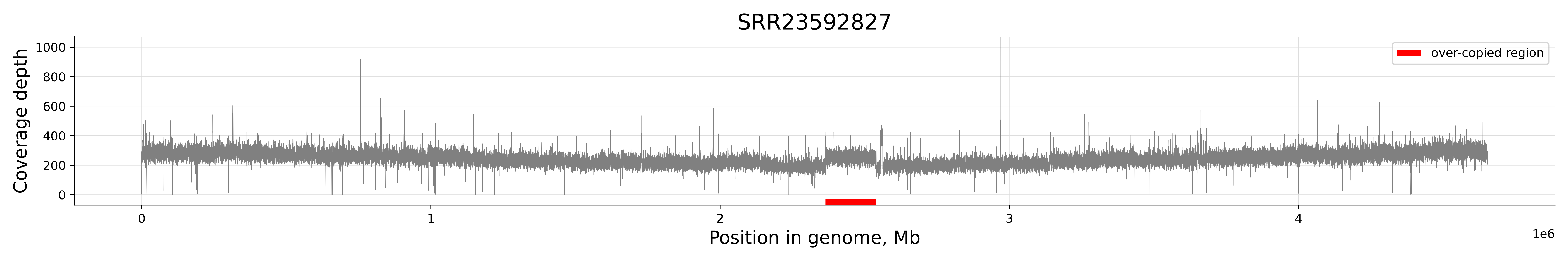

SRR23592827
Coverage depth
1000
800
600
400
200
0
over-copied region
0
1
2
3
4
Position in genome, Mb
1e6

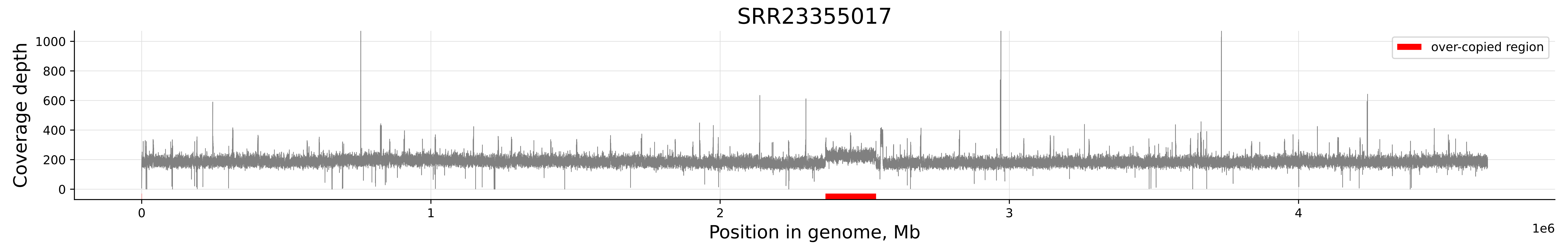

SRR23355017
Coverage depth
0
200
400
600
800
1000
over-copied region
0
1
2
3
4
1e6
Position in genome, Mb

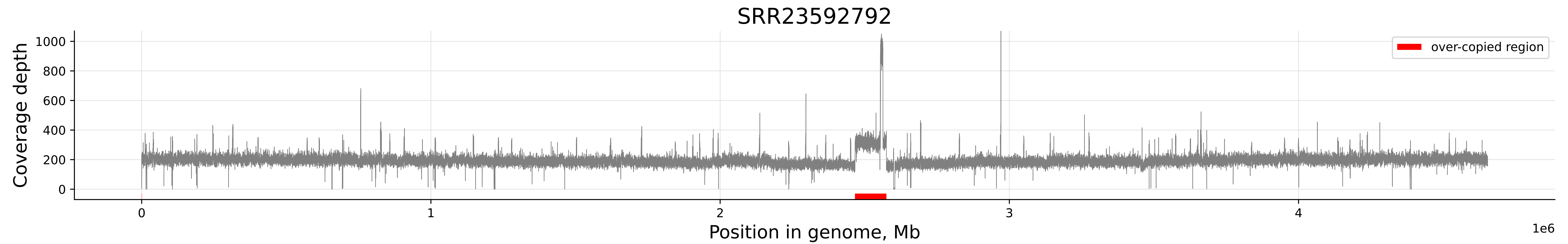
SRR23592792
Coverage depth
Position in genome, Mb
1e6
over-copied region
0
200
400
600
800
1000
0
1
2
3
4

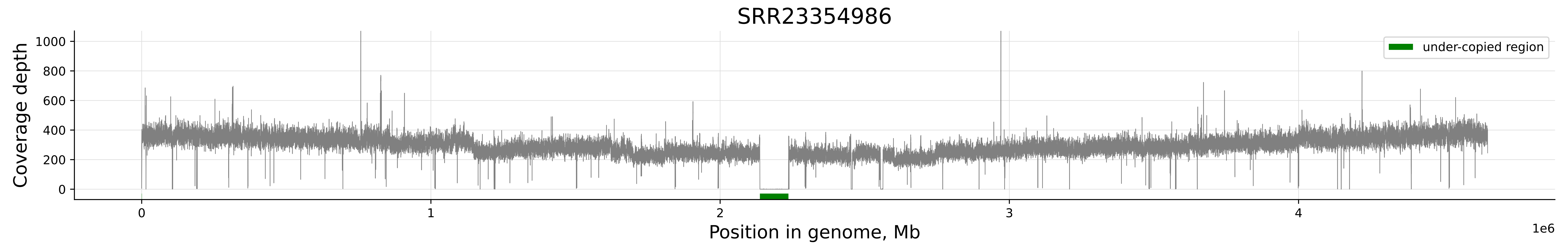
SRR23354986
Coverage depth
Position in genome, Mb
1e6
under-copied region
0
200
400
600
800
1000
0
1
2
3
4

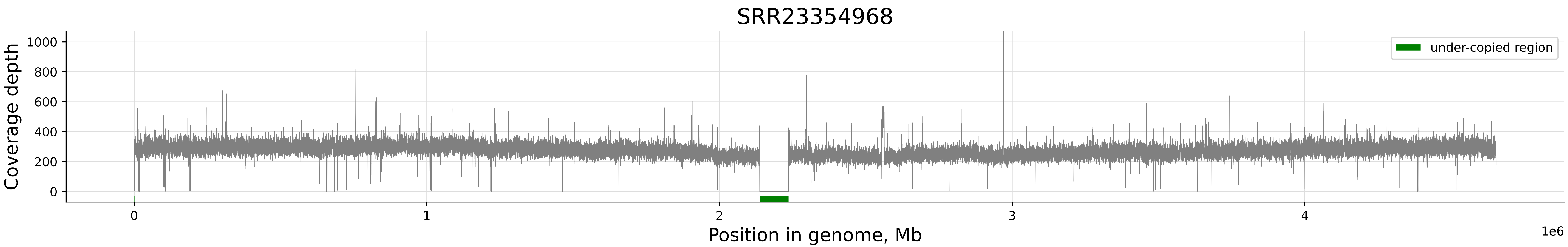

SRR23354968
under-copied region
Coverage depth
1000
800
600
400
200
0
0
1
2
3
4
1e6
Position in genome, Mb

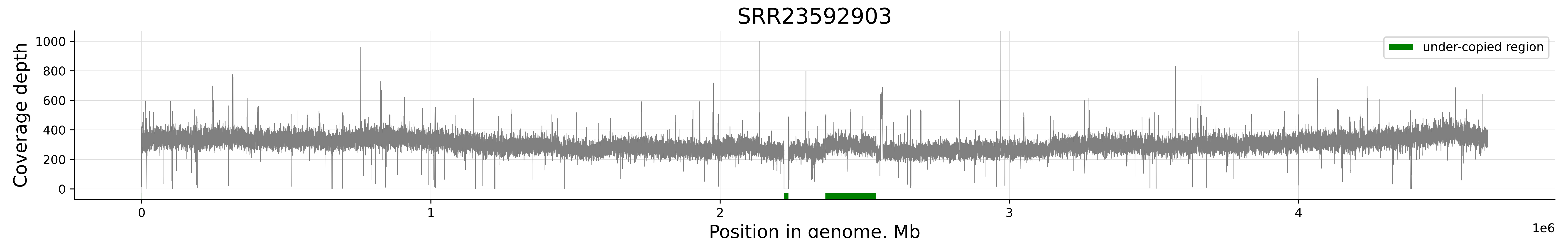

SRR23592903
Coverage depth
Position in genome, Mb
under-copied region
1e6

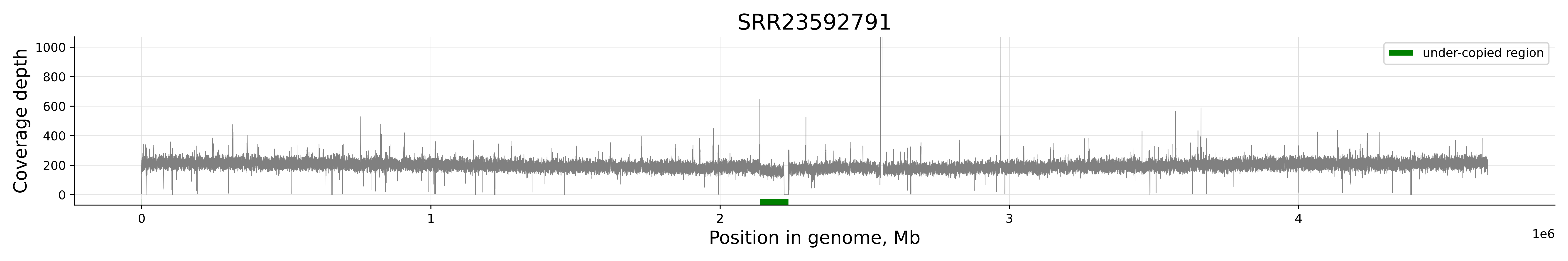
SRR23592791
Coverage depth
1000
800
600
400
200
0
under-copied region
0
1
2
3
4
1e6
Position in genome, Mb

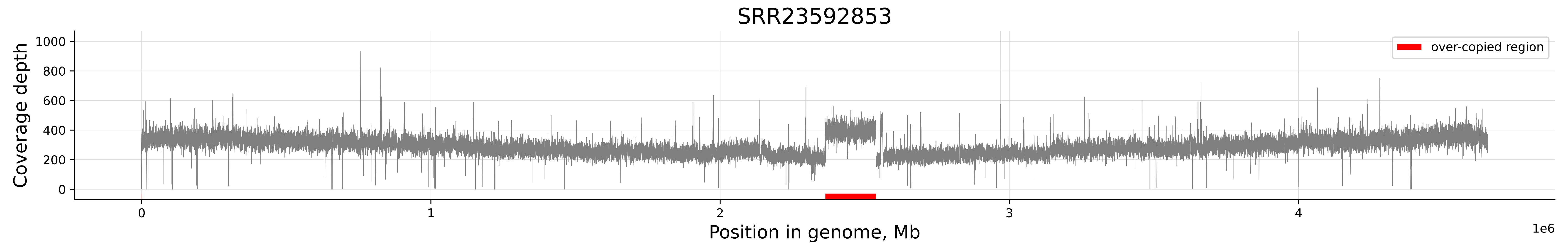

SRR23592853
over-copied region
Coverage depth
0
200
400
600
800
1000
0
1
2
3
4
1e6
Position in genome, Mb

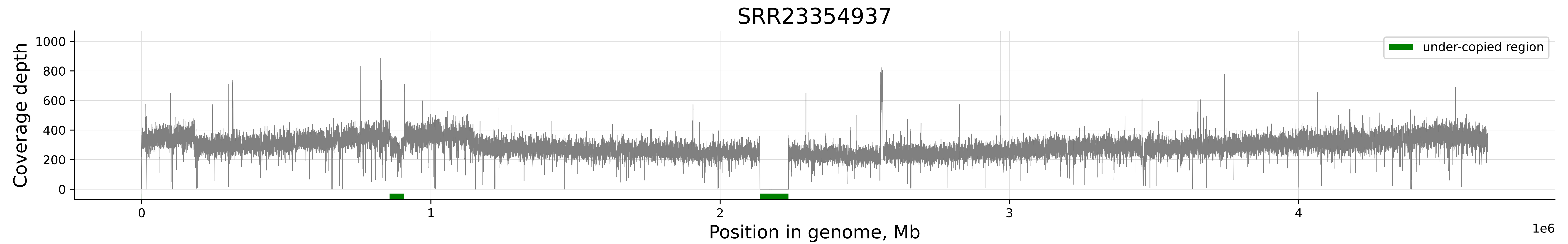

SRR23354937
Coverage depth
0
200
400
600
800
1000
under-copied region
0
1
2
3
4
1e6
Position in genome, Mb

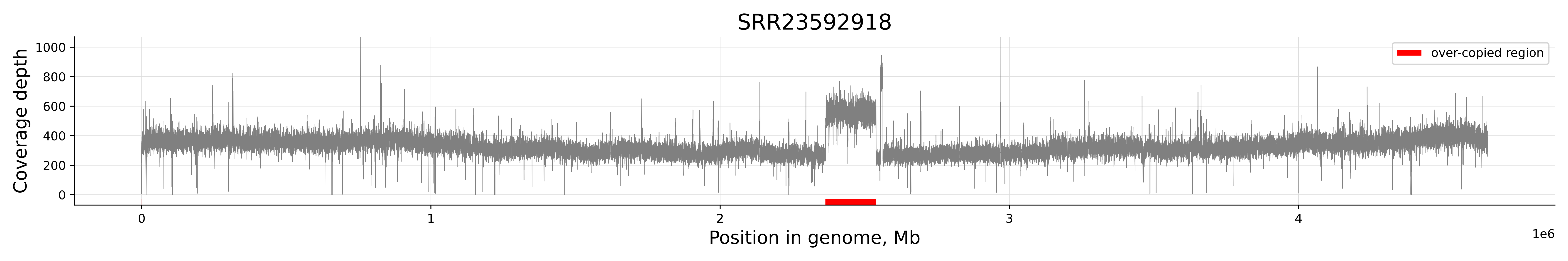

SRR23592918
over-copied region
Coverage depth
0
200
400
600
800
1000
0
1
2
3
4
1e6
Position in genome, Mb

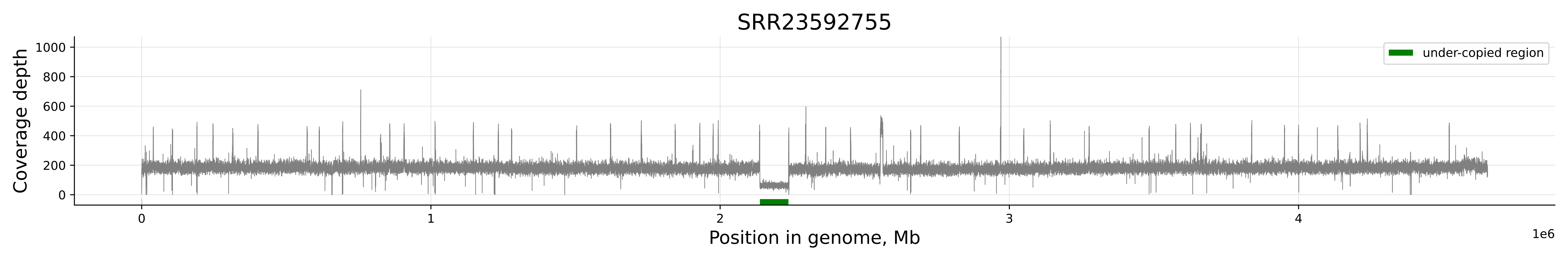

SRR23592755
Coverage depth
1000
800
600
400
200
0
under-copied region
0
1
2
3
4
1e6
Position in genome, Mb

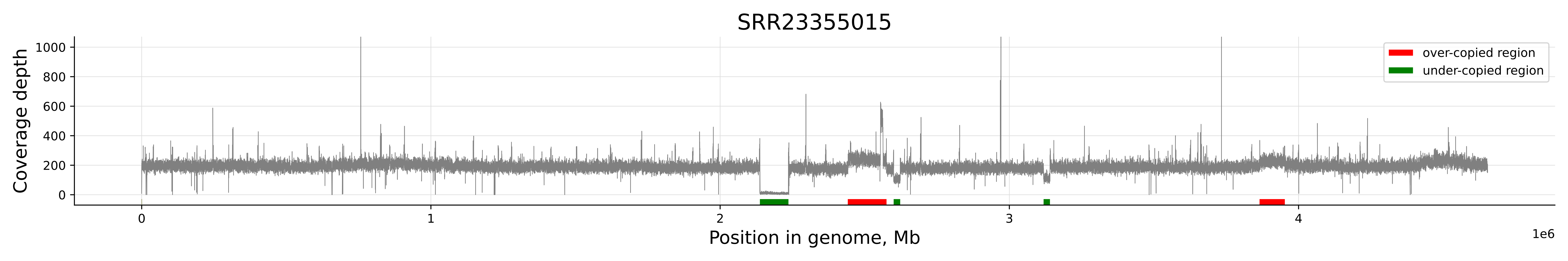
SRR23355015
Coverage depth
0
200
400
600
800
1000
0
1
2
3
4
1e6
Position in genome, Mb
over-copied region
under-copied region

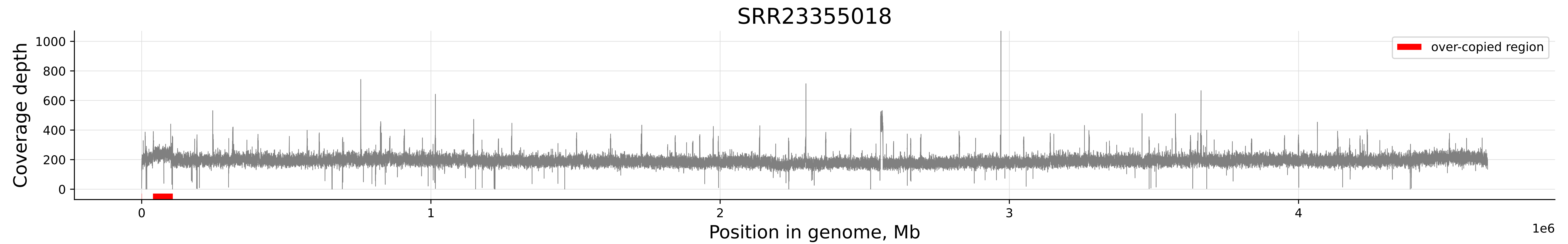

SRR23355018
over-copied region
Coverage depth
0
200
400
600
800
1000
0
1
2
3
4
1e6
Position in genome, Mb

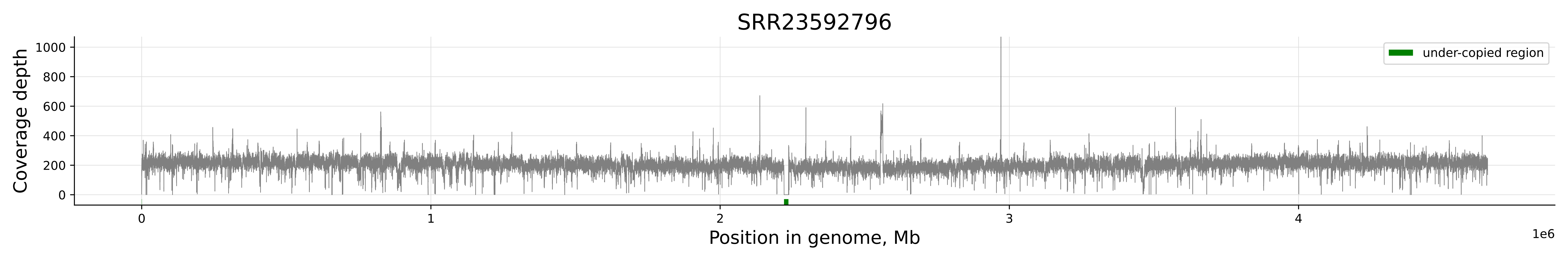

SRR23592796
Coverage depth
1000
800
600
400
200
0
under-copied region
0
1
2
3
4
Position in genome, Mb
1e6

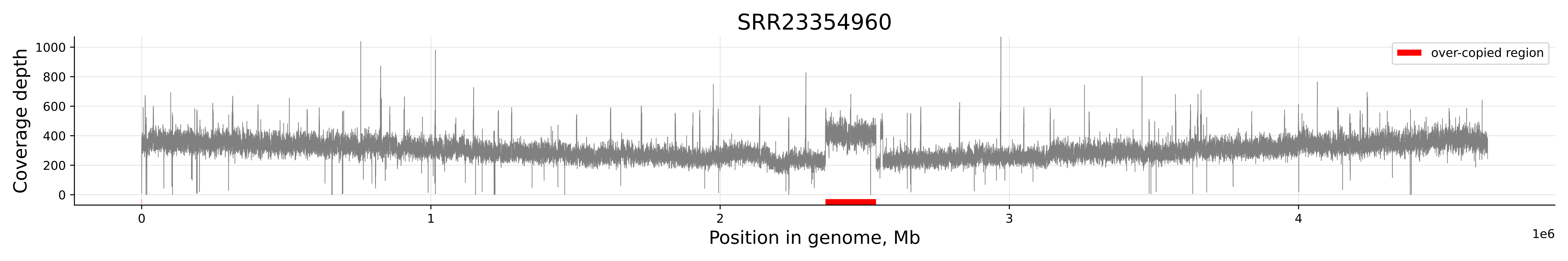

SRR23354960
Coverage depth
Position in genome, Mb
over-copied region
0
200
400
600
800
1000
1
2
3
4
1e6

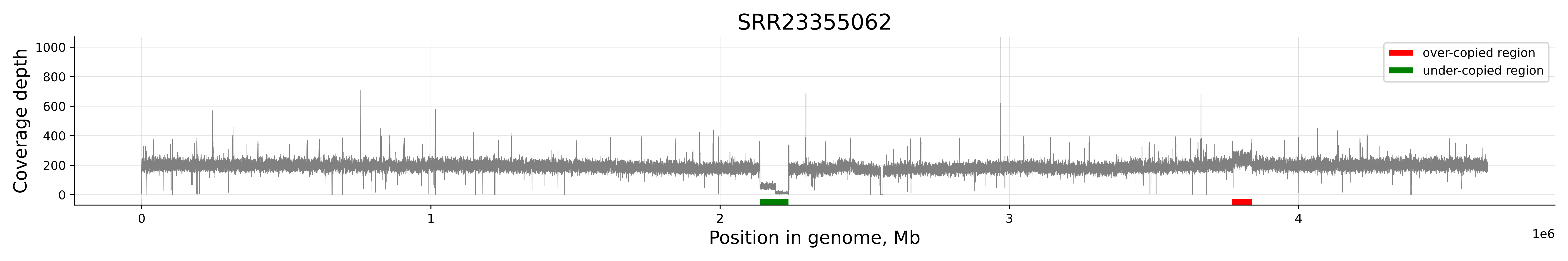

SRR23355062
Coverage depth
1000
800
600
400
200
0
0
1
2
3
4
1e6
Position in genome, Mb
over-copied region
under-copied region

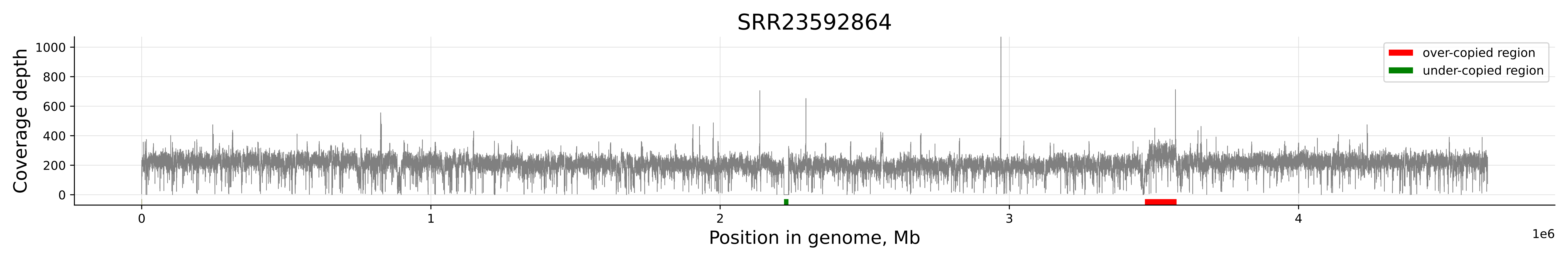
SRR23592864
Coverage depth
1000
800
600
400
200
0
over-copied region
under-copied region
0
1
2
3
4
1e6
Position in genome, Mb

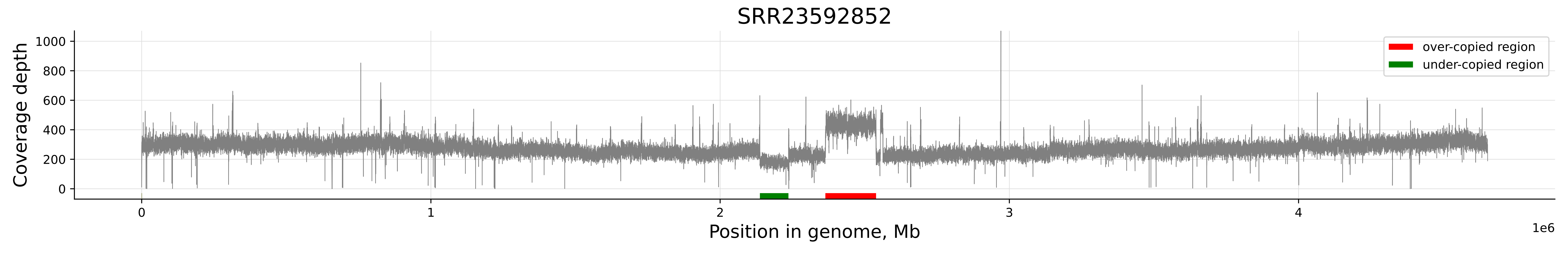

SRR23592852
over-copied region
under-copied region
Coverage depth
Position in genome, Mb
1e6
0
200
400
600
800
1000
0
1
2
3
4

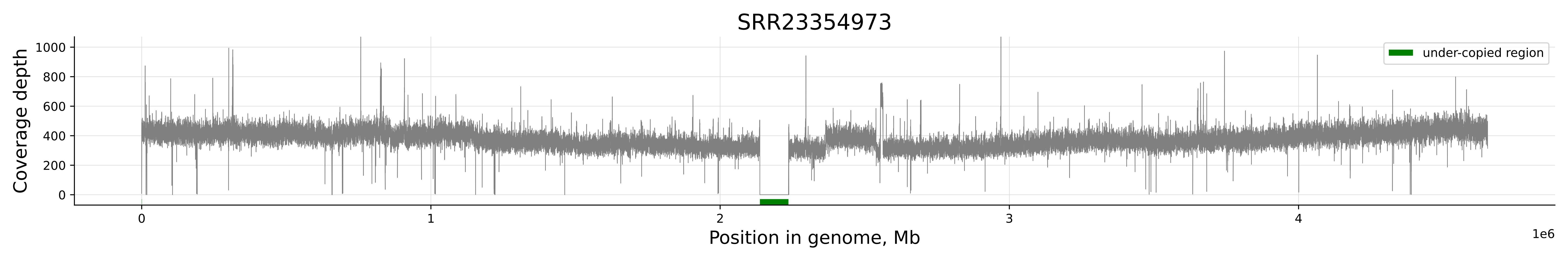
SRR23354973
Coverage depth
1000
800
600
400
200
0
under-copied region
0
1
2
3
4
1e6
Position in genome, Mb

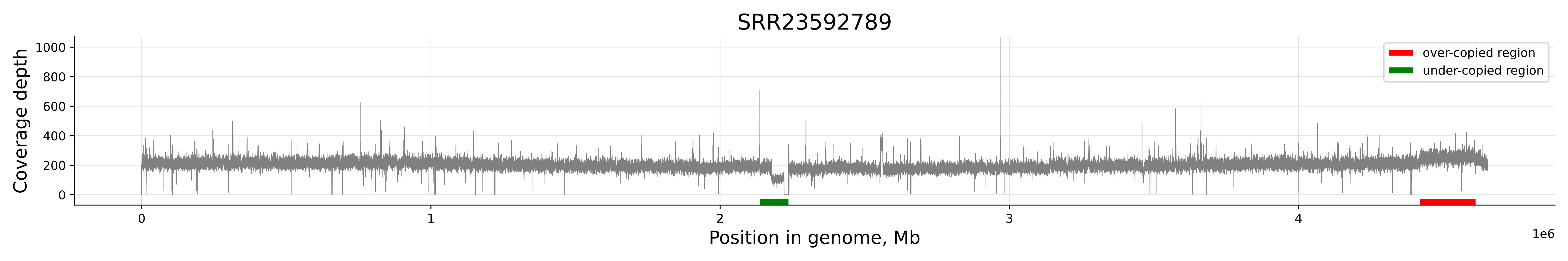

SRR23592789
Coverage depth
0
200
400
600
800
1000
0
1
2
3
4
1e6
Position in genome, Mb
over-copied region
under-copied region

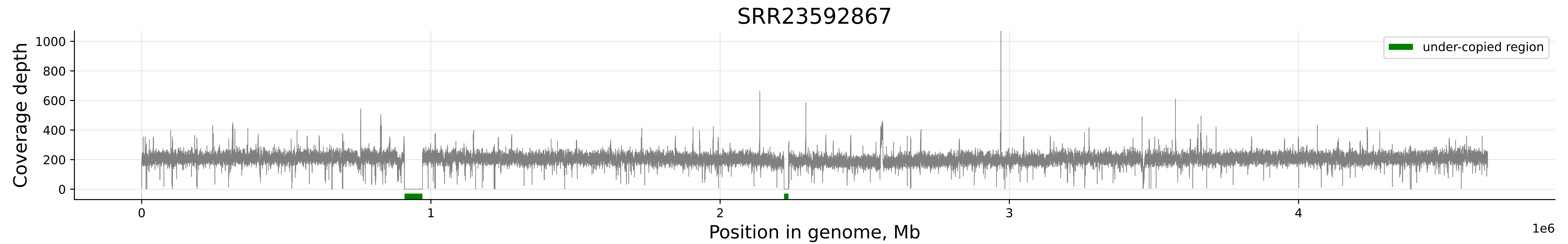

SRR23592867
under-copied region
Coverage depth
0
200
400
600
800
1000
0
1
2
3
4
1e6
Position in genome, Mb

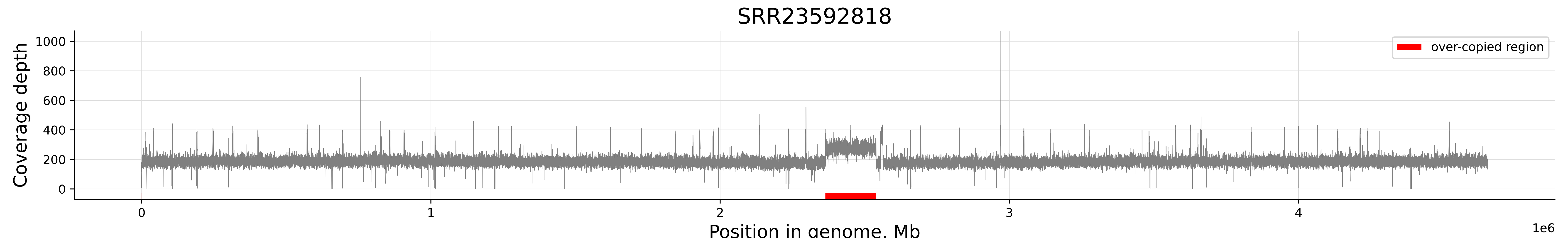

SRR23592818
over-copied region
Coverage depth
1000
800
600
400
200
0
Position in genome, Mb
0
1
2
3
4
1e6

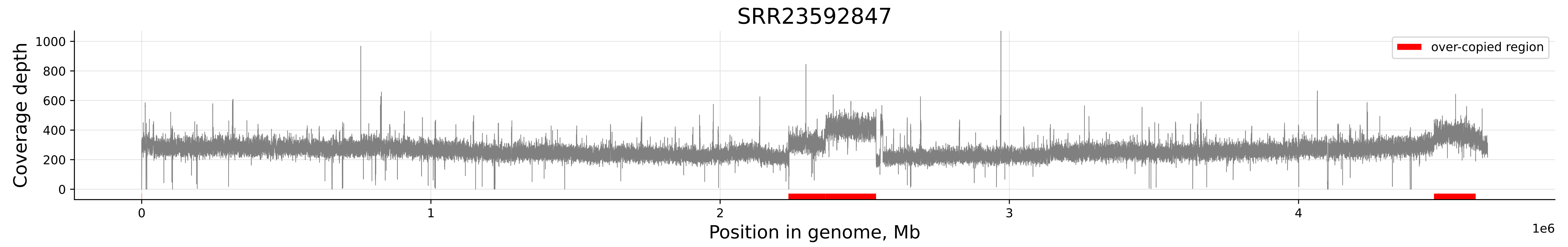

SRR23592847
over-copied region
Coverage depth
0
200
400
600
800
1000
0
1
2
3
4
1e6
Position in genome, Mb

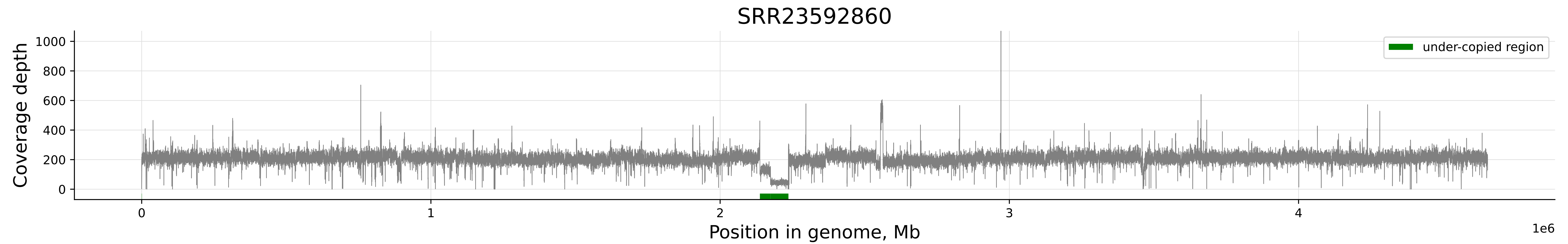

SRR23592860
under-copied region
Coverage depth
0
200
400
600
800
1000
0
1
2
3
4
1e6
Position in genome, Mb

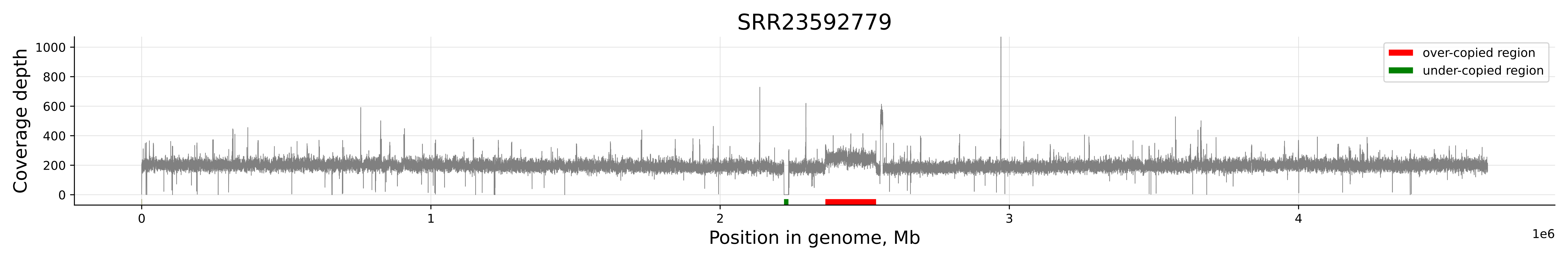

SRR23592779
Coverage depth
1000
800
600
400
200
0
0
1
2
3
4
1e6
Position in genome, Mb
over-copied region
under-copied region

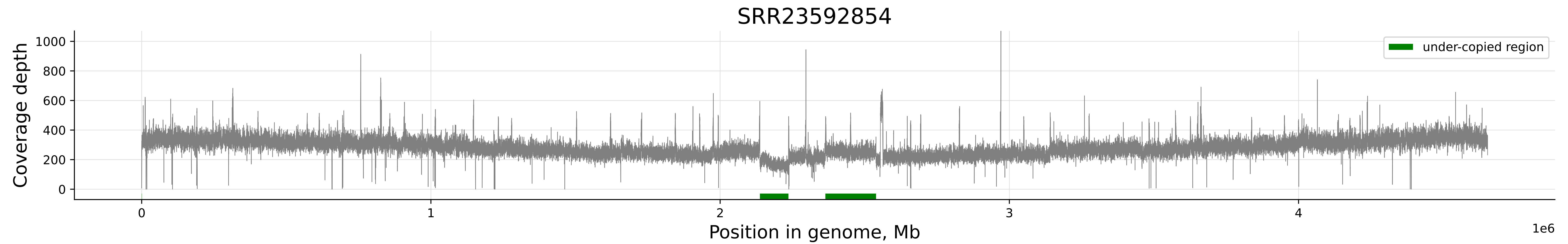

SRR23592854
Coverage depth
Position in genome, Mb
1e6
under-copied region
0
200
400
600
800
1000
1
2
3
4

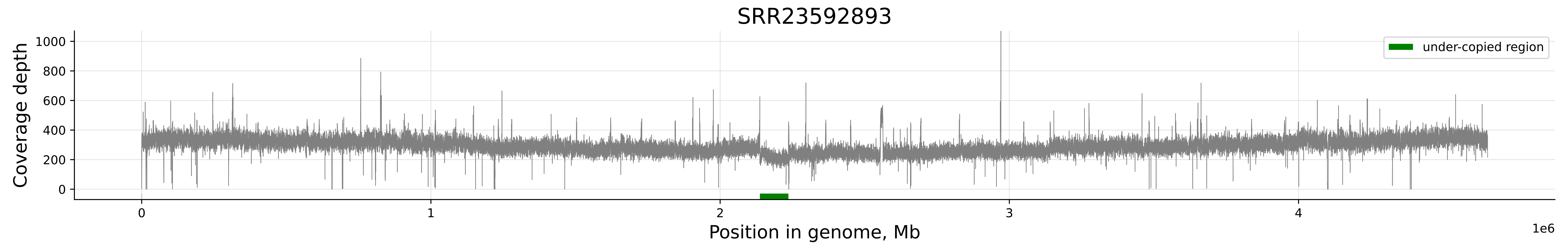
SRR23592893
Coverage depth
Position in genome, Mb
1e6
under-copied region
0
200
400
600
800
1000
0
1
2
3
4

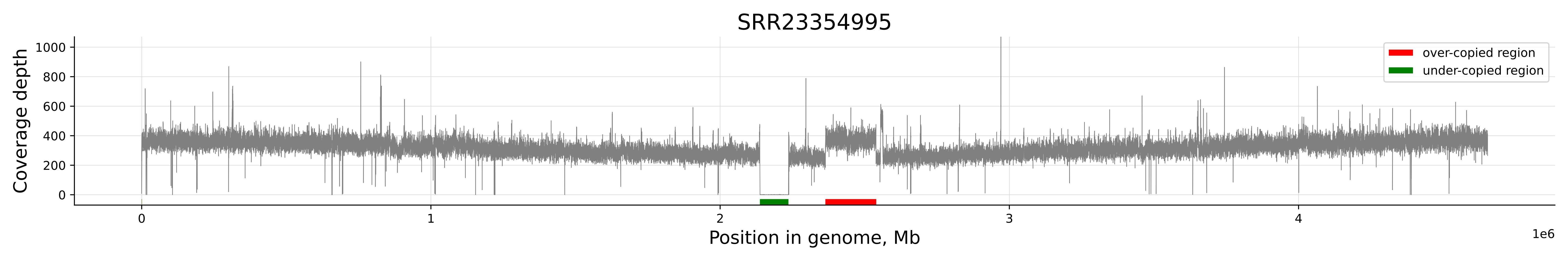

SRR23354995
Coverage depth
0
200
400
600
800
1000
0
1
2
3
4
1e6
Position in genome, Mb
over-copied region
under-copied region

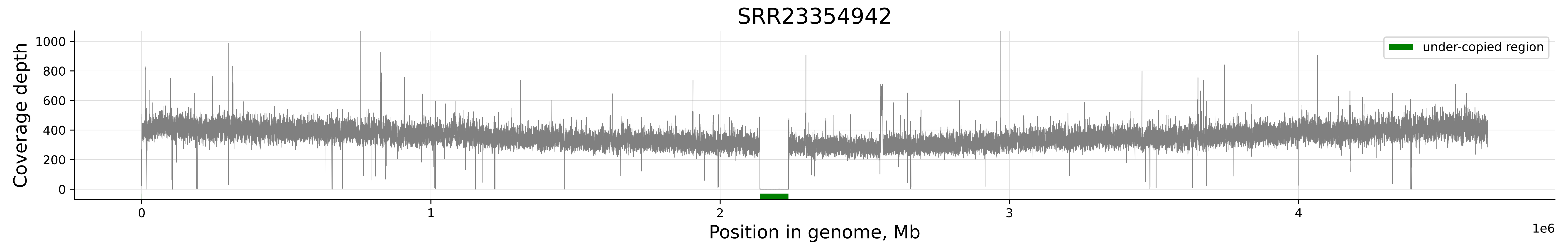
SRR23354942
Coverage depth
Position in genome, Mb
under-copied region
1000
800
600
400
200
0
0
1
2
3
4
1e6

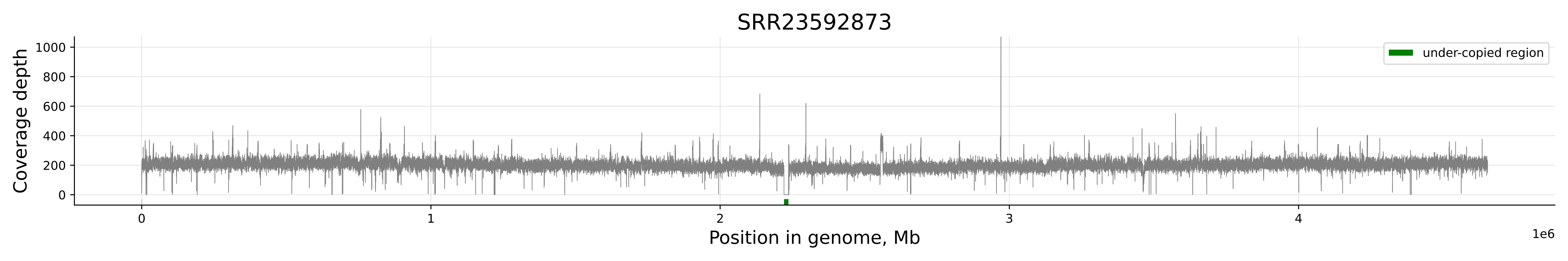

SRR23592873
Coverage depth
1000
800
600
400
200
0
under-copied region
0
1
2
3
4
1e6
Position in genome, Mb

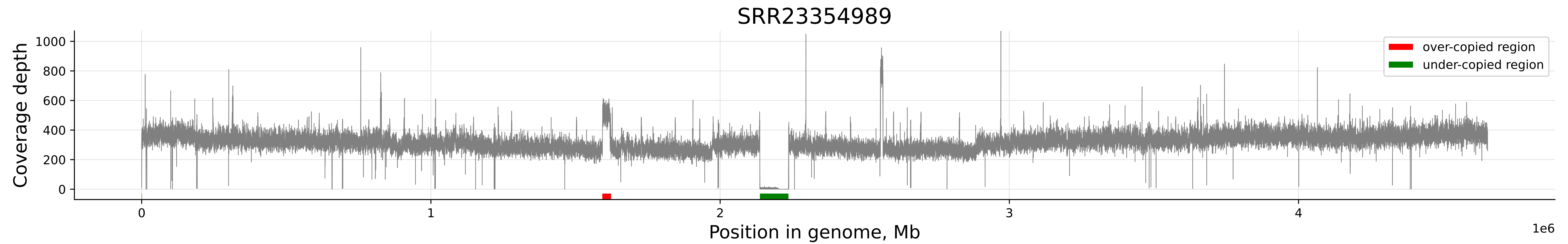

SRR23354989
over-copied region
under-copied region
Coverage depth
0
200
400
600
800
1000
0
1
2
3
4
1e6
Position in genome, Mb

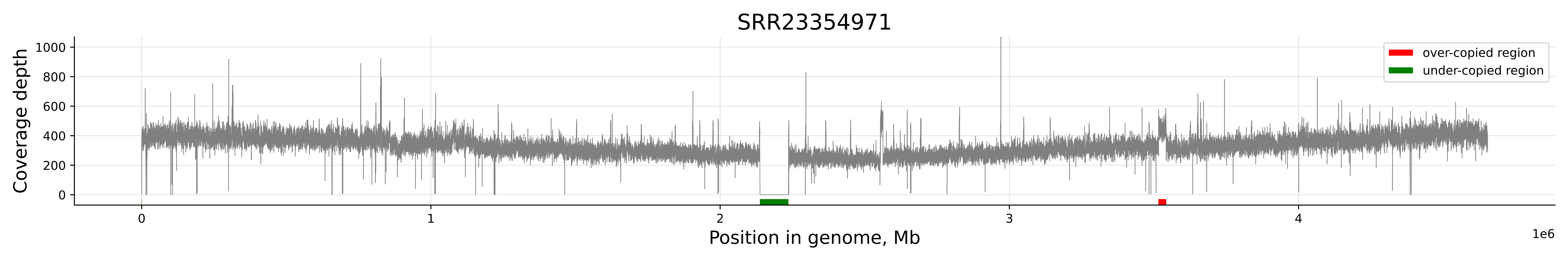

SRR23354971
Coverage depth
0
200
400
600
800
1000
Position in genome, Mb
0
1
2
3
4
1e6
over-copied region
under-copied region

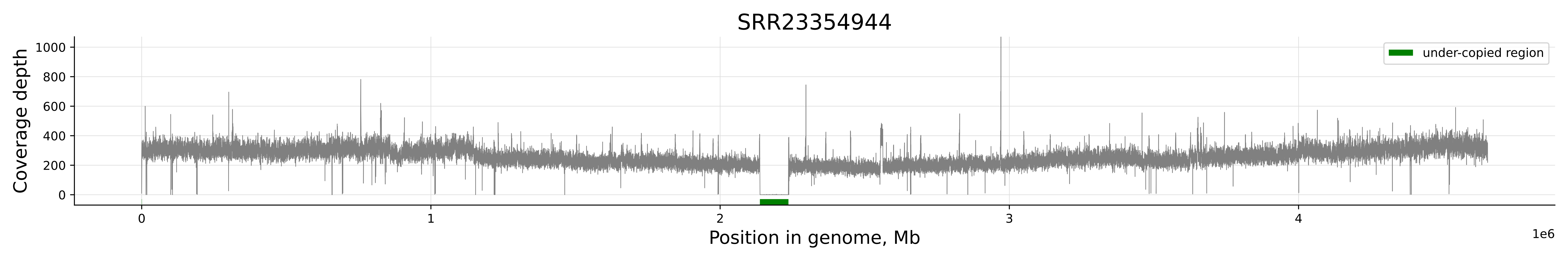
SRR23354944
under-copied region
Coverage depth
0
200
400
600
800
1000
0
1
2
3
4
Position in genome, Mb
1e6

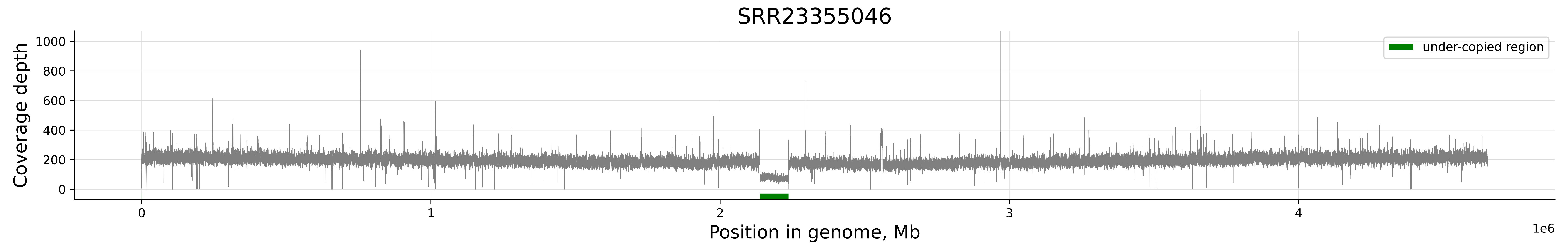

SRR23355046
under-copied region
Coverage depth
1000
800
600
400
200
0
0
1
2
3
4
1e6
Position in genome, Mb

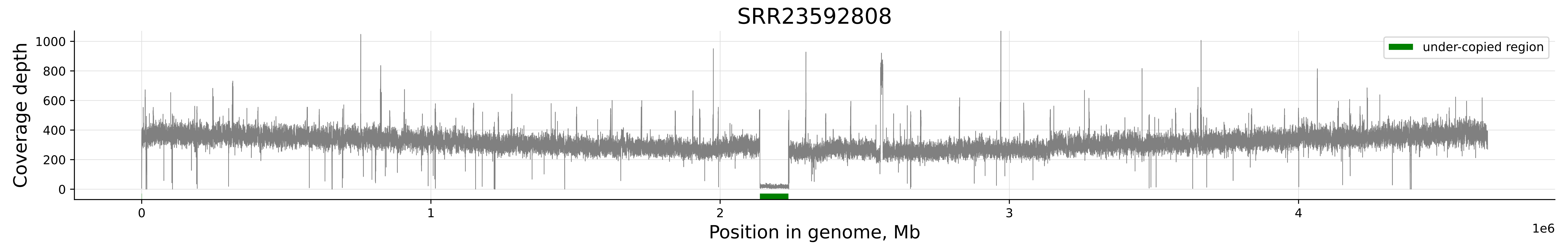

SRR23592808
Coverage depth
Position in genome, Mb
1e6
under-copied region
0
200
400
600
800
1000
0
1
2
3
4

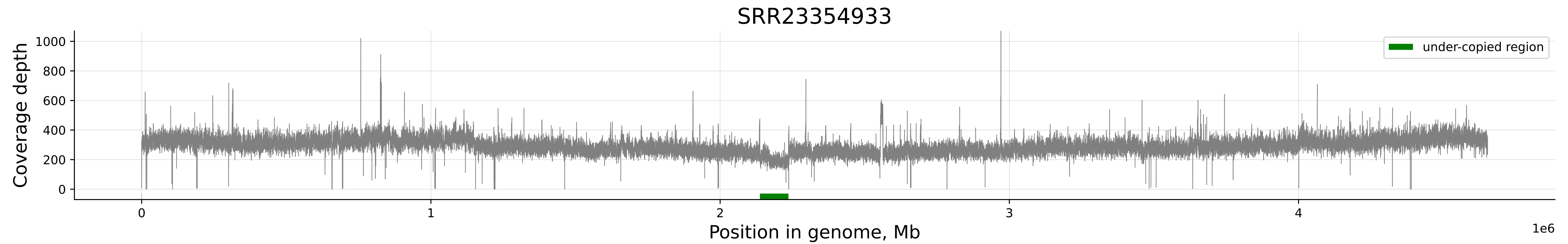
SRR23354933
Coverage depth
1000
800
600
400
200
0
under-copied region
0
1
2
3
4
1e6
Position in genome, Mb

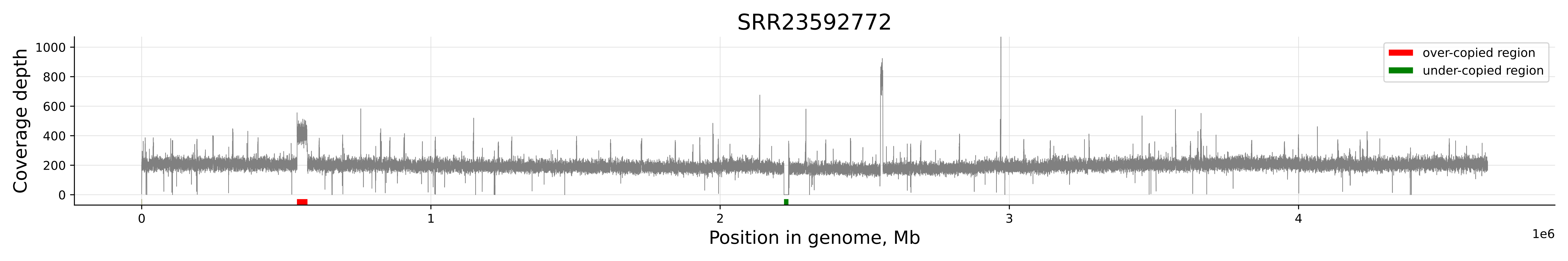

SRR23592772
Coverage depth
Position in genome, Mb
1e6
over-copied region
under-copied region
0
200
400
600
800
1000
0
1
2
3
4

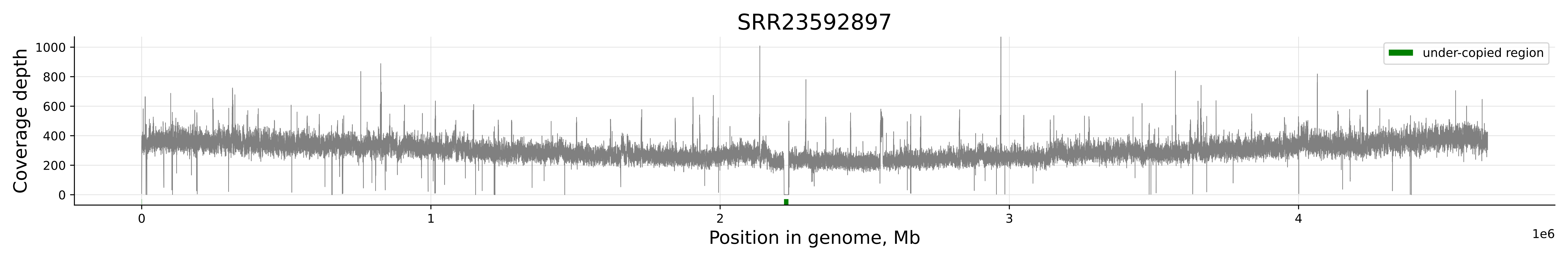

SRR23592897
under-copied region
Coverage depth
0
200
400
600
800
1000
Position in genome, Mb
0
1
2
3
4
1e6

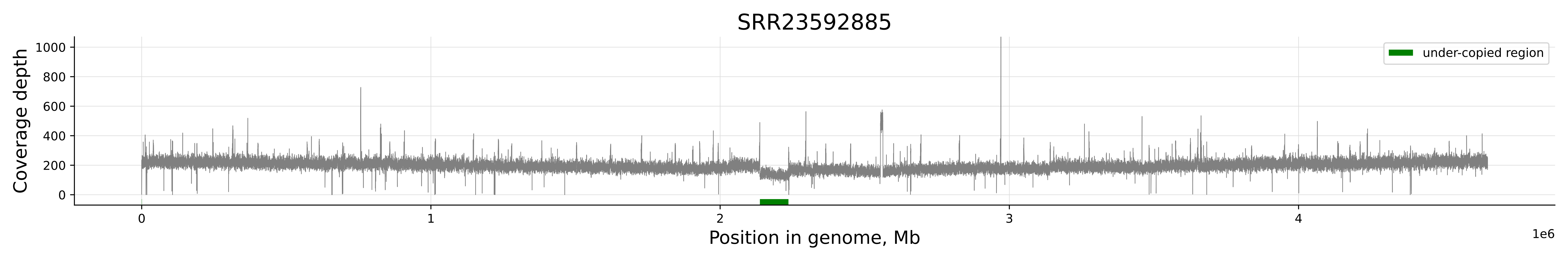
SRR23592885
under-copied region
Coverage depth
Position in genome, Mb
1e6
0
200
400
600
800
1000
0
1
2
3
4

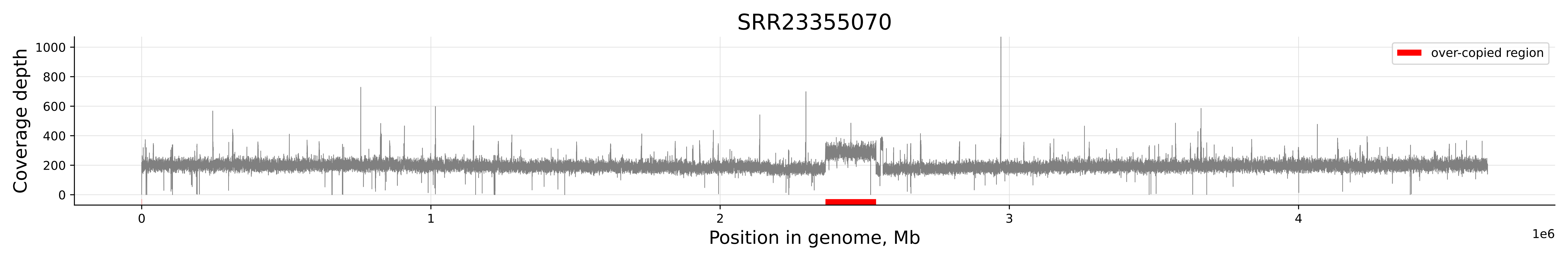
SRR23355070
Coverage depth
1000
800
600
400
200
0
over-copied region
0
1
2
3
4
1e6
Position in genome, Mb

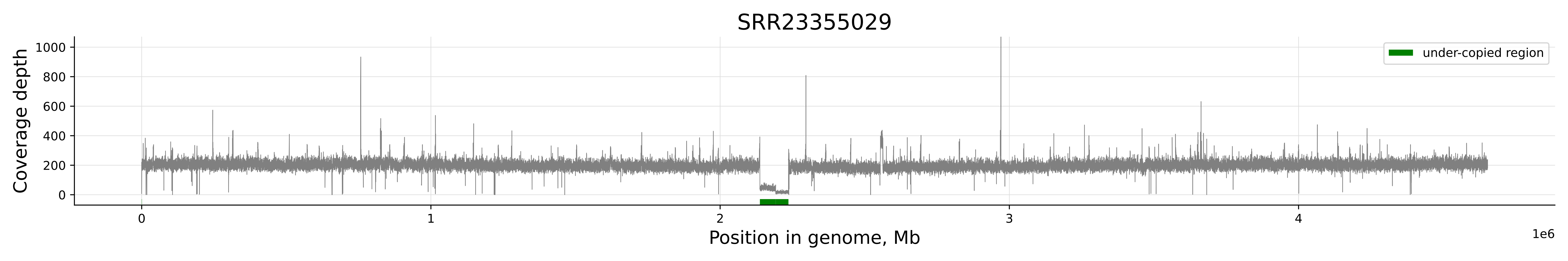
SRR23355029
Coverage depth
Position in genome, Mb
1e6
under-copied region
0
200
400
600
800
1000
0
1
2
3
4

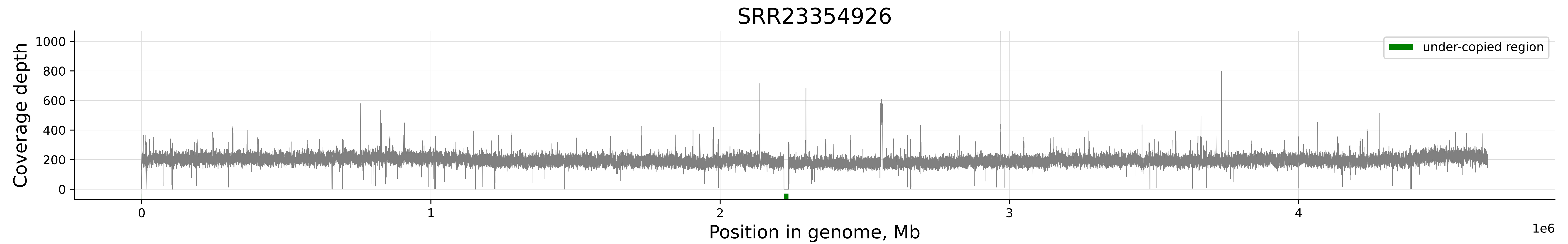
SRR23354926
Coverage depth
Position in genome, Mb
1e6
under-copied region
0
200
400
600
800
1000
0
1
2
3
4

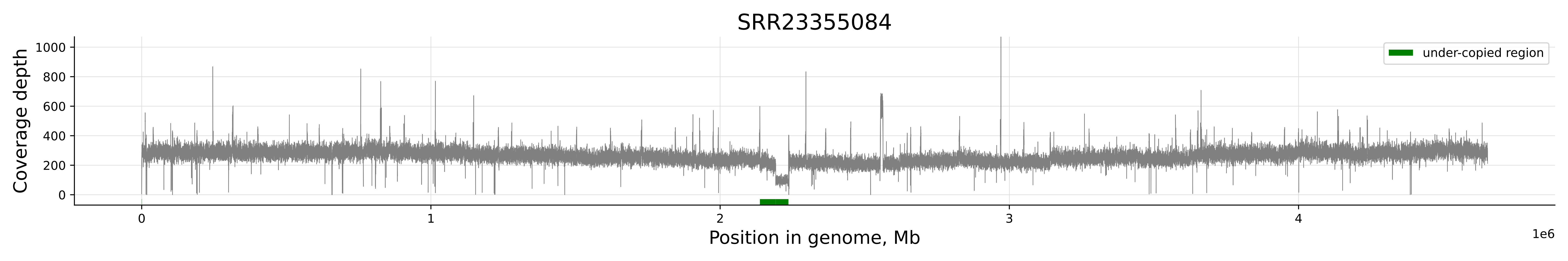
SRR23355084
under-copied region
Coverage depth
0
200
400
600
800
1000
Position in genome, Mb
0
1
2
3
4
1e6

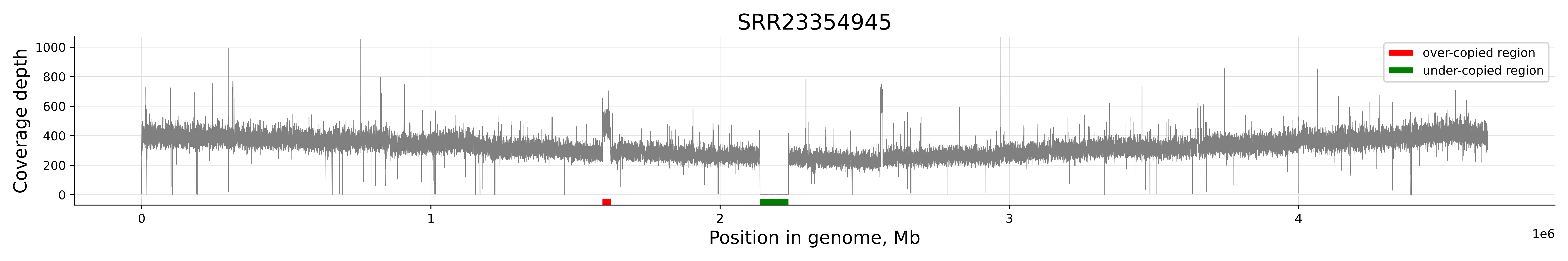

SRR23354945
Coverage depth
0
200
400
600
800
1000
over-copied region
under-copied region
0
1
2
3
4
1e6
Position in genome, Mb

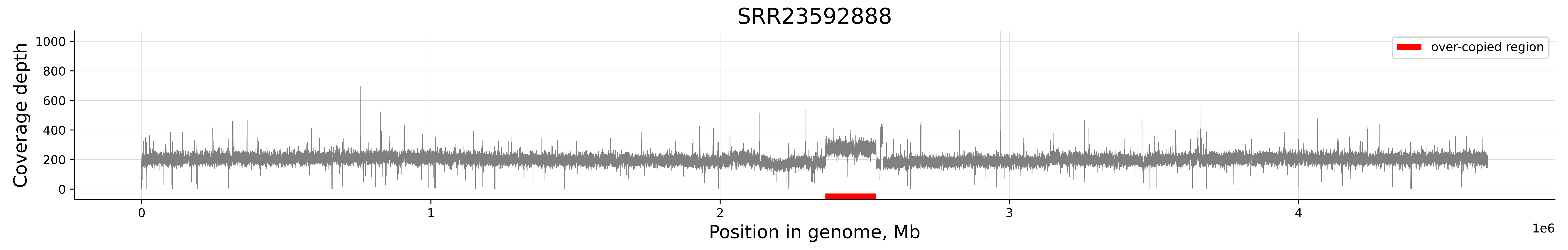

SRR23592888
Coverage depth
Position in genome, Mb
1e6
over-copied region
0
200
400
600
800
1000
0
1
2
3
4

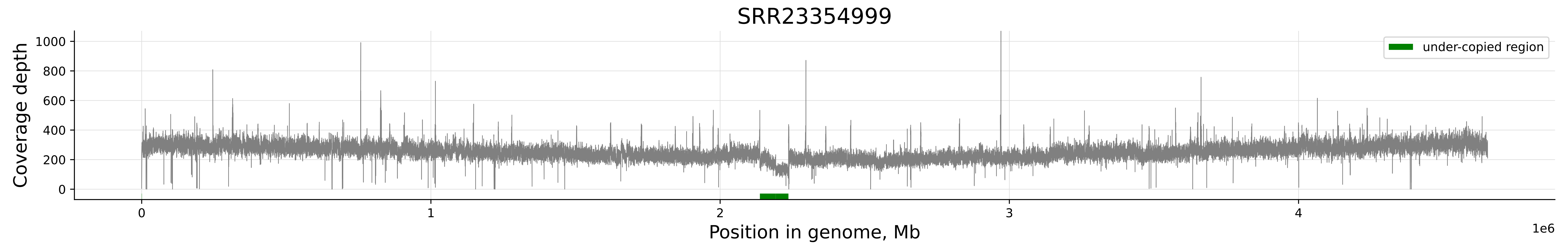
SRR23354999
Coverage depth
1000
800
600
400
200
0
under-copied region
0
1
2
3
4
1e6
Position in genome, Mb

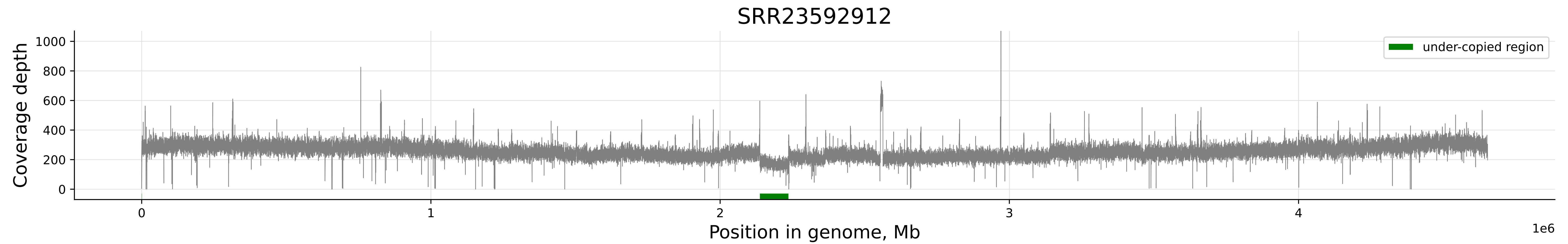

SRR23592912
Coverage depth
1000
800
600
400
200
0
Position in genome, Mb
1e6
under-copied region

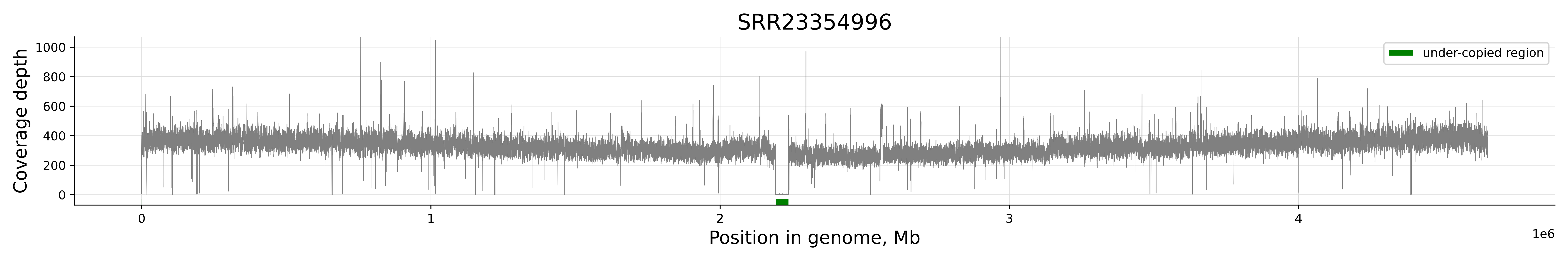
SRR23354996
Coverage depth
1000
800
600
400
200
0
0
1
2
3
4
1e6
Position in genome, Mb
under-copied region

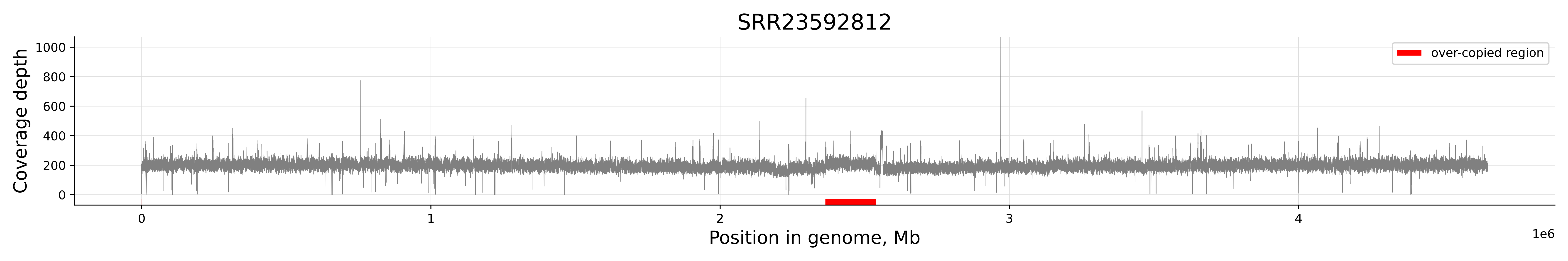

SRR23592812
Coverage depth
Position in genome, Mb
1e6
over-copied region
0
200
400
600
800
1000
1
2
3
4

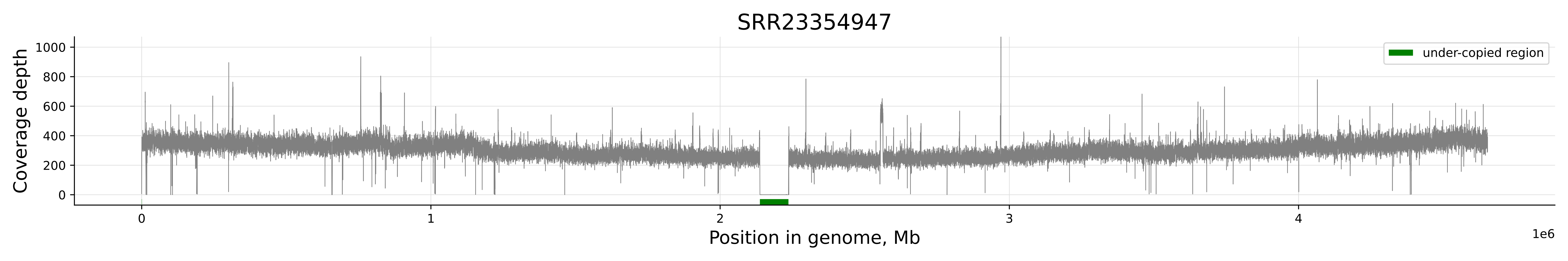
SRR23354947
Coverage depth
Position in genome, Mb
under-copied region
1e6
0
1
2
3
4
0
200
400
600
800
1000

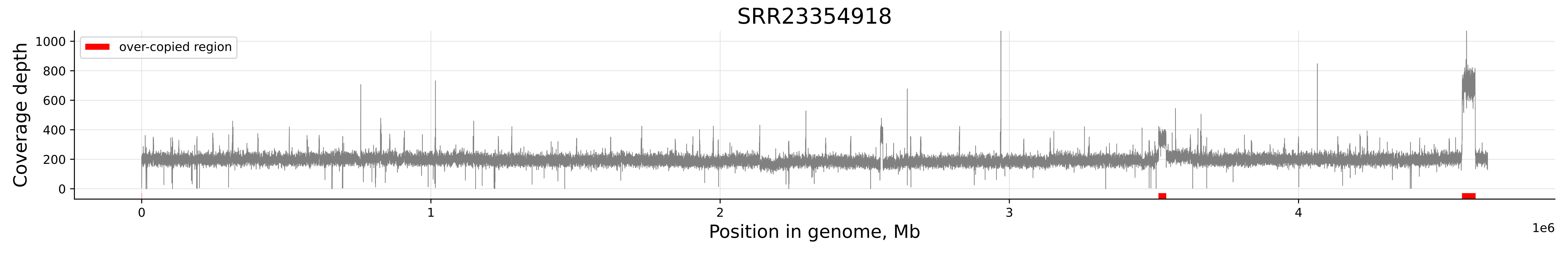

SRR23354918
over-copied region
Coverage depth
0
200
400
600
800
1000
0
1
2
3
4
Position in genome, Mb
1e6

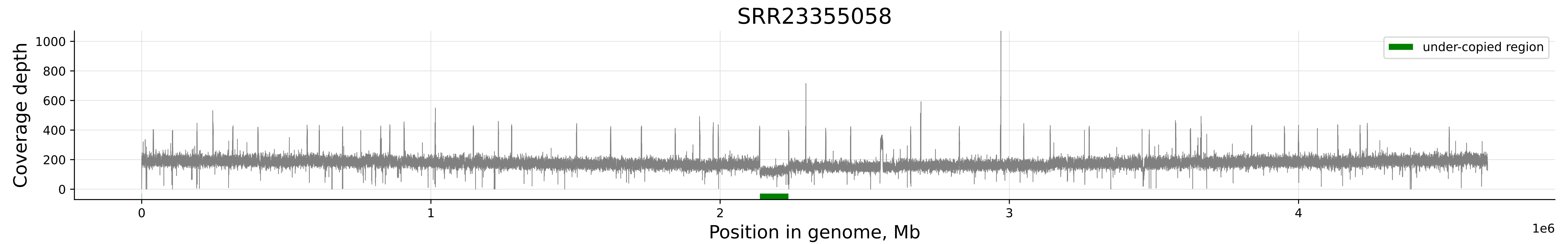
SRR23355058
Coverage depth
1000
800
600
400
200
0
under-copied region
0
1
2
3
4
1e6
Position in genome, Mb

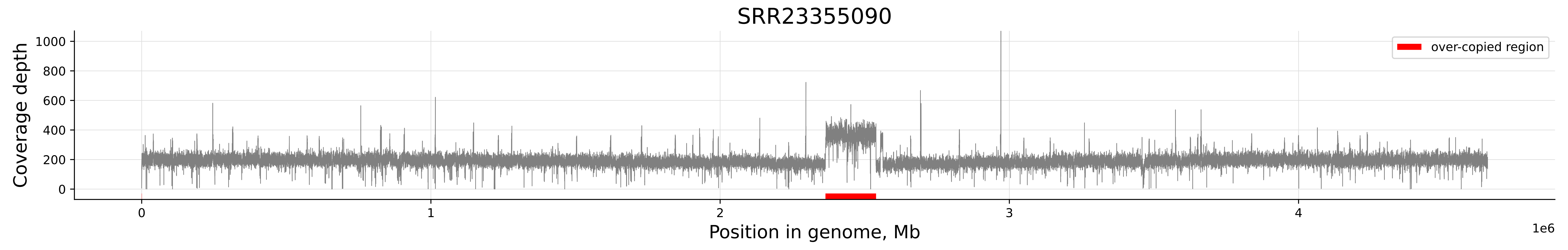
SRR23355090
Coverage depth
Position in genome, Mb
1e6
over-copied region
0
200
400
600
800
1000
0
1
2
3
4

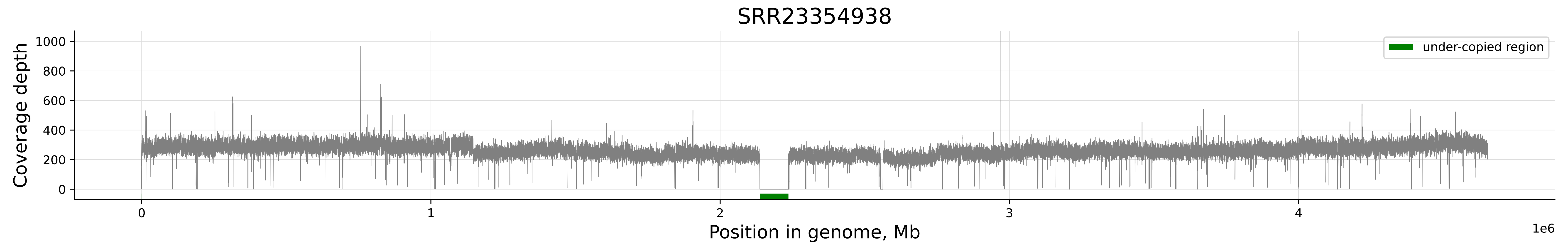
SRR23354938
under-copied region
Coverage depth
0
200
400
600
800
1000
Position in genome, Mb
0
1
2
3
4
1e6

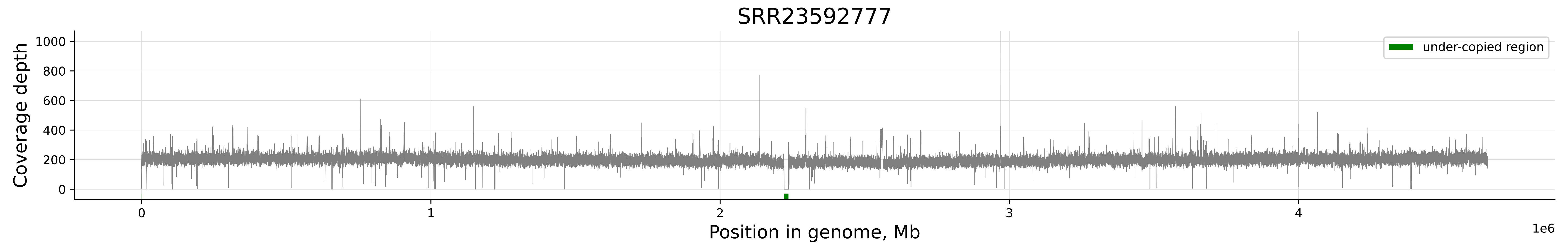

SRR23592777
Coverage depth
Position in genome, Mb
under-copied region
1e6

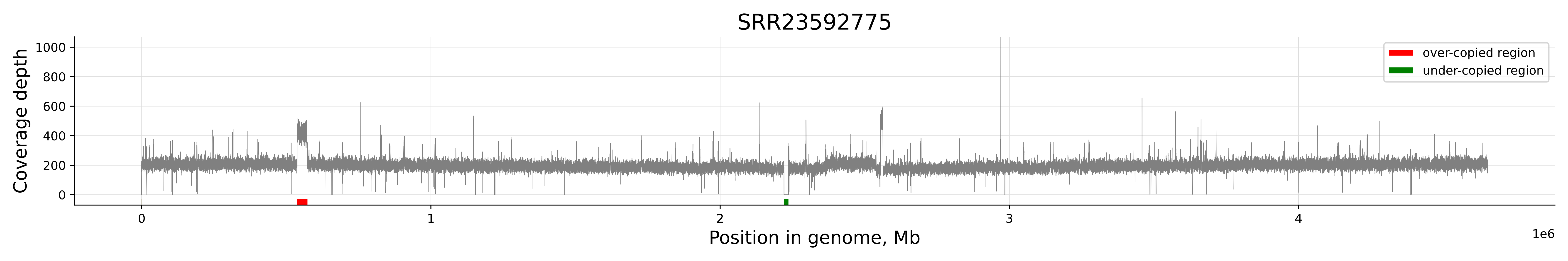

SRR23592775
Coverage depth
Position in genome, Mb
1e6
over-copied region
under-copied region
0
200
400
600
800
1000
0
1
2
3
4

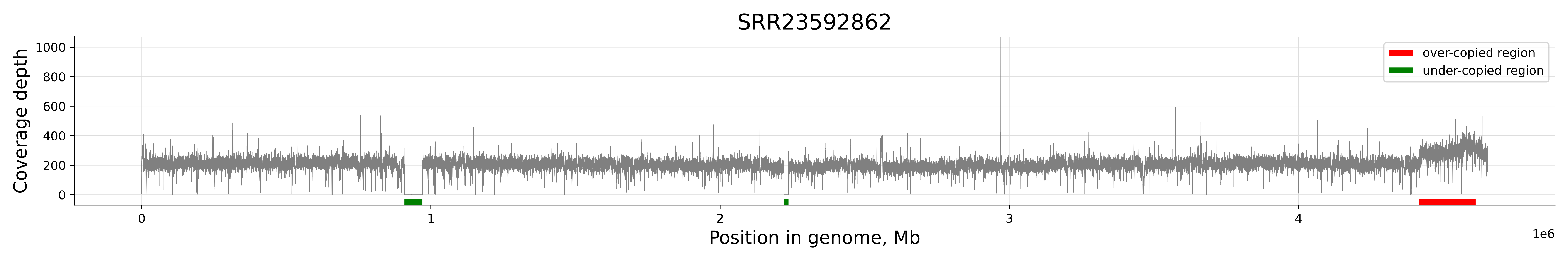
SRR23592862
Coverage depth
Position in genome, Mb
1e6
over-copied region
under-copied region
0
200
400
600
800
1000
0
1
2
3
4

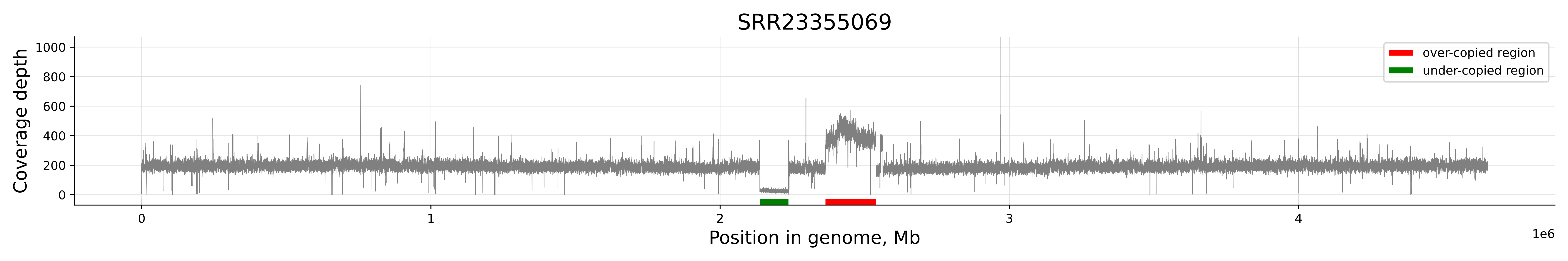
SRR23355069
Coverage depth
0
200
400
600
800
1000
over-copied region
under-copied region
0
1
2
3
4
1e6
Position in genome, Mb

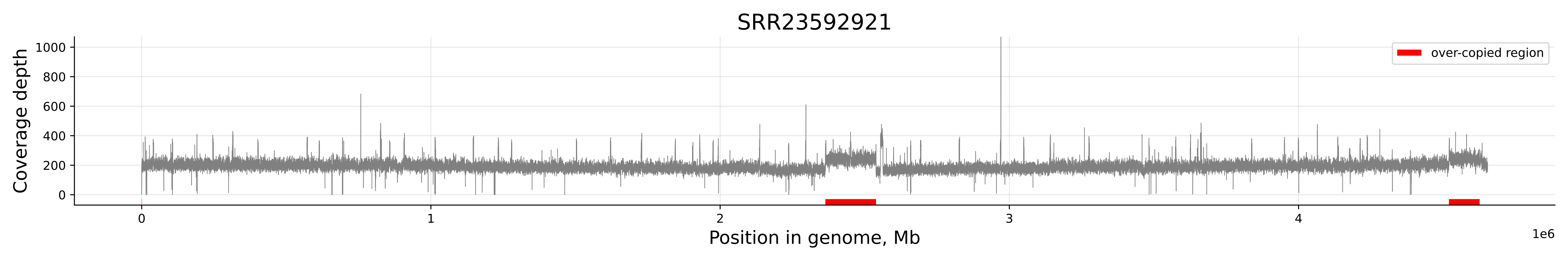

SRR23592921
Coverage depth
1000
800
600
400
200
0
over-copied region
0
1
2
3
4
1e6
Position in genome, Mb

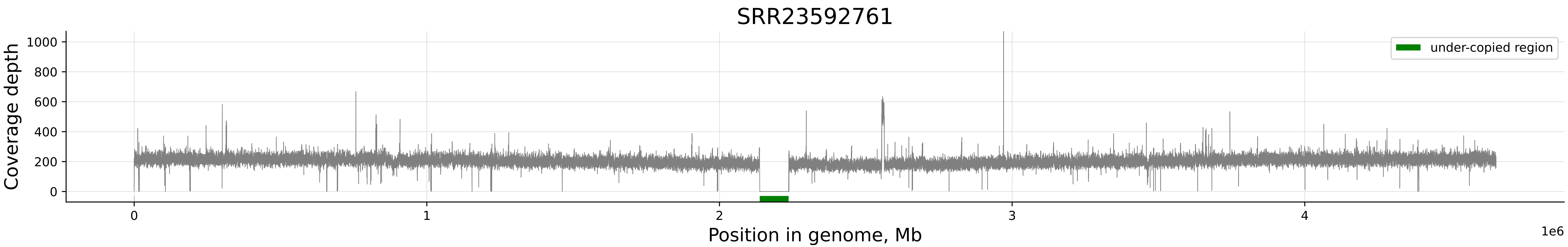
SRR23592761
under-copied region
Coverage depth
0
200
400
600
800
1000
0
1
2
3
4
1e6
Position in genome, Mb

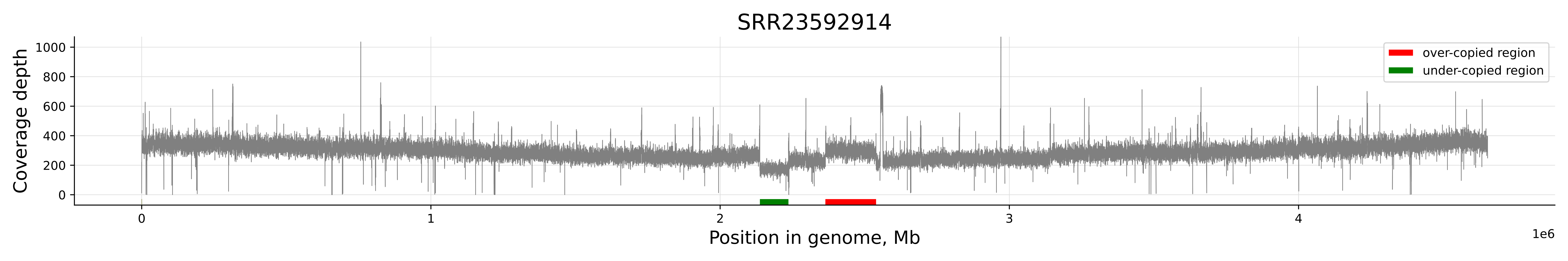
SRR23592914
Coverage depth
Position in genome, Mb
1e6
over-copied region
under-copied region
0
200
400
600
800
1000
0
1
2
3
4

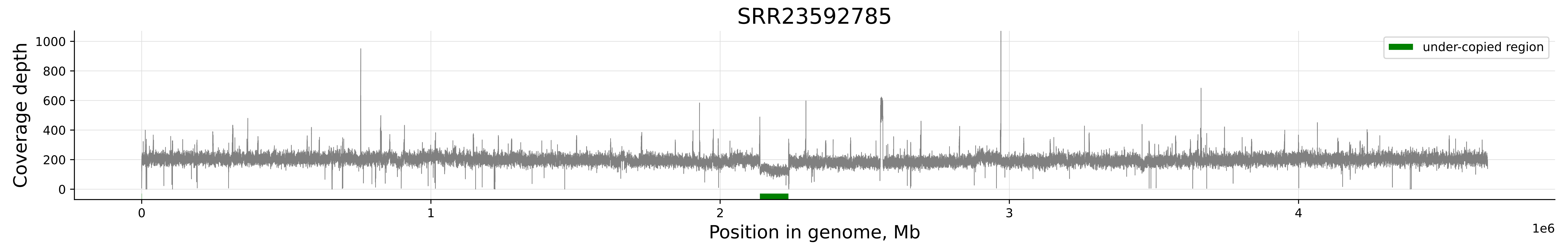

SRR23592785
Coverage depth
Position in genome, Mb
1e6
under-copied region
0
200
400
600
800
1000
0
1
2
3
4

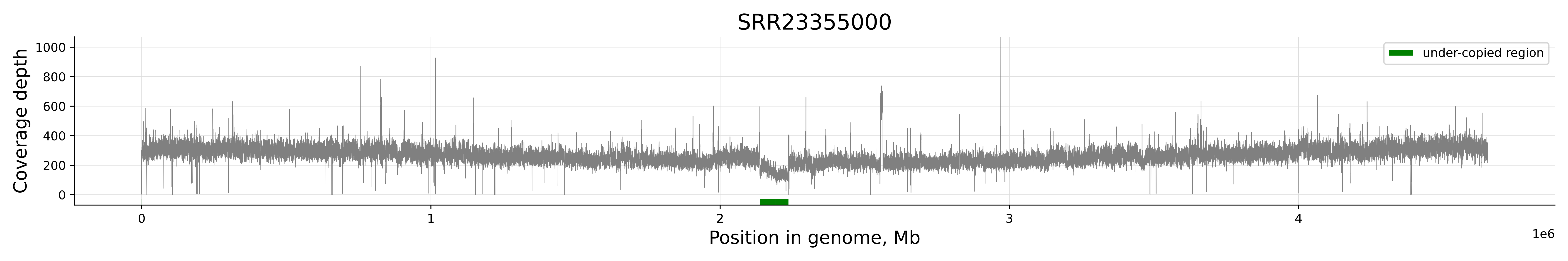

SRR23355000
under-copied region
Coverage depth
0
200
400
600
800
1000
0
1
2
3
4
1e6
Position in genome, Mb

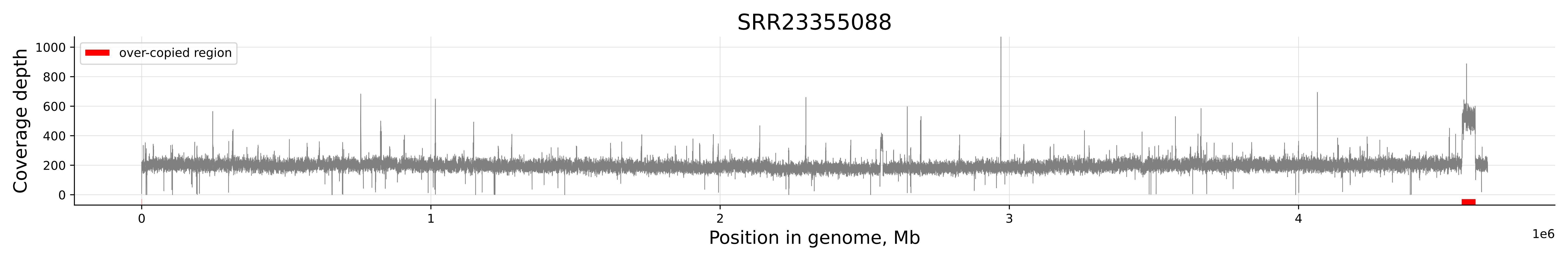

SRR23355088
over-copied region
Coverage depth
0
200
400
600
800
1000
0
1
2
3
4
1e6
Position in genome, Mb

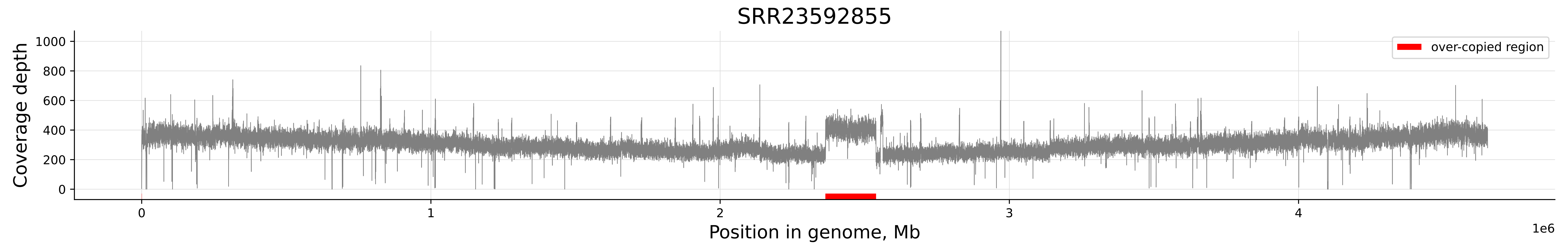
SRR23592855
over-copied region
Coverage depth
0
200
400
600
800
1000
0
1
2
3
4
1e6
Position in genome, Mb

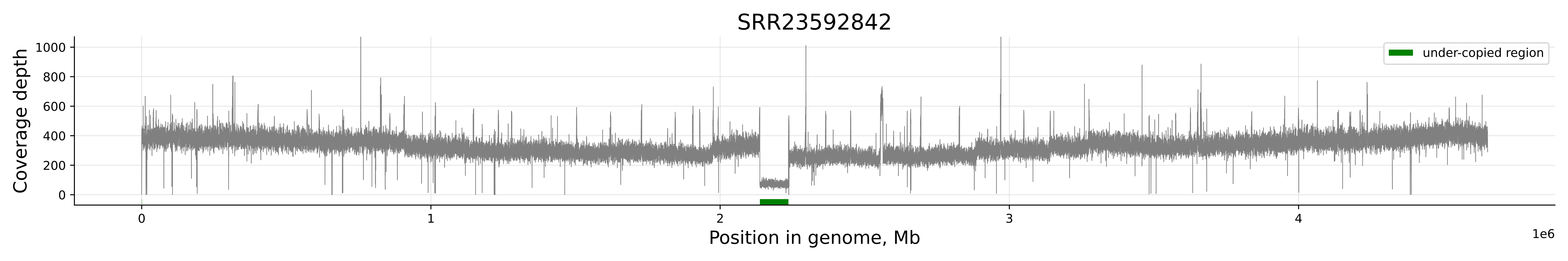

SRR23592842
Coverage depth
Position in genome, Mb
under-copied region
0
200
400
600
800
1000
0
1
2
3
4
1e6

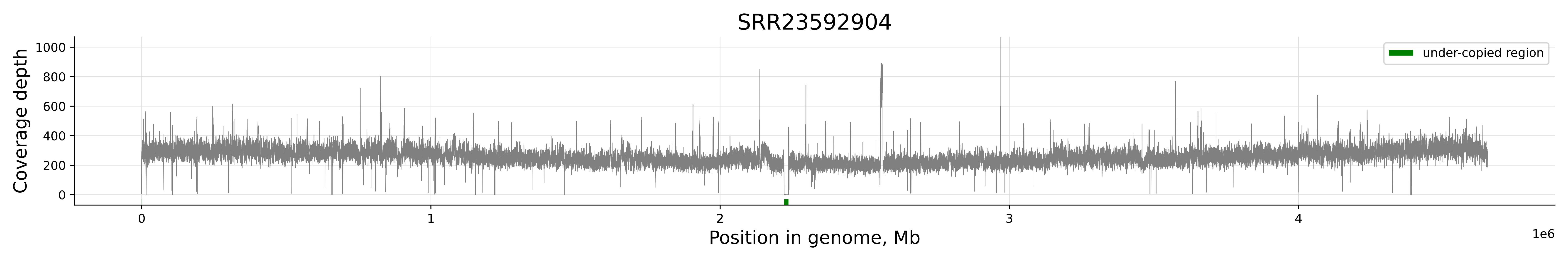

SRR23592904
Coverage depth
Position in genome, Mb
1e6
under-copied region
0
200
400
600
800
1000
0
1
2
3
4

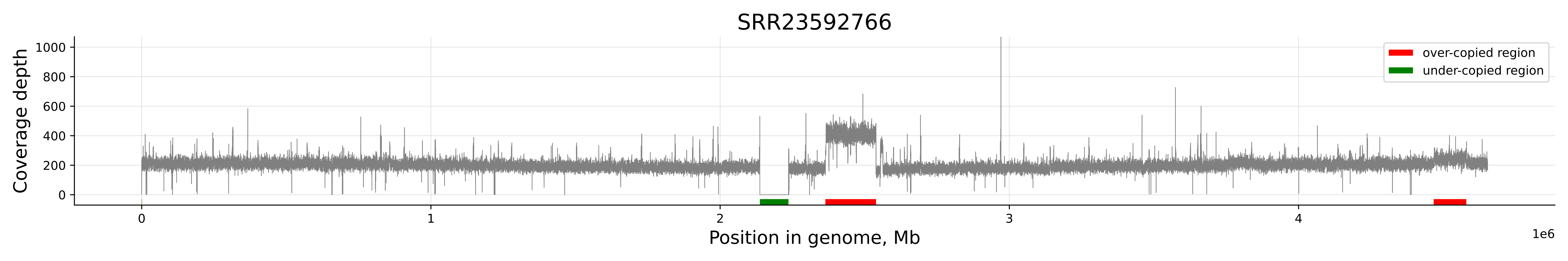

SRR23592766
Coverage depth
1000
800
600
400
200
0
0
1
2
3
4
1e6
Position in genome, Mb
over-copied region
under-copied region

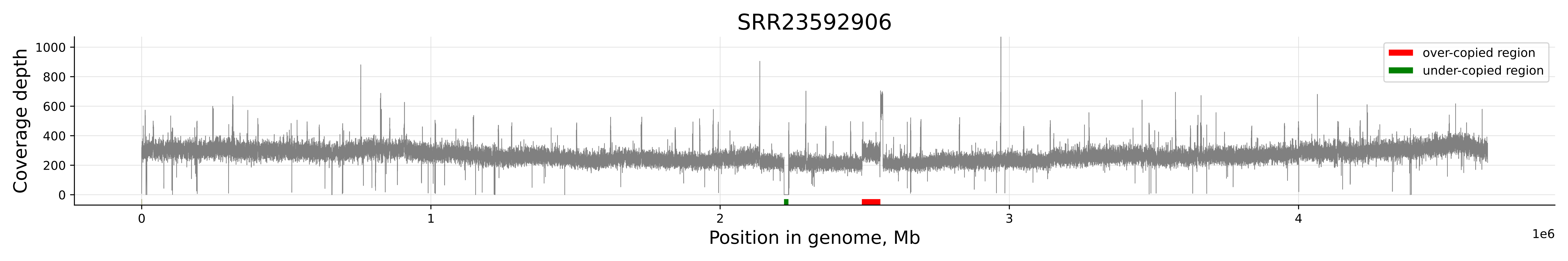

SRR23592906
Coverage depth
Position in genome, Mb
1e6
over-copied region
under-copied region
0
200
400
600
800
1000
0
1
2
3
4

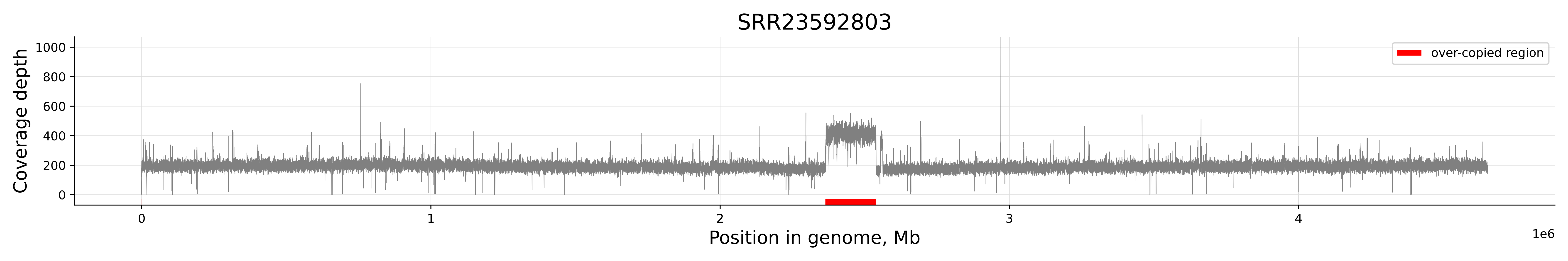

SRR23592803
Coverage depth
Position in genome, Mb
over-copied region
1e6
0
200
400
600
800
1000
1
2
3
4

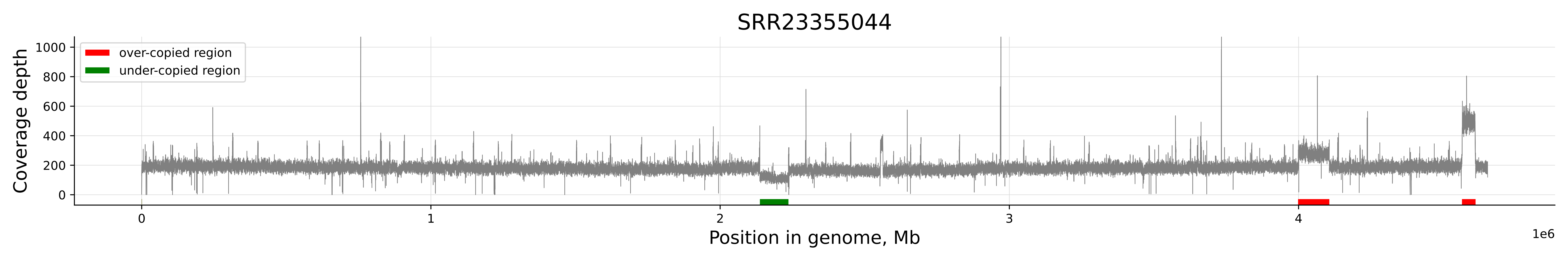
SRR23355044
over-copied region
under-copied region
Coverage depth
0
200
400
600
800
1000
0
1
2
3
4
1e6
Position in genome, Mb

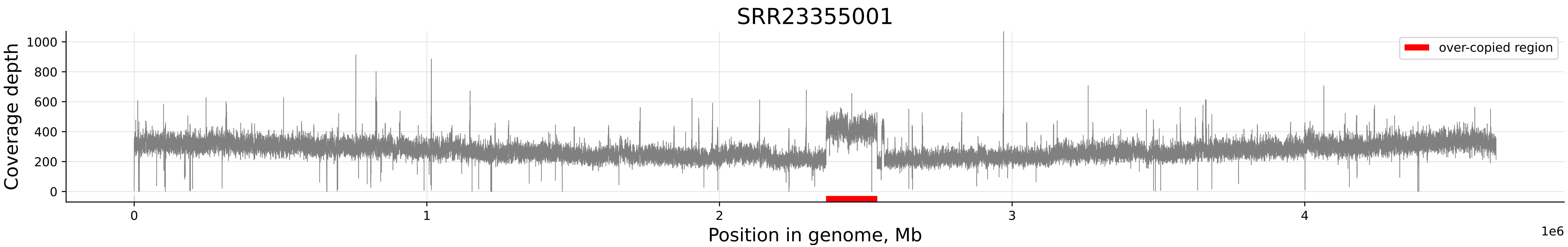

SRR23355001
Coverage depth
Position in genome, Mb
1e6
over-copied region
0
200
400
600
800
1000
0
1
2
3
4

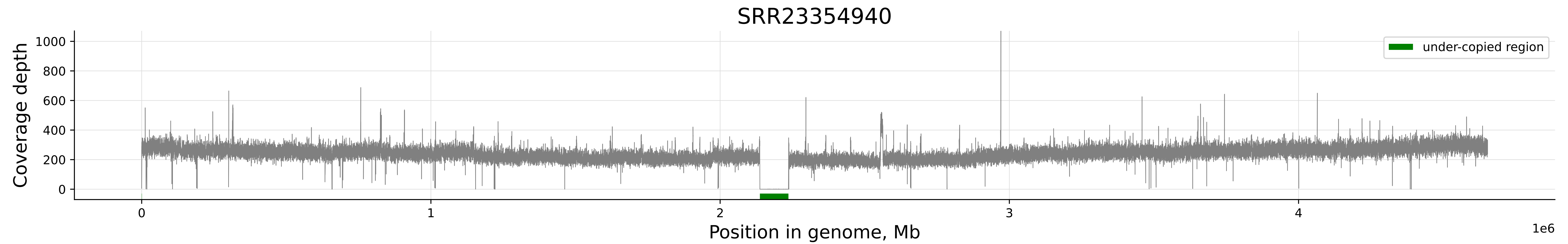
SRR23354940
under-copied region
Coverage depth
Position in genome, Mb
1e6
0
200
400
600
800
1000
0
1
2
3
4

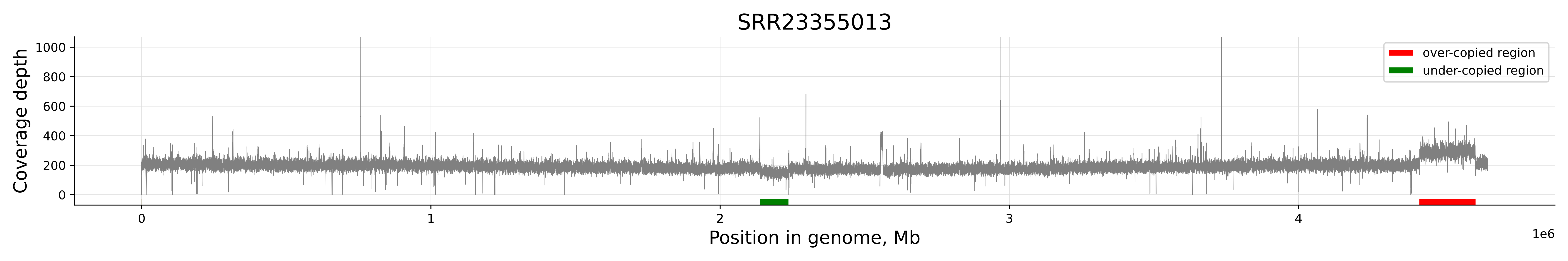
SRR23355013
Coverage depth
0
200
400
600
800
1000
Position in genome, Mb
0
1
2
3
4
1e6
over-copied region
under-copied region

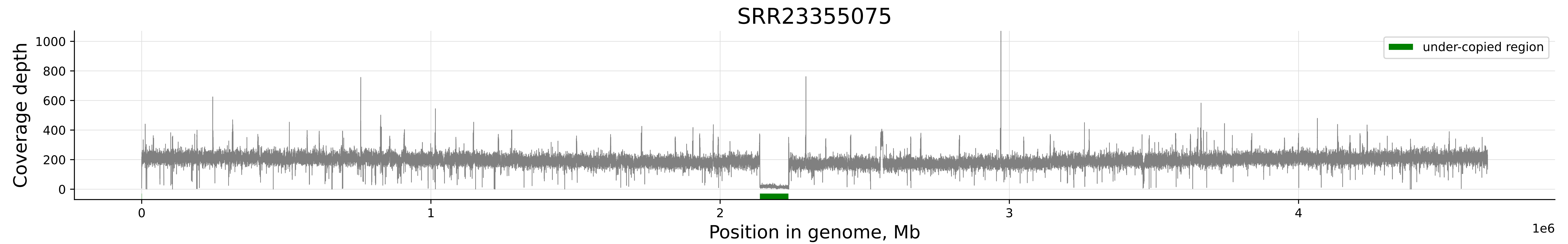

SRR23355075
Coverage depth
Position in genome, Mb
under-copied region
1e6

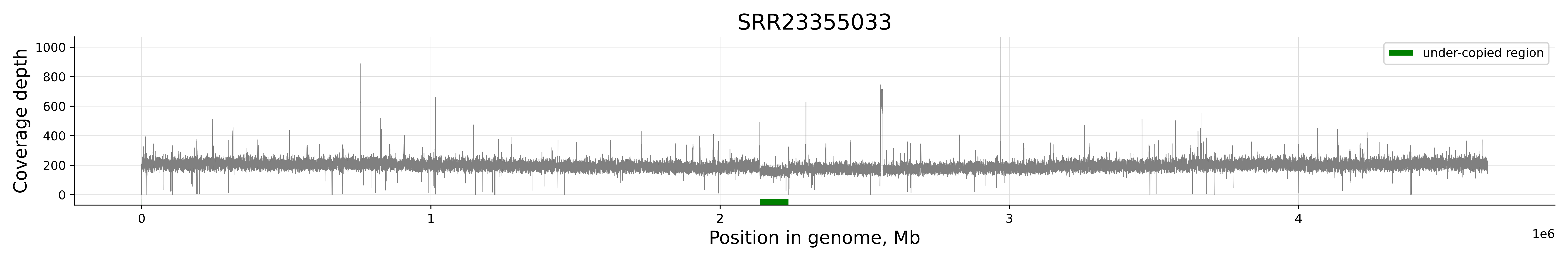

SRR23355033
Coverage depth
Position in genome, Mb
under-copied region
1000
800
600
400
200
0
0
1
2
3
4
1e6

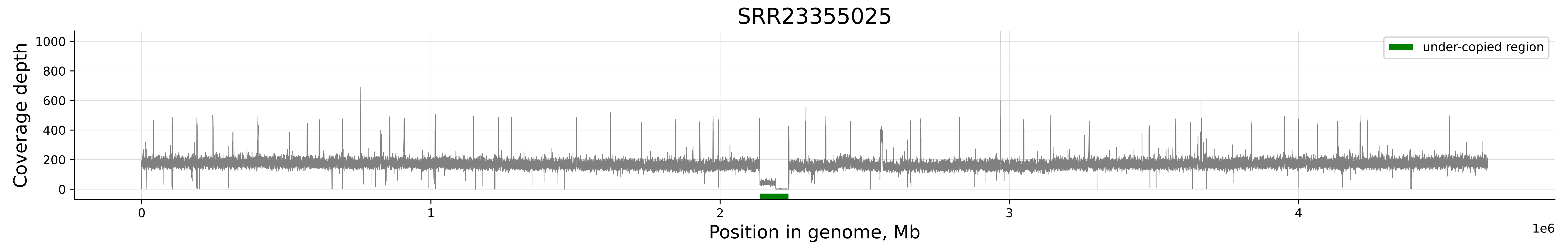

SRR23355025
under-copied region
Coverage depth
0
200
400
600
800
1000
0
1
2
3
4
1e6
Position in genome, Mb

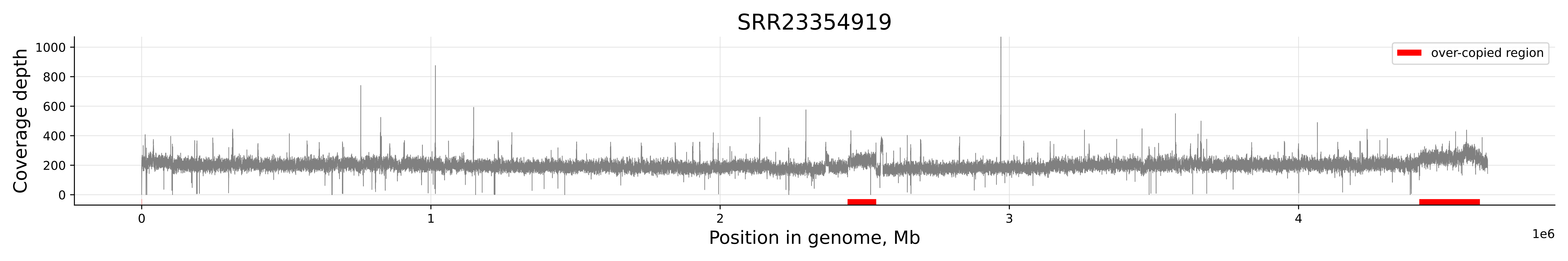

SRR23354919
Coverage depth
1000
800
600
400
200
0
over-copied region
0
1
2
3
4
1e6
Position in genome, Mb

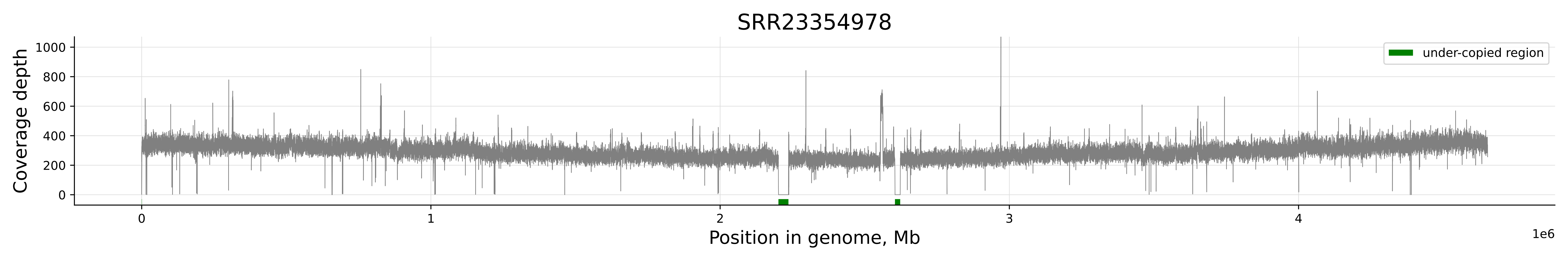
SRR23354978
Coverage depth
Position in genome, Mb
1e6
under-copied region
0
200
400
600
800
1000
0
1
2
3
4

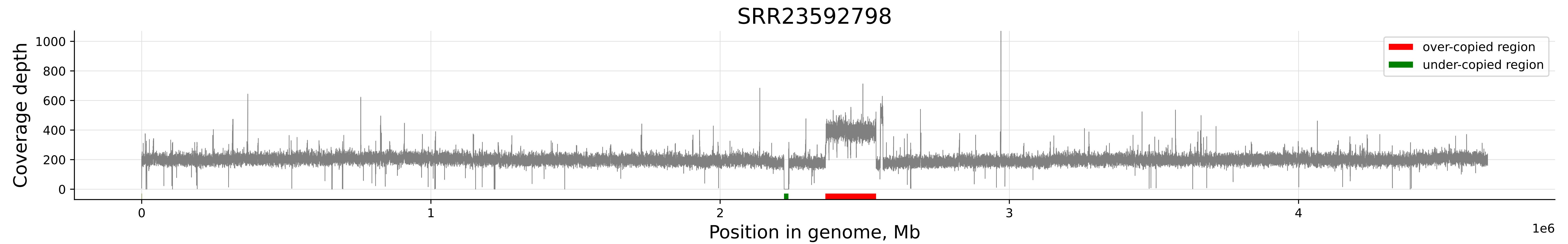
SRR23592798
Coverage depth
1000
800
600
400
200
0
over-copied region
under-copied region
0
1
2
3
4
1e6
Position in genome, Mb

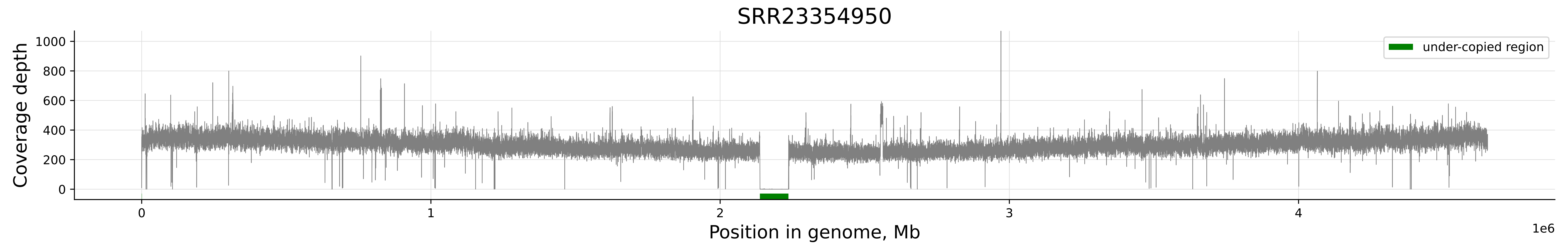

SRR23354950
Coverage depth
Position in genome, Mb
under-copied region
1000
800
600
400
200
0
0
1
2
3
4
1e6

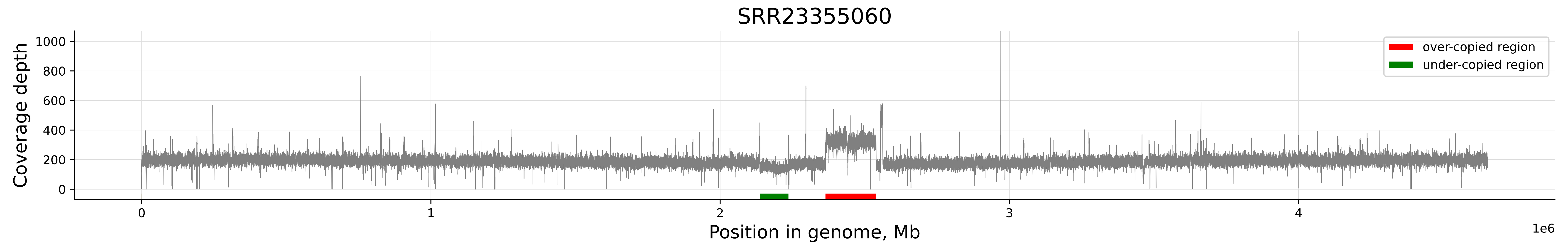

SRR23355060
Coverage depth
Position in genome, Mb
over-copied region
under-copied region
1000
800
600
400
200
0
0
1
2
3
4
1e6

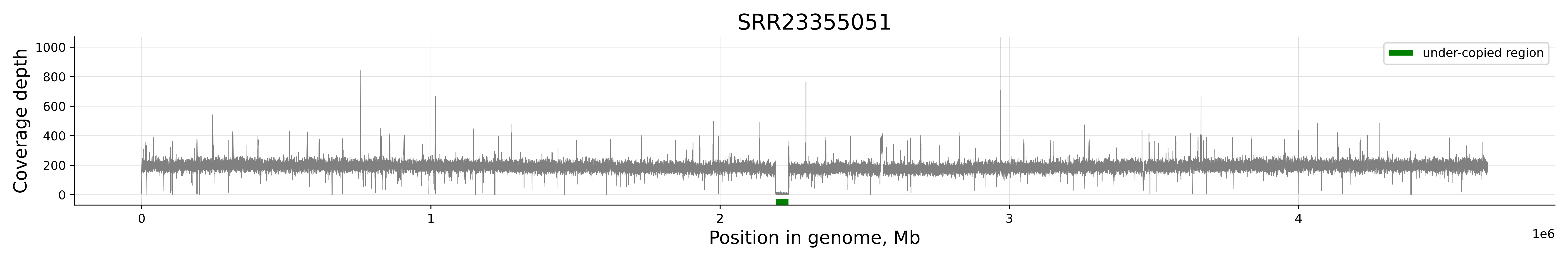
SRR23355051
under-copied region
Coverage depth
1000
800
600
400
200
0
0
1
2
3
4
1e6
Position in genome, Mb

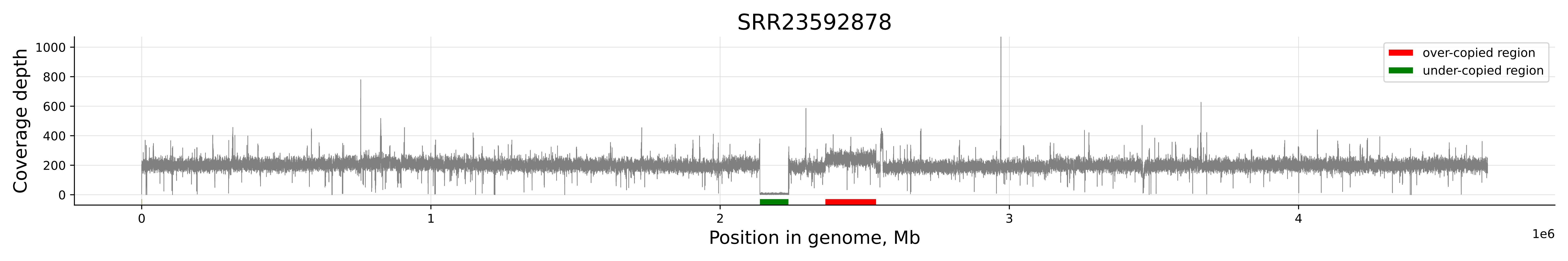
SRR23592878
Coverage depth
1000
800
600
400
200
0
0
1
2
3
4
1e6
Position in genome, Mb
over-copied region
under-copied region

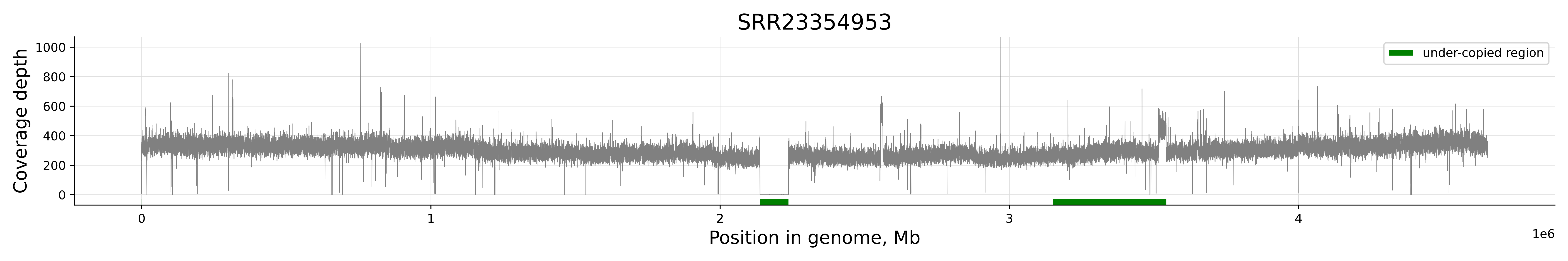

SRR23354953
under-copied region
Coverage depth
0
200
400
600
800
1000
0
1
2
3
4
1e6
Position in genome, Mb

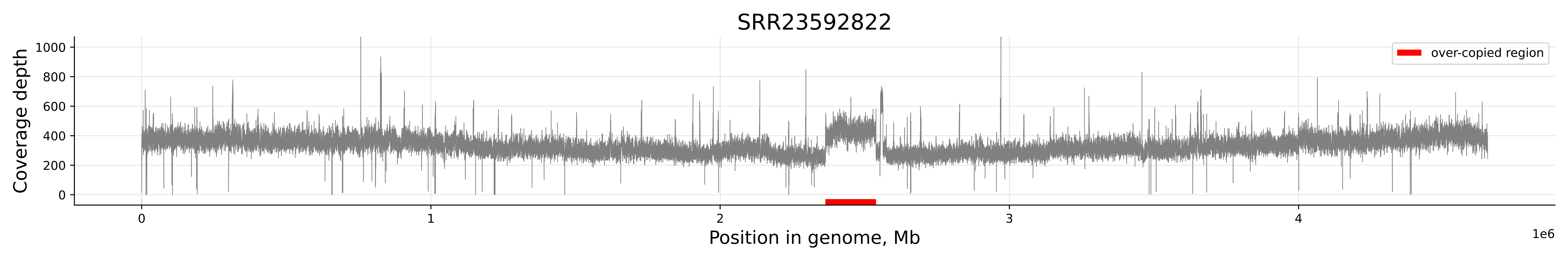

SRR23592822
over-copied region
Coverage depth
0
200
400
600
800
1000
0
1
2
3
4
1e6
Position in genome, Mb

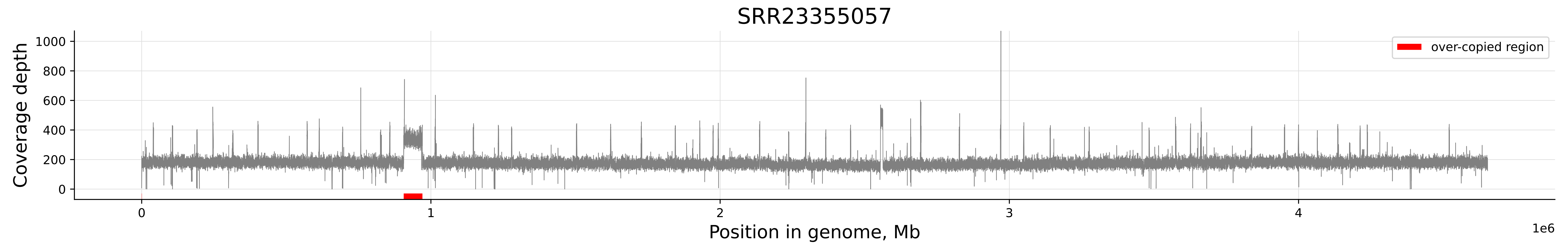

SRR23355057
Coverage depth
Position in genome, Mb
1e6
over-copied region
0
200
400
600
800
1000
0
1
2
3
4

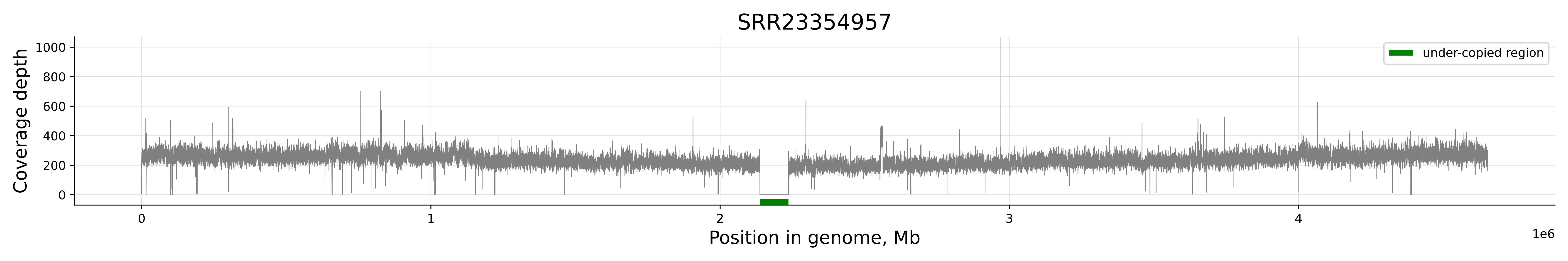
SRR23354957
under-copied region
Coverage depth
Position in genome, Mb
1e6
0
200
400
600
800
1000
0
1
2
3
4

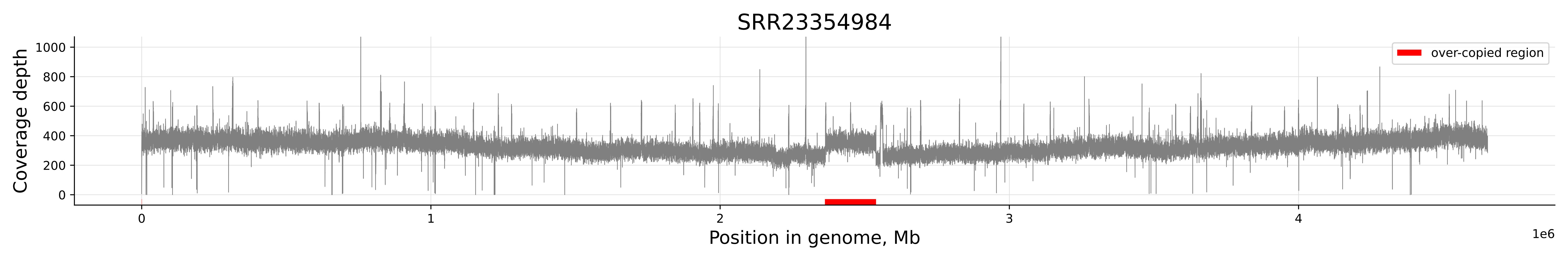

SRR23354984
Coverage depth
Position in genome, Mb
1e6
over-copied region
0
200
400
600
800
1000
0
1
2
3
4

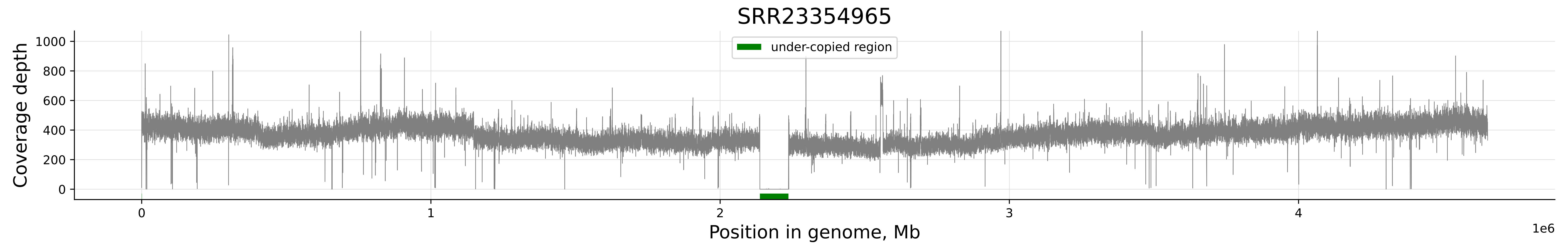

SRR23354965
under-copied region
Coverage depth
1000
800
600
400
200
0
0
1
2
3
4
1e6
Position in genome, Mb

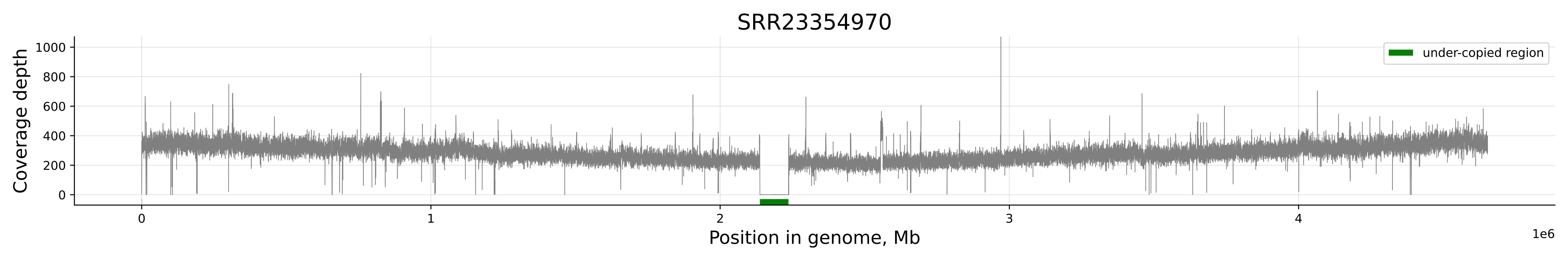

SRR23354970
Coverage depth
Position in genome, Mb
under-copied region
1000
800
600
400
200
0
0
1
2
3
4
1e6

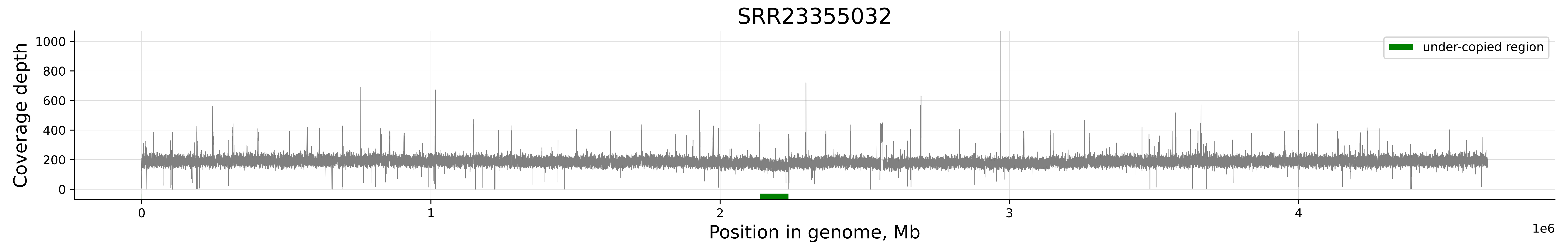

SRR23355032
under-copied region
Coverage depth
1000
800
600
400
200
0
0
1
2
3
4
1e6
Position in genome, Mb

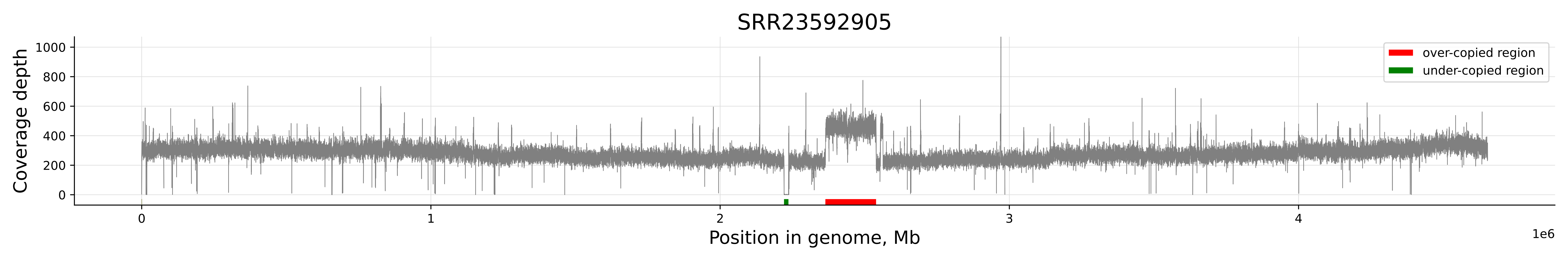
SRR23592905
Coverage depth
Position in genome, Mb
1e6
over-copied region
under-copied region
0
200
400
600
800
1000
0
1
2
3
4

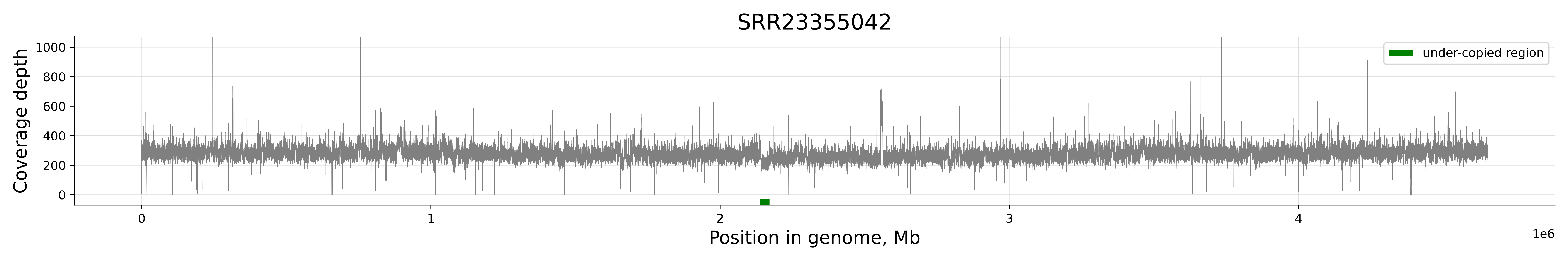

SRR23355042
Coverage depth
1000
800
600
400
200
0
under-copied region
0
1
2
3
4
1e6
Position in genome, Mb

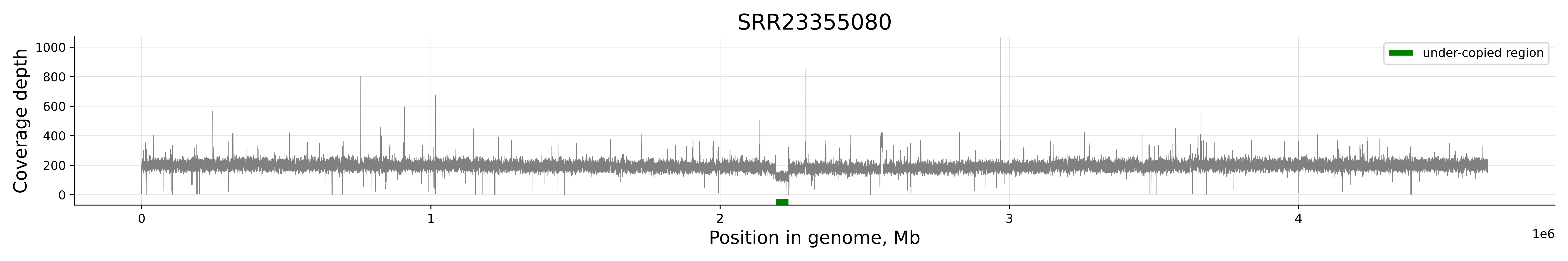

SRR23355080
Coverage depth
0
200
400
600
800
1000
under-copied region
0
1
2
3
4
1e6
Position in genome, Mb

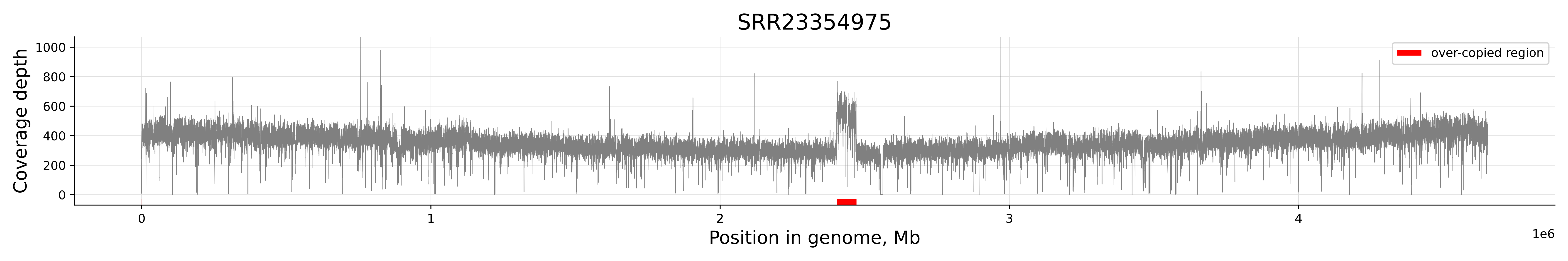
SRR23354975
Coverage depth
1000
800
600
400
200
0
over-copied region
0
1
2
3
4
1e6
Position in genome, Mb

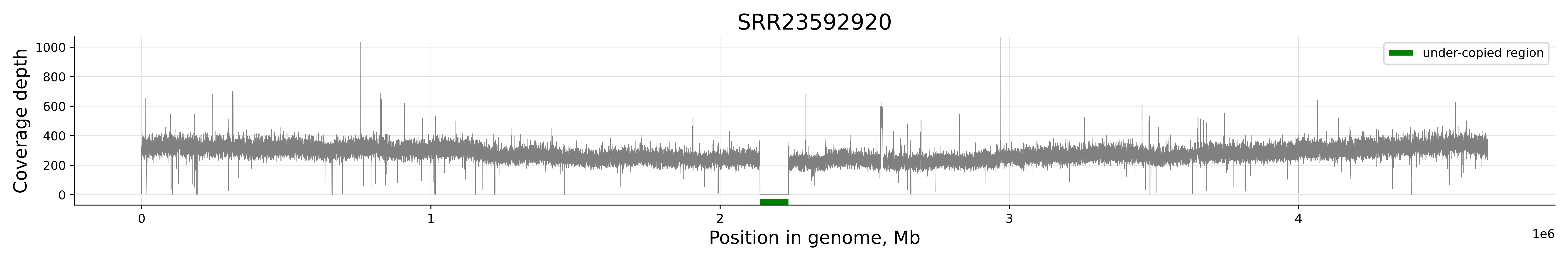
SRR23592920
Coverage depth
Position in genome, Mb
under-copied region
1e6
0
200
400
600
800
1000
0
1
2
3
4

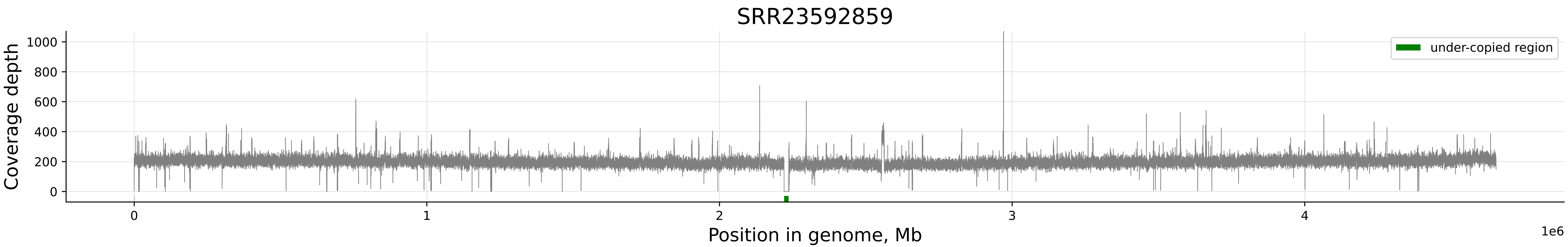

SRR23592859
Coverage depth
Position in genome, Mb
under-copied region
1000
800
600
400
200
0
1
2
3
4
1e6

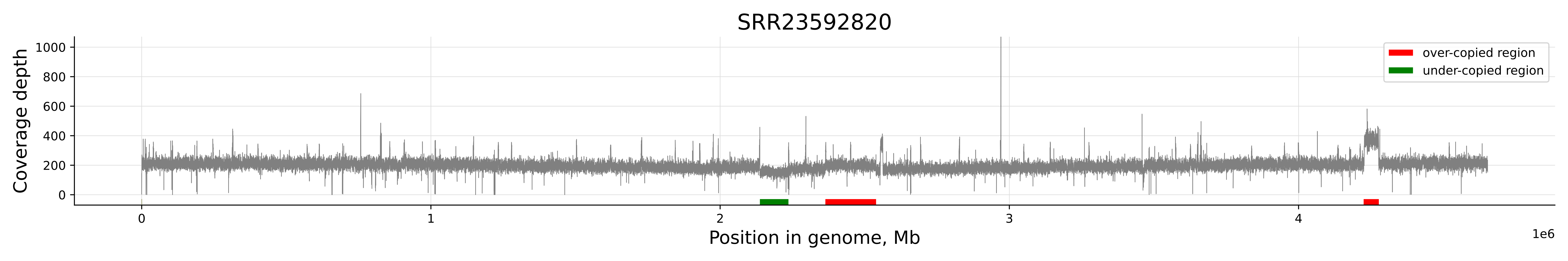

SRR23592820
Coverage depth
Position in genome, Mb
over-copied region
under-copied region
1e6

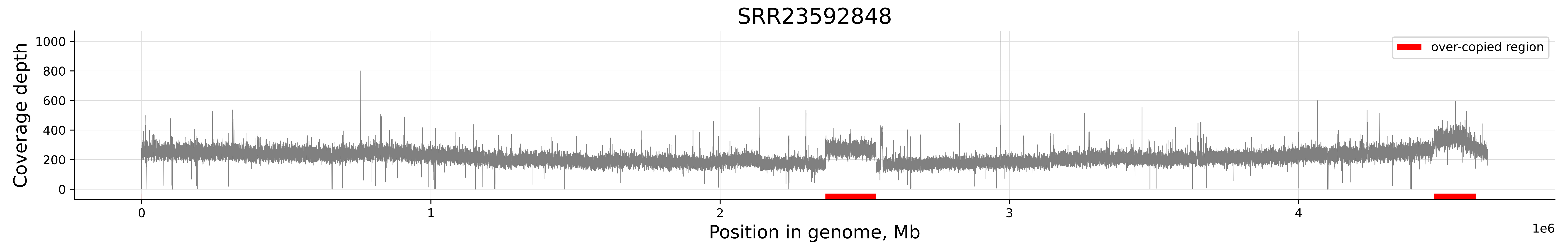

SRR23592848
over-copied region
Coverage depth
1000
800
600
400
200
0
0
1
2
3
4
1e6
Position in genome, Mb

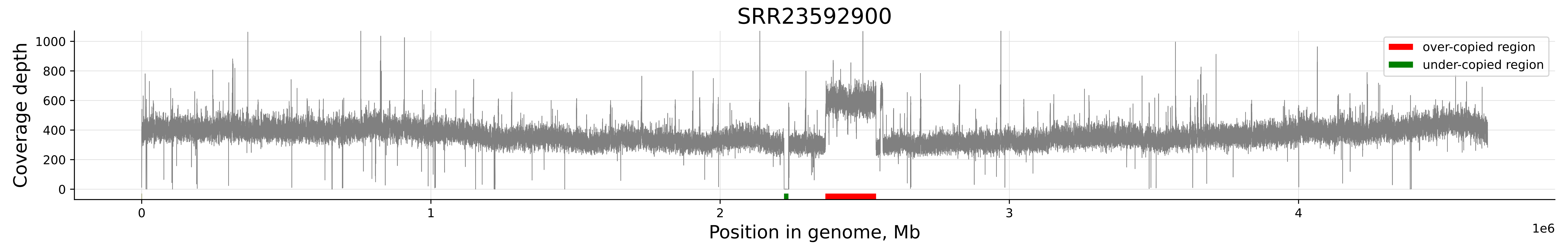

SRR23592900
over-copied region
under-copied region
Coverage depth
0
200
400
600
800
1000
0
1
2
3
4
1e6
Position in genome, Mb

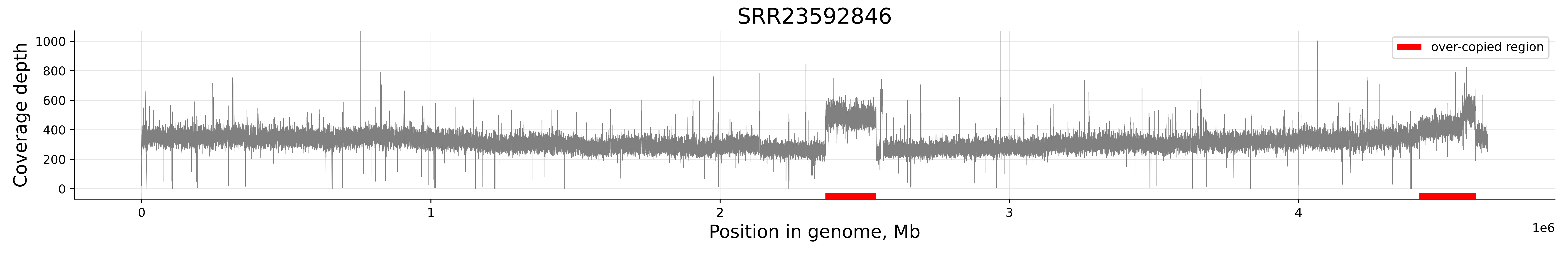

SRR23592846
Coverage depth
Position in genome, Mb
1e6
over-copied region
0
200
400
600
800
1000
0
1
2
3
4

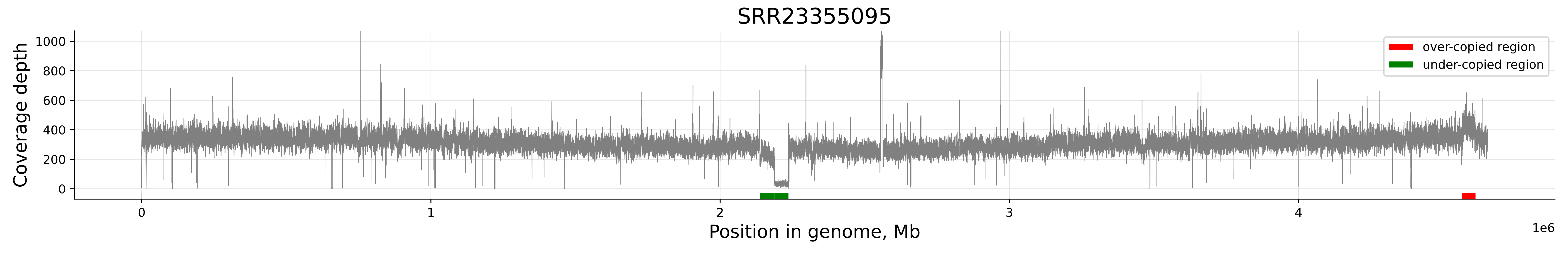
SRR23355095
Coverage depth
0
200
400
600
800
1000
Position in genome, Mb
0
1
2
3
4
1e6
over-copied region
under-copied region

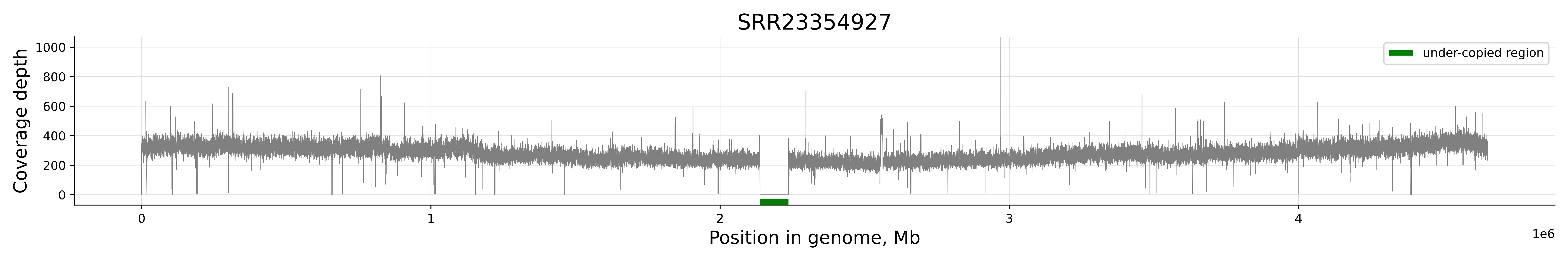

SRR23354927
under-copied region
Coverage depth
0
200
400
600
800
1000
0
1
2
3
4
1e6
Position in genome, Mb

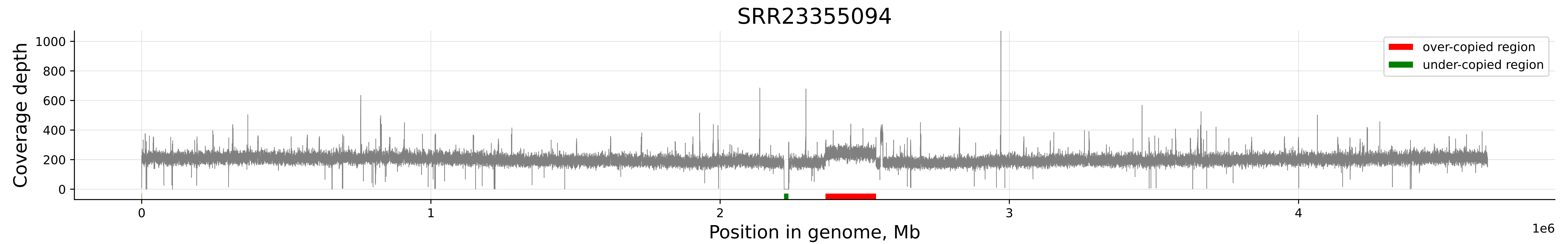

SRR23355094
Coverage depth
Position in genome, Mb
1e6
over-copied region
under-copied region
0
200
400
600
800
1000
0
1
2
3
4

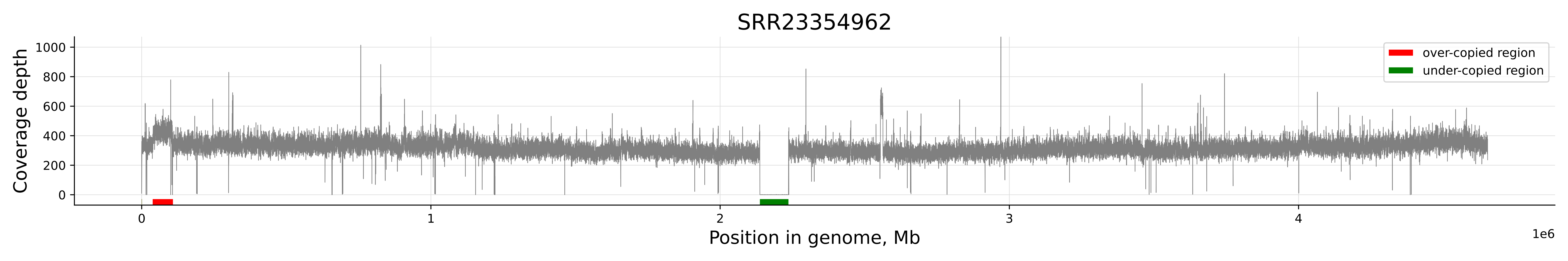
SRR23354962
Coverage depth
Position in genome, Mb
1e6
0
200
400
600
800
1000
0
1
2
3
4
over-copied region
under-copied region

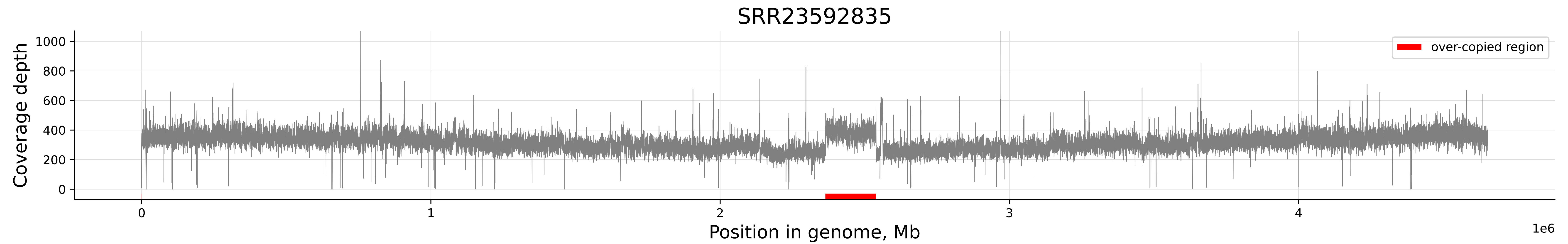

SRR23592835
over-copied region
Coverage depth
0
200
400
600
800
1000
Position in genome, Mb
0
1
2
3
4
1e6

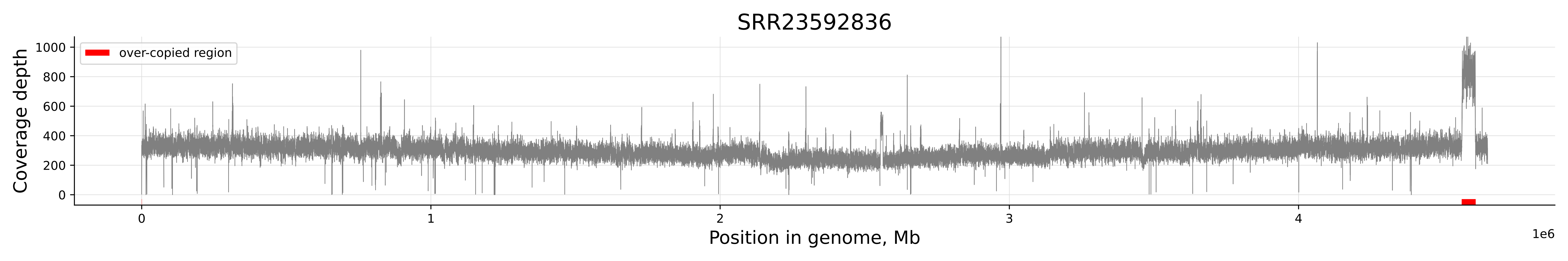

SRR23592836
over-copied region
Coverage depth
0
200
400
600
800
1000
0
1
2
3
4
1e6
Position in genome, Mb

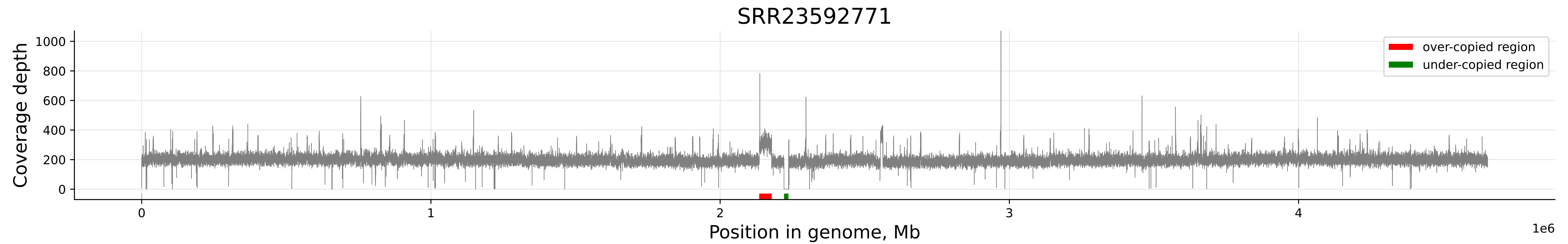

SRR23592771
Coverage depth
1000
800
600
400
200
0
0
1
2
3
4
1e6
Position in genome, Mb
over-copied region
under-copied region

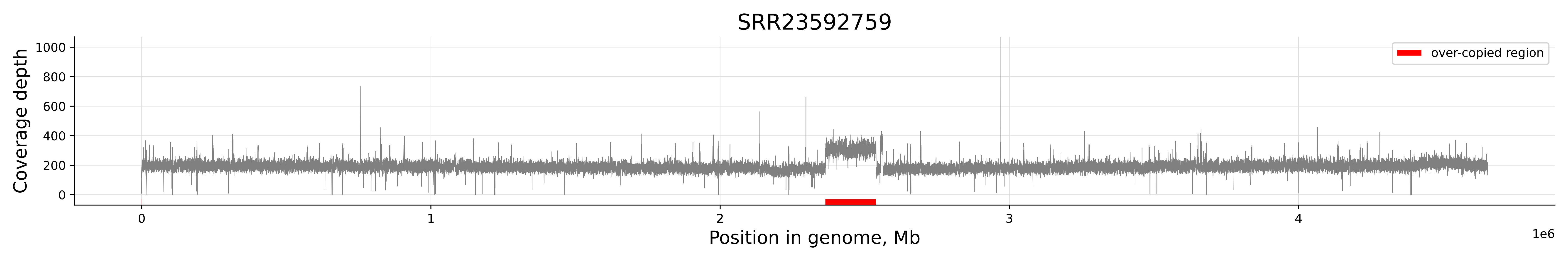
SRR23592759
Coverage depth
Position in genome, Mb
1e6
over-copied region
0
200
400
600
800
1000
0
1
2
3
4

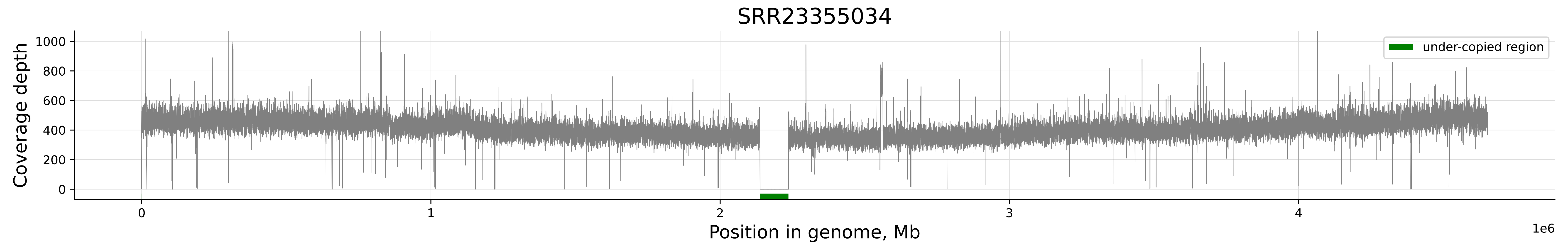

SRR23355034
Coverage depth
Position in genome, Mb
under-copied region
1000
800
600
400
200
0
0
1
2
3
4
1e6

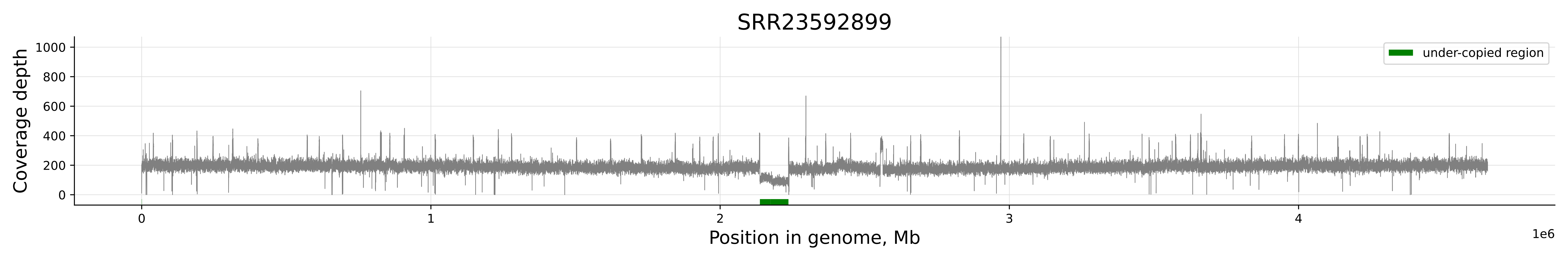
SRR23592899
Coverage depth
1000
800
600
400
200
0
under-copied region
0
1
2
3
4
1e6
Position in genome, Mb

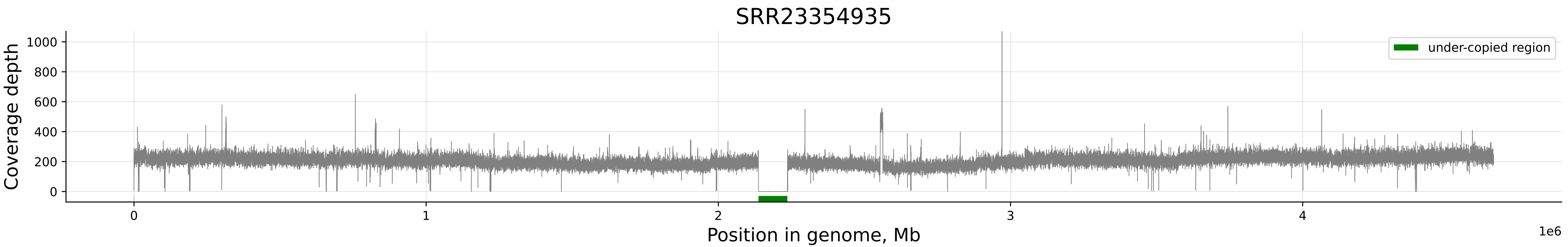

SRR23354935
under-copied region
Coverage depth
0
200
400
600
800
1000
0
1
2
3
4
1e6
Position in genome, Mb

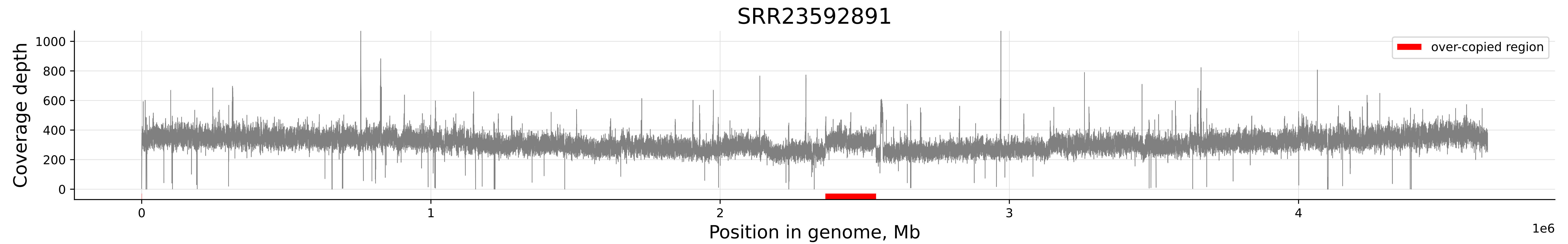

SRR23592891
Coverage depth
Position in genome, Mb
1e6
over-copied region
0
200
400
600
800
1000
0
1
2
3
4

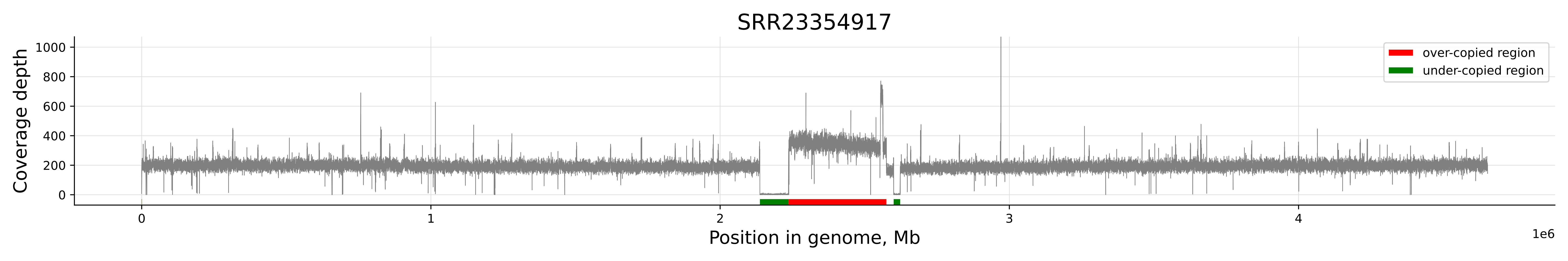

SRR23354917
over-copied region
under-copied region
Coverage depth
0
200
400
600
800
1000
0
1
2
3
4
1e6
Position in genome, Mb

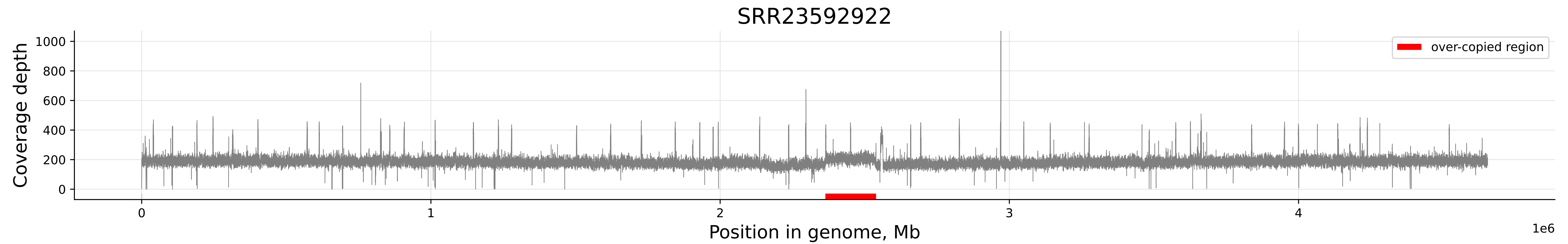

SRR23592922
over-copied region
Coverage depth
0
200
400
600
800
1000
0
1
2
3
4
1e6
Position in genome, Mb

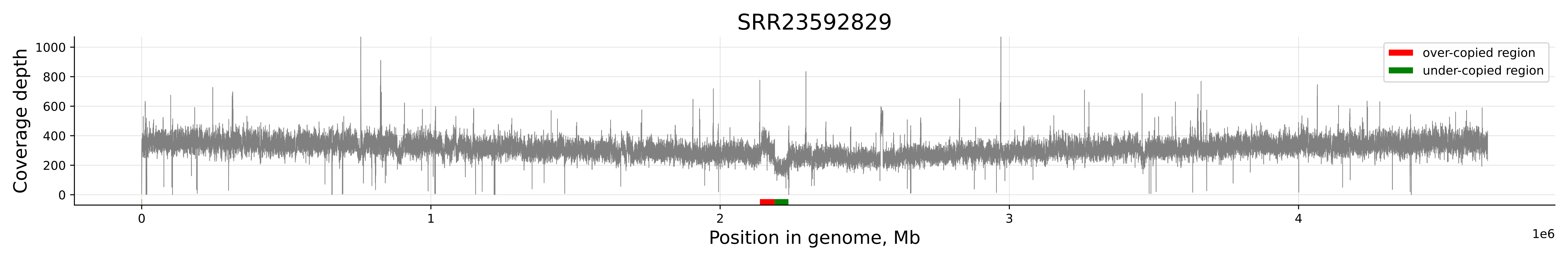

SRR23592829
over-copied region
under-copied region
Coverage depth
Position in genome, Mb
1e6
0
200
400
600
800
1000
0
1
2
3
4

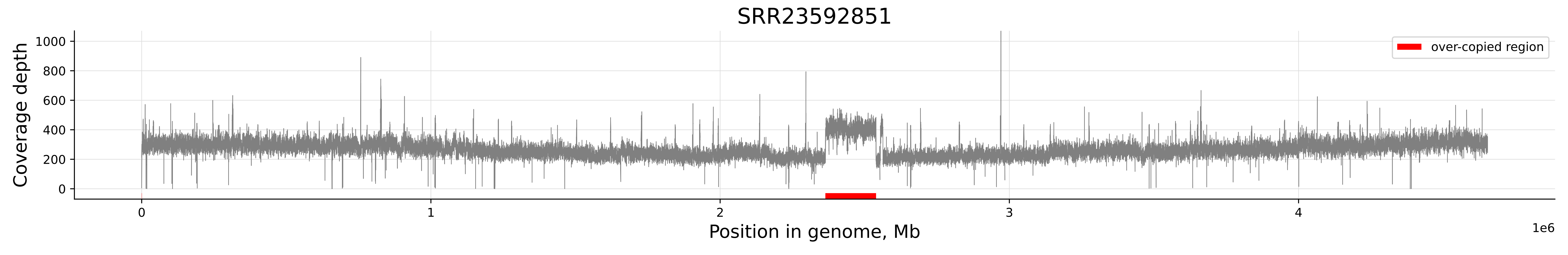

SRR23592851
Coverage depth
Position in genome, Mb
1e6
over-copied region
0
200
400
600
800
1000
0
1
2
3
4

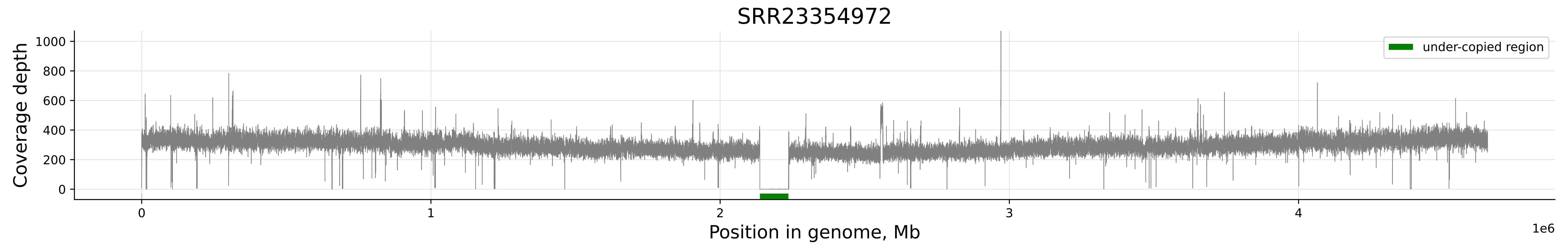

SRR23354972
under-copied region
Coverage depth
1000
800
600
400
200
0
0
1
2
3
4
1e6
Position in genome, Mb

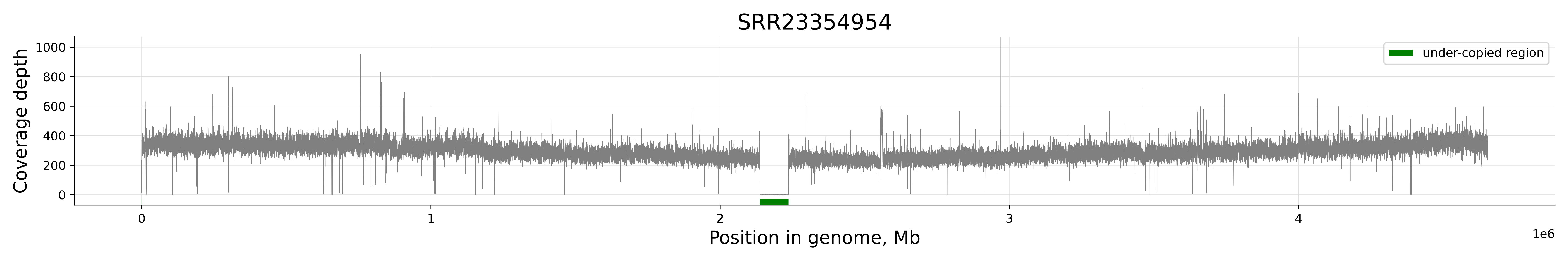
SRR23354954
Coverage depth
Position in genome, Mb
1e6
under-copied region
0
200
400
600
800
1000
0
1
2
3
4

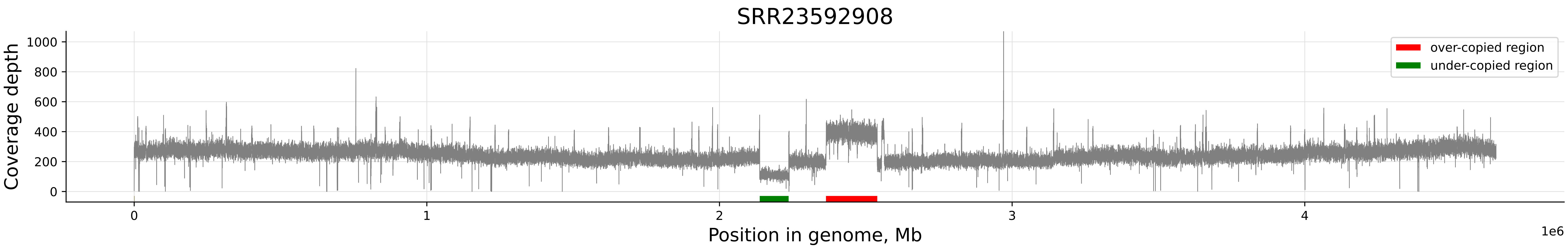

SRR23592908
over-copied region
under-copied region
Coverage depth
0
200
400
600
800
1000
0
1
2
3
4
1e6
Position in genome, Mb

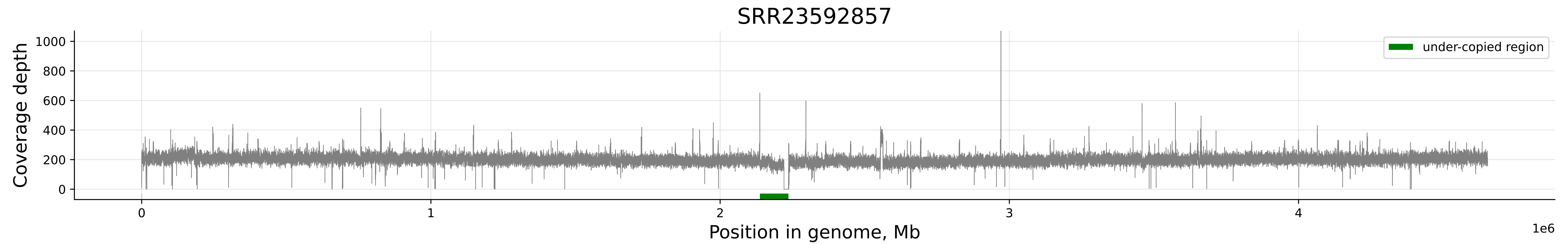

SRR23592857
under-copied region
Coverage depth
0
200
400
600
800
1000
Position in genome, Mb
0
1
2
3
4
1e6

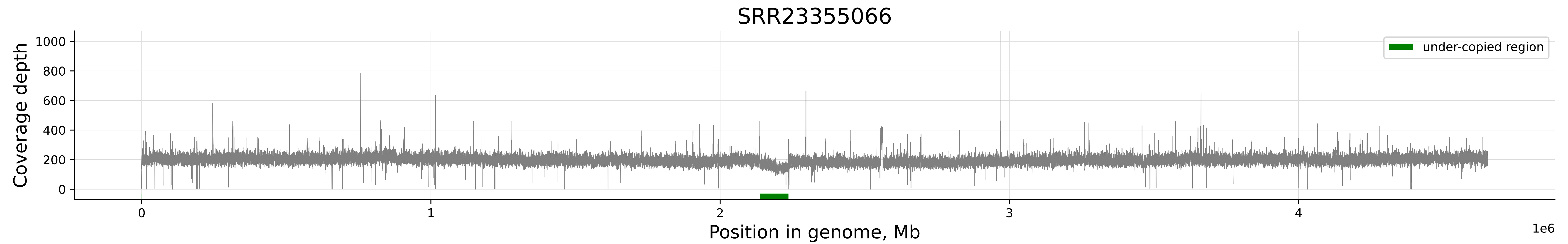

SRR23355066
Coverage depth
0
200
400
600
800
1000
under-copied region
0
1
2
3
4
1e6
Position in genome, Mb

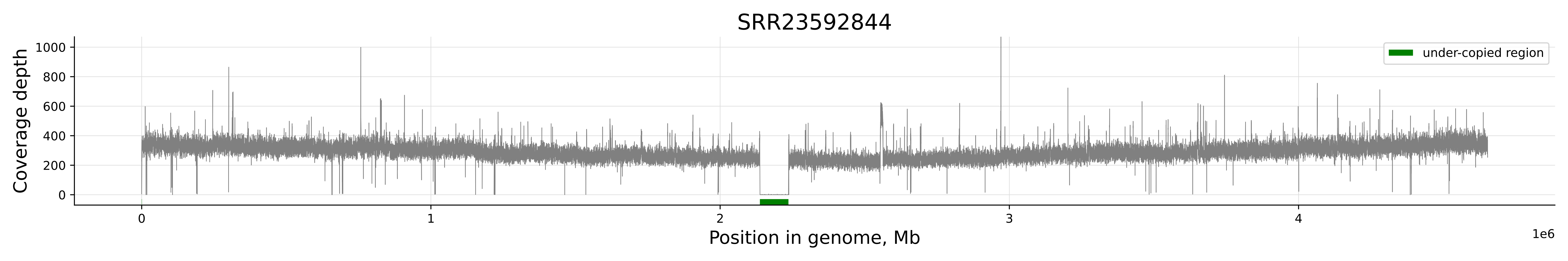
SRR23592844
Coverage depth
1000
800
600
400
200
0
under-copied region
0
1
2
3
4
1e6
Position in genome, Mb

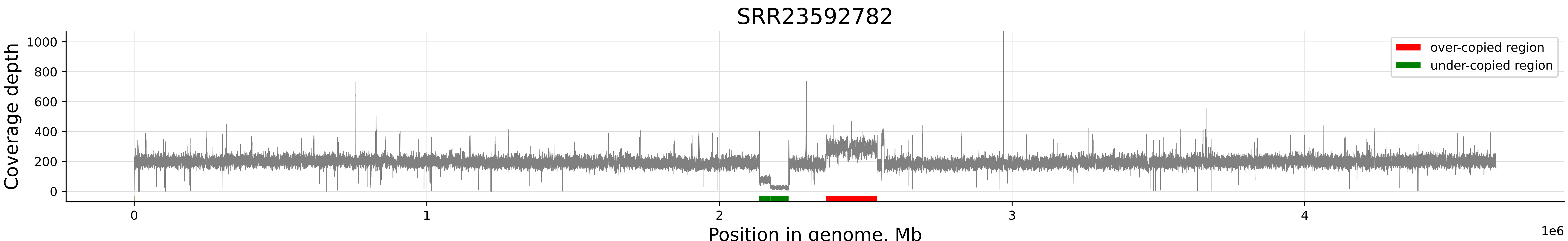
SRR23592782
Coverage depth
Position in genome, Mb
1e6
over-copied region
under-copied region
0
200
400
600
800
1000
0
1
2
3
4

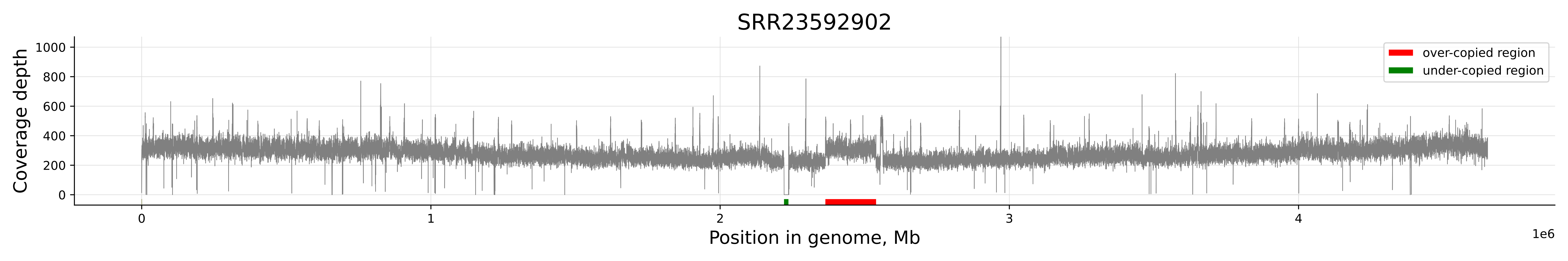

SRR23592902
Coverage depth
Position in genome, Mb
1e6
0
1
2
3
4
0
200
400
600
800
1000
over-copied region
under-copied region

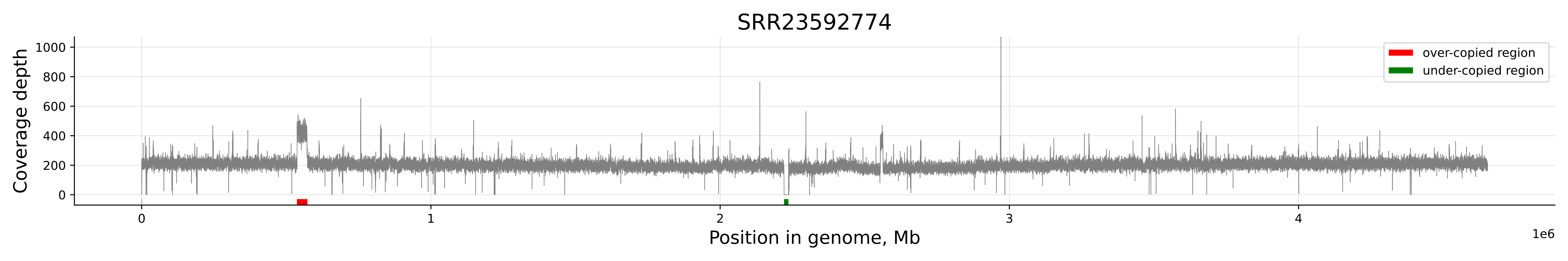
SRR23592774
Coverage depth
Position in genome, Mb
over-copied region
under-copied region
1000
800
600
400
200
0
0
1
2
3
4
1e6

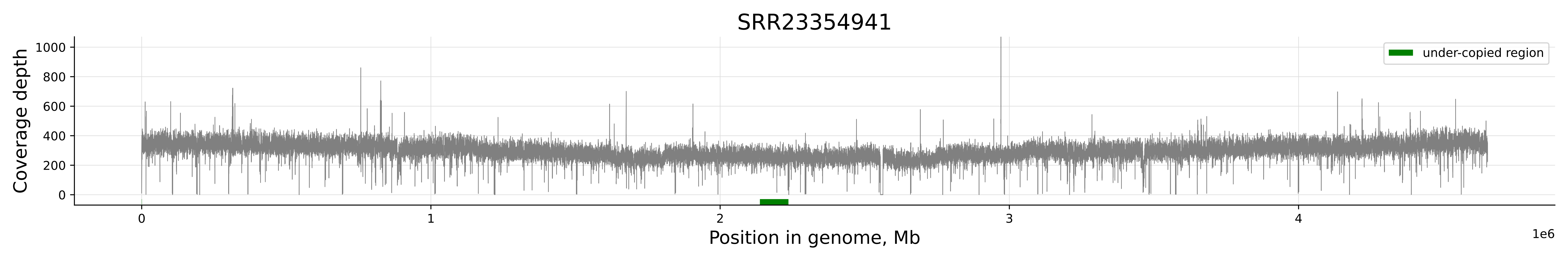
SRR23354941
under-copied region
Coverage depth
0
200
400
600
800
1000
0
1
2
3
4
1e6
Position in genome, Mb

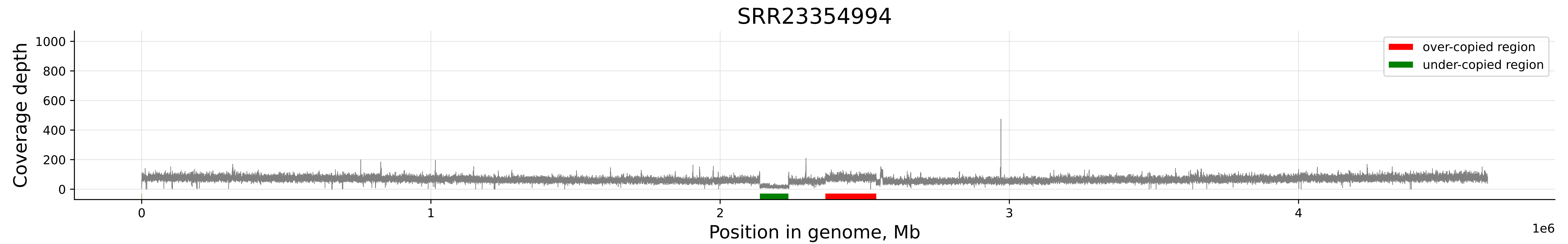
SRR23354994
Coverage depth
0
200
400
600
800
1000
over-copied region
under-copied region
0
1
2
3
4
1e6
Position in genome, Mb

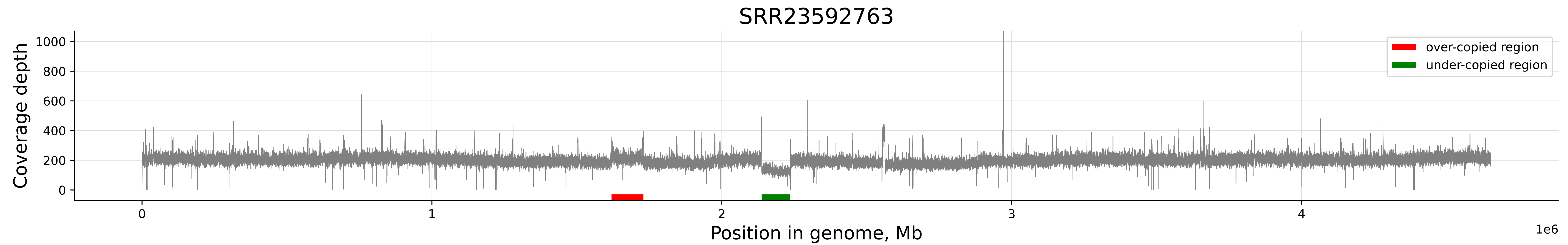

SRR23592763
Coverage depth
1000
800
600
400
200
0
over-copied region
under-copied region
0
1
2
3
4
1e6
Position in genome, Mb

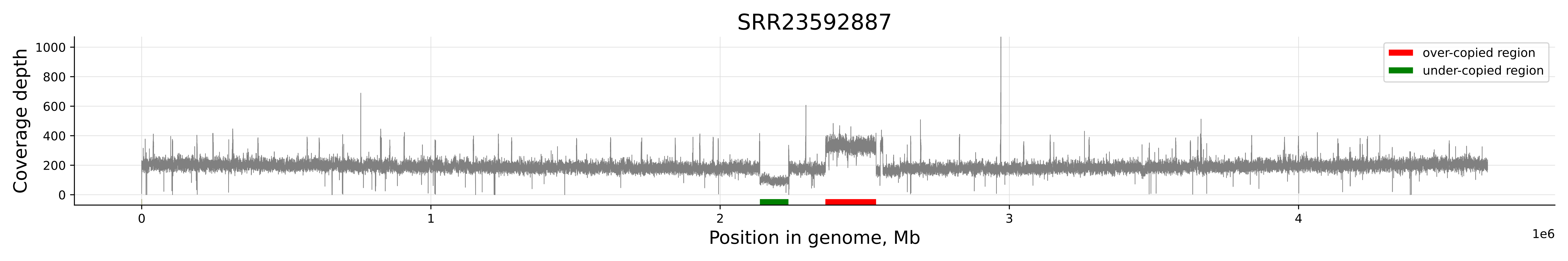
SRR23592887
Coverage depth
1000
800
600
400
200
0
over-copied region
under-copied region
0
1
2
3
4
1e6
Position in genome, Mb

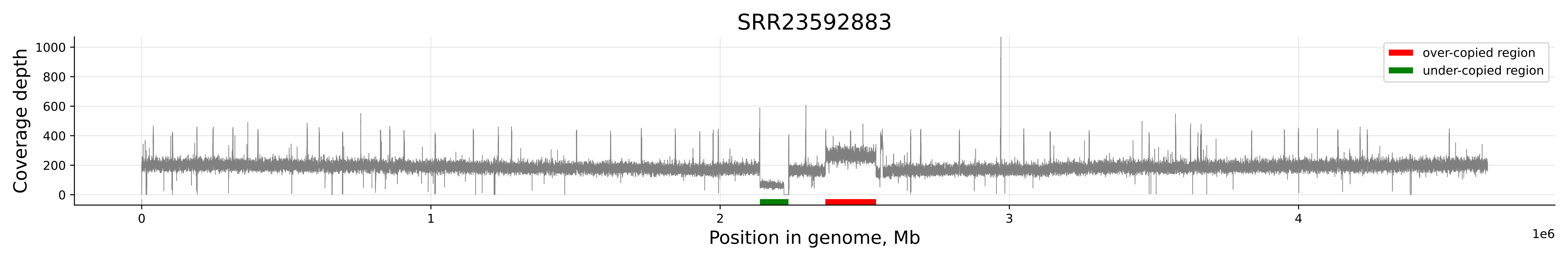
SRR23592883
Coverage depth
Position in genome, Mb
1e6
over-copied region
under-copied region
0
200
400
600
800
1000
0
1
2
3
4

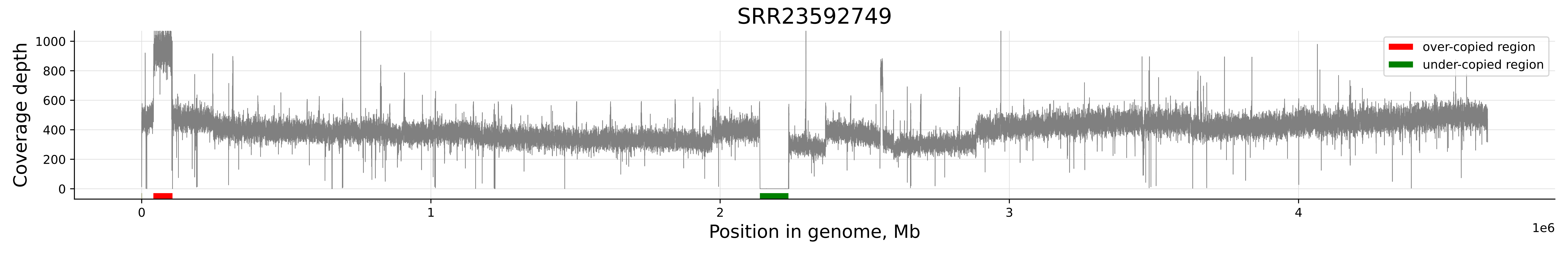
SRR23592749
Coverage depth
Position in genome, Mb
over-copied region
under-copied region
1e6

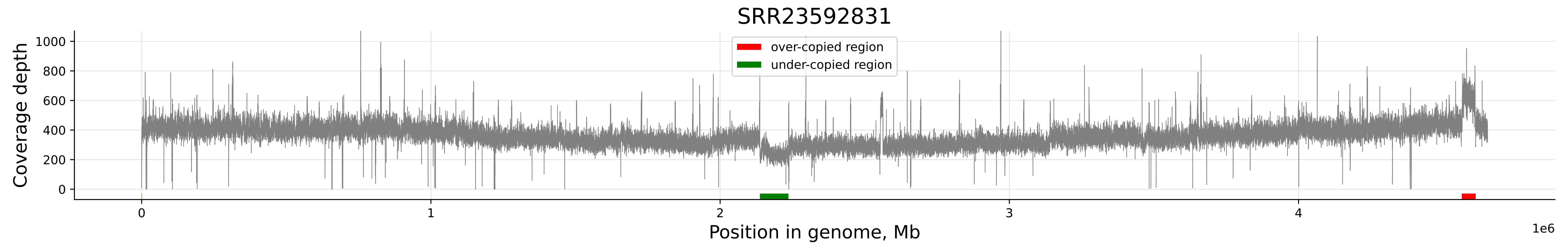
SRR23592831
over-copied region
under-copied region
Coverage depth
0
200
400
600
800
1000
0
1
2
3
4
1e6
Position in genome, Mb

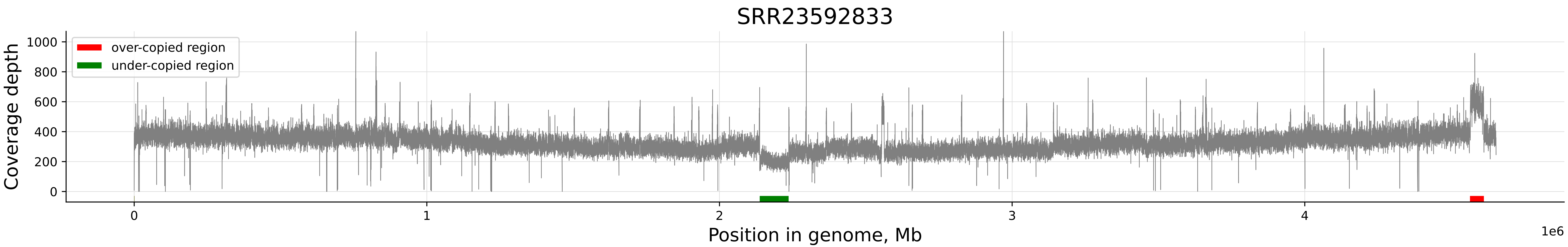
SRR23592833
over-copied region
under-copied region
Coverage depth
1000
800
600
400
200
0
0
1
2
3
4
1e6
Position in genome, Mb

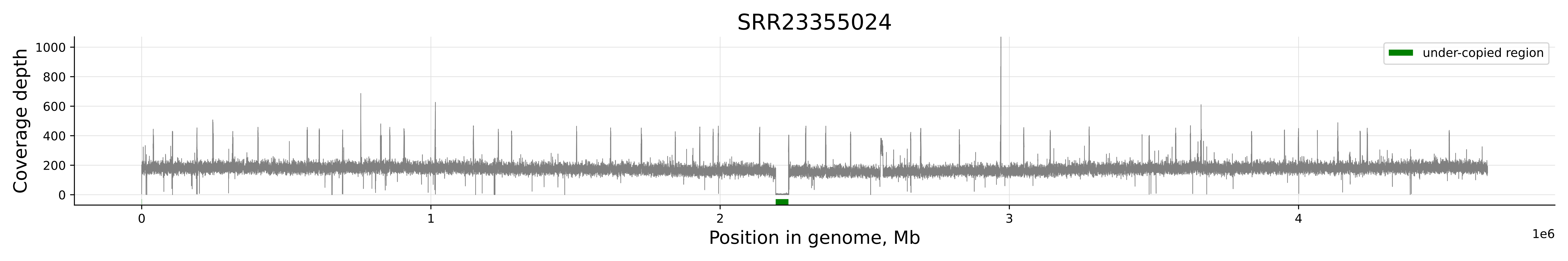

SRR23355024
under-copied region
Coverage depth
0
200
400
600
800
1000
0
1
2
3
4
1e6
Position in genome, Mb

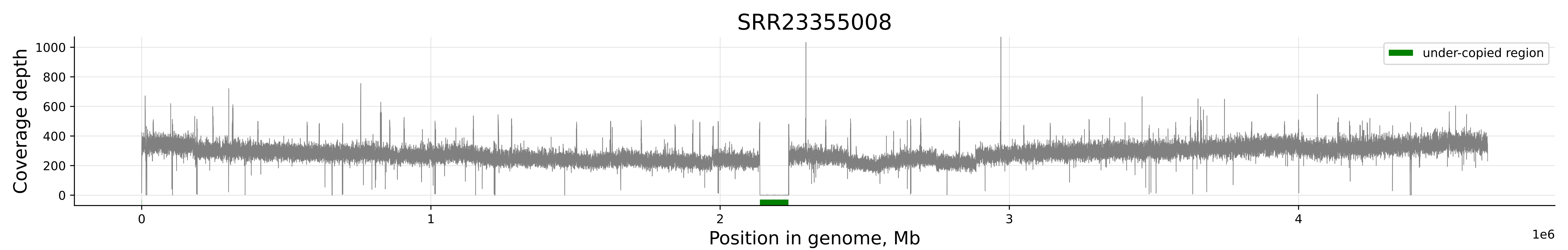

SRR23355008
Coverage depth
1000
800
600
400
200
0
0
1
2
3
4
1e6
Position in genome, Mb
under-copied region

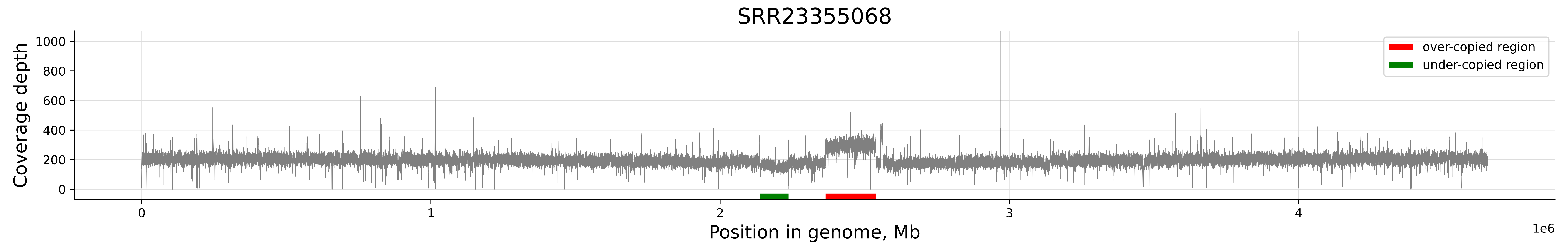
SRR23355068
over-copied region
under-copied region
Coverage depth
1000
800
600
400
200
0
0
1
2
3
4
1e6
Position in genome, Mb

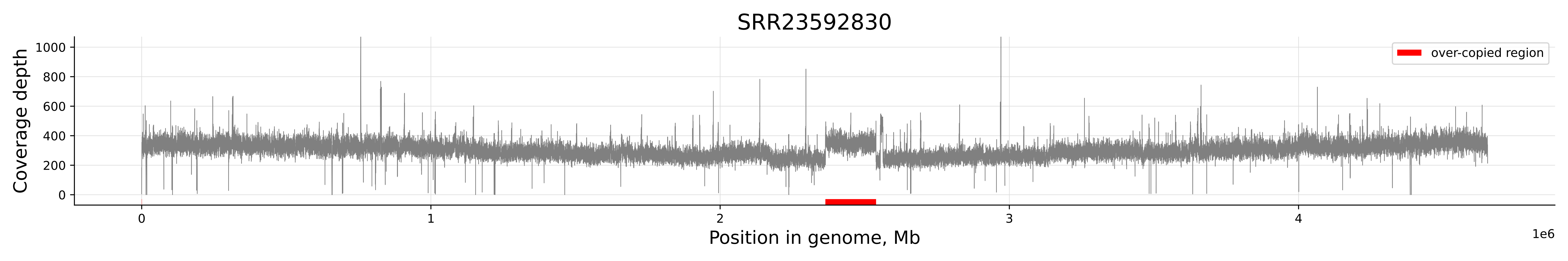

SRR23592830
over-copied region
Coverage depth
Position in genome, Mb
1e6
0
200
400
600
800
1000
0
1
2
3
4

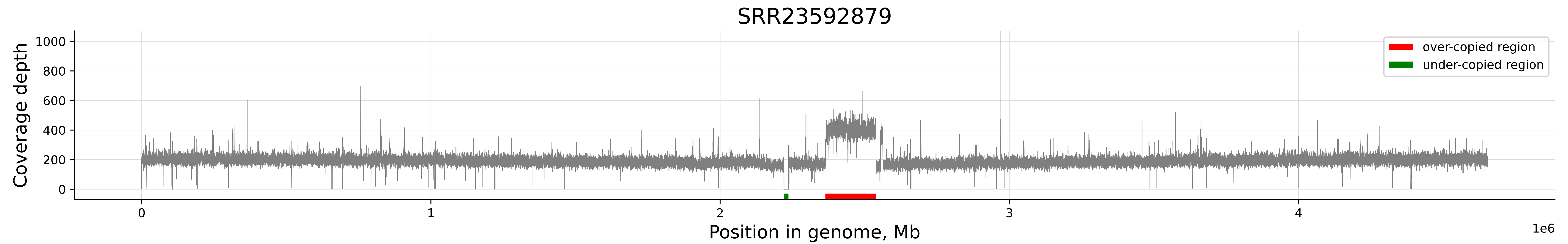

SRR23592879
Coverage depth
0
200
400
600
800
1000
over-copied region
under-copied region
0
1
2
3
4
1e6
Position in genome, Mb

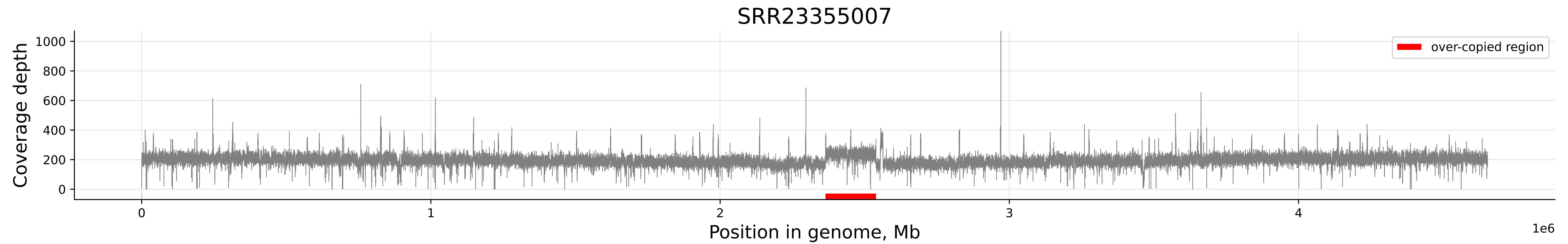
SRR23355007
Coverage depth
Position in genome, Mb
over-copied region
1000
800
600
400
200
0
0
1
2
3
4
1e6

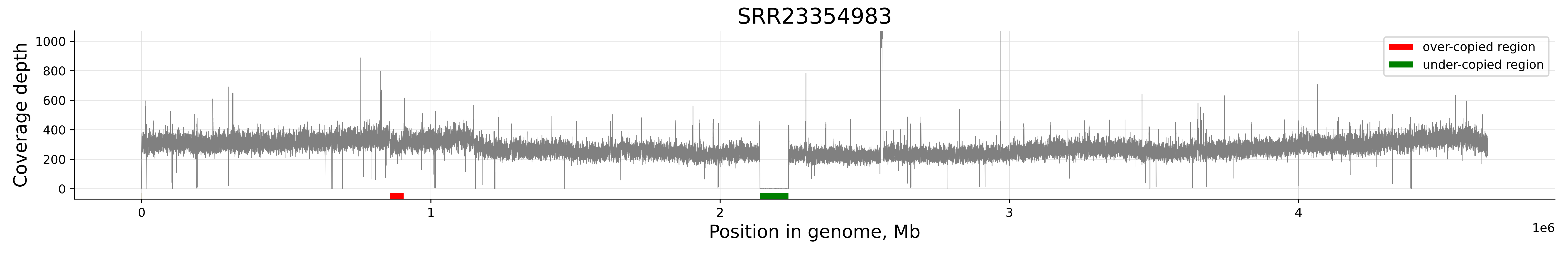

SRR23354983
Coverage depth
1000
800
600
400
200
0
over-copied region
under-copied region
0
1
2
3
4
1e6
Position in genome, Mb

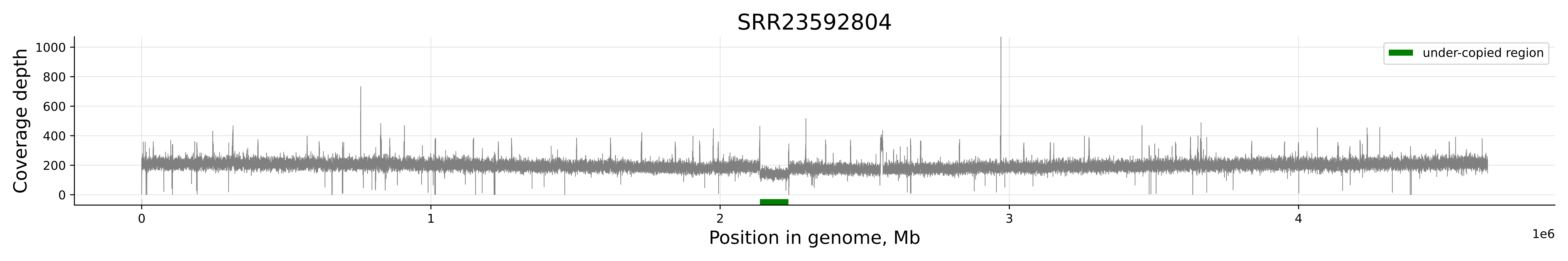

SRR23592804
under-copied region
Coverage depth
0
200
400
600
800
1000
0
1
2
3
4
1e6
Position in genome, Mb

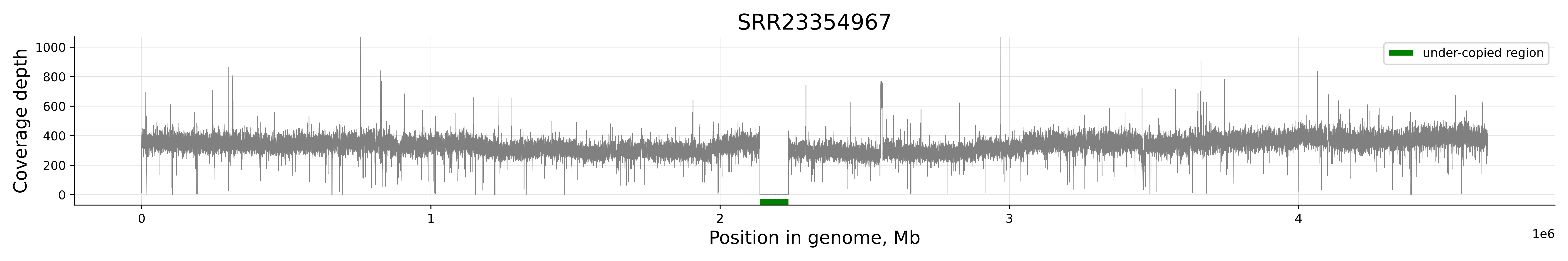

SRR23354967
Coverage depth
Position in genome, Mb
under-copied region
1000
800
600
400
200
0
1
2
3
4
1e6

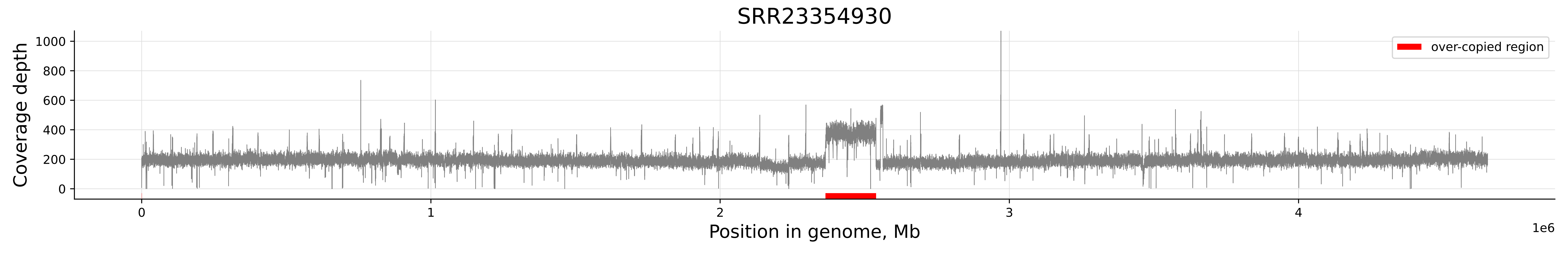

SRR23354930
over-copied region
Coverage depth
1000
800
600
400
200
0
0
1
2
3
4
1e6
Position in genome, Mb

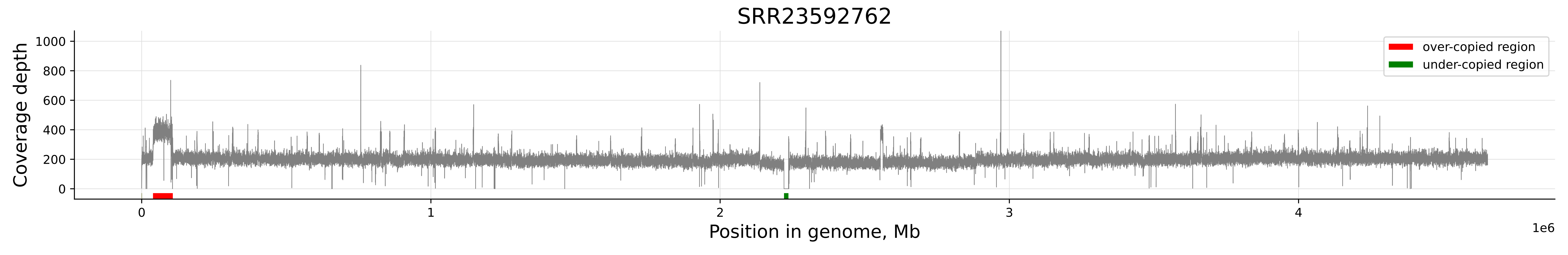

SRR23592762
Coverage depth
Position in genome, Mb
1e6
over-copied region
under-copied region
0
200
400
600
800
1000
0
1
2
3
4

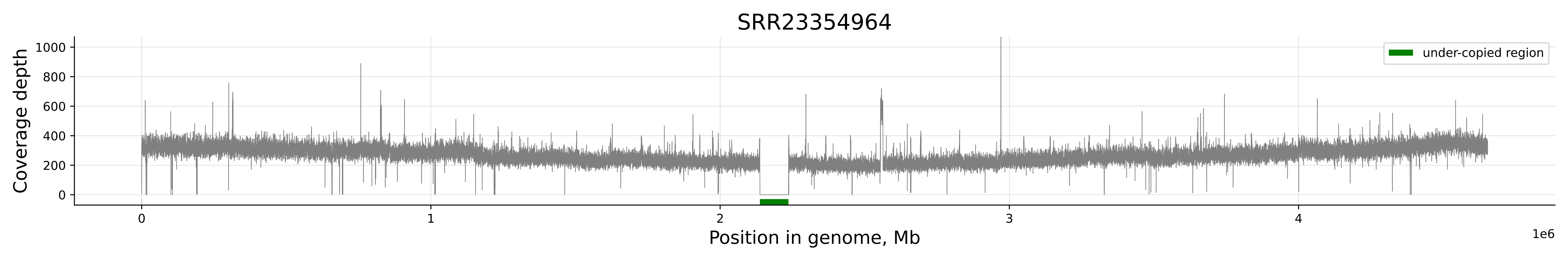
SRR23354964
under-copied region
Coverage depth
1000
800
600
400
200
0
0
1
2
3
4
1e6
Position in genome, Mb

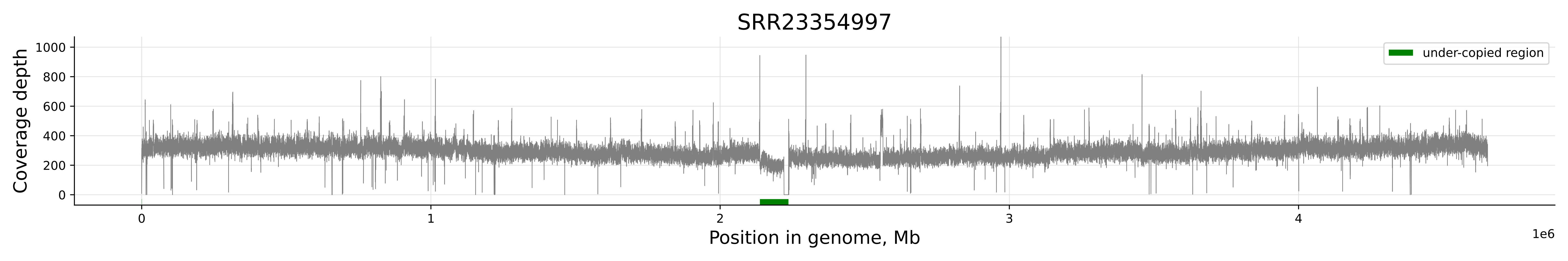

SRR23354997
under-copied region
Coverage depth
0
200
400
600
800
1000
0
1
2
3
4
1e6
Position in genome, Mb

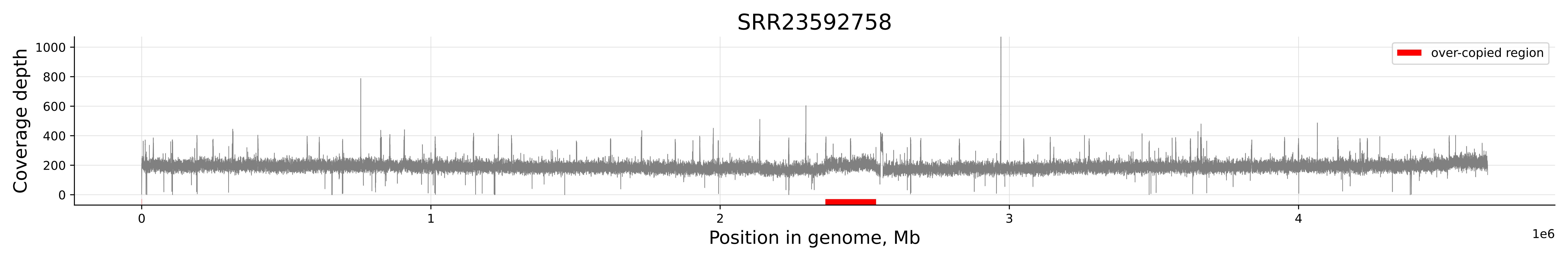

SRR23592758
over-copied region
Coverage depth
0
200
400
600
800
1000
Position in genome, Mb
0
1
2
3
4
1e6

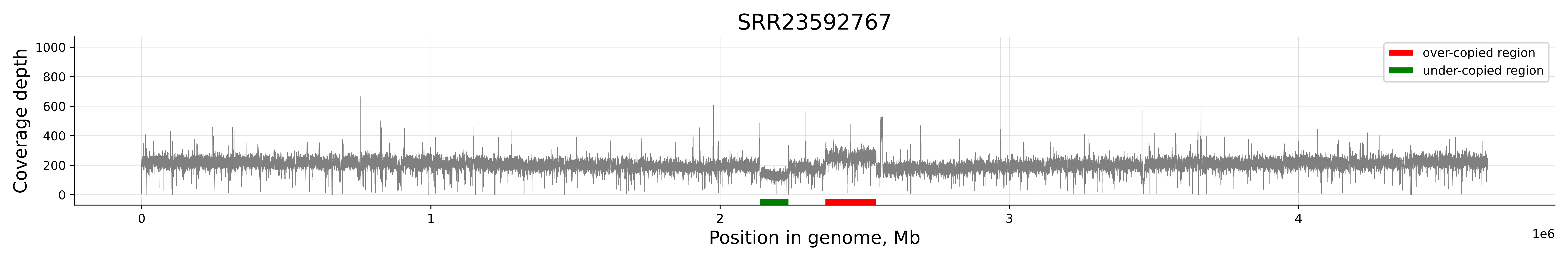

SRR23592767
Coverage depth
Position in genome, Mb
over-copied region
under-copied region
0
200
400
600
800
1000
0
1
2
3
4
1e6

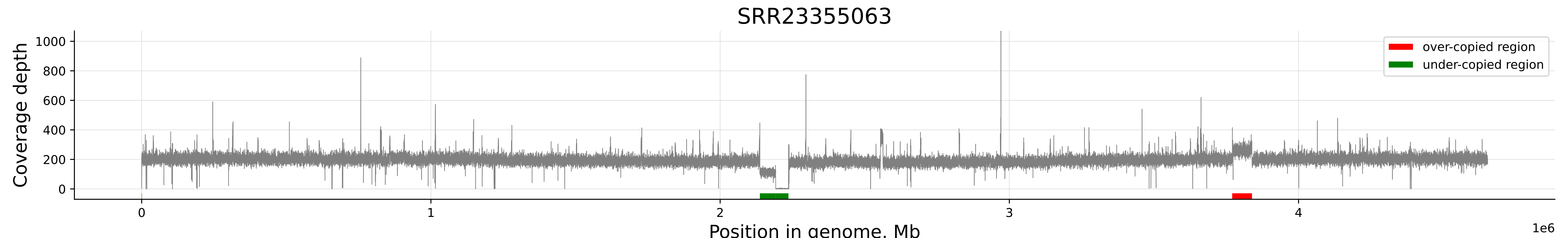
SRR23355063
Coverage depth
1000
800
600
400
200
0
over-copied region
under-copied region
0
1
2
3
4
1e6
Position in genome, Mb

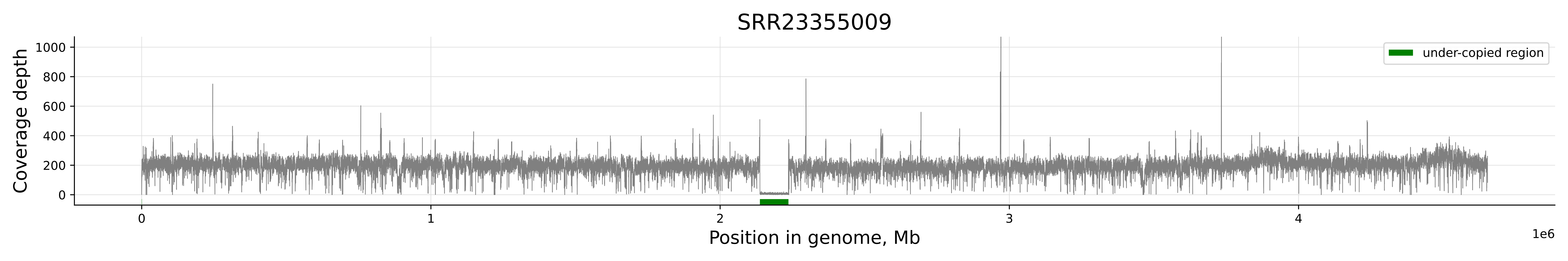

SRR23355009
Coverage depth
Position in genome, Mb
under-copied region
1e6
0
200
400
600
800
1000
0
1
2
3
4

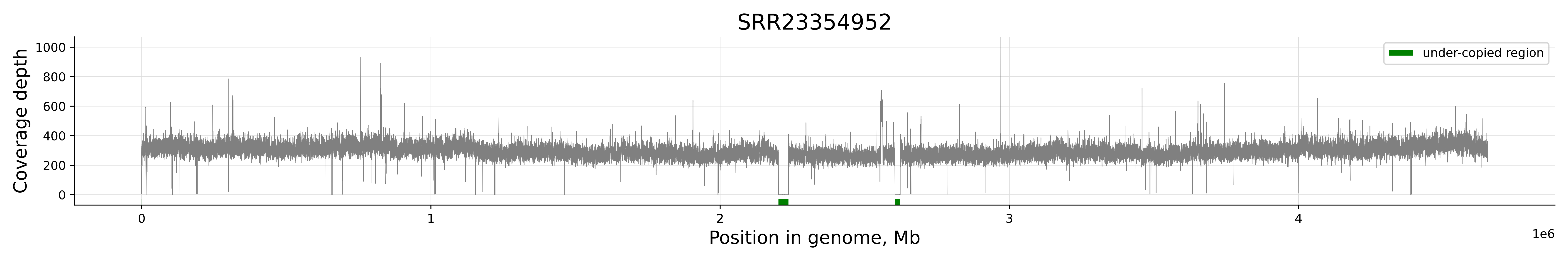

SRR23354952
Coverage depth
1000
800
600
400
200
0
0
1
2
3
4
1e6
Position in genome, Mb
under-copied region

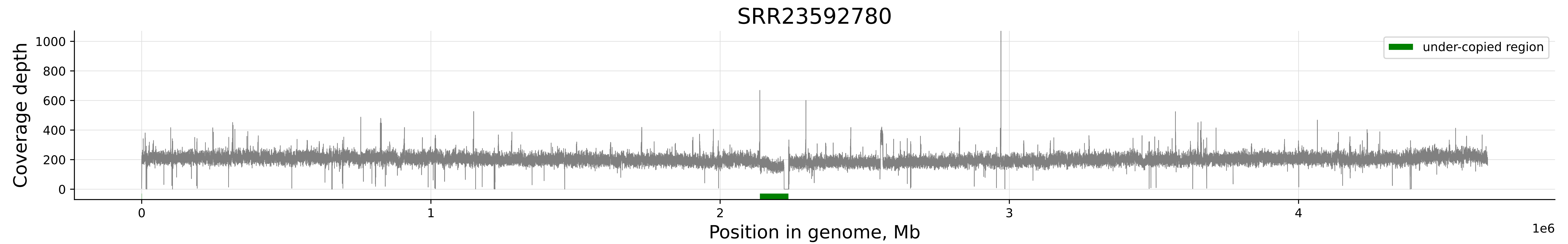

SRR23592780
under-copied region
Coverage depth
0
200
400
600
800
1000
0
1
2
3
4
1e6
Position in genome, Mb

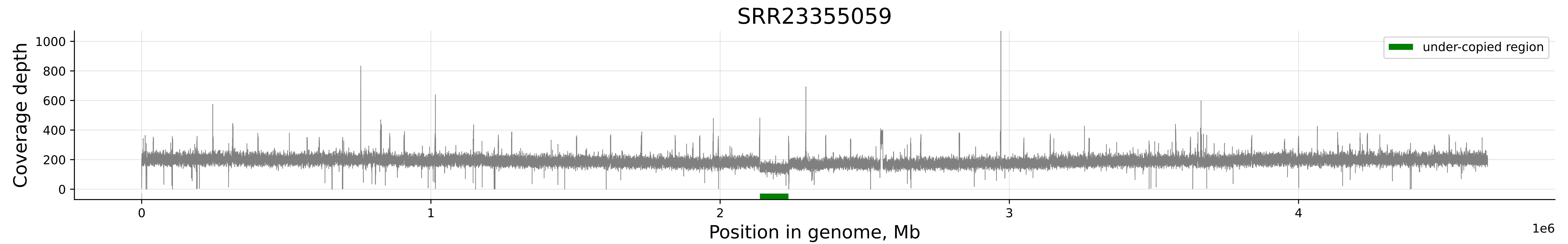
SRR23355059
under-copied region
Coverage depth
0
200
400
600
800
1000
0
1
2
3
4
1e6
Position in genome, Mb

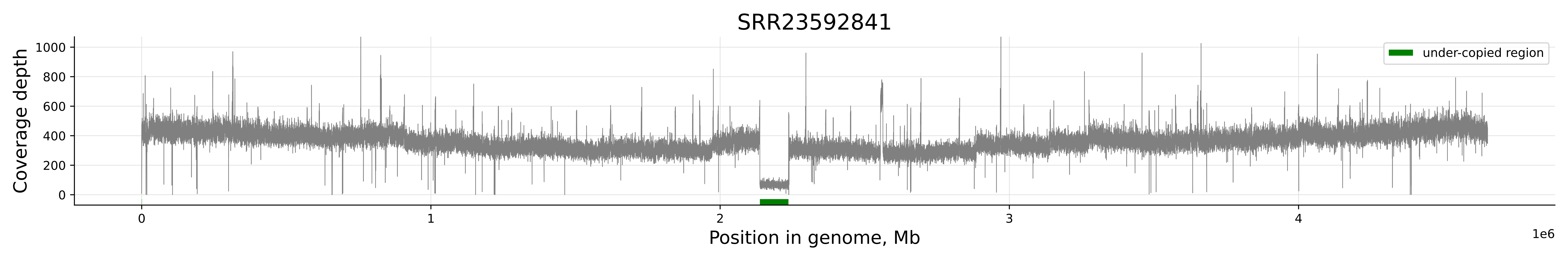

SRR23592841
under-copied region
Coverage depth
0
200
400
600
800
1000
0
1
2
3
4
1e6
Position in genome, Mb

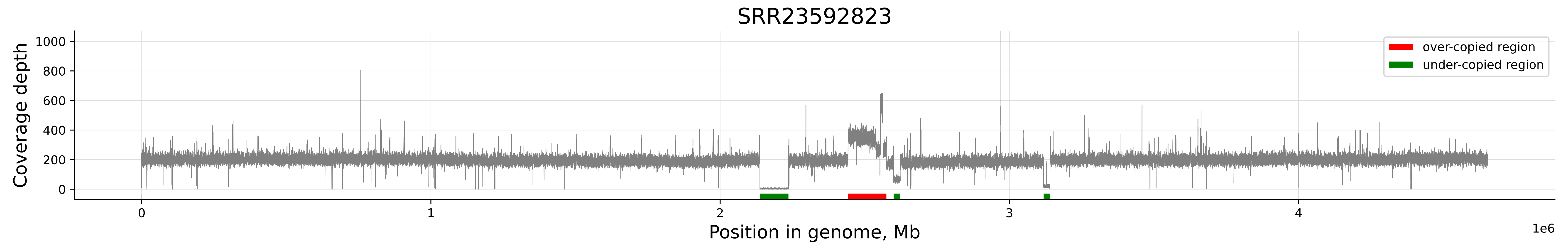
SRR23592823
Coverage depth
Position in genome, Mb
1e6
over-copied region
under-copied region
0
200
400
600
800
1000
0
1
2
3
4

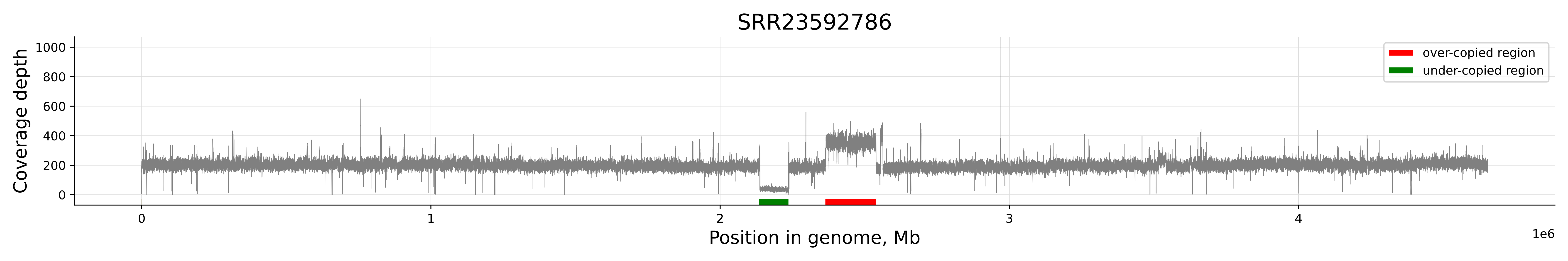

SRR23592786
over-copied region
under-copied region
Coverage depth
0
200
400
600
800
1000
0
1
2
3
4
1e6
Position in genome, Mb

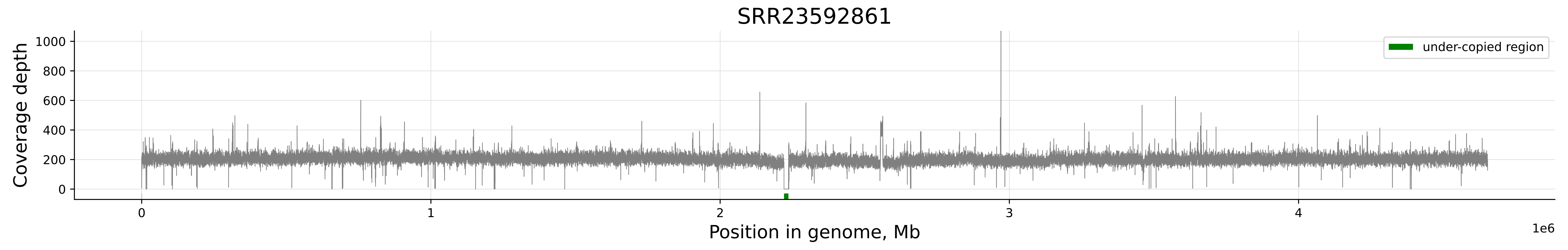

SRR23592861
under-copied region
Coverage depth
Position in genome, Mb
1e6
0
200
400
600
800
1000
0
1
2
3
4

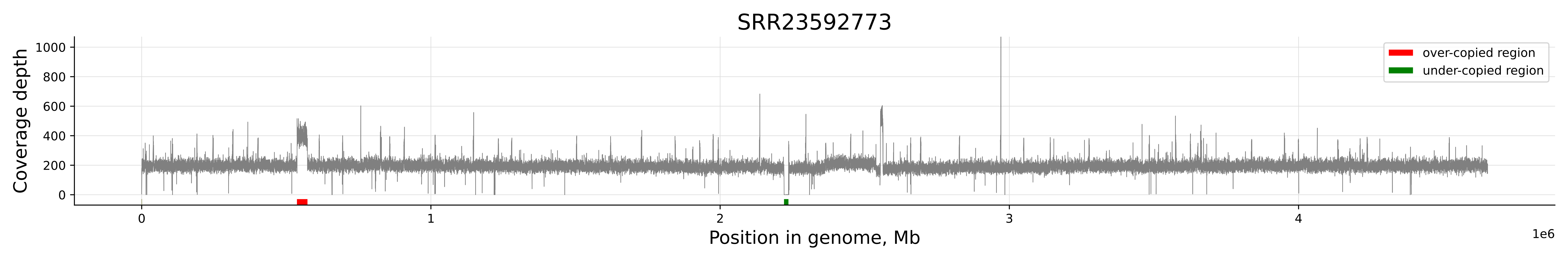

SRR23592773
over-copied region
under-copied region
Coverage depth
0
200
400
600
800
1000
0
1
2
3
4
1e6
Position in genome, Mb

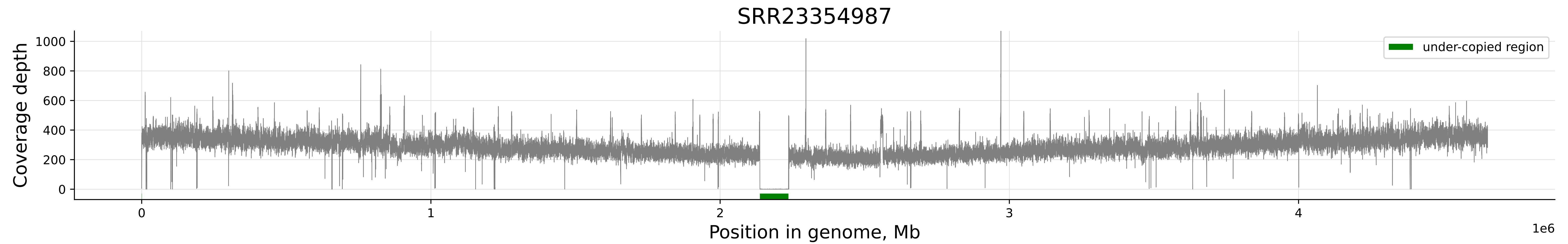
SRR23354987
Coverage depth
Position in genome, Mb
1e6
under-copied region
0
200
400
600
800
1000
0
1
2
3
4

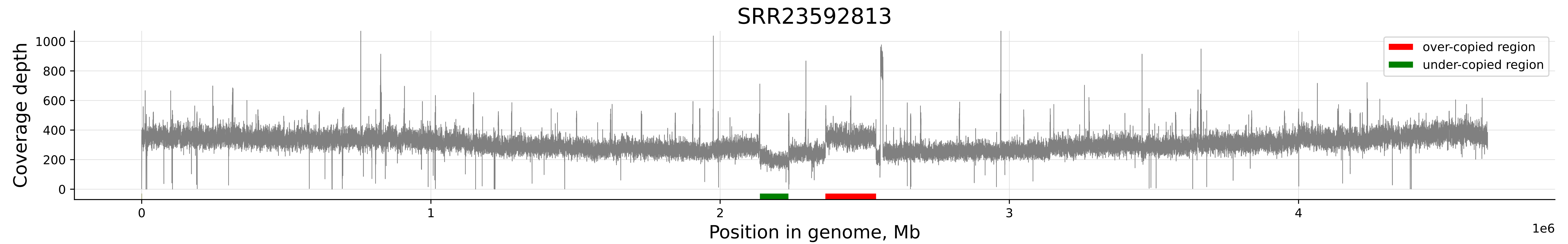

SRR23592813
over-copied region
under-copied region
Coverage depth
0
200
400
600
800
1000
0
1
2
3
4
1e6
Position in genome, Mb

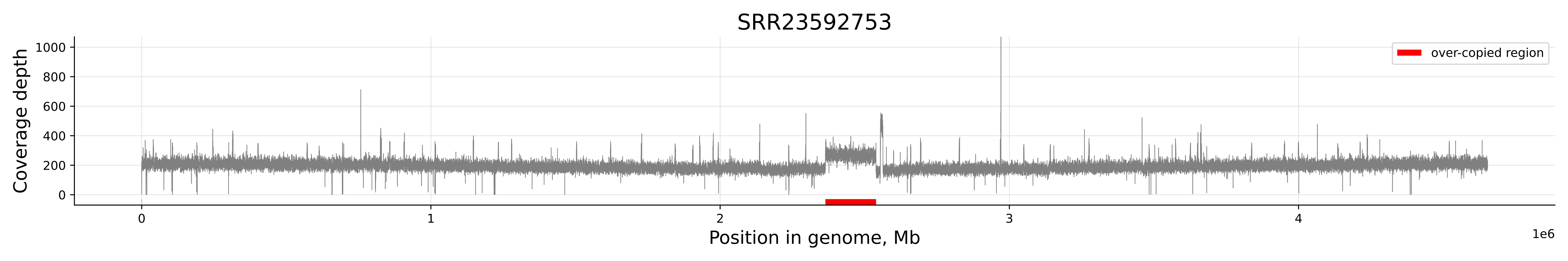

SRR23592753
Coverage depth
Position in genome, Mb
over-copied region
1000
800
600
400
200
0
0
1
2
3
4
1e6

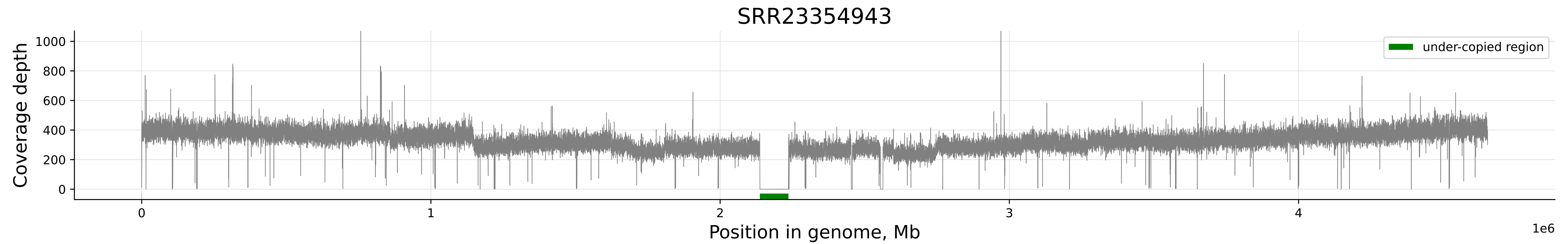
SRR23354943
Coverage depth
1000
800
600
400
200
0
0
1
2
3
4
1e6
Position in genome, Mb
under-copied region

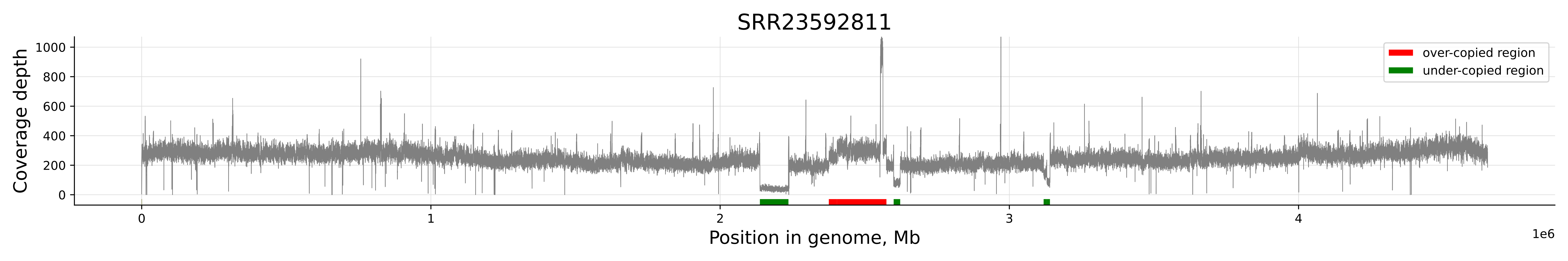

SRR23592811
Coverage depth
1000
800
600
400
200
0
over-copied region
under-copied region
0
1
2
3
4
1e6
Position in genome, Mb

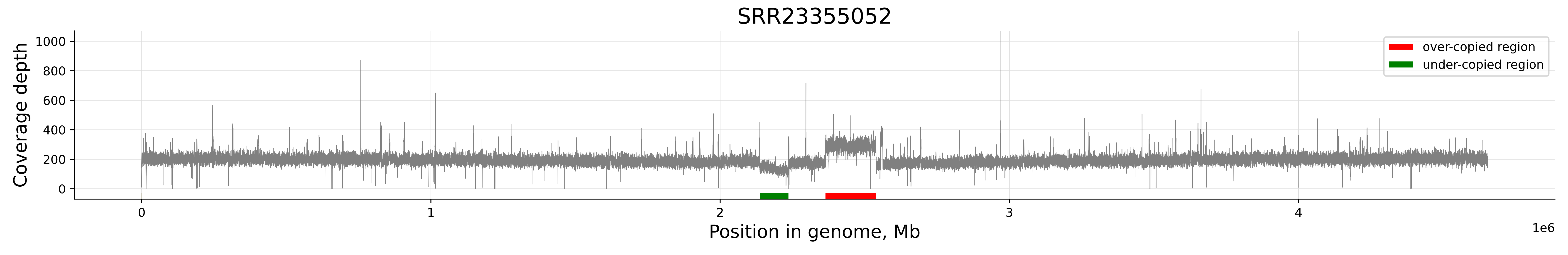

SRR23355052
Coverage depth
Position in genome, Mb
1e6
over-copied region
under-copied region
0
200
400
600
800
1000
0
1
2
3
4

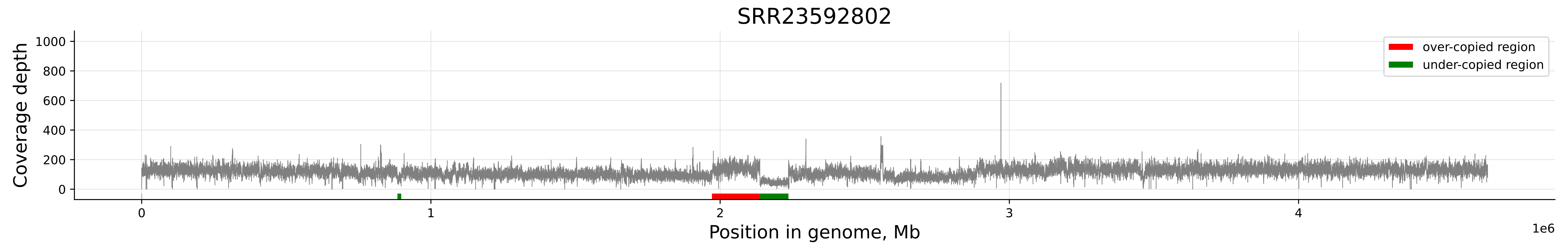

SRR23592802
Coverage depth
1000
800
600
400
200
0
over-copied region
under-copied region
0
1
2
3
4
1e6
Position in genome, Mb

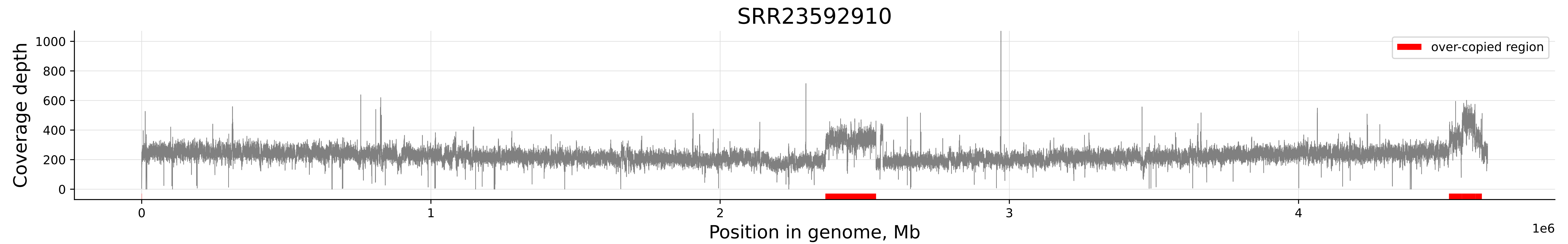
SRR23592910
Coverage depth
Position in genome, Mb
over-copied region
1e6
0
1
2
3
4
0
200
400
600
800
1000

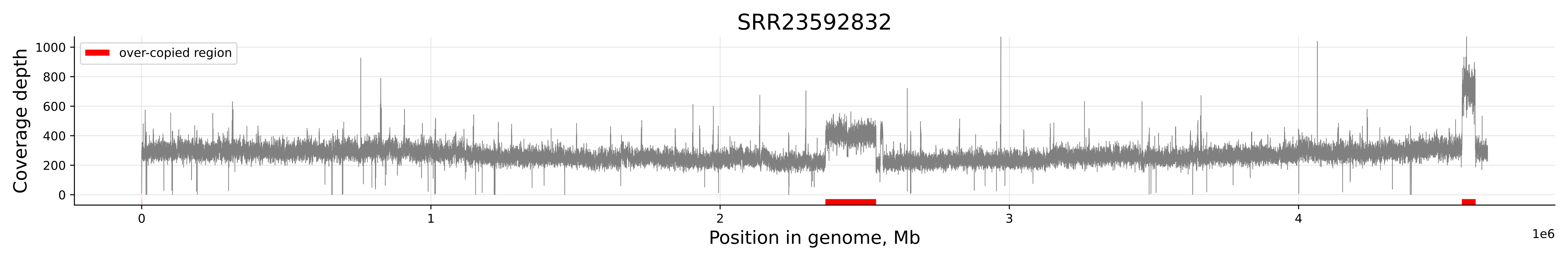

SRR23592832
over-copied region
Coverage depth
0
200
400
600
800
1000
0
1
2
3
4
1e6
Position in genome, Mb

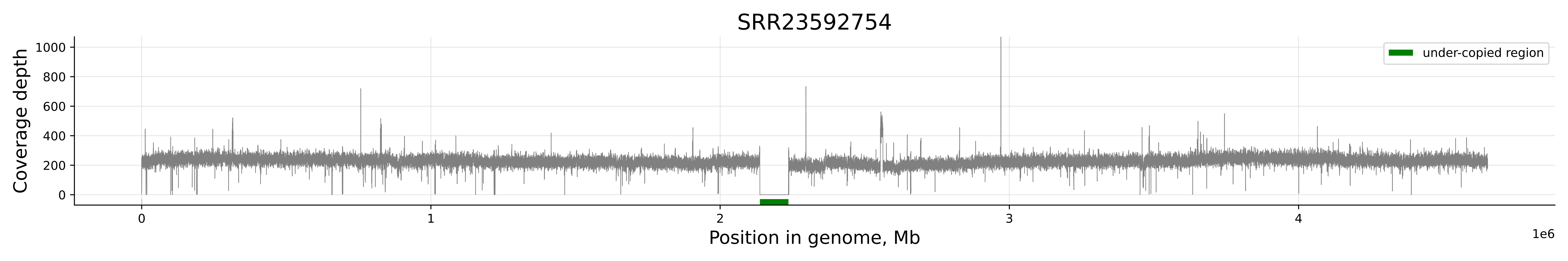
SRR23592754
Coverage depth
Position in genome, Mb
1e6
under-copied region
0
200
400
600
800
1000
0
1
2
3
4

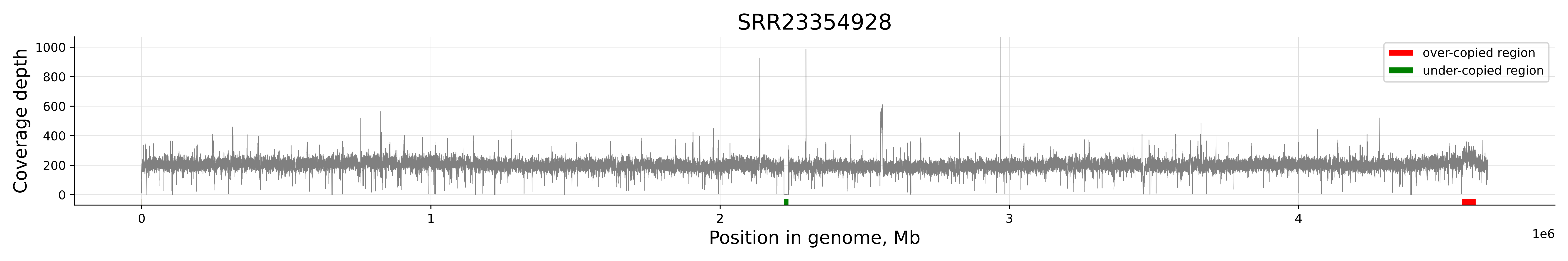

SRR23354928
Coverage depth
Position in genome, Mb
1e6
over-copied region
under-copied region
0
200
400
600
800
1000
0
1
2
3
4

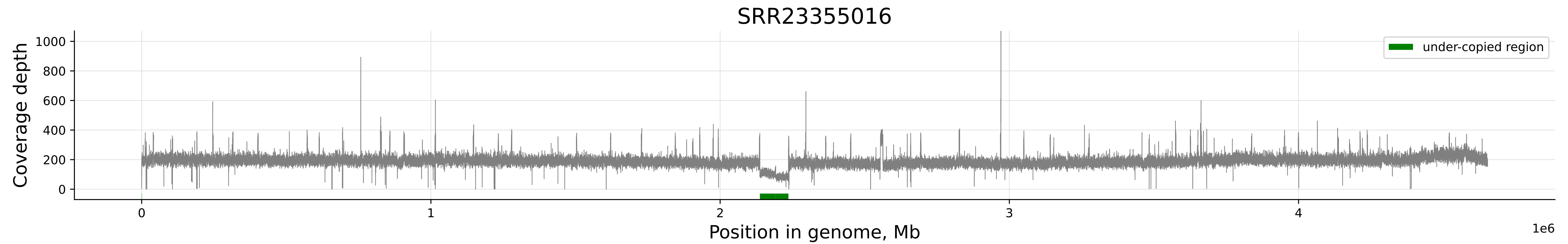
SRR23355016
under-copied region
Coverage depth
0
200
400
600
800
1000
0
1
2
3
4
1e6
Position in genome, Mb

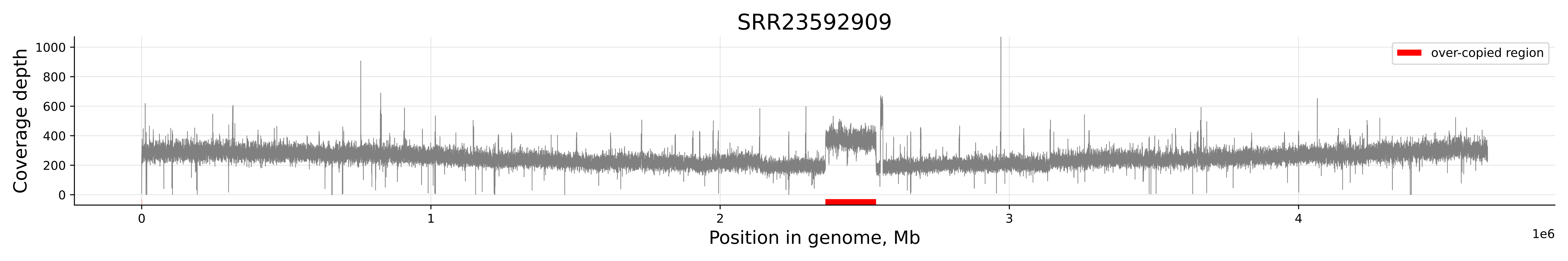
SRR23592909
Coverage depth
1000
800
600
400
200
0
over-copied region
0
1
2
3
4
1e6
Position in genome, Mb

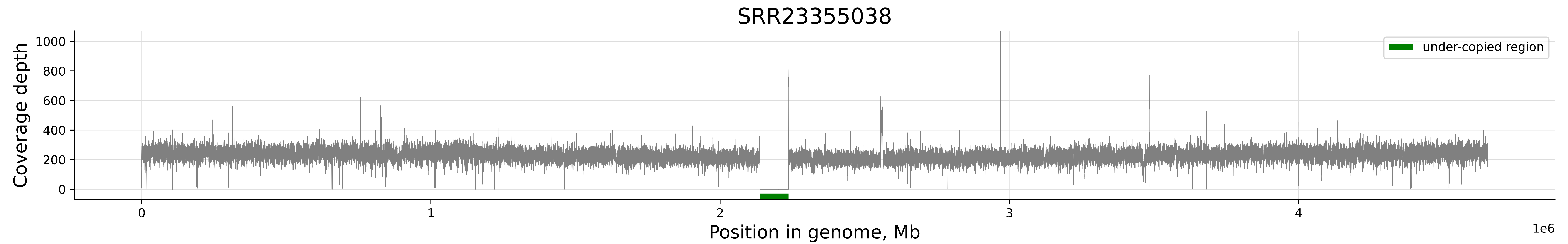

SRR23355038
under-copied region
Coverage depth
0
200
400
600
800
1000
0
1
2
3
4
1e6
Position in genome, Mb

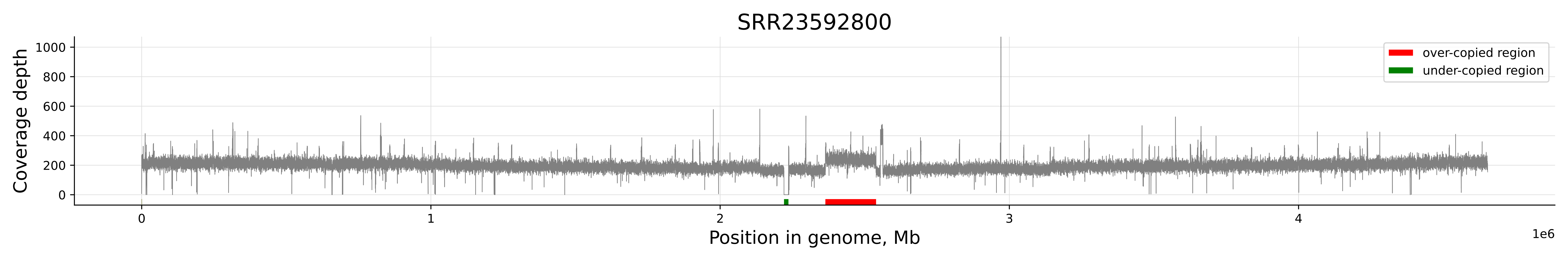

SRR23592800
Coverage depth
0
200
400
600
800
1000
0
1
2
3
4
1e6
Position in genome, Mb
over-copied region
under-copied region

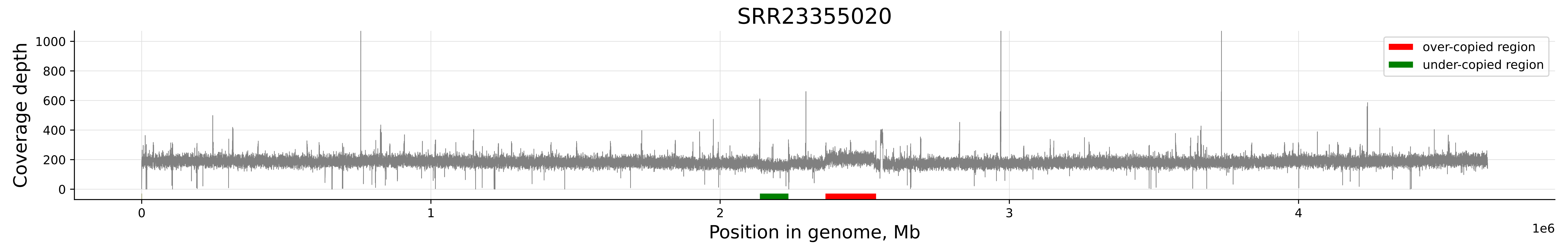
SRR23355020
Coverage depth
1000
800
600
400
200
0
0
1
2
3
4
1e6
Position in genome, Mb
over-copied region
under-copied region

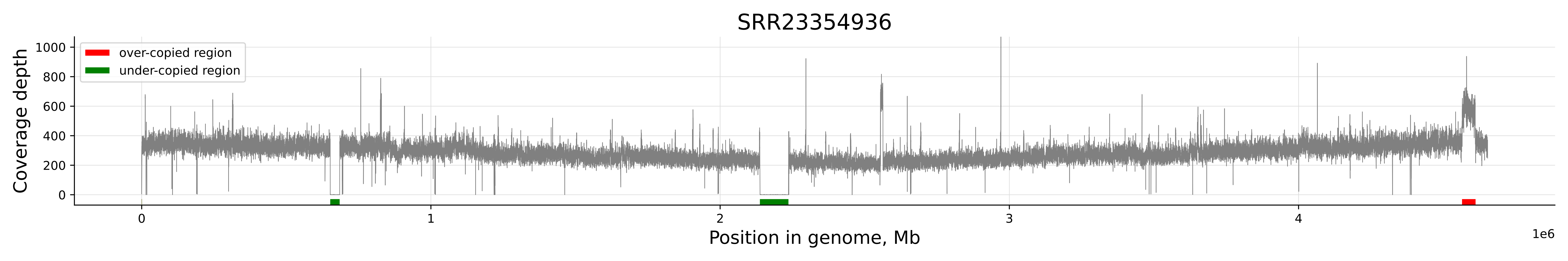

SRR23354936
over-copied region
under-copied region
Coverage depth
Position in genome, Mb
1e6
0
200
400
600
800
1000
0
1
2
3
4

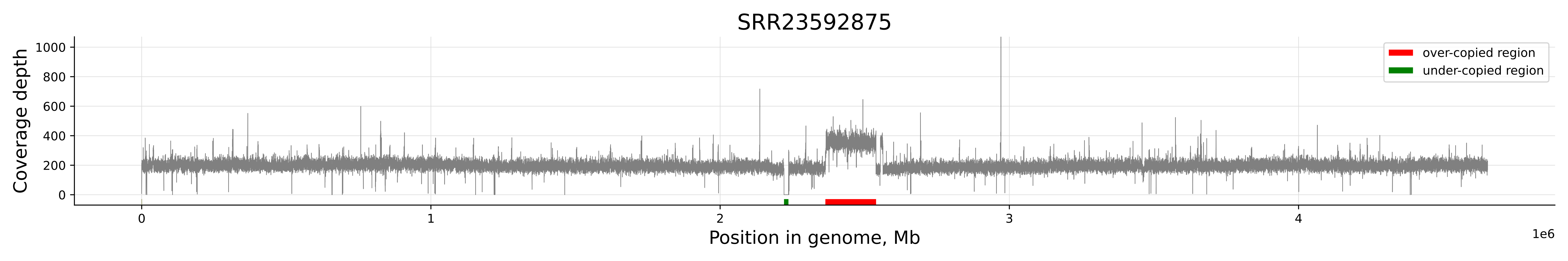

SRR23592875
Coverage depth
Position in genome, Mb
1e6
over-copied region
under-copied region
0
200
400
600
800
1000
0
1
2
3
4

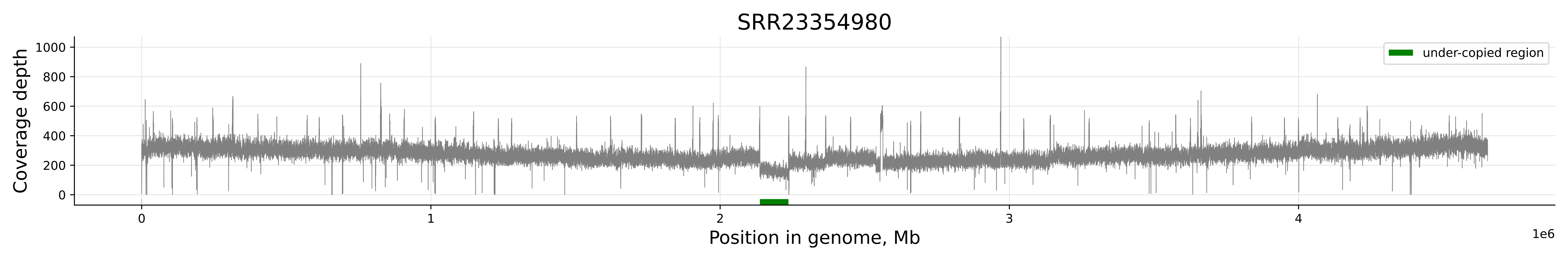

SRR23354980
Coverage depth
Position in genome, Mb
1e6
under-copied region
0
200
400
600
800
1000
0
1
2
3
4

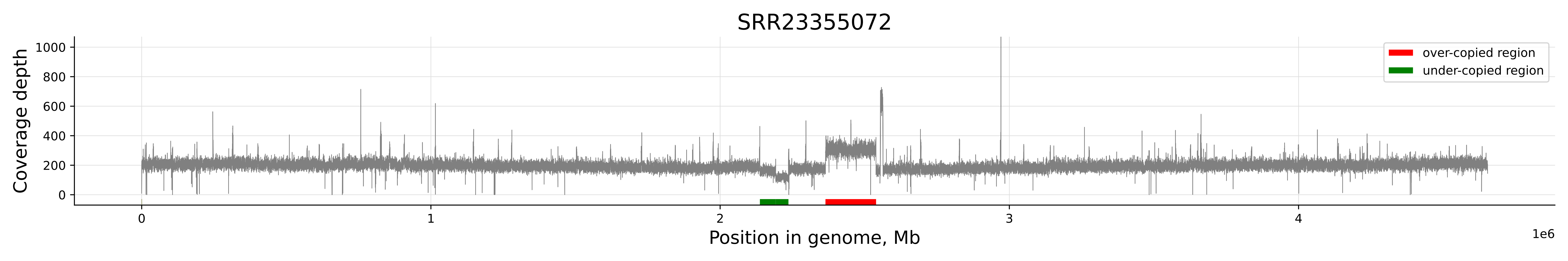
SRR23355072
Coverage depth
0
200
400
600
800
1000
0
1
2
3
4
1e6
Position in genome, Mb
over-copied region
under-copied region

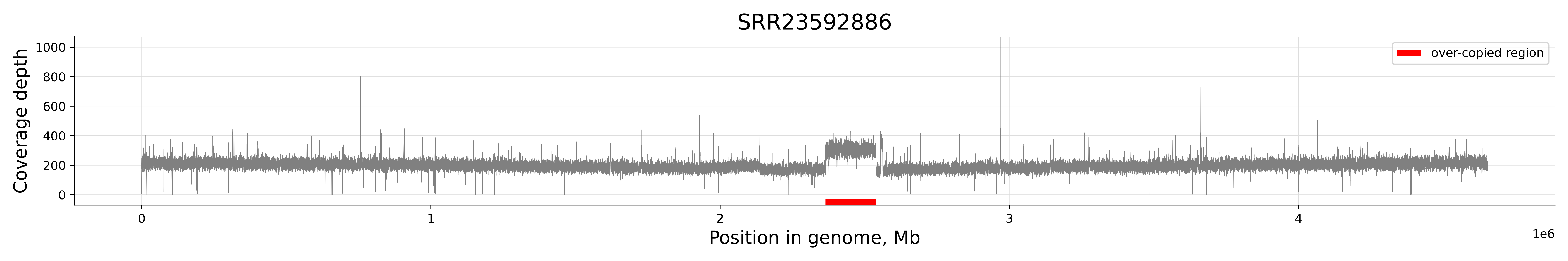
SRR23592886
over-copied region
Coverage depth
1000
800
600
400
200
0
0
1
2
3
4
1e6
Position in genome, Mb

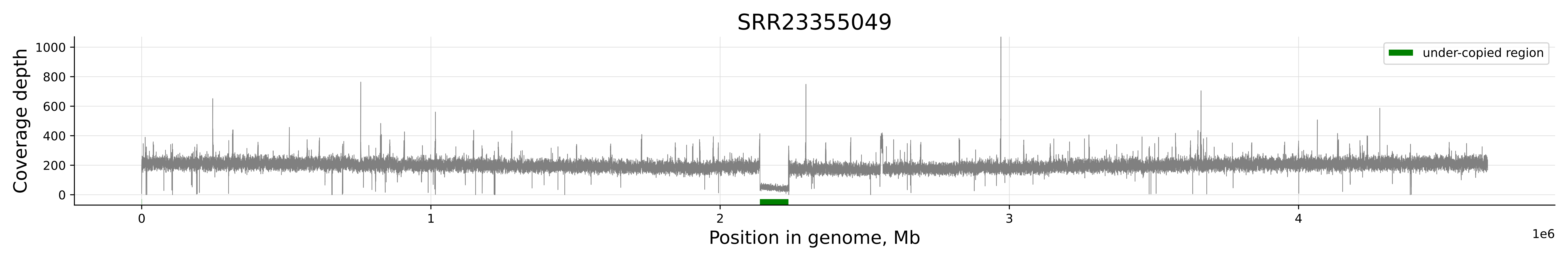

SRR23355049
under-copied region
Coverage depth
0
200
400
600
800
1000
0
1
2
3
4
1e6
Position in genome, Mb

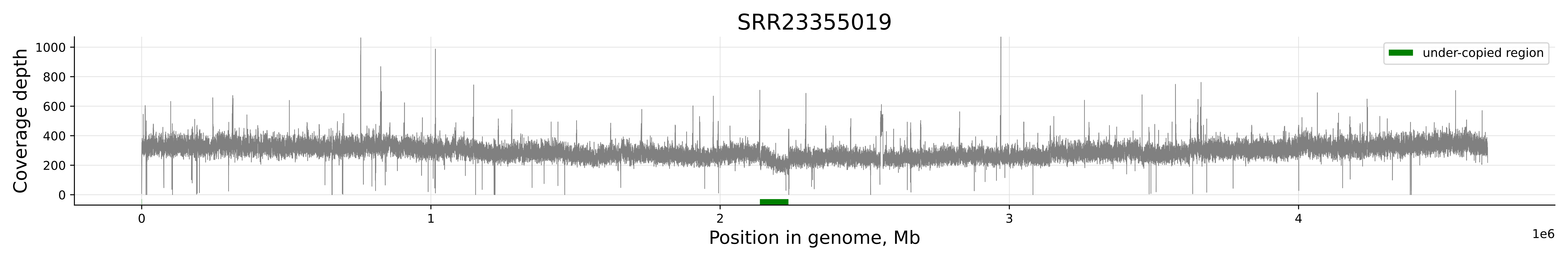

SRR23355019
Coverage depth
Position in genome, Mb
under-copied region
1e6
0
200
400
600
800
1000
1
2
3
4

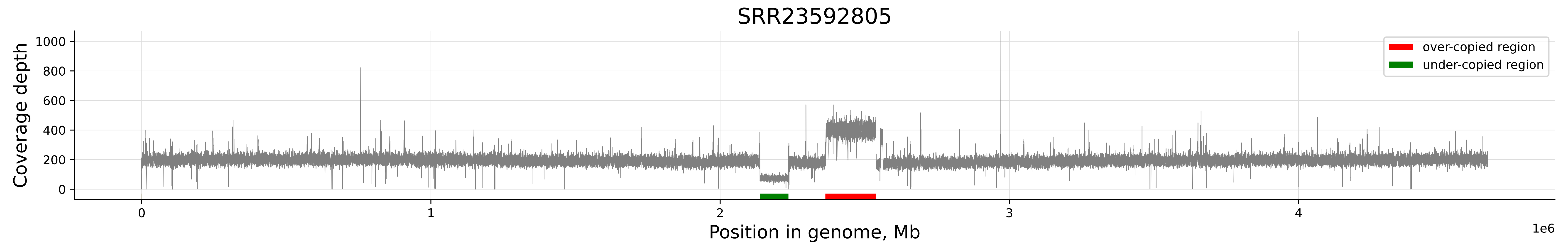

SRR23592805
Coverage depth
1000
800
600
400
200
0
over-copied region
under-copied region
0
1
2
3
4
1e6
Position in genome, Mb

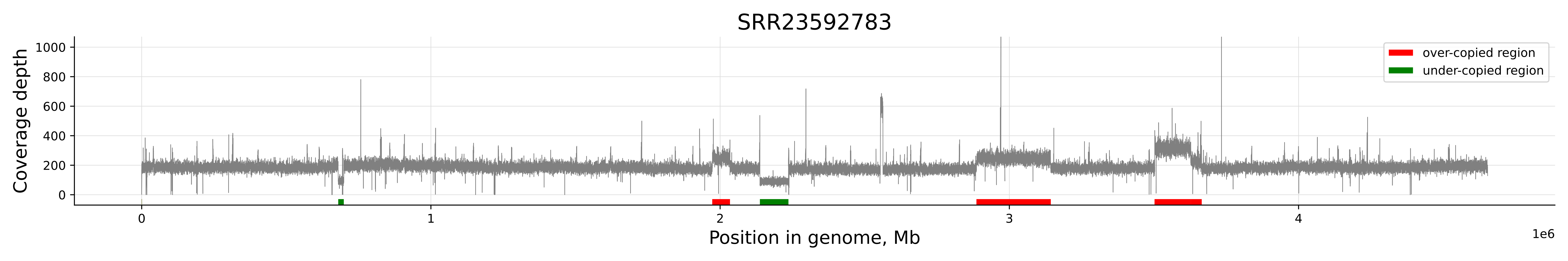
SRR23592783
Coverage depth
Position in genome, Mb
1e6
over-copied region
under-copied region
0
200
400
600
800
1000
0
1
2
3
4

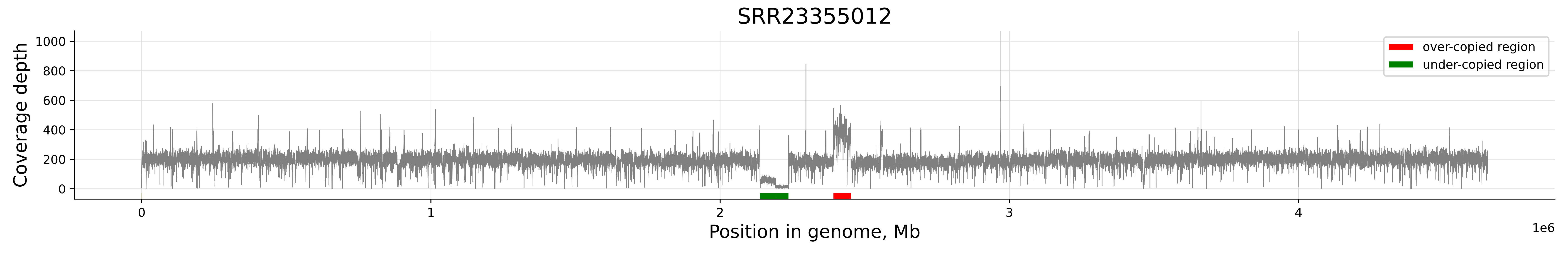

SRR23355012
Coverage depth
Position in genome, Mb
1e6
over-copied region
under-copied region
0
200
400
600
800
1000
0
1
2
3
4

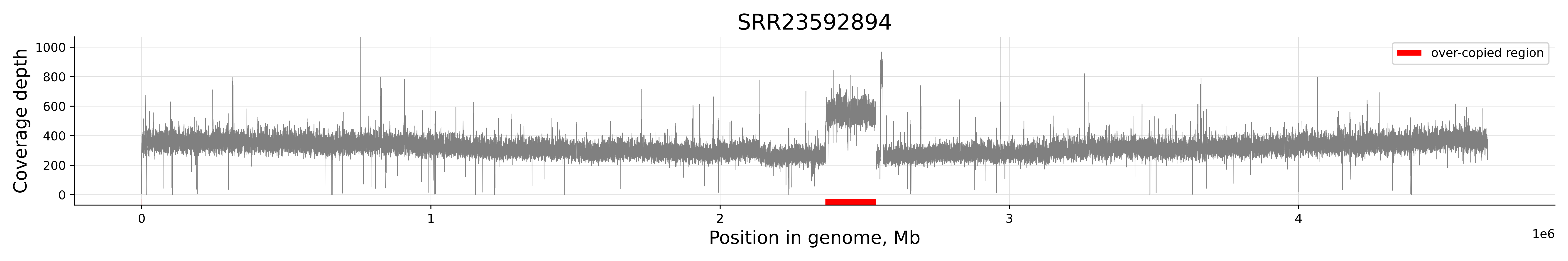

SRR23592894
Coverage depth
Position in genome, Mb
1e6
over-copied region
0
200
400
600
800
1000
0
1
2
3
4

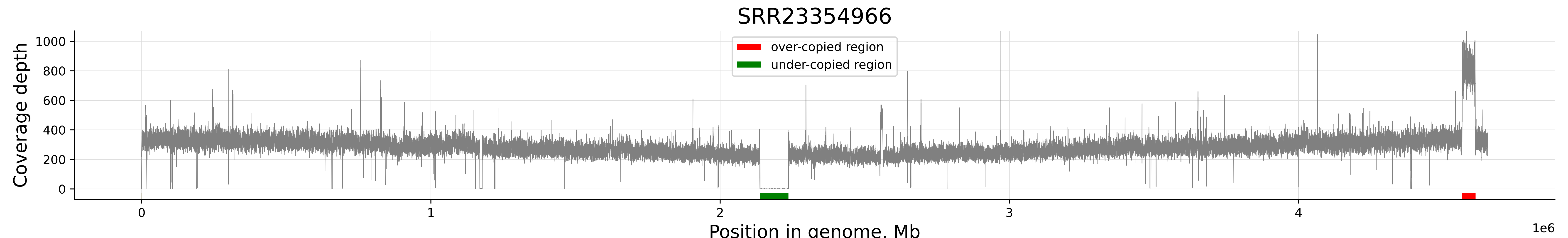

SRR23354966
over-copied region
under-copied region
Coverage depth
Position in genome, Mb
1e6
0
200
400
600
800
1000
0
1
2
3
4

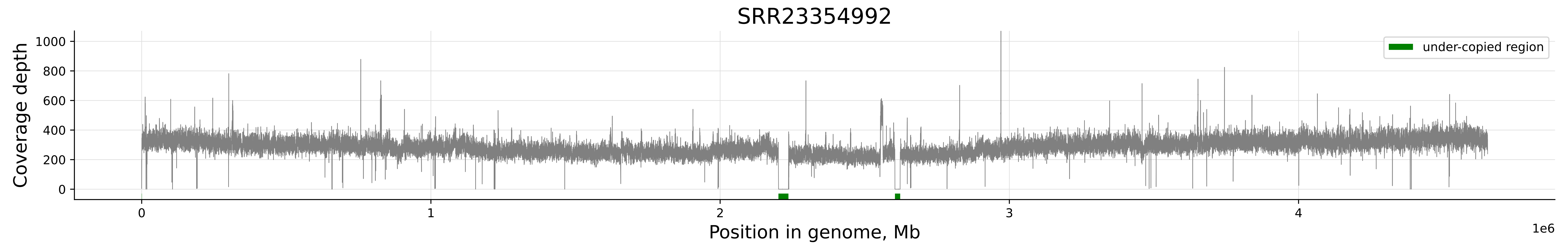
SRR23354992
under-copied region
Coverage depth
0
200
400
600
800
1000
Position in genome, Mb
0
1
2
3
4
1e6

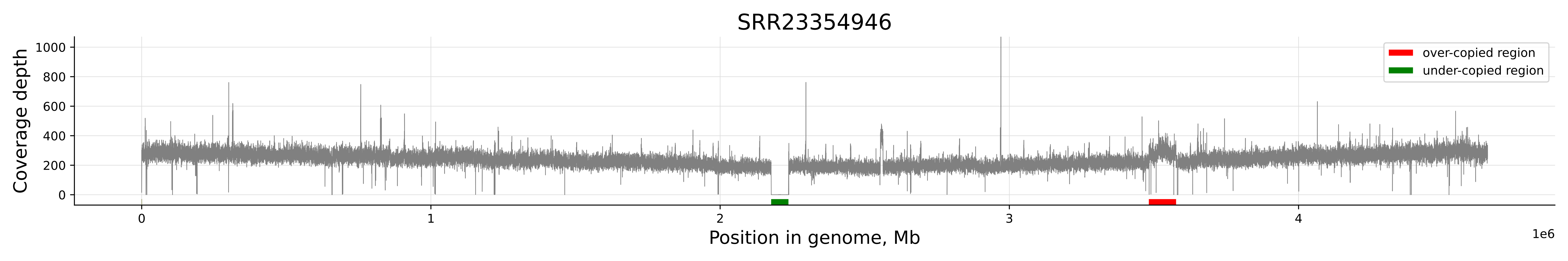

SRR23354946
Coverage depth
1000
800
600
400
200
0
0
1
2
3
4
1e6
Position in genome, Mb
over-copied region
under-copied region

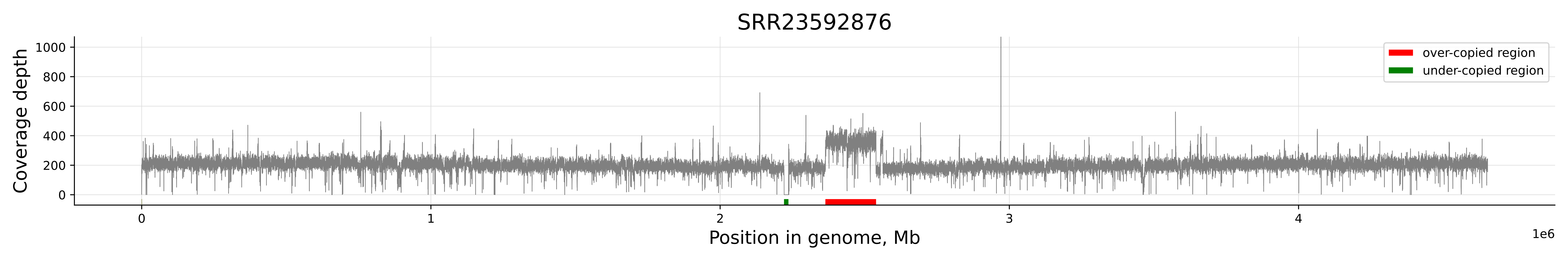

SRR23592876
Coverage depth
1000
800
600
400
200
0
over-copied region
under-copied region
0
1
2
3
4
1e6
Position in genome, Mb

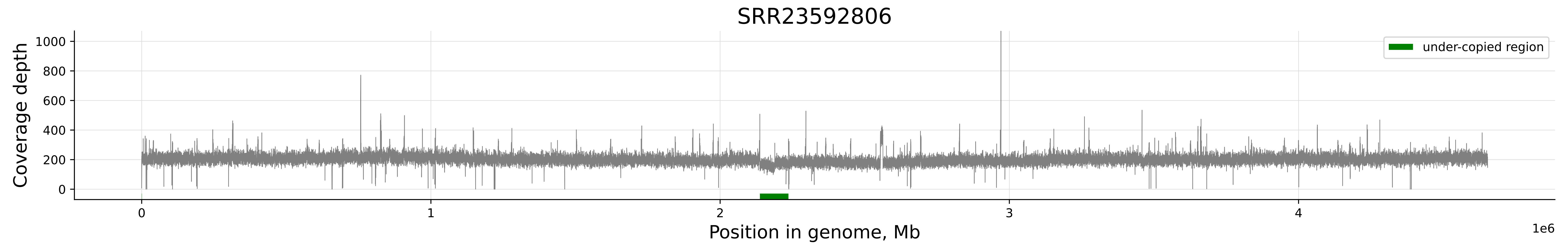

SRR23592806
under-copied region
Coverage depth
0
200
400
600
800
1000
0
1
2
3
4
1e6
Position in genome, Mb

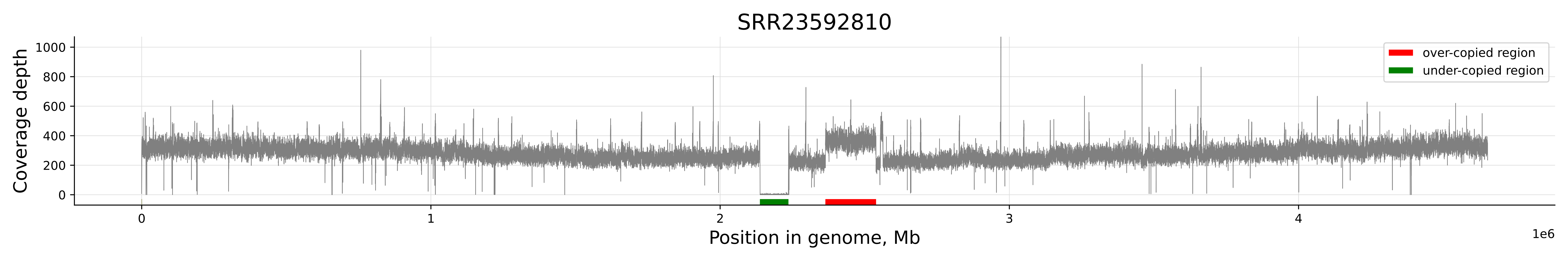

SRR23592810
Coverage depth
Position in genome, Mb
1e6
over-copied region
under-copied region
0
200
400
600
800
1000
0
1
2
3
4

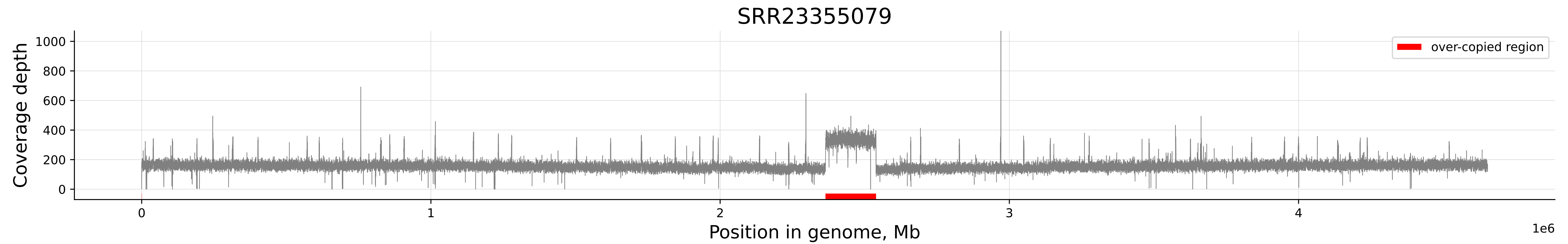

SRR23355079
over-copied region
Coverage depth
0
200
400
600
800
1000
0
1
2
3
4
1e6
Position in genome, Mb

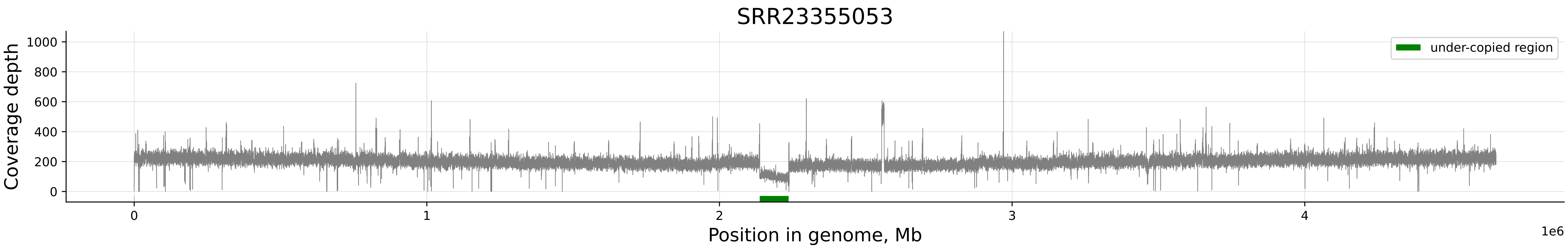
SRR23355053
Coverage depth
Position in genome, Mb
under-copied region
1e6
0
200
400
600
800
1000
0
1
2
3
4

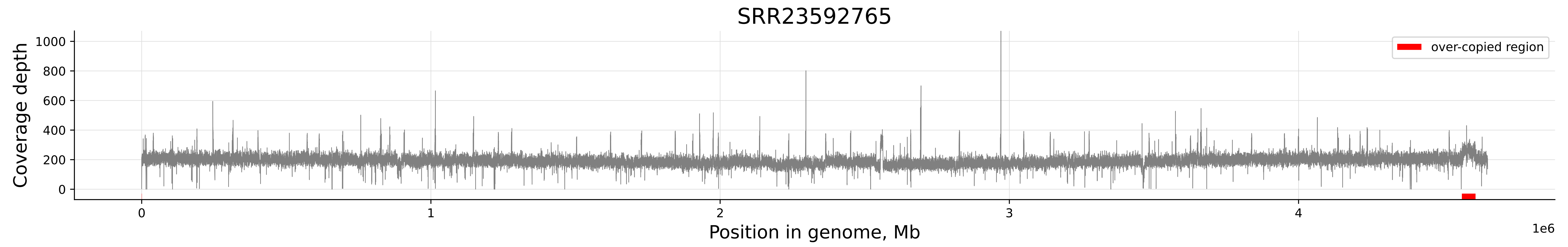

SRR23592765
over-copied region
Coverage depth
1000
800
600
400
200
0
Position in genome, Mb
0
1
2
3
4
1e6

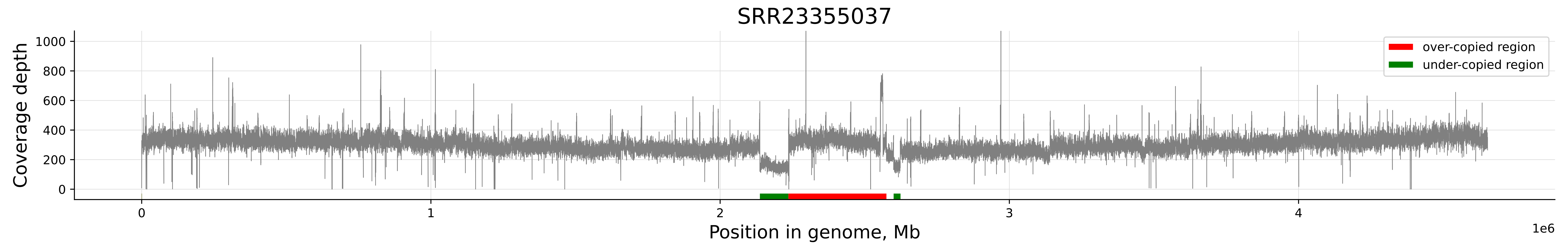

SRR23355037
Coverage depth
Position in genome, Mb
1e6
over-copied region
under-copied region
0
200
400
600
800
1000
1
2
3
4

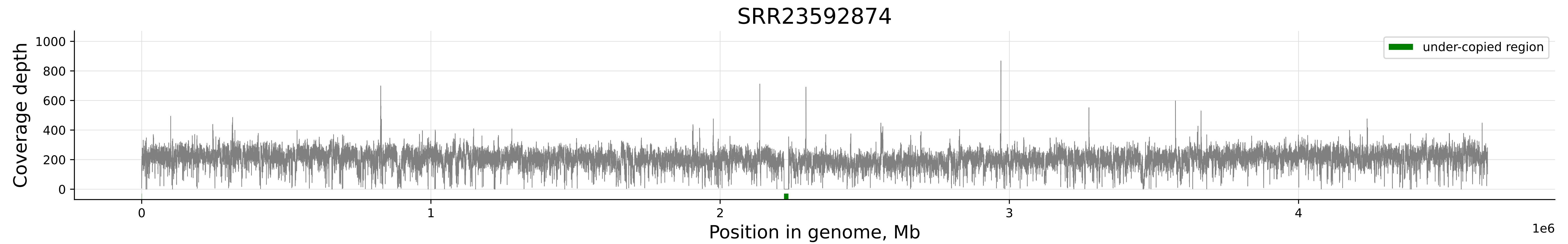

SRR23592874
under-copied region
Coverage depth
0
200
400
600
800
1000
0
1
2
3
4
1e6
Position in genome, Mb

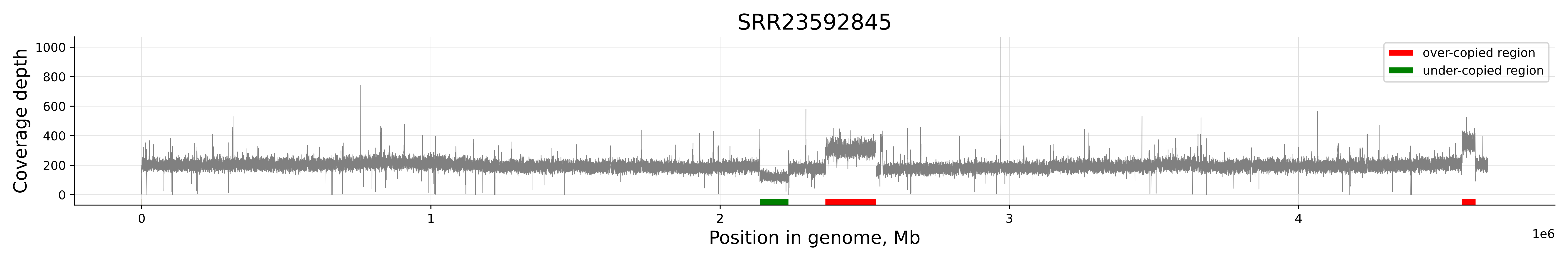

SRR23592845
Coverage depth
0
200
400
600
800
1000
over-copied region
under-copied region
0
1
2
3
4
1e6
Position in genome, Mb

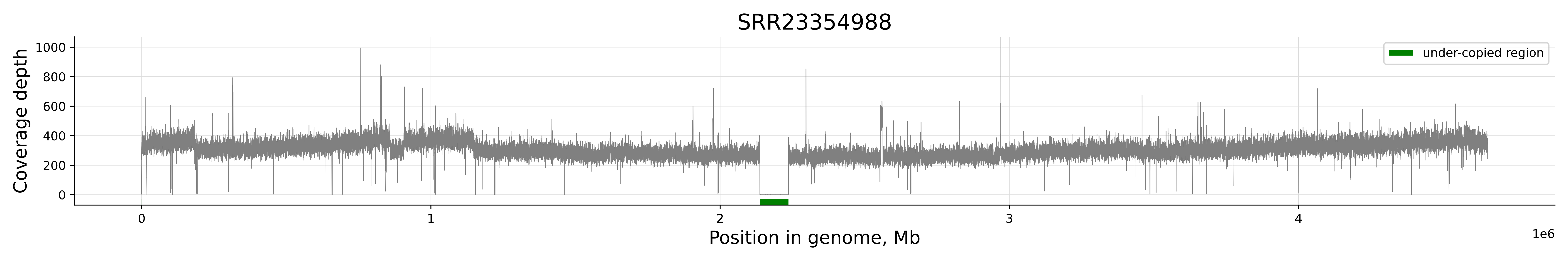

SRR23354988
under-copied region
Coverage depth
1000
800
600
400
200
0
0
1
2
3
4
1e6
Position in genome, Mb

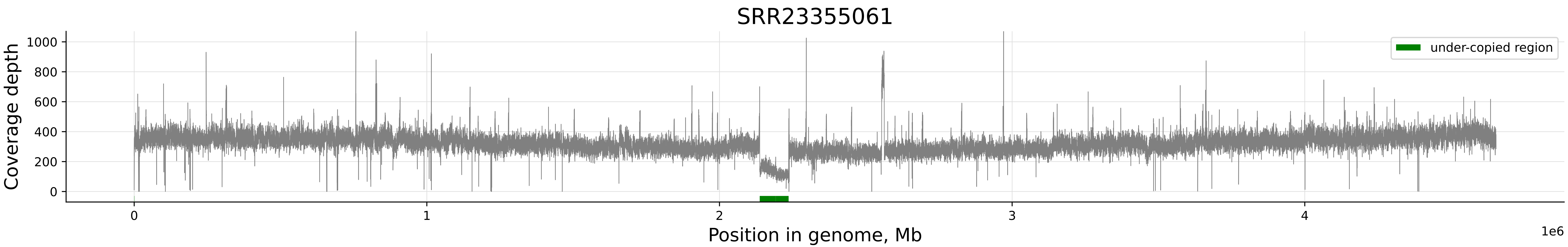
SRR23355061
under-copied region
Coverage depth
0
200
400
600
800
1000
0
1
2
3
4
1e6
Position in genome, Mb

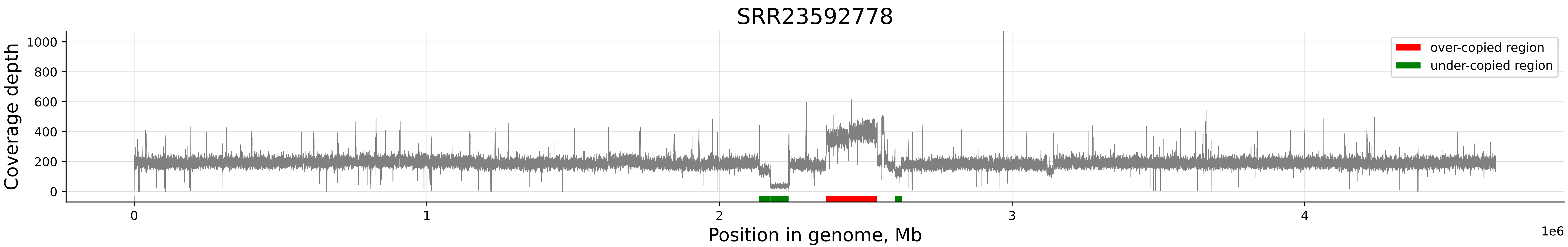

SRR23592778
over-copied region
under-copied region
Coverage depth
0
200
400
600
800
1000
0
1
2
3
4
1e6
Position in genome, Mb

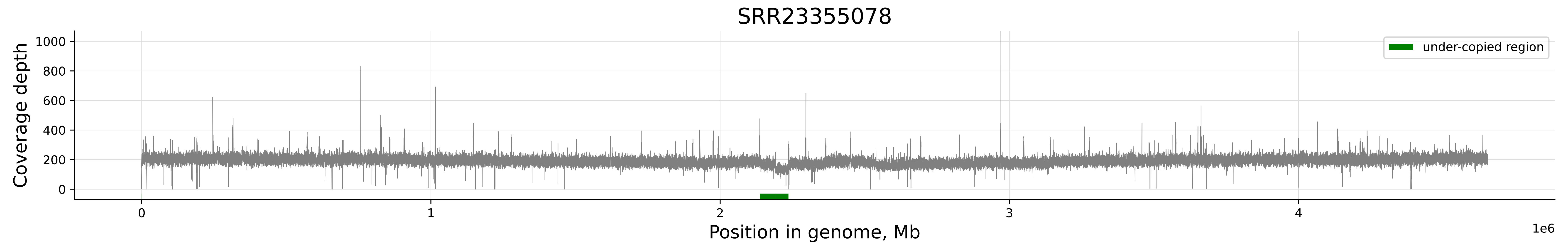

SRR23355078
under-copied region
Coverage depth
1000
800
600
400
200
0
0
1
2
3
4
1e6
Position in genome, Mb

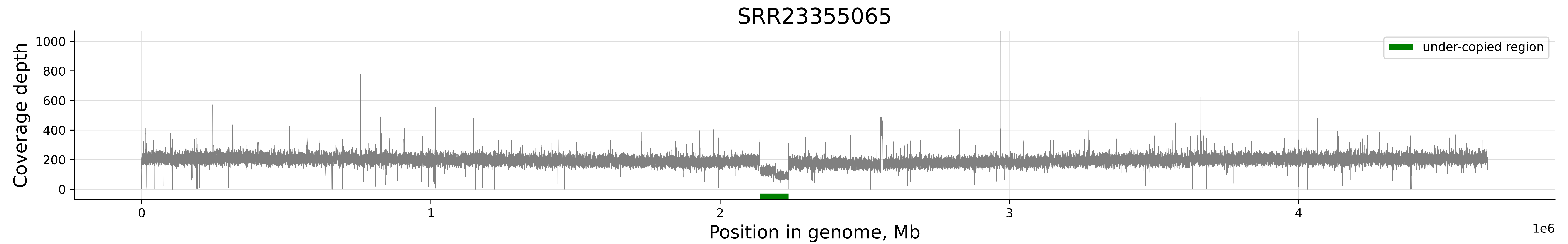

SRR23355065
Coverage depth
1000
800
600
400
200
0
under-copied region
0
1
2
3
4
1e6
Position in genome, Mb

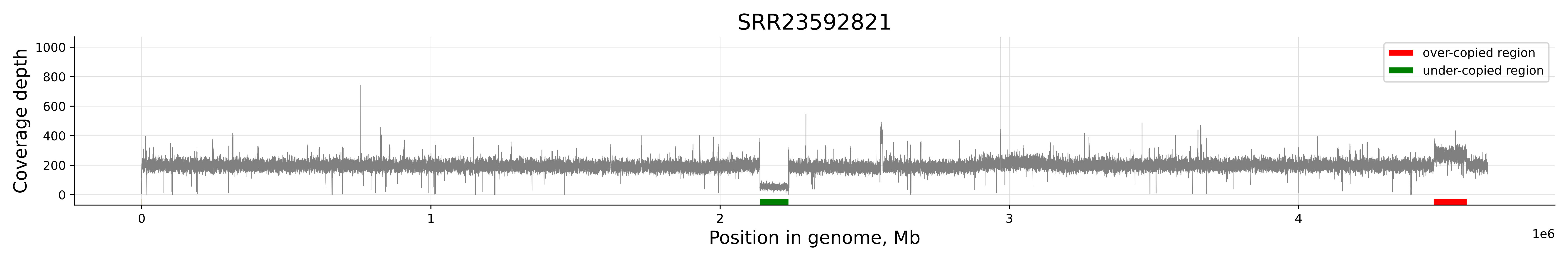

SRR23592821
Coverage depth
1000
800
600
400
200
0
0
1
2
3
4
1e6
Position in genome, Mb
over-copied region
under-copied region

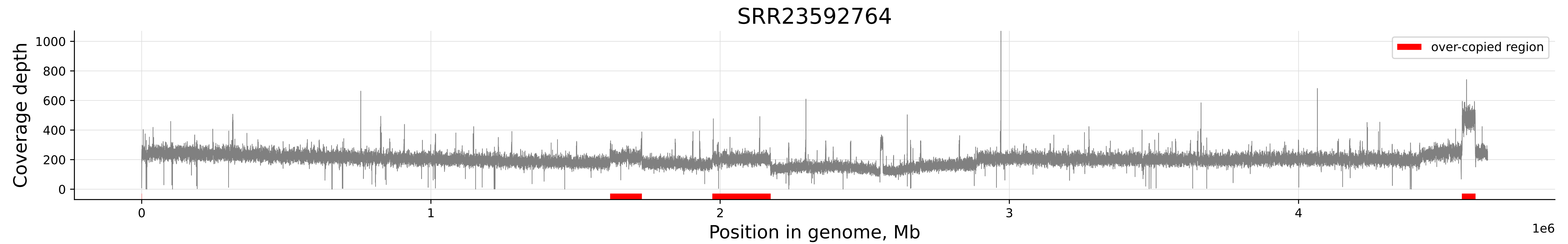

SRR23592764
Coverage depth
Position in genome, Mb
1e6
over-copied region
0
200
400
600
800
1000
0
1
2
3
4

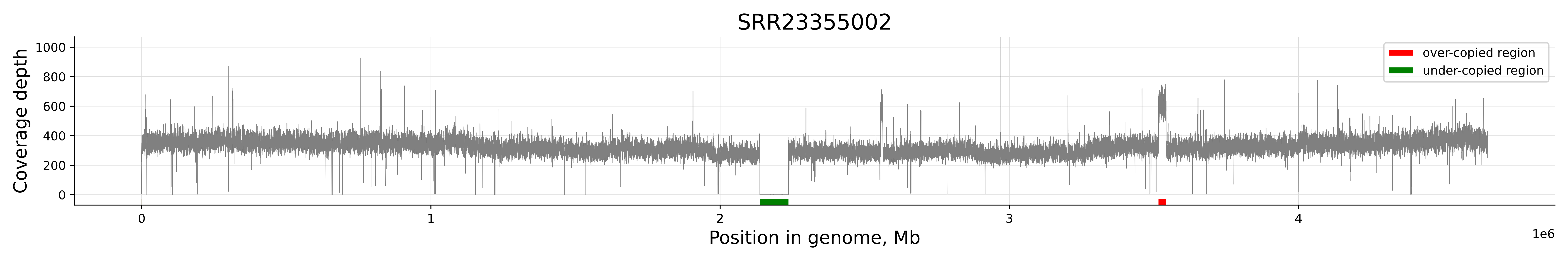

SRR23355002
Coverage depth
Position in genome, Mb
1e6
over-copied region
under-copied region
0
200
400
600
800
1000
0
1
2
3
4

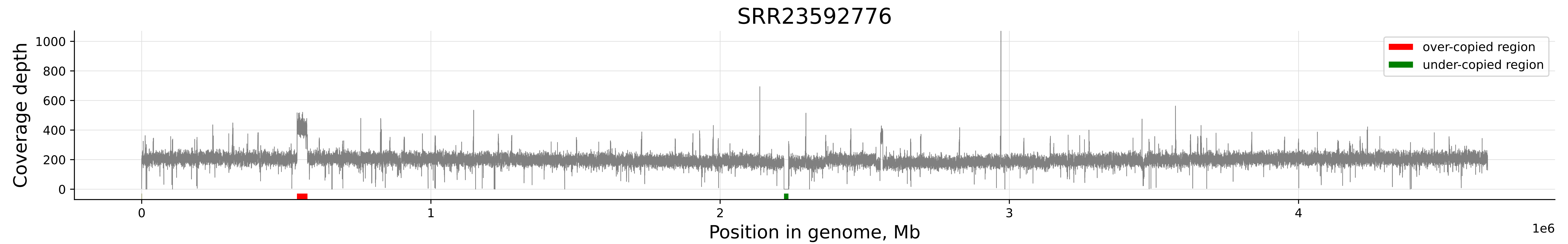
SRR23592776
Coverage depth
Position in genome, Mb
1e6
over-copied region
under-copied region
0
200
400
600
800
1000
0
1
2
3
4
